# Variation in the COVID-19 infection–fatality ratio by age, time, and geography during the pre-vaccine era: a systematic analysis

**DOI:** 10.1016/S0140-6736(21)02867-1

**Published:** 2022-04-16

**Authors:** COVID-19 Forecasting Team

## Abstract

**Background:**

The infection–fatality ratio (IFR) is a metric that quantifies the likelihood of an individual dying once infected with a pathogen. Understanding the determinants of IFR variation for COVID-19, the disease caused by the SARS-CoV-2 virus, has direct implications for mitigation efforts with respect to clinical practice, non-pharmaceutical interventions, and the prioritisation of risk groups for targeted vaccine delivery. The IFR is also a crucial parameter in COVID-19 dynamic transmission models, providing a way to convert a population's mortality rate into an estimate of infections.

**Methods:**

We estimated age-specific and all-age IFR by matching seroprevalence surveys to total COVID-19 mortality rates in a population. The term total COVID-19 mortality refers to an estimate of the total number of deaths directly attributable to COVID-19. After applying exclusion criteria to 5131 seroprevalence surveys, the IFR analyses were informed by 2073 all-age surveys and 718 age-specific surveys (3012 age-specific observations). When seroprevalence was reported by age group, we split total COVID-19 mortality into corresponding age groups using a Bayesian hierarchical model to characterise the non-linear age pattern of reported deaths for a given location. To remove the impact of vaccines on the estimated IFR age pattern, we excluded age-specific observations of seroprevalence and deaths that occurred after vaccines were introduced in a location. We estimated age-specific IFR with a non-linear meta-regression and used the resulting age pattern to standardise all-age IFR observations to the global age distribution. All IFR observations were adjusted for baseline and waning antibody-test sensitivity. We then modelled age-standardised IFR as a function of time, geography, and an ensemble of 100 of the top-performing covariate sets. The covariates included seven clinical predictors (eg, age-standardised obesity prevalence) and two measures of health system performance. Final estimates for 190 countries and territories, as well as subnational locations in 11 countries and territories, were obtained by predicting age-standardised IFR conditional on covariates and reversing the age standardisation.

**Findings:**

We report IFR estimates for April 15, 2020, to January 1, 2021, the period before the introduction of vaccines and widespread evolution of variants. We found substantial heterogeneity in the IFR by age, location, and time. Age-specific IFR estimates form a J shape, with the lowest IFR occurring at age 7 years (0·0023%, 95% uncertainty interval [UI] 0·0015–0·0039) and increasing exponentially through ages 30 years (0·0573%, 0·0418–0·0870), 60 years (1·0035%, 0·7002–1·5727), and 90 years (20·3292%, 14·6888–28·9754). The countries with the highest IFR on July 15, 2020, were Portugal (2·085%, 0·946–4·395), Monaco (1·778%, 1·265–2·915), Japan (1·750%, 1·302–2·690), Spain (1·710%, 0·991–2·718), and Greece (1·637%, 1·155–2·678). All-age IFR varied by a factor of more than 30 among 190 countries and territories. After age standardisation, the countries with the highest IFR on July 15, 2020, were Peru (0·911%, 0·636–1·538), Portugal (0·850%, 0·386–1·793), Oman (0·762%, 0·381–1·399), Spain (0·751%, 0·435–1·193), and Mexico (0·717%, 0·426–1·404). Subnational locations with high IFRs also included hotspots in the UK and southern and eastern states of the USA. Sub-Saharan African countries and Asian countries generally had the lowest all-age and age-standardised IFRs. Population age structure accounted for 74% of logit-scale variation in IFRs estimated for 39 in-sample countries on July 15, 2020. A post-hoc analysis showed that high rates of transmission in the care home population might account for higher IFRs in some locations. Among all countries and territories, we found that the median IFR decreased from 0·466% (interquartile range 0·223–0·840) to 0·314% (0·143–0·551) between April 15, 2020, and Jan 1, 2021.

**Interpretation:**

Estimating the IFR for global populations helps to identify relative vulnerabilities to COVID-19. Information about how IFR varies by age, time, and location informs clinical practice and non-pharmaceutical interventions like physical distancing measures, and underpins vaccine risk stratification. IFR and mortality risk form a J shape with respect to age, which previous research, such as that by Glynn and Moss in 2020, has identified to be a common pattern among infectious diseases. Understanding the experience of a population with COVID-19 mortality requires consideration for local factors; IFRs varied by a factor of more than 30 among 190 countries and territories in this analysis. In particular, the presence of elevated age-standardised IFRs in countries with well resourced health-care systems indicates that factors beyond health-care capacity are important. Potential extenuating circumstances include outbreaks among care home residents, variable burdens of severe cases, and the population prevalence of comorbid conditions that increase the severity of COVID-19 disease. During the pre-vaccine period, the estimated 33% decrease in median IFR over 8 months suggests that treatment for COVID-19 has improved over time. Estimating IFR for the pre-vaccine era provides an important baseline for describing the progression of COVID-19 mortality patterns.

**Funding:**

Bill & Melinda Gates Foundation, J Stanton, T Gillespie, and J and E Nordstrom

## Introduction

Understanding variation in the ratio of COVID-19 deaths to SARS-CoV-2 infections remains a crucially important metric for medical professionals and policy makers alike. We define the infection–fatality ratio (IFR) as the probability of an individual dying from pathogen-related disease complications once infected with a pathogen. Fatality is a distinct concept from mortality, with the latter describing the occurrence of deaths among all members of a population. COVID-19 mortality and fatality patterns by age, time, and location inform risk stratification in clinical case management,^1^ policy design, and implementation, and strategies for increasing vaccination uptake.^2^, ^3^ The age structure of the population has been suggested to be a contributor to the lower mortality rates observed in some low-income and middle-income countries,^4^ because these countries generally have younger age distributions.^5^ Adjusting for a population's age structure gives a clearer picture of the relative COVID-19 burden between countries and provides a better basis for assessing the effectiveness of interventions. Measuring variation in the age pattern of COVID-19 mortality in conjunction with the age-specific IFR can also provide important insights into the amount of community transmission by age group. For a given number of observed deaths, a lower IFR implies that more infections have occurred and a population's inferred immunity is greater.^6^, ^7^ COVID-19 mortality and IFR, including how they vary by age, are therefore crucial inputs for dynamic transmission models that attempt to describe the conditions that lead to herd immunity.^8^ Whether through clinical case management or prophylaxis, reducing the IFR is a primary goal of the global medical community. All other things being equal, IFR reductions would indicate that a person who is infected has a lower probability of dying. The IFR is thus a key metric for tracking whether medical advances that reduce the severity of disease are improving health outcomes over time.

**Research in context**

**Evidence before this study**

Previous meta-analyses have found that the infection-fatality ratio (IFR) for COVID-19 varies substantially between locations, with much of the variation attributable to differences in population age structure. Brazeau and colleagues (October, 2020) estimated IFRs of 0·23% for hypothetical populations with the age structure of a typical low-income country and 1·15% for those with an age structure of a typical high-income country. Levin and colleagues (December, 2020) estimated that 90% of variation in the IFR among countries from the Organisation for Economic Co-operation and Development was explainable by age. Meyerowitz-Katz and Merone (July, 2020) and Ioannidis (September, 2020) estimated IFRs of 0·68% and 0·27% in their respective samples, but did not account for age structure of the source populations. When mortality and IFR were estimated conditional on age, most meta-analyses assessed age effects with a log-linear model. To our knowledge, only O'Driscoll and colleagues (November, 2020) used a non-linear estimation method; they found that the age effects of mortality and IFR are both J shaped, with the lowest risk occurring among children aged 5–9 years. They note consistent age effects to age 65 years and heterogeneity in the age effect for older ages, probably attributable to different experiences and reporting practices for nursing homes and long-term care facilities.

**Added value of this study**

First, our search strategy yielded a larger and more globally representative set of seroprevalence studies than previous meta-analyses. This analysis includes 2073 all-age seroprevalence studies from 53 countries and 718 age-specific surveys from 36 countries. Second, we used mortality rates that represent an estimate of total COVID-19 mortality, rather than reported death counts, to inform the numerator of the IFR. Using total COVID-19 mortality reduced variation in the IFR because of differences across locations in ascertaining or assigning COVID-19 deaths. Third, in addition to baseline sensitivity, we corrected seroprevalence surveys for waning sensitivity of each specific anti-nucleocapsid and anti-spike antibody test. Fourth, our method for estimating mortality age patterns can accommodate heterogeneous age groups, and its hierarchical cascade structure allows information about non-linear patterns to be shared across locations. This method improves the estimates required to convert age-specific seroprevalence observations into age-specific IFR observations. Fifth, indirect age standardisation allows for meaningful comparisons of IFR across locations and expanded the set of observations that could be used to fit models of the age-standardised IFR. Sixth, the Bayesian nature of the age-standardised IFR model enables results from patient-level analyses to be incorporated into the estimation process. Seventh, to our knowledge, this is the first study to estimate global and location-specific change in the IFR over time.

**Implications of all the available evidence**

Our age-specific analysis of the IFR showed that the risk of death among people infected with COVID-19 is J shaped with respect to age, a result consistent with that of O'Driscoll and colleagues (2020). Policies and prevention efforts operating on the assumption that risk increases monotonically with age are underemphasising the risk of death for young children. Our all-age and age-standardised analyses of IFR both show substantial heterogeneity for locations around the world. This finding has profound implications for vaccine prioritisation rubrics, which should take into consideration comorbidities, variants, and other location-specific factors in addition to age. Confounding by clinical predictors of the IFR, such as obesity, complicates the use of death rates to assess the effectiveness of countries in responding to the pandemic.

Several meta-analyses of the COVID-19 IFR have been done since the beginning of the pandemic. Taken together, these studies suggest that the all-age IFR can vary by more than an order of magnitude, with much of this variation caused by differences in population age structure. Brazeau and colleagues^9^ estimated that the IFR ranges from 0·14% to 0·42% in low-income countries and 0·78% to 1·79% in high-income countries. Meyerowitz-Katz and Merone^10^ and Ioannidis^11^ estimated IFRs of 0·68% and 0·27% in their respective samples, but did not account for age structure of the source populations. Some meta-analyses estimated IFR conditional on age by matching age-disaggregated seroprevalence data to COVID-19 mortality. Using a log-linear model, Levin and colleagues^12^ estimated that the IFR is 0·001% at age 5 years and 8·4% at age 80 years, which means that the IFR was more than 8000 times greater at age 80 years than at age 5 years. O'Driscoll and colleagues^13^ used a non-linear estimation method; they found that the age effects of mortality and IFR are both J shaped, with the lowest risk occurring among children aged 5–9 years. Although these studies have been helpful in characterising the credible range of the IFR, they have often used coarse age groups (such as younger and older than 65 years), represented age groups as midpoints, or imposed an assumption of log linearity in the relationship between IFR and age.

In this study, we estimated how the IFR for COVID-19 varied by age, time, and geography during the pre-vaccine era. Epidemiological patterns were relatively stable during this period compared with the subsequent phase of the pandemic, which has been characterised by the heterogeneous rollout of vaccines and the rise of new variants.^14^ As such, these results provide an important baseline that describes COVID-19 risks in the absence of extenuating factors. Because of the pervasive under-reporting of COVID-19 deaths, we used an estimate of the true number of COVID-19 deaths in a population (ie, total COVID-19 mortality) as the numerator of the IFR.^15^, ^16^ We also used large databases of COVID-19 mortality rates for detailed age groups and published or released seroprevalence studies. In combination with inputs from the COVID-19 project at the Institute for Health Metrics and Evaluation, we used this information to estimate how the infection-fatality ratio varies by age, time, and geography, with adjustments for waning antibody-test sensitivity (unpublished methods, COVID-19 Cumulative Infections Collaborators), under-reporting of deaths,^15^, ^16^ and other known biases. In addition to time-invariant age-specific IFR, we modelled time-varying all-age and age-standardised IFR for 190 countries and territories and many subnational regions as defined by the Global Burden of Diseases, Injuries, and Risk Factors Study (GBD).^17^

## Methods

### Overview

This study has six key steps. First, we used available age-specific COVID-19 mortality data to estimate relative mortality age patterns that vary by location. Second, we matched seroprevalence observations to an estimate of total COVID-19 mortality occurring in the source population at the time. Total COVID-19 mortality was derived from location-specific estimates of excess mortality and the fraction of excess mortality attributable to COVID-19. When seroprevalence was disaggregated by age, we used the estimated mortality age patterns to split total COVID-19 mortality into corresponding age groups. Third, with the resulting age-specific IFR observations, we fit a non-linear meta-regression model to make age-specific estimates of the IFR. Fourth, we used this global IFR age pattern to standardise all-age IFR observations to the global age distribution. All IFR observations were adjusted for baseline and waning antibody-test sensitivity. Fifth, with a set of Bayesian meta-regression models that form a geographical cascade structure, we modelled age-standardised IFR as a function of time, seven clinical predictors of the IFR, and two measures of health system performance. The covariates were implemented as an ensemble model including the 100 top-performing covariate sets. Sixth, we predicted age-standardised IFRs conditional on covariates for global locations and reversed the age-standardisation to obtain estimates of all-age IFR. Finally, to quantify the potential impact of care home epidemics, we did a post-hoc analysis considering locations with especially high care home mortality relative to the older community-dwelling population.

This study complies with the Guidelines for Accurate and Transparent Health Estimates Reporting recommendations (appendix 1 section 2).^18^ All code used in the analysis can be found online on Github.

### Mortality by age

We sourced age-specific mortality data primarily through the COVerAGE-DB database of COVID-19 cases and deaths by age,^8^, ^19^ with additional supplementation via national and state ministry of health dashboards where identified. In total, we obtained data from 213 locations and used the most recent observation of cumulative deaths before vaccine introduction in each location. National-level age-specific mortality data were available for 64 countries (appendix 2 section 2; appendix 3 for date of reported cumulative deaths by location). We analysed the data using a hierarchical Bayesian implementation of B-splines. This method involves fitting a global age pattern to the logit of the age-specific reported mortality rates, then using the estimated spline coefficients as priors in models that are fit to region-specific subsets of the data. The coefficients of the region-specific models were subsequently used as priors in models fit to national and administrative level one (such as states and provinces) subsets of the data. The priors were stronger for locations with relatively few total deaths or if the data had little age granularity. When making predictions for locations without age-specific reported deaths, we used the model from the lowest-available level of the geographical hierarchy. The underlying meta-regression package MRTool^20^ is designed to accommodate data reported as heterogeneous age groups. On the basis of the observation that age effect is negative at younger ages and then becomes positive, we allowed the spline to take any shape for ages younger than 10 years and then required that the function rise monotonically with age. For age groups in the data with zero deaths observed, we combined adjacent age groups so that no age interval had zero deaths (appendix 1 section 5.2).

### Infection-fatality ratio by age

We identified seroprevalence studies through a search protocol that leveraged previous reviews,^11^, ^12^ SeroTracker,^21^ and routine inclusion of national and subnational surveys undertaken by governmental organisations. The search protocol yielded 5131 seroprevalence surveys. Of these, 1099 surveys occurred before vaccine introduction in a location and disaggregated the results by age. For consistency with the age-standardised IFR model, we further excluded seroprevalence surveys for which the source population was not geoaccordant with a national or administrative level one location or were otherwise determined to be an outlier (appendix 1 section 5.4) later in the analytic pipeline. This process led to a total of 3012 age-specific seroprevalence observations from 718 surveys in 36 countries (appendix 2 section 4). Nine studies had data exclusively for the under-5-year age group, including observations from Brazil, Jordan, Nepal, Norway, and Spain. Observations with greater risk of bias were marked for subsequent bias adjustment in this age-specific IFR model. To convert age-specific seroprevalence observations to IFR observations, we matched seroprevalence to the estimated number of total COVID-19 deaths^15^, ^16^ occurring in the population 9 days after the end of the survey. The period of 9 days corresponds to the expected length of time between seroconversion and death. Total COVID-19 mortality was estimated as the fraction of excess mortality attributable to COVID-19 (appendix 1 section 5.5). With the location-specific estimated mortality-age patterns and the population's age structure, we disaggregated total COVID-19 deaths into age groups corresponding to the age groups of the seroprevalence observations. The resulting age-specific IFR observations were adjusted for baseline and waning sensitivity of antibody tests. The formulas used in this step are described (appendix 1 section 5.3). Finally, using a Bayesian meta-regression framework,^20^ we estimated the non-linear age pattern of IFR using a B-spline with optimised knot locations. To minimise undue influence from studies with especially large sample sizes, we set each observation's measurement error to be the median SE in the dataset. We included an indicator variable in the model for whether an observation was derived from a sample with greater risk of bias and predicted for the reference level (appendix 1 section 5.3).

### Age-standardised and all-age IFR

Of the potential seroprevalence surveys, 2073 were derived from representative samples or otherwise met the inclusion criteria outlined in the appendix (appendix 1 section 5.4; inclusion criteria set by the COVID-19 Cumulative Infections Collaborators, unpublished). These seroprevalence observations were corrected for baseline and waning sensitivity of anti-nucleocapsid and anti-spike antibody tests, misclassification of infection status because of vaccination, and reinfection caused by escape variance (appendix 1 section 5.4). We matched these seroprevalence observations to an estimate of total COVID-19 mortality^15^, ^16^ occurring 9 days after the end date of the seroprevalence study. We standardised the resulting IFR observations to the global age distribution; inputs for indirect standardisation were the global age-specific IFR estimates from the previous step, global age-specific estimates of seroprevalence, and the age structure of the population. We then modelled logit-scale age-standardised IFR as a function of time in days and several location-level covariates. Observations were indexed in time according to the mean date of deaths occurring in the population before the survey. Information was shared across locations hierarchically in a set of Bayesian meta-regression models that form a geographical cascade structure. The assessed covariates included age-standardised prevalence of obesity, smoking, diabetes, chronic kidney disease, cancer, chronic obstructive pulmonary disease, cardiovascular disease, the Healthcare Access and Quality Index,^22^ and an index of universal health-care coverage.^23^ All unique combinations of predictors were evaluated, and the top 100 sets were included in an ensemble of 100 models. Clinical predictors were selected on the basis of a review of evidence by the US Centers for Disease Control and Prevention.^24^ Effect sizes for the clinical predictors were implemented as Gaussian priors on the basis of findings from an American Heart Association analysis of patients with COVID-19 in 107 US hospitals.^25^ The time effect was constrained to be monotonically decreasing and was informed by a Gaussian prior in the global stage of the cascade. The Gaussian prior was distributed as mean equal to –0.002 and standard deviation equal to 0.001. Models in the regional and location-specific stages of the cascade were specified without the prior. The inflection points at which the time effect became constant varied across models in the ensemble. More details about the methods for this model can be found in appendix 1 (section 5.4).

### Role of the funding source

The funders of the study had no role in the study design, data collection, data analysis, data interpretation, or the writing of the report.

## Results

**Figure 1:**
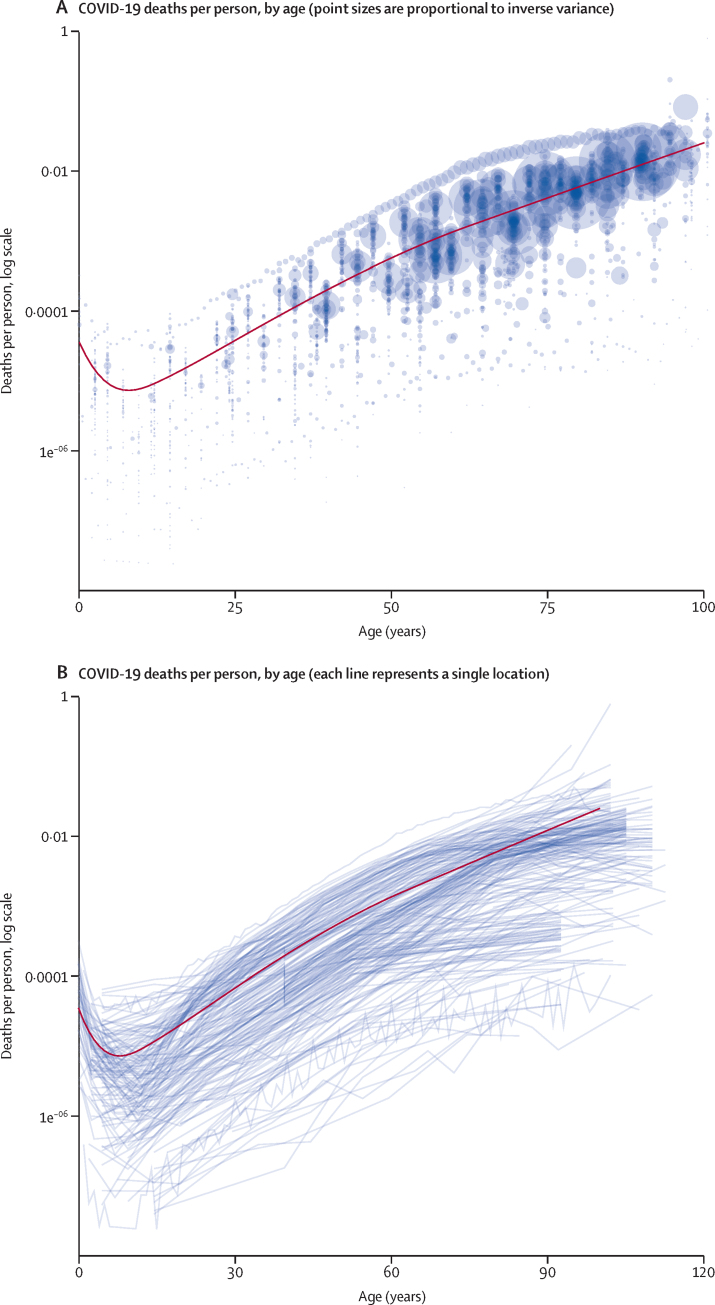
COVID-19 deaths per person by age COVID-19 deaths per person by age for the most recent observation before vaccine introduction in each location are shown.

**Figure 2:**
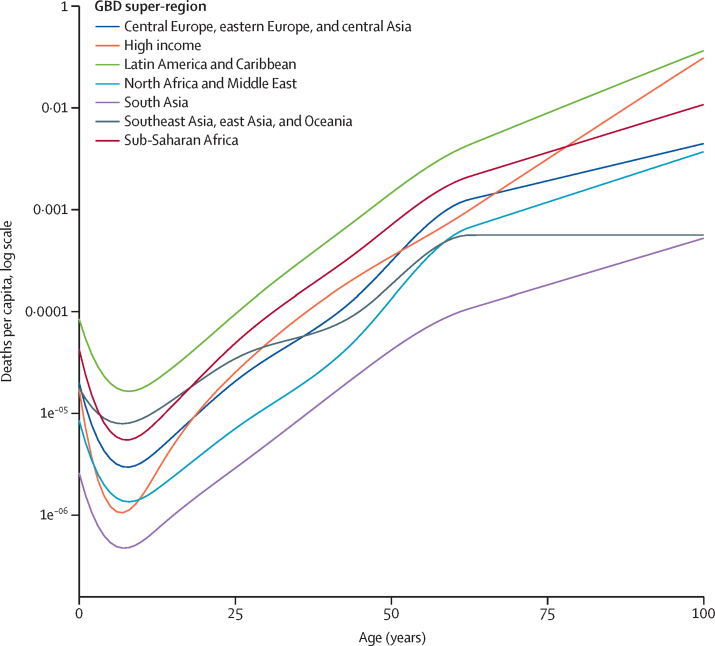
Age patterns of COVID-19 mortality by GBD super-region GBD=Global Burden of Diseases, Injuries, and Risk Factors Study.

We report IFR estimates for April 15, 2020, to Jan 1, 2021, the period before the introduction of vaccines and widespread evolution of variants. Globally, data on age-specific COVID-19 mortality formed a J-shaped pattern, with the lowest rates found in populations aged approximately 5 years to 10 years and progressively higher rates among younger and older populations (figure 1). Age midpoints connected with a line show that the slope of the prediction runs roughly parallel to age trends in the input data (figure 1B). Across GBD super-regions, age patterns of COVID-19 mortality were relatively consistent below age 60 years (figure 2). Age patterns exhibited greater variability above age 60 years, with the steepest age effect occurring in the high-income super-region. The flattest age effects occurred in the southeast Asia, east Asia, and Oceania super-region (note that these classifications exclude high-income countries). Age patterns above age 60 years varied considerably by location (appendix 3).

**Figure 3:**
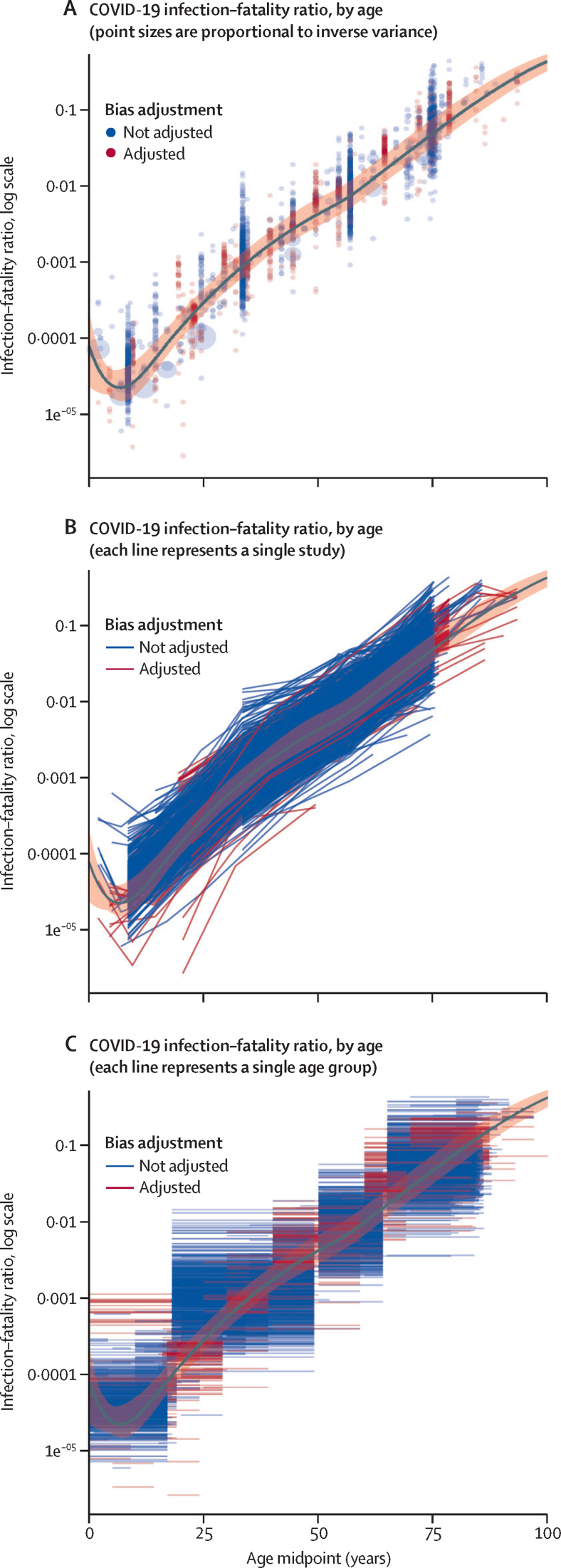
COVID-19 infection–fatality ratio by age

The age pattern of IFR was similarly J shaped (figure 3). The lowest IFR occurred at age 7 years and IFR increased in an approximately log-linear fashion among ages older than 30 years (table 1). The effect size for the risk of bias covariate was 0·00396 (SE 0·0493). The age-specific IFR 95% uncertainty interval (UI) incorporates uncertainty from the fixed-effect coefficients and γ, the parameter estimating the variance of between-study heterogeneity (orange shading in figure 3). The model fit follows the data and runs roughly parallel to within-study age trends (figure 3A, B). The observations can be further visualised as age intervals (figure 3C), an important way to assess fit to the data because the model integrates over the whole span of an age interval.

**Table 1:** COVID-19 IFR estimates by age

	**IFR and 95% uncertainty interval**
1 year	0·0054% (0·0021–0·0114)
2 years	0·0040% (0·0020–0·0070)
3 years	0·0032% (0·0019–0·0051)
4 years	0·0027% (0·0018–0·0043)
5 years	0·0024% (0·0017–0·0039)
6 years	0·0023% (0·0016–0·0038)
7 years	0·0023% (0·0015–0·0039)
8 years	0·0023% (0·0015–0·0042)
9 years	0·0025% (0·0016–0·0046)
10 years	0·0028% (0·0018–0·0050)
11 years	0·0031% (0·0021–0·0056)
12 years	0·0036% (0·0024–0·0063)
13 years	0·0042% (0·0028–0·0073)
14 years	0·0050% (0·0034–0·0084)
15 years	0·0060% (0·0041–0·0097)
16 years	0·0071% (0·0050–0·0113)
17 years	0·0085% (0·0060–0·0134)
18 years	0·0100% (0·0071–0·0157)
19 years	0·0118% (0·0084–0·0183)
20 years	0·0138% (0·0098–0·0214)
21 years	0·0162% (0·0114–0·0250)
22 years	0·0188% (0·0133–0·0292)
23 years	0·0219% (0·0153–0·0335)
24 years	0·0254% (0·0178–0·0385)
25 years	0·0293% (0·0207–0·0441)
26 years	0·0337% (0·0241–0·0504)
27 years	0·0386% (0·0280–0·0575)
28 years	0·0442% (0·0324–0·0658)
29 years	0·0504% (0·0372–0·0757)
30 years	0·0573% (0·0418–0·0870)
31 years	0·0650% (0·0469–0·0983)
32 years	0·0735% (0·0526–0·1108)
33 years	0·0829% (0·0590–0·1246)
34 years	0·0932% (0·0663–0·1398)
35 years	0·1046% (0·0747–0·1564)
36 years	0·1171% (0·0842–0·1746)
37 years	0·1307% (0·0950–0·1944)
38 years	0·1455% (0·1070–0·2161)
39 years	0·1616% (0·1197–0·2420)
40 years	0·1789% (0·1319–0·2706)
41 years	0·1976% (0·1440–0·3038)
42 years	0·2177% (0·1575–0·3397)
43 years	0·2391% (0·1714–0·3731)
44 years	0·2620% (0·1861–0·4122)
45 years	0·2863% (0·2016–0·4540)
46 years	0·3119% (0·2176–0·4980)
47 years	0·3389% (0·2350–0·5437)
48 years	0·3672% (0·2541–0·5906)
49 years	0·3968% (0·2748–0·6380)
50 years	0·4278% (0·2958–0·6858)
51 years	0·4606% (0·3180–0·7346)
52 years	0·4958% (0·3430–0·7855)
53 years	0·5342% (0·3720–0·8398)
54 years	0·5766% (0·4028–0·8992)
55 years	0·6242% (0·4358–0·9715)
56 years	0·6785% (0·4736–1·0602)
57 years	0·7413% (0·5181–1·1621)
58 years	0·8149% (0·5698–1·2796)
59 years	0·9022% (0·6304–1·4162)
60 years	1·0035% (0·7002–1·5727)
61 years	1·1162% (0·7776–1·7462)
62 years	1·2413% (0·8635–1·9438)
63 years	1·3803% (0·9588–2·1644)
64 years	1·5346% (1·0645–2·4094)
65 years	1·7058% (1·1817–2·6813)
66 years	1·8957% (1·3117–2·9830)
67 years	2·1064% (1·4557–3·3175)
68 years	2·3399% (1·6154–3·6881)
69 years	2·5986% (1·7928–4·0983)
70 years	2·8851% (1·9893–4·5519)
71 years	3·2022% (2·2069–5·0532)
72 years	3·5527% (2·4476–5·6064)
73 years	3·9402% (2·7139–6·2162)
74 years	4·3679% (3·0083–6·8875)
75 years	4·8397% (3·3336–7·6254)
76 years	5·3597% (3·6926–8·4353)
77 years	5·9320% (4·0887–9·3240)
78 years	6·5612% (4·5253–10·2959)
79 years	7·2520% (5·0061–11·3392)
80 years	8·0093% (5·5339–12·4411)
81 years	8·8381% (6·1140–13·6344)
82 years	9·7437% (6·7643–14·9249)
83 years	10·7311% (7·4783–16·3145)
84 years	11·8054% (8·2609–17·8063)
85 years	12·9717% (9·1175–19·4030)
86 years	14·2346% (10·0530–21·1061)
87 years	15·5984% (11·0729–22·9162)
88 years	17·0669% (12·1823–24·8326)
89 years	18·6431% (13·3860–26·8535)
90 years	20·3292% (14·6888–28·9754)
91 years	22·1263% (16·0949–31·1935)
92 years	24·0344% (17·6000–33·5013)
93 years	26·0519% (19·1384–35·8908)
94 years	28·1760% (20·7725–38·3524)
95 years	30·4021% (22·4676–40·8752)
96 years	32·7239% (24·2367–43·4272)
97 years	35·1335% (26·0981–46·0137)
98 years	37·6213% (28·0497–48·6219)
99 years	40·1762% (30·0877–51·2376)
100 years	42·7856% (32·2380–53·8466)

IFR=infection-fatality ratio.

**Figure 4:**
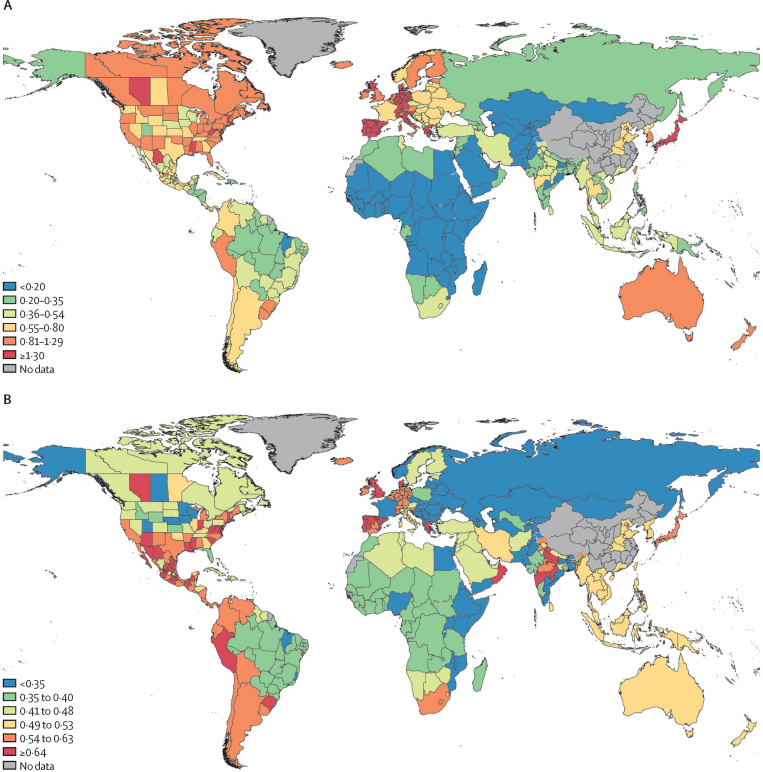
COVID-19 infection–fatality ratio by location (A) All-age COVID-19 infection–fatality ratio, July 15, 2020. (B) Age-standardised COVID-19 infection–fatality ratio, July 15, 2020.

Locations in North America and Europe generally had the highest IFRs without age standardisation on July 15, 2020 (figure 4A). After age standardisation, locations in both American continents and a smaller number of European countries had the highest IFRs on July 15, 2020 (figure 4B). Specifically, countries with the highest all-age IFR were Portugal, Monaco, Japan, Spain, and Greece (table 2). After age standardisation, the countries with the highest IFR were Peru, Portugal, Oman, Spain, and Mexico. Comparing the distribution of logit-scale IFR estimates before and after age standardisation, considering predictions for all countries and territories on July 15, 2020, the percentage of variation attributable to population age distribution was 87%. Population age structure accounted for 74% of logit-scale variation in IFRs estimated for 39 in-sample countries on July 15, 2020. Among regions with subnational estimates, we found pervasively high age-standardised IFRs among locations within Spain and Mexico. Subnational locations with high IFR also included hotspots in the UK and southern and eastern states of the USA. Sub-Saharan African countries and Asian countries generally had the lowest all-age and age-standardised IFRs.

**Table 2:** COVID-19 IFR estimates during April, 2020, July, 2020, October, 2020, and January, 2021, both all age and age standardised, for 190 countries and territories

		**IFR, April 15, 2020**	**IFR, July 15, 2020**	**IFR, Oct 15, 2020**	**IFR, Jan 1, 2021**	**Age-standardised IFR, April 15, 2020**	**Age-standardised IFR, July 15, 2020**	**Age-standardised IFR, Oct 15, 2020**	**Age-standardised IFR, Jan 1, 2021**
**Central Europe, eastern Europe, and central Asia**									
Central Asia									
	Armenia	0·494% (0·202–0·945)	0·386% (0·187–0·756)	0·335% (0·163–0·589)	0·318% (0·156–0·546)	0·374% (0·153–0·716)	0·292% (0·142–0·573)	0·254% (0·123–0·446)	0·241% (0·118–0·414)
	Azerbaijan	0·284% (0·117–0·544)	0·222% (0·107–0·432)	0·193% (0·095–0·337)	0·183% (0·090–0·312)	0·375% (0·154–0·717)	0·293% (0·141–0·570)	0·254% (0·125–0·444)	0·241% (0·119–0·412)
	Georgia	0·425% (0·177–1·072)	0·331% (0·147–0·852)	0·287% (0·141–0·704)	0·271% (0·138–0·698)	0·248% (0·103–0·625)	0·193% (0·086–0·497)	0·167% (0·082–0·411)	0·158% (0·081–0·407)
	Kazakhstan	0·222% (0·066–0·534)	0·172% (0·053–0·422)	0·150% (0·047–0·345)	0·142% (0·045–0·337)	0·272% (0·081–0·654)	0·210% (0·064–0·516)	0·183% (0·057–0·422)	0·174% (0·055–0·412)
	Kyrgyzstan	0·228% (0·094–0·433)	0·178% (0·086–0·348)	0·154% (0·076–0·272)	0·147% (0·073–0·252)	0·372% (0·154–0·708)	0·291% (0·141–0·568)	0·252% (0·124–0·444)	0·240% (0·119–0·412)
	Mongolia	0·216% (0·089–0·412)	0·169% (0·082–0·329)	0·146% (0·072–0·257)	0·139% (0·069–0·238)	0·377% (0·155–0·719)	0·295% (0·142–0·575)	0·256% (0·126–0·448)	0·243% (0·121–0·415)
	Tajikistan	0·157% (0·065–0·295)	0·122% (0·059–0·237)	0·106% (0·053–0·187)	0·101% (0·051–0·173)	0·364% (0·152–0·688)	0·285% (0·138–0·552)	0·247% (0·123–0·435)	0·235% (0·118–0·403)
	Turkmenistan	0·249% (0·102–0·479)	0·194% (0·093–0·376)	0·169% (0·083–0·293)	0·160% (0·079–0·273)	0·376% (0·153–0·722)	0·293% (0·141–0·567)	0·254% (0·126–0·442)	0·242% (0·119–0·412)
	Uzbekistan	0·230% (0·080–0·441)	0·180% (0·075–0·338)	0·158% (0·068–0·293)	0·151% (0·068–0·273)	0·476% (0·165–0·912)	0·373% (0·156–0·699)	0·327% (0·140–0·605)	0·312% (0·140–0·564)
Central Europe									
	Albania	0·391% (0·113–0·901)	0·308% (0·094–0·687)	0·270% (0·082–0·546)	0·258% (0·074–0·542)	0·254% (0·073–0·586)	0·200% (0·061–0·447)	0·176% (0·053–0·355)	0·168% (0·048–0·352)
	Bosnia and Herzegovina	0·705% (0·335–1·228)	0·552% (0·295–0·961)	0·479% (0·269–0·754)	0·456% (0·260–0·722)	0·400% (0·190–0·698)	0·314% (0·167–0·546)	0·272% (0·153–0·428)	0·259% (0·148–0·410)
	Bulgaria	0·873% (0·415–1·518)	0·684% (0·364–1·188)	0·594% (0·335–0·932)	0·565% (0·321–0·894)	0·403% (0·192–0·701)	0·316% (0·168–0·549)	0·274% (0·155–0·430)	0·261% (0·148–0·413)
	Croatia	0·864% (0·401–1·654)	0·674% (0·375–1·323)	0·584% (0·355–1·050)	0·555% (0·347–0·929)	0·402% (0·187–0·771)	0·314% (0·175–0·616)	0·272% (0·165–0·489)	0·258% (0·162–0·433)
	Czech Republic	0·584% (0·307–1·153)	0·454% (0·270–0·922)	0·394% (0·250–0·736)	0·375% (0·247–0·652)	0·287% (0·151–0·568)	0·224% (0·133–0·454)	0·194% (0·123–0·362)	0·184% (0·122–0·321)
	Hungary	0·835% (0·397–1·446)	0·654% (0·345–1·136)	0·568% (0·320–0·891)	0·540% (0·306–0·856)	0·401% (0·191–0·695)	0·314% (0·166–0·545)	0·273% (0·153–0·428)	0·260% (0·147–0·411)
	Montenegro	0·613% (0·292–1·063)	0·480% (0·254–0·833)	0·417% (0·235–0·653)	0·397% (0·225–0·628)	0·403% (0·192–0·699)	0·316% (0·167–0·548)	0·274% (0·154–0·430)	0·261% (0·148–0·413)
	North Macedonia	0·546% (0·260–0·953)	0·428% (0·228–0·745)	0·372% (0·208–0·584)	0·354% (0·201–0·559)	0·402% (0·191–0·702)	0·315% (0·168–0·549)	0·274% (0·154–0·430)	0·260% (0·148–0·412)
	Poland	0·872% (0·381–1·751)	0·680% (0·322–1·395)	0·591% (0·290–1·107)	0·563% (0·275–0·992)	0·466% (0·203–0·936)	0·363% (0·172–0·746)	0·316% (0·155–0·592)	0·301% (0·147–0·530)
	Romania	0·808% (0·387–1·393)	0·633% (0·334–1·093)	0·550% (0·312–0·863)	0·523% (0·298–0·828)	0·402% (0·192–0·692)	0·315% (0·166–0·543)	0·273% (0·155–0·429)	0·260% (0·148–0·412)
	Serbia	0·711% (0·337–1·234)	0·557% (0·297–0·970)	0·483% (0·270–0·761)	0·460% (0·259–0·729)	0·402% (0·191–0·698)	0·315% (0·168–0·549)	0·273% (0·153–0·430)	0·260% (0·147–0·413)
	Slovakia	0·668% (0·317–1·160)	0·523% (0·277–0·912)	0·454% (0·254–0·715)	0·432% (0·243–0·685)	0·397% (0·189–0·690)	0·311% (0·165–0·543)	0·270% (0·151–0·426)	0·257% (0·144–0·408)
	Slovenia	1·785% (0·459–3·957)	1·390% (0·384–3·091)	1·199% (0·339–2·396)	1·137% (0·331–2·298)	0·797% (0·205–1·766)	0·620% (0·171–1·379)	0·535% (0·151–1·069)	0·508% (0·148–1·026)
Eastern Europe									
	Belarus	0·709% (0·300–1·275)	0·556% (0·252–0·974)	0·484% (0·244–0·783)	0·461% (0·238–0·749)	0·417% (0·177–0·751)	0·328% (0·149–0·574)	0·285% (0·144–0·461)	0·272% (0·140–0·441)
	Estonia	1·313% (0·504–2·631)	1·025% (0·422–2·069)	0·886% (0·394–1·595)	0·838% (0·385–1·354)	0·609% (0·234–1·221)	0·476% (0·196–0·960)	0·411% (0·183–0·740)	0·389% (0·179–0·629)
	Latvia	0·936% (0·400–1·674)	0·734% (0·332–1·289)	0·640% (0·321–1·027)	0·609% (0·314–0·983)	0·418% (0·179–0·748)	0·328% (0·148–0·576)	0·286% (0·144–0·459)	0·272% (0·140–0·439)
	Lithuania	0·920% (0·393–1·653)	0·722% (0·327–1·264)	0·629% (0·318–1·015)	0·599% (0·311–0·971)	0·415% (0·177–0·746)	0·326% (0·148–0·570)	0·284% (0·143–0·458)	0·270% (0·140–0·438)
	Moldova	0·644% (0·276–1·154)	0·505% (0·229–0·880)	0·440% (0·224–0·707)	0·419% (0·219–0·677)	0·412% (0·177–0·738)	0·323% (0·146–0·563)	0·282% (0·143–0·452)	0·268% (0·140–0·433)
	Russia	0·276% (0·092–1·080)	0·217% (0·077–0·858)	0·194% (0·064–0·702)	0·187% (0·058–0·666)	0·173% (0·058–0·676)	0·136% (0·048–0·537)	0·121% (0·040–0·440)	0·117% (0·036–0·417)
	Ukraine	0·732% (0·314–1·314)	0·574% (0·260–1·004)	0·500% (0·253–0·805)	0·476% (0·247–0·770)	0·418% (0·179–0·750)	0·328% (0·148–0·573)	0·286% (0·144–0·459)	0·272% (0·141–0·440)
**High income**									
Australasia									
	Australia	1·190% (0·792–1·825)	0·924% (0·707–1·419)	0·809% (0·642–1·117)	0·772% (0·592–1·033)	0·682% (0·454–1·046)	0·530% (0·405–0·813)	0·464% (0·368–0·640)	0·443% (0·339–0·592)
	New Zealand	1·217% (0·810–1·866)	0·945% (0·723–1·454)	0·827% (0·657–1·143)	0·790% (0·605–1·056)	0·686% (0·456–1·052)	0·533% (0·407–0·819)	0·466% (0·370–0·644)	0·445% (0·341–0·595)
High-income countries in Asia Pacific									
	Brunei	0·407% (0·274–0·618)	0·316% (0·236–0·488)	0·277% (0·220–0·380)	0·264% (0·199–0·356)	0·694% (0·467–1·054)	0·539% (0·402–0·832)	0·472% (0·375–0·648)	0·450% (0·339–0·606)
	Japan	2·252% (1·535–3·444)	1·750% (1·302–2·690)	1·531% (1·211–2·106)	1·461% (1·086–1·984)	0·696% (0·474–1·064)	0·541% (0·402–0·831)	0·473% (0·374–0·651)	0·452% (0·336–0·613)
	Singapore	0·923% (0·627–1·403)	0·717% (0·544–1·100)	0·628% (0·502–0·862)	0·599% (0·454–0·807)	0·686% (0·466–1·042)	0·533% (0·404–0·817)	0·466% (0·373–0·640)	0·445% (0·337–0·600)
	South Korea	1·154% (0·786–1·765)	0·897% (0·669–1·382)	0·785% (0·621–1·081)	0·749% (0·557–1·014)	0·695% (0·473–1·062)	0·540% (0·403–0·832)	0·472% (0·374–0·651)	0·451% (0·335–0·610)
High-income countries in North America									
	Canada	1·059% (0·188–2·946)	0·815% (0·174–2·275)	0·707% (0·162–1·760)	0·673% (0·162–1·669)	0·544% (0·096–1·513)	0·418% (0·089–1·168)	0·363% (0·083–0·904)	0·345% (0·083–0·857)
	USA	1·280% (0·771–1·877)	0·909% (0·698–1·353)	0·791% (0·635–1·086)	0·754% (0·589–0·995)	0·733% (0·442–1·075)	0·521% (0·400–0·775)	0·453% (0·364–0·622)	0·432% (0·338–0·570)
Southern Latin America									
	Argentina	0·825% (0·548–1·259)	0·641% (0·487–0·986)	0·560% (0·448–0·773)	0·535% (0·412–0·716)	0·700% (0·464–1·068)	0·543% (0·413–0·836)	0·475% (0·380–0·655)	0·453% (0·349–0·608)
	Chile	0·924% (0·617–1·416)	0·718% (0·547–1·106)	0·628% (0·502–0·871)	0·599% (0·461–0·806)	0·713% (0·476–1·093)	0·554% (0·422–0·854)	0·485% (0·387–0·672)	0·463% (0·356–0·622)
	Uruguay	1·171% (0·779–1·792)	0·910% (0·690–1·404)	0·796% (0·634–1·101)	0·759% (0·585–1·020)	0·712% (0·474–1·089)	0·553% (0·419–0·853)	0·484% (0·385–0·669)	0·462% (0·356–0·620)
Western Europe									
	Andorra	1·115% (0·585–1·816)	0·875% (0·422–1·429)	0·772% (0·368–1·301)	0·737% (0·360–1·264)	0·625% (0·328–1·019)	0·491% (0·237–0·802)	0·433% (0·207–0·730)	0·413% (0·202–0·709)
	Austria	1·267% (0·552–2·284)	0·984% (0·482–1·682)	0·863% (0·456–1·602)	0·823% (0·438–1·591)	0·593% (0·258–1·070)	0·461% (0·226–0·787)	0·404% (0·214–0·750)	0·386% (0·205–0·745)
	Belgium	1·672% (1·105–2·754)	1·251% (0·793–2·137)	1·086% (0·698–1·776)	1·034% (0·641–1·608)	0·784% (0·518–1·291)	0·587% (0·372–1·002)	0·509% (0·327–0·833)	0·485% (0·301–0·754)
	Cyprus	1·143% (0·814–1·883)	0·895% (0·637–1·459)	0·788% (0·565–1·183)	0·754% (0·517–1·099)	0·802% (0·571–1·322)	0·629% (0·447–1·024)	0·553% (0·397–0·831)	0·529% (0·363–0·772)
	Denmark	1·837% (0·953–2·881)	1·446% (0·788–2·190)	1·279% (0·680–1·974)	1·226% (0·665–1·964)	0·898% (0·466–1·409)	0·707% (0·385–1·071)	0·626% (0·332–0·965)	0·600% (0·325–0·960)
	Finland	1·443% (0·313–3·904)	1·125% (0·256–3·013)	0·966% (0·256–2·325)	0·911% (0·256–2·128)	0·614% (0·133–1·662)	0·479% (0·109–1·283)	0·411% (0·109–0·990)	0·388% (0·109–0·906)
	France	0·858% (0·588–1·397)	0·674% (0·422–1·176)	0·595% (0·374–1·102)	0·570% (0·321–1·054)	0·384% (0·263–0·625)	0·302% (0·189–0·526)	0·266% (0·167–0·493)	0·255% (0·144–0·472)
	Germany	1·959% (1·389–3·233)	1·535% (1·087–2·504)	1·351% (0·979–2·030)	1·293% (0·895–1·879)	0·809% (0·574–1·335)	0·634% (0·449–1·034)	0·558% (0·404–0·838)	0·534% (0·370–0·776)
	Greece	2·089% (1·478–3·450)	1·637% (1·155–2·678)	1·441% (1·041–2·166)	1·378% (0·951–2·006)	0·821% (0·580–1·355)	0·643% (0·454–1·052)	0·566% (0·409–0·851)	0·541% (0·374–0·788)
	Iceland	1·307% (0·924–2·150)	1·024% (0·734–1·669)	0·902% (0·655–1·354)	0·862% (0·597–1·245)	0·788% (0·557–1·297)	0·617% (0·442–1·007)	0·544% (0·395–0·816)	0·520% (0·360–0·751)
	Ireland	1·216% (0·861–2·006)	0·952% (0·679–1·553)	0·839% (0·609–1·261)	0·802% (0·555–1·163)	0·800% (0·566–1·320)	0·627% (0·447–1·021)	0·552% (0·401–0·830)	0·528% (0·365–0·765)
	Israel	0·527% (0·328–1·003)	0·408% (0·301–0·735)	0·362% (0·253–0·680)	0·348% (0·225–0·680)	0·428% (0·266–0·815)	0·332% (0·245–0·597)	0·294% (0·206–0·553)	0·283% (0·183–0·553)
	Italy	1·721% (1·220–2·791)	1·368% (0·855–2·154)	1·211% (0·806–1·840)	1·161% (0·763–1·805)	0·655% (0·464–1·062)	0·521% (0·325–0·820)	0·461% (0·307–0·700)	0·442% (0·291–0·687)
	Luxembourg	1·343% (0·955–2·215)	1·052% (0·749–1·710)	0·926% (0·666–1·387)	0·886% (0·611–1·291)	0·800% (0·569–1·319)	0·627% (0·446–1·019)	0·552% (0·397–0·826)	0·528% (0·364–0·769)
	Malta	1·745% (1·236–2·869)	1·367% (0·972–2·234)	1·204% (0·870–1·803)	1·151% (0·796–1·666)	0·796% (0·564–1·308)	0·623% (0·443–1·019)	0·549% (0·397–0·822)	0·525% (0·363–0·760)
	Monaco	2·270% (1·595–3·744)	1·778% (1·265–2·915)	1·566% (1·146–2·368)	1·497% (1·027–2·154)	0·809% (0·568–1·334)	0·633% (0·451–1·039)	0·558% (0·408–0·844)	0·534% (0·366–0·768)
	Netherlands	1·417% (0·787–2·492)	1·150% (0·596–2·065)	1·027% (0·542–1·713)	0·987% (0·497–1·575)	0·692% (0·384–1·217)	0·561% (0·291–1·008)	0·502% (0·265–0·836)	0·482% (0·243–0·769)
	Norway	0·753% (0·020–2·280)	0·582% (0·015–1·724)	0·504% (0·013–1·401)	0·485% (0·013–1·367)	0·405% (0·011–1·227)	0·313% (0·008–0·927)	0·271% (0·007–0·754)	0·261% (0·007–0·736)
	Portugal	2·683% (1·083–5·567)	2·085% (0·946–4·395)	1·805% (0·901–3·456)	1·708% (0·901–3·269)	1·095% (0·442–2·271)	0·850% (0·386–1·793)	0·736% (0·368–1·410)	0·697% (0·368–1·334)
	San Marino	1·668% (1·181–2·752)	1·306% (0·930–2·130)	1·150% (0·835–1·730)	1·100% (0·762–1·596)	0·802% (0·568–1·323)	0·628% (0·447–1·024)	0·553% (0·401–0·832)	0·529% (0·366–0·768)
	Spain	2·175% (1·341–3·454)	1·710% (0·991–2·718)	1·512% (0·848–2·298)	1·447% (0·817–2·270)	0·955% (0·589–1·516)	0·751% (0·435–1·193)	0·664% (0·372–1·009)	0·635% (0·358–0·996)
	Sweden	1·310% (0·750–2·493)	1·035% (0·651–1·883)	0·916% (0·605–1·461)	0·878% (0·565–1·437)	0·606% (0·347–1·152)	0·478% (0·301–0·870)	0·423% (0·280–0·675)	0·406% (0·261–0·664)
	Switzerland	1·687% (1·202–2·782)	1·322% (0·943–2·148)	1·164% (0·835–1·743)	1·113% (0·766–1·621)	0·796% (0·567–1·312)	0·623% (0·445–1·013)	0·549% (0·394–0·822)	0·525% (0·361–0·765)
	UK	1·568% (1·222–2·473)	1·339% (1·054–2·155)	1·237% (0·953–1·877)	1·201% (0·880–1·727)	0·809% (0·631–1·276)	0·691% (0·544–1·112)	0·638% (0·492–0·969)	0·620% (0·454–0·891)
**Latin America and Caribbean**									
Andean Latin America									
	Bolivia	0·465% (0·309–0·845)	0·369% (0·260–0·621)	0·326% (0·229–0·527)	0·312% (0·208–0·511)	0·715% (0·475–1·300)	0·568% (0·400–0·956)	0·501% (0·353–0·810)	0·480% (0·320–0·785)
	Ecuador	0·573% (0·377–1·031)	0·455% (0·323–0·759)	0·401% (0·284–0·652)	0·384% (0·253–0·624)	0·725% (0·477–1·305)	0·576% (0·409–0·961)	0·508% (0·359–0·825)	0·486% (0·321–0·790)
	Peru	1·065% (0·669–1·849)	0·837% (0·585–1·412)	0·740% (0·482–1·249)	0·708% (0·439–1·175)	1·159% (0·728–2·013)	0·911% (0·636–1·538)	0·806% (0·525–1·360)	0·771% (0·478–1·279)
Caribbean									
	Antigua and Barbuda	0·584% (0·318–1·062)	0·461% (0·279–0·856)	0·403% (0·250–0·688)	0·384% (0·238–0·628)	0·550% (0·299–1·000)	0·434% (0·262–0·806)	0·379% (0·235–0·648)	0·362% (0·224–0·591)
	The Bahamas	0·522% (0·289–0·959)	0·412% (0·248–0·773)	0·360% (0·225–0·622)	0·343% (0·213–0·558)	0·554% (0·306–1·018)	0·437% (0·263–0·820)	0·382% (0·239–0·659)	0·364% (0·226–0·592)
	Barbados	0·910% (0·504–1·684)	0·718% (0·431–1·358)	0·628% (0·390–1·091)	0·599% (0·367–0·971)	0·553% (0·306–1·023)	0·436% (0·262–0·825)	0·381% (0·237–0·663)	0·364% (0·223–0·590)
	Belize	0·340% (0·188–0·623)	0·268% (0·162–0·502)	0·234% (0·147–0·404)	0·223% (0·139–0·363)	0·557% (0·309–1·021)	0·439% (0·265–0·823)	0·384% (0·241–0·661)	0·366% (0·228–0·594)
	Bermuda	1·181% (0·652–2·217)	0·931% (0·545–1·787)	0·814% (0·498–1·436)	0·776% (0·464–1·262)	0·554% (0·306–1·040)	0·437% (0·256–0·839)	0·382% (0·234–0·674)	0·364% (0·218–0·592)
	Cuba	0·980% (0·541–1·825)	0·773% (0·459–1·471)	0·676% (0·417–1·182)	0·645% (0·390–1·047)	0·567% (0·313–1·056)	0·447% (0·266–0·851)	0·391% (0·241–0·684)	0·373% (0·226–0·606)
	Dominica	0·721% (0·401–1·323)	0·568% (0·345–1·067)	0·497% (0·314–0·857)	0·474% (0·296–0·769)	0·558% (0·310–1·024)	0·440% (0·267–0·825)	0·385% (0·243–0·663)	0·367% (0·229–0·595)
	Dominican Republic	0·437% (0·240–0·795)	0·345% (0·209–0·641)	0·301% (0·189–0·515)	0·287% (0·179–0·468)	0·552% (0·304–1·005)	0·435% (0·264–0·810)	0·381% (0·238–0·651)	0·363% (0·226–0·592)
	Grenada	0·539% (0·294–0·975)	0·425% (0·259–0·786)	0·372% (0·233–0·631)	0·355% (0·221–0·580)	0·553% (0·301–1·000)	0·436% (0·265–0·806)	0·382% (0·239–0·647)	0·364% (0·226–0·595)
	Guyana	0·389% (0·215–0·706)	0·307% (0·188–0·569)	0·269% (0·170–0·457)	0·256% (0·161–0·417)	0·557% (0·307–1·009)	0·439% (0·269–0·813)	0·384% (0·243–0·654)	0·366% (0·230–0·596)
	Haiti	0·262% (0·143–0·459)	0·207% (0·130–0·367)	0·181% (0·117–0·295)	0·172% (0·106–0·284)	0·547% (0·299–0·959)	0·431% (0·272–0·766)	0·377% (0·245–0·616)	0·360% (0·222–0·592)
	Jamaica	0·603% (0·332–1·105)	0·476% (0·287–0·891)	0·416% (0·259–0·716)	0·397% (0·246–0·646)	0·560% (0·308–1·025)	0·441% (0·266–0·826)	0·386% (0·241–0·664)	0·368% (0·228–0·599)
	Puerto Rico	1·189% (0·613–2·443)	0·929% (0·460–1·963)	0·812% (0·436–1·552)	0·773% (0·422–1·365)	0·545% (0·281–1·119)	0·426% (0·211–0·899)	0·372% (0·200–0·711)	0·354% (0·193–0·626)
	Saint Kitts and Nevis	0·532% (0·292–0·977)	0·419% (0·252–0·787)	0·367% (0·227–0·633)	0·350% (0·215–0·569)	0·548% (0·301–1·007)	0·432% (0·260–0·812)	0·378% (0·235–0·652)	0·360% (0·222–0·587)
	Saint Lucia	0·657% (0·360–1·198)	0·518% (0·313–0·966)	0·453% (0·283–0·776)	0·432% (0·268–0·703)	0·555% (0·304–1·012)	0·437% (0·264–0·816)	0·383% (0·239–0·656)	0·365% (0·227–0·594)
	Saint Vincent and the Grenadines	0·627% (0·345–1·141)	0·494% (0·300–0·919)	0·432% (0·272–0·739)	0·412% (0·257–0·671)	0·561% (0·309–1·021)	0·442% (0·269–0·823)	0·387% (0·243–0·662)	0·369% (0·230–0·601)
	Suriname	0·545% (0·302–0·996)	0·430% (0·261–0·802)	0·376% (0·238–0·645)	0·359% (0·225–0·583)	0·564% (0·313–1·031)	0·445% (0·271–0·831)	0·389% (0·246–0·668)	0·371% (0·233–0·604)
	Trinidad and Tobago	0·721% (0·403–1·336)	0·568% (0·342–1·076)	0·497% (0·314–0·865)	0·474% (0·294–0·768)	0·563% (0·315–1·044)	0·444% (0·267–0·841)	0·389% (0·245–0·676)	0·371% (0·229–0·600)
	Virgin Islands	0·954% (0·539–1·828)	0·752% (0·445–1·473)	0·658% (0·417–1·158)	0·627% (0·380–1·022)	0·560% (0·316–1·073)	0·442% (0·261–0·865)	0·386% (0·245–0·680)	0·368% (0·223–0·600)
Central Latin America									
	Colombia	0·777% (0·452–1·483)	0·616% (0·415–1·149)	0·542% (0·353–0·928)	0·517% (0·328–0·873)	0·707% (0·411–1·349)	0·560% (0·378–1·045)	0·493% (0·321–0·844)	0·471% (0·298–0·794)
	Costa Rica	0·757% (0·440–1·449)	0·600% (0·402–1·121)	0·528% (0·345–0·899)	0·504% (0·321–0·855)	0·713% (0·414–1·365)	0·565% (0·379–1·056)	0·497% (0·325–0·847)	0·475% (0·303–0·805)
	El Salvador	0·666% (0·389–1·270)	0·528% (0·356–0·983)	0·464% (0·303–0·794)	0·443% (0·282–0·749)	0·703% (0·410–1·339)	0·556% (0·375–1·037)	0·489% (0·320–0·837)	0·468% (0·297–0·789)
	Guatemala	0·400% (0·233–0·760)	0·317% (0·215–0·590)	0·279% (0·183–0·480)	0·266% (0·167–0·443)	0·702% (0·409–1·333)	0·556% (0·378–1·034)	0·489% (0·322–0·841)	0·467% (0·293–0·778)
	Honduras	0·369% (0·145–0·787)	0·291% (0·110–0·627)	0·255% (0·100–0·495)	0·244% (0·100–0·433)	0·679% (0·266–1·447)	0·536% (0·203–1·152)	0·469% (0·184–0·909)	0·448% (0·184–0·796)
	Mexico	0·796% (0·413–1·528)	0·626% (0·372–1·226)	0·549% (0·349–0·981)	0·523% (0·340–0·888)	0·912% (0·473–1·751)	0·717% (0·426–1·404)	0·629% (0·400–1·123)	0·600% (0·389–1·017)
	Nicaragua	0·424% (0·247–0·808)	0·336% (0·227–0·627)	0·296% (0·193–0·508)	0·282% (0·178–0·474)	0·704% (0·409–1·341)	0·558% (0·377–1·039)	0·490% (0·321–0·842)	0·469% (0·295–0·786)
	Panama	0·681% (0·396–1·296)	0·539% (0·365–1·004)	0·474% (0·310–0·814)	0·453% (0·285–0·760)	0·699% (0·406–1·331)	0·554% (0·375–1·031)	0·487% (0·318–0·836)	0·465% (0·293–0·780)
	Venezuela	0·692% (0·403–1·320)	0·548% (0·370–1·022)	0·482% (0·315–0·826)	0·461% (0·292–0·776)	0·709% (0·413–1·353)	0·562% (0·379–1·048)	0·494% (0·322–0·847)	0·472% (0·299–0·796)
Tropical Latin America									
	Brazil	0·442% (0·344–0·603)	0·394% (0·318–0·523)	0·372% (0·297–0·484)	0·363% (0·272–0·463)	0·425% (0·331–0·580)	0·380% (0·306–0·503)	0·358% (0·286–0·466)	0·350% (0·262–0·446)
	Paraguay	0·378% (0·271–0·575)	0·299% (0·236–0·442)	0·264% (0·199–0·354)	0·253% (0·179–0·329)	0·505% (0·362–0·769)	0·400% (0·315–0·592)	0·353% (0·266–0·473)	0·339% (0·240–0·440)
**North Africa and Middle East**									
Afghanistan		0·170% (0·057–0·374)	0·133% (0·049–0·298)	0·116% (0·042–0·255)	0·111% (0·041–0·244)	0·603% (0·204–1·328)	0·473% (0·173–1·059)	0·413% (0·151–0·905)	0·393% (0·144–0·866)
Algeria		0·372% (0·151–0·739)	0·294% (0·132–0·595)	0·257% (0·123–0·475)	0·245% (0·121–0·406)	0·524% (0·212–1·040)	0·413% (0·186–0·838)	0·362% (0·173–0·668)	0·344% (0·171–0·572)
Bahrain		0·336% (0·136–0·660)	0·265% (0·116–0·532)	0·232% (0·112–0·423)	0·221% (0·109–0·370)	0·525% (0·213–1·032)	0·414% (0·182–0·832)	0·363% (0·175–0·662)	0·345% (0·171–0·579)
Egypt		0·203% (0·073–0·461)	0·159% (0·058–0·390)	0·140% (0·051–0·303)	0·134% (0·050–0·283)	0·380% (0·137–0·861)	0·298% (0·109–0·728)	0·262% (0·096–0·566)	0·251% (0·093–0·528)
Iran		0·506% (0·203–0·965)	0·397% (0·173–0·728)	0·347% (0·164–0·615)	0·331% (0·155–0·594)	0·632% (0·254–1·207)	0·496% (0·216–0·911)	0·434% (0·205–0·769)	0·414% (0·194–0·744)
Iraq		0·248% (0·101–0·491)	0·195% (0·088–0·395)	0·171% (0·082–0·315)	0·163% (0·081–0·270)	0·524% (0·213–1·038)	0·413% (0·187–0·837)	0·362% (0·174–0·668)	0·344% (0·172–0·571)
Jordan		0·285% (0·099–0·624)	0·222% (0·088–0·499)	0·191% (0·086–0·395)	0·181% (0·086–0·348)	0·582% (0·202–1·276)	0·454% (0·180–1·021)	0·391% (0·175–0·807)	0·370% (0·175–0·712)
Kuwait		0·311% (0·124–0·607)	0·245% (0·106–0·489)	0·215% (0·102–0·389)	0·204% (0·102–0·346)	0·534% (0·214–1·042)	0·421% (0·182–0·840)	0·369% (0·175–0·668)	0·351% (0·174–0·595)
Lebanon		0·519% (0·210–1·026)	0·409% (0·179–0·827)	0·358% (0·171–0·658)	0·341% (0·167–0·572)	0·537% (0·217–1·062)	0·424% (0·186–0·856)	0·371% (0·177–0·681)	0·353% (0·173–0·592)
Libya		0·370% (0·151–0·727)	0·292% (0·131–0·586)	0·255% (0·124–0·467)	0·243% (0·121–0·406)	0·526% (0·214–1·034)	0·415% (0·186–0·833)	0·363% (0·176–0·664)	0·345% (0·172–0·577)
Morocco		0·392% (0·159–0·776)	0·310% (0·140–0·626)	0·271% (0·131–0·499)	0·258% (0·129–0·427)	0·517% (0·210–1·023)	0·408% (0·185–0·825)	0·357% (0·172–0·658)	0·340% (0·170–0·563)
Oman		0·363% (0·162–0·675)	0·284% (0·142–0·522)	0·249% (0·128–0·428)	0·237% (0·124–0·375)	0·973% (0·433–1·809)	0·762% (0·381–1·399)	0·666% (0·342–1·146)	0·636% (0·332–1·006)
Palestine		0·216% (0·087–0·430)	0·170% (0·077–0·347)	0·149% (0·071–0·277)	0·142% (0·071–0·235)	0·524% (0·212–1·043)	0·413% (0·187–0·841)	0·362% (0·172–0·671)	0·344% (0·172–0·569)
Qatar		0·077% (0·023–0·320)	0·061% (0·018–0·274)	0·054% (0·015–0·238)	0·052% (0·015–0·204)	0·208% (0·063–0·860)	0·165% (0·050–0·737)	0·146% (0·041–0·640)	0·139% (0·041–0·549)
Saudi Arabia		0·241% (0·097–0·471)	0·190% (0·083–0·380)	0·166% (0·080–0·302)	0·158% (0·079–0·266)	0·523% (0·211–1·024)	0·413% (0·181–0·826)	0·361% (0·174–0·657)	0·344% (0·171–0·579)
Sudan		0·209% (0·085–0·413)	0·165% (0·075–0·333)	0·144% (0·070–0·266)	0·137% (0·070–0·227)	0·518% (0·211–1·022)	0·408% (0·186–0·824)	0·357% (0·174–0·658)	0·340% (0·172–0·562)
Syria		0·379% (0·154–0·752)	0·299% (0·135–0·606)	0·262% (0·125–0·483)	0·249% (0·124–0·413)	0·525% (0·213–1·042)	0·414% (0·187–0·839)	0·363% (0·173–0·669)	0·345% (0·172–0·572)
Tunisia		0·528% (0·214–1·047)	0·417% (0·185–0·843)	0·365% (0·174–0·672)	0·347% (0·170–0·579)	0·526% (0·213–1·041)	0·415% (0·184–0·839)	0·363% (0·173–0·668)	0·345% (0·170–0·576)
Turkey		0·548% (0·222–1·087)	0·432% (0·192–0·877)	0·379% (0·181–0·697)	0·360% (0·177–0·601)	0·533% (0·216–1·056)	0·420% (0·187–0·853)	0·368% (0·176–0·678)	0·350% (0·172–0·585)
United Arab Emirates		0·246% (0·100–0·478)	0·194% (0·086–0·386)	0·170% (0·082–0·307)	0·161% (0·082–0·272)	0·526% (0·213–1·024)	0·415% (0·184–0·826)	0·363% (0·176–0·658)	0·346% (0·175–0·582)
Yemen		0·143% (0·045–0·312)	0·113% (0·038–0·260)	0·100% (0·034–0·212)	0·095% (0·030–0·192)	0·393% (0·125–0·856)	0·310% (0·104–0·715)	0·274% (0·094–0·582)	0·262% (0·083–0·528)
**South Asia**									
Bangladesh		0·320% (0·132–0·646)	0·251% (0·114–0·515)	0·218% (0·099–0·409)	0·207% (0·097–0·357)	0·436% (0·180–0·880)	0·342% (0·156–0·702)	0·297% (0·134–0·557)	0·282% (0·132–0·487)
Bhutan		0·285% (0·117–0·574)	0·223% (0·101–0·460)	0·194% (0·088–0·365)	0·184% (0·086–0·319)	0·426% (0·175–0·859)	0·334% (0·151–0·688)	0·290% (0·132–0·546)	0·276% (0·129–0·476)
India		0·524% (0·206–1·027)	0·407% (0·175–0·779)	0·354% (0·151–0·614)	0·337% (0·148–0·571)	0·733% (0·288–1·437)	0·569% (0·246–1·091)	0·495% (0·212–0·859)	0·471% (0·207–0·799)
Nepal		0·415% (0·147–1·043)	0·321% (0·112–0·793)	0·277% (0·097–0·616)	0·261% (0·097–0·560)	0·660% (0·233–1·657)	0·511% (0·178–1·259)	0·440% (0·154–0·978)	0·415% (0·154–0·890)
Pakistan		0·155% (0·054–0·332)	0·121% (0·040–0·257)	0·106% (0·035–0·201)	0·101% (0·035–0·188)	0·375% (0·130–0·805)	0·294% (0·098–0·624)	0·256% (0·086–0·488)	0·244% (0·086–0·455)
**Southeast Asia, east Asia, and Oceania**									
East Asia									
	China	0·853% (0·550–1·333)	0·673% (0·497–1·056)	0·590% (0·459–0·827)	0·564% (0·413–0·772)	0·646% (0·417–1·010)	0·510% (0·376–0·800)	0·447% (0·348–0·626)	0·427% (0·313–0·585)
	North Korea	0·724% (0·465–1·137)	0·572% (0·416–0·894)	0·502% (0·386–0·699)	0·479% (0·344–0·658)	0·639% (0·410–1·003)	0·505% (0·367–0·789)	0·443% (0·341–0·617)	0·423% (0·304–0·581)
	Taiwan, province of China	1·111% (0·724–1·732)	0·877% (0·658–1·383)	0·769% (0·591–1·079)	0·735% (0·557–0·992)	0·649% (0·423–1·012)	0·513% (0·384–0·808)	0·450% (0·346–0·630)	0·429% (0·326–0·580)
Oceania									
	Fiji	0·447% (0·291–0·695)	0·353% (0·266–0·557)	0·309% (0·239–0·437)	0·296% (0·225–0·401)	0·664% (0·433–1·034)	0·524% (0·395–0·828)	0·460% (0·355–0·650)	0·439% (0·334–0·597)
	Guam	0·709% (0·453–1·123)	0·560% (0·412–0·879)	0·491% (0·372–0·686)	0·469% (0·355–0·637)	0·672% (0·429–1·064)	0·530% (0·390–0·832)	0·465% (0·352–0·649)	0·444% (0·336–0·603)
	Northern Mariana Islands	0·684% (0·440–1·081)	0·539% (0·401–0·846)	0·473% (0·362–0·667)	0·452% (0·342–0·615)	0·671% (0·432–1·061)	0·529% (0·394–0·830)	0·465% (0·355–0·655)	0·444% (0·335–0·603)
	Papua New Guinea	0·257% (0·165–0·401)	0·203% (0·150–0·317)	0·178% (0·138–0·249)	0·170% (0·125–0·233)	0·657% (0·423–1·025)	0·519% (0·382–0·811)	0·455% (0·353–0·637)	0·435% (0·318–0·596)
	Vanuatu	0·315% (0·205–0·488)	0·249% (0·187–0·392)	0·218% (0·168–0·307)	0·209% (0·157–0·283)	0·648% (0·422–1·001)	0·511% (0·384–0·805)	0·449% (0·346–0·630)	0·428% (0·323–0·582)
Southeast Asia									
	Cambodia	0·393% (0·253–0·615)	0·310% (0·228–0·485)	0·272% (0·211–0·380)	0·260% (0·188–0·356)	0·642% (0·413–1·005)	0·507% (0·372–0·793)	0·445% (0·345–0·621)	0·425% (0·308–0·582)
	Indonesia	0·467% (0·302–0·729)	0·369% (0·274–0·581)	0·324% (0·251–0·454)	0·309% (0·230–0·422)	0·657% (0·425–1·025)	0·519% (0·386–0·817)	0·455% (0·352–0·639)	0·435% (0·323–0·593)
	Laos	0·336% (0·216–0·525)	0·265% (0·195–0·415)	0·233% (0·181–0·325)	0·222% (0·162–0·304)	0·646% (0·416–1·009)	0·510% (0·376–0·798)	0·447% (0·348–0·626)	0·427% (0·312–0·584)
	Malaysia	0·488% (0·318–0·759)	0·385% (0·290–0·609)	0·338% (0·259–0·475)	0·323% (0·245–0·436)	0·650% (0·423–1·010)	0·513% (0·385–0·810)	0·450% (0·345–0·632)	0·430% (0·326–0·580)
	Maldives	0·374% (0·242–0·583)	0·295% (0·219–0·464)	0·259% (0·201–0·364)	0·247% (0·183–0·337)	0·645% (0·417–1·005)	0·509% (0·377–0·800)	0·447% (0·346–0·627)	0·427% (0·315–0·582)
	Mauritius	0·842% (0·550–1·303)	0·665% (0·502–1·049)	0·583% (0·448–0·821)	0·557% (0·424–0·754)	0·654% (0·427–1·012)	0·516% (0·390–0·815)	0·453% (0·348–0·637)	0·433% (0·329–0·585)
	Myanmar	0·477% (0·307–0·744)	0·376% (0·278–0·590)	0·330% (0·257–0·462)	0·315% (0·230–0·431)	0·645% (0·415–1·007)	0·509% (0·376–0·797)	0·446% (0·347–0·625)	0·426% (0·312–0·583)
	Philippines	0·384% (0·248–0·599)	0·303% (0·224–0·476)	0·266% (0·206–0·373)	0·254% (0·187–0·347)	0·646% (0·417–1·008)	0·510% (0·377–0·801)	0·447% (0·347–0·627)	0·427% (0·315–0·583)
	Seychelles	0·640% (0·417–0·990)	0·505% (0·381–0·797)	0·443% (0·341–0·623)	0·423% (0·322–0·573)	0·651% (0·424–1·007)	0·514% (0·388–0·811)	0·451% (0·347–0·634)	0·431% (0·327–0·583)
	Sri Lanka	0·674% (0·436–1·046)	0·532% (0·395–0·834)	0·467% (0·362–0·655)	0·446% (0·330–0·606)	0·638% (0·412–0·989)	0·503% (0·374–0·789)	0·442% (0·342–0·620)	0·422% (0·312–0·574)
	Thailand	0·927% (0·602–1·436)	0·732% (0·551–1·155)	0·642% (0·495–0·900)	0·613% (0·463–0·830)	0·647% (0·420–1·002)	0·511% (0·385–0·806)	0·448% (0·345–0·628)	0·428% (0·323–0·579)
	Timor Leste	0·333% (0·214–0·522)	0·263% (0·192–0·411)	0·231% (0·178–0·322)	0·220% (0·159–0·302)	0·650% (0·417–1·020)	0·513% (0·375–0·803)	0·450% (0·347–0·628)	0·430% (0·311–0·590)
	Vietnam	0·560% (0·360–0·876)	0·442% (0·325–0·692)	0·388% (0·301–0·542)	0·370% (0·269–0·507)	0·644% (0·415–1·008)	0·509% (0·374–0·797)	0·446% (0·347–0·624)	0·426% (0·310–0·584)
**Sub-Saharan Africa**									
Central sub-Saharan Africa									
	Angola	0·151% (0·069–0·272)	0·118% (0·063–0·205)	0·103% (0·056–0·179)	0·099% (0·052–0·161)	0·494% (0·226–0·893)	0·388% (0·208–0·673)	0·339% (0·183–0·586)	0·323% (0·172–0·530)
	Central African Republic	0·159% (0·073–0·286)	0·124% (0·066–0·215)	0·109% (0·059–0·188)	0·104% (0·056–0·170)	0·491% (0·225–0·885)	0·386% (0·206–0·666)	0·337% (0·183–0·581)	0·322% (0·173–0·527)
	Congo (Brazzaville)	0·208% (0·095–0·376)	0·163% (0·087–0·283)	0·142% (0·078–0·247)	0·136% (0·073–0·222)	0·497% (0·226–0·898)	0·390% (0·209–0·678)	0·341% (0·186–0·590)	0·325% (0·174–0·530)
	DR Congo	0·167% (0·076–0·301)	0·131% (0·070–0·227)	0·114% (0·062–0·197)	0·109% (0·058–0·178)	0·489% (0·223–0·884)	0·384% (0·205–0·666)	0·336% (0·181–0·580)	0·320% (0·171–0·524)
	Equatorial Guinea	0·150% (0·069–0·272)	0·118% (0·062–0·206)	0·103% (0·057–0·179)	0·098% (0·052–0·158)	0·501% (0·230–0·909)	0·393% (0·208–0·687)	0·343% (0·190–0·596)	0·328% (0·173–0·529)
	Gabon	0·258% (0·118–0·467)	0·202% (0·107–0·353)	0·177% (0·097–0·307)	0·169% (0·090–0·273)	0·501% (0·230–0·908)	0·393% (0·209–0·686)	0·343% (0·189–0·596)	0·328% (0·174–0·531)
Eastern sub-Saharan Africa									
	Burundi	0·141% (0·060–0·282)	0·110% (0·050–0·212)	0·097% (0·047–0·181)	0·092% (0·043–0·161)	0·442% (0·188–0·885)	0·347% (0·158–0·666)	0·304% (0·146–0·568)	0·290% (0·136–0·506)
	Comoros	0·270% (0·114–0·541)	0·212% (0·096–0·408)	0·185% (0·091–0·347)	0·177% (0·083–0·308)	0·446% (0·188–0·894)	0·350% (0·158–0·673)	0·306% (0·150–0·574)	0·292% (0·137–0·509)
	Djibouti	0·189% (0·080–0·379)	0·148% (0·067–0·286)	0·130% (0·063–0·244)	0·124% (0·058–0·216)	0·452% (0·191–0·907)	0·355% (0·160–0·684)	0·311% (0·151–0·583)	0·296% (0·139–0·517)
	Eritrea	0·144% (0·061–0·288)	0·113% (0·052–0·217)	0·099% (0·048–0·185)	0·094% (0·044–0·165)	0·441% (0·188–0·882)	0·346% (0·158–0·664)	0·303% (0·147–0·566)	0·289% (0·136–0·504)
	Ethiopia	0·148% (0·063–0·297)	0·117% (0·053–0·224)	0·102% (0·049–0·191)	0·097% (0·045–0·170)	0·439% (0·186–0·879)	0·344% (0·157–0·661)	0·301% (0·145–0·564)	0·288% (0·134–0·502)
	Kenya	0·153% (0·071–0·336)	0·121% (0·054–0·264)	0·107% (0·050–0·221)	0·102% (0·045–0·206)	0·407% (0·189–0·897)	0·322% (0·143–0·705)	0·284% (0·134–0·590)	0·271% (0·120–0·549)
	Madagascar	0·151% (0·064–0·302)	0·118% (0·054–0·227)	0·104% (0·050–0·194)	0·099% (0·046–0·173)	0·446% (0·190–0·893)	0·350% (0·159–0·673)	0·307% (0·149–0·574)	0·293% (0·137–0·511)
	Malawi	0·151% (0·064–0·303)	0·119% (0·054–0·228)	0·104% (0·051–0·195)	0·099% (0·047–0·173)	0·445% (0·189–0·892)	0·349% (0·158–0·672)	0·306% (0·149–0·573)	0·292% (0·137–0·508)
	Mozambique	0·138% (0·044–0·303)	0·108% (0·036–0·237)	0·095% (0·035–0·205)	0·091% (0·033–0·193)	0·441% (0·141–0·969)	0·344% (0·115–0·758)	0·304% (0·112–0·656)	0·292% (0·107–0·618)
	Rwanda	0·178% (0·076–0·358)	0·140% (0·063–0·270)	0·123% (0·059–0·230)	0·117% (0·055–0·204)	0·446% (0·189–0·895)	0·350% (0·159–0·674)	0·306% (0·148–0·575)	0·292% (0·137–0·511)
	Somalia	0·118% (0·051–0·237)	0·093% (0·043–0·178)	0·081% (0·039–0·152)	0·078% (0·037–0·135)	0·444% (0·190–0·886)	0·349% (0·160–0·667)	0·305% (0·148–0·569)	0·291% (0·137–0·507)
	South Sudan	0·158% (0·067–0·316)	0·124% (0·056–0·238)	0·108% (0·053–0·203)	0·103% (0·049–0·179)	0·447% (0·190–0·894)	0·351% (0·160–0·674)	0·307% (0·151–0·574)	0·293% (0·139–0·508)
	Tanzania	0·169% (0·071–0·339)	0·133% (0·060–0·256)	0·116% (0·057–0·218)	0·111% (0·052–0·193)	0·449% (0·189–0·901)	0·352% (0·159–0·679)	0·308% (0·151–0·579)	0·294% (0·139–0·512)
	Uganda	0·131% (0·056–0·263)	0·103% (0·047–0·198	0·090% (0·044–0·169)	0·086% (0·040–0·150)	0·444% (0·188–0·890)	0·349% (0·158–0·671	0·305% (0·148–0·572)	0·291% (0·136–0·508)
	Zambia	0·142% (0·060–0·286)	0·112% (0·050–0·216)	0·098% (0·048–0·184)	0·093% (0·044–0·162)	0·451% (0·190–0·906)	0·354% (0·160–0·684)	0·310% (0·152–0·583)	0·296% (0·139–0·515)
Southern sub-Saharan Africa									
	Botswana	0·269% (0·130–0·504)	0·212% (0·114–0·370)	0·185% (0·098–0·317)	0·177% (0·093–0·299)	0·540% (0·261–1·011)	0·424% (0·228–0·741)	0·371% (0·196–0·635)	0·354% (0·186–0·599)
	Eswatini	0·226% (0·110–0·424)	0·178% (0·096–0·313)	0·156% (0·083–0·267)	0·149% (0·079–0·252)	0·534% (0·258–1·001)	0·420% (0·227–0·739)	0·368% (0·197–0·629)	0·351% (0·187–0·596)
	Lesotho	0·272% (0·132–0·509)	0·214% (0·115–0·374)	0·187% (0·100–0·320)	0·179% (0·095–0·302)	0·540% (0·262–1·009)	0·424% (0·228–0·741)	0·371% (0·198–0·634)	0·354% (0·188–0·598)
	Namibia	0·277% (0·135–0·519)	0·218% (0·117–0·379)	0·191% (0·101–0·326)	0·182% (0·095–0·307)	0·535% (0·260–1·001)	0·421% (0·226–0·731)	0·368% (0·195–0·628)	0·351% (0·184–0·592)
	South Africa	0·511% (0·199–1·275)	0·400% (0·173–0·987)	0·348% (0·145–0·774)	0·331% (0·137–0·724)	0·713% (0·278–1·780)	0·558% (0·241–1·377)	0·486% (0·202–1·081)	0·462% (0·191–1·010)
	Zimbabwe	0·210% (0·102–0·391)	0·165% (0·089–0·285)	0·144% (0·077–0·245)	0·138% (0·072–0·231)	0·537% (0·262–1·001)	0·422% (0·227–0·730)	0·369% (0·196–0·629)	0·352% (0·186–0·592)
Western sub-Saharan Africa									
	Benin	0·151% (0·065–0·287)	0·118% (0·055–0·215)	0·103% (0·051–0·183)	0·098% (0·050–0·171)	0·451% (0·195–0·858)	0·353% (0·163–0·643)	0·308% (0·153–0·546)	0·294% (0·151–0·510)
	Burkina Faso	0·155% (0·067–0·294)	0·121% (0·056–0·220)	0·106% (0·052–0·188)	0·101% (0·052–0·176)	0·451% (0·196–0·858)	0·354% (0·164–0·643)	0·309% (0·153–0·548)	0·294% (0·152–0·512)
	Cape Verde	0·325% (0·141–0·620)	0·255% (0·116–0·465)	0·222% (0·109–0·395)	0·212% (0·107–0·370)	0·450% (0·195–0·858)	0·353% (0·161–0·643)	0·308% (0·151–0·547)	0·293% (0·148–0·512)
	Cameroon	0·159% (0·068–0·302)	0·124% (0·057–0·227)	0·109% (0·054–0·193)	0·103% (0·053–0·179)	0·453% (0·195–0·864)	0·355% (0·163–0·648)	0·310% (0·155–0·550)	0·296% (0·151–0·511)
	Chad	0·132% (0·058–0·251)	0·104% (0·048–0·188)	0·090% (0·045–0·161)	0·086% (0·045–0·150)	0·453% (0·197–0·859)	0·355% (0·165–0·643)	0·310% (0·154–0·550)	0·295% (0·153–0·515)
	Côte d'Ivoire	0·157% (0·068–0·299)	0·123% (0·057–0·225)	0·108% (0·054–0·191)	0·103% (0·053–0·178)	0·454% (0·197–0·863)	0·356% (0·164–0·647)	0·310% (0·154–0·550)	0·296% (0·152–0·514)
	The Gambia	0·171% (0·074–0·326)	0·134% (0·062–0·244)	0·117% (0·058–0·208)	0·112% (0·057–0·194)	0·453% (0·196–0·860)	0·355% (0·164–0·645)	0·309% (0·153–0·549)	0·295% (0·151–0·513)
	Ghana	0·197% (0·085–0·375)	0·154% (0·071–0·281)	0·135% (0·067–0·239)	0·128% (0·065–0·223)	0·450% (0·195–0·857)	0·353% (0·162–0·642)	0·308% (0·152–0·546)	0·293% (0·150–0·510)
	Guinea	0·173% (0·075–0·329)	0·136% (0·063–0·247)	0·119% (0·059–0·210)	0·113% (0·059–0·197)	0·454% (0·197–0·862)	0·356% (0·165–0·646)	0·311% (0·155–0·551)	0·296% (0·153–0·515)
	Guinea-Bissau	0·143% (0·062–0·272)	0·112% (0·052–0·204)	0·098% (0·049–0·174)	0·093% (0·048–0·162)	0·449% (0·195–0·852)	0·352% (0·164–0·638)	0·307% (0·152–0·545)	0·293% (0·152–0·510)
	Liberia	0·171% (0·074–0·324)	0·134% (0·062–0·243)	0·117% (0·058–0·207)	0·111% (0·057–0·193)	0·451% (0·195–0·858)	0·353% (0·163–0·644)	0·309% (0·154–0·547)	0·294% (0·151–0·510)
	Mali	0·151% (0·066–0·286)	0·118% (0·055–0·215)	0·103% (0·051–0·183)	0·098% (0·051–0·171)	0·452% (0·197–0·859)	0·354% (0·164–0·644)	0·309% (0·153–0·549)	0·295% (0·152–0·514)
	Mauritania	0·213% (0·092–0·406)	0·167% (0·076–0·305)	0·146% (0·072–0·259)	0·139% (0·070–0·240)	0·467% (0·201–0·891)	0·366% (0·167–0·668)	0·319% (0·159–0·568)	0·304% (0·154–0·527)
	Niger	0·124% (0·054–0·236)	0·097% (0·045–0·176)	0·085% (0·042–0·151)	0·081% (0·042–0·141)	0·450% (0·196–0·856)	0·353% (0·164–0·640)	0·308% (0·152–0·547)	0·293% (0·152–0·512)
	Nigeria	0·114% (0·038–0·255)	0·088% (0·028–0·204)	0·078% (0·026–0·173)	0·074% (0·023–0·170)	0·324% (0·108–0·726)	0·252% (0·079–0·582)	0·221% (0·074–0·494)	0·211% (0·067–0·485)
	São Tomé and Príncipe	0·201% (0·087–0·383)	0·158% (0·072–0·287)	0·138% (0·068–0·244)	0·131% (0·067–0·229)	0·448% (0·194–0·853)	0·351% (0·161–0·640)	0·307% (0·151–0·544)	0·292% (0·149–0·509)
	Senegal	0·194% (0·084–0·369)	0·152% (0·070–0·277)	0·133% (0·066–0·236)	0·127% (0·065–0·220)	0·453% (0·196–0·861)	0·355% (0·164–0·645)	0·310% (0·153–0·549)	0·295% (0·152–0·514)
	Sierra Leone	0·172% (0·075–0·327)	0·135% (0·063–0·245)	0·118% (0·058–0·209)	0·112% (0·058–0·196)	0·456% (0·198–0·867)	0·357% (0·166–0·650)	0·312% (0·154–0·554)	0·297% (0·153–0·518)
	Togo	0·174% (0·076–0·331)	0·136% (0·063–0·248)	0·119% (0·059–0·211)	0·113% (0·058–0·197)	0·450% (0·196–0·856)	0·353% (0·163–0·641)	0·308% (0·152–0·546)	0·293% (0·151–0·511)
	Uganda	0·131% (0·056–0·263)	0·103% (0·047–0·198)	0·090% (0·044–0·169)	0·086% (0·040–0·150)	0·444% (0·188–0·890)	0·349% (0·158–0·671)	0·305% (0·148–0·572)	0·291% (0·136–0·508)

Data are presented as IFR (95% uncertainty interval)**.** IFR=infection–fatality ratio.

Among 190 countries and territories, the age-standardised IFR varied from 0·173% (95% UI 0·058–0·676) to 1·159% (0·728–2·013) on April 15, 2020, and from 0·117% (0·036–0·417) to 0·771% (0·036–0·417) on Jan 1, 2021 (table 2). Median country-specific age-standardised IFR decreased from 0·539% (interquartile range [IQR] 0·447–0·657) to 0·353% (IQR 0·293–0·433) during that period. All-age IFR varied from 0·077% (95% UI 0·023–0·320) to 2·683% (1·083–5·567) on April 15, 2020, and from 0·052% (0·015–0·204) to 1·708% (0·901–3·269) on Jan 1, 2021. Median country-specific all-age IFR decreased from 0·466% (IQR 0·223–0·840) to 0·314% (IQR 0·143–0·551) during that period. In addition to national estimates, IFRs for all subnational locations are provided (appendix 1 section 7, table S2).

## Discussion

Our analysis shows that the all-age COVID-19 IFR varied by a factor of more than 30 across countries and territories during the pre-vaccine era. Because IFR is strongly related to age, population age structure accounted for nearly three-quarters of variation in IFR estimates for in-sample countries on July 15, 2020. Age patterns for COVID-19 mortality and IFR both form a J-shaped curve, with the lowest risk occurring at approximately age 7 years. After age standardisation, many North American and European countries continued to have high IFRs despite having greater access to health-care resources. This finding is probably not attributable to differentials in death under-reporting because our analysis used an estimate of the true number of COVID-19 deaths, a concept we call total COVID-19 mortality, as the numerator of the IFR. Potential alternative explanations include high SARS-CoV-2 transmission rates in the care home population of some locations, a higher prevalence of comorbidities that increase the severity of COVID-19 disease, or other causes. Our analysis of change over time found that the global median IFR decreased by approximately 33% between April 15, 2020, and Jan 1, 2021, from 0·466% (IQR 0·223–0·840) to 0·314% (0·143–0·551).

Age is the strongest predictor of IFR variation, but the reason for elevated COVID-19 fatality at older ages is not yet clear. Research has focused on understanding the particular forms of immunosenescence that lead to severe COVID-19 outcomes,^26^ and non-immunological pathways might also have a role. Proposed non-immunological mechanisms include age-related endothelial damage, differences in clotting function, differences in the expression of angiotensin converting enzyme 2 receptors, and higher prevalence of comorbidities,^27^ among others. To our knowledge, only one other study has recognised the J-shaped nature of the age pattern for COVID-19 mortality and the IFR.^13^ This age pattern is common among infectious diseases.^28^ Some policy recommendations, such as school openings that would permit younger students (especially prekindergarten and kindergarten students) to return to the classroom before older students, have been based on the presumption that risk is lower for younger children.^29^ However, the age-specific IFR and mortality estimates show that children under age 5 years are at higher risk than children aged 10–14 years or even 15–19 years. Modelling studies often evaluate school-opening policies on the basis of anticipated health consequences to the community,^30^ which is appropriate given the exponential increase in COVID-19 fatality risk at older ages, but risk to the students themselves also warrants consideration. In addition, the J-shaped IFR curve strengthens the case for promoting vaccination among young children, a strategy that has proven effective for similar respiratory illnesses such as pneumonia.^31^

Analysis of the mortality rates by age suggests that mortality risk is shifted to younger ages in parts of south Asia, southeast Asia, sub-Saharan Africa, and the Middle East. There are several possible explanations. First, because of higher amounts of background disease in many of these areas,^32^ comorbidities, such as tuberculosis,^32^ might explain higher death rates at younger ages.^34^ Second, the pattern of social interaction might vary such that older individuals could be more effectively shielded by their families. Care facilities for older people in high-income countries have had high transmission rates and were hit particularly hard at the onset of the pandemic^35^ (appendix 1 section 5.6 shows a quantification of this effect). But where older individuals are cared for at home, this might lead to reduced transmission. Third, the pattern could be caused by bias in the data, in which older individuals in low-resource settings are not going to facilities to be tested and their deaths might be more likely to go undetected.^36^ With the data available at the time of this study, it is difficult to establish which of these explanations is most likely.

With research reporting the beneficial effect of glucocorticoids (eg, dexamethasone)^37^ on mortality in patients who are severely ill, we should expect the IFR to decline over time. In randomised clinical trials, monoclonal antibody therapy, remdesivir, and oxygenation strategies have shown promising improvements to intermediate clinical outcomes. These include decreasing hospitalisation rates,^5^ decreasing recovery time,^6^ and decreasing the need for mechanical ventilation.^7^ The evidence suggests that a range of improvements in clinical management have contributed to substantive improvements in clinical outcomes that are likely to decrease the IFR over time. Clinical management protocols could also have improved through experience, such as when and how to use mechanical ventilation and the adoption of simple measures such as prone positioning of patients in intensive care.^38^, ^39^ In the present study, the estimated 33% decrease in median IFR over 8 months is consistent with these expectations, but the result should be interpreted with caution. We constrained the time effect to be negative and included a weakly informative Gaussian prior on time for the global stage (but not the regional or location-specific stages) of the age-standardised IFR model. Each location's IFR time trend is thus informed by both the prior and empirical data. A sensitivity analysis removing the prior did not reduce the magnitude of the average time slope across locations (appendix 1 section 5.4). The magnitude of the prior comes from an analysis of 20 736 hospitalised COVID-19 patients in 107 US hospitals.^25^ We encoded the effect of obesity and other clinical predictors as priors on the basis of the same study. This synthesis of population-level and patient-level information is a strength of our modelling strategy that allows predictions to better reflect the current state of knowledge.

This study is subject to several limitations. First, if multiple age-specific seroprevalence surveys were done in the same location at different points in time, all of them were included in the analytic dataset. Because the IFR incorporates cumulative measures of deaths and infections, this modelling choice implicitly gives greater weight to age patterns occurring earlier in the pandemic. Second, although we ensured that the sampling frame of each serological study aligns with a GBD national or subnational location, sampling strategies are not guaranteed to be representative of a population. We attempted to address this limitation by careful determination of outliers in the age-standardised IFR model and additionally including a correction for sampling bias in the age-specific IFR model. Third, the analysis did not include surveys specifically dedicated to care facilities for older people, which might be under-sampled by general population surveys. Fourth, in principle, non-therapeutic explanations for the decrease in IFR over time are possible, such as shifts in age-specific contact rates and health-care systems becoming overwhelmed at different points in time. However, these explanations are not likely to influence the results because of the cumulative nature of the dependent variable (cumulative deaths divided by cumulative infections). For cumulative IFR to be affected by age-specific contact rates, for example, changes in the age distribution would need to persist for a long period. We are unaware of evidence to suggest that surges in transmission among one segment of a population are contained to that segment for an extended period. The effects of health-care constraints are similarly likely to be transitory. Fifth, antibody tests have variable sensitivity and specificity.^40^, ^41^, ^42^ Although several commercially available tests achieve sensitivity and specificity above 98%,^40^ tests with lower sensitivity and specificity are sometimes used.^43^ Low sensitivity would lead, all other things being equal, to an overestimate of the IFR, whereas in low-prevalence settings, lower specificity could appreciably underestimate the IFR. Because the sensitivity of anti-nucleocapsid and anti-spike antibody tests tends to decrease over time, we adjusted all-age and age-specific seroprevalence observations for the baseline and waning sensitivity corresponding to each individual test. Sixth, COVID-19 reported deaths undercount the total mortality related to COVID-19 as seen in various analyses of excess mortality.^44^ The larger number of deaths detected in the change in all-cause mortality as compared to reported COVID-19 has three important potential causes: under-registration of deaths directly because of COVID-19, such as the missed deaths in care facilities for older people and missed deaths caused by stricter criteria for diagnosis early in the pandemic; deaths from other causes exacerbated by COVID-19, such as some reports of ischaemic heart disease; or deaths that have increased because of physical distancing mandates and deferred care.^45^ We have attempted to adjust for death under-registration by using an estimate of the true number of deaths attributable to COVID-19 as the numerator of the IFR.^15^, ^16^ Seventh, another potential source of bias is outmigration. In selected settings with large epidemics such as New York, USA, individuals who are infected might have moved out to lower-prevalence states. This factor could raise seroprevalence in those locations but not increase mortality risk, leading to an under-estimate of the IFR in those locations. Many of the studies included here are national and much less likely to be affected by this migration bias. Taken together, there are many potential directions for bias with no clear consistent direction overall. Results here should be interpreted judiciously given the uncertainties in the data available.

Patterns of COVID mortality and IFR are profoundly related to age, but time-varying and location-specific factors also have a role. The risk of millions of additional deaths at the global level remains high until vaccines are widely and equitably deployed and more effective therapies developed and distributed widely.

Correspondence to: Dr Reed J D Sorensen, Institute for Health Metrics and Evaluation, University of Washington, Seattle, WA 98195, USA **rsoren@uw.edu**

## Data sharing

To download the data used in these analyses, please visit the Global Health Data Exchange website. Data sources are also listed by location and institution in appendix 2.

**This online publication has been corrected. The corrected version first appeared at thelancet.com on April 14, 2022**

## Declaration of interests

CA reports support for the present manuscript from the Benificus Foundation as funding support for the collection of data on state-level social-distancing policies in the USA. XD reports support for the present manuscript from the University of Washington through their employment at the Institute for Health Metrics and Evaluation. ADF reports stock or stock options in Agathos for technical advising, and other financial or non-financial interest from Janssen, SwissRe, Merck for Mothers, and Sanofi, for technical advising on simulation modelling, all outside the submitted work. NF reports receiving funding for work from WHO as a consultant from June, 2019, to September, 2019, and Gates Ventures since June, 2020, all outside the submitted work. SN reports support for the present manuscript from the Ministry of Education, Culture, Sports, Science and Technology of Japan as a grant. DMP report support for the present manuscript from the Bill & Melinda Gates Foundation. All other authors declare no competing interests.
