
JOURNAL INFORMATION
==============================
NLM Title Abbreviation: Lancet
Journal ID: Lancet
NLM Journal ID: 2985213R

Lancet (London, England)

ISSN: 0140-6736
EISSN: 1474-547X

ARTICLE INFORMATION
==============================
PMCID: PMC9009837
PMID: 35430020
DOI: 10.1016/S0140-6736(22)00666-3
Article ID: S0140-6736(22)00666-3
Article version: 2
Subjects: Department of Error

Department of Error

Print publication date: 2022 Apr 16
Volume: 399
Issue: 10334
First page: 1468
Last page: 1468
Copyright: © 2022 The Author(s). Published by Elsevier Ltd.
Copyright year: 2022
Copyright holder: Elsevier Ltd
License: This is an open access article under the CC BY license (http://creativecommons.org/licenses/by/4.0/).
License URL: https://creativecommons.org/licenses/by/4.0/

Erratum for: Variation in the COVID-19 infection–fatality ratio by age, time, and geography during the pre-vaccine era: a systematic analysis 399 10334 16 4 2022 1469 1488 Lancet (London, England) 10.1016/S0140-6736(21)02867-1 PMC8871594 35219376

==============================
COVID-19 Forecasting Team. Variation in the COVID-19 infection–fatality ratio by age, time, and geography during the pre-vaccine era: a systematic analysis. Lancet 2022; 399: 1469–88—In this Article, Tanzania and Uganda should have been listed under eastern sub-Saharan Africa in table 2. The totals for eastern sub-Saharan Africa correctly inclxnzania and Uganda in the original version and have not been changed. These corrections have been made to the online version as of April 14, 2022, and the printed version is correct.

