# Supplementary material for: Variation in the COVID-19 infection–fatality ratio by age, time, and geography during the pre-vaccine era: a systematic analysis
Source: Lancet. 2022 Apr 16;399(10334):1469–88. doi: 10.1016/S0140-6736(21)02867-1 (PMC8871594; doi:10.1016/S0140-6736(21)02867-1)
Supplement: Supplementary appendix 1 [file mmc1.pdf]

THE LANCET

Supplementary appendix

This appendix formed part of the original submission and has been peer reviewed. We post it as supplied by the authors.

Supplement to: COVID-19 Forecasting Team. Variation in the COVID-19 infection–fatality ratio by age, time, and geography during the pre-vaccine era: a systematic analysis. *Lancet* 2022; published online Feb 24. https://doi.org/10.1016/S0140-6736(21)02867-1.

Appendix 1: supplemental methods and results to “Variation in the COVID-19 infection-fatality ratio by age, time, and geography during the pre-vaccine era: a systematic analysis”

This appendix provides further methodological and supplementary results for “Variation in the COVID-19 infection-fatality ratio by age, time, and geography during the pre-vaccine era: a systematic analysis.”

Table of Contents

Section 1: List of abbreviations 2
Section 2: GATHER compliance 3
Section 3: Locations and time period of analysis 3
Section 4: Data inputs 3
Section 5: Methods 3
Section 5.1: Overview 3
Figure 5.1.1: IFR pipeline 3
Section 5.2: Mortality age patterns 4
Section 5.3: Age-specific infection-fatality ratio 5
Section 5.4: All-age and age-standardised infection-fatality ratio 6
Figure 5.4.1: Sensitivity decay functions for eight commercial serology assays 6
Figure 5.4.2: Distribution of time slopes by location in a model without a Gaussian prior on the time effect 8
Figure 5.4.3: Distribution of time slopes by location in a model with the Gaussian prior on the time effect 9
Section 5.5: Total COVID-19 mortality 9
Section 5.6: Potential impact of nursing home epidemics on population-level IFR 10
Figure 5.6.1: Age patterns of seroprevalence in Belgium and globally 11
Figure 5.6.2: Age-specific IFR for three location sets 11
Table 5.6.2: Percentage reduction in all-age IFR in the counterfactual compared to the baseline scenario 12
Section 6: References 13
Section 7: Supplemental tables 15
Table S1. GATHER Checklist 15
Table S2. COVID-19 infection-fatality ratios (IFR) in April 2020, July 2020, October 2020, and January 2021, all-age and age-standardised; countries, territories, and subnational locations 17
Section 8: Author contributions 21

Section 1: List of abbreviations

Abbreviation	Full phrase
AHA	American Heart Association
CDC	United States Centers for Disease Control and Prevention
COVID-19	coronavirus disease 2019
GATHER	Guidelines for Accurate and Transparent Health Estimates Reporting
GBD	Global Burden of Diseases, Injuries, and Risk Factors Study
IFR	Infection-fatality ratio
IHME	Institute for Health Metrics and Evaluation
MR-BRT	Meta-regression—Bayesian, regularised, trimmed
MRTool	Meta-Regression Tool
NPI	Non-pharmaceutical intervention
OECD	Organisation for Economic Co-operation and Development
SARS-CoV-2	severe acute respiratory syndrome coronavirus 2
UI	uncertainty interval
WHO	World Health Organization

Section 2: GATHER compliance

This study complies with GATHER recommendations.[1] We have documented the steps in our analytical procedures and detailed the data sources used. See section 7, table S1 for the GATHER checklist. The GATHER recommendations can be found on the GATHER website.

Section 3: Locations and time period of analysis

Age-specific seroprevalence studies were limited to those occurring before the date of vaccine introduction in each location. Most seroprevalence surveys occurred before January 1, 2021. Age-specific cumulative mortality data included the most recent observation prior to vaccine introduction in each location. All-age seroprevalence and mortality data include those used to estimate the full time series of COVID-19 deaths and infection-fatality ratio as of October 2021, although we report results only for January 1, 2021, and earlier. More information on data preparation, analysis and prediction for the all-age mortality parameters can be found in COVID-19 Excess Mortality Collaborators.[2,3] Note that methods describing how excess mortality contributes to the estimation of total COVID-19 mortality, the metric representing the numerator of the IFR, are detailed in section 5.5.

We made predictions of all-age and age-standardised IFR for 190 countries and territories, as well as subnational locations in 11 countries and territories. A full list of prediction locations can be found in section 7, table S2. We predicted all-age and age-standardised IFR for April 15, 2020; July 15, 2020; October 15, 2020; and January 1, 2021.

Section 4: Data inputs

Appendix 2 includes source lists for all-age deaths (appendix 2, section 1), age-specific deaths (appendix 2, section 2), all-age seroprevalence (appendix 2, section 3) and age-specific seroprevalence (appendix 2, section 4).

Section 5: Methods

Section 5.1: Overview

Figure 5.1.1: IFR pipeline

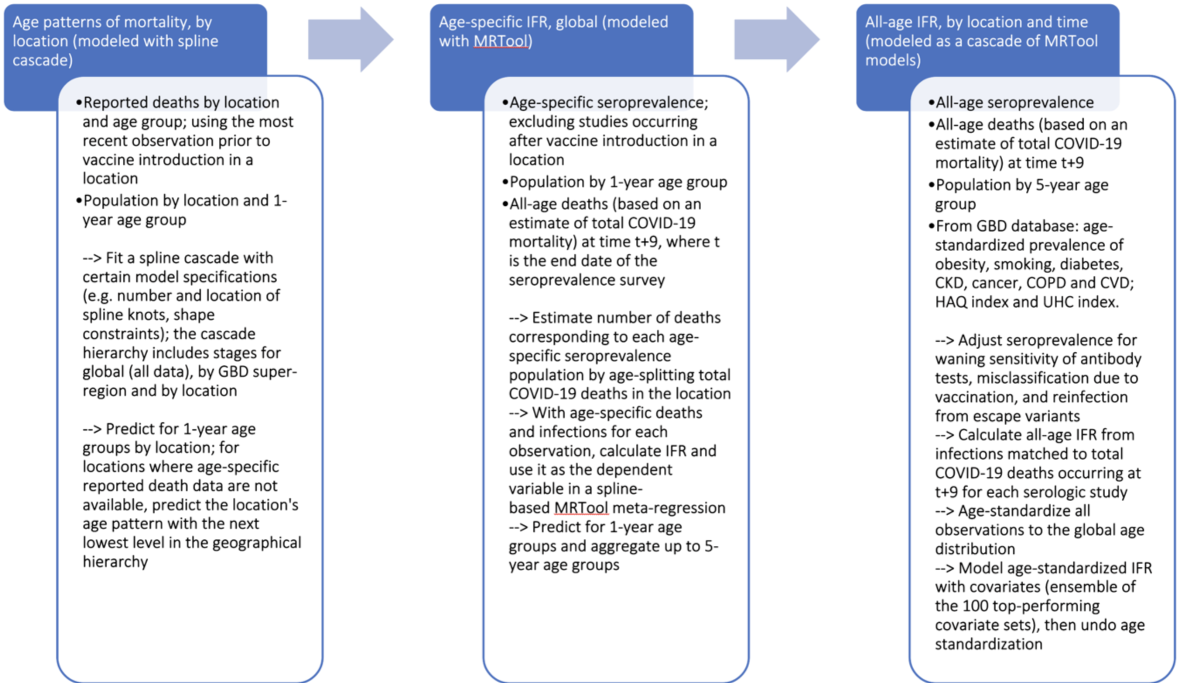

Blue boxes are modelled outputs. White boxes are inputs (bullets) and processes (arrows) needed for the associated blue box.

Section 5.2: Mortality age patterns

We first obtained relative mortality age patterns using age-specific reported death counts. This estimation was done using the spline cascade framework detailed in Causey et al.,[4] which we describe below. The dependent variable passed to the model was logit-scale cumulative deaths divided by population, modelled in a hierarchical cascade structure with age represented as a B-spline. We sourced age-specific mortality data primarily through the COVerAGE-DB database of COVID-19 cases and deaths by age,[5,6] with additional supplementation via national and state ministry of health dashboards where identified. In total, we obtained data from 213 locations and used the most recent observation of cumulative deaths prior to vaccine introduction in each location. National-level age-specific mortality data were available for 64 countries. Sources are listed in appendix 2 (section 2).

We implemented several data processing steps prior to modelling. First, the meta-regression package used to implement the spline cascade (MRTool)[7] required the dependent variable to be transformed into the appropriate space, which in the case of infection-fatality ratio was logit space. Because the logit of zero is negative infinity, we dealt with observations with mean zero by increasing the upper bound of the age group until there was a non-zero number of deaths. Second, because the dependent variable in a meta-regression is a random variable with uncertainty, transforming the dependent variable into logit space required a corresponding transformation of the random variable's standard deviation. For this, we used the delta method.[8] Third, MRTool has the capability of integrate over the entire interval of an age group, thus avoiding the need to make simplifying assumptions like taking the midpoint of the interval as the observed covariate value. This approach required special consideration for the open-ended age group, for example age 80 years and older, the upper bound of which is often represented as an arbitrarily high number. We modified the upper bound of the open age group such that midpoint of the new group was equal to the mean of the population distribution within the original group. We based this calculation on GBD 2019 population estimates.[9]

The model consisted of Bayesian meta-regression models that form a geographical cascade structure, representing the age effect as a cubic B-spline. Each model included a term for random location effects, except in the models low in the cascade where data came from only one location. In that case, the variance of between-location heterogeneity was constrained to be zero. The regression model can be represented as the following:

$$logit\left({}^{cumulative\ deaths}/_{population}\right) \sim spline(age) + random(location)$$

The spline cascade method was implemented by first fitting a model describing the global age pattern of logit-scale age-specific mortality rates and subsequently using the estimated spline coefficients as priors in models fit to super-region-specific subsets of the data. The coefficients of the super-region-specific models were then used as priors in models fit to national and administrative level 1 subsets of the data. In this way, information from the model where we had the most information (global model) partially informed the predictions for more detailed locations in the hierarchy. Predictions were made using the lowest available level in the geographical hierarchy. For example, if data existed for Peru, the predictions for Peru would be based on the country-specific model with prior information passed down from the model fit for the Latin America and Caribbean super-region. If data did not exist for Peru, predictions for Peru would come directly from the Latin America and Caribbean model informed by priors from the global model. Based on the observation in the raw data that the age trend of population mortality clearly decreased and then increased, we implemented a constraint that the age effect be monotonically increasing above age 10 years and free to follow the data below age 10 years. The model included four internal knots. The locations of the knots were determined by running the global model on each of 50 random knot sets and determining which knot set provided the best fit to the data. Once the model was fit, we obtained outputs for each one-year age group in each location, transformed to linear space, and normalised by the minimum value of total deaths/population, obtaining location-specific relative mortality age patterns. Appendix 3 shows global, region-specific, and location-specific fits overlaid on each location's age-specific mortality data.

Section 5.3: Age-specific infection-fatality ratio

Next, we obtained age-specific IFR estimates based on seroprevalence studies that reported results for at least two age groups. The model was a single MRTool meta-regression model that characterises the age effect as a B-spline and adjusts observations derived from non-representative samples according to a fixed effect for bias. We identified seroprevalence studies through a search protocol that leveraged prior reviews,[10,11] SeroTracker,[12] and routine inclusion of national and subnational surveys undertaken by governmental organisations. The search protocol yielded 5131 seroprevalence surveys. Of these, 1099 seroprevalence surveys occurred prior to vaccine introduction in a location and disaggregated the results by age. We further excluded seroprevalence surveys for which the source population was not geo-accordant with a national or administrative level 1 location or were otherwise determined to be an outlier later in the analytic pipeline. Exclusions later in the analytic pipeline were made considering a number of factors, including quality of the data source, representativeness of the sample, and plausibility of the seroprevalence value considering the full set of seroprevalence observations through time in a location. This process led to a total of 3012 age-specific seroprevalence observations from 718 studies.

We implemented several data processing steps. First, we adjusted the upper bound of the open-ended age interval such that the midpoint matches the mean of the population distribution within the group, as we did for mortality age pattern data preparation. Second, for each of the age-specific seroprevalence observations, we calculate cumulative age-specific infections at time t (end date of the seroprevalence study) by multiplying age-specific seroprevalence by the population of the age group according to GBD 2019 estimates of population.[9] The standard error of the seroprevalence observation was likewise multiplied by population to scale the uncertainty appropriately. Third, for each age-specific estimate of infections, we estimated the number of deaths occurring in that location and age-group at time t+9, where t is the end date of the seroprevalence survey. The period of nine days refers to the expected time between seroconversion and death. We implemented the age-splitting procedure by computing the weights corresponding to each age group, by multiplying the proportion of the population and the location-specific relative mortality pattern estimated by hierarchical spline cascade model:

$$age\ specific\ weight = population\ proportion\ for\ age * relative\ mortality\ pattern\ at\ age$$

After normalising the weights for each individual age such that the weights sum to 1, the number of deaths in a given age group is the total number of deaths (based on an estimate of total COVID-19 mortality; see section 5.5 for details)[2] multiplied by the sum of age-specific weights in the age group. With deaths and infections estimated for the age-specific seroprevalence observation, we calculated the standard error of an age-specific IFR observation as:

$$\mu_{IFR} = {}^{\mu_x}/_{\mu_y}$$

$$s_{IFR} = \sqrt{\left(\frac{\mu_x^2}{\mu_y^2}\right) * \left(\frac{s_x^2}{\mu_x^2} + \frac{s_y^2}{\mu_y^2}\right)}$$

where μ_x indicates the number of deaths occurring in the age group, μ_y indicates deaths plus infections occurring in the age group, and s_{IFR} is the standard error of the infection-fatality ratio. We included deaths in the denominator of the IFR μ_y to account for the fact that people who died of COVID-19 would not be available for a seroprevalence survey but should be counted. Fourth, we adjusted this IFR value for baseline sensitivity and waning sensitivity of antibody tests over time. The methods for this adjustment are described below in section 5.4. Finally, we used the delta method to convert the resulting IFR and its standard error into logit space. To avoid undue influence from a small number of serologic studies with particularly large sample sizes, which would otherwise dominate the shape of the estimated age-specific IFR curve, we set all standard errors to be the median observed standard error in the sample.

With logit-scale age-specific IFR observations in hand, we fit a mixed effects model:

$$Logit(IFR) \sim spline(age) + bias + random(study)$$

modelling logit(IFR) as a B-spline on age, study-specific random intercepts, and a risk-of-bias covariate. The bias covariate is an indicator of non-representative samples. As with the mortality age pattern model, age is represented as the full age interval (except for open-ended age-groups which have been truncated). With the fitted model we used the fixed effects (setting bias = 0) to predict a time-invariant global age-specific IFR curve for one-year age bins. The estimated coefficient for the risk-of-bias covariate was 0.00396 (standard error: 0.0493). The variance of between-study heterogeneity was 0.101.

Section 5.4: All-age and age-standardised infection-fatality ratio

We summarise the methodological approach modelling age-standardised IFR here. Observations of all-age seroprevalence underwent three corrections prior to statistical modelling. First, reported seroprevalence was corrected for the waning sensitivity of antibody tests over time. Each all-age seroprevalence survey received an adjustment that corresponds to the timing of infections in the past (according to the deaths time series divided by an un-adjusted time-varying estimate of IFR) and a sensitivity decay function. Sensitivity decay functions reported for each antibody test were extrapolated forward two years with smoothing splines (Figure 5.4.1 below).[13–15] Second, the all-age seroprevalence observations were corrected for the presence of vaccinations in a population (unpublished methods; COVID-19 Forecasting Team). The correction assumes that 90% of individuals receiving a COVID-19 vaccine would test positive. This correction was applied to surveys using anti-spike assays, except those that excluded vaccinated people from their sample. Third, all-age seroprevalence observations were corrected for potential reinfection from escape variants (unpublished methods; COVID-19 Forecasting Team). After this correction, the quantity represented by the seroprevalence observation is the percentage of the population with at least one SARS-CoV-2 infection.

Figure 5.4.1: Sensitivity decay functions for eight commercial serology assays

Source: unpublished estimates; COVID-19 Cumulative Infection Collaborators

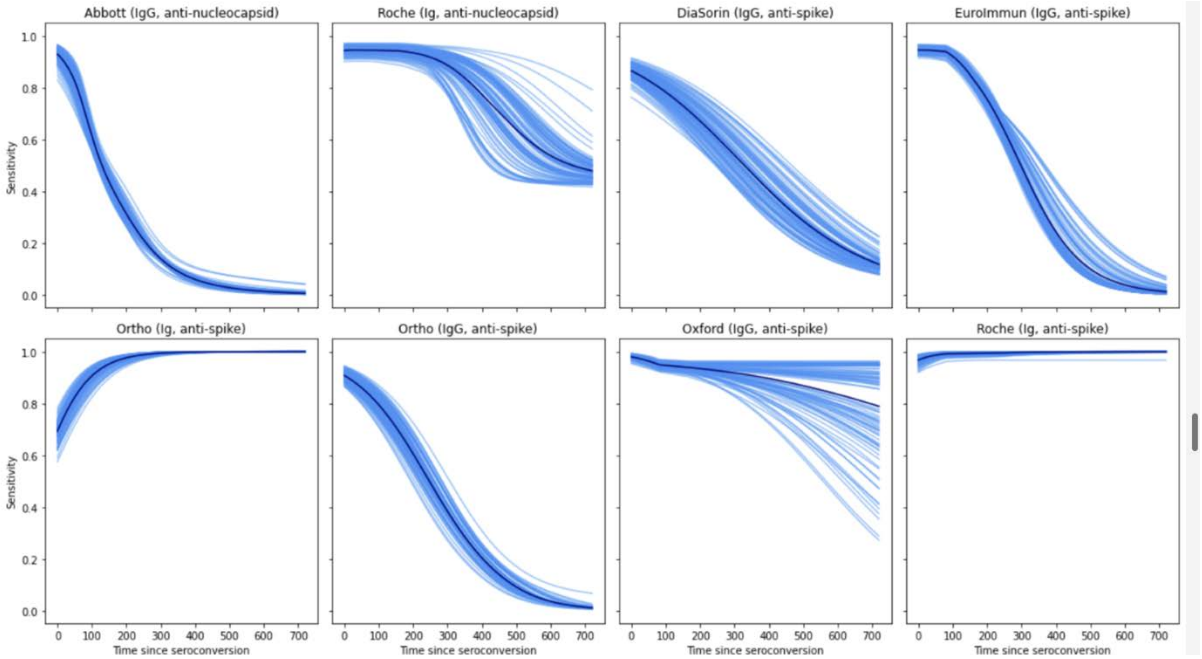

These corrected seroprevalence observations were converted into IFR observations by matching the number of infections (population * seroprevalence) to the number of total COVID-19 deaths occurring in the population at nine days after the end of the seroprevalence survey. Observations were indexed in time according to the mean date of deaths occurring in the population prior to the survey. Seroprevalence surveys with estimates less than 3% were excluded; surveys with sampling strategies not geo-accordant with a national or administrative level 1 GBD location were also excluded. Methods for obtaining the estimate of total COVID-19 deaths are summarised in section 5.5 of this appendix and described in detail by Wang and colleagues.[2]

Indirect age-standardisation required three inputs: the global IFR age pattern, the global seroprevalence age pattern, and population by location and five-year age group. Standardisation was implemented as:

$$r^g = \sum_{a=0}^{125} \frac{r_a^g s_a^g p_a^g}{s_a^g p_a^g}$$

$$r^l = \sum_{a=0}^{125} \frac{r_a^g s_a^g p_a^l}{s_a^g p_a^l}$$

$$s^l = \frac{r^g}{r^l}$$

where r is the infection-fatality ratio, g means global, l means location-specific, a means age-specific, s is seroprevalence, and p is population. The quantity s^l is the scalar by which we multiply observed all-age IFR to standardise it to the global age distribution. The resulting age-standardised IFR values and their measurement errors were transformed into logit space using the delta method.

Logit-scale age-standardised IFR served as the dependent variable in a geographically hierarchical set of models. We call this modelling approach a "cascade"; coefficients are first estimated using all data, and these are used as empirical priors in models fit to geographical subsets of the data. This process may be repeated multiple times for increasingly detailed geographical subsets of the data. Each model in the age-standardised IFR cascade included an

intercept, a piecewise-linear spline on time, and several covariates which we describe below. The cascade included stages for global, super-region, region, country, administrative unit level 1 locations, and administrative unit level 2 locations. The strength of the priors passed from stage to stage vary according to a user-defined parameter called lambda. Lambda is a stage-specific multiplier of the prior's standard deviation. The lambdas for the intercept term were 2, 2, 100, 100, and 100 for each non-global stage, respectively. The lambdas for time were 0.5, 1, 10, 10, and 10, respectively. The lambdas for ensemble covariates were all 1.

The model allowed IFR to vary as a function of time in days and several location-level covariates, which were incorporated as an ensemble of individual models. The assessed covariates included the age-standardised prevalence of obesity, smoking, diabetes, chronic kidney disease, cancer, chronic obstructive pulmonary disease, cardiovascular disease, as well as the Healthcare Access and Quality Index, and an index of universal health-care coverage. All 383 unique combinations of predictors (restricting UHC and HAQ Index from being included in the same model) were evaluated, and the top 100 sets were included in an ensemble of 100 models. The covariates are all estimates from the Global Burden of Disease study.[16] Effect sizes for the prevalence covariates were informed by Gaussian priors, which come from the log-scale odds ratios estimated in an analysis of hospitalised COVID-19 patients from 107 USA hospitals.[17] Time was parameterised as a piecewise linear spline with one knot; the time period earlier than the knot is constrained to have decreasing age-standardised IFR, and the time period after the knot is constrained to be constant. The location of the knot is empirically estimated by determining which knot location minimises the root mean square error in implied seroprevalence. The potential knot locations included the first day of each month between May 2020 and March 2021. The decreasing portion of time effect had a Gaussian prior of $N(-0{\cdot}002, 0{\cdot}001^2)$ in the global stage of the cascade. Models in the regional and location-specific stages of the cascade were specified without the Gaussian prior on the time effect.

To test the sensitivity of the results to the $N(-0{\cdot}002, 0{\cdot}001^2)$ prior on the time effect in the global model, we ran a separate model removing the prior. The magnitude of location-specific time slopes did not change substantially after removing the prior. The changes that did occur generally move the time effect away from the null (greater magnitude of the negative slope), indicating that the prior in the global model is not driving the time trend results. The parameter shown in figures 5.3 and 5.4 is, by location, the median daily difference in logit-scale IFR among non-zero values.

Figure 5.4.2: Distribution of time slopes by location in a model without a Gaussian prior on the time effect

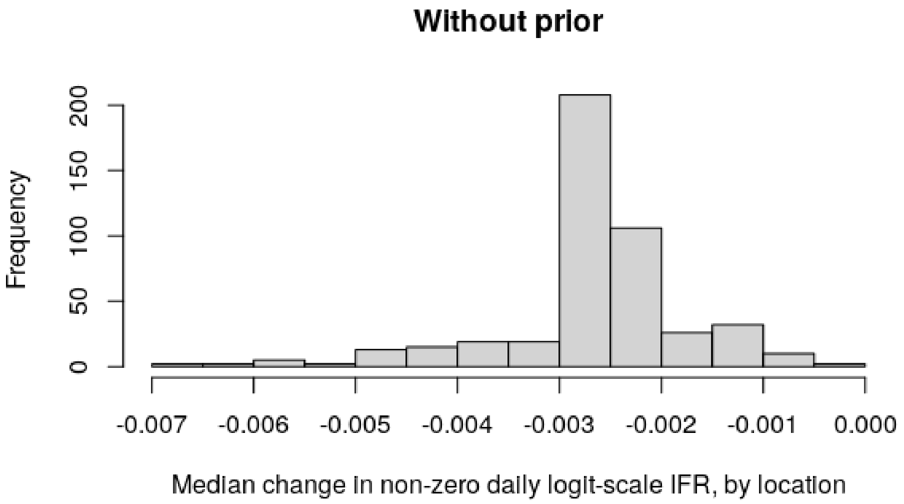

Min.	1st Qu.	Median	Mean	3rd Qu.	Max.
-0.0066937	-0.0026227	-0.0025397	-0.0025990	-0.0022838	-0.000210

Figure 5.4.3: Distribution of time slopes by location in a model with the Gaussian prior on the time effect

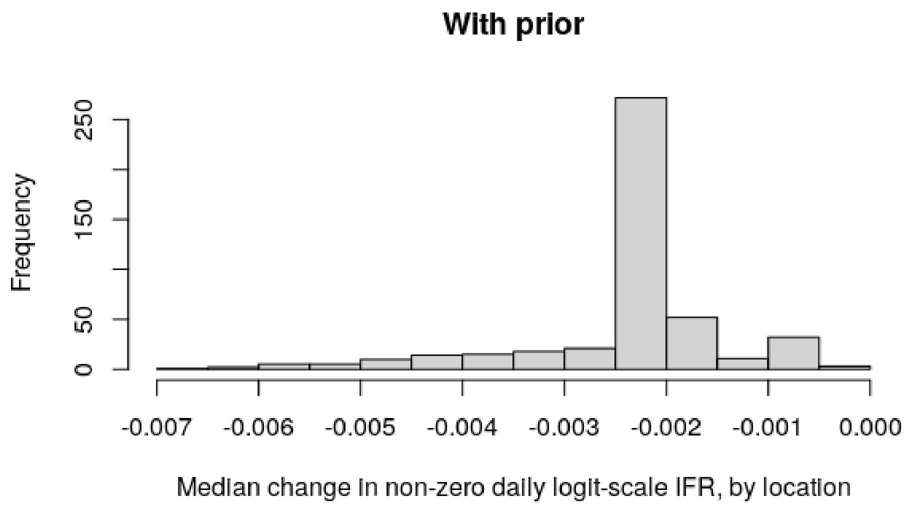

Min.	1st Qu.	Median	Mean	3rd Qu.	Max.
-0.0065094	-0.0023538	-0.0022161	-0.0023913	-0.0020705	-0.0001866

Section 5.5: Total COVID-19 mortality

Total COVID-19 mortality is an estimate of the true number of COVID-19 deaths occurring in a population, defined as all deaths where the deceased were actively infected by SARS-CoV-2 at the time of death. The method is an attempt to account for pervasive under-reporting of COVID-19 deaths in a globally comprehensive set of locations. Here, we summarise methods described by COVID-19 Excess Mortality Collaborators[2] and in unpublished methods (COVID-19 Cumulative Infection Collaborators) in sufficient detail to fully comprehend all of the elements contributing to this work. Total COVID-19 mortality is estimated in two stages: 1) estimating excess mortality during the pandemic, by location; and 2) estimating the fraction of excess mortality that is directly attributable to COVID-19.

Excess mortality was estimated using data from 69 countries and an additional 266 subnational locations where all-cause mortality data are reported by week or month during the COVID-19 epidemic. The data were corrected for late registration and under-registration when needed based on the completeness assessment from the Global Burden of Disease Study 2019.[9] With these data, estimates of expected mortality for various cumulative time periods specific to each location were made with an ensemble of six models. Details of the models in the ensemble are available elsewhere.[2,3] Excess mortality was calculated by comparing the number of deaths during the pandemic to the expected number of deaths based on trends from non-pandemic time periods.

These estimates are supplemented by excess mortality rates provided by the Medical Research Council of South Africa at the national and subnational level. Although time-specific data on all-cause mortality were not available for India, the analysis was supplemented by excess mortality rate estimated based on reported deaths from the Civil Registration System for 12 states for certain months in 2020 and 2021, and the deaths reported in the same time frames for year 2018 and 2019, after accounting for under-registration of the Civil Registration System in India by state.

With these estimates of excess mortality for locations with data as the dependent variable, another statistical model was developed to predict excess mortality rate for all locations for the period from January 1, 2020, to September

26, 2021. This model included the following covariates to aid in prediction: cumulative seroprevalence with lag, mobility with lag, infection-detection rate with lag, reported COVID-19 crude death rate, crude death rate for year 2019, prevalence of diabetes for year 2019, prevalence of smoking for year 2019, crude death rate due to HIV/AIDS for year 2019, inpatient admission rate for year 2019, quality of death registration system, average latitude, proportion of population over age 75 in year 2019, prevalence of hypertension for year 2019, and the Healthcare Access and Quality Index for year 2019. From a larger set of prospective covariates, these were the ones chosen by Least Absolute Shrinkage and Selection Operator (LASSO) that had effect sizes in the expected directions.

Finally, using the fitted model of excess mortality for all locations, the fraction of excess mortality estimated to be attributable to COVID-19 was calculated as a counterfactual. By location, total COVID-19 is obtained predicting from the fitted model setting mobility to pre-pandemic levels and the infection-detection rate to the maximum observed level among all locations. If this predicted counterfactual number of deaths was less than the reported number of deaths, the reported number of deaths was used instead.

Section 5.6: Potential impact of nursing home epidemics on population-level IFR

We conducted a post-hoc analysis to quantify the potential impact of nursing home epidemics on population-level IFR in 13 locations. The locations were Belgium; Canada; Ireland; and ten states of the United States. Belgium, Canada, and Ireland were chosen because they are the three countries with higher-than-average COVID-19 mortality rates among residents of long-term care (LTC) facilities relative to the community-dwelling population aged 65 and older, among the 12 OECD countries assessed by Sepulveda and colleagues.[18] Following the same criteria, the selected USA states (Connecticut, Delaware, Minnesota, New Hampshire, North Carolina, Ohio, Oregon, Rhode Island, Washington) are those which had a higher-than-average ratio of LTC mortality rates compared to mortality rates among the community-dwelling population aged 65 and older. New York was included as well after accounting for the way that New York City is isolated in the CDC reporting framework. Data on population size and cumulative COVID-19 deaths among residents of LTC facilities came from the Kaiser Family Foundation.[19,20] Among these locations determined to have elevated mortality in the LTC population, Belgium was the only location with serological data for detailed age groups above age 65 (Figure 5.5).

The goal of the analysis is to estimate the proportional reduction in IFR for each location under the hypothetical scenario that no LTC epidemic had occurred, assuming that the existing seroprevalence age pattern follows that of Belgium. Using the population age structure of each location, we calculated the number of infections and deaths as:

$$I(a) = N(a) * S(a)$$
$$D(a) = I(a) * IFR(a)$$

where I is infections, N is population, S is seroprevalence, D is deaths, IFR is the infection-fatality ratio, and a is a specific 5-year age group.

The second pane of Figure 5.5 shows the seroprevalence age patterns for the "LTC epidemic" and "No LTC epidemic", hereafter called the baseline and counterfactual scenarios, respectively. The baseline seroprevalence age pattern comes from fitting a meta-regression to age-specific serological data from Belgium. The model specification includes a spline on age group and random intercepts by serological study, setting measurement error to be constant. The counterfactual seroprevalence age pattern is the same global age pattern used for indirect standardisation of all-age IFRs in the main analysis.

Figure 5.6.1: Age patterns of seroprevalence in Belgium and globally

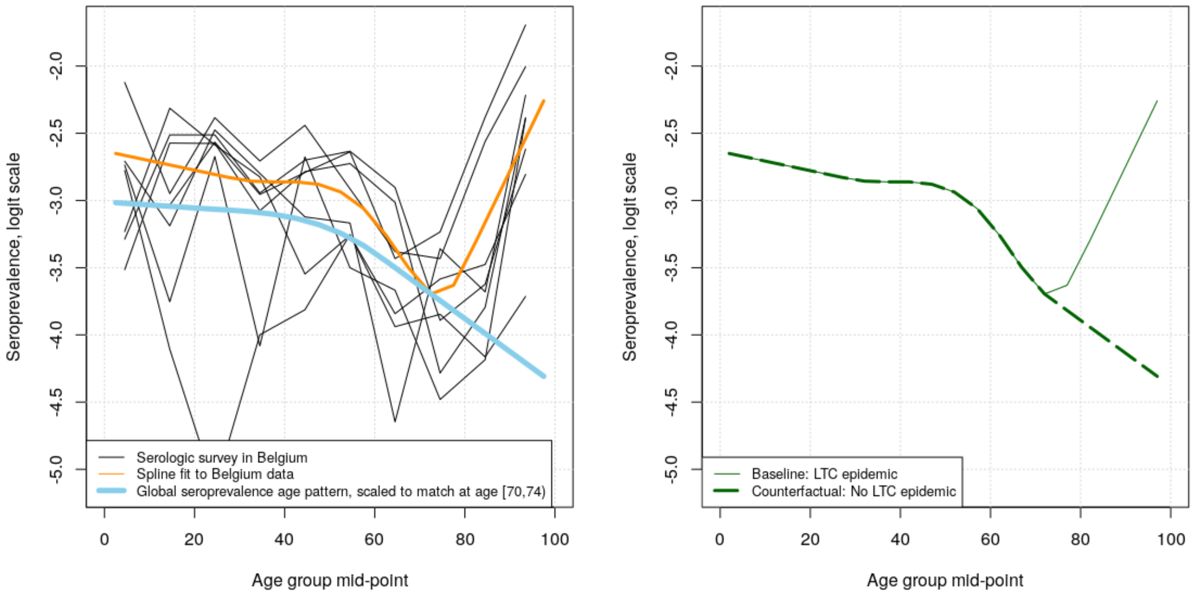

Figure 5.6.1 shows age-specific IFRs for the baseline and counterfactual scenarios. The baseline age-specific IFR estimates were derived from serological data in the 13 locations with elevated mortality in the LTC population (denoted “High income, NH epidemic”), and the counterfactual age-specific IFR estimates were derived from serological data in high-income locations not among the 13-location set (denoted “High income, other”).

Figure 5.6.2: Age-specific IFR for three location sets

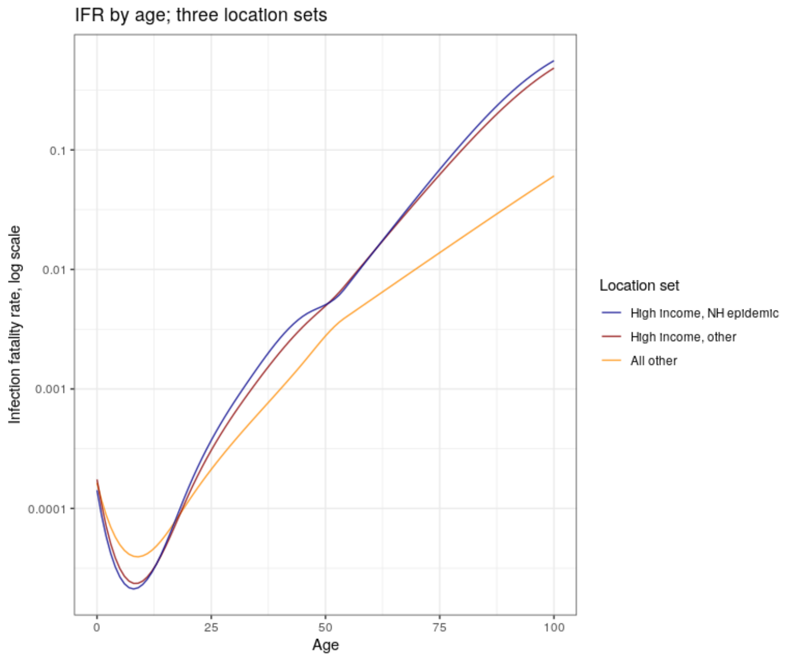

All-age IFR for each scenario was calculated as:

$$IFR = \frac{\Sigma_{a \in A} D}{\Sigma_{a \in A} I}$$

summing over age-specific deaths and infections. The percent reduction in IFR under the hypothetical scenario that no LTC epidemic had occurred is calculated as:

$$\theta_l = 100 \cdot (1 - (IFR_{lc}/IFR_{lb})),$$

where l means location-specific, c means counterfactual, and b means baseline.

The results of the analysis are given in Table 5.1. Reduction in IFR, using counterfactual age-specific IFR and seroprevalence age patterns, was 45.0% to 51.5% lower than baseline. Most of this difference is due to lower seroprevalence at older ages rather than higher age-specific IFR at older ages.

Table 5.6.2: Percentage reduction in all-age IFR in the counterfactual compared to the baseline scenario

Location	% reduction	% reduction changing seroprevalence only
Belgium	50.6	46.1
Ireland	43.6	39.2
Canada	47.4	43.1
Connecticut	51.0	46.9
Delaware	46.0	41.6
Minnesota	48.4	44.2
New Hampshire	45.0	40.7
New York	51.5	47.4
North Carolina	44.1	39.8
Ohio	48.4	44.1
Oregon	46.4	42.0
Rhode Island	51.5	47.4
Washington	45.4	41.1

Section 6: References

1 Stevens GA, Alkema L, Black RE, *et al.* Guidelines for Accurate and Transparent Health Estimates Reporting: the GATHER statement. *The Lancet* 2016; **388**: e19–23.

2 COVID-19 Excess Mortality Collaborators. Estimating excess mortality due to the SARS-CoV-2 pandemic: a systematic analysis of COVID-19 related mortality between January 1, 2020 and December 31, 2021. *Lancet* in press.

3 Institute for Health Metrics and Evaluation. Estimation of total and excess mortality due to COVID-19. 2021; published online Oct 15. http://www.healthdata.org/special-analysis/estimation-excess-mortality-due-covid-19-and-scalars-reported-covid-19-deaths (accessed Oct 15, 2021).

4 Causey K, Fullman N, Sorensen R, et al. Estimating global and regional disruptions to routine childhood vaccine coverage during the COVID-19 pandemic in 2020: a modelling study. *Lancet* 2021; **398**: 522–34.

5 Reiner RC, Barber RM, Collins JK, *et al.* Modeling COVID-19 scenarios for the United States. *Nat Med* 2021; **27**: 94–105.

6 Riffe T, Acosta E, Schöley J, Donzowa J. COVerAGE-DB: A database of COVID-19 cases and deaths by age. 2020; published online April 9. DOI:10.17605/OSF.IO/MPWJQ.

7 Zheng P, Barber R, Sorensen R, Murray C, Aravkin A. Trimmed constrained mixed effects models: formulations and algorithms. *J Comput Graph Stat* 2021; **0**: 1–34.

8 Oehlert GW. A Note on the delta method. *Am Stat* 1992; **46**: 27–9.

9 Wang H, Abbas KM, Abbasifard M, *et al.* Global age-sex-specific fertility, mortality, healthy life expectancy (HALE), and population estimates in 204 countries and territories, 1950–2019: a comprehensive demographic analysis for the Global Burden of Disease Study 2019. *Lancet* 2020; **396**: 1160–203.

10 Ioannidis, John P A. Infection fatality rate of COVID-19 inferred from seroprevalence data. *Bull World Health Organ* 2021; **99**: 19-33F.

11 Levin AT, Hanage WP, Owusu-Boaitey N, Cochran KB, Walsh SP, Meyerowitz-Katz G. Assessing the age specificity of infection fatality rates for COVID-19: systematic review, meta-analysis, and public policy implications. *Eur J Epidemiol* 2020; **35**: 1123–38.

12 Arora RK, Joseph A, Wyk JV, *et al.* SeroTracker: a global SARS-CoV-2 seroprevalence dashboard. *Lancet Infect Dis* 2021; **21**: e75–6.

13 Muecksch F, Wise H, Batchelor B, *et al.* Longitudinal serological analysis and neutralizing antibody levels in coronavirus disease 2019 convalescent patients. *J Infect Dis* 2021; **223**: 389–98.

14 Peluso MJ, Takahashi S, Hakim J, *et al.* SARS-CoV-2 antibody magnitude and detectability are driven by disease severity, timing, and assay. *medRxiv* 2021; : 2021.03.03.21251639.

15 Perez-Saez J, Zaballa M-E, Yerly S, *et al.* Persistence and detection of anti-SARS-CoV-2 antibodies: immunoassay heterogeneity and implications for serosurveillance. *medRxiv* 2021; : 2021.03.16.21253710.

16 Abbasi-Kangevari M, Abd-Allah F, Abdelalim A, *et al.* Global burden of 87 risk factors in 204 countries and territories, 1990–2019: a systematic analysis for the Global Burden of Disease Study 2019. *The Lancet* 2020; **396**: 1223–49.

17 Roth GA, Emmons-Bell S, Alger HM, *et al.* Trends in patient characteristics and COVID-19 in-hospital mortality in the United States during the COVID-19 pandemic. *JAMA Netw Open* 2021; **4**: e218828.

18 Sepulveda ER, Stall NM, Sinha SK. A Comparison of COVID-19 Mortality Rates Among Long-Term Care Residents in 12 OECD Countries. *J Am Med Dir Assoc* 2020; **21**: 1572-1574.e3.

19 Total Number of Residents in Certified Nursing Facilities. KFF. 2021; published online June 30. https://www.kff.org/other/state-indicator/number-of-nursing-facility-residents/ (accessed Oct 27, 2021).

20 Oct 27 P, 2021. State COVID-19 Data and Policy Actions. KFF. 2021; published online Oct 27. https://www.kff.org/coronavirus-covid-19/issue-brief/state-covid-19-data-and-policy-actions/ (accessed Oct 27, 2021).

Section 7: Supplemental tables

Table S1. GATHER Checklist

Item #	Checklist item	Reported on page #
**Objectives and funding**		
**1**	Define the indicator(s), populations (including age, sex, and geographic entities), and time period(s) for which estimates were made.	Main text: Introduction (last paragraph); Appendix 1, section 3
**2**	List the funding sources for the work.	Main text: "Funding" section of the summary, and "Acknowledgments" section
**Data Inputs**		
For all data inputs from multiple sources that are synthesized as part of the study:		
**3**	Describe how the data were identified and how the data were accessed.	Main text: "Mortality by age" subsection of Methods and "Infection fatality ratio by age" subsection of Methods; Appendix 2 lists all data sources
**4**	Specify the inclusion and exclusion criteria. Identify all ad-hoc exclusions.	Main text: "Mortality by age", "Infection-fatality ratio by age", and "Age-standardised and all-age IFR" subsections of the Methods; appendix 1, section 5.3
**5**	Provide information on all included data sources and their main characteristics. For each data source used, report reference information or contact name/institution, population represented, data collection method, year(s) of data collection, sex and age range, diagnostic criteria or measurement method, and sample size, as relevant.	Appendix 2; online data citation tool: http://ghdx.healthdata.org/record/ihme-data/covid_19_infection_fatality_ratio
**6**	Identify and describe any categories of input data that have potentially important biases (e.g., based on characteristics listed in item 5).	Main text: "Mortality by age" and "Infection-fatality ratio by age" subsections of Methods; appendix 2
For data inputs that contribute to the analysis but were not synthesized as part of the study:		
**7**	Describe and give sources for any other data inputs.	Appendix 1, section 5.4 (GBD covariates)
For all data inputs:		
**8**	Provide all data inputs in a file format from which data can be efficiently extracted (e.g., a spreadsheet rather than a PDF), including all relevant meta-data listed in item 5. For any data inputs that cannot be shared because of ethical or legal reasons, such as third-party ownership, provide a contact name or the name of the institution that retains the right to the data.	Institution names for all data inputs by location are given in appendix 2; http://ghdx.healthdata.org/record/ihme-data/covid_19_infection_fatality_ratio
**Data analysis**		
**9**	Provide a conceptual overview of the data analysis method. A diagram may be helpful.	Main text: "Overview" subsection of the Methods
**10**	Provide a detailed description of all steps of the analysis, including mathematical formulae. This description should cover, as relevant, data cleaning, data pre-processing, data adjustments and weighting of data sources, and mathematical or statistical model(s).	Main text: short description of all steps of the analysis given in the Methods section<br>Appendix 1, section 5: detailed description of all steps of the analysis.
**11**	Describe how candidate models were evaluated and how the final model(s) were selected.	Appendix 1, section 5
**12**	Provide the results of an evaluation of model performance, if done, as well as the results of any relevant sensitivity analysis.	Appendix 1, section 5
**13**	Describe methods for calculating uncertainty of the estimates. State which sources of uncertainty were, and were not, accounted for in the uncertainty analysis.	Main text: "Infection-fatality ratio by age" subsection of Methods; Appendix 1, section 5
**14**	State how analytic or statistical source code used to generate estimates can be accessed.	Github link: https://github.com/ihmeuw/covid-age-patterns and https://github.com/ihmeuw/covid-historical-model
**Results and Discussion**		
**15**	Provide published estimates in a file format from which data can be efficiently extracted.	http://ghdx.healthdata.org/record/ihme-data/covid_19_infection_fatality_ratio
**16**	Report a quantitative measure of the uncertainty of the estimates (e.g. uncertainty intervals).	Main text: Table 1, Table 2, Table S2

**17**	Interpret results in light of existing evidence. If updating a previous set of estimates, describe the reasons for changes in estimates.	Main text: "Research in Context: Evidence before this study" and Introduction section
**18**	Discuss limitations of the estimates. Include a discussion of any modelling assumptions or data limitations that affect interpretation of the estimates.	Main text: "Mortality by age", "Infection-fatality ratio by age", and "Age-standardised and all-age infection-fatality ratio" subsections of the Methods; Discussion section; appendix 1, section 5

Table S2. COVID-19 infection-fatality ratios (IFR) in April 2020, July 2020, October 2020, and January 2021; all-age and age-standardised; countries, territories, and subnational locations

Location	IFR (%), April 15, 2020	IFR (%), July 15, 2020	IFR (%), October 15, 2020	IFR (%), January 1, 2021	IFR (%), April 15, 2020, age-standardized	IFR (%), July 15, 2020, age-standardized	IFR (%), October 15, 2020, age-standardized	IFR (%), January 1, 2021, age-standardized
**Central Europe, eastern Europe, and central Asia**								
**Central Asia**								
Armenia	0.494 (0.202, 0.945)	0.386 (0.187, 0.756)	0.335 (0.163, 0.589)	0.318 (0.156, 0.546)	0.374 (0.153, 0.716)	0.292 (0.142, 0.573)	0.254 (0.123, 0.446)	0.241 (0.118, 0.414)
Azerbaijan	0.284 (0.117, 0.544)	0.222 (0.107, 0.432)	0.193 (0.095, 0.337)	0.183 (0.090, 0.312)	0.375 (0.154, 0.717)	0.293 (0.141, 0.570)	0.254 (0.125, 0.444)	0.241 (0.119, 0.412)
Georgia	0.425 (0.177, 1.072)	0.331 (0.147, 0.852)	0.287 (0.141, 0.704)	0.271 (0.138, 0.698)	0.248 (0.103, 0.625)	0.193 (0.086, 0.497)	0.167 (0.082, 0.411)	0.158 (0.081, 0.407)
Kazakhstan	0.222 (0.066, 0.534)	0.172 (0.053, 0.422)	0.150 (0.047, 0.345)	0.142 (0.045, 0.337)	0.272 (0.081, 0.654)	0.210 (0.064, 0.516)	0.183 (0.057, 0.422)	0.174 (0.055, 0.412)
Kyrgyzstan	0.228 (0.094, 0.433)	0.178 (0.086, 0.348)	0.154 (0.076, 0.272)	0.147 (0.073, 0.252)	0.372 (0.154, 0.708)	0.291 (0.141, 0.568)	0.252 (0.124, 0.444)	0.240 (0.119, 0.412)
Mongolia	0.216 (0.089, 0.412)	0.169 (0.082, 0.329)	0.146 (0.072, 0.257)	0.139 (0.069, 0.238)	0.377 (0.155, 0.719)	0.295 (0.142, 0.575)	0.256 (0.126, 0.448)	0.243 (0.121, 0.415)
Tajikistan	0.157 (0.065, 0.295)	0.122 (0.059, 0.237)	0.106 (0.053, 0.187)	0.101 (0.051, 0.173)	0.364 (0.152, 0.688)	0.285 (0.138, 0.552)	0.247 (0.123, 0.435)	0.235 (0.118, 0.403)
Turkmenistan	0.249 (0.102, 0.479)	0.194 (0.093, 0.376)	0.169 (0.083, 0.293)	0.160 (0.079, 0.273)	0.376 (0.153, 0.722)	0.293 (0.141, 0.567)	0.254 (0.126, 0.442)	0.242 (0.119, 0.412)
Uzbekistan	0.230 (0.080, 0.441)	0.180 (0.075, 0.338)	0.158 (0.068, 0.293)	0.151 (0.068, 0.273)	0.476 (0.165, 0.912)	0.373 (0.156, 0.699)	0.327 (0.140, 0.605)	0.312 (0.140, 0.564)
**Central Europe**								
Albania	0.391 (0.113, 0.901)	0.308 (0.094, 0.687)	0.270 (0.082, 0.546)	0.258 (0.074, 0.542)	0.254 (0.073, 0.586)	0.200 (0.061, 0.447)	0.176 (0.053, 0.355)	0.168 (0.048, 0.352)
Bosnia and Herzegovina	0.705 (0.335, 1.228)	0.552 (0.295, 0.961)	0.479 (0.269, 0.754)	0.456 (0.260, 0.722)	0.400 (0.190, 0.698)	0.314 (0.167, 0.546)	0.272 (0.153, 0.428)	0.259 (0.148, 0.410)
Bulgaria	0.873 (0.415, 1.518)	0.684 (0.364, 1.188)	0.594 (0.335, 0.932)	0.565 (0.321, 0.894)	0.403 (0.192, 0.701)	0.316 (0.168, 0.549)	0.274 (0.155, 0.430)	0.261 (0.148, 0.413)
Croatia	0.864 (0.401, 1.654)	0.674 (0.375, 1.323)	0.584 (0.355, 1.050)	0.555 (0.347, 0.929)	0.402 (0.187, 0.771)	0.314 (0.175, 0.616)	0.272 (0.165, 0.489)	0.258 (0.162, 0.433)
Czechia	0.584 (0.307, 1.153)	0.454 (0.270, 0.922)	0.394 (0.250, 0.736)	0.375 (0.247, 0.652)	0.287 (0.151, 0.568)	0.224 (0.133, 0.454)	0.194 (0.123, 0.362)	0.184 (0.122, 0.321)
Hungary	0.835 (0.397, 1.446)	0.654 (0.345, 1.136)	0.568 (0.320, 0.891)	0.540 (0.306, 0.856)	0.401 (0.191, 0.695)	0.314 (0.166, 0.545)	0.273 (0.153, 0.428)	0.260 (0.147, 0.411)
Montenegro	0.613 (0.292, 1.063)	0.480 (0.254, 0.833)	0.417 (0.235, 0.653)	0.397 (0.225, 0.628)	0.403 (0.192, 0.699)	0.316 (0.167, 0.548)	0.274 (0.154, 0.430)	0.261 (0.148, 0.413)
North Macedonia	0.546 (0.260, 0.953)	0.428 (0.228, 0.745)	0.372 (0.208, 0.584)	0.354 (0.201, 0.559)	0.402 (0.191, 0.702)	0.315 (0.168, 0.549)	0.274 (0.154, 0.430)	0.260 (0.148, 0.412)
Poland	0.872 (0.381, 1.751)	0.680 (0.322, 1.395)	0.591 (0.290, 1.107)	0.563 (0.275, 0.992)	0.466 (0.203, 0.936)	0.363 (0.172, 0.746)	0.316 (0.155, 0.592)	0.301 (0.147, 0.530)
Romania	0.808 (0.387, 1.393)	0.633 (0.334, 1.093)	0.550 (0.312, 0.863)	0.523 (0.298, 0.828)	0.402 (0.192, 0.692)	0.315 (0.166, 0.543)	0.273 (0.155, 0.429)	0.260 (0.148, 0.412)
Serbia	0.711 (0.337, 1.234)	0.557 (0.297, 0.970)	0.483 (0.270, 0.761)	0.460 (0.259, 0.729)	0.402 (0.191, 0.698)	0.315 (0.168, 0.549)	0.273 (0.153, 0.430)	0.260 (0.147, 0.413)
Slovakia	0.668 (0.317, 1.160)	0.523 (0.277, 0.912)	0.454 (0.254, 0.715)	0.432 (0.243, 0.685)	0.397 (0.189, 0.690)	0.311 (0.165, 0.543)	0.270 (0.151, 0.426)	0.257 (0.144, 0.408)
Slovenia	1.785 (0.459, 3.957)	1.390 (0.384, 3.091)	1.199 (0.339, 2.396)	1.137 (0.331, 2.298)	0.797 (0.205, 1.766)	0.620 (0.171, 1.379)	0.535 (0.151, 1.069)	0.508 (0.148, 1.026)
**Eastern Europe**								
Belarus	0.709 (0.300, 1.275)	0.556 (0.252, 0.974)	0.484 (0.244, 0.783)	0.461 (0.238, 0.749)	0.417 (0.177, 0.751)	0.328 (0.149, 0.574)	0.285 (0.144, 0.461)	0.272 (0.140, 0.441)
Estonia	1.313 (0.504, 2.631)	1.025 (0.422, 2.069)	0.886 (0.394, 1.595)	0.838 (0.385, 1.354)	0.609 (0.234, 1.221)	0.476 (0.196, 0.960)	0.411 (0.183, 0.740)	0.389 (0.179, 0.629)
Latvia	0.936 (0.400, 1.674)	0.734 (0.332, 1.289)	0.640 (0.321, 1.027)	0.609 (0.314, 0.983)	0.418 (0.179, 0.748)	0.328 (0.148, 0.576)	0.286 (0.144, 0.459)	0.272 (0.140, 0.439)
Lithuania	0.920 (0.393, 1.653)	0.722 (0.327, 1.264)	0.629 (0.318, 1.015)	0.599 (0.311, 0.971)	0.415 (0.177, 0.746)	0.326 (0.148, 0.570)	0.284 (0.143, 0.458)	0.270 (0.140, 0.438)
Moldova	0.644 (0.276, 1.154)	0.505 (0.229, 0.880)	0.440 (0.224, 0.707)	0.419 (0.219, 0.677)	0.412 (0.177, 0.738)	0.323 (0.146, 0.563)	0.282 (0.143, 0.452)	0.268 (0.140, 0.433)
Russia	0.276 (0.092, 1.080)	0.217 (0.077, 0.858)	0.194 (0.064, 0.702)	0.187 (0.058, 0.666)	0.173 (0.058, 0.676)	0.136 (0.048, 0.537)	0.121 (0.040, 0.440)	0.117 (0.036, 0.417)
Ukraine	0.732 (0.314, 1.314)	0.574 (0.260, 1.004)	0.500 (0.253, 0.805)	0.476 (0.247, 0.770)	0.418 (0.179, 0.750)	0.328 (0.148, 0.573)	0.286 (0.144, 0.459)	0.272 (0.141, 0.440)
**High income**								
**Australasia**								
Australia	1.190 (0.792, 1.825)	0.924 (0.707, 1.419)	0.809 (0.642, 1.117)	0.772 (0.592, 1.033)	0.682 (0.454, 1.046)	0.530 (0.405, 0.813)	0.464 (0.368, 0.640)	0.443 (0.339, 0.592)
New Zealand	1.217 (0.810, 1.866)	0.945 (0.723, 1.454)	0.827 (0.657, 1.143)	0.790 (0.605, 1.056)	0.686 (0.456, 1.052)	0.533 (0.407, 0.819)	0.466 (0.370, 0.644)	0.445 (0.341, 0.595)
**High-income Asia Pacific**								
Brunei	0.407 (0.274, 0.618)	0.316 (0.236, 0.488)	0.277 (0.220, 0.380)	0.264 (0.199, 0.356)	0.694 (0.467, 1.054)	0.539 (0.402, 0.832)	0.472 (0.375, 0.648)	0.450 (0.339, 0.606)
Japan	2.252 (1.535, 3.444)	1.750 (1.302, 2.690)	1.531 (1.211, 2.106)	1.461 (1.086, 1.984)	0.696 (0.474, 1.064)	0.541 (0.402, 0.831)	0.473 (0.374, 0.651)	0.452 (0.336, 0.613)
Singapore	0.923 (0.627, 1.403)	0.717 (0.544, 1.100)	0.628 (0.502, 0.862)	0.599 (0.454, 0.807)	0.686 (0.466, 1.042)	0.533 (0.404, 0.817)	0.466 (0.373, 0.640)	0.445 (0.337, 0.600)
South Korea	1.154 (0.786, 1.765)	0.897 (0.669, 1.382)	0.785 (0.621, 1.081)	0.749 (0.557, 1.014)	0.695 (0.473, 1.062)	0.540 (0.403, 0.832)	0.472 (0.374, 0.651)	0.451 (0.335, 0.610)
**High-income North America**								
Canada	1.059 (0.188, 2.946)	0.815 (0.174, 2.275)	0.707 (0.162, 1.760)	0.673 (0.162, 1.669)	0.544 (0.096, 1.513)	0.418 (0.089, 1.168)	0.363 (0.083, 0.904)	0.345 (0.083, 0.857)
Alberta	3.192 (0.188, 7.621)	1.500 (0.174, 4.065)	1.078 (0.162, 2.278)	0.995 (0.162, 2.101)	1.639 (0.096, 3.913)	0.770 (0.089, 2.087)	0.553 (0.083, 1.170)	0.511 (0.083, 1.079)
British Columbia	1.059 (0.188, 2.946)	0.815 (0.174, 2.275)	0.707 (0.162, 1.760)	0.673 (0.162, 1.669)	0.544 (0.096, 1.513)	0.418 (0.089, 1.168)	0.363 (0.083, 0.904)	0.345 (0.083, 0.857)
Manitoba	1.661 (0.249, 4.916)	0.955 (0.230, 2.510)	0.751 (0.230, 1.760)	0.695 (0.213, 1.669)	0.853 (0.128, 2.524)	0.490 (0.118, 1.289)	0.386 (0.118, 0.904)	0.357 (0.109, 0.857)
New Brunswick	1.059 (0.188, 2.946)	0.815 (0.174, 2.275)	0.707 (0.162, 1.760)	0.673 (0.162, 1.669)	0.544 (0.096, 1.513)	0.418 (0.089, 1.168)	0.363 (0.083, 0.904)	0.345 (0.083, 0.857)
Newfoundland and Labrador	1.059 (0.188, 2.946)	0.815 (0.174, 2.275)	0.707 (0.162, 1.760)	0.673 (0.162, 1.669)	0.544 (0.096, 1.513)	0.418 (0.089, 1.168)	0.363 (0.083, 0.904)	0.345 (0.083, 0.857)
Northwest Territories	1.059 (0.188, 2.946)	0.815 (0.174, 2.275)	0.707 (0.162, 1.760)	0.673 (0.162, 1.669)	0.544 (0.096, 1.513)	0.418 (0.089, 1.168)	0.363 (0.083, 0.904)	0.345 (0.083, 0.857)
Nova Scotia	1.059 (0.188, 2.946)	0.815 (0.174, 2.275)	0.707 (0.162, 1.760)	0.673 (0.162, 1.669)	0.544 (0.096, 1.513)	0.418 (0.089, 1.168)	0.363 (0.083, 0.904)	0.345 (0.083, 0.857)
Nunavut	1.059 (0.188, 2.946)	0.815 (0.174, 2.275)	0.707 (0.162, 1.760)	0.673 (0.162, 1.669)	0.544 (0.096, 1.513)	0.418 (0.089, 1.168)	0.363 (0.083, 0.904)	0.345 (0.083, 0.857)
Ontario	1.059 (0.188, 2.946)	0.815 (0.174, 2.275)	0.707 (0.162, 1.760)	0.673 (0.162, 1.669)	0.544 (0.096, 1.513)	0.418 (0.089, 1.168)	0.363 (0.083, 0.904)	0.345 (0.083, 0.857)
Quebec	1.059 (0.188, 2.946)	0.815 (0.174, 2.275)	0.707 (0.162, 1.760)	0.673 (0.162, 1.669)	0.544 (0.096, 1.513)	0.418 (0.089, 1.168)	0.363 (0.083, 0.904)	0.345 (0.083, 0.857)
Saskatchewan	1.037 (0.154, 2.946)	0.676 (0.099, 2.275)	0.561 (0.093, 1.760)	0.529 (0.088, 1.669)	0.532 (0.079, 1.513)	0.347 (0.051, 1.168)	0.288 (0.048, 0.904)	0.272 (0.045, 0.857)
Yukon	1.059 (0.188, 2.946)	0.815 (0.174, 2.275)	0.707 (0.162, 1.760)	0.673 (0.162, 1.669)	0.544 (0.096, 1.513)	0.418 (0.089, 1.168)	0.363 (0.083, 0.904)	0.345 (0.083, 0.857)
USA	1.280 (0.771, 1.877)	0.909 (0.698, 1.353)	0.791 (0.635, 1.086)	0.754 (0.589, 0.995)	0.733 (0.442, 1.075)	0.521 (0.400, 0.775)	0.453 (0.364, 0.622)	0.432 (0.338, 0.570)
Alabama	1.242 (0.682, 2.223)	0.951 (0.672, 1.621)	0.905 (0.667, 1.355)	0.890 (0.667, 1.281)	0.708 (0.389, 1.268)	0.543 (0.383, 0.925)	0.516 (0.381, 0.773)	0.508 (0.381, 0.731)
Alaska	0.513 (0.239, 0.777)	0.334 (0.226, 0.503)	0.284 (0.210, 0.374)	0.270 (0.185, 0.357)	0.406 (0.189, 0.614)	0.264 (0.179, 0.397)	0.225 (0.166, 0.295)	0.214 (0.146, 0.282)
Arizona	1.898 (1.003, 3.186)	1.296 (0.894, 2.105)	1.119 (0.825, 1.707)	1.065 (0.775, 1.454)	1.062 (0.562, 1.784)	0.725 (0.501, 1.178)	0.626 (0.462, 0.956)	0.596 (0.434, 0.814)
Arkansas	1.617 (0.826, 2.548)	1.136 (0.735, 1.782)	1.014 (0.714, 1.438)	0.974 (0.714, 1.358)	0.909 (0.465, 1.433)	0.639 (0.414, 1.003)	0.570 (0.402, 0.809)	0.548 (0.402, 0.764)
California	1.242 (0.738, 2.232)	0.950 (0.633, 1.425)	0.886 (0.620, 1.297)	0.866 (0.618, 1.235)	0.770 (0.458, 1.383)	0.589 (0.392, 0.883)	0.549 (0.384, 0.804)	0.537 (0.383, 0.765)
Colorado	1.119 (0.588, 2.015)	0.715 (0.448, 1.315)	0.610 (0.436, 0.975)	0.577 (0.411, 0.837)	0.702 (0.369, 1.264)	0.449 (0.281, 0.825)	0.383 (0.273, 0.612)	0.362 (0.258, 0.525)
Connecticut	2.309 (1.379, 3.731)	1.268 (0.855, 1.743)	0.997 (0.713, 1.476)	0.924 (0.538, 1.459)	1.176 (0.702, 1.901)	0.646 (0.435, 0.888)	0.508 (0.363, 0.752)	0.471 (0.274, 0.743)
Delaware	1.829 (1.008, 3.017)	1.419 (0.926, 2.082)	1.299 (0.884, 1.801)	1.257 (0.866, 1.794)	0.961 (0.530, 1.585)	0.746 (0.486, 1.094)	0.682 (0.464, 0.947)	0.661 (0.455, 0.943)
Washington, DC	1.755 (0.967, 3.451)	1.043 (0.781, 1.420)	0.878 (0.617, 1.166)	0.829 (0.520, 1.166)	1.195 (0.659, 2.350)	0.710 (0.532, 0.967)	0.598 (0.420, 0.794)	0.564 (0.354, 0.794)
Florida	1.139 (0.715, 1.948)	0.829 (0.650, 1.255)	0.764 (0.613, 1.094)	0.742 (0.577, 1.012)	0.519 (0.326, 0.888)	0.378 (0.296, 0.572)	0.348 (0.279, 0.498)	0.338 (0.263, 0.461)
Georgia	1.131 (0.636, 1.973)	0.766 (0.588, 1.058)	0.685 (0.529, 0.912)	0.662 (0.470, 0.875)	0.774 (0.435, 1.350)	0.524 (0.402, 0.724)	0.469 (0.362, 0.624)	0.453 (0.322, 0.599)
Hawaii	1.041 (0.399, 2.308)	0.614 (0.302, 1.037)	0.531 (0.300, 0.959)	0.509 (0.270, 0.920)	0.516 (0.198, 1.145)	0.304 (0.150, 0.514)	0.264 (0.149, 0.476)	0.253 (0.134, 0.456)
Idaho	0.878 (0.393, 1.564)	0.596 (0.348, 1.042)	0.512 (0.329, 0.803)	0.487 (0.328, 0.687)	0.543 (0.243, 0.968)	0.369 (0.215, 0.645)	0.317 (0.204, 0.497)	0.302 (0.203, 0.425)
Illinois	1.517 (0.693, 2.558)	0.875 (0.607, 1.471)	0.714 (0.564, 1.046)	0.669 (0.448, 0.950)	0.880 (0.402, 1.484)	0.508 (0.352, 0.854)	0.414 (0.327, 0.607)	0.388 (0.260, 0.551)
Indiana	1.797 (0.923, 3.104)	1.196 (0.793, 2.034)	1.017 (0.743, 1.397)	0.958 (0.725, 1.287)	1.057 (0.543, 1.826)	0.704 (0.467, 1.197)	0.598 (0.437, 0.822)	0.564 (0.427, 0.757)
Iowa	0.643 (0.419, 1.345)	0.510 (0.397, 0.656)	0.504 (0.397, 0.605)	0.501 (0.397, 0.605)	0.332 (0.216, 0.694)	0.263 (0.205, 0.338)	0.260 (0.205, 0.312)	0.259 (0.205, 0.312)
Kansas	0.888 (0.517, 1.749)	0.691 (0.501, 0.949)	0.677 (0.501, 0.949)	0.674 (0.501, 0.949)	0.518 (0.302, 1.021)	0.403 (0.293, 0.554)	0.395 (0.293, 0.554)	0.393 (0.293, 0.554)
Kentucky	1.196 (0.610, 2.482)	0.897 (0.558, 1.393)	0.833 (0.546, 1.284)	0.813 (0.546, 1.275)	0.693 (0.353, 1.438)	0.520 (0.323, 0.807)	0.483 (0.316, 0.744)	0.471 (0.316, 0.739)
Louisiana	1.234 (0.774, 2.196)	1.081 (0.774, 1.675)	1.025 (0.765, 1.503)	1.006 (0.747, 1.493)	0.754 (0.473, 1.342)	0.661 (0.473, 1.024)	0.626 (0.467, 0.919)	0.614 (0.457, 0.912)
Maine	1.808 (0.689, 3.564)	1.216 (0.567, 2.309)	1.035 (0.562, 1.907)	0.976 (0.552, 1.600)	0.827 (0.315, 1.631)	0.556 (0.260, 1.056)	0.474 (0.257, 0.872)	0.446 (0.252, 0.732)
Maryland	1.594 (0.815, 2.950)	1.154 (0.708, 2.235)	0.999 (0.643, 1.625)	0.946 (0.595, 1.436)	0.950 (0.486, 1.758)	0.688 (0.422, 1.332)	0.595 (0.383, 0.968)	0.563 (0.355, 0.856)
Massachusetts	3.284 (1.510, 8.114)	1.670 (1.200, 2.284)	1.331 (0.944, 1.983)	1.243 (0.763, 1.983)	1.725 (0.793, 4.263)	0.877 (0.630, 1.200)	0.699 (0.496, 1.042)	0.653 (0.401, 1.042)
Michigan	1.630 (0.891, 2.937)	1.135 (0.768, 1.877)	0.984 (0.722, 1.419)	0.934 (0.662, 1.344)	0.849 (0.464, 1.529)	0.591 (0.400, 0.977)	0.512 (0.376, 0.739)	0.486 (0.344, 0.700)
Minnesota	0.677 (0.379, 1.565)	0.507 (0.379, 0.728)	0.481 (0.379, 0.601)	0.473 (0.379, 0.594)	0.383 (0.214, 0.884)	0.286 (0.214, 0.411)	0.272 (0.214, 0.340)	0.267 (0.214, 0.335)
Mississippi	1.967 (0.947, 3.735)	1.305 (0.811, 2.510)	1.101 (0.793, 1.734)	1.039 (0.722, 1.445)	1.181 (0.569, 2.243)	0.783 (0.487, 1.507)	0.661 (0.476, 1.041)	0.624 (0.434, 0.868)
Missouri	1.079 (0.577, 2.065)	0.788 (0.522, 1.230)	0.726 (0.507, 1.078)	0.706 (0.505, 1.032)	0.593 (0.317, 1.134)	0.433 (0.287, 0.675)	0.399 (0.279, 0.592)	0.388 (0.277, 0.567)
Montana	1.233 (0.590, 1.933)	0.828 (0.534, 1.269)	0.702 (0.530, 0.973)	0.664 (0.507, 0.907)	0.616 (0.294, 0.965)	0.413 (0.266, 0.634)	0.350 (0.265, 0.486)	0.331 (0.253, 0.453)
Nebraska	0.531 (0.272, 1.051)	0.412 (0.268, 0.719)	0.390 (0.260, 0.629)	0.383 (0.260, 0.578)	0.304 (0.156, 0.602)	0.236 (0.153, 0.412)	0.223 (0.149, 0.360)	0.219 (0.149, 0.331)
Nevada	0.892 (0.556, 1.558)	0.676 (0.541, 0.960)	0.649 (0.538, 0.901)	0.639 (0.538, 0.882)	0.591 (0.368, 1.032)	0.448 (0.358, 0.635)	0.430 (0.356, 0.597)	0.423 (0.356, 0.584)
New Hampshire	1.904 (0.880, 3.419)	1.254 (0.676, 2.148)	1.038 (0.644, 1.628)	0.973 (0.611, 1.442)	0.972 (0.449, 1.744)	0.640 (0.345, 1.096)	0.529 (0.329, 0.831)	0.496 (0.312, 0.736)
New Jersey	1.779 (1.197, 2.854)	1.182 (0.924, 1.785)	0.995 (0.705, 1.405)	0.939 (0.598, 1.358)	0.962 (0.648, 1.544)	0.640 (0.500, 0.966)	0.538 (0.381, 0.760)	0.508 (0.323, 0.734)
New Mexico	1.472 (0.738, 2.695)	1.093 (0.705, 1.914)	1.004 (0.673, 1.628)	0.975 (0.649, 1.459)	0.806 (0.404, 1.477)	0.599 (0.386, 1.049)	0.550 (0.369, 0.892)	0.534 (0.356, 0.800)
New York	1.268 (0.929, 2.034)	1.159 (0.834, 1.783)	1.101 (0.792, 1.612)	1.079 (0.758, 1.597)	0.679 (0.497, 1.089)	0.621 (0.447, 0.954)	0.589 (0.424, 0.863)	0.578 (0.406, 0.855)
North Carolina	1.481 (0.802, 2.631)	1.053 (0.585, 1.612)	0.937 (0.567, 1.324)	0.901 (0.555, 1.274)	0.878 (0.475, 1.559)	0.624 (0.346, 0.955)	0.555 (0.336, 0.784)	0.534 (0.329, 0.755)
North Dakota	1.249 (0.508, 2.987)	0.846 (0.464, 1.519)	0.770 (0.464, 1.228)	0.750 (0.464, 1.167)	0.657 (0.267, 1.571)	0.445 (0.244, 0.799)	0.405 (0.244, 0.646)	0.394 (0.244, 0.614)
Ohio	1.235 (0.663, 2.152)	0.861 (0.550, 1.619)	0.747 (0.522, 1.236)	0.709 (0.499, 1.093)	0.656 (0.352, 1.144)	0.458 (0.292, 0.861)	0.397 (0.278, 0.657)	0.377 (0.265, 0.581)
Oklahoma	1.156 (0.651, 2.019)	0.849 (0.541, 1.284)	0.779 (0.538, 1.123)	0.757 (0.538, 1.081)	0.687 (0.387, 1.199)	0.504 (0.321, 0.762)	0.463 (0.320, 0.667)	0.450 (0.320, 0.642)
Oregon	1.157 (0.469, 2.558)	0.899 (0.465, 1.603)	0.847 (0.465, 1.432)	0.831 (0.465, 1.358)	0.605 (0.245, 1.338)	0.470 (0.243, 0.838)	0.443 (0.243, 0.749)	0.435 (0.243, 0.711)
Pennsylvania	1.125 (0.802, 2.282)	0.989 (0.796, 1.365)	0.972 (0.796, 1.330)	0.966 (0.796, 1.308)	0.556 (0.396, 1.128)	0.489 (0.393, 0.675)	0.480 (0.393, 0.657)	0.477 (0.393, 0.647)
Rhode Island	2.639 (1.231, 4.675)	1.510 (0.950, 2.214)	1.216 (0.831, 1.669)	1.135 (0.750, 1.669)	1.335 (0.623, 2.365)	0.764 (0.481, 1.120)	0.615 (0.420, 0.845)	0.574 (0.380, 0.845)
South Carolina	1.751 (0.797, 2.942)	1.225 (0.762, 1.932)	1.081 (0.727, 1.530)	1.036 (0.706, 1.460)	0.986 (0.449, 1.656)	0.690 (0.429, 1.088)	0.609 (0.409, 0.861)	0.583 (0.397, 0.822)
South Dakota	1.103 (0.634, 1.870)	0.817 (0.531, 1.247)	0.744 (0.531, 1.015)	0.720 (0.518, 0.956)	0.591 (0.340, 1.003)	0.438 (0.285, 0.668)	0.399 (0.285, 0.544)	0.386 (0.277, 0.513)
Tennessee	0.988 (0.569, 1.889)	0.734 (0.569, 1.025)	0.707 (0.569, 1.002)	0.699 (0.562, 0.963)	0.567 (0.327, 1.084)	0.421 (0.327, 0.588)	0.406 (0.327, 0.575)	0.401 (0.323, 0.553)
Texas	1.126 (0.669, 1.910)	0.783 (0.580, 1.184)	0.698 (0.529, 1.023)	0.671 (0.506, 0.947)	0.813 (0.483, 1.378)	0.565 (0.419, 0.854)	0.504 (0.382, 0.738)	0.484 (0.365, 0.683)
Utah	0.414 (0.218, 0.765)	0.305 (0.210, 0.513)	0.287 (0.209, 0.429)	0.282 (0.207, 0.429)	0.350 (0.184, 0.647)	0.258 (0.178, 0.434)	0.243 (0.177, 0.363)	0.238 (0.176, 0.363)
Vermont	4.722 (1.352, 9.756)	3.096 (1.092, 5.788)	2.649 (0.974, 4.662)	2.498 (0.974, 4.242)	2.244 (0.643, 4.636)	1.471 (0.519, 2.751)	1.259 (0.463, 2.216)	1.187 (0.463, 2.016)
Virginia	1.704 (0.808, 3.266)	1.262 (0.741, 2.092)	1.146 (0.721, 1.910)	1.108 (0.712, 1.897)	1.039 (0.492, 1.991)	0.770 (0.451, 1.275)	0.699 (0.440, 1.164)	0.676 (0.434, 1.157)
Washington	1.009 (0.540, 2.098)	0.794 (0.534, 1.227)	0.761 (0.506, 1.110)	0.749 (0.506, 1.110)	0.590 (0.315, 1.226)	0.464 (0.312, 0.717)	0.445 (0.295, 0.649)	0.438 (0.295, 0.649)
West Virginia	2.396 (1.124, 4.024)	1.758 (0.985, 2.882)	1.628 (0.924, 2.586)	1.593 (0.924, 2.410)	1.148 (0.538, 1.928)	0.842 (0.472, 1.381)	0.780 (0.443, 1.239)	0.763 (0.443, 1.155)
Wisconsin	0.743 (0.352, 1.478)	0.528 (0.352, 0.888)	0.476 (0.352, 0.649)	0.459 (0.352, 0.619)	0.403 (0.191, 0.802)	0.286 (0.191, 0.482)	0.258 (0.191, 0.352)	0.249 (0.191, 0.336)
Wyoming	0.934 (0.426, 1.566)	0.645 (0.390, 1.079)	0.557 (0.385, 0.896)	0.530 (0.385, 0.878)	0.550 (0.251, 0.922)	0.380 (0.230, 0.635)	0.328 (0.227, 0.527)	0.312 (0.227, 0.517)
**Southern Latin America**								
Argentina	0.825 (0.548, 1.259)	0.641 (0.487, 0.986)	0.560 (0.448, 0.773)	0.535 (0.412, 0.716)	0.700 (0.464, 1.068)	0.543 (0.413, 0.836)	0.475 (0.380, 0.655)	0.453 (0.349, 0.608)
Chile	0.924 (0.617, 1.416)	0.718 (0.547, 1.106)	0.628 (0.502, 0.871)	0.599 (0.461, 0.806)	0.713 (0.476, 1.093)	0.554 (0.422, 0.854)	0.485 (0.387, 0.672)	0.463 (0.356, 0.622)
Uruguay	1.171 (0.779, 1.792)	0.910 (0.690, 1.404)	0.796 (0.634, 1.101)	0.759 (0.585, 1.020)	0.712 (0.474, 1.089)	0.553 (0.419, 0.853)	0.484 (0.385, 0.669)	0.462 (0.356, 0.620)
**Western Europe**								
Andorra	1.115 (0.585, 1.816)	0.875 (0.422, 1.429)	0.772 (0.368, 1.301)	0.737 (0.360, 1.264)	0.625 (0.328, 1.019)	0.491 (0.237, 0.802)	0.433 (0.207, 0.730)	0.413 (0.202, 0.709)
Austria	1.267 (0.552, 2.284)	0.984 (0.482, 1.682)	0.863 (0.456, 1.602)	0.823 (0.438, 1.591)	0.593 (0.258, 1.070)	0.461 (0.226, 0.787)	0.404 (0.214, 0.750)	0.386 (0.205, 0.745)
Belgium	1.672 (1.105, 2.754)	1.251 (0.793, 2.137)	1.086 (0.698, 1.776)	1.034 (0.641, 1.608)	0.784 (0.518, 1.291)	0.587 (0.372, 1.002)	0.509 (0.327, 0.833)	0.485 (0.301, 0.754)

Cyprus	1.143 (0.814, 1.883)	0.895 (0.637, 1.459)	0.788 (0.565, 1.183)	0.754 (0.517, 1.099)	0.802 (0.571, 1.322)	0.629 (0.447, 1.024)	0.553 (0.397, 0.831)	0.529 (0.363, 0.772)
Denmark	1.837 (0.953, 2.881)	1.446 (0.788, 2.190)	1.279 (0.680, 1.974)	1.226 (0.665, 1.964)	0.898 (0.466, 1.409)	0.707 (0.385, 1.071)	0.626 (0.332, 0.965)	0.600 (0.325, 0.960)
Finland	1.443 (0.313, 3.904)	1.125 (0.256, 3.013)	0.966 (0.256, 2.325)	0.911 (0.256, 2.128)	0.614 (0.133, 1.662)	0.479 (0.109, 1.283)	0.411 (0.109, 0.990)	0.388 (0.109, 0.906)
France	0.858 (0.588, 1.397)	0.674 (0.422, 1.176)	0.595 (0.374, 1.102)	0.570 (0.321, 1.054)	0.384 (0.263, 0.625)	0.302 (0.189, 0.526)	0.266 (0.167, 0.493)	0.255 (0.144, 0.472)
Germany	1.959 (1.389, 3.233)	1.535 (1.087, 2.504)	1.351 (0.979, 2.030)	1.293 (0.895, 1.879)	0.809 (0.574, 1.335)	0.634 (0.449, 1.034)	0.558 (0.404, 0.838)	0.534 (0.370, 0.776)
Baden-Württemberg	1.959 (1.389, 3.233)	1.535 (1.087, 2.504)	1.351 (0.979, 2.030)	1.293 (0.895, 1.879)	0.809 (0.574, 1.335)	0.634 (0.449, 1.034)	0.558 (0.404, 0.838)	0.534 (0.370, 0.776)
Bavaria	1.959 (1.389, 3.233)	1.535 (1.087, 2.504)	1.351 (0.979, 2.030)	1.293 (0.895, 1.879)	0.809 (0.574, 1.335)	0.634 (0.449, 1.034)	0.558 (0.404, 0.838)	0.534 (0.370, 0.776)
Berlin	1.959 (1.389, 3.233)	1.535 (1.087, 2.504)	1.351 (0.979, 2.030)	1.293 (0.895, 1.879)	0.809 (0.574, 1.335)	0.634 (0.449, 1.034)	0.558 (0.404, 0.838)	0.534 (0.370, 0.776)
Brandenburg	1.959 (1.389, 3.233)	1.535 (1.087, 2.504)	1.351 (0.979, 2.030)	1.293 (0.895, 1.879)	0.809 (0.574, 1.335)	0.634 (0.449, 1.034)	0.558 (0.404, 0.838)	0.534 (0.370, 0.776)
Bremen	1.959 (1.389, 3.233)	1.535 (1.087, 2.504)	1.351 (0.979, 2.030)	1.293 (0.895, 1.879)	0.809 (0.574, 1.335)	0.634 (0.449, 1.034)	0.558 (0.404, 0.838)	0.534 (0.370, 0.776)
Hamburg	1.959 (1.389, 3.233)	1.535 (1.087, 2.504)	1.351 (0.979, 2.030)	1.293 (0.895, 1.879)	0.809 (0.574, 1.335)	0.634 (0.449, 1.034)	0.558 (0.404, 0.838)	0.534 (0.370, 0.776)
Hesse	1.959 (1.389, 3.233)	1.535 (1.087, 2.504)	1.351 (0.979, 2.030)	1.293 (0.895, 1.879)	0.809 (0.574, 1.335)	0.634 (0.449, 1.034)	0.558 (0.404, 0.838)	0.534 (0.370, 0.776)
Lower Saxony	1.959 (1.389, 3.233)	1.535 (1.087, 2.504)	1.351 (0.979, 2.030)	1.293 (0.895, 1.879)	0.809 (0.574, 1.335)	0.634 (0.449, 1.034)	0.558 (0.404, 0.838)	0.534 (0.370, 0.776)
Mecklenburg-Vorpommern	1.959 (1.389, 3.233)	1.535 (1.087, 2.504)	1.351 (0.979, 2.030)	1.293 (0.895, 1.879)	0.809 (0.574, 1.335)	0.634 (0.449, 1.034)	0.558 (0.404, 0.838)	0.534 (0.370, 0.776)
North Rhine-Westphalia	1.959 (1.389, 3.233)	1.535 (1.087, 2.504)	1.351 (0.979, 2.030)	1.293 (0.895, 1.879)	0.809 (0.574, 1.335)	0.634 (0.449, 1.034)	0.558 (0.404, 0.838)	0.534 (0.370, 0.776)
Rhineland-Palatinate	1.959 (1.389, 3.233)	1.535 (1.087, 2.504)	1.351 (0.979, 2.030)	1.293 (0.895, 1.879)	0.809 (0.574, 1.335)	0.634 (0.449, 1.034)	0.558 (0.404, 0.838)	0.534 (0.370, 0.776)
Saarland	1.959 (1.389, 3.233)	1.535 (1.087, 2.504)	1.351 (0.979, 2.030)	1.293 (0.895, 1.879)	0.809 (0.574, 1.335)	0.634 (0.449, 1.034)	0.558 (0.404, 0.838)	0.534 (0.370, 0.776)
Saxony	1.959 (1.389, 3.233)	1.535 (1.087, 2.504)	1.351 (0.979, 2.030)	1.293 (0.895, 1.879)	0.809 (0.574, 1.335)	0.634 (0.449, 1.034)	0.558 (0.404, 0.838)	0.534 (0.370, 0.776)
Saxony-Anhalt	1.959 (1.389, 3.233)	1.535 (1.087, 2.504)	1.351 (0.979, 2.030)	1.293 (0.895, 1.879)	0.809 (0.574, 1.335)	0.634 (0.449, 1.034)	0.558 (0.404, 0.838)	0.534 (0.370, 0.776)
Schleswig-Holstein	1.959 (1.389, 3.233)	1.535 (1.087, 2.504)	1.351 (0.979, 2.030)	1.293 (0.895, 1.879)	0.809 (0.574, 1.335)	0.634 (0.449, 1.034)	0.558 (0.404, 0.838)	0.534 (0.370, 0.776)
Thuringia	1.959 (1.389, 3.233)	1.535 (1.087, 2.504)	1.351 (0.979, 2.030)	1.293 (0.895, 1.879)	0.809 (0.574, 1.335)	0.634 (0.449, 1.034)	0.558 (0.404, 0.838)	0.534 (0.370, 0.776)
Greece	2.089 (1.478, 3.450)	1.637 (1.155, 2.678)	1.441 (1.041, 2.166)	1.378 (0.951, 2.006)	0.821 (0.580, 1.355)	0.643 (0.454, 1.052)	0.566 (0.409, 0.851)	0.541 (0.374, 0.788)
Iceland	1.307 (0.924, 2.150)	1.024 (0.734, 1.669)	0.902 (0.655, 1.354)	0.862 (0.597, 1.245)	0.788 (0.557, 1.297)	0.617 (0.442, 1.007)	0.544 (0.395, 0.816)	0.520 (0.360, 0.751)
Ireland	1.216 (0.861, 2.006)	0.952 (0.679, 1.553)	0.839 (0.609, 1.261)	0.802 (0.555, 1.163)	0.800 (0.566, 1.320)	0.627 (0.447, 1.021)	0.552 (0.401, 0.830)	0.528 (0.365, 0.765)
Israel	0.527 (0.328, 1.003)	0.408 (0.301, 0.735)	0.362 (0.253, 0.680)	0.348 (0.225, 0.680)	0.428 (0.266, 0.815)	0.332 (0.245, 0.597)	0.294 (0.206, 0.553)	0.283 (0.183, 0.553)
Italy	1.721 (1.220, 2.791)	1.368 (0.855, 2.154)	1.211 (0.806, 1.840)	1.161 (0.763, 1.805)	0.655 (0.464, 1.062)	0.521 (0.325, 0.820)	0.461 (0.307, 0.700)	0.442 (0.291, 0.687)
Abruzzo	1.811 (1.283, 2.938)	1.439 (0.900, 2.268)	1.275 (0.849, 1.936)	1.221 (0.803, 1.903)	0.654 (0.463, 1.061)	0.520 (0.325, 0.819)	0.460 (0.307, 0.699)	0.441 (0.290, 0.687)
Basilicata	1.782 (1.263, 2.877)	1.416 (0.888, 2.225)	1.254 (0.833, 1.909)	1.201 (0.791, 1.866)	0.652 (0.462, 1.053)	0.518 (0.325, 0.814)	0.459 (0.305, 0.698)	0.440 (0.290, 0.683)
Calabria	1.575 (1.118, 2.531)	1.252 (0.786, 1.957)	1.108 (0.737, 1.682)	1.062 (0.700, 1.646)	0.645 (0.458, 1.036)	0.512 (0.322, 0.801)	0.454 (0.302, 0.689)	0.435 (0.287, 0.674)
Campania	1.387 (0.984, 2.234)	1.103 (0.687, 1.727)	0.976 (0.644, 1.489)	0.935 (0.618, 1.444)	0.663 (0.471, 1.068)	0.527 (0.328, 0.826)	0.467 (0.308, 0.712)	0.447 (0.296, 0.690)
Emilia-Romagna	1.853 (1.308, 3.023)	1.473 (0.916, 2.319)	1.304 (0.865, 1.984)	1.250 (0.819, 1.945)	0.659 (0.466, 1.076)	0.524 (0.326, 0.825)	0.464 (0.308, 0.706)	0.445 (0.292, 0.692)
Friuli-Venezia Giulia	2.003 (1.416, 3.262)	1.592 (0.995, 2.511)	1.410 (0.937, 2.145)	1.351 (0.884, 2.108)	0.656 (0.464, 1.068)	0.521 (0.326, 0.823)	0.462 (0.307, 0.703)	0.442 (0.290, 0.690)
Lazio	1.681 (1.184, 2.745)	1.336 (0.827, 2.102)	1.183 (0.783, 1.797)	1.133 (0.744, 1.757)	0.665 (0.469, 1.087)	0.529 (0.327, 0.832)	0.468 (0.310, 0.711)	0.449 (0.294, 0.695)
Liguria	2.387 (1.662, 3.805)	1.796 (1.069, 2.952)	1.588 (0.863, 2.400)	1.523 (0.838, 2.366)	0.691 (0.481, 1.101)	0.520 (0.309, 0.854)	0.459 (0.250, 0.695)	0.441 (0.242, 0.685)
Lombardia	1.520 (1.114, 2.152)	1.147 (0.627, 1.804)	1.021 (0.588, 1.636)	0.981 (0.567, 1.591)	0.581 (0.426, 0.823)	0.439 (0.240, 0.690)	0.390 (0.225, 0.626)	0.375 (0.217, 0.609)
Marche	1.886 (1.337, 3.057)	1.499 (0.940, 2.359)	1.327 (0.884, 2.017)	1.272 (0.835, 1.983)	0.649 (0.460, 1.052)	0.516 (0.323, 0.812)	0.457 (0.304, 0.694)	0.438 (0.288, 0.683)
Molise	1.933 (1.373, 3.124)	1.537 (0.960, 2.416)	1.361 (0.903, 2.069)	1.304 (0.861, 2.021)	0.655 (0.465, 1.058)	0.520 (0.325, 0.818)	0.461 (0.306, 0.701)	0.442 (0.292, 0.684)
Piemonte	2.513 (1.387, 4.705)	1.864 (0.986, 3.463)	1.646 (0.929, 3.106)	1.581 (0.845, 3.106)	0.848 (0.468, 1.588)	0.629 (0.333, 1.169)	0.556 (0.314, 1.049)	0.534 (0.285, 1.049)
Prov. autonoma di Bolzano	1.299 (0.659, 2.450)	0.995 (0.355, 1.893)	0.878 (0.327, 1.585)	0.841 (0.327, 1.415)	0.561 (0.285, 1.058)	0.430 (0.153, 0.818)	0.379 (0.141, 0.685)	0.363 (0.141, 0.611)
Prov. autonoma di Trento	1.683 (1.199, 2.914)	1.292 (0.664, 2.218)	1.141 (0.563, 1.818)	1.094 (0.563, 1.768)	0.657 (0.468, 1.138)	0.504 (0.259, 0.866)	0.446 (0.220, 0.710)	0.427 (0.220, 0.690)
Puglia	1.601 (1.135, 2.577)	1.273 (0.799, 1.992)	1.127 (0.749, 1.715)	1.080 (0.711, 1.673)	0.649 (0.460, 1.045)	0.516 (0.324, 0.808)	0.457 (0.304, 0.695)	0.438 (0.288, 0.679)
Sardegna	1.764 (1.249, 2.843)	1.402 (0.881, 2.198)	1.241 (0.826, 1.892)	1.189 (0.782, 1.852)	0.652 (0.462, 1.051)	0.518 (0.326, 0.813)	0.459 (0.305, 0.700)	0.440 (0.289, 0.685)
Sicilia	1.539 (1.090, 2.476)	1.224 (0.766, 1.914)	1.084 (0.718, 1.654)	1.038 (0.684, 1.610)	0.653 (0.462, 1.051)	0.519 (0.325, 0.812)	0.460 (0.305, 0.702)	0.441 (0.290, 0.683)
Toscana	1.936 (1.372, 3.144)	1.539 (0.963, 2.423)	1.363 (0.908, 2.071)	1.306 (0.857, 2.037)	0.650 (0.461, 1.056)	0.517 (0.324, 0.814)	0.458 (0.305, 0.696)	0.439 (0.288, 0.684)
Umbria	1.924 (1.363, 3.118)	1.529 (0.958, 2.408)	1.354 (0.902, 2.060)	1.297 (0.852, 2.022)	0.652 (0.462, 1.056)	0.518 (0.324, 0.816)	0.459 (0.305, 0.698)	0.439 (0.289, 0.685)
Valle d'Aosta	1.782 (1.236, 2.879)	1.398 (0.849, 2.321)	1.244 (0.666, 1.979)	1.195 (0.666, 1.943)	0.641 (0.445, 1.036)	0.503 (0.306, 0.835)	0.448 (0.240, 0.712)	0.430 (0.240, 0.699)
Veneto	1.725 (1.221, 2.803)	1.371 (0.857, 2.162)	1.214 (0.808, 1.845)	1.163 (0.763, 1.813)	0.656 (0.465, 1.067)	0.522 (0.326, 0.823)	0.462 (0.307, 0.702)	0.443 (0.290, 0.690)
Luxembourg	1.343 (0.955, 2.215)	1.052 (0.749, 1.710)	0.926 (0.666, 1.387)	0.886 (0.611, 1.291)	0.800 (0.569, 1.319)	0.627 (0.446, 1.019)	0.552 (0.397, 0.826)	0.528 (0.364, 0.769)
Malta	1.745 (1.236, 2.869)	1.367 (0.972, 2.234)	1.204 (0.870, 1.803)	1.151 (0.796, 1.666)	0.796 (0.564, 1.308)	0.623 (0.443, 1.019)	0.549 (0.397, 0.822)	0.525 (0.363, 0.760)
Monaco	2.270 (1.595, 3.744)	1.778 (1.265, 2.915)	1.566 (1.146, 2.368)	1.497 (1.027, 2.154)	0.809 (0.568, 1.334)	0.633 (0.451, 1.039)	0.558 (0.408, 0.844)	0.534 (0.366, 0.768)
Netherlands	1.417 (0.787, 2.492)	1.150 (0.596, 2.065)	1.027 (0.542, 1.713)	0.987 (0.497, 1.575)	0.692 (0.384, 1.217)	0.561 (0.291, 1.008)	0.502 (0.265, 0.836)	0.482 (0.243, 0.769)
Norway	0.753 (0.020, 2.280)	0.582 (0.015, 1.724)	0.504 (0.013, 1.401)	0.485 (0.013, 1.367)	0.405 (0.011, 1.227)	0.313 (0.008, 0.927)	0.271 (0.007, 0.754)	0.261 (0.007, 0.736)
Portugal	2.683 (1.083, 5.567)	2.085 (0.946, 4.395)	1.805 (0.901, 3.456)	1.708 (0.901, 3.269)	1.095 (0.442, 2.271)	0.850 (0.386, 1.793)	0.736 (0.368, 1.410)	0.697 (0.368, 1.334)
San Marino	1.668 (1.181, 2.752)	1.306 (0.930, 2.130)	1.150 (0.835, 1.730)	1.100 (0.762, 1.596)	0.802 (0.568, 1.323)	0.628 (0.447, 1.024)	0.553 (0.401, 0.832)	0.529 (0.366, 0.768)
Spain	2.175 (1.341, 3.454)	1.710 (0.991, 2.718)	1.512 (0.848, 2.298)	1.447 (0.817, 2.270)	0.955 (0.589, 1.516)	0.751 (0.435, 1.193)	0.664 (0.372, 1.009)	0.635 (0.358, 0.996)
Andalusia	2.001 (0.519, 7.067)	1.455 (0.337, 2.969)	1.309 (0.337, 2.619)	1.261 (0.337, 2.619)	0.879 (0.228, 3.102)	0.639 (0.148, 1.303)	0.574 (0.148, 1.150)	0.554 (0.148, 1.150)
Aragon	2.278 (1.347, 4.142)	1.908 (0.883, 3.739)	1.798 (0.772, 3.379)	1.752 (0.732, 3.288)	1.000 (0.591, 1.818)	0.838 (0.388, 1.642)	0.789 (0.339, 1.483)	0.769 (0.322, 1.443)
Asturias	4.613 (1.447, 11.731)	2.666 (1.069, 5.062)	2.187 (0.927, 4.017)	2.068 (0.898, 3.685)	2.025 (0.635, 5.150)	1.170 (0.469, 2.222)	0.960 (0.407, 1.763)	0.908 (0.394, 1.617)
Balearic Islands	2.342 (0.852, 5.492)	1.436 (0.590, 2.655)	1.223 (0.447, 2.268)	1.162 (0.400, 2.268)	1.028 (0.374, 2.411)	0.631 (0.259, 1.166)	0.537 (0.196, 0.996)	0.510 (0.176, 0.996)
Basque Country	2.962 (1.796, 5.157)	2.145 (1.299, 3.755)	1.913 (1.088, 3.121)	1.836 (1.064, 2.968)	1.300 (0.788, 2.264)	0.942 (0.570, 1.648)	0.840 (0.478, 1.370)	0.806 (0.467, 1.303)
Canary Islands	2.418 (0.750, 6.835)	1.457 (0.503, 2.557)	1.246 (0.390, 2.434)	1.185 (0.390, 2.394)	1.061 (0.329, 3.000)	0.640 (0.221, 1.123)	0.547 (0.171, 1.069)	0.520 (0.171, 1.051)
Cantabria	2.111 (0.863, 3.934)	1.590 (0.557, 3.161)	1.397 (0.420, 2.767)	1.334 (0.420, 2.695)	0.927 (0.379, 1.727)	0.698 (0.245, 1.388)	0.613 (0.184, 1.215)	0.586 (0.184, 1.183)
Castile and León	1.896 (1.150, 3.402)	1.611 (0.726, 3.158)	1.519 (0.707, 2.905)	1.486 (0.707, 2.830)	0.832 (0.505, 1.494)	0.707 (0.319, 1.386)	0.667 (0.310, 1.275)	0.652 (0.310, 1.242)
Castilla–La Mancha	2.044 (1.367, 3.107)	1.455 (0.802, 2.082)	1.280 (0.677, 2.044)	1.228 (0.631, 2.028)	0.897 (0.600, 1.364)	0.639 (0.352, 0.914)	0.562 (0.297, 0.897)	0.539 (0.277, 0.890)
Catalonia	3.292 (1.855, 4.970)	2.193 (1.064, 3.445)	1.888 (0.789, 3.301)	1.801 (0.747, 3.301)	1.445 (0.814, 2.181)	0.962 (0.467, 1.512)	0.829 (0.346, 1.449)	0.791 (0.328, 1.449)
Ceuta	2.794 (0.987, 7.490)	1.676 (0.762, 3.839)	1.374 (0.581, 2.706)	1.289 (0.581, 2.317)	1.227 (0.433, 3.288)	0.736 (0.334, 1.685)	0.603 (0.255, 1.188)	0.566 (0.255, 1.017)
Community of Madrid	1.756 (1.116, 3.080)	1.295 (0.762, 2.388)	1.134 (0.567, 2.151)	1.085 (0.567, 1.936)	0.771 (0.490, 1.352)	0.568 (0.335, 1.048)	0.498 (0.249, 0.944)	0.476 (0.249, 0.850)
Extremadura	2.389 (1.197, 4.742)	1.790 (0.764, 3.509)	1.567 (0.693, 2.757)	1.497 (0.686, 2.576)	1.048 (0.525, 2.081)	0.786 (0.335, 1.540)	0.688 (0.304, 1.210)	0.657 (0.301, 1.131)
Galicia	3.183 (1.366, 7.904)	1.852 (1.023, 3.046)	1.584 (0.776, 2.560)	1.506 (0.697, 2.560)	1.397 (0.600, 3.469)	0.813 (0.449, 1.337)	0.695 (0.341, 1.124)	0.661 (0.306, 1.124)
La Rioja	4.254 (2.383, 6.233)	3.051 (1.791, 5.133)	2.659 (1.577, 4.123)	2.540 (1.423, 4.048)	1.867 (1.046, 2.736)	1.339 (0.786, 2.253)	1.167 (0.692, 1.810)	1.115 (0.624, 1.777)
Melilla	1.047 (0.227, 4.665)	0.767 (0.141, 2.389)	0.691 (0.133, 1.965)	0.673 (0.133, 1.841)	0.460 (0.099, 2.048)	0.337 (0.062, 1.049)	0.303 (0.058, 0.863)	0.295 (0.058, 0.808)
Murcia	2.992 (0.822, 8.028)	1.652 (0.676, 3.380)	1.365 (0.619, 2.313)	1.291 (0.562, 2.286)	1.314 (0.361, 3.524)	0.725 (0.297, 1.484)	0.599 (0.272, 1.015)	0.567 (0.247, 1.003)
Navarre	2.093 (1.096, 3.696)	1.498 (0.746, 2.823)	1.294 (0.591, 2.220)	1.228 (0.573, 2.097)	0.919 (0.481, 1.622)	0.658 (0.328, 1.239)	0.568 (0.259, 0.975)	0.539 (0.252, 0.921)
Valencian Community	2.522 (1.139, 6.236)	1.530 (0.812, 2.718)	1.302 (0.670, 2.172)	1.237 (0.598, 2.112)	1.107 (0.500, 2.738)	0.672 (0.356, 1.193)	0.571 (0.294, 0.954)	0.543 (0.263, 0.927)
Sweden	1.310 (0.750, 2.493)	1.035 (0.651, 1.883)	0.916 (0.605, 1.461)	0.878 (0.565, 1.437)	0.606 (0.347, 1.152)	0.478 (0.301, 0.870)	0.423 (0.280, 0.675)	0.406 (0.261, 0.664)
Switzerland	1.687 (1.202, 2.782)	1.322 (0.943, 2.148)	1.164 (0.835, 1.743)	1.113 (0.766, 1.621)	0.796 (0.567, 1.312)	0.623 (0.445, 1.013)	0.549 (0.394, 0.822)	0.525 (0.361, 0.765)
UK	1.568 (1.222, 2.473)	1.339 (1.054, 2.155)	1.237 (0.953, 1.877)	1.201 (0.880, 1.727)	0.809 (0.631, 1.276)	0.691 (0.544, 1.112)	0.638 (0.492, 0.969)	0.620 (0.454, 0.891)
England	1.373 (1.054, 2.141)	1.258 (0.989, 2.013)	1.218 (0.965, 1.881)	1.205 (0.941, 1.826)	0.715 (0.549, 1.115)	0.656 (0.515, 1.049)	0.635 (0.503, 0.980)	0.628 (0.490, 0.951)
Northern Ireland	3.196 (1.018, 10.353)	2.224 (0.793, 4.922)	2.071 (0.705, 4.173)	2.026 (0.705, 4.173)	1.836 (0.585, 5.950)	1.278 (0.456, 2.829)	1.190 (0.405, 2.398)	1.164 (0.405, 2.398)
Scotland	1.901 (1.291, 3.211)	1.618 (1.035, 2.869)	1.547 (0.987, 2.688)	1.520 (0.905, 2.676)	0.924 (0.628, 1.562)	0.787 (0.503, 1.395)	0.752 (0.480, 1.307)	0.739 (0.440, 1.301)
Wales	2.210 (0.911, 4.924)	1.415 (0.764, 2.450)	1.249 (0.706, 2.029)	1.199 (0.634, 1.911)	1.014 (0.418, 2.258)	0.649 (0.350, 1.124)	0.573 (0.324, 0.931)	0.550 (0.291, 0.877)
**Latin America and Caribbean**								
**Andean Latin America**								
Bolivia	0.465 (0.309, 0.845)	0.369 (0.260, 0.621)	0.326 (0.229, 0.527)	0.312 (0.208, 0.511)	0.715 (0.475, 1.300)	0.568 (0.400, 0.956)	0.501 (0.353, 0.810)	0.480 (0.320, 0.785)
Ecuador	0.573 (0.377, 1.031)	0.455 (0.323, 0.759)	0.401 (0.284, 0.652)	0.384 (0.253, 0.624)	0.725 (0.477, 1.305)	0.576 (0.409, 0.961)	0.508 (0.359, 0.825)	0.486 (0.321, 0.790)
Peru	1.065 (0.669, 1.849)	0.837 (0.585, 1.412)	0.740 (0.482, 1.249)	0.708 (0.439, 1.175)	1.159 (0.728, 2.013)	0.911 (0.636, 1.538)	0.806 (0.525, 1.360)	0.771 (0.478, 1.279)
**Caribbean**								
Antigua and Barbuda	0.584 (0.318, 1.062)	0.461 (0.279, 0.856)	0.403 (0.250, 0.688)	0.384 (0.238, 0.628)	0.550 (0.299, 1.000)	0.434 (0.262, 0.806)	0.379 (0.235, 0.648)	0.362 (0.224, 0.591)
The Bahamas	0.522 (0.289, 0.959)	0.412 (0.248, 0.773)	0.360 (0.225, 0.622)	0.343 (0.213, 0.558)	0.554 (0.306, 1.018)	0.437 (0.263, 0.820)	0.382 (0.239, 0.659)	0.364 (0.226, 0.592)
Barbados	0.910 (0.504, 1.684)	0.718 (0.431, 1.358)	0.628 (0.390, 1.091)	0.599 (0.367, 0.971)	0.553 (0.306, 1.023)	0.436 (0.262, 0.825)	0.381 (0.237, 0.663)	0.364 (0.223, 0.590)
Belize	0.340 (0.188, 0.623)	0.268 (0.162, 0.502)	0.234 (0.147, 0.404)	0.223 (0.139, 0.363)	0.557 (0.309, 1.021)	0.439 (0.265, 0.823)	0.384 (0.241, 0.661)	0.366 (0.228, 0.594)
Bermuda	1.181 (0.652, 2.217)	0.931 (0.545, 1.787)	0.814 (0.498, 1.436)	0.776 (0.464, 1.262)	0.554 (0.306, 1.040)	0.437 (0.256, 0.839)	0.382 (0.234, 0.674)	0.364 (0.218, 0.592)
Cuba	0.980 (0.541, 1.825)	0.773 (0.459, 1.471)	0.676 (0.417, 1.182)	0.645 (0.390, 1.047)	0.567 (0.313, 1.056)	0.447 (0.266, 0.851)	0.391 (0.241, 0.684)	0.373 (0.226, 0.606)
Dominica	0.721 (0.401, 1.323)	0.568 (0.345, 1.067)	0.497 (0.314, 0.857)	0.474 (0.296, 0.769)	0.558 (0.310, 1.024)	0.440 (0.267, 0.825)	0.385 (0.243, 0.663)	0.367 (0.229, 0.595)
Dominican Republic	0.437 (0.240, 0.795)	0.345 (0.209, 0.641)	0.301 (0.189, 0.515)	0.287 (0.179, 0.468)	0.552 (0.304, 1.005)	0.435 (0.264, 0.810)	0.381 (0.238, 0.651)	0.363 (0.226, 0.592)
Grenada	0.539 (0.294, 0.975)	0.425 (0.259, 0.786)	0.372 (0.233, 0.631)	0.355 (0.221, 0.580)	0.553 (0.301, 1.000)	0.436 (0.265, 0.806)	0.382 (0.239, 0.647)	0.364 (0.226, 0.595)
Guyana	0.389 (0.215, 0.706)	0.307 (0.188, 0.569)	0.269 (0.170, 0.457)	0.256 (0.161, 0.417)	0.557 (0.307, 1.009)	0.439 (0.269, 0.813)	0.384 (0.243, 0.654)	0.366 (0.230, 0.596)
Haiti	0.262 (0.143, 0.459)	0.207 (0.130, 0.367)	0.181 (0.117, 0.295)	0.172 (0.106, 0.284)	0.547 (0.299, 0.959)	0.431 (0.272, 0.766)	0.377 (0.245, 0.616)	0.360 (0.222, 0.592)
Jamaica	0.603 (0.332, 1.105)	0.476 (0.287, 0.891)	0.416 (0.259, 0.716)	0.397 (0.246, 0.646)	0.560 (0.308, 1.025)	0.441 (0.266, 0.826)	0.386 (0.241, 0.664)	0.368 (0.228, 0.599)
Puerto Rico	1.189 (0.613, 2.443)	0.929 (0.460, 1.963)	0.812 (0.436, 1.552)	0.773 (0.422, 1.365)	0.545 (0.281, 1.119)	0.426 (0.211, 0.899)	0.372 (0.200, 0.711)	0.354 (0.193, 0.626)
Saint Kitts and Nevis	0.532 (0.292, 0.977)	0.419 (0.252, 0.787)	0.367 (0.227, 0.633)	0.350 (0.215, 0.569)	0.548 (0.301, 1.007)	0.432 (0.260, 0.812)	0.378 (0.235, 0.652)	0.360 (0.222, 0.587)
Saint Lucia	0.657 (0.360, 1.198)	0.518 (0.313, 0.966)	0.453 (0.283, 0.776)	0.432 (0.268, 0.703)	0.555 (0.304, 1.012)	0.437 (0.264, 0.816)	0.383 (0.239, 0.656)	0.365 (0.227, 0.594)
Saint Vincent and the Grenadines	0.627 (0.345, 1.141)	0.494 (0.300, 0.919)	0.432 (0.272, 0.739)	0.412 (0.257, 0.671)	0.561 (0.309, 1.021)	0.442 (0.269, 0.823)	0.387 (0.243, 0.662)	0.369 (0.230, 0.601)
Suriname	0.545 (0.302, 0.996)	0.430 (0.261, 0.802)	0.376 (0.238, 0.645)	0.359 (0.225, 0.583)	0.564 (0.313, 1.031)	0.445 (0.271, 0.831)	0.389 (0.246, 0.668)	0.371 (0.233, 0.604)
Trinidad and Tobago	0.721 (0.403, 1.336)	0.568 (0.342, 1.076)	0.497 (0.314, 0.865)	0.474 (0.294, 0.768)	0.563 (0.315, 1.044)	0.444 (0.267, 0.841)	0.389 (0.245, 0.676)	0.371 (0.229, 0.600)
Virgin Islands	0.954 (0.539, 1.828)	0.752 (0.445, 1.473)	0.658 (0.417, 1.158)	0.627 (0.380, 1.022)	0.560 (0.316, 1.073)	0.442 (0.261, 0.865)	0.386 (0.245, 0.680)	0.368 (0.223, 0.600)
**Central Latin America**								
Colombia	0.777 (0.452, 1.483)	0.616 (0.415, 1.149)	0.542 (0.353, 0.928)	0.517 (0.328, 0.873)	0.707 (0.411, 1.349)	0.560 (0.378, 1.045)	0.493 (0.321, 0.844)	0.471 (0.298, 0.794)
Costa Rica	0.757 (0.440, 1.449)	0.600 (0.402, 1.121)	0.528 (0.345, 0.899)	0.504 (0.321, 0.855)	0.713 (0.414, 1.365)	0.565 (0.379, 1.056)	0.497 (0.325, 0.847)	0.475 (0.303, 0.805)
El Salvador	0.666 (0.389, 1.270)	0.528 (0.356, 0.983)	0.464 (0.303, 0.794)	0.443 (0.282, 0.749)	0.703 (0.410, 1.339)	0.556 (0.375, 1.037)	0.489 (0.320, 0.837)	0.468 (0.297, 0.789)
Guatemala	0.400 (0.233, 0.760)	0.317 (0.215, 0.590)	0.279 (0.183, 0.480)	0.266 (0.167, 0.443)	0.702 (0.409, 1.333)	0.556 (0.378, 1.034)	0.489 (0.322, 0.841)	0.467 (0.293, 0.778)
Honduras	0.369 (0.145, 0.787)	0.291 (0.110, 0.627)	0.255 (0.100, 0.495)	0.244 (0.100, 0.433)	0.679 (0.266, 1.447)	0.536 (0.203, 1.152)	0.469 (0.184, 0.909)	0.448 (0.184, 0.796)
Mexico	0.796 (0.413, 1.528)	0.626 (0.372, 1.226)	0.549 (0.349, 0.981)	0.523 (0.340, 0.888)	0.912 (0.473, 1.751)	0.717 (0.426, 1.404)	0.629 (0.400, 1.123)	0.600 (0.389, 1.017)
Aguascalientes	1.306 (0.417, 3.851)	0.751 (0.372, 1.964)	0.635 (0.320, 1.448)	0.602 (0.302, 1.448)	1.734 (0.554, 5.115)	0.998 (0.494, 2.608)	0.844 (0.425, 1.923)	0.799 (0.402, 1.923)
Baja California	0.725 (0.294, 1.692)	0.401 (0.226, 1.001)	0.338 (0.178, 0.796)	0.320 (0.150, 0.697)	1.007 (0.408, 2.348)	0.556 (0.314, 1.389)	0.469 (0.246, 1.105)	0.444 (0.208, 0.968)
Baja California Sur	0.828 (0.317, 2.143)	0.479 (0.289, 0.957)	0.405 (0.234, 0.756)	0.384 (0.180, 0.700)	1.214 (0.465, 3.142)	0.702 (0.424, 1.403)	0.594 (0.343, 1.108)	0.563 (0.264, 1.027)
Campeche	1.099 (0.432, 2.441)	0.623 (0.375, 1.111)	0.527 (0.278, 0.917)	0.500 (0.265, 0.862)	1.332 (0.524, 2.958)	0.755 (0.454, 1.347)	0.639 (0.337, 1.111)	0.607 (0.322, 1.045)
Chiapas	0.828 (0.311, 2.151)	0.510 (0.215, 1.118)	0.433 (0.180, 0.890)	0.411 (0.163, 0.875)	1.242 (0.466, 3.224)	0.764 (0.323, 1.675)	0.649 (0.269, 1.334)	0.616 (0.244, 1.312)
Chihuahua	2.259 (0.452, 6.101)	1.368 (0.396, 3.755)	1.116 (0.347, 2.715)	1.052 (0.338, 2.626)	2.750 (0.550, 7.427)	1.666 (0.482, 4.571)	1.359 (0.422, 3.305)	1.281 (0.412, 3.196)
Coahuila	0.862 (0.279, 1.867)	0.479 (0.245, 0.925)	0.391 (0.233, 0.744)	0.369 (0.194, 0.711)	1.041 (0.337, 2.254)	0.578 (0.296, 1.117)	0.472 (0.281, 0.899)	0.446 (0.235, 0.858)
Colima	1.400 (0.452, 3.284)	0.785 (0.388, 1.341)	0.647 (0.368, 1.007)	0.613 (0.348, 0.927)	1.628 (0.526, 3.820)	0.913 (0.451, 1.559)	0.753 (0.428, 1.171)	0.713 (0.405, 1.078)

Durango	0.641 (0.207, 1.579)	0.408 (0.177, 1.225)	0.352 (0.119, 0.969)	0.335 (0.102, 0.886)	0.705 (0.228, 1.737)	0.449 (0.195, 1.347)	0.387 (0.131, 1.066)	0.369 (0.113, 0.974)
Guanajuato	0.687 (0.206, 1.468)	0.407 (0.159, 0.850)	0.338 (0.129, 0.827)	0.321 (0.109, 0.824)	0.839 (0.252, 1.794)	0.498 (0.195, 1.039)	0.414 (0.158, 1.011)	0.392 (0.134, 1.007)
Guerrero	1.027 (0.429, 2.277)	0.588 (0.380, 1.186)	0.497 (0.286, 0.952)	0.471 (0.248, 0.839)	1.194 (0.498, 2.647)	0.684 (0.441, 1.379)	0.578 (0.332, 1.106)	0.548 (0.288, 0.975)
Hidalgo	0.767 (0.275, 1.788)	0.431 (0.219, 1.018)	0.365 (0.150, 0.964)	0.346 (0.119, 0.917)	0.818 (0.294, 1.908)	0.460 (0.234, 1.086)	0.389 (0.160, 1.029)	0.369 (0.127, 0.979)
Jalisco	0.990 (0.396, 2.520)	0.554 (0.313, 1.167)	0.460 (0.264, 0.914)	0.435 (0.215, 0.860)	1.145 (0.458, 2.914)	0.640 (0.362, 1.349)	0.532 (0.306, 1.056)	0.503 (0.249, 0.994)
México	1.028 (0.404, 2.605)	0.600 (0.369, 1.081)	0.512 (0.319, 0.881)	0.485 (0.288, 0.804)	1.337 (0.525, 3.387)	0.780 (0.480, 1.406)	0.665 (0.414, 1.146)	0.631 (0.374, 1.045)
Mexico City	1.220 (0.548, 2.799)	0.768 (0.470, 1.446)	0.658 (0.352, 1.227)	0.626 (0.314, 1.092)	1.052 (0.473, 2.413)	0.662 (0.405, 1.247)	0.567 (0.304, 1.058)	0.539 (0.271, 0.941)
Michoacán de Ocampo	1.108 (0.362, 2.363)	0.595 (0.338, 1.095)	0.487 (0.265, 0.869)	0.458 (0.200, 0.869)	1.121 (0.366, 2.390)	0.602 (0.342, 1.107)	0.493 (0.268, 0.878)	0.463 (0.202, 0.878)
Morelos	0.825 (0.317, 1.736)	0.500 (0.249, 1.069)	0.426 (0.186, 0.907)	0.405 (0.159, 0.900)	0.857 (0.330, 1.804)	0.519 (0.258, 1.111)	0.442 (0.193, 0.943)	0.421 (0.165, 0.935)
Nayarit	1.081 (0.374, 2.676)	0.575 (0.311, 1.286)	0.480 (0.224, 1.049)	0.454 (0.175, 0.970)	1.125 (0.390, 2.785)	0.599 (0.323, 1.339)	0.500 (0.234, 1.092)	0.472 (0.182, 1.009)
Nuevo León	1.053 (0.372, 2.306)	0.563 (0.318, 1.070)	0.455 (0.284, 0.912)	0.429 (0.216, 0.852)	1.208 (0.427, 2.645)	0.646 (0.365, 1.226)	0.521 (0.325, 1.046)	0.492 (0.247, 0.978)
Oaxaca	1.046 (0.390, 2.423)	0.641 (0.359, 1.294)	0.542 (0.243, 1.069)	0.512 (0.215, 0.976)	1.031 (0.384, 2.388)	0.632 (0.354, 1.275)	0.534 (0.240, 1.054)	0.505 (0.212, 0.962)
Puebla	0.928 (0.392, 2.051)	0.545 (0.288, 0.975)	0.455 (0.255, 0.749)	0.431 (0.200, 0.705)	1.115 (0.470, 2.465)	0.654 (0.346, 1.172)	0.547 (0.307, 0.900)	0.518 (0.240, 0.847)
Querétaro	1.393 (0.365, 3.238)	0.851 (0.335, 2.005)	0.715 (0.320, 1.776)	0.677 (0.304, 1.748)	1.859 (0.488, 4.321)	1.136 (0.448, 2.676)	0.954 (0.427, 2.370)	0.903 (0.405, 2.333)
Quintana Roo	0.617 (0.230, 1.542)	0.352 (0.194, 0.651)	0.299 (0.153, 0.540)	0.284 (0.133, 0.474)	1.104 (0.411, 2.759)	0.631 (0.347, 1.165)	0.536 (0.273, 0.967)	0.508 (0.237, 0.849)
San Luis Potosí	1.222 (0.383, 3.138)	0.748 (0.346, 1.516)	0.622 (0.324, 1.086)	0.586 (0.310, 0.982)	1.273 (0.399, 3.269)	0.779 (0.360, 1.580)	0.649 (0.338, 1.132)	0.611 (0.323, 1.023)
Sinaloa	0.933 (0.385, 1.831)	0.562 (0.317, 1.130)	0.472 (0.226, 0.926)	0.447 (0.183, 0.913)	0.941 (0.388, 1.845)	0.567 (0.320, 1.139)	0.476 (0.228, 0.933)	0.450 (0.184, 0.919)
Sonora	0.922 (0.398, 2.127)	0.550 (0.336, 1.210)	0.468 (0.242, 0.968)	0.445 (0.192, 0.858)	1.078 (0.465, 2.486)	0.643 (0.393, 1.414)	0.547 (0.283, 1.131)	0.520 (0.224, 1.003)
Tabasco	0.799 (0.294, 1.979)	0.448 (0.240, 0.965)	0.381 (0.176, 0.728)	0.361 (0.154, 0.706)	1.019 (0.375, 2.523)	0.571 (0.306, 1.230)	0.485 (0.225, 0.928)	0.461 (0.197, 0.900)
Tamaulipas	1.070 (0.324, 2.782)	0.607 (0.299, 1.189)	0.509 (0.266, 0.922)	0.481 (0.232, 0.888)	1.185 (0.359, 3.080)	0.672 (0.332, 1.317)	0.563 (0.294, 1.021)	0.532 (0.257, 0.983)
Tlaxcala	1.127 (0.326, 2.859)	0.635 (0.252, 1.206)	0.534 (0.223, 0.954)	0.506 (0.223, 0.889)	1.368 (0.396, 3.470)	0.771 (0.306, 1.464)	0.649 (0.270, 1.158)	0.614 (0.270, 1.079)
Veracruz de Ignacio de la Llave	0.960 (0.329, 2.288)	0.565 (0.273, 1.355)	0.478 (0.244, 1.090)	0.454 (0.198, 1.019)	0.943 (0.323, 2.247)	0.555 (0.268, 1.330)	0.470 (0.240, 1.070)	0.446 (0.195, 1.001)
Yucatán	0.777 (0.296, 1.697)	0.470 (0.219, 0.825)	0.396 (0.165, 0.715)	0.376 (0.135, 0.715)	0.851 (0.324, 1.858)	0.514 (0.240, 0.904)	0.434 (0.180, 0.783)	0.412 (0.148, 0.783)
Zacatecas	1.342 (0.419, 3.614)	0.782 (0.385, 1.716)	0.633 (0.371, 1.092)	0.595 (0.289, 1.023)	1.392 (0.435, 3.751)	0.812 (0.400, 1.781)	0.657 (0.385, 1.134)	0.618 (0.300, 1.062)
Nicaragua	0.424 (0.247, 0.808)	0.336 (0.227, 0.627)	0.296 (0.193, 0.508)	0.282 (0.178, 0.474)	0.704 (0.409, 1.341)	0.558 (0.377, 1.039)	0.490 (0.321, 0.842)	0.469 (0.295, 0.786)
Panama	0.681 (0.396, 1.296)	0.539 (0.365, 1.004)	0.474 (0.310, 0.814)	0.453 (0.285, 0.760)	0.699 (0.406, 1.331)	0.554 (0.375, 1.031)	0.487 (0.318, 0.836)	0.465 (0.293, 0.780)
Venezuela	0.692 (0.403, 1.320)	0.548 (0.370, 1.022)	0.482 (0.315, 0.826)	0.461 (0.292, 0.776)	0.709 (0.413, 1.353)	0.562 (0.379, 1.048)	0.494 (0.322, 0.847)	0.472 (0.299, 0.796)
**Tropical Latin America**								
Brazil	0.442 (0.344, 0.603)	0.394 (0.318, 0.523)	0.372 (0.297, 0.484)	0.363 (0.272, 0.463)	0.425 (0.331, 0.580)	0.380 (0.306, 0.503)	0.358 (0.286, 0.466)	0.350 (0.262, 0.446)
Acre	0.257 (0.200, 0.350)	0.230 (0.185, 0.304)	0.217 (0.172, 0.282)	0.212 (0.159, 0.269)	0.426 (0.331, 0.581)	0.381 (0.306, 0.504)	0.359 (0.286, 0.467)	0.351 (0.263, 0.446)
Alagoas	0.350 (0.271, 0.478)	0.313 (0.251, 0.413)	0.295 (0.234, 0.384)	0.288 (0.216, 0.365)	0.425 (0.329, 0.580)	0.379 (0.305, 0.501)	0.358 (0.284, 0.466)	0.349 (0.262, 0.443)
Amapá	0.237 (0.185, 0.323)	0.212 (0.171, 0.281)	0.200 (0.160, 0.260)	0.195 (0.146, 0.249)	0.424 (0.330, 0.578)	0.379 (0.306, 0.503)	0.357 (0.285, 0.465)	0.349 (0.261, 0.445)
Amazonas	0.371 (0.209, 0.963)	0.226 (0.150, 0.358)	0.203 (0.115, 0.304)	0.196 (0.113, 0.295)	0.585 (0.330, 1.519)	0.356 (0.236, 0.566)	0.321 (0.182, 0.481)	0.310 (0.179, 0.466)
Bahia	0.416 (0.325, 0.570)	0.372 (0.299, 0.491)	0.351 (0.279, 0.458)	0.343 (0.258, 0.434)	0.421 (0.328, 0.576)	0.376 (0.302, 0.497)	0.355 (0.282, 0.462)	0.346 (0.261, 0.439)
Ceará	0.403 (0.314, 0.551)	0.360 (0.290, 0.476)	0.339 (0.271, 0.441)	0.331 (0.249, 0.421)	0.422 (0.329, 0.577)	0.377 (0.304, 0.499)	0.355 (0.284, 0.462)	0.347 (0.260, 0.441)
Distrito Federal	0.337 (0.257, 0.471)	0.301 (0.237, 0.405)	0.283 (0.226, 0.374)	0.277 (0.203, 0.363)	0.431 (0.330, 0.603)	0.385 (0.304, 0.519)	0.363 (0.289, 0.479)	0.355 (0.260, 0.464)
Espírito Santo	0.615 (0.358, 1.364)	0.355 (0.187, 0.499)	0.320 (0.165, 0.465)	0.310 (0.165, 0.465)	0.593 (0.345, 1.314)	0.342 (0.180, 0.480)	0.309 (0.159, 0.448)	0.298 (0.159, 0.448)
Goiás	0.401 (0.312, 0.548)	0.358 (0.289, 0.474)	0.337 (0.269, 0.439)	0.330 (0.247, 0.420)	0.423 (0.330, 0.579)	0.378 (0.305, 0.501)	0.356 (0.285, 0.464)	0.348 (0.261, 0.443)
Maranhão	0.297 (0.103, 0.837)	0.172 (0.080, 0.342)	0.156 (0.048, 0.301)	0.151 (0.046, 0.293)	0.401 (0.139, 1.130)	0.233 (0.108, 0.461)	0.211 (0.065, 0.406)	0.204 (0.062, 0.396)
Mato Grosso	0.360 (0.279, 0.490)	0.321 (0.259, 0.426)	0.303 (0.242, 0.395)	0.296 (0.221, 0.378)	0.425 (0.330, 0.579)	0.379 (0.305, 0.504)	0.358 (0.286, 0.466)	0.350 (0.261, 0.446)
Mato Grosso do Sul	0.415 (0.321, 0.565)	0.371 (0.298, 0.492)	0.350 (0.279, 0.455)	0.342 (0.255, 0.436)	0.428 (0.331, 0.582)	0.382 (0.307, 0.507)	0.360 (0.288, 0.469)	0.352 (0.263, 0.450)
Minas Gerais	0.507 (0.395, 0.693)	0.453 (0.365, 0.599)	0.427 (0.341, 0.555)	0.417 (0.313, 0.530)	0.424 (0.330, 0.580)	0.379 (0.305, 0.501)	0.357 (0.285, 0.465)	0.349 (0.262, 0.444)
Pará	0.298 (0.232, 0.407)	0.266 (0.214, 0.352)	0.251 (0.200, 0.327)	0.245 (0.184, 0.311)	0.423 (0.329, 0.577)	0.377 (0.304, 0.499)	0.356 (0.283, 0.464)	0.348 (0.261, 0.442)
Paraíba	0.456 (0.356, 0.624)	0.407 (0.328, 0.538)	0.384 (0.306, 0.500)	0.375 (0.282, 0.476)	0.422 (0.329, 0.578)	0.377 (0.303, 0.498)	0.355 (0.283, 0.463)	0.347 (0.261, 0.441)
Paraná	0.473 (0.367, 0.644)	0.422 (0.340, 0.560)	0.398 (0.318, 0.518)	0.389 (0.291, 0.497)	0.428 (0.332, 0.583)	0.382 (0.307, 0.507)	0.360 (0.288, 0.469)	0.352 (0.263, 0.450)
Pernambuco	0.396 (0.308, 0.541)	0.354 (0.284, 0.468)	0.333 (0.265, 0.435)	0.326 (0.245, 0.414)	0.424 (0.330, 0.579)	0.378 (0.304, 0.501)	0.357 (0.284, 0.465)	0.349 (0.262, 0.443)
Piauí	0.420 (0.328, 0.576)	0.375 (0.301, 0.496)	0.354 (0.281, 0.462)	0.345 (0.260, 0.437)	0.420 (0.328, 0.576)	0.375 (0.301, 0.496)	0.354 (0.281, 0.462)	0.345 (0.260, 0.437)
Rio de Janeiro	0.525 (0.407, 0.716)	0.469 (0.377, 0.623)	0.442 (0.354, 0.577)	0.432 (0.323, 0.552)	0.426 (0.330, 0.580)	0.380 (0.306, 0.505)	0.359 (0.287, 0.468)	0.350 (0.262, 0.448)
Rio Grande do Norte	0.434 (0.338, 0.594)	0.388 (0.313, 0.513)	0.366 (0.292, 0.475)	0.357 (0.268, 0.454)	0.424 (0.329, 0.579)	0.378 (0.305, 0.501)	0.357 (0.285, 0.463)	0.349 (0.261, 0.443)
Rio Grande do Sul	1.875 (0.542, 4.317)	0.998 (0.484, 2.066)	0.801 (0.433, 1.164)	0.748 (0.426, 0.907)	1.385 (0.401, 3.188)	0.737 (0.357, 1.526)	0.592 (0.320, 0.860)	0.552 (0.315, 0.670)
Rondônia	0.339 (0.263, 0.462)	0.303 (0.244, 0.402)	0.285 (0.228, 0.372)	0.279 (0.209, 0.356)	0.425 (0.330, 0.579)	0.380 (0.306, 0.504)	0.358 (0.286, 0.466)	0.350 (0.262, 0.446)
Roraima	0.235 (0.183, 0.321)	0.210 (0.169, 0.278)	0.198 (0.158, 0.258)	0.194 (0.145, 0.246)	0.424 (0.329, 0.579)	0.379 (0.305, 0.502)	0.357 (0.285, 0.464)	0.349 (0.261, 0.444)
Santa Catarina	0.455 (0.354, 0.621)	0.406 (0.327, 0.539)	0.383 (0.307, 0.499)	0.374 (0.280, 0.478)	0.425 (0.331, 0.581)	0.380 (0.306, 0.503)	0.358 (0.287, 0.466)	0.350 (0.262, 0.446)
São Paulo	0.495 (0.324, 1.202)	0.412 (0.309, 0.583)	0.408 (0.295, 0.555)	0.406 (0.292, 0.547)	0.434 (0.284, 1.052)	0.361 (0.271, 0.510)	0.357 (0.258, 0.486)	0.356 (0.256, 0.479)
Sergipe	1.227 (0.455, 2.719)	0.669 (0.429, 0.961)	0.580 (0.344, 0.714)	0.557 (0.313, 0.714)	1.385 (0.514, 3.069)	0.756 (0.484, 1.085)	0.655 (0.388, 0.806)	0.629 (0.353, 0.806)
Tocantins	0.342 (0.268, 0.469)	0.306 (0.246, 0.404)	0.288 (0.230, 0.376)	0.282 (0.212, 0.358)	0.421 (0.329, 0.577)	0.376 (0.303, 0.497)	0.355 (0.282, 0.463)	0.346 (0.261, 0.440)
Paraguay	0.378 (0.271, 0.575)	0.299 (0.236, 0.442)	0.264 (0.199, 0.354)	0.253 (0.179, 0.329)	0.505 (0.362, 0.769)	0.400 (0.315, 0.592)	0.353 (0.266, 0.473)	0.339 (0.240, 0.440)
**North Africa and Middle East**								
**North Africa and Middle East**								
Afghanistan	0.170 (0.057, 0.374)	0.133 (0.049, 0.298)	0.116 (0.042, 0.255)	0.111 (0.041, 0.244)	0.603 (0.204, 1.328)	0.473 (0.173, 1.059)	0.413 (0.151, 0.905)	0.393 (0.144, 0.866)
Algeria	0.372 (0.151, 0.739)	0.294 (0.132, 0.595)	0.257 (0.123, 0.475)	0.245 (0.121, 0.406)	0.524 (0.212, 1.040)	0.413 (0.186, 0.838)	0.362 (0.173, 0.668)	0.344 (0.171, 0.572)
Bahrain	0.336 (0.136, 0.660)	0.265 (0.116, 0.532)	0.232 (0.112, 0.423)	0.221 (0.109, 0.370)	0.525 (0.213, 1.032)	0.414 (0.182, 0.832)	0.363 (0.175, 0.662)	0.345 (0.171, 0.579)
Egypt	0.203 (0.073, 0.461)	0.159 (0.058, 0.390)	0.140 (0.051, 0.303)	0.134 (0.050, 0.283)	0.380 (0.137, 0.861)	0.298 (0.109, 0.728)	0.262 (0.096, 0.566)	0.251 (0.093, 0.528)
Iran	0.506 (0.203, 0.965)	0.397 (0.173, 0.728)	0.347 (0.164, 0.615)	0.331 (0.155, 0.594)	0.632 (0.254, 1.207)	0.496 (0.216, 0.911)	0.434 (0.205, 0.769)	0.414 (0.194, 0.744)
Iraq	0.248 (0.101, 0.491)	0.195 (0.088, 0.395)	0.171 (0.082, 0.315)	0.163 (0.081, 0.270)	0.524 (0.213, 1.038)	0.413 (0.187, 0.837)	0.362 (0.174, 0.668)	0.344 (0.172, 0.571)
Jordan	0.285 (0.099, 0.624)	0.222 (0.088, 0.499)	0.191 (0.086, 0.395)	0.181 (0.086, 0.348)	0.582 (0.202, 1.276)	0.454 (0.180, 1.021)	0.391 (0.175, 0.807)	0.370 (0.175, 0.712)
Kuwait	0.311 (0.124, 0.607)	0.245 (0.106, 0.489)	0.215 (0.102, 0.389)	0.204 (0.102, 0.346)	0.534 (0.214, 1.042)	0.421 (0.182, 0.840)	0.369 (0.175, 0.668)	0.351 (0.174, 0.595)
Lebanon	0.519 (0.210, 1.026)	0.409 (0.179, 0.827)	0.358 (0.171, 0.658)	0.341 (0.167, 0.572)	0.537 (0.217, 1.062)	0.424 (0.186, 0.856)	0.371 (0.177, 0.681)	0.353 (0.173, 0.592)
Libya	0.370 (0.151, 0.727)	0.292 (0.131, 0.586)	0.255 (0.124, 0.467)	0.243 (0.121, 0.406)	0.526 (0.214, 1.034)	0.415 (0.186, 0.833)	0.363 (0.176, 0.664)	0.345 (0.172, 0.577)
Morocco	0.392 (0.159, 0.776)	0.310 (0.140, 0.626)	0.271 (0.131, 0.499)	0.258 (0.129, 0.427)	0.517 (0.210, 1.023)	0.408 (0.185, 0.825)	0.357 (0.172, 0.658)	0.340 (0.170, 0.563)
Oman	0.363 (0.162, 0.675)	0.284 (0.142, 0.522)	0.249 (0.128, 0.428)	0.237 (0.124, 0.375)	0.973 (0.433, 1.809)	0.762 (0.381, 1.399)	0.666 (0.342, 1.146)	0.636 (0.332, 1.006)
Palestine	0.216 (0.087, 0.430)	0.170 (0.077, 0.347)	0.149 (0.071, 0.277)	0.142 (0.071, 0.235)	0.524 (0.212, 1.043)	0.413 (0.187, 0.841)	0.362 (0.172, 0.671)	0.344 (0.172, 0.569)
Qatar	0.077 (0.023, 0.320)	0.061 (0.018, 0.274)	0.054 (0.015, 0.238)	0.052 (0.015, 0.204)	0.208 (0.063, 0.860)	0.165 (0.050, 0.737)	0.146 (0.041, 0.640)	0.139 (0.041, 0.549)
Saudi Arabia	0.241 (0.097, 0.471)	0.190 (0.083, 0.380)	0.166 (0.080, 0.302)	0.158 (0.079, 0.266)	0.523 (0.211, 1.024)	0.413 (0.181, 0.826)	0.361 (0.174, 0.657)	0.344 (0.171, 0.579)
Sudan	0.209 (0.085, 0.413)	0.165 (0.075, 0.333)	0.144 (0.070, 0.266)	0.137 (0.070, 0.227)	0.518 (0.211, 1.022)	0.408 (0.186, 0.824)	0.357 (0.174, 0.658)	0.340 (0.172, 0.562)
Syria	0.379 (0.154, 0.752)	0.299 (0.135, 0.606)	0.262 (0.125, 0.483)	0.249 (0.124, 0.413)	0.525 (0.213, 1.042)	0.414 (0.187, 0.839)	0.363 (0.173, 0.669)	0.345 (0.172, 0.572)
Tunisia	0.528 (0.214, 1.047)	0.417 (0.185, 0.843)	0.365 (0.174, 0.672)	0.347 (0.170, 0.579)	0.526 (0.213, 1.041)	0.415 (0.184, 0.839)	0.363 (0.173, 0.668)	0.345 (0.170, 0.576)
Turkey	0.548 (0.222, 1.087)	0.432 (0.192, 0.877)	0.379 (0.181, 0.697)	0.360 (0.177, 0.601)	0.533 (0.216, 1.056)	0.420 (0.187, 0.853)	0.368 (0.176, 0.678)	0.350 (0.172, 0.585)
United Arab Emirates	0.246 (0.100, 0.478)	0.194 (0.086, 0.386)	0.170 (0.082, 0.307)	0.161 (0.082, 0.272)	0.526 (0.213, 1.024)	0.415 (0.184, 0.826)	0.363 (0.176, 0.658)	0.346 (0.175, 0.582)
Yemen	0.143 (0.045, 0.312)	0.113 (0.038, 0.260)	0.100 (0.034, 0.212)	0.095 (0.030, 0.192)	0.393 (0.125, 0.856)	0.310 (0.104, 0.715)	0.274 (0.094, 0.582)	0.262 (0.083, 0.528)
**South Asia**								
**South Asia**								
Bangladesh	0.320 (0.132, 0.646)	0.251 (0.114, 0.515)	0.218 (0.099, 0.409)	0.207 (0.097, 0.357)	0.436 (0.180, 0.880)	0.342 (0.156, 0.702)	0.297 (0.134, 0.557)	0.282 (0.132, 0.487)
Bhutan	0.285 (0.117, 0.574)	0.223 (0.101, 0.460)	0.194 (0.088, 0.365)	0.184 (0.086, 0.319)	0.426 (0.175, 0.859)	0.334 (0.151, 0.688)	0.290 (0.132, 0.546)	0.276 (0.129, 0.476)
India	0.524 (0.206, 1.027)	0.407 (0.175, 0.779)	0.354 (0.151, 0.614)	0.337 (0.148, 0.571)	0.733 (0.288, 1.437)	0.569 (0.246, 1.091)	0.495 (0.212, 0.859)	0.471 (0.207, 0.799)
Andhra Pradesh	0.738 (0.136, 1.660)	0.304 (0.107, 0.729)	0.214 (0.091, 0.456)	0.193 (0.078, 0.386)	0.797 (0.147, 1.791)	0.328 (0.116, 0.787)	0.231 (0.098, 0.492)	0.208 (0.084, 0.416)
Arunachal Pradesh	0.332 (0.130, 0.651)	0.257 (0.111, 0.493)	0.224 (0.096, 0.388)	0.213 (0.094, 0.361)	0.746 (0.293, 1.463)	0.579 (0.250, 1.109)	0.503 (0.215, 0.873)	0.479 (0.211, 0.811)
Assam	0.433 (0.077, 1.119)	0.205 (0.058, 0.466)	0.164 (0.052, 0.333)	0.155 (0.046, 0.329)	0.751 (0.133, 1.939)	0.356 (0.101, 0.807)	0.285 (0.090, 0.577)	0.268 (0.079, 0.570)
Bihar	0.374 (0.073, 1.010)	0.188 (0.065, 0.425)	0.159 (0.051, 0.309)	0.151 (0.048, 0.282)	0.670 (0.131, 1.811)	0.337 (0.117, 0.761)	0.285 (0.091, 0.554)	0.271 (0.087, 0.505)
Chhattisgarh	0.949 (0.212, 1.902)	0.471 (0.159, 1.073)	0.355 (0.140, 0.798)	0.327 (0.140, 0.619)	1.469 (0.328, 2.944)	0.729 (0.246, 1.660)	0.550 (0.217, 1.235)	0.506 (0.217, 0.958)
Delhi	0.333 (0.100, 0.974)	0.161 (0.076, 0.270)	0.140 (0.067, 0.240)	0.134 (0.066, 0.238)	0.496 (0.150, 1.451)	0.240 (0.114, 0.402)	0.208 (0.100, 0.357)	0.200 (0.099, 0.354)
Goa	1.545 (0.425, 4.299)	0.842 (0.327, 1.935)	0.688 (0.287, 1.470)	0.650 (0.276, 1.434)	1.452 (0.400, 4.042)	0.791 (0.308, 1.819)	0.647 (0.269, 1.382)	0.611 (0.260, 1.348)
Gujarat	0.545 (0.075, 1.164)	0.257 (0.057, 0.594)	0.188 (0.054, 0.411)	0.170 (0.054, 0.363)	0.777 (0.107, 1.661)	0.367 (0.081, 0.848)	0.268 (0.077, 0.587)	0.243 (0.077, 0.518)
Haryana	0.602 (0.126, 1.624)	0.308 (0.095, 0.688)	0.260 (0.084, 0.537)	0.248 (0.079, 0.484)	0.815 (0.171, 2.199)	0.417 (0.129, 0.932)	0.352 (0.114, 0.728)	0.336 (0.107, 0.656)
Himachal Pradesh	0.890 (0.210, 2.098)	0.489 (0.174, 1.083)	0.376 (0.123, 0.773)	0.344 (0.123, 0.704)	0.965 (0.228, 2.275)	0.531 (0.188, 1.175)	0.408 (0.134, 0.838)	0.373 (0.134, 0.764)
Jammu and Kashmir	0.682 (0.141, 1.667)	0.367 (0.106, 0.908)	0.284 (0.093, 0.580)	0.259 (0.093, 0.512)	1.052 (0.218, 2.570)	0.567 (0.163, 1.399)	0.437 (0.143, 0.894)	0.400 (0.143, 0.789)
Jharkhand	0.616 (0.081, 1.977)	0.268 (0.054, 0.766)	0.197 (0.049, 0.473)	0.182 (0.039, 0.451)	0.944 (0.124, 3.028)	0.410 (0.082, 1.173)	0.302 (0.075, 0.725)	0.278 (0.059, 0.691)
Karnataka	0.628 (0.100, 1.470)	0.297 (0.098, 0.694)	0.241 (0.097, 0.509)	0.228 (0.090, 0.410)	0.762 (0.121, 1.784)	0.360 (0.119, 0.842)	0.293 (0.117, 0.618)	0.276 (0.110, 0.498)
Kerala	0.699 (0.119, 1.660)	0.328 (0.101, 1.033)	0.242 (0.088, 0.801)	0.220 (0.085, 0.731)	0.563 (0.096, 1.336)	0.264 (0.082, 0.831)	0.195 (0.071, 0.645)	0.177 (0.068, 0.588)
Madhya Pradesh	0.825 (0.110, 1.745)	0.366 (0.083, 0.868)	0.261 (0.081, 0.534)	0.235 (0.073, 0.439)	1.310 (0.175, 2.770)	0.580 (0.132, 1.378)	0.414 (0.128, 0.848)	0.373 (0.116, 0.698)
Maharashtra	1.195 (0.264, 2.758)	0.563 (0.208, 1.127)	0.431 (0.192, 0.786)	0.398 (0.192, 0.668)	1.394 (0.308, 3.217)	0.657 (0.243, 1.315)	0.503 (0.224, 0.917)	0.465 (0.224, 0.779)
Manipur	0.475 (0.186, 0.932)	0.369 (0.159, 0.709)	0.321 (0.137, 0.558)	0.306 (0.134, 0.519)	0.751 (0.294, 1.473)	0.583 (0.251, 1.120)	0.507 (0.217, 0.882)	0.483 (0.212, 0.819)
Meghalaya	0.355 (0.139, 0.695)	0.276 (0.119, 0.530)	0.240 (0.103, 0.417)	0.228 (0.100, 0.387)	0.754 (0.296, 1.476)	0.585 (0.252, 1.125)	0.509 (0.218, 0.885)	0.485 (0.213, 0.822)
Mizoram	0.456 (0.179, 0.894)	0.354 (0.152, 0.681)	0.308 (0.131, 0.536)	0.293 (0.129, 0.496)	0.767 (0.300, 1.502)	0.596 (0.256, 1.145)	0.518 (0.221, 0.901)	0.493 (0.216, 0.834)
Nagaland	0.402 (0.158, 0.789)	0.312 (0.135, 0.598)	0.271 (0.116, 0.471)	0.258 (0.114, 0.438)	0.736 (0.290, 1.445)	0.572 (0.247, 1.096)	0.497 (0.213, 0.863)	0.473 (0.209, 0.802)
Odisha	0.426 (0.064, 1.058)	0.188 (0.060, 0.549)	0.154 (0.054, 0.409)	0.148 (0.044, 0.389)	0.532 (0.080, 1.321)	0.235 (0.075, 0.685)	0.192 (0.067, 0.511)	0.184 (0.055, 0.485)
Punjab	1.099 (0.208, 2.714)	0.501 (0.167, 1.029)	0.368 (0.153, 0.646)	0.337 (0.140, 0.563)	1.217 (0.230, 3.004)	0.554 (0.185, 1.139)	0.408 (0.170, 0.715)	0.373 (0.155, 0.623)
Rajasthan	0.461 (0.092, 1.045)	0.221 (0.064, 0.567)	0.166 (0.063, 0.421)	0.153 (0.063, 0.378)	0.752 (0.150, 1.706)	0.360 (0.104, 0.925)	0.271 (0.102, 0.687)	0.249 (0.102, 0.617)
Sikkim	0.508 (0.199, 1.000)	0.395 (0.169, 0.752)	0.343 (0.146, 0.594)	0.327 (0.143, 0.549)	0.741 (0.290, 1.457)	0.575 (0.247, 1.096)	0.500 (0.212, 0.865)	0.476 (0.208, 0.801)
Tamil Nadu	0.603 (0.166, 1.392)	0.260 (0.125, 0.547)	0.201 (0.095, 0.407)	0.188 (0.089, 0.353)	0.677 (0.186, 1.564)	0.292 (0.141, 0.614)	0.226 (0.107, 0.457)	0.211 (0.100, 0.396)
Telangana	0.423 (0.093, 0.990)	0.182 (0.067, 0.362)	0.136 (0.062, 0.280)	0.125 (0.061, 0.232)	0.568 (0.125, 1.331)	0.245 (0.090, 0.486)	0.183 (0.083, 0.377)	0.168 (0.082, 0.312)
Tripura	0.532 (0.128, 1.222)	0.303 (0.087, 0.724)	0.252 (0.084, 0.581)	0.239 (0.081, 0.572)	0.720 (0.173, 1.653)	0.410 (0.117, 0.979)	0.341 (0.113, 0.786)	0.323 (0.109, 0.774)
Uttar Pradesh	0.806 (0.147, 1.851)	0.372 (0.103, 0.895)	0.260 (0.095, 0.553)	0.234 (0.091, 0.471)	1.454 (0.265, 3.340)	0.671 (0.185, 1.614)	0.468 (0.171, 0.998)	0.423 (0.164, 0.849)
Uttarakhand	0.956 (0.197, 2.142)	0.529 (0.162, 1.350)	0.395 (0.143, 0.813)	0.350 (0.140, 0.637)	1.351 (0.279, 3.029)	0.749 (0.229, 1.908)	0.558 (0.202, 1.150)	0.494 (0.198, 0.901)
West Bengal	0.649 (0.101, 1.603)	0.284 (0.081, 0.756)	0.215 (0.074, 0.522)	0.198 (0.074, 0.397)	0.819 (0.128, 2.024)	0.358 (0.103, 0.955)	0.271 (0.094, 0.660)	0.250 (0.093, 0.501)
Nepal	0.415 (0.147, 1.043)	0.321 (0.112, 0.793)	0.277 (0.097, 0.616)	0.261 (0.097, 0.560)	0.660 (0.233, 1.657)	0.511 (0.178, 1.259)	0.440 (0.154, 0.978)	0.415 (0.154, 0.890)
Pakistan	0.155 (0.054, 0.332)	0.121 (0.040, 0.257)	0.106 (0.035, 0.201)	0.101 (0.035, 0.188)	0.375 (0.130, 0.805)	0.294 (0.098, 0.624)	0.256 (0.086, 0.488)	0.244 (0.086, 0.455)
Azad Jammu & Kashmir	0.176 (0.061, 0.379)	0.138 (0.046, 0.293)	0.120 (0.041, 0.230)	0.115 (0.041, 0.214)	0.374 (0.130, 0.804)	0.293 (0.097, 0.621)	0.256 (0.086, 0.487)	0.243 (0.086, 0.454)
Balochistan	0.102 (0.035, 0.219)	0.080 (0.027, 0.170)	0.070 (0.023, 0.133)	0.066 (0.023, 0.124)	0.376 (0.130, 0.807)	0.295 (0.098, 0.627)	0.257 (0.086, 0.489)	0.245 (0.086, 0.457)
Gilgit-Baltistan	0.108 (0.037, 0.233)	0.085 (0.028, 0.181)	0.074 (0.025, 0.141)	0.071 (0.025, 0.132)	0.375 (0.129, 0.804)	0.294 (0.098, 0.624)	0.256 (0.086, 0.487)	0.244 (0.086, 0.455)
Islamabad Capital Territory	0.126 (0.045, 0.272)	0.099 (0.033, 0.209)	0.086 (0.030, 0.166)	0.082 (0.030, 0.153)	0.375 (0.135, 0.810)	0.294 (0.099, 0.621)	0.256 (0.090, 0.494)	0.244 (0.089, 0.454)

Khyber Pakhtunkhwa	0.186 (0.052, 0.499)	0.111 (0.035, 0.226)	0.095 (0.029, 0.182)	0.090 (0.028, 0.181)	0.517 (0.146, 1.385)	0.307 (0.097, 0.626)	0.264 (0.080, 0.506)	0.250 (0.079, 0.502)
Punjab	0.182 (0.042, 0.385)	0.113 (0.029, 0.288)	0.097 (0.024, 0.225)	0.091 (0.021, 0.210)	0.394 (0.092, 0.833)	0.245 (0.063, 0.624)	0.209 (0.053, 0.488)	0.198 (0.045, 0.455)
Sindh	0.210 (0.060, 0.599)	0.126 (0.046, 0.241)	0.107 (0.038, 0.198)	0.101 (0.036, 0.182)	0.549 (0.155, 1.562)	0.328 (0.120, 0.629)	0.279 (0.098, 0.517)	0.264 (0.093, 0.475)
**Southeast Asia, east Asia, and Oceania**								
**East Asia**								
China	0.853 (0.550, 1.333)	0.673 (0.497, 1.056)	0.590 (0.459, 0.827)	0.564 (0.413, 0.772)	0.646 (0.417, 1.010)	0.510 (0.376, 0.800)	0.447 (0.348, 0.626)	0.427 (0.313, 0.585)
Beijing	0.871 (0.565, 1.355)	0.687 (0.516, 1.088)	0.603 (0.464, 0.843)	0.576 (0.434, 0.785)	0.656 (0.426, 1.022)	0.518 (0.389, 0.820)	0.454 (0.350, 0.635)	0.434 (0.327, 0.592)
Guangdong	0.684 (0.442, 1.072)	0.540 (0.398, 0.848)	0.474 (0.368, 0.662)	0.453 (0.330, 0.620)	0.648 (0.418, 1.015)	0.511 (0.376, 0.803)	0.449 (0.348, 0.627)	0.428 (0.313, 0.587)
Hainan	0.702 (0.452, 1.099)	0.554 (0.407, 0.868)	0.486 (0.377, 0.679)	0.464 (0.338, 0.637)	0.648 (0.417, 1.015)	0.512 (0.376, 0.801)	0.449 (0.348, 0.627)	0.429 (0.312, 0.588)
Hebei	0.799 (0.515, 1.245)	0.631 (0.466, 0.991)	0.553 (0.429, 0.776)	0.528 (0.389, 0.721)	0.645 (0.416, 1.005)	0.509 (0.376, 0.800)	0.446 (0.346, 0.626)	0.426 (0.314, 0.582)
Henan	0.774 (0.499, 1.208)	0.611 (0.451, 0.960)	0.536 (0.416, 0.751)	0.512 (0.376, 0.700)	0.645 (0.416, 1.007)	0.509 (0.376, 0.800)	0.447 (0.347, 0.626)	0.426 (0.313, 0.583)
Hong Kong Special Administrative Region of China	1.324 (0.857, 2.057)	1.045 (0.781, 1.644)	0.917 (0.709, 1.288)	0.876 (0.650, 1.189)	0.634 (0.411, 0.985)	0.501 (0.374, 0.788)	0.439 (0.340, 0.617)	0.419 (0.311, 0.569)
Hubei	0.955 (0.616, 1.495)	0.754 (0.556, 1.183)	0.661 (0.514, 0.925)	0.632 (0.461, 0.865)	0.644 (0.415, 1.008)	0.509 (0.375, 0.798)	0.446 (0.347, 0.624)	0.426 (0.311, 0.583)
Shandong	0.925 (0.597, 1.440)	0.730 (0.541, 1.147)	0.640 (0.497, 0.899)	0.612 (0.451, 0.833)	0.642 (0.414, 0.999)	0.507 (0.376, 0.796)	0.445 (0.345, 0.625)	0.425 (0.313, 0.578)
Shanghai	0.979 (0.635, 1.532)	0.773 (0.573, 1.218)	0.678 (0.525, 0.946)	0.648 (0.479, 0.886)	0.648 (0.420, 1.014)	0.512 (0.380, 0.806)	0.449 (0.347, 0.626)	0.429 (0.317, 0.587)
Democratic People's Republic of Korea	0.724 (0.465, 1.137)	0.572 (0.416, 0.894)	0.502 (0.386, 0.699)	0.479 (0.344, 0.658)	0.639 (0.410, 1.003)	0.505 (0.367, 0.789)	0.443 (0.341, 0.617)	0.423 (0.304, 0.581)
Taiwan (province of China)	1.111 (0.724, 1.732)	0.877 (0.658, 1.383)	0.769 (0.591, 1.079)	0.735 (0.557, 0.992)	0.649 (0.423, 1.012)	0.513 (0.384, 0.808)	0.450 (0.346, 0.630)	0.429 (0.326, 0.580)
**Oceania**								
Fiji	0.447 (0.291, 0.695)	0.353 (0.266, 0.557)	0.309 (0.239, 0.437)	0.296 (0.225, 0.401)	0.664 (0.433, 1.034)	0.524 (0.395, 0.828)	0.460 (0.355, 0.650)	0.439 (0.334, 0.597)
Guam	0.709 (0.453, 1.123)	0.560 (0.412, 0.879)	0.491 (0.372, 0.686)	0.469 (0.355, 0.637)	0.672 (0.429, 1.064)	0.530 (0.390, 0.832)	0.465 (0.352, 0.649)	0.444 (0.336, 0.603)
Northern Mariana Islands	0.684 (0.440, 1.081)	0.539 (0.401, 0.846)	0.473 (0.362, 0.667)	0.452 (0.342, 0.615)	0.671 (0.432, 1.061)	0.529 (0.394, 0.830)	0.465 (0.355, 0.655)	0.444 (0.335, 0.603)
Papua New Guinea	0.257 (0.165, 0.401)	0.203 (0.150, 0.317)	0.178 (0.138, 0.249)	0.170 (0.125, 0.233)	0.657 (0.423, 1.025)	0.519 (0.382, 0.811)	0.455 (0.353, 0.637)	0.435 (0.318, 0.596)
Vanuatu	0.315 (0.205, 0.488)	0.249 (0.187, 0.392)	0.218 (0.168, 0.307)	0.209 (0.157, 0.283)	0.648 (0.422, 1.001)	0.511 (0.384, 0.805)	0.449 (0.346, 0.630)	0.428 (0.323, 0.582)
**Southeast Asia**								
Cambodia	0.393 (0.253, 0.615)	0.310 (0.228, 0.485)	0.272 (0.211, 0.380)	0.260 (0.188, 0.356)	0.642 (0.413, 1.005)	0.507 (0.372, 0.793)	0.445 (0.345, 0.621)	0.425 (0.308, 0.582)
Indonesia	0.467 (0.302, 0.729)	0.369 (0.274, 0.581)	0.324 (0.251, 0.454)	0.309 (0.230, 0.422)	0.657 (0.425, 1.025)	0.519 (0.386, 0.817)	0.455 (0.352, 0.639)	0.435 (0.323, 0.593)
Laos	0.336 (0.216, 0.525)	0.265 (0.195, 0.415)	0.233 (0.181, 0.325)	0.222 (0.162, 0.304)	0.646 (0.416, 1.009)	0.510 (0.376, 0.798)	0.447 (0.348, 0.626)	0.427 (0.312, 0.584)
Malaysia	0.488 (0.318, 0.759)	0.385 (0.290, 0.609)	0.338 (0.259, 0.475)	0.323 (0.245, 0.436)	0.650 (0.423, 1.010)	0.513 (0.385, 0.810)	0.450 (0.345, 0.632)	0.430 (0.326, 0.580)
Maldives	0.374 (0.242, 0.583)	0.295 (0.219, 0.464)	0.259 (0.201, 0.364)	0.247 (0.183, 0.337)	0.645 (0.417, 1.005)	0.509 (0.377, 0.800)	0.447 (0.346, 0.627)	0.427 (0.315, 0.582)
Mauritius	0.842 (0.550, 1.303)	0.665 (0.502, 1.049)	0.583 (0.448, 0.821)	0.557 (0.424, 0.754)	0.654 (0.427, 1.012)	0.516 (0.390, 0.815)	0.453 (0.348, 0.637)	0.433 (0.329, 0.585)
Myanmar	0.477 (0.307, 0.744)	0.376 (0.278, 0.590)	0.330 (0.257, 0.462)	0.315 (0.230, 0.431)	0.645 (0.415, 1.007)	0.509 (0.376, 0.797)	0.446 (0.347, 0.625)	0.426 (0.312, 0.583)
Philippines	0.384 (0.248, 0.599)	0.303 (0.224, 0.476)	0.266 (0.206, 0.373)	0.254 (0.187, 0.347)	0.646 (0.417, 1.008)	0.510 (0.377, 0.801)	0.447 (0.347, 0.627)	0.427 (0.315, 0.583)
Seychelles	0.640 (0.417, 0.990)	0.505 (0.381, 0.797)	0.443 (0.341, 0.623)	0.423 (0.322, 0.573)	0.651 (0.424, 1.007)	0.514 (0.388, 0.811)	0.451 (0.347, 0.634)	0.431 (0.327, 0.583)
Sri Lanka	0.674 (0.436, 1.046)	0.532 (0.395, 0.834)	0.467 (0.362, 0.655)	0.446 (0.330, 0.606)	0.638 (0.412, 0.989)	0.503 (0.374, 0.789)	0.442 (0.342, 0.620)	0.422 (0.312, 0.574)
Thailand	0.927 (0.602, 1.436)	0.732 (0.551, 1.155)	0.642 (0.495, 0.900)	0.613 (0.463, 0.830)	0.647 (0.420, 1.002)	0.511 (0.385, 0.806)	0.448 (0.345, 0.628)	0.428 (0.323, 0.579)
Timor-Leste	0.333 (0.214, 0.522)	0.263 (0.192, 0.411)	0.231 (0.178, 0.322)	0.220 (0.159, 0.302)	0.650 (0.417, 1.020)	0.513 (0.375, 0.803)	0.450 (0.347, 0.628)	0.430 (0.311, 0.590)
Vietnam	0.560 (0.360, 0.876)	0.442 (0.325, 0.692)	0.388 (0.301, 0.542)	0.370 (0.269, 0.507)	0.644 (0.415, 1.008)	0.509 (0.374, 0.797)	0.446 (0.347, 0.624)	0.426 (0.310, 0.584)
**Sub-Saharan Africa**								
**Central sub-Saharan Africa**								
Angola	0.151 (0.069, 0.272)	0.118 (0.063, 0.205)	0.103 (0.056, 0.179)	0.099 (0.052, 0.161)	0.494 (0.226, 0.893)	0.388 (0.208, 0.673)	0.339 (0.183, 0.586)	0.323 (0.172, 0.530)
Central African Republic	0.159 (0.073, 0.286)	0.124 (0.066, 0.215)	0.109 (0.059, 0.188)	0.104 (0.056, 0.170)	0.491 (0.225, 0.885)	0.386 (0.206, 0.666)	0.337 (0.183, 0.581)	0.322 (0.173, 0.527)
Congo (Brazzaville)	0.208 (0.095, 0.376)	0.163 (0.087, 0.283)	0.142 (0.078, 0.247)	0.136 (0.073, 0.222)	0.497 (0.226, 0.898)	0.390 (0.209, 0.678)	0.341 (0.186, 0.590)	0.325 (0.174, 0.530)
DR Congo	0.167 (0.076, 0.301)	0.131 (0.070, 0.227)	0.114 (0.062, 0.197)	0.109 (0.058, 0.178)	0.489 (0.223, 0.884)	0.384 (0.205, 0.666)	0.336 (0.181, 0.580)	0.320 (0.171, 0.524)
Equatorial Guinea	0.150 (0.069, 0.272)	0.118 (0.062, 0.206)	0.103 (0.057, 0.179)	0.098 (0.052, 0.158)	0.501 (0.230, 0.909)	0.393 (0.208, 0.687)	0.343 (0.190, 0.596)	0.328 (0.173, 0.529)
Gabon	0.258 (0.118, 0.467)	0.202 (0.107, 0.353)	0.177 (0.097, 0.307)	0.169 (0.090, 0.273)	0.501 (0.230, 0.908)	0.393 (0.209, 0.686)	0.343 (0.189, 0.596)	0.328 (0.174, 0.531)
**Eastern sub-Saharan Africa**								
Burundi	0.141 (0.060, 0.282)	0.110 (0.050, 0.212)	0.097 (0.047, 0.181)	0.092 (0.043, 0.161)	0.442 (0.188, 0.885)	0.347 (0.158, 0.666)	0.304 (0.146, 0.568)	0.290 (0.136, 0.506)
Comoros	0.270 (0.114, 0.541)	0.212 (0.096, 0.408)	0.185 (0.091, 0.347)	0.177 (0.083, 0.308)	0.446 (0.188, 0.894)	0.350 (0.158, 0.673)	0.306 (0.150, 0.574)	0.292 (0.137, 0.509)
Djibouti	0.189 (0.080, 0.379)	0.148 (0.067, 0.286)	0.130 (0.063, 0.244)	0.124 (0.058, 0.216)	0.452 (0.191, 0.907)	0.355 (0.160, 0.684)	0.311 (0.151, 0.583)	0.296 (0.139, 0.517)
Eritrea	0.144 (0.061, 0.288)	0.113 (0.052, 0.217)	0.099 (0.048, 0.185)	0.094 (0.044, 0.165)	0.441 (0.188, 0.882)	0.346 (0.158, 0.664)	0.303 (0.147, 0.566)	0.289 (0.136, 0.504)
Ethiopia	0.148 (0.063, 0.297)	0.117 (0.053, 0.224)	0.102 (0.049, 0.191)	0.097 (0.045, 0.170)	0.439 (0.186, 0.879)	0.344 (0.157, 0.661)	0.301 (0.145, 0.564)	0.288 (0.134, 0.502)
Kenya	0.153 (0.071, 0.336)	0.121 (0.054, 0.264)	0.107 (0.050, 0.221)	0.102 (0.045, 0.206)	0.407 (0.189, 0.897)	0.322 (0.143, 0.705)	0.284 (0.134, 0.590)	0.271 (0.120, 0.549)
Madagascar	0.151 (0.064, 0.302)	0.118 (0.054, 0.227)	0.104 (0.050, 0.194)	0.099 (0.046, 0.173)	0.446 (0.190, 0.893)	0.350 (0.159, 0.673)	0.307 (0.149, 0.574)	0.293 (0.137, 0.511)
Malawi	0.151 (0.064, 0.303)	0.119 (0.054, 0.228)	0.104 (0.051, 0.195)	0.099 (0.047, 0.173)	0.445 (0.189, 0.892)	0.349 (0.158, 0.672)	0.306 (0.149, 0.573)	0.292 (0.137, 0.508)
Mozambique	0.138 (0.044, 0.303)	0.108 (0.036, 0.237)	0.095 (0.035, 0.205)	0.091 (0.033, 0.193)	0.441 (0.141, 0.969)	0.344 (0.115, 0.758)	0.304 (0.112, 0.656)	0.292 (0.107, 0.618)
Rwanda	0.178 (0.076, 0.358)	0.140 (0.063, 0.270)	0.123 (0.059, 0.230)	0.117 (0.055, 0.204)	0.446 (0.189, 0.895)	0.350 (0.159, 0.674)	0.306 (0.148, 0.575)	0.292 (0.137, 0.511)
Somalia	0.118 (0.051, 0.237)	0.093 (0.043, 0.178)	0.081 (0.039, 0.152)	0.078 (0.037, 0.135)	0.444 (0.190, 0.886)	0.349 (0.160, 0.667)	0.305 (0.148, 0.569)	0.291 (0.137, 0.507)
South Sudan	0.158 (0.067, 0.316)	0.124 (0.056, 0.238)	0.108 (0.053, 0.203)	0.103 (0.049, 0.179)	0.447 (0.190, 0.894)	0.351 (0.160, 0.674)	0.307 (0.151, 0.574)	0.293 (0.139, 0.508)
Zambia	0.142 (0.060, 0.286)	0.112 (0.050, 0.216)	0.098 (0.048, 0.184)	0.093 (0.044, 0.162)	0.451 (0.190, 0.906)	0.354 (0.160, 0.684)	0.310 (0.152, 0.583)	0.296 (0.139, 0.515)
**Southern sub-Saharan Africa**								
Botswana	0.269 (0.130, 0.504)	0.212 (0.114, 0.370)	0.185 (0.098, 0.317)	0.177 (0.093, 0.299)	0.540 (0.261, 1.011)	0.424 (0.228, 0.741)	0.371 (0.196, 0.635)	0.354 (0.186, 0.599)
Eswatini	0.226 (0.110, 0.424)	0.178 (0.096, 0.313)	0.156 (0.083, 0.267)	0.149 (0.079, 0.252)	0.534 (0.258, 1.001)	0.420 (0.227, 0.739)	0.368 (0.197, 0.629)	0.351 (0.187, 0.596)
Lesotho	0.272 (0.132, 0.509)	0.214 (0.115, 0.374)	0.187 (0.100, 0.320)	0.179 (0.095, 0.302)	0.540 (0.262, 1.009)	0.424 (0.228, 0.741)	0.371 (0.198, 0.634)	0.354 (0.188, 0.598)
Namibia	0.277 (0.135, 0.519)	0.218 (0.117, 0.379)	0.191 (0.101, 0.326)	0.182 (0.095, 0.307)	0.535 (0.260, 1.001)	0.421 (0.226, 0.731)	0.368 (0.195, 0.628)	0.351 (0.184, 0.592)
South Africa	0.511 (0.199, 1.275)	0.400 (0.173, 0.987)	0.348 (0.145, 0.774)	0.331 (0.137, 0.724)	0.713 (0.278, 1.780)	0.558 (0.241, 1.377)	0.486 (0.202, 1.081)	0.462 (0.191, 1.010)
Zimbabwe	0.210 (0.102, 0.391)	0.165 (0.089, 0.285)	0.144 (0.077, 0.245)	0.138 (0.072, 0.231)	0.537 (0.262, 1.001)	0.422 (0.227, 0.730)	0.369 (0.196, 0.629)	0.352 (0.186, 0.592)
**Western sub-Saharan Africa**								
Benin	0.151 (0.065, 0.287)	0.118 (0.055, 0.215)	0.103 (0.051, 0.183)	0.098 (0.050, 0.171)	0.451 (0.195, 0.858)	0.353 (0.163, 0.643)	0.308 (0.153, 0.546)	0.294 (0.151, 0.510)
Burkina Faso	0.155 (0.067, 0.294)	0.121 (0.056, 0.220)	0.106 (0.052, 0.188)	0.101 (0.052, 0.176)	0.451 (0.196, 0.858)	0.354 (0.164, 0.643)	0.309 (0.153, 0.548)	0.294 (0.152, 0.512)
Cape Verde	0.325 (0.141, 0.620)	0.255 (0.116, 0.465)	0.222 (0.109, 0.395)	0.212 (0.107, 0.370)	0.450 (0.195, 0.858)	0.353 (0.161, 0.643)	0.308 (0.151, 0.547)	0.293 (0.148, 0.512)
Cameroon	0.159 (0.068, 0.302)	0.124 (0.057, 0.227)	0.109 (0.054, 0.193)	0.103 (0.053, 0.179)	0.453 (0.195, 0.864)	0.355 (0.163, 0.648)	0.310 (0.155, 0.550)	0.296 (0.151, 0.511)
Chad	0.132 (0.058, 0.251)	0.104 (0.048, 0.188)	0.090 (0.045, 0.161)	0.086 (0.045, 0.150)	0.453 (0.197, 0.859)	0.355 (0.165, 0.643)	0.310 (0.154, 0.550)	0.295 (0.153, 0.515)
Côte d'Ivoire	0.157 (0.068, 0.299)	0.123 (0.057, 0.225)	0.108 (0.054, 0.191)	0.103 (0.053, 0.178)	0.454 (0.197, 0.863)	0.356 (0.164, 0.647)	0.310 (0.154, 0.550)	0.296 (0.152, 0.514)
The Gambia	0.171 (0.074, 0.326)	0.134 (0.062, 0.244)	0.117 (0.058, 0.208)	0.112 (0.057, 0.194)	0.453 (0.196, 0.860)	0.355 (0.164, 0.645)	0.309 (0.153, 0.549)	0.295 (0.151, 0.513)
Ghana	0.197 (0.085, 0.375)	0.154 (0.071, 0.281)	0.135 (0.067, 0.239)	0.128 (0.065, 0.223)	0.450 (0.195, 0.857)	0.353 (0.162, 0.642)	0.308 (0.152, 0.546)	0.293 (0.150, 0.510)
Guinea	0.173 (0.075, 0.329)	0.136 (0.063, 0.247)	0.119 (0.059, 0.210)	0.113 (0.059, 0.197)	0.454 (0.197, 0.862)	0.356 (0.165, 0.646)	0.311 (0.155, 0.551)	0.296 (0.153, 0.515)
Guinea-Bissau	0.143 (0.062, 0.272)	0.112 (0.052, 0.204)	0.098 (0.049, 0.174)	0.093 (0.048, 0.162)	0.449 (0.195, 0.852)	0.352 (0.164, 0.638)	0.307 (0.152, 0.545)	0.293 (0.152, 0.510)
Liberia	0.171 (0.074, 0.324)	0.134 (0.062, 0.243)	0.117 (0.058, 0.207)	0.111 (0.057, 0.193)	0.451 (0.195, 0.858)	0.353 (0.163, 0.644)	0.309 (0.154, 0.547)	0.294 (0.151, 0.510)
Mali	0.151 (0.066, 0.286)	0.118 (0.055, 0.215)	0.103 (0.051, 0.183)	0.098 (0.051, 0.171)	0.452 (0.197, 0.859)	0.354 (0.164, 0.644)	0.309 (0.153, 0.549)	0.295 (0.152, 0.514)
Mauritania	0.213 (0.092, 0.406)	0.167 (0.076, 0.305)	0.146 (0.072, 0.259)	0.139 (0.070, 0.240)	0.467 (0.201, 0.891)	0.366 (0.167, 0.668)	0.319 (0.159, 0.568)	0.304 (0.154, 0.527)
Niger	0.124 (0.054, 0.236)	0.097 (0.045, 0.176)	0.085 (0.042, 0.151)	0.081 (0.042, 0.141)	0.450 (0.196, 0.856)	0.353 (0.164, 0.640)	0.308 (0.152, 0.547)	0.293 (0.152, 0.512)
Nigeria	0.114 (0.038, 0.255)	0.088 (0.028, 0.204)	0.078 (0.026, 0.173)	0.074 (0.023, 0.170)	0.324 (0.108, 0.726)	0.252 (0.079, 0.582)	0.221 (0.074, 0.494)	0.211 (0.067, 0.485)
São Tomé and Príncipe	0.201 (0.087, 0.383)	0.158 (0.072, 0.287)	0.138 (0.068, 0.244)	0.131 (0.067, 0.229)	0.448 (0.194, 0.853)	0.351 (0.161, 0.640)	0.307 (0.151, 0.544)	0.292 (0.149, 0.509)
Senegal	0.194 (0.084, 0.369)	0.152 (0.070, 0.277)	0.133 (0.066, 0.236)	0.127 (0.065, 0.220)	0.453 (0.196, 0.861)	0.355 (0.164, 0.645)	0.310 (0.153, 0.549)	0.295 (0.152, 0.514)
Sierra Leone	0.172 (0.075, 0.327)	0.135 (0.063, 0.245)	0.118 (0.058, 0.209)	0.112 (0.058, 0.196)	0.456 (0.198, 0.867)	0.357 (0.166, 0.650)	0.312 (0.154, 0.554)	0.297 (0.153, 0.518)
Togo	0.174 (0.076, 0.331)	0.136 (0.063, 0.248)	0.119 (0.059, 0.211)	0.113 (0.058, 0.197)	0.450 (0.196, 0.856)	0.353 (0.163, 0.641)	0.308 (0.152, 0.546)	0.293 (0.151, 0.511)
Tanzania	0.169 (0.071, 0.339)	0.133 (0.060, 0.256)	0.116 (0.057, 0.218)	0.111 (0.052, 0.193)	0.449 (0.189, 0.901)	0.352 (0.159, 0.679)	0.308 (0.151, 0.579)	0.294 (0.139, 0.512)
Uganda	0.131 (0.056, 0.263)	0.103 (0.047, 0.198)	0.090 (0.044, 0.169)	0.086 (0.040, 0.150)	0.444 (0.188, 0.890)	0.349 (0.158, 0.671)	0.305 (0.148, 0.572)	0.291 (0.136, 0.508)

Section 8: Author contributions

Managing the estimation or publications process
Emmanuela Gakidou, Simon I Hay, Christopher J L Murray, and Robert C Reiner Jr.

Writing the first draft of the manuscript
Christopher J L Murray and Reed J D Sorensen.

Primary responsibility for applying analytical methods to produce estimates
Ryan M Barber, Austin Carter, Reed J D Sorensen, and Cory N Spencer.

Primary responsibility for seeking, cataloguing, extracting, or cleaning data; designing or coding figures and tables
1Ferrari, Gaorui Guo, Monika Helak, Erin N Hulland, Alice Lazzar-Atwood, Kate E LeGrand, Akiaja Lindstrom, Johan Månsson, Ali H Mokdad, Christopher J L Murray, Mohsen Naghavi, Hasan Nassereldine, Latera Tesfaye Olana, Samuel M Ostroff, Maja Pasovic, Louise Penberthy, David M Pigott, Damian Francesco Santomauro, Reed J D Sorensen, Cory N Spencer, Emma Elizabeth Spurlock, Ruri Syailendrawati, Anh Truc Vo, and Stefanie Watson.

Providing data or critical feedback on data sources
Cristiana Abbafati, Christopher Adolph, Bree L Bang-Jensen, Ryan M Barber, Rachel Castellano, Haley Comfort, Xiaochen Dai, William James Dangel, Carolyn Dapper, Megan Erickson, Abraham D Flaxman, Nancy Fullman, Emmanuela Gakidou, Amiran Gamkrelidze, John R Giles, Gaorui Guo, Simon I Hay, Monika Helak, Erin N Hulland, Maia Kereselidze, Kate E LeGrand, Rafael Lozano, Beatrice Magistro, Deborah Carvalho Malta, Ana M Mantilla Herrera, Fatima Marinho, Alemnesh H Mirkuzie, Ali H Mokdad, Lorenzo Monasta, Christopher J L Murray, Mohsen Naghavi, Hasan Nassereldine, Shuhei Nomura, Latera Tesfaye Olana, Maja Pasovic, Spencer A Pease, David M Pigott, Grace Reinke, Antonio Luiz P Ribeiro, Damian Francesco Santomauro, Natia Skhvitaridze, Reed J D Sorensen, Emma Elizabeth Spurlock, Ruri Syailendrawati, Roman Topor-Madry, Anh Truc Vo, Theo Vos, Rebecca Walcott, Stefanie Watson, Charles Shey Wiysonge, and Nahom Alemseged Worku.

Developing methods or computational machinery
Adrien Allorant, Aleksandr Y Aravkin, Ryan M Barber, Austin Carter, Emma Castro, James K Collins, Haley Comfort, Xiaochen Dai, Abraham D Flaxman, Joseph Jon Frostad, John R Giles, Jiawei He, Emily Linebarger, Ali H Mokdad, Christopher J L Murray, Mohsen Naghavi, Spencer A Pease, Aleksei Sholokhov, Reed J D Sorensen, Cory N Spencer, Theo Vos, and Peng Zheng.

Providing critical feedback on methods or results
Cristiana Abbafati, Austin Carter, James K Collins, William James Dangel, Emmanuela Gakidou, Amiran Gamkrelidze, Simon I Hay, Deborah Carvalho Malta, Alemnesh H Mirkuzie, Ali H Mokdad, Christopher J L Murray, Mohsen Naghavi, Christopher M Odell, Latera Tesfaye Olana, Samuel M Ostroff, Maja Pasovic, David M Pigott, Antonio Luiz P Ribeiro, Reed J D Sorensen, Cory N Spencer, Roman Topor-Madry, Theo Vos, Ally Walker, Charles Shey Wiysonge, and Nahom Alemseged Worku.

Drafting the work or revising is critically for important intellectual content
Cristiana Abbafati, Catherine Bisignano, Simon I Hay, Deborah Carvalho Malta, Alemnesh H Mirkuzie, Ali H Mokdad, Lorenzo Monasta, Christopher J L Murray, Mohsen Naghavi, Samuel M Ostroff, David M Pigott, Reed J D Sorensen, Theo Vos, and Charles Shey Wiysonge.

Managing the overall research enterprise
Joanne O Amlag, Sabina S Bloom, William James Dangel, Amanda Deen, Bethany M Huntley, Ali H Mokdad, Christopher J L Murray, Mohsen Naghavi, Christopher M Odell, Emma Elizabeth Spurlock, and Roman Topor-Madry.
