# Supplementary material for: Variation in the COVID-19 infection–fatality ratio by age, time, and geography during the pre-vaccine era: a systematic analysis
Source: Lancet. 2022 Apr 16;399(10334):1469–88. doi: 10.1016/S0140-6736(21)02867-1 (PMC8871594; doi:10.1016/S0140-6736(21)02867-1)
Supplement: Supplementary appendix 2 [file mmc2.pdf]

THE LANCET

Supplementary appendix

This appendix formed part of the original submission and has been peer reviewed. We post it as supplied by the authors.

Supplement to: COVID-19 Forecasting Team. Variation in the COVID-19 infection–fatality ratio by age, time, and geography during the pre-vaccine era: a systematic analysis. *Lancet* 2022; published online Feb 24. https://doi.org/10.1016/S0140-6736(21)02867-1.

Appendix 2: Data sources for "Variations in the COVID-19 infection-fatality ratio by age, time, and geography during the pre-vaccine era"

This appendix provides information on data sources for "Variations in the COVID-19 infection-fatality ratio by age, time, and geography during the pre-vaccine era"

Contents

Section 1: Death sources 3
Figure S1. Death data coverage by location 3
Table S1. Death data sources by location 3
Section 2: Age-stratified death sources 50
Figure S2. Age-stratified death data coverage by location 50
Table S2. Age-stratified death data sources by location 50
Section 3: Seroprevalence sources 61
Figure S3. Seroprevalence data coverage by location 61
Table S3. Seroprevalence data sources by location 61
Section 4: Age-stratified seroprevalence sources 91
Figure S4: Age-stratified seroprevalence data coverage by location 91
Table S4. Age-stratified seroprevalence data sources by location 91

Section 1: Death sources

Figure S1. Death data coverage by location

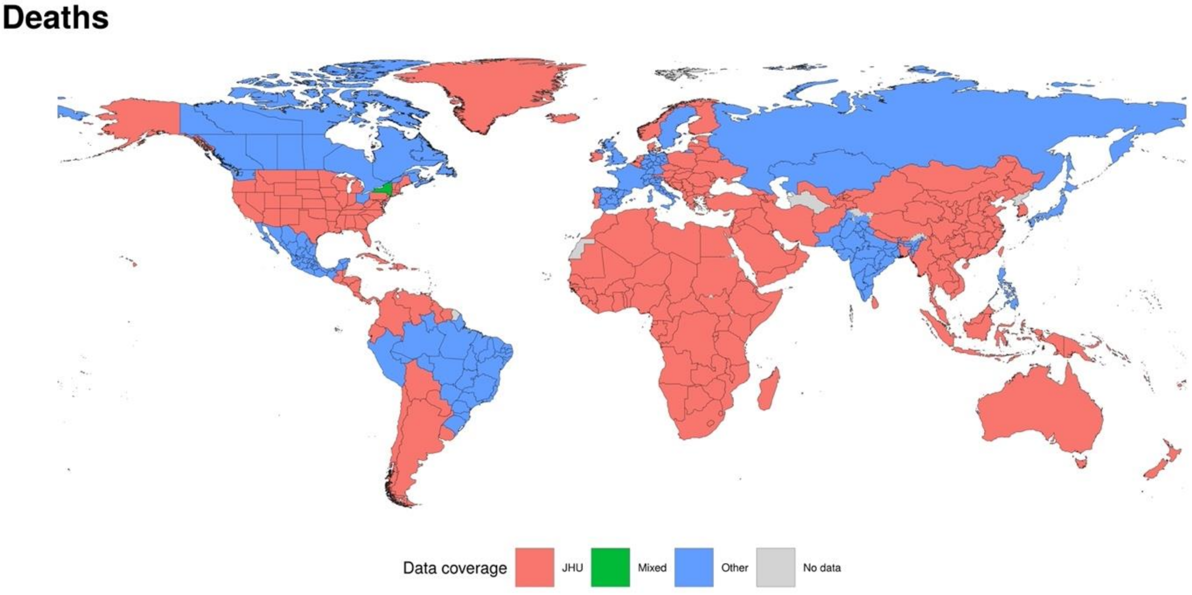

Table S1. Death data sources by location

Location	Source
Afghanistan	Johns Hopkins University. 2019 Novel Coronavirus COVID-19 (2019-nCoV) Data Repository by Johns Hopkins CSSE. Baltimore, Maryland: Johns Hopkins University.
Albania	Johns Hopkins University. 2019 Novel Coronavirus COVID-19 (2019-nCoV) Data Repository by Johns Hopkins CSSE. Baltimore, Maryland: Johns Hopkins University.
Algeria	Johns Hopkins University. 2019 Novel Coronavirus COVID-19 (2019-nCoV) Data Repository by Johns Hopkins CSSE. Baltimore, Maryland: Johns Hopkins University.
American Samoa	Johns Hopkins University. 2019 Novel Coronavirus COVID-19 (2019-nCoV) Data Repository by Johns Hopkins CSSE. Baltimore, Maryland: Johns Hopkins University.
Andorra	Johns Hopkins University. 2019 Novel Coronavirus COVID-19 (2019-nCoV) Data Repository by Johns Hopkins CSSE. Baltimore, Maryland: Johns Hopkins University.
Angola	Johns Hopkins University. 2019 Novel Coronavirus COVID-19 (2019-nCoV) Data Repository by Johns Hopkins CSSE.

	Baltimore, Maryland: Johns Hopkins University.
Antigua and Barbuda	Johns Hopkins University. 2019 Novel Coronavirus COVID-19 (2019-nCoV) Data Repository by Johns Hopkins CSSE. Baltimore, Maryland: Johns Hopkins University.
Argentina	Johns Hopkins University. 2019 Novel Coronavirus COVID-19 (2019-nCoV) Data Repository by Johns Hopkins CSSE. Baltimore, Maryland: Johns Hopkins University.
Armenia	Johns Hopkins University. 2019 Novel Coronavirus COVID-19 (2019-nCoV) Data Repository by Johns Hopkins CSSE. Baltimore, Maryland: Johns Hopkins University.
Australia	Johns Hopkins University. 2019 Novel Coronavirus COVID-19 (2019-nCoV) Data Repository by Johns Hopkins CSSE. Baltimore, Maryland: Johns Hopkins University.
Austria	Johns Hopkins University. 2019 Novel Coronavirus COVID-19 (2019-nCoV) Data Repository by Johns Hopkins CSSE. Baltimore, Maryland: Johns Hopkins University.
Azerbaijan	Johns Hopkins University. 2019 Novel Coronavirus COVID-19 (2019-nCoV) Data Repository by Johns Hopkins CSSE. Baltimore, Maryland: Johns Hopkins University.
Bahrain	Johns Hopkins University. 2019 Novel Coronavirus COVID-19 (2019-nCoV) Data Repository by Johns Hopkins CSSE. Baltimore, Maryland: Johns Hopkins University.
Bangladesh	Johns Hopkins University. 2019 Novel Coronavirus COVID-19 (2019-nCoV) Data Repository by Johns Hopkins CSSE. Baltimore, Maryland: Johns Hopkins University.
Barbados	Johns Hopkins University. 2019 Novel Coronavirus COVID-19 (2019-nCoV) Data Repository by Johns Hopkins CSSE. Baltimore, Maryland: Johns Hopkins University.
Belarus	Johns Hopkins University. 2019 Novel Coronavirus COVID-19 (2019-nCoV) Data Repository by Johns Hopkins CSSE. Baltimore, Maryland: Johns Hopkins University.

Belgium	Johns Hopkins University. 2019 Novel Coronavirus COVID-19 (2019-nCoV) Data Repository by Johns Hopkins CSSE. Baltimore, Maryland: Johns Hopkins University.
Belize	Johns Hopkins University. 2019 Novel Coronavirus COVID-19 (2019-nCoV) Data Repository by Johns Hopkins CSSE. Baltimore, Maryland: Johns Hopkins University.
Benin	Johns Hopkins University. 2019 Novel Coronavirus COVID-19 (2019-nCoV) Data Repository by Johns Hopkins CSSE. Baltimore, Maryland: Johns Hopkins University.
Bermuda	Johns Hopkins University. 2019 Novel Coronavirus COVID-19 (2019-nCoV) Data Repository by Johns Hopkins CSSE. Baltimore, Maryland: Johns Hopkins University.
Bhutan	Johns Hopkins University. 2019 Novel Coronavirus COVID-19 (2019-nCoV) Data Repository by Johns Hopkins CSSE. Baltimore, Maryland: Johns Hopkins University.
Bolivia	Johns Hopkins University. 2019 Novel Coronavirus COVID-19 (2019-nCoV) Data Repository by Johns Hopkins CSSE. Baltimore, Maryland: Johns Hopkins University.
Bosnia and Herzegovina	Johns Hopkins University. 2019 Novel Coronavirus COVID-19 (2019-nCoV) Data Repository by Johns Hopkins CSSE. Baltimore, Maryland: Johns Hopkins University.
Botswana	Johns Hopkins University. 2019 Novel Coronavirus COVID-19 (2019-nCoV) Data Repository by Johns Hopkins CSSE. Baltimore, Maryland: Johns Hopkins University.
Brazil, Acre	Ministry of Health (Brazil). Brazil Ministry of Health COVID-19 Coronavirus Panel. Rio de Janeiro, Brazil: Ministry of Health (Brazil).
Brazil, Alagoas	Ministry of Health (Brazil). Brazil Ministry of Health COVID-19 Coronavirus Panel. Rio de Janeiro, Brazil: Ministry of Health (Brazil).
Brazil, Amapá	Ministry of Health (Brazil). Brazil Ministry of Health COVID-19 Coronavirus Panel. Rio de Janeiro, Brazil: Ministry of Health (Brazil).

Brazil, Amazonas	Ministry of Health (Brazil). Brazil Ministry of Health COVID-19 Coronavirus Panel. Rio de Janeiro, Brazil: Ministry of Health (Brazil).
Brazil, Bahia	Ministry of Health (Brazil). Brazil Ministry of Health COVID-19 Coronavirus Panel. Rio de Janeiro, Brazil: Ministry of Health (Brazil).
Brazil, Ceará	Ministry of Health (Brazil). Brazil Ministry of Health COVID-19 Coronavirus Panel. Rio de Janeiro, Brazil: Ministry of Health (Brazil).
Brazil, Distrito Federal	Ministry of Health (Brazil). Brazil Ministry of Health COVID-19 Coronavirus Panel. Rio de Janeiro, Brazil: Ministry of Health (Brazil).
Brazil, Espírito Santo	Ministry of Health (Brazil). Brazil Ministry of Health COVID-19 Coronavirus Panel. Rio de Janeiro, Brazil: Ministry of Health (Brazil).
Brazil, Goiás	Ministry of Health (Brazil). Brazil Ministry of Health COVID-19 Coronavirus Panel. Rio de Janeiro, Brazil: Ministry of Health (Brazil).
Brazil, Maranhão	Ministry of Health (Brazil). Brazil Ministry of Health COVID-19 Coronavirus Panel. Rio de Janeiro, Brazil: Ministry of Health (Brazil).
Brazil, Mato Grosso	Ministry of Health (Brazil). Brazil Ministry of Health COVID-19 Coronavirus Panel. Rio de Janeiro, Brazil: Ministry of Health (Brazil).
Brazil, Mato Grosso do Sul	Ministry of Health (Brazil). Brazil Ministry of Health COVID-19 Coronavirus Panel. Rio de Janeiro, Brazil: Ministry of Health (Brazil).
Brazil, Minas Gerais	Ministry of Health (Brazil). Brazil Ministry of Health COVID-19 Coronavirus Panel. Rio de Janeiro, Brazil: Ministry of Health (Brazil).
Brazil, Pará	Ministry of Health (Brazil). Brazil Ministry of Health COVID-19 Coronavirus Panel. Rio de Janeiro, Brazil: Ministry of Health (Brazil).

Brazil, Paraíba	Ministry of Health (Brazil). Brazil Ministry of Health COVID-19 Coronavirus Panel. Rio de Janeiro, Brazil: Ministry of Health (Brazil).
Brazil, Paraná	Ministry of Health (Brazil). Brazil Ministry of Health COVID-19 Coronavirus Panel. Rio de Janeiro, Brazil: Ministry of Health (Brazil).
Brazil, Pernambuco	Ministry of Health (Brazil). Brazil Ministry of Health COVID-19 Coronavirus Panel. Rio de Janeiro, Brazil: Ministry of Health (Brazil).
Brazil, Piauí	Ministry of Health (Brazil). Brazil Ministry of Health COVID-19 Coronavirus Panel. Rio de Janeiro, Brazil: Ministry of Health (Brazil).
Brazil, Rio de Janeiro	Ministry of Health (Brazil). Brazil Ministry of Health COVID-19 Coronavirus Panel. Rio de Janeiro, Brazil: Ministry of Health (Brazil).
Brazil, Rio Grande do Norte	Ministry of Health (Brazil). Brazil Ministry of Health COVID-19 Coronavirus Panel. Rio de Janeiro, Brazil: Ministry of Health (Brazil).
Brazil, Rio Grande do Sul	Ministry of Health (Brazil). Brazil Ministry of Health COVID-19 Coronavirus Panel. Rio de Janeiro, Brazil: Ministry of Health (Brazil).
Brazil, Rondônia	Ministry of Health (Brazil). Brazil Ministry of Health COVID-19 Coronavirus Panel. Rio de Janeiro, Brazil: Ministry of Health (Brazil).
Brazil, Roraima	Ministry of Health (Brazil). Brazil Ministry of Health COVID-19 Coronavirus Panel. Rio de Janeiro, Brazil: Ministry of Health (Brazil).
Brazil, Santa Catarina	Ministry of Health (Brazil). Brazil Ministry of Health COVID-19 Coronavirus Panel. Rio de Janeiro, Brazil: Ministry of Health (Brazil).
Brazil, São Paulo	Ministry of Health (Brazil). Brazil Ministry of Health COVID-19 Coronavirus Panel. Rio de Janeiro, Brazil: Ministry of Health (Brazil).

Brazil, Sergipe	Ministry of Health (Brazil). Brazil Ministry of Health COVID-19 Coronavirus Panel. Rio de Janeiro, Brazil: Ministry of Health (Brazil).
Brazil, Tocantins	Ministry of Health (Brazil). Brazil Ministry of Health COVID-19 Coronavirus Panel. Rio de Janeiro, Brazil: Ministry of Health (Brazil).
Brunei	Johns Hopkins University. 2019 Novel Coronavirus COVID-19 (2019-nCoV) Data Repository by Johns Hopkins CSSE. Baltimore, Maryland: Johns Hopkins University.
Bulgaria	Johns Hopkins University. 2019 Novel Coronavirus COVID-19 (2019-nCoV) Data Repository by Johns Hopkins CSSE. Baltimore, Maryland: Johns Hopkins University.
Burkina Faso	Johns Hopkins University. 2019 Novel Coronavirus COVID-19 (2019-nCoV) Data Repository by Johns Hopkins CSSE. Baltimore, Maryland: Johns Hopkins University.
Burundi	Johns Hopkins University. 2019 Novel Coronavirus COVID-19 (2019-nCoV) Data Repository by Johns Hopkins CSSE. Baltimore, Maryland: Johns Hopkins University.
Cambodia	Johns Hopkins University. 2019 Novel Coronavirus COVID-19 (2019-nCoV) Data Repository by Johns Hopkins CSSE. Baltimore, Maryland: Johns Hopkins University.
Cameroon	Johns Hopkins University. 2019 Novel Coronavirus COVID-19 (2019-nCoV) Data Repository by Johns Hopkins CSSE. Baltimore, Maryland: Johns Hopkins University.
Canada, Alberta	Government of Canada. Canada Public Health Infobase Number of Total Cases of COVID-19. Ottawa, Canada: Government of Canada.
Canada, Alberta	Government of Canada. Canada Coronavirus Disease 2019 (COVID-19) Daily Epidemiology Update. Ottawa, Canada: Government of Canada.
Canada, British Columbia	Government of Canada. Canada Public Health Infobase Number of Total Cases of COVID-19. Ottawa, Canada: Government of Canada.

Canada, British Columbia	Government of Canada. Canada Coronavirus Disease 2019 (COVID-19) Daily Epidemiology Update. Ottawa, Canada: Government of Canada.
Canada, Manitoba	Government of Canada. Canada Public Health Infobase Number of Total Cases of COVID-19. Ottawa, Canada: Government of Canada.
Canada, Manitoba	Government of Canada. Canada Coronavirus Disease 2019 (COVID-19) Daily Epidemiology Update. Ottawa, Canada: Government of Canada.
Canada, New Brunswick	Government of Canada. Canada Public Health Infobase Number of Total Cases of COVID-19. Ottawa, Canada: Government of Canada.
Canada, New Brunswick	Government of Canada. Canada Coronavirus Disease 2019 (COVID-19) Daily Epidemiology Update. Ottawa, Canada: Government of Canada.
Canada, Newfoundland and Labrador	Government of Canada. Canada Public Health Infobase Number of Total Cases of COVID-19. Ottawa, Canada: Government of Canada.
Canada, Newfoundland and Labrador	Government of Canada. Canada Coronavirus Disease 2019 (COVID-19) Daily Epidemiology Update. Ottawa, Canada: Government of Canada.
Canada, Northwest Territories	Government of Canada. Canada Public Health Infobase Number of Total Cases of COVID-19. Ottawa, Canada: Government of Canada.
Canada, Northwest Territories	Government of Canada. Canada Coronavirus Disease 2019 (COVID-19) Daily Epidemiology Update. Ottawa, Canada: Government of Canada.
Canada, Nova Scotia	Government of Canada. Canada Public Health Infobase Number of Total Cases of COVID-19. Ottawa, Canada: Government of Canada.
Canada, Nova Scotia	Government of Canada. Canada Coronavirus Disease 2019 (COVID-19) Daily Epidemiology Update. Ottawa, Canada: Government of Canada.

Canada, Nunavut	Government of Canada. Canada Public Health Infobase Number of Total Cases of COVID-19. Ottawa, Canada: Government of Canada.
Canada, Nunavut	Government of Canada. Canada Coronavirus Disease 2019 (COVID-19) Daily Epidemiology Update. Ottawa, Canada: Government of Canada.
Canada, Ontario	Government of Canada. Canada Public Health Infobase Number of Total Cases of COVID-19. Ottawa, Canada: Government of Canada.
Canada, Ontario	Government of Canada. Canada Coronavirus Disease 2019 (COVID-19) Daily Epidemiology Update. Ottawa, Canada: Government of Canada.
Canada, Prince Edward Island	Government of Canada. Canada Public Health Infobase Number of Total Cases of COVID-19. Ottawa, Canada: Government of Canada.
Canada, Prince Edward Island	Government of Canada. Canada Coronavirus Disease 2019 (COVID-19) Daily Epidemiology Update. Ottawa, Canada: Government of Canada.
Canada, Quebec	Government of Canada. Canada Public Health Infobase Number of Total Cases of COVID-19. Ottawa, Canada: Government of Canada.
Canada, Quebec	Government of Canada. Canada Coronavirus Disease 2019 (COVID-19) Daily Epidemiology Update. Ottawa, Canada: Government of Canada.
Canada, Saskatchewan	Government of Canada. Canada Public Health Infobase Number of Total Cases of COVID-19. Ottawa, Canada: Government of Canada.
Canada, Saskatchewan	Government of Canada. Canada Coronavirus Disease 2019 (COVID-19) Daily Epidemiology Update. Ottawa, Canada: Government of Canada.
Canada, Yukon	Government of Canada. Canada Public Health Infobase Number of Total Cases of COVID-19. Ottawa, Canada: Government of Canada.

Canada, Yukon	Government of Canada. Canada Coronavirus Disease 2019 (COVID-19) Daily Epidemiology Update. Ottawa, Canada: Government of Canada.
Cape Verde	Johns Hopkins University. 2019 Novel Coronavirus COVID-19 (2019-nCoV) Data Repository by Johns Hopkins CSSE. Baltimore, Maryland: Johns Hopkins University.
Central African Republic	Johns Hopkins University. 2019 Novel Coronavirus COVID-19 (2019-nCoV) Data Repository by Johns Hopkins CSSE. Baltimore, Maryland: Johns Hopkins University.
Chad	Johns Hopkins University. 2019 Novel Coronavirus COVID-19 (2019-nCoV) Data Repository by Johns Hopkins CSSE. Baltimore, Maryland: Johns Hopkins University.
Chile	Johns Hopkins University. 2019 Novel Coronavirus COVID-19 (2019-nCoV) Data Repository by Johns Hopkins CSSE. Baltimore, Maryland: Johns Hopkins University.
China, Anhui	Johns Hopkins University. 2019 Novel Coronavirus COVID-19 (2019-nCoV) Data Repository by Johns Hopkins CSSE. Baltimore, Maryland: Johns Hopkins University.
China, Beijing	Johns Hopkins University. 2019 Novel Coronavirus COVID-19 (2019-nCoV) Data Repository by Johns Hopkins CSSE. Baltimore, Maryland: Johns Hopkins University.
China, Chongqing	Johns Hopkins University. 2019 Novel Coronavirus COVID-19 (2019-nCoV) Data Repository by Johns Hopkins CSSE. Baltimore, Maryland: Johns Hopkins University.
China, Fujian	Johns Hopkins University. 2019 Novel Coronavirus COVID-19 (2019-nCoV) Data Repository by Johns Hopkins CSSE. Baltimore, Maryland: Johns Hopkins University.
China, Gansu	Johns Hopkins University. 2019 Novel Coronavirus COVID-19 (2019-nCoV) Data Repository by Johns Hopkins CSSE. Baltimore, Maryland: Johns Hopkins University.
China, Guangdong	Johns Hopkins University. 2019 Novel Coronavirus COVID-19 (2019-nCoV) Data Repository by Johns Hopkins CSSE. Baltimore, Maryland: Johns Hopkins University.

China, Guangxi	Johns Hopkins University. 2019 Novel Coronavirus COVID-19 (2019-nCoV) Data Repository by Johns Hopkins CSSE. Baltimore, Maryland: Johns Hopkins University.
China, Guizhou	Johns Hopkins University. 2019 Novel Coronavirus COVID-19 (2019-nCoV) Data Repository by Johns Hopkins CSSE. Baltimore, Maryland: Johns Hopkins University.
China, Hainan	Johns Hopkins University. 2019 Novel Coronavirus COVID-19 (2019-nCoV) Data Repository by Johns Hopkins CSSE. Baltimore, Maryland: Johns Hopkins University.
China, Hebei	Johns Hopkins University. 2019 Novel Coronavirus COVID-19 (2019-nCoV) Data Repository by Johns Hopkins CSSE. Baltimore, Maryland: Johns Hopkins University.
China, Heilongjiang	Johns Hopkins University. 2019 Novel Coronavirus COVID-19 (2019-nCoV) Data Repository by Johns Hopkins CSSE. Baltimore, Maryland: Johns Hopkins University.
China, Henan	Johns Hopkins University. 2019 Novel Coronavirus COVID-19 (2019-nCoV) Data Repository by Johns Hopkins CSSE. Baltimore, Maryland: Johns Hopkins University.
China, Hong Kong Special Administrative Region of China	Johns Hopkins University. 2019 Novel Coronavirus COVID-19 (2019-nCoV) Data Repository by Johns Hopkins CSSE. Baltimore, Maryland: Johns Hopkins University.
China, Hubei	Johns Hopkins University. 2019 Novel Coronavirus COVID-19 (2019-nCoV) Data Repository by Johns Hopkins CSSE. Baltimore, Maryland: Johns Hopkins University.
China, Hunan	Johns Hopkins University. 2019 Novel Coronavirus COVID-19 (2019-nCoV) Data Repository by Johns Hopkins CSSE. Baltimore, Maryland: Johns Hopkins University.
China, Inner Mongolia	Johns Hopkins University. 2019 Novel Coronavirus COVID-19 (2019-nCoV) Data Repository by Johns Hopkins CSSE. Baltimore, Maryland: Johns Hopkins University.
China, Jiangsu	Johns Hopkins University. 2019 Novel Coronavirus COVID-19 (2019-nCoV) Data Repository by Johns Hopkins CSSE. Baltimore, Maryland: Johns Hopkins University.

China, Jiangxi	Johns Hopkins University. 2019 Novel Coronavirus COVID-19 (2019-nCoV) Data Repository by Johns Hopkins CSSE. Baltimore, Maryland: Johns Hopkins University.
China, Jilin	Johns Hopkins University. 2019 Novel Coronavirus COVID-19 (2019-nCoV) Data Repository by Johns Hopkins CSSE. Baltimore, Maryland: Johns Hopkins University.
China, Liaoning	Johns Hopkins University. 2019 Novel Coronavirus COVID-19 (2019-nCoV) Data Repository by Johns Hopkins CSSE. Baltimore, Maryland: Johns Hopkins University.
China, Macao Special Administrative Region of China	Johns Hopkins University. 2019 Novel Coronavirus COVID-19 (2019-nCoV) Data Repository by Johns Hopkins CSSE. Baltimore, Maryland: Johns Hopkins University.
China, Ningxia	Johns Hopkins University. 2019 Novel Coronavirus COVID-19 (2019-nCoV) Data Repository by Johns Hopkins CSSE. Baltimore, Maryland: Johns Hopkins University.
China, Qinghai	Johns Hopkins University. 2019 Novel Coronavirus COVID-19 (2019-nCoV) Data Repository by Johns Hopkins CSSE. Baltimore, Maryland: Johns Hopkins University.
China, Shaanxi	Johns Hopkins University. 2019 Novel Coronavirus COVID-19 (2019-nCoV) Data Repository by Johns Hopkins CSSE. Baltimore, Maryland: Johns Hopkins University.
China, Shandong	Johns Hopkins University. 2019 Novel Coronavirus COVID-19 (2019-nCoV) Data Repository by Johns Hopkins CSSE. Baltimore, Maryland: Johns Hopkins University.
China, Shanghai	Johns Hopkins University. 2019 Novel Coronavirus COVID-19 (2019-nCoV) Data Repository by Johns Hopkins CSSE. Baltimore, Maryland: Johns Hopkins University.
China, Shanxi	Johns Hopkins University. 2019 Novel Coronavirus COVID-19 (2019-nCoV) Data Repository by Johns Hopkins CSSE. Baltimore, Maryland: Johns Hopkins University.
China, Sichuan	Johns Hopkins University. 2019 Novel Coronavirus COVID-19 (2019-nCoV) Data Repository by Johns Hopkins CSSE. Baltimore, Maryland: Johns Hopkins University.

China, Tianjin	Johns Hopkins University. 2019 Novel Coronavirus COVID-19 (2019-nCoV) Data Repository by Johns Hopkins CSSE. Baltimore, Maryland: Johns Hopkins University.
China, Tibet	Johns Hopkins University. 2019 Novel Coronavirus COVID-19 (2019-nCoV) Data Repository by Johns Hopkins CSSE. Baltimore, Maryland: Johns Hopkins University.
China, Xinjiang	Johns Hopkins University. 2019 Novel Coronavirus COVID-19 (2019-nCoV) Data Repository by Johns Hopkins CSSE. Baltimore, Maryland: Johns Hopkins University.
China, Yunnan	Johns Hopkins University. 2019 Novel Coronavirus COVID-19 (2019-nCoV) Data Repository by Johns Hopkins CSSE. Baltimore, Maryland: Johns Hopkins University.
China, Zhejiang	Johns Hopkins University. 2019 Novel Coronavirus COVID-19 (2019-nCoV) Data Repository by Johns Hopkins CSSE. Baltimore, Maryland: Johns Hopkins University.
Colombia	Johns Hopkins University. 2019 Novel Coronavirus COVID-19 (2019-nCoV) Data Repository by Johns Hopkins CSSE. Baltimore, Maryland: Johns Hopkins University.
Comoros	Johns Hopkins University. 2019 Novel Coronavirus COVID-19 (2019-nCoV) Data Repository by Johns Hopkins CSSE. Baltimore, Maryland: Johns Hopkins University.
Congo (Brazzaville)	Johns Hopkins University. 2019 Novel Coronavirus COVID-19 (2019-nCoV) Data Repository by Johns Hopkins CSSE. Baltimore, Maryland: Johns Hopkins University.
Costa Rica	Johns Hopkins University. 2019 Novel Coronavirus COVID-19 (2019-nCoV) Data Repository by Johns Hopkins CSSE. Baltimore, Maryland: Johns Hopkins University.
Côte d’Ivoire	Johns Hopkins University. 2019 Novel Coronavirus COVID-19 (2019-nCoV) Data Repository by Johns Hopkins CSSE. Baltimore, Maryland: Johns Hopkins University.
Croatia	Johns Hopkins University. 2019 Novel Coronavirus COVID-19 (2019-nCoV) Data Repository by Johns Hopkins CSSE. Baltimore, Maryland: Johns Hopkins University.

Cuba	Johns Hopkins University. 2019 Novel Coronavirus COVID-19 (2019-nCoV) Data Repository by Johns Hopkins CSSE. Baltimore, Maryland: Johns Hopkins University.
Cyprus	Johns Hopkins University. 2019 Novel Coronavirus COVID-19 (2019-nCoV) Data Repository by Johns Hopkins CSSE. Baltimore, Maryland: Johns Hopkins University.
Czechia	Johns Hopkins University. 2019 Novel Coronavirus COVID-19 (2019-nCoV) Data Repository by Johns Hopkins CSSE. Baltimore, Maryland: Johns Hopkins University.
Denmark	Johns Hopkins University. 2019 Novel Coronavirus COVID-19 (2019-nCoV) Data Repository by Johns Hopkins CSSE. Baltimore, Maryland: Johns Hopkins University.
Djibouti	Johns Hopkins University. 2019 Novel Coronavirus COVID-19 (2019-nCoV) Data Repository by Johns Hopkins CSSE. Baltimore, Maryland: Johns Hopkins University.
Dominica	Johns Hopkins University. 2019 Novel Coronavirus COVID-19 (2019-nCoV) Data Repository by Johns Hopkins CSSE. Baltimore, Maryland: Johns Hopkins University.
Dominican Republic	Johns Hopkins University. 2019 Novel Coronavirus COVID-19 (2019-nCoV) Data Repository by Johns Hopkins CSSE. Baltimore, Maryland: Johns Hopkins University.
DR Congo	Johns Hopkins University. 2019 Novel Coronavirus COVID-19 (2019-nCoV) Data Repository by Johns Hopkins CSSE. Baltimore, Maryland: Johns Hopkins University.
Ecuador	Johns Hopkins University. 2019 Novel Coronavirus COVID-19 (2019-nCoV) Data Repository by Johns Hopkins CSSE. Baltimore, Maryland: Johns Hopkins University.
Egypt	Johns Hopkins University. 2019 Novel Coronavirus COVID-19 (2019-nCoV) Data Repository by Johns Hopkins CSSE. Baltimore, Maryland: Johns Hopkins University.
El Salvador	Johns Hopkins University. 2019 Novel Coronavirus COVID-19 (2019-nCoV) Data Repository by Johns Hopkins CSSE. Baltimore, Maryland: Johns Hopkins University.

Equatorial Guinea	Johns Hopkins University. 2019 Novel Coronavirus COVID-19 (2019-nCoV) Data Repository by Johns Hopkins CSSE. Baltimore, Maryland: Johns Hopkins University.
Eritrea	Johns Hopkins University. 2019 Novel Coronavirus COVID-19 (2019-nCoV) Data Repository by Johns Hopkins CSSE. Baltimore, Maryland: Johns Hopkins University.
Estonia	Johns Hopkins University. 2019 Novel Coronavirus COVID-19 (2019-nCoV) Data Repository by Johns Hopkins CSSE. Baltimore, Maryland: Johns Hopkins University.
Eswatini	Johns Hopkins University. 2019 Novel Coronavirus COVID-19 (2019-nCoV) Data Repository by Johns Hopkins CSSE. Baltimore, Maryland: Johns Hopkins University.
Ethiopia	Johns Hopkins University. 2019 Novel Coronavirus COVID-19 (2019-nCoV) Data Repository by Johns Hopkins CSSE. Baltimore, Maryland: Johns Hopkins University.
Federated States of Micronesia	Johns Hopkins University. 2019 Novel Coronavirus COVID-19 (2019-nCoV) Data Repository by Johns Hopkins CSSE. Baltimore, Maryland: Johns Hopkins University.
Fiji	Johns Hopkins University. 2019 Novel Coronavirus COVID-19 (2019-nCoV) Data Repository by Johns Hopkins CSSE. Baltimore, Maryland: Johns Hopkins University.
Finland	Johns Hopkins University. 2019 Novel Coronavirus COVID-19 (2019-nCoV) Data Repository by Johns Hopkins CSSE. Baltimore, Maryland: Johns Hopkins University.
France	Etalab (France), Ministry of Health (France), Ministry of Health and Solidarity (DRESS) (France). France COVID-19 Epidemic Monitoring Dashboard.
Gabon	Johns Hopkins University. 2019 Novel Coronavirus COVID-19 (2019-nCoV) Data Repository by Johns Hopkins CSSE. Baltimore, Maryland: Johns Hopkins University.
Georgia	Johns Hopkins University. 2019 Novel Coronavirus COVID-19 (2019-nCoV) Data Repository by Johns Hopkins CSSE. Baltimore, Maryland: Johns Hopkins University.

Germany, Baden-Württemberg	Robert Koch Institute. Coronavirus Disease 2019 (COVID-19) Daily Situation Report - Robert Koch Institute. Berlin, Germany: Robert Koch Institute.
Germany, Baden-Württemberg	Wikipedia. COVID-19 pandemic in Germany. San Francisco, United States of America: Wikipedia.
Germany, Bavaria	Robert Koch Institute. Coronavirus Disease 2019 (COVID-19) Daily Situation Report - Robert Koch Institute. Berlin, Germany: Robert Koch Institute.
Germany, Bavaria	Wikipedia. COVID-19 pandemic in Germany. San Francisco, United States of America: Wikipedia.
Germany, Berlin	Robert Koch Institute. Coronavirus Disease 2019 (COVID-19) Daily Situation Report - Robert Koch Institute. Berlin, Germany: Robert Koch Institute.
Germany, Berlin	Wikipedia. COVID-19 pandemic in Germany. San Francisco, United States of America: Wikipedia.
Germany, Brandenburg	Robert Koch Institute. Coronavirus Disease 2019 (COVID-19) Daily Situation Report - Robert Koch Institute. Berlin, Germany: Robert Koch Institute.
Germany, Brandenburg	Wikipedia. COVID-19 pandemic in Germany. San Francisco, United States of America: Wikipedia.
Germany, Bremen	Robert Koch Institute. Coronavirus Disease 2019 (COVID-19) Daily Situation Report - Robert Koch Institute. Berlin, Germany: Robert Koch Institute.
Germany, Bremen	Wikipedia. COVID-19 pandemic in Germany. San Francisco, United States of America: Wikipedia.
Germany, Hamburg	Robert Koch Institute. Coronavirus Disease 2019 (COVID-19) Daily Situation Report - Robert Koch Institute. Berlin, Germany: Robert Koch Institute.
Germany, Hamburg	Wikipedia. COVID-19 pandemic in Germany. San Francisco, United States of America: Wikipedia.

Germany, Hesse	Robert Koch Institute. Coronavirus Disease 2019 (COVID-19) Daily Situation Report - Robert Koch Institute. Berlin, Germany: Robert Koch Institute.
Germany, Hesse	Wikipedia. COVID-19 pandemic in Germany. San Francisco, United States of America: Wikipedia.
Germany, Lower Saxony	Robert Koch Institute. Coronavirus Disease 2019 (COVID-19) Daily Situation Report - Robert Koch Institute. Berlin, Germany: Robert Koch Institute.
Germany, Lower Saxony	Wikipedia. COVID-19 pandemic in Germany. San Francisco, United States of America: Wikipedia.
Germany, Mecklenburg-Vorpommern	Robert Koch Institute. Coronavirus Disease 2019 (COVID-19) Daily Situation Report - Robert Koch Institute. Berlin, Germany: Robert Koch Institute.
Germany, Mecklenburg-Vorpommern	Wikipedia. COVID-19 pandemic in Germany. San Francisco, United States of America: Wikipedia.
Germany, North Rhine-Westphalia	Robert Koch Institute. Coronavirus Disease 2019 (COVID-19) Daily Situation Report - Robert Koch Institute. Berlin, Germany: Robert Koch Institute.
Germany, North Rhine-Westphalia	Wikipedia. COVID-19 pandemic in Germany. San Francisco, United States of America: Wikipedia.
Germany, Rhineland-Palatinate	Robert Koch Institute. Coronavirus Disease 2019 (COVID-19) Daily Situation Report - Robert Koch Institute. Berlin, Germany: Robert Koch Institute.
Germany, Rhineland-Palatinate	Wikipedia. COVID-19 pandemic in Germany. San Francisco, United States of America: Wikipedia.
Germany, Saarland	Robert Koch Institute. Coronavirus Disease 2019 (COVID-19) Daily Situation Report - Robert Koch Institute. Berlin, Germany: Robert Koch Institute.
Germany, Saarland	Wikipedia. COVID-19 pandemic in Germany. San Francisco, United States of America: Wikipedia.

Germany, Saxony	Robert Koch Institute. Coronavirus Disease 2019 (COVID-19) Daily Situation Report - Robert Koch Institute. Berlin, Germany: Robert Koch Institute.
Germany, Saxony	Wikipedia. COVID-19 pandemic in Germany. San Francisco, United States of America: Wikipedia.
Germany, Saxony-Anhalt	Robert Koch Institute. Coronavirus Disease 2019 (COVID-19) Daily Situation Report - Robert Koch Institute. Berlin, Germany: Robert Koch Institute.
Germany, Saxony-Anhalt	Wikipedia. COVID-19 pandemic in Germany. San Francisco, United States of America: Wikipedia.
Germany, Schleswig-Holstein	Robert Koch Institute. Coronavirus Disease 2019 (COVID-19) Daily Situation Report - Robert Koch Institute. Berlin, Germany: Robert Koch Institute.
Germany, Schleswig-Holstein	Wikipedia. COVID-19 pandemic in Germany. San Francisco, United States of America: Wikipedia.
Germany, Thuringia	Robert Koch Institute. Coronavirus Disease 2019 (COVID-19) Daily Situation Report - Robert Koch Institute. Berlin, Germany: Robert Koch Institute.
Germany, Thuringia	Wikipedia. COVID-19 pandemic in Germany. San Francisco, United States of America: Wikipedia.
Ghana	Johns Hopkins University. 2019 Novel Coronavirus COVID-19 (2019-nCoV) Data Repository by Johns Hopkins CSSE. Baltimore, Maryland: Johns Hopkins University.
Greece	Johns Hopkins University. 2019 Novel Coronavirus COVID-19 (2019-nCoV) Data Repository by Johns Hopkins CSSE. Baltimore, Maryland: Johns Hopkins University.
Greenland	Johns Hopkins University. 2019 Novel Coronavirus COVID-19 (2019-nCoV) Data Repository by Johns Hopkins CSSE. Baltimore, Maryland: Johns Hopkins University.
Grenada	Johns Hopkins University. 2019 Novel Coronavirus COVID-19 (2019-nCoV) Data Repository by Johns Hopkins CSSE. Baltimore, Maryland: Johns Hopkins University.

Guam	Johns Hopkins University. 2019 Novel Coronavirus COVID-19 (2019-nCoV) Data Repository by Johns Hopkins CSSE. Baltimore, Maryland: Johns Hopkins University.
Guatemala	Johns Hopkins University. 2019 Novel Coronavirus COVID-19 (2019-nCoV) Data Repository by Johns Hopkins CSSE. Baltimore, Maryland: Johns Hopkins University.
Guinea	Johns Hopkins University. 2019 Novel Coronavirus COVID-19 (2019-nCoV) Data Repository by Johns Hopkins CSSE. Baltimore, Maryland: Johns Hopkins University.
Guinea-Bissau	Johns Hopkins University. 2019 Novel Coronavirus COVID-19 (2019-nCoV) Data Repository by Johns Hopkins CSSE. Baltimore, Maryland: Johns Hopkins University.
Guyana	Johns Hopkins University. 2019 Novel Coronavirus COVID-19 (2019-nCoV) Data Repository by Johns Hopkins CSSE. Baltimore, Maryland: Johns Hopkins University.
Haiti	Johns Hopkins University. 2019 Novel Coronavirus COVID-19 (2019-nCoV) Data Repository by Johns Hopkins CSSE. Baltimore, Maryland: Johns Hopkins University.
Honduras	Johns Hopkins University. 2019 Novel Coronavirus COVID-19 (2019-nCoV) Data Repository by Johns Hopkins CSSE. Baltimore, Maryland: Johns Hopkins University.
Hungary	Johns Hopkins University. 2019 Novel Coronavirus COVID-19 (2019-nCoV) Data Repository by Johns Hopkins CSSE. Baltimore, Maryland: Johns Hopkins University.
Iceland	Johns Hopkins University. 2019 Novel Coronavirus COVID-19 (2019-nCoV) Data Repository by Johns Hopkins CSSE. Baltimore, Maryland: Johns Hopkins University.
India, Andhra Pradesh	COVID-19 India. India COVID-19 Crowdsourced Patient Database: National Level Time Series, State-Wise Stats and Test Counts. India: COVID-19 India.
India, Andhra Pradesh	COVID-19 India. India COVID-19 Crowdsourced Patient Database: State Level Daily Changes. India: COVID-19 India.

India, Arunachal Pradesh	COVID-19 India. India COVID-19 Crowdsourced Patient Database: National Level Time Series, State-Wise Stats and Test Counts. India: COVID-19 India.
India, Arunachal Pradesh	COVID-19 India. India COVID-19 Crowdsourced Patient Database: State Level Daily Changes. India: COVID-19 India.
India, Assam	COVID-19 India. India COVID-19 Crowdsourced Patient Database: National Level Time Series, State-Wise Stats and Test Counts. India: COVID-19 India.
India, Assam	COVID-19 India. India COVID-19 Crowdsourced Patient Database: State Level Daily Changes. India: COVID-19 India.
India, Bihar	COVID-19 India. India COVID-19 Crowdsourced Patient Database: National Level Time Series, State-Wise Stats and Test Counts. India: COVID-19 India.
India, Bihar	COVID-19 India. India COVID-19 Crowdsourced Patient Database: State Level Daily Changes. India: COVID-19 India.
India, Chhattisgarh	COVID-19 India. India COVID-19 Crowdsourced Patient Database: National Level Time Series, State-Wise Stats and Test Counts. India: COVID-19 India.
India, Chhattisgarh	COVID-19 India. India COVID-19 Crowdsourced Patient Database: State Level Daily Changes. India: COVID-19 India.
India, Dadra and Nagar Haveli and Daman and Diu	COVID-19 India. India COVID-19 Crowdsourced Patient Database: National Level Time Series, State-Wise Stats and Test Counts. India: COVID-19 India.
India, Dadra and Nagar Haveli and Daman and Diu	COVID-19 India. India COVID-19 Crowdsourced Patient Database: State Level Daily Changes. India: COVID-19 India.
India, Delhi	COVID-19 India. India COVID-19 Crowdsourced Patient Database: National Level Time Series, State-Wise Stats and Test Counts. India: COVID-19 India.
India, Delhi	COVID-19 India. India COVID-19 Crowdsourced Patient Database: State Level Daily Changes. India: COVID-19 India.

India, Goa	COVID-19 India. India COVID-19 Crowdsourced Patient Database: National Level Time Series, State-Wise Stats and Test Counts. India: COVID-19 India.
India, Goa	COVID-19 India. India COVID-19 Crowdsourced Patient Database: State Level Daily Changes. India: COVID-19 India.
India, Gujarat	COVID-19 India. India COVID-19 Crowdsourced Patient Database: National Level Time Series, State-Wise Stats and Test Counts. India: COVID-19 India.
India, Gujarat	COVID-19 India. India COVID-19 Crowdsourced Patient Database: State Level Daily Changes. India: COVID-19 India.
India, Haryana	COVID-19 India. India COVID-19 Crowdsourced Patient Database: National Level Time Series, State-Wise Stats and Test Counts. India: COVID-19 India.
India, Haryana	COVID-19 India. India COVID-19 Crowdsourced Patient Database: State Level Daily Changes. India: COVID-19 India.
India, Himachal Pradesh	COVID-19 India. India COVID-19 Crowdsourced Patient Database: National Level Time Series, State-Wise Stats and Test Counts. India: COVID-19 India.
India, Himachal Pradesh	COVID-19 India. India COVID-19 Crowdsourced Patient Database: State Level Daily Changes. India: COVID-19 India.
India, Jammu & Kashmir and Ladakh	COVID-19 India. India COVID-19 Crowdsourced Patient Database: National Level Time Series, State-Wise Stats and Test Counts. India: COVID-19 India.
India, Jammu & Kashmir and Ladakh	COVID-19 India. India COVID-19 Crowdsourced Patient Database: State Level Daily Changes. India: COVID-19 India.
India, Jharkhand	COVID-19 India. India COVID-19 Crowdsourced Patient Database: National Level Time Series, State-Wise Stats and Test Counts. India: COVID-19 India.
India, Jharkhand	COVID-19 India. India COVID-19 Crowdsourced Patient Database: State Level Daily Changes. India: COVID-19 India.

India, Karnataka	COVID-19 India. India COVID-19 Crowdsourced Patient Database: National Level Time Series, State-Wise Stats and Test Counts. India: COVID-19 India.
India, Karnataka	COVID-19 India. India COVID-19 Crowdsourced Patient Database: State Level Daily Changes. India: COVID-19 India.
India, Kerala	COVID-19 India. India COVID-19 Crowdsourced Patient Database: National Level Time Series, State-Wise Stats and Test Counts. India: COVID-19 India.
India, Kerala	COVID-19 India. India COVID-19 Crowdsourced Patient Database: State Level Daily Changes. India: COVID-19 India.
India, Madhya Pradesh	COVID-19 India. India COVID-19 Crowdsourced Patient Database: National Level Time Series, State-Wise Stats and Test Counts. India: COVID-19 India.
India, Madhya Pradesh	COVID-19 India. India COVID-19 Crowdsourced Patient Database: State Level Daily Changes. India: COVID-19 India.
India, Maharashtra	COVID-19 India. India COVID-19 Crowdsourced Patient Database: National Level Time Series, State-Wise Stats and Test Counts. India: COVID-19 India.
India, Maharashtra	COVID-19 India. India COVID-19 Crowdsourced Patient Database: State Level Daily Changes. India: COVID-19 India.
India, Manipur	COVID-19 India. India COVID-19 Crowdsourced Patient Database: National Level Time Series, State-Wise Stats and Test Counts. India: COVID-19 India.
India, Manipur	COVID-19 India. India COVID-19 Crowdsourced Patient Database: State Level Daily Changes. India: COVID-19 India.
India, Meghalaya	COVID-19 India. India COVID-19 Crowdsourced Patient Database: National Level Time Series, State-Wise Stats and Test Counts. India: COVID-19 India.
India, Meghalaya	COVID-19 India. India COVID-19 Crowdsourced Patient Database: State Level Daily Changes. India: COVID-19 India.

India, Mizoram	COVID-19 India. India COVID-19 Crowdsourced Patient Database: National Level Time Series, State-Wise Stats and Test Counts. India: COVID-19 India.
India, Mizoram	COVID-19 India. India COVID-19 Crowdsourced Patient Database: State Level Daily Changes. India: COVID-19 India.
India, Nagaland	COVID-19 India. India COVID-19 Crowdsourced Patient Database: National Level Time Series, State-Wise Stats and Test Counts. India: COVID-19 India.
India, Nagaland	COVID-19 India. India COVID-19 Crowdsourced Patient Database: State Level Daily Changes. India: COVID-19 India.
India, Odisha	COVID-19 India. India COVID-19 Crowdsourced Patient Database: National Level Time Series, State-Wise Stats and Test Counts. India: COVID-19 India.
India, Odisha	COVID-19 India. India COVID-19 Crowdsourced Patient Database: State Level Daily Changes. India: COVID-19 India.
India, Punjab	COVID-19 India. India COVID-19 Crowdsourced Patient Database: National Level Time Series, State-Wise Stats and Test Counts. India: COVID-19 India.
India, Punjab	COVID-19 India. India COVID-19 Crowdsourced Patient Database: State Level Daily Changes. India: COVID-19 India.
India, Rajasthan	COVID-19 India. India COVID-19 Crowdsourced Patient Database: National Level Time Series, State-Wise Stats and Test Counts. India: COVID-19 India.
India, Rajasthan	COVID-19 India. India COVID-19 Crowdsourced Patient Database: State Level Daily Changes. India: COVID-19 India.
India, Sikkim	COVID-19 India. India COVID-19 Crowdsourced Patient Database: National Level Time Series, State-Wise Stats and Test Counts. India: COVID-19 India.
India, Sikkim	COVID-19 India. India COVID-19 Crowdsourced Patient Database: State Level Daily Changes. India: COVID-19 India.

India, Tamil Nadu	COVID-19 India. India COVID-19 Crowdsourced Patient Database: National Level Time Series, State-Wise Stats and Test Counts. India: COVID-19 India.
India, Tamil Nadu	COVID-19 India. India COVID-19 Crowdsourced Patient Database: State Level Daily Changes. India: COVID-19 India.
India, Telangana	COVID-19 India. India COVID-19 Crowdsourced Patient Database: National Level Time Series, State-Wise Stats and Test Counts. India: COVID-19 India.
India, Telangana	COVID-19 India. India COVID-19 Crowdsourced Patient Database: State Level Daily Changes. India: COVID-19 India.
India, Tripura	COVID-19 India. India COVID-19 Crowdsourced Patient Database: National Level Time Series, State-Wise Stats and Test Counts. India: COVID-19 India.
India, Tripura	COVID-19 India. India COVID-19 Crowdsourced Patient Database: State Level Daily Changes. India: COVID-19 India.
India, Uttar Pradesh	COVID-19 India. India COVID-19 Crowdsourced Patient Database: National Level Time Series, State-Wise Stats and Test Counts. India: COVID-19 India.
India, Uttar Pradesh	COVID-19 India. India COVID-19 Crowdsourced Patient Database: State Level Daily Changes. India: COVID-19 India.
India, Uttarakhand	COVID-19 India. India COVID-19 Crowdsourced Patient Database: National Level Time Series, State-Wise Stats and Test Counts. India: COVID-19 India.
India, Uttarakhand	COVID-19 India. India COVID-19 Crowdsourced Patient Database: State Level Daily Changes. India: COVID-19 India.
India, West Bengal	COVID-19 India. India COVID-19 Crowdsourced Patient Database: National Level Time Series, State-Wise Stats and Test Counts. India: COVID-19 India.
India, West Bengal	COVID-19 India. India COVID-19 Crowdsourced Patient Database: State Level Daily Changes. India: COVID-19 India.

Indonesia	Johns Hopkins University. 2019 Novel Coronavirus COVID-19 (2019-nCoV) Data Repository by Johns Hopkins CSSE. Baltimore, Maryland: Johns Hopkins University.
Iran	Johns Hopkins University. 2019 Novel Coronavirus COVID-19 (2019-nCoV) Data Repository by Johns Hopkins CSSE. Baltimore, Maryland: Johns Hopkins University.
Iraq	Johns Hopkins University. 2019 Novel Coronavirus COVID-19 (2019-nCoV) Data Repository by Johns Hopkins CSSE. Baltimore, Maryland: Johns Hopkins University.
Ireland	Johns Hopkins University. 2019 Novel Coronavirus COVID-19 (2019-nCoV) Data Repository by Johns Hopkins CSSE. Baltimore, Maryland: Johns Hopkins University.
Israel	Johns Hopkins University. 2019 Novel Coronavirus COVID-19 (2019-nCoV) Data Repository by Johns Hopkins CSSE. Baltimore, Maryland: Johns Hopkins University.
Italy, Abruzzo	Department of Civil Protection (Italy). Italy COVID-19 Situation Monitoring - Department of Civil Protection. Rome, Italy: Department of Civil Protection (Italy).
Italy, Basilicata	Department of Civil Protection (Italy). Italy COVID-19 Situation Monitoring - Department of Civil Protection. Rome, Italy: Department of Civil Protection (Italy).
Italy, Calabria	Department of Civil Protection (Italy). Italy COVID-19 Situation Monitoring - Department of Civil Protection. Rome, Italy: Department of Civil Protection (Italy).
Italy, Campania	Department of Civil Protection (Italy). Italy COVID-19 Situation Monitoring - Department of Civil Protection. Rome, Italy: Department of Civil Protection (Italy).
Italy, Emilia-Romagna	Department of Civil Protection (Italy). Italy COVID-19 Situation Monitoring - Department of Civil Protection. Rome, Italy: Department of Civil Protection (Italy).
Italy, Friuli-Venezia Giulia	Department of Civil Protection (Italy). Italy COVID-19 Situation Monitoring - Department of Civil Protection. Rome,

	Italy: Department of Civil Protection (Italy).
Italy, Lazio	Department of Civil Protection (Italy). Italy COVID-19 Situation Monitoring - Department of Civil Protection. Rome, Italy: Department of Civil Protection (Italy).
Italy, Liguria	Department of Civil Protection (Italy). Italy COVID-19 Situation Monitoring - Department of Civil Protection. Rome, Italy: Department of Civil Protection (Italy).
Italy, Lombardia	Department of Civil Protection (Italy). Italy COVID-19 Situation Monitoring - Department of Civil Protection. Rome, Italy: Department of Civil Protection (Italy).
Italy, Marche	Department of Civil Protection (Italy). Italy COVID-19 Situation Monitoring - Department of Civil Protection. Rome, Italy: Department of Civil Protection (Italy).
Italy, Molise	Department of Civil Protection (Italy). Italy COVID-19 Situation Monitoring - Department of Civil Protection. Rome, Italy: Department of Civil Protection (Italy).
Italy, Piemonte	Department of Civil Protection (Italy). Italy COVID-19 Situation Monitoring - Department of Civil Protection. Rome, Italy: Department of Civil Protection (Italy).
Italy, Prov. autonoma di Bolzano	Department of Civil Protection (Italy). Italy COVID-19 Situation Monitoring - Department of Civil Protection. Rome, Italy: Department of Civil Protection (Italy).
Italy, Prov. autonoma di Trento	Department of Civil Protection (Italy). Italy COVID-19 Situation Monitoring - Department of Civil Protection. Rome, Italy: Department of Civil Protection (Italy).
Italy, Puglia	Department of Civil Protection (Italy). Italy COVID-19 Situation Monitoring - Department of Civil Protection. Rome, Italy: Department of Civil Protection (Italy).
Italy, Sardegna	Department of Civil Protection (Italy). Italy COVID-19 Situation Monitoring - Department of Civil Protection. Rome, Italy: Department of Civil Protection (Italy).

Italy, Sicilia	Department of Civil Protection (Italy). Italy COVID-19 Situation Monitoring - Department of Civil Protection. Rome, Italy: Department of Civil Protection (Italy).
Italy, Toscana	Department of Civil Protection (Italy). Italy COVID-19 Situation Monitoring - Department of Civil Protection. Rome, Italy: Department of Civil Protection (Italy).
Italy, Umbria	Department of Civil Protection (Italy). Italy COVID-19 Situation Monitoring - Department of Civil Protection. Rome, Italy: Department of Civil Protection (Italy).
Italy, Valle d'Aosta	Department of Civil Protection (Italy). Italy COVID-19 Situation Monitoring - Department of Civil Protection. Rome, Italy: Department of Civil Protection (Italy).
Italy, Veneto	Department of Civil Protection (Italy). Italy COVID-19 Situation Monitoring - Department of Civil Protection. Rome, Italy: Department of Civil Protection (Italy).
Jamaica	Johns Hopkins University. 2019 Novel Coronavirus COVID-19 (2019-nCoV) Data Repository by Johns Hopkins CSSE. Baltimore, Maryland: Johns Hopkins University.
Japan	Ministry of Health, Labour and Welfare (Japan). Japan Coronavirus Disease (COVID-19) Situation Report.
Jordan	Johns Hopkins University. 2019 Novel Coronavirus COVID-19 (2019-nCoV) Data Repository by Johns Hopkins CSSE. Baltimore, Maryland: Johns Hopkins University.
Kazakhstan	*
Kenya	Johns Hopkins University. 2019 Novel Coronavirus COVID-19 (2019-nCoV) Data Repository by Johns Hopkins CSSE. Baltimore, Maryland: Johns Hopkins University.
Kiribati	Johns Hopkins University. 2019 Novel Coronavirus COVID-19 (2019-nCoV) Data Repository by Johns Hopkins CSSE. Baltimore, Maryland: Johns Hopkins University.
Kuwait	Johns Hopkins University. 2019 Novel Coronavirus COVID-19 (2019-nCoV) Data Repository by Johns Hopkins CSSE. Baltimore, Maryland: Johns Hopkins University.

Kyrgyzstan	Johns Hopkins University. 2019 Novel Coronavirus COVID-19 (2019-nCoV) Data Repository by Johns Hopkins CSSE. Baltimore, Maryland: Johns Hopkins University.
Laos	Johns Hopkins University. 2019 Novel Coronavirus COVID-19 (2019-nCoV) Data Repository by Johns Hopkins CSSE. Baltimore, Maryland: Johns Hopkins University.
Latvia	Johns Hopkins University. 2019 Novel Coronavirus COVID-19 (2019-nCoV) Data Repository by Johns Hopkins CSSE. Baltimore, Maryland: Johns Hopkins University.
Lebanon	Johns Hopkins University. 2019 Novel Coronavirus COVID-19 (2019-nCoV) Data Repository by Johns Hopkins CSSE. Baltimore, Maryland: Johns Hopkins University.
Lesotho	Johns Hopkins University. 2019 Novel Coronavirus COVID-19 (2019-nCoV) Data Repository by Johns Hopkins CSSE. Baltimore, Maryland: Johns Hopkins University.
Liberia	Johns Hopkins University. 2019 Novel Coronavirus COVID-19 (2019-nCoV) Data Repository by Johns Hopkins CSSE. Baltimore, Maryland: Johns Hopkins University.
Libya	Johns Hopkins University. 2019 Novel Coronavirus COVID-19 (2019-nCoV) Data Repository by Johns Hopkins CSSE. Baltimore, Maryland: Johns Hopkins University.
Lithuania	Johns Hopkins University. 2019 Novel Coronavirus COVID-19 (2019-nCoV) Data Repository by Johns Hopkins CSSE. Baltimore, Maryland: Johns Hopkins University.
Luxembourg	Johns Hopkins University. 2019 Novel Coronavirus COVID-19 (2019-nCoV) Data Repository by Johns Hopkins CSSE. Baltimore, Maryland: Johns Hopkins University.
Madagascar	Johns Hopkins University. 2019 Novel Coronavirus COVID-19 (2019-nCoV) Data Repository by Johns Hopkins CSSE. Baltimore, Maryland: Johns Hopkins University.
Malawi	Johns Hopkins University. 2019 Novel Coronavirus COVID-19 (2019-nCoV) Data Repository by Johns Hopkins CSSE. Baltimore, Maryland: Johns Hopkins University.

Malaysia	Johns Hopkins University. 2019 Novel Coronavirus COVID-19 (2019-nCoV) Data Repository by Johns Hopkins CSSE. Baltimore, Maryland: Johns Hopkins University.
Maldives	Johns Hopkins University. 2019 Novel Coronavirus COVID-19 (2019-nCoV) Data Repository by Johns Hopkins CSSE. Baltimore, Maryland: Johns Hopkins University.
Mali	Johns Hopkins University. 2019 Novel Coronavirus COVID-19 (2019-nCoV) Data Repository by Johns Hopkins CSSE. Baltimore, Maryland: Johns Hopkins University.
Malta	Johns Hopkins University. 2019 Novel Coronavirus COVID-19 (2019-nCoV) Data Repository by Johns Hopkins CSSE. Baltimore, Maryland: Johns Hopkins University.
Marshall Islands	Johns Hopkins University. 2019 Novel Coronavirus COVID-19 (2019-nCoV) Data Repository by Johns Hopkins CSSE. Baltimore, Maryland: Johns Hopkins University.
Mauritania	Johns Hopkins University. 2019 Novel Coronavirus COVID-19 (2019-nCoV) Data Repository by Johns Hopkins CSSE. Baltimore, Maryland: Johns Hopkins University.
Mauritius	Johns Hopkins University. 2019 Novel Coronavirus COVID-19 (2019-nCoV) Data Repository by Johns Hopkins CSSE. Baltimore, Maryland: Johns Hopkins University.
Mexico, Aguascalientes	Directorate General of Epidemiology, Secretariat of Health (Mexico), National Institute of Statistics and Geography (INEGI) (Mexico). Mexico COVID-19 Deaths 2020.
Mexico, Baja California	Directorate General of Epidemiology, Secretariat of Health (Mexico), National Institute of Statistics and Geography (INEGI) (Mexico). Mexico COVID-19 Deaths 2020.
Mexico, Baja California Sur	Directorate General of Epidemiology, Secretariat of Health (Mexico), National Institute of Statistics and Geography (INEGI) (Mexico). Mexico COVID-19 Deaths 2020.
Mexico, Campeche	Directorate General of Epidemiology, Secretariat of Health (Mexico), National Institute of Statistics and Geography (INEGI) (Mexico). Mexico COVID-19 Deaths 2020.
Mexico, Chiapas	Directorate General of Epidemiology, Secretariat of Health (Mexico), National Institute of Statistics and Geography (INEGI) (Mexico). Mexico COVID-19 Deaths 2020.
Mexico, Chihuahua	Directorate General of Epidemiology, Secretariat of Health (Mexico), National Institute of Statistics and Geography (INEGI) (Mexico). Mexico COVID-19 Deaths 2020.

Mexico, Coahuila	Directorate General of Epidemiology, Secretariat of Health (Mexico), National Institute of Statistics and Geography (INEGI) (Mexico). Mexico COVID-19 Deaths 2020.
Mexico, Colima	Directorate General of Epidemiology, Secretariat of Health (Mexico), National Institute of Statistics and Geography (INEGI) (Mexico). Mexico COVID-19 Deaths 2020.
Mexico, Durango	Directorate General of Epidemiology, Secretariat of Health (Mexico), National Institute of Statistics and Geography (INEGI) (Mexico). Mexico COVID-19 Deaths 2020.
Mexico, Guanajuato	Directorate General of Epidemiology, Secretariat of Health (Mexico), National Institute of Statistics and Geography (INEGI) (Mexico). Mexico COVID-19 Deaths 2020.
Mexico, Guerrero	Directorate General of Epidemiology, Secretariat of Health (Mexico), National Institute of Statistics and Geography (INEGI) (Mexico). Mexico COVID-19 Deaths 2020.
Mexico, Hidalgo	Directorate General of Epidemiology, Secretariat of Health (Mexico), National Institute of Statistics and Geography (INEGI) (Mexico). Mexico COVID-19 Deaths 2020.
Mexico, Jalisco	Directorate General of Epidemiology, Secretariat of Health (Mexico), National Institute of Statistics and Geography (INEGI) (Mexico). Mexico COVID-19 Deaths 2020.
Mexico, México	Directorate General of Epidemiology, Secretariat of Health (Mexico), National Institute of Statistics and Geography (INEGI) (Mexico). Mexico COVID-19 Deaths 2020.
Mexico, Mexico City	Directorate General of Epidemiology, Secretariat of Health (Mexico), National Institute of Statistics and Geography (INEGI) (Mexico). Mexico COVID-19 Deaths 2020.
Mexico, Michoacán de Ocampo	Directorate General of Epidemiology, Secretariat of Health (Mexico), National Institute of Statistics and Geography (INEGI) (Mexico). Mexico COVID-19 Deaths 2020.
Mexico, Morelos	Directorate General of Epidemiology, Secretariat of Health (Mexico), National Institute of Statistics and Geography (INEGI) (Mexico). Mexico COVID-19 Deaths 2020.
Mexico, Nayarit	Directorate General of Epidemiology, Secretariat of Health (Mexico), National Institute of Statistics and Geography (INEGI) (Mexico). Mexico COVID-19 Deaths 2020.
Mexico, Nuevo León	Directorate General of Epidemiology, Secretariat of Health (Mexico), National Institute of Statistics and Geography (INEGI) (Mexico). Mexico COVID-19 Deaths 2020.
Mexico, Oaxaca	Directorate General of Epidemiology, Secretariat of Health (Mexico), National Institute of Statistics and Geography (INEGI) (Mexico). Mexico COVID-19 Deaths 2020.
Mexico, Puebla	Directorate General of Epidemiology, Secretariat of Health (Mexico), National Institute of Statistics and Geography (INEGI) (Mexico). Mexico COVID-19 Deaths 2020.
Mexico, Querétaro	Directorate General of Epidemiology, Secretariat of Health (Mexico), National Institute of Statistics and Geography (INEGI) (Mexico). Mexico COVID-19 Deaths 2020.
Mexico, Quintana Roo	Directorate General of Epidemiology, Secretariat of Health (Mexico), National Institute of Statistics and Geography (INEGI) (Mexico). Mexico COVID-19 Deaths 2020.
Mexico, San Luis Potosí	Directorate General of Epidemiology, Secretariat of Health (Mexico), National Institute of Statistics and Geography (INEGI) (Mexico). Mexico COVID-19 Deaths 2020.

Mexico, Sinaloa	Directorate General of Epidemiology, Secretariat of Health (Mexico), National Institute of Statistics and Geography (INEGI) (Mexico). Mexico COVID-19 Deaths 2020.
Mexico, Sonora	Directorate General of Epidemiology, Secretariat of Health (Mexico), National Institute of Statistics and Geography (INEGI) (Mexico). Mexico COVID-19 Deaths 2020.
Mexico, Tabasco	Directorate General of Epidemiology, Secretariat of Health (Mexico), National Institute of Statistics and Geography (INEGI) (Mexico). Mexico COVID-19 Deaths 2020.
Mexico, Tamaulipas	Directorate General of Epidemiology, Secretariat of Health (Mexico), National Institute of Statistics and Geography (INEGI) (Mexico). Mexico COVID-19 Deaths 2020.
Mexico, Tlaxcala	Directorate General of Epidemiology, Secretariat of Health (Mexico), National Institute of Statistics and Geography (INEGI) (Mexico). Mexico COVID-19 Deaths 2020.
Mexico, Veracruz de Ignacio de la Llave	Directorate General of Epidemiology, Secretariat of Health (Mexico), National Institute of Statistics and Geography (INEGI) (Mexico). Mexico COVID-19 Deaths 2020.
Mexico, Yucatán	Directorate General of Epidemiology, Secretariat of Health (Mexico), National Institute of Statistics and Geography (INEGI) (Mexico). Mexico COVID-19 Deaths 2020.
Mexico, Zacatecas	Directorate General of Epidemiology, Secretariat of Health (Mexico), National Institute of Statistics and Geography (INEGI) (Mexico). Mexico COVID-19 Deaths 2020.
Moldova	Johns Hopkins University. 2019 Novel Coronavirus COVID-19 (2019-nCoV) Data Repository by Johns Hopkins CSSE. Baltimore, Maryland: Johns Hopkins University.
Monaco	Johns Hopkins University. 2019 Novel Coronavirus COVID-19 (2019-nCoV) Data Repository by Johns Hopkins CSSE. Baltimore, Maryland: Johns Hopkins University.
Mongolia	Johns Hopkins University. 2019 Novel Coronavirus COVID-19 (2019-nCoV) Data Repository by Johns Hopkins CSSE. Baltimore, Maryland: Johns Hopkins University.
Montenegro	Johns Hopkins University. 2019 Novel Coronavirus COVID-19 (2019-nCoV) Data Repository by Johns Hopkins CSSE. Baltimore, Maryland: Johns Hopkins University.
Morocco	Johns Hopkins University. 2019 Novel Coronavirus COVID-19 (2019-nCoV) Data Repository by Johns Hopkins CSSE. Baltimore, Maryland: Johns Hopkins University.
Mozambique	Johns Hopkins University. 2019 Novel Coronavirus COVID-19 (2019-nCoV) Data Repository by Johns Hopkins CSSE. Baltimore, Maryland: Johns Hopkins University.

Myanmar	Johns Hopkins University. 2019 Novel Coronavirus COVID-19 (2019-nCoV) Data Repository by Johns Hopkins CSSE. Baltimore, Maryland: Johns Hopkins University.
Namibia	Johns Hopkins University. 2019 Novel Coronavirus COVID-19 (2019-nCoV) Data Repository by Johns Hopkins CSSE. Baltimore, Maryland: Johns Hopkins University.
Nepal	Johns Hopkins University. 2019 Novel Coronavirus COVID-19 (2019-nCoV) Data Repository by Johns Hopkins CSSE. Baltimore, Maryland: Johns Hopkins University.
Netherlands	Johns Hopkins University. 2019 Novel Coronavirus COVID-19 (2019-nCoV) Data Repository by Johns Hopkins CSSE. Baltimore, Maryland: Johns Hopkins University.
New Zealand	Johns Hopkins University. 2019 Novel Coronavirus COVID-19 (2019-nCoV) Data Repository by Johns Hopkins CSSE. Baltimore, Maryland: Johns Hopkins University.
Nicaragua	Johns Hopkins University. 2019 Novel Coronavirus COVID-19 (2019-nCoV) Data Repository by Johns Hopkins CSSE. Baltimore, Maryland: Johns Hopkins University.
Niger	Johns Hopkins University. 2019 Novel Coronavirus COVID-19 (2019-nCoV) Data Repository by Johns Hopkins CSSE. Baltimore, Maryland: Johns Hopkins University.
Nigeria	Johns Hopkins University. 2019 Novel Coronavirus COVID-19 (2019-nCoV) Data Repository by Johns Hopkins CSSE. Baltimore, Maryland: Johns Hopkins University.
North Macedonia	Johns Hopkins University. 2019 Novel Coronavirus COVID-19 (2019-nCoV) Data Repository by Johns Hopkins CSSE. Baltimore, Maryland: Johns Hopkins University.
Northern Mariana Islands	Johns Hopkins University. 2019 Novel Coronavirus COVID-19 (2019-nCoV) Data Repository by Johns Hopkins CSSE. Baltimore, Maryland: Johns Hopkins University.
Norway	Johns Hopkins University. 2019 Novel Coronavirus COVID-19 (2019-nCoV) Data Repository by Johns Hopkins CSSE. Baltimore, Maryland: Johns Hopkins University.

Oman	Johns Hopkins University. 2019 Novel Coronavirus COVID-19 (2019-nCoV) Data Repository by Johns Hopkins CSSE. Baltimore, Maryland: Johns Hopkins University.
Pakistan, Azad Jammu & Kashmir	Ministry of National Health Services, Regulations & Coordination (Pakistan). Pakistan - Azad Jammu and Kashmir COVID-19 Statistics. Islamabad, Pakistan: Government of Pakistan.
Pakistan, Azad Jammu & Kashmir	Ministry of National Health Services, Regulations & Coordination (Pakistan). Pakistan COVID-19 Dashboard.
Pakistan, Balochistan	Ministry of National Health Services, Regulations & Coordination (Pakistan). Pakistan - Balochistan COVID-19 Statistics. Islamabad, Pakistan: Government of Pakistan.
Pakistan, Balochistan	Ministry of National Health Services, Regulations & Coordination (Pakistan). Pakistan COVID-19 Dashboard.
Pakistan, Gilgit-Baltistan	Ministry of National Health Services, Regulations & Coordination (Pakistan). Pakistan - Gilgit-Baltistan COVID-19 Statistics. Islamabad, Pakistan: Government of Pakistan.
Pakistan, Gilgit-Baltistan	Ministry of National Health Services, Regulations & Coordination (Pakistan). Pakistan COVID-19 Dashboard.
Pakistan, Islamabad Capital Territory	Ministry of National Health Services, Regulations & Coordination (Pakistan). Pakistan - Islāmābād COVID-19 Statistics. Islāmābād, Pakistan: Government of Pakistan.
Pakistan, Islamabad Capital Territory	Ministry of National Health Services, Regulations & Coordination (Pakistan). Pakistan COVID-19 Dashboard.
Pakistan, Khyber Pakhtunkhwa	Ministry of National Health Services, Regulations & Coordination (Pakistan). Pakistan - Khyber Pakhtunkhwa COVID-19 Statistics 2020.
Pakistan, Khyber Pakhtunkhwa	Ministry of National Health Services, Regulations & Coordination (Pakistan). Pakistan COVID-19 Dashboard.
Pakistan, Punjab	Ministry of National Health Services, Regulations & Coordination (Pakistan). Pakistan - Punjab COVID-19 Statistics. Islamabad, Pakistan: Government of Pakistan.

Pakistan, Punjab	Ministry of National Health Services, Regulations & Coordination (Pakistan). Pakistan COVID-19 Dashboard.
Pakistan, Sindh	Wikipedia. COVID-19 pandemic in Pakistan. San Francisco, United States of America: Wikipedia. Retrieved on April 29, 2021 fromÂ https://en.wikipedia.org/wiki/COVID-19_pandemic_in_Pakistan
Pakistan, Sindh	Ministry of National Health Services, Regulations & Coordination (Pakistan). Pakistan - Sindh COVID-19 Statistics. Islamabad, Pakistan: Government of Pakistan.
Pakistan, Sindh	Ministry of National Health Services, Regulations & Coordination (Pakistan). Pakistan COVID-19 Dashboard.
Palau	Johns Hopkins University. 2019 Novel Coronavirus COVID-19 (2019-nCoV) Data Repository by Johns Hopkins CSSE. Baltimore, Maryland: Johns Hopkins University.
Palestine	Johns Hopkins University. 2019 Novel Coronavirus COVID-19 (2019-nCoV) Data Repository by Johns Hopkins CSSE. Baltimore, Maryland: Johns Hopkins University.
Panama	Johns Hopkins University. 2019 Novel Coronavirus COVID-19 (2019-nCoV) Data Repository by Johns Hopkins CSSE. Baltimore, Maryland: Johns Hopkins University.
Papua New Guinea	Johns Hopkins University. 2019 Novel Coronavirus COVID-19 (2019-nCoV) Data Repository by Johns Hopkins CSSE. Baltimore, Maryland: Johns Hopkins University.
Paraguay	Johns Hopkins University. 2019 Novel Coronavirus COVID-19 (2019-nCoV) Data Repository by Johns Hopkins CSSE. Baltimore, Maryland: Johns Hopkins University.
Peru	Ministry of Health (Peru), National Center for Epidemiology, Prevention and Disease Control (Peru). Peru Deaths by COVID-19.
Philippines	Department of Health (Philippines). Philippines Department of Health COVID-19 Tracker. Manila, Philippines: Department of Health (Philippines).

Poland	Johns Hopkins University. 2019 Novel Coronavirus COVID-19 (2019-nCoV) Data Repository by Johns Hopkins CSSE. Baltimore, Maryland: Johns Hopkins University.
Portugal	Johns Hopkins University. 2019 Novel Coronavirus COVID-19 (2019-nCoV) Data Repository by Johns Hopkins CSSE. Baltimore, Maryland: Johns Hopkins University.
Puerto Rico	Johns Hopkins University. 2019 Novel Coronavirus COVID-19 (2019-nCoV) Data Repository by Johns Hopkins CSSE. Baltimore, Maryland: Johns Hopkins University.
Qatar	Johns Hopkins University. 2019 Novel Coronavirus COVID-19 (2019-nCoV) Data Repository by Johns Hopkins CSSE. Baltimore, Maryland: Johns Hopkins University.
Romania	Johns Hopkins University. 2019 Novel Coronavirus COVID-19 (2019-nCoV) Data Repository by Johns Hopkins CSSE. Baltimore, Maryland: Johns Hopkins University.
Russia	Federal State Statistics Service (Russia). Russia Natural Movement of the Population 2021. Moscow, Russian Federation: Federal State Statistics Service (Russia).
Rwanda	Johns Hopkins University. 2019 Novel Coronavirus COVID-19 (2019-nCoV) Data Repository by Johns Hopkins CSSE. Baltimore, Maryland: Johns Hopkins University.
Saint Kitts and Nevis	Johns Hopkins University. 2019 Novel Coronavirus COVID-19 (2019-nCoV) Data Repository by Johns Hopkins CSSE. Baltimore, Maryland: Johns Hopkins University.
Saint Lucia	Johns Hopkins University. 2019 Novel Coronavirus COVID-19 (2019-nCoV) Data Repository by Johns Hopkins CSSE. Baltimore, Maryland: Johns Hopkins University.
Saint Vincent and the Grenadines	Johns Hopkins University. 2019 Novel Coronavirus COVID-19 (2019-nCoV) Data Repository by Johns Hopkins CSSE. Baltimore, Maryland: Johns Hopkins University.
Samoa	Johns Hopkins University. 2019 Novel Coronavirus COVID-19 (2019-nCoV) Data Repository by Johns Hopkins CSSE.

	Baltimore, Maryland: Johns Hopkins University.
San Marino	Johns Hopkins University. 2019 Novel Coronavirus COVID-19 (2019-nCoV) Data Repository by Johns Hopkins CSSE. Baltimore, Maryland: Johns Hopkins University.
São Tomé and Príncipe	Johns Hopkins University. 2019 Novel Coronavirus COVID-19 (2019-nCoV) Data Repository by Johns Hopkins CSSE. Baltimore, Maryland: Johns Hopkins University.
Saudi Arabia	Johns Hopkins University. 2019 Novel Coronavirus COVID-19 (2019-nCoV) Data Repository by Johns Hopkins CSSE. Baltimore, Maryland: Johns Hopkins University.
Senegal	Johns Hopkins University. 2019 Novel Coronavirus COVID-19 (2019-nCoV) Data Repository by Johns Hopkins CSSE. Baltimore, Maryland: Johns Hopkins University.
Serbia	Johns Hopkins University. 2019 Novel Coronavirus COVID-19 (2019-nCoV) Data Repository by Johns Hopkins CSSE. Baltimore, Maryland: Johns Hopkins University.
Seychelles	Johns Hopkins University. 2019 Novel Coronavirus COVID-19 (2019-nCoV) Data Repository by Johns Hopkins CSSE. Baltimore, Maryland: Johns Hopkins University.
Sierra Leone	Johns Hopkins University. 2019 Novel Coronavirus COVID-19 (2019-nCoV) Data Repository by Johns Hopkins CSSE. Baltimore, Maryland: Johns Hopkins University.
Singapore	Johns Hopkins University. 2019 Novel Coronavirus COVID-19 (2019-nCoV) Data Repository by Johns Hopkins CSSE. Baltimore, Maryland: Johns Hopkins University.
Slovakia	Johns Hopkins University. 2019 Novel Coronavirus COVID-19 (2019-nCoV) Data Repository by Johns Hopkins CSSE. Baltimore, Maryland: Johns Hopkins University.
Slovenia	Johns Hopkins University. 2019 Novel Coronavirus COVID-19 (2019-nCoV) Data Repository by Johns Hopkins CSSE. Baltimore, Maryland: Johns Hopkins University.

Solomon Islands	Johns Hopkins University. 2019 Novel Coronavirus COVID-19 (2019-nCoV) Data Repository by Johns Hopkins CSSE. Baltimore, Maryland: Johns Hopkins University.
Somalia	Johns Hopkins University. 2019 Novel Coronavirus COVID-19 (2019-nCoV) Data Repository by Johns Hopkins CSSE. Baltimore, Maryland: Johns Hopkins University.
South Africa	Johns Hopkins University. 2019 Novel Coronavirus COVID-19 (2019-nCoV) Data Repository by Johns Hopkins CSSE. Baltimore, Maryland: Johns Hopkins University.
South Korea	Johns Hopkins University. 2019 Novel Coronavirus COVID-19 (2019-nCoV) Data Repository by Johns Hopkins CSSE. Baltimore, Maryland: Johns Hopkins University.
South Sudan	Johns Hopkins University. 2019 Novel Coronavirus COVID-19 (2019-nCoV) Data Repository by Johns Hopkins CSSE. Baltimore, Maryland: Johns Hopkins University.
Spain, Andalusia	Andalusia Ministry of Health and Families (Spain). Spain - Andalusia Ministry of Health and Families Coronavirus Report.
Spain, Aragon	Ministry of Health, Consumption and Social Welfare (Spain). Spain Ministry of Health, Consumption, and Social Welfare COVID-19 Situation Update. Spain: Ministry of Health, Consumption and Social Welfare (Spain).
Spain, Aragon	Institute of Health Carlos III (Spain), Ministry of Health, Consumption and Social Welfare (Spain). Spain Carlos III Health Institute Situation of COVID-19. Madrid, Spain: Institute of Health Carlos III (Spain).
Spain, Asturias	Ministry of Health, Consumption and Social Welfare (Spain). Spain Ministry of Health, Consumption, and Social Welfare COVID-19 Situation Update. Spain: Ministry of Health, Consumption and Social Welfare (Spain).
Spain, Asturias	Institute of Health Carlos III (Spain), Ministry of Health, Consumption and Social Welfare (Spain). Spain Carlos III Health Institute Situation of COVID-19. Madrid, Spain: Institute of Health Carlos III (Spain).

Spain, Balearic Islands	Government of the Balearic Islands. Spain - Balearic Islands Ministry of Health and Consumption News About the Coronavirus COVID-19.
Spain, Basque Country	Basque Government Department of Health. Spain - Basque Country Information on the Evolution of the Coronavirus Epidemiological Bulletin.
Spain, Canary Islands	Government of the Canary Islands (Spain). Spain - Canary Islands Government COVID-19 Dashboard.
Spain, Cantabria	Cantabrian Health Service (Spain), Government of Cantabria (Spain). Spain - Cantabria Epidemiological Situation of COVID-19. Spain: Cantabrian Health Service (Spain).
Spain, Castile and León	Castile and León Board, Health Commission (Spain). Spain - Castile and León Open Data: Coronavirus (COVID-19) Epidemiological Situation.
Spain, Castile and León	Castile and León Board, Health Commission (Spain). Spain - Castile and León Open Data: Situation of Hospitalized by Coronavirus.
Spain, Castilla–La Mancha	Ministry of Health, Consumption and Social Welfare (Spain). Spain Ministry of Health, Consumption, and Social Welfare COVID-19 Situation Update. Spain: Ministry of Health, Consumption and Social Welfare (Spain).
Spain, Castilla–La Mancha	Institute of Health Carlos III (Spain), Ministry of Health, Consumption and Social Welfare (Spain). Spain Carlos III Health Institute Situation of COVID-19. Madrid, Spain: Institute of Health Carlos III (Spain).
Spain, Catalonia	Statistical Institute of Catalonia (IDESCAT) (Spain). Spain - Catalonia COVID-19 Weekly Confirmed Cases and Deaths. Barcelona, Spain: Statistical Institute of Catalonia (IDESCAT) (Spain).
Spain, Catalonia	Statistical Institute of Catalonia (IDESCAT) (Spain). Spain - Catalonia COVID-19 Daily Confirmed Cases and Deaths 2020. Barcelona, Spain: Statistical Institute of Catalonia (IDESCAT) (Spain), 2020.
Spain, Ceuta	Ministry of Health, Consumption and Social Welfare (Spain). Spain Ministry of Health, Consumption, and Social Welfare

	COVID-19 Situation Update. Spain: Ministry of Health, Consumption and Social Welfare (Spain).
Spain, Ceuta	Institute of Health Carlos III (Spain), Ministry of Health, Consumption and Social Welfare (Spain). Spain Carlos III Health Institute Situation of COVID-19. Madrid, Spain: Institute of Health Carlos III (Spain).
Spain, Community of Madrid	Ministry of Health, Consumption and Social Welfare (Spain). Spain Ministry of Health, Consumption, and Social Welfare COVID-19 Situation Update. Spain: Ministry of Health, Consumption and Social Welfare (Spain).
Spain, Community of Madrid	City of Madrid (Spain), Madrid Health Service (Spain). Spain - Madrid Health Service COVID-19 Current Situation Daily Status Report. Madrid, Spain: City of Madrid (Spain).
Spain, Extremadura	Extremadura Health Service (SES) (Spain), Government of Extremadura (Spain). Spain - Extremadura Summary of Accumulated COVID-19 Epidemiological Data. Canal Extremadura.
Spain, Galicia	Galician Healthcare Service (Spain), Regional Government of Galicia (Spain). Spain - Galicia Regional Government COVID-19 Press Releases 2020. Spain: Regional Government of Galicia (Spain).
Spain, La Rioja	Government of La Rioja (Spain). Spain - La Rioja Covid-19 Tests Performed per Days. Brazil: Government of La Rioja (Spain).
Spain, Melilla	Ministry of Health, Consumption and Social Welfare (Spain). Spain Ministry of Health, Consumption, and Social Welfare COVID-19 Situation Update. Spain: Ministry of Health, Consumption and Social Welfare (Spain).
Spain, Melilla	Institute of Health Carlos III (Spain), Ministry of Health, Consumption and Social Welfare (Spain). Spain Carlos III Health Institute Situation of COVID-19. Madrid, Spain: Institute of Health Carlos III (Spain).
Spain, Murcia	Institute of Health Carlos III (Spain), Ministry of Health, Consumption and Social Welfare (Spain). Spain Carlos III Health Institute Situation of COVID-19. Madrid, Spain: Institute of Health Carlos III (Spain).

Spain, Murcia	Ministry of Health of the Region of Murcia (Spain). COVID-19 Region of Murcia - Spain. Spain: Ministry of Health of the Region of Murcia (Spain).
Spain, Navarre	Government of Navarra (Spain). Spain - Navarra COVID-19 Testing, Deaths, Hospitalization Data May-June 2020. Spain: Government of Navarra (Spain), 2020.
Spain, Valencian Community	Valencia Ministry of Universal Health and Public Health (Spain). Spain - Valencia COVID-19: Monitoring of the Situation. Spain: Valencia Ministry of Universal Health and Public Health (Spain).
Sri Lanka	Johns Hopkins University. 2019 Novel Coronavirus COVID-19 (2019-nCoV) Data Repository by Johns Hopkins CSSE. Baltimore, Maryland: Johns Hopkins University.
Sudan	Johns Hopkins University. 2019 Novel Coronavirus COVID-19 (2019-nCoV) Data Repository by Johns Hopkins CSSE. Baltimore, Maryland: Johns Hopkins University.
Suriname	Johns Hopkins University. 2019 Novel Coronavirus COVID-19 (2019-nCoV) Data Repository by Johns Hopkins CSSE. Baltimore, Maryland: Johns Hopkins University.
Sweden	Public Health Agency of Sweden. Sweden Public Health Agency COVID-19 Confirmed Cases Daily Update. Östersund, Sweden: Public Health Agency of Sweden.
Switzerland	Federal Office of Public Health (Switzerland). Switzerland Federal Office of Public Health New Coronavirus Current Situation. Berne, Switzerland: Federal Office of Public Health (Switzerland).
Syria	Johns Hopkins University. 2019 Novel Coronavirus COVID-19 (2019-nCoV) Data Repository by Johns Hopkins CSSE. Baltimore, Maryland: Johns Hopkins University.
Taiwan (province of China)	Johns Hopkins University. 2019 Novel Coronavirus COVID-19 (2019-nCoV) Data Repository by Johns Hopkins CSSE. Baltimore, Maryland: Johns Hopkins University.

Tajikistan	Johns Hopkins University. 2019 Novel Coronavirus COVID-19 (2019-nCoV) Data Repository by Johns Hopkins CSSE. Baltimore, Maryland: Johns Hopkins University.
Tanzania	Johns Hopkins University. 2019 Novel Coronavirus COVID-19 (2019-nCoV) Data Repository by Johns Hopkins CSSE. Baltimore, Maryland: Johns Hopkins University.
Thailand	Johns Hopkins University. 2019 Novel Coronavirus COVID-19 (2019-nCoV) Data Repository by Johns Hopkins CSSE. Baltimore, Maryland: Johns Hopkins University.
The Bahamas	Johns Hopkins University. 2019 Novel Coronavirus COVID-19 (2019-nCoV) Data Repository by Johns Hopkins CSSE. Baltimore, Maryland: Johns Hopkins University.
The Gambia	Johns Hopkins University. 2019 Novel Coronavirus COVID-19 (2019-nCoV) Data Repository by Johns Hopkins CSSE. Baltimore, Maryland: Johns Hopkins University.
Timor-Leste	Johns Hopkins University. 2019 Novel Coronavirus COVID-19 (2019-nCoV) Data Repository by Johns Hopkins CSSE. Baltimore, Maryland: Johns Hopkins University.
Togo	Johns Hopkins University. 2019 Novel Coronavirus COVID-19 (2019-nCoV) Data Repository by Johns Hopkins CSSE. Baltimore, Maryland: Johns Hopkins University.
Trinidad and Tobago	Johns Hopkins University. 2019 Novel Coronavirus COVID-19 (2019-nCoV) Data Repository by Johns Hopkins CSSE. Baltimore, Maryland: Johns Hopkins University.
Tunisia	Johns Hopkins University. 2019 Novel Coronavirus COVID-19 (2019-nCoV) Data Repository by Johns Hopkins CSSE. Baltimore, Maryland: Johns Hopkins University.
Turkey	Johns Hopkins University. 2019 Novel Coronavirus COVID-19 (2019-nCoV) Data Repository by Johns Hopkins CSSE. Baltimore, Maryland: Johns Hopkins University.
Uganda	Johns Hopkins University. 2019 Novel Coronavirus COVID-19 (2019-nCoV) Data Repository by Johns Hopkins CSSE. Baltimore, Maryland: Johns Hopkins University.

UK, England	Office for National Statistics (ONS) (United Kingdom). United Kingdom - England and Wales Deaths Registered Weekly, Provisional.
UK, Northern Ireland	Office for National Statistics (ONS) (United Kingdom). United Kingdom - England and Wales Deaths Registered Weekly, Provisional.
UK, Scotland	Office for National Statistics (ONS) (United Kingdom). United Kingdom - England and Wales Deaths Registered Weekly, Provisional.
UK, Wales	Office for National Statistics (ONS) (United Kingdom). United Kingdom - England and Wales Deaths Registered Weekly, Provisional.
Ukraine	Johns Hopkins University. 2019 Novel Coronavirus COVID-19 (2019-nCoV) Data Repository by Johns Hopkins CSSE. Baltimore, Maryland: Johns Hopkins University.
United Arab Emirates	Johns Hopkins University. 2019 Novel Coronavirus COVID-19 (2019-nCoV) Data Repository by Johns Hopkins CSSE. Baltimore, Maryland: Johns Hopkins University.
Uruguay	Johns Hopkins University. 2019 Novel Coronavirus COVID-19 (2019-nCoV) Data Repository by Johns Hopkins CSSE. Baltimore, Maryland: Johns Hopkins University.
USA, Alabama	Johns Hopkins University. 2019 Novel Coronavirus COVID-19 (2019-nCoV) Data Repository by Johns Hopkins CSSE. Baltimore, Maryland: Johns Hopkins University.
USA, Alaska	Johns Hopkins University. 2019 Novel Coronavirus COVID-19 (2019-nCoV) Data Repository by Johns Hopkins CSSE. Baltimore, Maryland: Johns Hopkins University.
USA, Arizona	Johns Hopkins University. 2019 Novel Coronavirus COVID-19 (2019-nCoV) Data Repository by Johns Hopkins CSSE. Baltimore, Maryland: Johns Hopkins University.
USA, Arkansas	Johns Hopkins University. 2019 Novel Coronavirus COVID-19 (2019-nCoV) Data Repository by Johns Hopkins CSSE. Baltimore, Maryland: Johns Hopkins University.

USA, California	Johns Hopkins University. 2019 Novel Coronavirus COVID-19 (2019-nCoV) Data Repository by Johns Hopkins CSSE. Baltimore, Maryland: Johns Hopkins University.
USA, Colorado	Johns Hopkins University. 2019 Novel Coronavirus COVID-19 (2019-nCoV) Data Repository by Johns Hopkins CSSE. Baltimore, Maryland: Johns Hopkins University.
USA, Connecticut	Johns Hopkins University. 2019 Novel Coronavirus COVID-19 (2019-nCoV) Data Repository by Johns Hopkins CSSE. Baltimore, Maryland: Johns Hopkins University.
USA, Delaware	Delaware Division of Public Health (United States). United States - Delaware Division of Public Health Coronavirus Disease (COVID-19) Data Dashboard. United States of America: Delaware Division of Public Health (United States).
USA, Florida	Johns Hopkins University. 2019 Novel Coronavirus COVID-19 (2019-nCoV) Data Repository by Johns Hopkins CSSE. Baltimore, Maryland: Johns Hopkins University.
USA, Georgia	Johns Hopkins University. 2019 Novel Coronavirus COVID-19 (2019-nCoV) Data Repository by Johns Hopkins CSSE. Baltimore, Maryland: Johns Hopkins University.
USA, Hawaii	Johns Hopkins University. 2019 Novel Coronavirus COVID-19 (2019-nCoV) Data Repository by Johns Hopkins CSSE. Baltimore, Maryland: Johns Hopkins University.
USA, Idaho	Johns Hopkins University. 2019 Novel Coronavirus COVID-19 (2019-nCoV) Data Repository by Johns Hopkins CSSE. Baltimore, Maryland: Johns Hopkins University.
USA, Illinois	Johns Hopkins University. 2019 Novel Coronavirus COVID-19 (2019-nCoV) Data Repository by Johns Hopkins CSSE. Baltimore, Maryland: Johns Hopkins University.
USA, Indiana	Johns Hopkins University. 2019 Novel Coronavirus COVID-19 (2019-nCoV) Data Repository by Johns Hopkins CSSE. Baltimore, Maryland: Johns Hopkins University.
USA, Iowa	Johns Hopkins University. 2019 Novel Coronavirus COVID-19 (2019-nCoV) Data Repository by Johns Hopkins CSSE. Baltimore, Maryland: Johns Hopkins University.

USA, Kansas	Johns Hopkins University. 2019 Novel Coronavirus COVID-19 (2019-nCoV) Data Repository by Johns Hopkins CSSE. Baltimore, Maryland: Johns Hopkins University.
USA, Kentucky	Johns Hopkins University. 2019 Novel Coronavirus COVID-19 (2019-nCoV) Data Repository by Johns Hopkins CSSE. Baltimore, Maryland: Johns Hopkins University.
USA, Louisiana	Johns Hopkins University. 2019 Novel Coronavirus COVID-19 (2019-nCoV) Data Repository by Johns Hopkins CSSE. Baltimore, Maryland: Johns Hopkins University.
USA, Maine	Johns Hopkins University. 2019 Novel Coronavirus COVID-19 (2019-nCoV) Data Repository by Johns Hopkins CSSE. Baltimore, Maryland: Johns Hopkins University.
USA, Maryland	Johns Hopkins University. 2019 Novel Coronavirus COVID-19 (2019-nCoV) Data Repository by Johns Hopkins CSSE. Baltimore, Maryland: Johns Hopkins University.
USA, Massachusetts	Johns Hopkins University. 2019 Novel Coronavirus COVID-19 (2019-nCoV) Data Repository by Johns Hopkins CSSE. Baltimore, Maryland: Johns Hopkins University.
USA, Michigan	Johns Hopkins University. 2019 Novel Coronavirus COVID-19 (2019-nCoV) Data Repository by Johns Hopkins CSSE. Baltimore, Maryland: Johns Hopkins University.
USA, Minnesota	Johns Hopkins University. 2019 Novel Coronavirus COVID-19 (2019-nCoV) Data Repository by Johns Hopkins CSSE. Baltimore, Maryland: Johns Hopkins University.
USA, Mississippi	Johns Hopkins University. 2019 Novel Coronavirus COVID-19 (2019-nCoV) Data Repository by Johns Hopkins CSSE. Baltimore, Maryland: Johns Hopkins University.
USA, Missouri	Johns Hopkins University. 2019 Novel Coronavirus COVID-19 (2019-nCoV) Data Repository by Johns Hopkins CSSE. Baltimore, Maryland: Johns Hopkins University.
USA, Montana	Johns Hopkins University. 2019 Novel Coronavirus COVID-19 (2019-nCoV) Data Repository by Johns Hopkins CSSE.

	Baltimore, Maryland: Johns Hopkins University.
USA, Nebraska	Johns Hopkins University. 2019 Novel Coronavirus COVID-19 (2019-nCoV) Data Repository by Johns Hopkins CSSE. Baltimore, Maryland: Johns Hopkins University.
USA, Nevada	Johns Hopkins University. 2019 Novel Coronavirus COVID-19 (2019-nCoV) Data Repository by Johns Hopkins CSSE. Baltimore, Maryland: Johns Hopkins University.
USA, New Hampshire	Johns Hopkins University. 2019 Novel Coronavirus COVID-19 (2019-nCoV) Data Repository by Johns Hopkins CSSE. Baltimore, Maryland: Johns Hopkins University.
USA, New Jersey	Johns Hopkins University. 2019 Novel Coronavirus COVID-19 (2019-nCoV) Data Repository by Johns Hopkins CSSE. Baltimore, Maryland: Johns Hopkins University.
USA, New Mexico	Johns Hopkins University. 2019 Novel Coronavirus COVID-19 (2019-nCoV) Data Repository by Johns Hopkins CSSE. Baltimore, Maryland: Johns Hopkins University.
USA, New York	New York Times. COVID-19 Cumulative Deaths and Cases in the United States by County - New York Times. New York, United States of America: New York Times.
USA, New York	New York City Department of Health and Mental Hygiene. United States - New York City Department of Health and Mental Hygiene COVID-19 Data. New York, NY, United States of America: New York City Department of Health and Mental Hygiene.
USA, New York	Johns Hopkins University. 2019 Novel Coronavirus COVID-19 (2019-nCoV) Data Repository by Johns Hopkins CSSE. Baltimore, Maryland: Johns Hopkins University.
USA, North Carolina	Johns Hopkins University. 2019 Novel Coronavirus COVID-19 (2019-nCoV) Data Repository by Johns Hopkins CSSE. Baltimore, Maryland: Johns Hopkins University.
USA, North Dakota	Johns Hopkins University. 2019 Novel Coronavirus COVID-19 (2019-nCoV) Data Repository by Johns Hopkins CSSE. Baltimore, Maryland: Johns Hopkins University.

USA, Ohio	Ohio Department of Health. United States - Ohio Department of Health Coronavirus (COVID-19) Updates. Columbus, OH, United States of America: Ohio Department of Health.
USA, Oklahoma	Johns Hopkins University. 2019 Novel Coronavirus COVID-19 (2019-nCoV) Data Repository by Johns Hopkins CSSE. Baltimore, Maryland: Johns Hopkins University.
USA, Oregon	Johns Hopkins University. 2019 Novel Coronavirus COVID-19 (2019-nCoV) Data Repository by Johns Hopkins CSSE. Baltimore, Maryland: Johns Hopkins University.
USA, Pennsylvania	Johns Hopkins University. 2019 Novel Coronavirus COVID-19 (2019-nCoV) Data Repository by Johns Hopkins CSSE. Baltimore, Maryland: Johns Hopkins University.
USA, Rhode Island	Johns Hopkins University. 2019 Novel Coronavirus COVID-19 (2019-nCoV) Data Repository by Johns Hopkins CSSE. Baltimore, Maryland: Johns Hopkins University.
USA, South Carolina	Johns Hopkins University. 2019 Novel Coronavirus COVID-19 (2019-nCoV) Data Repository by Johns Hopkins CSSE. Baltimore, Maryland: Johns Hopkins University.
USA, South Dakota	Johns Hopkins University. 2019 Novel Coronavirus COVID-19 (2019-nCoV) Data Repository by Johns Hopkins CSSE. Baltimore, Maryland: Johns Hopkins University.
USA, Tennessee	Johns Hopkins University. 2019 Novel Coronavirus COVID-19 (2019-nCoV) Data Repository by Johns Hopkins CSSE. Baltimore, Maryland: Johns Hopkins University.
USA, Texas	Johns Hopkins University. 2019 Novel Coronavirus COVID-19 (2019-nCoV) Data Repository by Johns Hopkins CSSE. Baltimore, Maryland: Johns Hopkins University.
USA, Utah	Johns Hopkins University. 2019 Novel Coronavirus COVID-19 (2019-nCoV) Data Repository by Johns Hopkins CSSE. Baltimore, Maryland: Johns Hopkins University.
USA, Vermont	Johns Hopkins University. 2019 Novel Coronavirus COVID-19 (2019-nCoV) Data Repository by Johns Hopkins CSSE. Baltimore, Maryland: Johns Hopkins University.

USA, Virginia	Johns Hopkins University. 2019 Novel Coronavirus COVID-19 (2019-nCoV) Data Repository by Johns Hopkins CSSE. Baltimore, Maryland: Johns Hopkins University.
USA, Washington	New York Times. COVID-19 Cumulative Deaths and Cases in the United States by County - New York Times. New York, United States of America: New York Times.
USA, Washington, DC	Johns Hopkins University. 2019 Novel Coronavirus COVID-19 (2019-nCoV) Data Repository by Johns Hopkins CSSE. Baltimore, Maryland: Johns Hopkins University.
USA, West Virginia	Johns Hopkins University. 2019 Novel Coronavirus COVID-19 (2019-nCoV) Data Repository by Johns Hopkins CSSE. Baltimore, Maryland: Johns Hopkins University.
USA, Wisconsin	Johns Hopkins University. 2019 Novel Coronavirus COVID-19 (2019-nCoV) Data Repository by Johns Hopkins CSSE. Baltimore, Maryland: Johns Hopkins University.
USA, Wyoming	Johns Hopkins University. 2019 Novel Coronavirus COVID-19 (2019-nCoV) Data Repository by Johns Hopkins CSSE. Baltimore, Maryland: Johns Hopkins University.
Uzbekistan	Johns Hopkins University. 2019 Novel Coronavirus COVID-19 (2019-nCoV) Data Repository by Johns Hopkins CSSE. Baltimore, Maryland: Johns Hopkins University.
Vanuatu	Johns Hopkins University. 2019 Novel Coronavirus COVID-19 (2019-nCoV) Data Repository by Johns Hopkins CSSE. Baltimore, Maryland: Johns Hopkins University.
Venezuela	Johns Hopkins University. 2019 Novel Coronavirus COVID-19 (2019-nCoV) Data Repository by Johns Hopkins CSSE. Baltimore, Maryland: Johns Hopkins University.
Vietnam	Johns Hopkins University. 2019 Novel Coronavirus COVID-19 (2019-nCoV) Data Repository by Johns Hopkins CSSE. Baltimore, Maryland: Johns Hopkins University.
Virgin Islands	Johns Hopkins University. 2019 Novel Coronavirus COVID-19 (2019-nCoV) Data Repository by Johns Hopkins CSSE. Baltimore, Maryland: Johns Hopkins University.

Yemen	Johns Hopkins University. 2019 Novel Coronavirus COVID-19 (2019-nCoV) Data Repository by Johns Hopkins CSSE. Baltimore, Maryland: Johns Hopkins University.
Zambia	Johns Hopkins University. 2019 Novel Coronavirus COVID-19 (2019-nCoV) Data Repository by Johns Hopkins CSSE. Baltimore, Maryland: Johns Hopkins University.
Zimbabwe	Johns Hopkins University. 2019 Novel Coronavirus COVID-19 (2019-nCoV) Data Repository by Johns Hopkins CSSE. Baltimore, Maryland: Johns Hopkins University.

Section 2: Age-stratified death sources

Figure S2. Age-stratified death data coverage by location

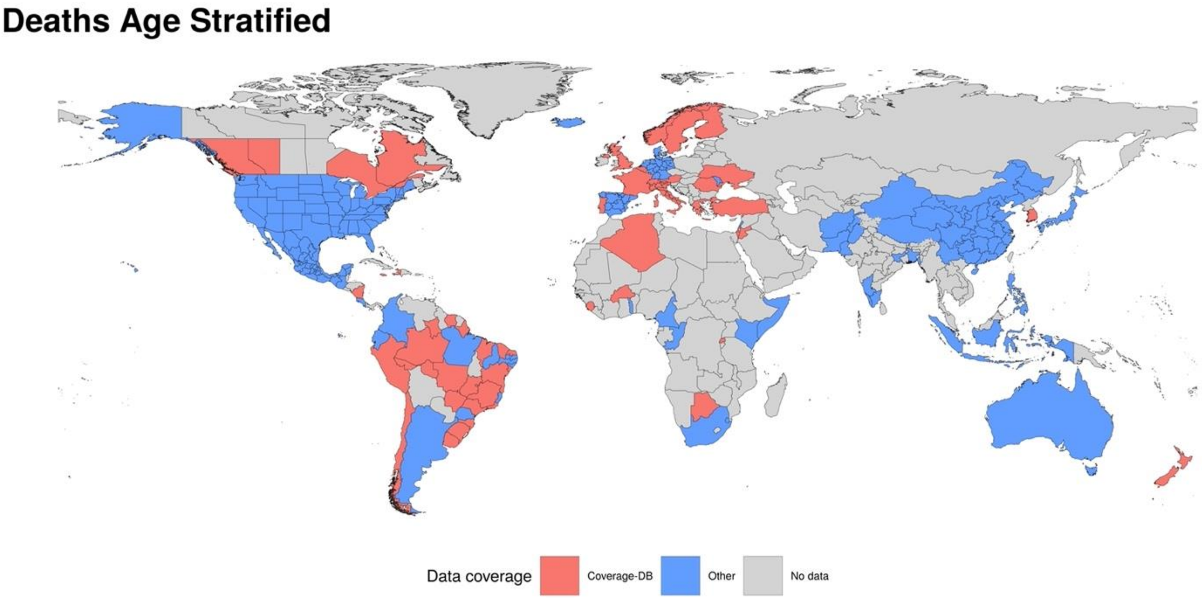

Table S2. Age-stratified death data sources by location

Location	Source
Afghanistan	Ministry of Public Health (Afghanistan). Afghanistan COVID-19 Epidemic Monitoring Dashboard. Kabul, Afghanistan: Ministry of Public Health (Afghanistan).
Algeria	Max Planck Institute for Demographic Research. COVerAGE-DB: A database of COVID-19 cases and deaths by age. United States of America: OSF, Center for Open Science (United States).
Argentina	Ministry of Health (Argentina). Argentina Ministry of Health COVID-19 Cases. Buenos Aires, Argentina: Ministry of Health (Argentina).
Australia	Coronavirus (COVID-19) in Australia. Coronavirus (COVID-19) in Australia - Fairfacts Data. Australia: Coronavirus (COVID-19) in Australia.
Australia	Department of Health (Australia). Australia Notifications of COVID-19 by Age Group and Sex . Canberra, Australia: Department of Health (Australia).
Austria	Max Planck Institute for Demographic Research. COVerAGE-DB: A database of COVID-19 cases and deaths by age. United States of America: OSF, Center for Open Science (United States).
Bangladesh	Ministry of Health and Family Welfare (Bangladesh). Bangladesh COVID-19 Status Dashboard.
Belgium	Max Planck Institute for Demographic Research. COVerAGE-DB: A database of COVID-19 cases and deaths by age. United States of America: OSF, Center for Open Science (United States).
Botswana	Max Planck Institute for Demographic Research. COVerAGE-DB: A database of COVID-19 cases and deaths by age. United States of America: OSF, Center for Open Science (United States).
Brazil	Max Planck Institute for Demographic Research. COVerAGE-DB: A database of COVID-19 cases and deaths by age. United States of America: OSF, Center for Open Science (United States).
Brazil, Acre	Max Planck Institute for Demographic Research. COVerAGE-DB: A database of COVID-19 cases and deaths by age. United States of America: OSF, Center for Open Science (United States).

Brazil, Alagoas	Government of the State of Alagoas (Brazil). Brazil - Alagoas Technical Group for Emergency Monitoring COVID-19 Epidemiological Report. Brazil: Government of the State of Alagoas (Brazil).
Brazil, Amapá	Max Planck Institute for Demographic Research. COVerAGE-DB: A database of COVID-19 cases and deaths by age. United States of America: OSF, Center for Open Science (United States).
Brazil, Amazonas	Max Planck Institute for Demographic Research. COVerAGE-DB: A database of COVID-19 cases and deaths by age. United States of America: OSF, Center for Open Science (United States).
Brazil, Bahia	Max Planck Institute for Demographic Research. COVerAGE-DB: A database of COVID-19 cases and deaths by age. United States of America: OSF, Center for Open Science (United States).
Brazil, Ceará	Max Planck Institute for Demographic Research. COVerAGE-DB: A database of COVID-19 cases and deaths by age. United States of America: OSF, Center for Open Science (United States).
Brazil, Distrito Federal	Max Planck Institute for Demographic Research. COVerAGE-DB: A database of COVID-19 cases and deaths by age. United States of America: OSF, Center for Open Science (United States).
Brazil, Espírito Santo	Government of Espírito Santo (Brazil). Brazil - Espírito Santo COVID-19 Panel.
Brazil, Goiás	Max Planck Institute for Demographic Research. COVerAGE-DB: A database of COVID-19 cases and deaths by age. United States of America: OSF, Center for Open Science (United States).
Brazil, Maranhão	Max Planck Institute for Demographic Research. COVerAGE-DB: A database of COVID-19 cases and deaths by age. United States of America: OSF, Center for Open Science (United States).
Brazil, Mato Grosso	Max Planck Institute for Demographic Research. COVerAGE-DB: A database of COVID-19 cases and deaths by age. United States of America: OSF, Center for Open Science (United States).
Brazil, Mato Grosso do Sul	Max Planck Institute for Demographic Research. COVerAGE-DB: A database of COVID-19 cases and deaths by age. United States of America: OSF, Center for Open Science (United States).
Brazil, Minas Gerais	Max Planck Institute for Demographic Research. COVerAGE-DB: A database of COVID-19 cases and deaths by age. United States of America: OSF, Center for Open Science (United States).
Brazil, Pará	Government of Pará (Brazil). Brazil - Pará, State of Coronavirus. Brazil: Government of Pará (Brazil).
Brazil, Paraíba	Government of the State of Paraíba. Brazil - Paraíba COVID-19 Epidemiological Data.
Brazil, Paraná	Government of Paraná (Brazil), Secretary of Health (Parana). Brazil - Paraná COVID-19 Epidemiological Data. Curitiba, Brazil: Secretary of Health (Parana), 2020.
Brazil, Pernambuco	State Department of Health Pernambuco (Brazil). COVID-19 in the World, in Brazil and in Pernambuco.
Brazil, Piauí	Piaui State Government. Brazil - Piaui COVID-19 Epidemiological Dashboard. Teresina, Brazil: Piaui State Government.
Brazil, Rio de Janeiro	Max Planck Institute for Demographic Research. COVerAGE-DB: A database of COVID-19 cases and deaths by age. United States of America: OSF, Center for Open Science (United States).
Brazil, Rio Grande do Norte	Max Planck Institute for Demographic Research. COVerAGE-DB: A database of COVID-19 cases and deaths by age. United States of America: OSF, Center for Open Science (United States).
Brazil, Rio Grande do Sul	Max Planck Institute for Demographic Research. COVerAGE-DB: A database of COVID-19 cases and deaths by age. United States of America: OSF, Center for Open Science (United States).

Brazil, Rondônia	Max Planck Institute for Demographic Research. COVerAGE-DB: A database of COVID-19 cases and deaths by age. United States of America: OSF, Center for Open Science (United States).
Brazil, Roraima	Max Planck Institute for Demographic Research. COVerAGE-DB: A database of COVID-19 cases and deaths by age. United States of America: OSF, Center for Open Science (United States).
Brazil, Santa Catarina	Max Planck Institute for Demographic Research. COVerAGE-DB: A database of COVID-19 cases and deaths by age. United States of America: OSF, Center for Open Science (United States).
Brazil, São Paulo	Max Planck Institute for Demographic Research. COVerAGE-DB: A database of COVID-19 cases and deaths by age. United States of America: OSF, Center for Open Science (United States).
Brazil, Sergipe	Max Planck Institute for Demographic Research. COVerAGE-DB: A database of COVID-19 cases and deaths by age. United States of America: OSF, Center for Open Science (United States).
Burkina Faso	Max Planck Institute for Demographic Research. COVerAGE-DB: A database of COVID-19 cases and deaths by age. United States of America: OSF, Center for Open Science (United States).
Cameroon	Ministry of Public Health (Cameroon). Cameroon COVID-19 Press Briefing. Yaoundé, Cameroon: Ministry of Public Health (Cameroon).
Canada	Government of Canada. Canada Coronavirus Disease 2019 (COVID-19) Daily Epidemiology Update. Ottawa, Canada: Government of Canada.
Canada, Alberta	Max Planck Institute for Demographic Research. COVerAGE-DB: A database of COVID-19 cases and deaths by age. United States of America: OSF, Center for Open Science (United States).
Canada, British Columbia	Max Planck Institute for Demographic Research. COVerAGE-DB: A database of COVID-19 cases and deaths by age. United States of America: OSF, Center for Open Science (United States).
Canada, Ontario	Max Planck Institute for Demographic Research. COVerAGE-DB: A database of COVID-19 cases and deaths by age. United States of America: OSF, Center for Open Science (United States).
Canada, Quebec	Max Planck Institute for Demographic Research. COVerAGE-DB: A database of COVID-19 cases and deaths by age. United States of America: OSF, Center for Open Science (United States).
Chile	Max Planck Institute for Demographic Research. COVerAGE-DB: A database of COVID-19 cases and deaths by age. United States of America: OSF, Center for Open Science (United States).
China	The Novel Coronavirus Pneumonia Emergency Response Epidemiology Team. The Epidemiological Characteristics of an Outbreak of 2019 Novel Coronavirus Diseases (COVID-19) — China, 2020[J]. China CDC Weekly, 2020, 2(8): 113-122.
Colombia	National Institute of Health (Colombia). Colombia COVID-19 Cases Bulletin. Colombia: National Institute of Health (Colombia).
Congo (Brazzaville)	Ministry of Health, Population, Promotion (Congo). Congo COVID-19 Epidemiological Situation. Congo: Ministry of Health and Population (Congo).
Costa Rica	Distance State University (UNED) (Costa Rica), Ministry of Health (Costa Rica). Costa Rica COVID-19 National Situation - Distance State University. San Jose, Costa Rica: Distance State University (UNED) (Costa Rica).
Denmark	State Serum Institute (Denmark). Denmark State Serum Institute Monitoring of COVID-19. Denmark: State Serum Institute (Denmark).
Ecuador	Ministry of Public Health (Ecuador). Ecuador Coronavirus Covid-19 Epidemiological Bulletins 2020. Quito, Ecuador: Ministry of Public Health (Ecuador), 2020.
Eswatini	Botswana International University of Science And Technology, DataConvergence, Ministry of Health (Eswatini), National Research Foundation (South Africa), University of Eswatini, University of Witwatersrand. Eswatini COVID-19 Dashboard. Eswatini: Ministry of Health (Eswatini).

Finland	Max Planck Institute for Demographic Research. COVerAGE-DB: A database of COVID-19 cases and deaths by age. United States of America: OSF, Center for Open Science (United States).
France	Max Planck Institute for Demographic Research. COVerAGE-DB: A database of COVID-19 cases and deaths by age. United States of America: OSF, Center for Open Science (United States).
Germany	Robert Koch Institute. Coronavirus Disease 2019 (COVID-19) Daily Situation Report - Robert Koch Institute. Berlin, Germany: Robert Koch Institute.
Greece	Max Planck Institute for Demographic Research. COVerAGE-DB: A database of COVID-19 cases and deaths by age. United States of America: OSF, Center for Open Science (United States).
Guatemala	Guatemala Ministry of Health and Social Assistance. Guatemala Daily Report Of COVID-19 Cases. Menlo Park, United States of America: Facebook.
Guatemala	Ministry of Public Health and Social Assistance (Guatemala). Guatemala COVID-19 Situation Deaths. Guatemala City, Guatemala: Ministry of Public Health and Social Assistance (Guatemala).
Haiti	Max Planck Institute for Demographic Research. COVerAGE-DB: A database of COVID-19 cases and deaths by age. United States of America: OSF, Center for Open Science (United States).
Iceland	Directorate of Health (Iceland). Iceland COVID-19 Statistics 2020. Reykjavík, Iceland: Directorate of Health (Iceland), 2020.
India	Max Planck Institute for Demographic Research. COVerAGE-DB: A database of COVID-19 cases and deaths by age. United States of America: OSF, Center for Open Science (United States).
India, Delhi	Public Health Foundation of India. India Disease Burden Initiative Covid Data by Age, Sex, and State, March 9-May 27, 2020.
India, Jharkhand	Bureau of Outreach and Communication, Regional Outreach Bureau (Jharkhand). India - Jharkhand Regional Outreach Bureau Twitter Communications and COVID-19 Updates. India: Bureau of Outreach and Communication, Regional Outreach Bureau (Jharkhand).
India, Karnataka	Public Health Foundation of India. India Disease Burden Initiative Covid Data by Age, Sex, and State, March 9-July 5, 2020.
India, Kerala	Government of Kerala. India - Kerala COVID-19 Dashboard.
India, Tamil Nadu	Health and Family Welfare Department, Government of Tamil Nadu. India - Tamil Nadu (COVID-19) Daily Media Bulletin. Chennai, India: Health and Family Welfare Department, Government of Tamil Nadu.
Indonesia	Government of Indonesia. Indonesia Situation of the COVID-19 Virus Live Data Impressions.
Indonesia, Aceh	Government of Indonesia. Indonesia Situation of the COVID-19 Virus Live Data Impressions.
Indonesia, Bali	Government of Indonesia. Indonesia Situation of the COVID-19 Virus Live Data Impressions.
Indonesia, Banten	Government of Indonesia. Indonesia Situation of the COVID-19 Virus Live Data Impressions.
Indonesia, Bengkulu	Government of Indonesia. Indonesia Situation of the COVID-19 Virus Live Data Impressions.
Indonesia, Central Java	Government of Indonesia. Indonesia Situation of the COVID-19 Virus Live Data Impressions.
Indonesia, Central Kalimantan	Government of Indonesia. Indonesia Situation of the COVID-19 Virus Live Data Impressions.
Indonesia, East Java	Government of Indonesia. Indonesia Situation of the COVID-19 Virus Live Data Impressions.
Indonesia, East Kalimantan	Government of Indonesia. Indonesia Situation of the COVID-19 Virus Live Data Impressions.

Indonesia, Gorontalo	Government of Indonesia. Indonesia Situation of the COVID-19 Virus Live Data Impressions.
Indonesia, Jakarta	Government of Indonesia. Indonesia Situation of the COVID-19 Virus Live Data Impressions.
Indonesia, Lampung	Government of Indonesia. Indonesia Situation of the COVID-19 Virus Live Data Impressions.
Indonesia, Maluku	Government of Indonesia. Indonesia Situation of the COVID-19 Virus Live Data Impressions.
Indonesia, North Maluku	Government of Indonesia. Indonesia Situation of the COVID-19 Virus Live Data Impressions.
Indonesia, North Sulawesi	Government of Indonesia. Indonesia Situation of the COVID-19 Virus Live Data Impressions.
Indonesia, North Sumatra	Government of Indonesia. Indonesia Situation of the COVID-19 Virus Live Data Impressions.
Indonesia, Papua	Government of Indonesia. Indonesia Situation of the COVID-19 Virus Live Data Impressions.
Indonesia, Riau	Government of Indonesia. Indonesia Situation of the COVID-19 Virus Live Data Impressions.
Indonesia, Riau Islands	Government of Indonesia. Indonesia Situation of the COVID-19 Virus Live Data Impressions.
Indonesia, South Kalimantan	Government of Indonesia. Indonesia Situation of the COVID-19 Virus Live Data Impressions.
Indonesia, South Sulawesi	Government of Indonesia. Indonesia Situation of the COVID-19 Virus Live Data Impressions.
Indonesia, South Sumatra	Government of Indonesia. Indonesia Situation of the COVID-19 Virus Live Data Impressions.
Indonesia, Southeast Sulawesi	Government of Indonesia. Indonesia Situation of the COVID-19 Virus Live Data Impressions.
Indonesia, West Java	Government of Indonesia. Indonesia Situation of the COVID-19 Virus Live Data Impressions.
Indonesia, West Nusa Tenggara	Government of Indonesia. Indonesia Situation of the COVID-19 Virus Live Data Impressions.
Indonesia, West Papua	Government of Indonesia. Indonesia Situation of the COVID-19 Virus Live Data Impressions.
Indonesia, West Sumatra	Government of Indonesia. Indonesia Situation of the COVID-19 Virus Live Data Impressions.
Indonesia, Yogyakarta	Government of Indonesia. Indonesia Situation of the COVID-19 Virus Live Data Impressions.
Israel	Max Planck Institute for Demographic Research. COVerAGE-DB: A database of COVID-19 cases and deaths by age. United States of America: OSF, Center for Open Science (United States).
Italy	Max Planck Institute for Demographic Research. COVerAGE-DB: A database of COVID-19 cases and deaths by age. United States of America: OSF, Center for Open Science (United States).
Jamaica	Max Planck Institute for Demographic Research. COVerAGE-DB: A database of COVID-19 cases and deaths by age. United States of America: OSF, Center for Open Science (United States).
Japan	Ministry of Health, Labour and Welfare (Japan). Japan Coronavirus Disease (COVID-19) Situation Report.
Jordan	Max Planck Institute for Demographic Research. COVerAGE-DB: A database of COVID-19 cases and deaths by age. United States of America: OSF, Center for Open Science (United States).
Kenya	Ministry of Health (Kenya). Kenya COVID-19 Situation Reports 2020. Nairobi, Kenya: Ministry of Health (Kenya), 2020.

Lebanon	Ministry of Public Health (Lebanon). Lebanon Ministry of Public Health Monitoring of COVID-19 Infection. Beirut, Lebanon: Ministry of Public Health (Lebanon).
Lebanon	Ministry of Public Health (Lebanon). Lebanon COVID-19 Dashboard. Beirut, Lebanon: Ministry of Public Health (Lebanon).
Mexico	Directorate General of Epidemiology, Secretariat of Health (Mexico). Mexico Epidemiological Report on the Situation of COVID-19.
Mexico, Aguascalientes	Directorate General of Epidemiology, Secretariat of Health (Mexico). Mexico Epidemiological Report on the Situation of COVID-19.
Mexico, Baja California	Directorate General of Epidemiology, Secretariat of Health (Mexico). Mexico Epidemiological Report on the Situation of COVID-19.
Mexico, Baja California Sur	Directorate General of Epidemiology, Secretariat of Health (Mexico). Mexico Epidemiological Report on the Situation of COVID-19.
Mexico, Campeche	Directorate General of Epidemiology, Secretariat of Health (Mexico). Mexico Epidemiological Report on the Situation of COVID-19.
Mexico, Chiapas	Directorate General of Epidemiology, Secretariat of Health (Mexico). Mexico Epidemiological Report on the Situation of COVID-19.
Mexico, Chihuahua	Directorate General of Epidemiology, Secretariat of Health (Mexico). Mexico Epidemiological Report on the Situation of COVID-19.
Mexico, Coahuila	Directorate General of Epidemiology, Secretariat of Health (Mexico). Mexico Epidemiological Report on the Situation of COVID-19.
Mexico, Colima	Directorate General of Epidemiology, Secretariat of Health (Mexico). Mexico Epidemiological Report on the Situation of COVID-19.
Mexico, Durango	Directorate General of Epidemiology, Secretariat of Health (Mexico). Mexico Epidemiological Report on the Situation of COVID-19.
Mexico, Guanajuato	Directorate General of Epidemiology, Secretariat of Health (Mexico). Mexico Epidemiological Report on the Situation of COVID-19.
Mexico, Guerrero	Directorate General of Epidemiology, Secretariat of Health (Mexico). Mexico Epidemiological Report on the Situation of COVID-19.
Mexico, Hidalgo	Directorate General of Epidemiology, Secretariat of Health (Mexico). Mexico Epidemiological Report on the Situation of COVID-19.
Mexico, Jalisco	Directorate General of Epidemiology, Secretariat of Health (Mexico). Mexico Epidemiological Report on the Situation of COVID-19.
Mexico, México	Directorate General of Epidemiology, Secretariat of Health (Mexico). Mexico Epidemiological Report on the Situation of COVID-19.
Mexico, Mexico City	Directorate General of Epidemiology, Secretariat of Health (Mexico). Mexico Epidemiological Report on the Situation of COVID-19.
Mexico, Michoacán de Ocampo	Directorate General of Epidemiology, Secretariat of Health (Mexico). Mexico Epidemiological Report on the Situation of COVID-19.
Mexico, Morelos	Directorate General of Epidemiology, Secretariat of Health (Mexico). Mexico Epidemiological Report on the Situation of COVID-19.
Mexico, Nayarit	Directorate General of Epidemiology, Secretariat of Health (Mexico). Mexico Epidemiological Report on the Situation of COVID-19.
Mexico, Nuevo León	Directorate General of Epidemiology, Secretariat of Health (Mexico). Mexico Epidemiological Report on the Situation of COVID-19.
Mexico, Oaxaca	Directorate General of Epidemiology, Secretariat of Health (Mexico). Mexico Epidemiological Report on the Situation of COVID-19.
Mexico, Puebla	Directorate General of Epidemiology, Secretariat of Health (Mexico). Mexico Epidemiological Report on the Situation of COVID-19.
Mexico, Querétaro	Directorate General of Epidemiology, Secretariat of Health (Mexico). Mexico Epidemiological Report on the Situation of COVID-19.
Mexico, Quintana Roo	Directorate General of Epidemiology, Secretariat of Health (Mexico). Mexico Epidemiological Report on the Situation of COVID-19.
Mexico, San Luis Potosí	Directorate General of Epidemiology, Secretariat of Health (Mexico). Mexico Epidemiological Report on the Situation of COVID-19.

Mexico, Sinaloa	Directorate General of Epidemiology, Secretariat of Health (Mexico). Mexico Epidemiological Report on the Situation of COVID-19.
Mexico, Sonora	Directorate General of Epidemiology, Secretariat of Health (Mexico). Mexico Epidemiological Report on the Situation of COVID-19.
Mexico, Tabasco	Directorate General of Epidemiology, Secretariat of Health (Mexico). Mexico Epidemiological Report on the Situation of COVID-19.
Mexico, Tamaulipas	Directorate General of Epidemiology, Secretariat of Health (Mexico). Mexico Epidemiological Report on the Situation of COVID-19.
Mexico, Tlaxcala	Directorate General of Epidemiology, Secretariat of Health (Mexico). Mexico Epidemiological Report on the Situation of COVID-19.
Mexico, Veracruz de Ignacio de la Llave	Directorate General of Epidemiology, Secretariat of Health (Mexico). Mexico Epidemiological Report on the Situation of COVID-19.
Mexico, Yucatán	Directorate General of Epidemiology, Secretariat of Health (Mexico). Mexico Epidemiological Report on the Situation of COVID-19.
Mexico, Zacatecas	Directorate General of Epidemiology, Secretariat of Health (Mexico). Mexico Epidemiological Report on the Situation of COVID-19.
Moldova	Ministry of Health, Labor and Social Protection of the Republic of Moldova. Republic of Moldova COVID-19 Dashboard. Ministry of Health, Labor and Social Protection of the Republic of Moldova.
Netherlands	National Institute for Public Health and the Environment (Netherlands). Netherlands Epidemiological Situation of COVID-19. Bilthoven, Netherlands: National Institute for Public Health and the Environment (Netherlands).
New Zealand	Max Planck Institute for Demographic Research. COVerAGE-DB: A database of COVID-19 cases and deaths by age. United States of America: OSF, Center for Open Science (United States).
Nicaragua	Max Planck Institute for Demographic Research. COVerAGE-DB: A database of COVID-19 cases and deaths by age. United States of America: OSF, Center for Open Science (United States).
Norway	Max Planck Institute for Demographic Research. COVerAGE-DB: A database of COVID-19 cases and deaths by age. United States of America: OSF, Center for Open Science (United States).
Pakistan	Ministry of National Health Services, Regulations & Coordination (Pakistan). Pakistan COVID-19 Dashboard.
Peru	Max Planck Institute for Demographic Research. COVerAGE-DB: A database of COVID-19 cases and deaths by age. United States of America: OSF, Center for Open Science (United States).
Philippines	Department of Health (Philippines). Philippines Department of Health COVID-19 Tracker. Manila, Philippines: Department of Health (Philippines).
Portugal	Max Planck Institute for Demographic Research. COVerAGE-DB: A database of COVID-19 cases and deaths by age. United States of America: OSF, Center for Open Science (United States).
Romania	Max Planck Institute for Demographic Research. COVerAGE-DB: A database of COVID-19 cases and deaths by age. United States of America: OSF, Center for Open Science (United States).
Rwanda	Max Planck Institute for Demographic Research. COVerAGE-DB: A database of COVID-19 cases and deaths by age. United States of America: OSF, Center for Open Science (United States).
Sierra Leone	Max Planck Institute for Demographic Research. COVerAGE-DB: A database of COVID-19 cases and deaths by age. United States of America: OSF, Center for Open Science (United States).
Singapore	Ministry of Health (Singapore). Singapore COVID-19 Tracker - Visualizing COVID-19/Coronavirus Cases and Cluster Zones 2020.

Somalia	Humanitarian Response, United Nations Office for the Coordination of Humanitarian Affairs (OCHA), World Health Organization (WHO). Somalia COVID-19 Dashboard - WHO. Geneva, Switzerland: World Health Organization (WHO).
South Africa	Department of Health (South Africa). South Africa National Department of Health Update on Coronavirus. Pretoria, South Africa: Department of Health (South Africa), 2020.
South Africa, Gauteng	Gauteng Provincial Government (South Africa). South Africa - Gauteng Province COVID-19 Status July 2020. Gauteng, South Africa: Gauteng Provincial Government (South Africa), 2020.
South Africa, Western Cape	Western Cape Government (South Africa). South Africa - Western Cape COVID-19 Dashboard.
South Korea	Max Planck Institute for Demographic Research. COVerAGE-DB: A database of COVID-19 cases and deaths by age. United States of America: OSF, Center for Open Science (United States).
Spain	Ministry of Health, Consumption and Social Welfare (Spain). Spain Ministry of Health, Consumption, and Social Welfare COVID-19 Situation Update. Spain: Ministry of Health, Consumption and Social Welfare (Spain).
Suriname	Max Planck Institute for Demographic Research. COVerAGE-DB: A database of COVID-19 cases and deaths by age. United States of America: OSF, Center for Open Science (United States).
Sweden	Max Planck Institute for Demographic Research. COVerAGE-DB: A database of COVID-19 cases and deaths by age. United States of America: OSF, Center for Open Science (United States).
Switzerland	Max Planck Institute for Demographic Research. COVerAGE-DB: A database of COVID-19 cases and deaths by age. United States of America: OSF, Center for Open Science (United States).
Togo	Government of Togo. Coronavirus in Togo: Evolution in Graphics. Lomé, Togo: Government of Togo.
Turkey	Max Planck Institute for Demographic Research. COVerAGE-DB: A database of COVID-19 cases and deaths by age. United States of America: OSF, Center for Open Science (United States).
UK	Office for National Statistics (ONS) (United Kingdom). United Kingdom - England and Wales Deaths Registered Weekly, Provisional.
UK, England	Max Planck Institute for Demographic Research. COVerAGE-DB: A database of COVID-19 cases and deaths by age. United States of America: OSF, Center for Open Science (United States).
UK, Northern Ireland	Max Planck Institute for Demographic Research. COVerAGE-DB: A database of COVID-19 cases and deaths by age. United States of America: OSF, Center for Open Science (United States).
UK, Scotland	Max Planck Institute for Demographic Research. COVerAGE-DB: A database of COVID-19 cases and deaths by age. United States of America: OSF, Center for Open Science (United States).
Ukraine	Max Planck Institute for Demographic Research. COVerAGE-DB: A database of COVID-19 cases and deaths by age. United States of America: OSF, Center for Open Science (United States).
Uruguay	Max Planck Institute for Demographic Research. COVerAGE-DB: A database of COVID-19 cases and deaths by age. United States of America: OSF, Center for Open Science (United States).
USA	National Center for Health Statistics, Centers for Disease Control and Prevention. United States NVSS Provisional Death Counts for Coronavirus Disease (COVID-19): Weekly Updates by Select Demographic and Geographic Characteristics.
USA, Alabama	National Center for Health Statistics, Centers for Disease Control and Prevention. United States NVSS Provisional Death Counts for Coronavirus Disease (COVID-19): Weekly Updates by Select Demographic and Geographic Characteristics.

USA, Alaska	National Center for Health Statistics, Centers for Disease Control and Prevention. United States NVSS Provisional Death Counts for Coronavirus Disease (COVID-19): Weekly Updates by Select Demographic and Geographic Characteristics.
USA, Arizona	National Center for Health Statistics, Centers for Disease Control and Prevention. United States NVSS Provisional Death Counts for Coronavirus Disease (COVID-19): Weekly Updates by Select Demographic and Geographic Characteristics.
USA, Arkansas	National Center for Health Statistics, Centers for Disease Control and Prevention. United States NVSS Provisional Death Counts for Coronavirus Disease (COVID-19): Weekly Updates by Select Demographic and Geographic Characteristics.
USA, California	National Center for Health Statistics, Centers for Disease Control and Prevention. United States NVSS Provisional Death Counts for Coronavirus Disease (COVID-19): Weekly Updates by Select Demographic and Geographic Characteristics.
USA, Colorado	National Center for Health Statistics, Centers for Disease Control and Prevention. United States NVSS Provisional Death Counts for Coronavirus Disease (COVID-19): Weekly Updates by Select Demographic and Geographic Characteristics.
USA, Connecticut	National Center for Health Statistics, Centers for Disease Control and Prevention. United States NVSS Provisional Death Counts for Coronavirus Disease (COVID-19): Weekly Updates by Select Demographic and Geographic Characteristics.
USA, Delaware	National Center for Health Statistics, Centers for Disease Control and Prevention. United States NVSS Provisional Death Counts for Coronavirus Disease (COVID-19): Weekly Updates by Select Demographic and Geographic Characteristics.
USA, Florida	National Center for Health Statistics, Centers for Disease Control and Prevention. United States NVSS Provisional Death Counts for Coronavirus Disease (COVID-19): Weekly Updates by Select Demographic and Geographic Characteristics.
USA, Georgia	National Center for Health Statistics, Centers for Disease Control and Prevention. United States NVSS Provisional Death Counts for Coronavirus Disease (COVID-19): Weekly Updates by Select Demographic and Geographic Characteristics.
USA, Hawaii	National Center for Health Statistics, Centers for Disease Control and Prevention. United States NVSS Provisional Death Counts for Coronavirus Disease (COVID-19): Weekly Updates by Select Demographic and Geographic Characteristics.
USA, Idaho	National Center for Health Statistics, Centers for Disease Control and Prevention. United States NVSS Provisional Death Counts for Coronavirus Disease (COVID-19): Weekly Updates by Select Demographic and Geographic Characteristics.
USA, Illinois	National Center for Health Statistics, Centers for Disease Control and Prevention. United States NVSS Provisional Death Counts for Coronavirus Disease (COVID-19): Weekly Updates by Select Demographic and Geographic Characteristics.
USA, Indiana	National Center for Health Statistics, Centers for Disease Control and Prevention. United States NVSS Provisional Death Counts for Coronavirus Disease (COVID-19): Weekly Updates by Select Demographic and Geographic Characteristics.
USA, Iowa	National Center for Health Statistics, Centers for Disease Control and Prevention. United States NVSS Provisional Death Counts for Coronavirus Disease (COVID-19): Weekly Updates by Select Demographic and Geographic Characteristics.
USA, Kansas	National Center for Health Statistics, Centers for Disease Control and Prevention. United States NVSS Provisional Death Counts for Coronavirus Disease (COVID-19): Weekly Updates by Select Demographic and Geographic Characteristics.
USA, Kentucky	National Center for Health Statistics, Centers for Disease Control and Prevention. United States NVSS Provisional Death Counts for Coronavirus Disease (COVID-19): Weekly Updates by Select Demographic and Geographic Characteristics.
USA, Louisiana	National Center for Health Statistics, Centers for Disease Control and Prevention. United States NVSS Provisional Death Counts for Coronavirus Disease (COVID-19): Weekly Updates by Select Demographic and Geographic Characteristics.
USA, Maine	National Center for Health Statistics, Centers for Disease Control and Prevention. United States NVSS Provisional Death Counts for Coronavirus Disease (COVID-19): Weekly Updates by Select Demographic and Geographic Characteristics.

USA, Maryland	National Center for Health Statistics, Centers for Disease Control and Prevention. United States NVSS Provisional Death Counts for Coronavirus Disease (COVID-19): Weekly Updates by Select Demographic and Geographic Characteristics.
USA, Massachusetts	National Center for Health Statistics, Centers for Disease Control and Prevention. United States NVSS Provisional Death Counts for Coronavirus Disease (COVID-19): Weekly Updates by Select Demographic and Geographic Characteristics.
USA, Michigan	National Center for Health Statistics, Centers for Disease Control and Prevention. United States NVSS Provisional Death Counts for Coronavirus Disease (COVID-19): Weekly Updates by Select Demographic and Geographic Characteristics.
USA, Minnesota	National Center for Health Statistics, Centers for Disease Control and Prevention. United States NVSS Provisional Death Counts for Coronavirus Disease (COVID-19): Weekly Updates by Select Demographic and Geographic Characteristics.
USA, Mississippi	National Center for Health Statistics, Centers for Disease Control and Prevention. United States NVSS Provisional Death Counts for Coronavirus Disease (COVID-19): Weekly Updates by Select Demographic and Geographic Characteristics.
USA, Missouri	National Center for Health Statistics, Centers for Disease Control and Prevention. United States NVSS Provisional Death Counts for Coronavirus Disease (COVID-19): Weekly Updates by Select Demographic and Geographic Characteristics.
USA, Montana	National Center for Health Statistics, Centers for Disease Control and Prevention. United States NVSS Provisional Death Counts for Coronavirus Disease (COVID-19): Weekly Updates by Select Demographic and Geographic Characteristics.
USA, Nebraska	National Center for Health Statistics, Centers for Disease Control and Prevention. United States NVSS Provisional Death Counts for Coronavirus Disease (COVID-19): Weekly Updates by Select Demographic and Geographic Characteristics.
USA, Nevada	National Center for Health Statistics, Centers for Disease Control and Prevention. United States NVSS Provisional Death Counts for Coronavirus Disease (COVID-19): Weekly Updates by Select Demographic and Geographic Characteristics.
USA, New Hampshire	National Center for Health Statistics, Centers for Disease Control and Prevention. United States NVSS Provisional Death Counts for Coronavirus Disease (COVID-19): Weekly Updates by Select Demographic and Geographic Characteristics.
USA, New Jersey	National Center for Health Statistics, Centers for Disease Control and Prevention. United States NVSS Provisional Death Counts for Coronavirus Disease (COVID-19): Weekly Updates by Select Demographic and Geographic Characteristics.
USA, New Mexico	National Center for Health Statistics, Centers for Disease Control and Prevention. United States NVSS Provisional Death Counts for Coronavirus Disease (COVID-19): Weekly Updates by Select Demographic and Geographic Characteristics.
USA, New York	National Center for Health Statistics, Centers for Disease Control and Prevention. United States NVSS Provisional Death Counts for Coronavirus Disease (COVID-19): Weekly Updates by Select Demographic and Geographic Characteristics.
USA, North Carolina	National Center for Health Statistics, Centers for Disease Control and Prevention. United States NVSS Provisional Death Counts for Coronavirus Disease (COVID-19): Weekly Updates by Select Demographic and Geographic Characteristics.
USA, North Dakota	National Center for Health Statistics, Centers for Disease Control and Prevention. United States NVSS Provisional Death Counts for Coronavirus Disease (COVID-19): Weekly Updates by Select Demographic and Geographic Characteristics.
USA, Ohio	National Center for Health Statistics, Centers for Disease Control and Prevention. United States NVSS Provisional Death Counts for Coronavirus Disease (COVID-19): Weekly Updates by Select Demographic and Geographic Characteristics.
USA, Oklahoma	National Center for Health Statistics, Centers for Disease Control and Prevention. United States NVSS Provisional Death Counts for Coronavirus Disease (COVID-19): Weekly Updates by Select Demographic and Geographic Characteristics.
USA, Oregon	National Center for Health Statistics, Centers for Disease Control and Prevention. United States NVSS Provisional Death Counts for Coronavirus Disease (COVID-19): Weekly Updates by Select Demographic and Geographic Characteristics.

USA, Pennsylvania	National Center for Health Statistics, Centers for Disease Control and Prevention. United States NVSS Provisional Death Counts for Coronavirus Disease (COVID-19): Weekly Updates by Select Demographic and Geographic Characteristics.
USA, Rhode Island	National Center for Health Statistics, Centers for Disease Control and Prevention. United States NVSS Provisional Death Counts for Coronavirus Disease (COVID-19): Weekly Updates by Select Demographic and Geographic Characteristics.
USA, South Carolina	National Center for Health Statistics, Centers for Disease Control and Prevention. United States NVSS Provisional Death Counts for Coronavirus Disease (COVID-19): Weekly Updates by Select Demographic and Geographic Characteristics.
USA, South Dakota	National Center for Health Statistics, Centers for Disease Control and Prevention. United States NVSS Provisional Death Counts for Coronavirus Disease (COVID-19): Weekly Updates by Select Demographic and Geographic Characteristics.
USA, Tennessee	National Center for Health Statistics, Centers for Disease Control and Prevention. United States NVSS Provisional Death Counts for Coronavirus Disease (COVID-19): Weekly Updates by Select Demographic and Geographic Characteristics.
USA, Texas	National Center for Health Statistics, Centers for Disease Control and Prevention. United States NVSS Provisional Death Counts for Coronavirus Disease (COVID-19): Weekly Updates by Select Demographic and Geographic Characteristics.
USA, Utah	National Center for Health Statistics, Centers for Disease Control and Prevention. United States NVSS Provisional Death Counts for Coronavirus Disease (COVID-19): Weekly Updates by Select Demographic and Geographic Characteristics.
USA, Vermont	National Center for Health Statistics, Centers for Disease Control and Prevention. United States NVSS Provisional Death Counts for Coronavirus Disease (COVID-19): Weekly Updates by Select Demographic and Geographic Characteristics.
USA, Virginia	National Center for Health Statistics, Centers for Disease Control and Prevention. United States NVSS Provisional Death Counts for Coronavirus Disease (COVID-19): Weekly Updates by Select Demographic and Geographic Characteristics.
USA, Washington	National Center for Health Statistics, Centers for Disease Control and Prevention. United States NVSS Provisional Death Counts for Coronavirus Disease (COVID-19): Weekly Updates by Select Demographic and Geographic Characteristics.
USA, Washington, DC	National Center for Health Statistics, Centers for Disease Control and Prevention. United States NVSS Provisional Death Counts for Coronavirus Disease (COVID-19): Weekly Updates by Select Demographic and Geographic Characteristics.
USA, West Virginia	National Center for Health Statistics, Centers for Disease Control and Prevention. United States NVSS Provisional Death Counts for Coronavirus Disease (COVID-19): Weekly Updates by Select Demographic and Geographic Characteristics.
USA, Wisconsin	National Center for Health Statistics, Centers for Disease Control and Prevention. United States NVSS Provisional Death Counts for Coronavirus Disease (COVID-19): Weekly Updates by Select Demographic and Geographic Characteristics.
USA, Wyoming	National Center for Health Statistics, Centers for Disease Control and Prevention. United States NVSS Provisional Death Counts for Coronavirus Disease (COVID-19): Weekly Updates by Select Demographic and Geographic Characteristics.

Section 3: Seroprevalence sources

Figure S3. Seroprevalence data coverage by location

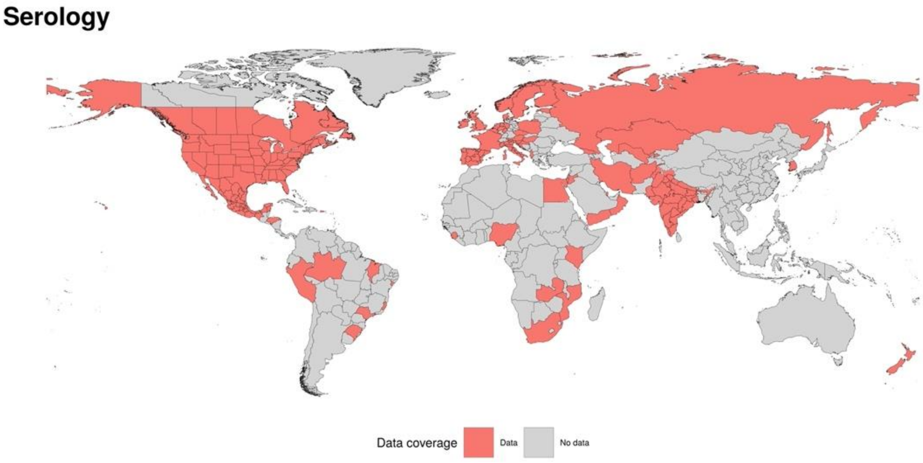

Table S3. Seroprevalence data sources by location

Location	Source
Afghanistan	World Health Organization (WHO). Prevalence of COVID-19 and its Related Deaths in Afghanistan: A Nationwide, Population-Based Seroepidemiological Study July 2020. Kabul, Afghanistan: Ministry of Public Health (Afghanistan), 2021.
Albania	Sulcebe G, Ylli A, Cenko F, Kurti-Prifti M. Rapid increase of SARS-CoV-2 seroprevalence during the 2020 pandemic year in the population of the city of Tirana, Albania. Preprint. medRxiv. 2021.
Andorra	Government of Andorra. [Martinez Benazet thanks institutions, volunteers and the population who have participated in the national antibody study]. Government of Andorra [Internet]. 2020 May 28; Press Releases.
Andorra	Royo-Cebrecos C, Vilanova D, López J, Arroyo V, Francisco G, Pons M, Carrasco MG, Piqué JM, Sanz S, Dobaño C, García-Basteiro A. Mass SARS-CoV-2 serological screening for the Principality of Andorra. Preprint. Res Sq. 2020.
Austria	Statistics Austria. 4.7% of the Austrian population had antibodies against SARS-CoV -2 in mid / late October. Vienna, Austria: Statistics Austria, 2020.
Belgium	Sciensano (Belgium). Belgium Prevalence of anti-SARS-CoV-2 antibodies (IgG) among blood donors. Belgium: Sciensano (Belgium).
Belgium	Herzog S, De Bie J, Abrams S, Wouters I, Ekinci E, Patteet L, Coppens A, De Spiegeleer S, Beutels P, Van Damme P, Hens N, Theeten H. Seroprevalence of IgG antibodies against SARS coronavirus 2 in Belgium - a serial prospective cross-sectional nationwide study of residual samples. Preprint. medRxiv. 2020.
Brazil	Hallal PC, Hartwig FP, Horta BL, Silveira MF, Struchiner CJ, Vidaletti LP, Neumann NA, Pellanda LC, Dellagostin OA, Burattini MN, Victora GD, Menezes AMB, Barros FC, Barros AJD, Victora CG. SARS-CoV-2 antibody prevalence in Brazil: results from two successive nationwide serological household surveys. Lancet Glob Health. 2020; 8(11): e1390-e1398.
Brazil	Menezes AMB, Victora CG, Hartwig FP, Silveira MF, Horta BL, Barros AJD, Mesenburg MA, Wehrmeister FC, Pellanda LC, Dellagostin OA, Struchiner CJ, Burattini MN, Barros FC, Hallal PC. High prevalence of symptoms among Brazilian subjects with antibodies against SARS-CoV-2. Sci Rep. 2021; 11(1): 13279.
Brazil, Amazonas	Buss LF, Prete CA Jr, Abrahim CMM, Mendrone A Jr, Salomon T, de Almeida-Neto C, França RFO, Belotti MC, Carvalho MPSS, Costa AG, Crispim MAE, Ferreira SC, Fraiji NA, Gurzenda S, Whittaker C, Kamaura LT, Takecian PL, da Silva Peixoto P, Oikawa MK, Nishiya AS, Rocha V, Salles NA, de Souza Santos AA, da Silva MA, Custer B, Parag KV, Barral-Netto M,

	Kraemer MUG, Pereira RHM, Pybus OG, Busch MP, Castro MC, Dye C, Nascimento VH, Faria NR, Sabino EC. Three-quarters attack rate of SARS-CoV-2 in the Brazilian Amazon during a largely unmitigated epidemic. Science. 2021; 371(6526): 288-292.
Brazil, Espírito Santo	Gomes CC, Cerutti Jr C, Zandonade E, Maciel ELN, Carvalho de Alencar FE, Almada GL, Cardoso OA, Medeiros Jabor P, Zanotti RL, Queiroz Reuter T, Gomes de Andrade VL, Bastos WM, Fernandes de Medeiros Jr N. A population-based study of the prevalence of COVID-19 infection in Espírito Santo, Brazil: methodology and results of the first stage. Preprint. medRxiv. 2020.
Brazil, Espírito Santo	Government of Espírito Santo (Brazil). Brazil - Government of Espírito Santo COVID-19 Serological Survey.
Brazil, Maranhão	Silva AAM da, Lima Neto LG, Azevedo C de MP e S de, Costa LMM da, Bragança MLBM, Barros Filho AKD, Wittlin BB, Souza BF de, Oliveira BLCA de, Carvalho CA de, Thomaz ÉBAF, Simões Neto EA, Leite Júnior JF, Cosme LMSS, Campos MAG, Queiroz RC de S, Costa SS, Carvalho VA de, Simóes VMF, Alves MTSS de B e, Santos AM dos. Population-based seroprevalence of SARS-CoV-2 is more than halfway through the herd immunity threshold in the State of Maranhao, Brazil. Preprint. medRxiv. 2020.
Brazil, Maranhão	Secretary of State for Health (Brazil - Maranhão). Prevalence of SARS-CoV-2 virus infection in Maranhão, Brazil, Final Research Report, Phase II, October 2020.
Brazil, Rio Grande do Sul	Government of Rio Grande do Sol. Brazil - Rio Grande Do Sol Epicovid19 Relevance Study 2020.
Brazil, São Paulo	Albuquerque JOM, Kamioka GA, Madalosso G, Costa SA, Ferreira PB, Pino FA, Sato APS, Carvalho ACA, Amorim ABP, Aires CC, Kataoka APAG, Savani ESMM, Bessa TAF, Aguiar BS, Failla MA, Santos EA, Brito EMT, Santos MCH, Silva SMS, Caldeira LAV, Zamarco LC, Fonseca SMS, Lima MMC, Marques IA, Silva FEV, Glasser PR, Burihan PCPR, Cavazzana CL, Lara RC, Mello DS, Pellini ACG, Nishio FY, Kian FM, Braga ES, Bertelli NMP, Fracini W, Gonçalves MDA, Zular PS, Piva RS, Masi E de. Prevalence evolution of SARS-CoV-2 infection in the Municipality of São Paulo, 2020 - 2021. medRxiv. 2021.
Brazil, São Paulo	Buss LF, Prete CA Jr, Abrahim CMM, Mendrone A Jr, Salomon T, de Almeida-Neto C, França RFO, Belotti MC, Carvalho MPSS, Costa AG, Crispim MAE, Ferreira SC, Fraiji NA, Gurzenda S, Whittaker C, Kamaura LT, Takecian PL, da Silva Peixoto P, Oikawa MK, Nishiya AS, Rocha V, Salles NA, de Souza Santos AA, da Silva MA, Custer B, Parag KV, Barral-Netto M, Kraemer MUG, Pereira RHM, Pybus OG, Busch MP, Castro MC, Dye C, Nascimento VH, Faria NR, Sabino EC. Three-quarters attack rate of SARS-CoV-2 in the Brazilian Amazon during a largely unmitigated epidemic. Science. 2021; 371(6526): 288-292.
Brazil, São Paulo	Brazilian Institute of Public Opinion and Statistics (IBOPE), Fleury Group, SEMEIA Institute (Brazil). Brazil - São Paulo Household Survey to Estimate the Seroprevalence of SARS-CoV-2 Infection, Pilot Study (SoroEpi MSP).
Brazil, São Paulo	Brazilian Institute of Public Opinion and Statistics (IBOPE), Fleury Group, SEMEIA Institute (Brazil). Brazil - São Paulo Household Survey to Estimate the Seroprevalence of SARS-CoV-2 Infection, Phase 2 (SoroEpi MSP).
Brazil, Sergipe	Federal University of Sergipe (Brazil). Brazil - Evolution of the Prevalence of Infection by SARS-CoV-2 in Sergipe. Third Phase Results: February 18 to March 30, 2021.
Canada	Investigators A to beat coronavirus/Action pour battre le coronavirus (Ab-CS, Jha P. COVID Seroprevalence, Symptoms and Mortality During the First Wave of SARS-CoV-2 in Canada. medRxiv. 2021.
Canada	Canadian Blood Services. Canada COVID-19 Seroprevalence Report - August 19, 2020. Ottawa, Canada: Canadian Blood Services, 2020.
Canada	Canadian Blood Services. Canada COVID-19 Seroprevalence Report - December 18, 2020. Ottawa, Canada: Canadian Blood Services, 2020.
Canada	Canadian Blood Services. Canada COVID-19 Seroprevalence Report - January 12, 2021. Ottawa, Canada: Canadian Blood Services, 2021.
Canada	Canadian Blood Services. Canada COVID-19 Seroprevalence Report - April 2020 to January 2021. Ottawa, Canada: Canadian Blood Services, 2021.

Canada, Alberta	Canadian Blood Services. Canada COVID-19 Seroprevalence Report - August 19, 2020. Ottawa, Canada: Canadian Blood Services, 2020.
Canada, Alberta	Saeed S, Drews SJ, Pambrun C, Yi QL, Osmond L, O'Brien SF. SARS-CoV-2 seroprevalence among blood donors after the first COVID-19 wave in Canada. Transfusion. 2021; 61(3): 862-872.
Canada, Alberta	Canadian Blood Services. Canada COVID-19 Seroprevalence Report - December 18, 2020. Ottawa, Canada: Canadian Blood Services, 2020.
Canada, Alberta	Canadian Blood Services. Canada COVID-19 Seroprevalence Report - January 12, 2021. Ottawa, Canada: Canadian Blood Services, 2021.
Canada, British Columbia	Investigators A to beat coronavirus/Action pour battre le coronavirus (Ab-CS, Jha P. COVID Seroprevalence, Symptoms and Mortality During the First Wave of SARS-CoV-2 in Canada. medRxiv. 2021.
Canada, British Columbia	Canadian Blood Services. Canada COVID-19 Seroprevalence Report - August 19, 2020. Ottawa, Canada: Canadian Blood Services, 2020.
Canada, British Columbia	Saeed S, Drews SJ, Pambrun C, Yi QL, Osmond L, O'Brien SF. SARS-CoV-2 seroprevalence among blood donors after the first COVID-19 wave in Canada. Transfusion. 2021; 61(3): 862-872.
Canada, British Columbia	Canadian Blood Services. Canada COVID-19 Seroprevalence Report - December 18, 2020. Ottawa, Canada: Canadian Blood Services, 2020.
Canada, British Columbia	Canadian Blood Services. Canada COVID-19 Seroprevalence Report - January 12, 2021. Ottawa, Canada: Canadian Blood Services, 2021.
Canada, Manitoba	Canadian Blood Services. Canada COVID-19 Seroprevalence Report - December 18, 2020. Ottawa, Canada: Canadian Blood Services, 2020.
Canada, Manitoba	Canadian Blood Services. Canada COVID-19 Seroprevalence Report - January 12, 2021. Ottawa, Canada: Canadian Blood Services, 2021.
Canada, New Brunswick	Canadian Blood Services. Canada COVID-19 Seroprevalence Report - August 19, 2020. Ottawa, Canada: Canadian Blood Services, 2020.
Canada, New Brunswick	Canadian Blood Services. Canada COVID-19 Seroprevalence Report - December 18, 2020. Ottawa, Canada: Canadian Blood Services, 2020.
Canada, New Brunswick	Canadian Blood Services. Canada COVID-19 Seroprevalence Report - January 12, 2021. Ottawa, Canada: Canadian Blood Services, 2021.
Canada, Newfoundland and Labrador	Canadian Blood Services. Canada COVID-19 Seroprevalence Report - August 19, 2020. Ottawa, Canada: Canadian Blood Services, 2020.
Canada, Newfoundland and Labrador	Canadian Blood Services. Canada COVID-19 Seroprevalence Report - December 18, 2020. Ottawa, Canada: Canadian Blood Services, 2020.
Canada, Newfoundland and Labrador	Canadian Blood Services. Canada COVID-19 Seroprevalence Report - January 12, 2021. Ottawa, Canada: Canadian Blood Services, 2021.
Canada, Nova Scotia	Canadian Blood Services. Canada COVID-19 Seroprevalence Report - August 19, 2020. Ottawa, Canada: Canadian Blood Services, 2020.
Canada, Nova Scotia	Canadian Blood Services. Canada COVID-19 Seroprevalence Report - December 18, 2020. Ottawa, Canada: Canadian Blood Services, 2020.
Canada, Nova Scotia	Canadian Blood Services. Canada COVID-19 Seroprevalence Report - January 12, 2021. Ottawa, Canada: Canadian Blood Services, 2021.

Canada, Ontario	Bolotin S, Tran V, Osman S, Brown KA, Buchan SA, Joh E, Deeks SL, Allen VG. SARS-CoV-2 Seroprevalence Survey Estimates Are Affected by Anti-Nucleocapsid Antibody Decline. J Infect Dis. 2021; 223(8): 1334-1338.
Canada, Ontario	Investigators A to beat coronavirus/Action pour battre le coronavirus (Ab-CS, Jha P. COVID Seroprevalence, Symptoms and Mortality During the First Wave of SARS-CoV-2 in Canada. medRxiv. 2021.
Canada, Ontario	Canadian Blood Services. Canada COVID-19 Seroprevalence Report - August 19, 2020. Ottawa, Canada: Canadian Blood Services, 2020.
Canada, Ontario	Saeed S, Drews SJ, Pambrun C, Yi QL, Osmond L, O'Brien SF. SARS-CoV-2 seroprevalence among blood donors after the first COVID-19 wave in Canada. Transfusion. 2021; 61(3): 862-872.
Canada, Ontario	Canadian Blood Services. Canada COVID-19 Seroprevalence Report - December 18, 2020. Ottawa, Canada: Canadian Blood Services, 2020.
Canada, Ontario	Canadian Blood Services. Canada COVID-19 Seroprevalence Report - January 12, 2021. Ottawa, Canada: Canadian Blood Services, 2021.
Canada, Ontario	Government of Ontario (Canada), Public Health Ontario (Canada). Canada COVID-19 Seroprevalence in Ontario: March 27, 2020 to June 30, 2020. Canada: Public Health Ontario (Canada), 2020.
Canada, Ontario	Government of Ontario (Canada), Public Health Ontario (Canada). Canada COVID-19 Seroprevalence in Ontario: July 4 to July 31, 2020. Canada: Public Health Ontario (Canada), 2020.
Canada, Ontario	Government of Ontario (Canada), Public Health Ontario (Canada). Canada COVID-19 Seroprevalence in Ontario: August 1 to August 31, 2020. Canada: Public Health Ontario (Canada), 2020.
Canada, Ontario	Government of Ontario (Canada), Public Health Ontario (Canada). Canada COVID-19 Seroprevalence in Ontario: September 3 to October 30, 2020. Canada: Public Health Ontario (Canada), 2020.
Canada, Quebec	Investigators A to beat coronavirus/Action pour battre le coronavirus (Ab-CS, Jha P. COVID Seroprevalence, Symptoms and Mortality During the First Wave of SARS-CoV-2 in Canada. medRxiv. 2021.
Canada, Quebec	Hema-Quebec (Canada), Quebec National Institute of Public Health (Canada). Canada - Quebec COVID-19 First Wave Blood Donor Seroprevalence Study 2020. Canada: Hema-Quebec (Canada).
Canada, Saskatchewan	Canadian Blood Services. Canada COVID-19 Seroprevalence Report - January 12, 2021. Ottawa, Canada: Canadian Blood Services, 2021.
Croatia	Vilibic-Cavlek T, Stevanovic V, Ilic M, Barbic L, Capak K, Tabain I, Krleza JL, Ferenc T, Hruskar Z, Topic RZ, Kaliterna V, Antolovic-Pozgain A, Kucinar J, Koscak I, Mayer D, Sviben M, Antolasic L, Milasincic L, Bucic L, Ferencak I, Kaic B. SARS-CoV-2 Seroprevalence and Neutralizing Antibody Response after the First and Second COVID-19 Pandemic Wave in Croatia. Pathogens. 2021; 10(6): 774.
Czechia	Piler P, Thon V, Andrýsková L, Doležel K, Kostka D, Pavlík T, Dušek L, Pikhart H, Bobák M, Matic S, Klánová J. Dynamics of seroconversion of anti-SARS-CoV-2 IgG antibodies in the Czech unvaccinated population: nationwide prospective seroconversion (PROSECO) study. Preprint. medRxiv. 2021.
Denmark	Blood Donor (Denmark). Denmark Blood Donors are Now Being Re-examined for Coronavirus Infection. Denmark: Blood Donor (Denmark), 2020.
Denmark	Erikstrup C, Hother CE, Pedersen OBV, Mølbak K, Skov RL, Holm DK, Sækmose SG, Nilsson AC, Brooks PT, Boldsen JK, Mikkelsen C, Gybel-Brask M, Sørensen E, Dinh KM, Mikkelsen S, Møller BK, Haunstrup T, Harritshøj L, Jensen BA, Hjalgrim H, Lillevang ST, Ullum H. Estimation of SARS-CoV-2 infection fatality rate by real-time antibody screening of blood donors. Clin Infect Dis. 2020.
Denmark	Espenhain L, Tribler S, Jørgensen CS, Hansen CH, Sönksen UW, Ethelberg S. Prevalence of SARS-CoV-2 antibodies in Denmark 2020: results from nationwide, population-based sero-epidemiological surveys. Preprint. medRxiv. 2021.

Egypt	Gomaa MR, El Rifay AS, Shehata M, Kandeil A, Nabil Kamel M, Marouf MA, GabAllah M, El Taweel A, Kayed AE, Kutkat O, Moatasim Y, Mahmoud SH, Abo Shama NM, El Sayes M, Mostafa A, El-Shesheny R, McKenzie PP, Webby RJ, Kayali G, Ali MA. Incidence, household transmission, and neutralizing antibody seroprevalence of Coronavirus Disease 2019 in Egypt: Results of a community-based cohort. PLOS Pathog. 2021; 17(3): e1009413.
Estonia	Soeorg H, Jõgi P, Naaber P, Ottas A, Toompere K, Lutsar I. Anti-spike protein receptor-binding domain IgG levels after COVID-19 infection or vaccination against SARS-CoV-2 in a seroprevalence study. Preprint. medRxiv. 2021.
Estonia	University of Tartu. Estonia COVID-19 Coronavirus prevalence study.
Finland	National Institute for Health and Welfare (THL) (Finland). Finland Weekly Report of the Population Serology Survey of the Corona Epidemic. Helsinki, Finland: National Institute for Health and Welfare (THL) (Finland).
France	Warszawski J, Bajos N, Meyer L, de Lamballerie X, Seng R, Beaumont A, Slama R, Hisbergues M, Rahib D, Lydie N, Legendre B, Barlet M, Rey S, Raynaud P, Leduc A, Costemalle V, Beck F, Legleye S, Castell L, Givord P, Favre-Martinoz C, Paliod N, Sihol J, Sillard P. In May 2020, 4.5% of the population of metropolitan France had developed antibodies against SARS-CoV-2. Études et Résultats. 2020; (1167): 1-6.
France	Vu SL, Jones G, Anna F, Rose T, Richard J-B, Bernard-Stoecklin S, Goyard S, Demeret C, Helynck O, Robin C, Monnet V, Facci LP de, Ungeheuer M-N, Léon L, Guillois Y, Filleul L, Charneau P, Lévy-Bruhl D, van der Werf S, Noel H. Prevalence of SARS-CoV-2 antibodies in France: results from nationwide serological surveillance. Preprint. medRxiv. 2020.
Georgia	*
Germany	Robert Koch Institute. Coronavirus Disease (COVID-19) Nationwide Monitoring, Overview of First Results (RKI-SOEP study) - Robert Koch Institute. Berlin, Germany: Robert Koch Institute, 2021.
Germany	Federal Ministry of Health (Germany). The Germans and Corona, Final Report of the Federal Ministry of Health's 'Corona-BUND-Study' . 2020.
Germany, Hesse	Bastian F, Cornelius K, Tanja V. SARS-CoV-2 IgG seroprevalence in blood donors located in three different federal states, Germany, March to June 2020. Euro Surveill. 2020; 25(28).
Germany, Lower Saxony	Bastian F, Cornelius K, Tanja V. SARS-CoV-2 IgG seroprevalence in blood donors located in three different federal states, Germany, March to June 2020. Euro Surveill. 2020; 25(28).
Germany, North Rhine-Westphalia	Bastian F, Cornelius K, Tanja V. SARS-CoV-2 IgG seroprevalence in blood donors located in three different federal states, Germany, March to June 2020. Euro Surveill. 2020; 25(28).
Germany, Saarland	Saarland University Hospital and Medical Faculty of Saarland University.Germany - Saarland antibody study on coronavirus infection completed: Institute for Virology at Saarland University Hospital presents results. Saarland University Hospital and Medical Faculty of Saarland University [Internet]. 2021 Jan 21.
Honduras	Núñez MRM, Lara JÁ, Pleitez SJA, Ramírez GA. [Seroepidemiological surveillance of SARS-CoV-2 circulation in 41 municipalities in Honduras with no report of active cases, COVID-19, June 16-23, 2020]. Poblac Salud Mesoam. 2021; 18(2).
Hungary	Merkely B, Szabó AJ, Kosztin A, Berényi E, Sebestyén A, Lengyel C, Merkely G, Karády J, Várkonyi I, Papp C, Miseta A, Betlehem J, Burián K, Csóka I, Vásárhelyi B, Ludwig E, Prinz G, Sinkó J, Hankó B, Varga P, Fülöp GÁ, Mag K, Vokó Z, HUNgarian COronaVirus-19 Epidemiological Research (H-UNCOVER) investigators. Novel coronavirus epidemic in the Hungarian population, a cross-sectional nationwide survey to support the exit policy in Hungary. GeroScience. 2020; 42(4): 1063-74.
India	Naushin S, Sardana V, Ujjainiya R, Bhatheja N, Kutum R, Bhaskar AK, Pradhan S, Prakash S, Khan R, Rawat BS, Tallapaka KB, Anumalla M, Chandak GR, Lahiri A, Kar S, Mulay SR, Mugale MN, Srivastava M, Khan S, Srivastava A, Tomar B, Veerapandian M, Venkatachalam G, Vijayakumar SR, Agarwal A, Gupta D, Halami PH, Peddha MS, Sundaram GM, Veeranna RP, Pal A, Agarwal VK, Maurya AK, Singh RVK, Raman AK, Anandasadagopan SK, Karuppanan P, Venkatesan S, Sardana HK, Kothari A, Jain R, Thakur A, Parihar DS, Saifi A, Kaur J, Kumar V, Mishra A, Gogeri I, Rayasam G, Singh P, Chakraborty R, Chaturvedi G,

	Karunakar P, Yadav R, Singhmar S, Singh D, Sarkar S, Bhattacharya P, Acharya S, Singh V, Verma S, Soni D, Seth S, Vashisht S, Thakran S, Fatima F, Singh AP, Sharma A, Sharma B, Subramanian M, Padwad Y, Hallan V, Patial V, Singh D, Tirpude NV, Chakrabarti P, Maity SK, Ganguly D, Sarkar J, Ramakrishna S. Insights from a Pan India Sero-Epidemiological survey (Phenome-India Cohort) for SARS-CoV2. Preprint. medRxiv. 2021.
India	Murhekar MV, Bhatnagar T, Thangaraj J, Saravanakumar V, Kumar MS, Selvaraju S, Rade K, Kumar C, Sabarinathan R, Turuk A, Asthana S, Balachandar R, Bangar SD, Bansal AK, Chopra V, Das D, Deb AK, Devi KR, Dhikav V, Dwivedi GR, ICMR Serosurveillance Group (2021). I SARS-CoV-2 seroprevalence among the general population and healthcare workers in India, December 2020-January 2021. 2021; 108: 145-155.
India	Government of India. India COVID-19 Official Government of India updates - Twitter.
India	Murhekar MV, Bhatnagar T, Selvaraju S, Rade K, Saravanakumar V, Vivian Thangaraj JW, Kumar MS, Shah N, Sabarinathan R, Turuk A, Anand PK, Asthana S, Balachandar R, Bangar SD, Bansal AK, Bhat J, Chakraborty D, Rangaraju C, Chopra V, Das D, Deb AK, Devi KR, Dwivedi GR, Salim Khan SM, Haq I, Kumar MS, Laxmaiah A, Madhuka, Mahapatra A, Mitra A, Nirmala AR, Pagdhune A, Qurieshi MA, Ramarao T, Sahay S, Sharma YK, Shrinivasa MB, Shukla VK, Singh PK, Viramgami A, Wilson VC, Yadav R, Girish Kumar CP, Luke HE, Ranganathan UD, Babu S, Sekar K, Yadav PD, Sapkal GN, Das A, Das P, Dutta S, Hemalatha R, Kumar A, Narain K, Narasimhaiah S, Panda S, Pati S, Patil S, Sarkar K, Singh S, Kant R, Tripathy S, Toteja GS, Babu GR, Kant S, Muliyil JP, Pandey RM, Sarkar S, Singh SK, Zodpey S, Gangakhedkar RR, S Reddy DC, Bhargava B. Prevalence of SARS-CoV-2 infection in India: Findings from the national serosurvey, May-June 2020. Indian J Med Res. 2020; 152(1,2): 48-60.
India	Murhekar MV, Bhatnagar T, Selvaraju S, Saravanakumar V, Thangaraj JWV, Shah N, Kumar MS, Rade K, Sabarinathan R, Asthana S, Balachandar R, Bangar SD, Bansal AK, Bhat J, Chopra V, Das D, Deb AK, Devi KR, Dwivedi GR, Khan SMS, Kumar CPG, Kumar MS, Laxmaiah A, Madhukar M, Mahapatra A, Mohanty SS, Rangaraju C, Turuk A, Baradwaj DK, Chahal AS, Debnath F, Haq I, Kalliath A, Kanungo S, Kshatri JS, Lakshmi GGJN, Mitra A, Nirmala AR, Prasad GV, Qurieshi MA, Sahay S, Sangwan RK, Sekar K, Shukla VK, Singh PK, Singh P, Singh R, Varma DS, Viramgami A, Panda S, Reddy DCS, Bhargava B, ICMR Serosurveillance Group. SARS-CoV-2 antibody seroprevalence in India, August-September, 2020: findings from the second nationwide household serosurvey. Lancet Glob Health. 2021; 9(3): e257-e266.
India, Andhra Pradesh	Pandey A. Andhra Pradesh Sero Survey Show Nearly 20% of State's Population Exposed to Covid-19. India Today [Internet]. 2020 Sept 11.
India, Andhra Pradesh	Sadam R. 20% of Andhra Exposed to Covid, Sero-survey Finds, Exposure Highest Where Case Tally Lowest. ThePrint [Internet]. 2020 Sept 11.
India, Andhra Pradesh	Rao U. Andhra Pradesh: '59% found to have Covid antibodies in sero-survey'. The Times of India [Internet]. 2021 May 27; Visakhapatnam.
India, Andhra Pradesh	*
India, Andhra Pradesh	Mordani S. Seropositivity highest in Madhya Pradesh, lowest in Kerala, finds ICMR's national sero-survey. India Today [Internet]. 2021 Jul 28.
India, Assam	Pathak J, Das M, Siddique K. Assessment of Anti-SARS CoV-2 seroprevalence in habitants of Assam (AASSHA): report of the first serosurvey in Assam. Int J Community Med Public Health. 2021; 8(5): 2490-6.
India, Assam	*
India, Assam	Mordani S. Seropositivity highest in Madhya Pradesh, lowest in Kerala, finds ICMR's national sero-survey. India Today [Internet]. 2021 Jul 28.
India, Bihar	*
India, Bihar	Mordani S. Seropositivity highest in Madhya Pradesh, lowest in Kerala, finds ICMR's national sero-survey. India Today [Internet]. 2021 Jul 28.
India, Chhattisgarh	Outlook India. C"garh: 5.5 pc people have COVID-19 antibodies, says sero survey. Outlook India [Internet]. 2020 Oct 3.

India, Chhattisgarh	*
India, Chhattisgarh	Mordani S. Seropositivity highest in Madhya Pradesh, lowest in Kerala, finds ICMR's national sero-survey. India Today [Internet]. 2021 Jul 28.
India, Delhi	Express News Service. Press Briefing: Sero Survey Delhi. IndiaSpend [Internet]. 2020 Aug 20.
India, Delhi	Jain P. Over 56% of Delhi has Covid-19 Antibodies: Health Minister Satyendar Jain on 5th Serosurvey. India Today [Internet]. 2021 Feb 2.
India, Delhi	Dey S. Serial SARS-CoV-2 Seropravelence Studies in Delhi July-August 2020: Indications of Pre-existing Cross-reactive Antibodies and Implications for Disease Progression. Res Sq. 2020.
India, Delhi	Sharma N, Sharma P, Basu S, Saxena S, Chawla R, Dushyant K, Mundeja N, Marak ZS, Singh S, Singh GK, Rustagi R. The seroprevalence and trends of SARS-CoV-2 in Delhi, India: A repeated population-based seroepidemiological study. Preprint. medRxiv. 2020
India, Delhi	Press Trust of India. 29.1% people in Delhi developed antibodies against COVID-19, finds latest sero-survey. Firstpost [Internet]. 2020 Aug 20.
India, Goa	Naushin S, Sardana V, Ujjainiya R, et al. Insights from a Pan India Sero-Epidemiological survey (Phenome-India Cohort) for SARS-CoV2. Iqbal J, Zaidi M, Kanitkar M, Agrawal M, editors. eLife eLife Sciences Publications Ltd. 2021; 10: e66537.
India, Gujarat	*
India, Gujarat	Mordani S. Seropositivity highest in Madhya Pradesh, lowest in Kerala, finds ICMR's national sero-survey. India Today [Internet]. 2021 Jul 28.
India, Haryana	Health Department, Haryana (India). India - Haryana Covid -19 Sero Survey Round 1, August 2020. India: Health Department, Haryana (India).
India, Haryana	Health Department, Haryana (India). India - Haryana Covid-19 Sero Survey Round 2, October 2020.
India, Haryana	Mordani S. Seropositivity highest in Madhya Pradesh, lowest in Kerala, finds ICMR's national sero-survey. India Today [Internet]. 2021 Jul 28.
India, Himachal Pradesh	Mordani S. Seropositivity highest in Madhya Pradesh, lowest in Kerala, finds ICMR's national sero-survey. India Today [Internet]. 2021 Jul 28.
India, Himachal Pradesh	Naushin S, Sardana V, Ujjainiya R, et al. Insights from a Pan India Sero-Epidemiological survey (Phenome-India Cohort) for SARS-CoV2. Iqbal J, Zaidi M, Kanitkar M, Agrawal M, editors. eLife eLife Sciences Publications Ltd. 2021; 10: e66537.
India, Jammu & Kashmir and Ladakh	Mordani S. Seropositivity highest in Madhya Pradesh, lowest in Kerala, finds ICMR's national sero-survey. India Today [Internet]. 2021 Jul 28.
India, Jharkhand	Phase 2 of Sero Survey Begins in Latehar, Simdega, Pakur. Telegraph [Internet]. 2020 Aug 20.
India, Jharkhand	*
India, Jharkhand	Mordani S. Seropositivity highest in Madhya Pradesh, lowest in Kerala, finds ICMR's national sero-survey. India Today [Internet]. 2021 Jul 28.
India, Jharkhand	The Times of India. Jharkhand: 45% of population in 10 districts positive before 2nd wave: Sero survey . Mumbai, India: The Times of India, 2021.
India, Karnataka	*
India, Karnataka	Mordani S. Seropositivity highest in Madhya Pradesh, lowest in Kerala, finds ICMR's national sero-survey. India Today [Internet]. 2021 Jul 28.
India, Karnataka	Babu GR, Sundaresan R, Athreya S, Akhtar J, Pandey PK, Maroor PS, Padma MR, Lalitha R, Shariff M, Krishnappa L, Manjunath CN, Sudarshan MK, Gururaj G, Ranganath TS, Kumar V, Banandur P, Deepa R, Shiju S, Lobo E, Satapathy A, Alahari L, Prameela, Vinitha T, Desai A, Ravi V. The burden of active infection and anti-SARS-CoV-2 IgG antibodies in the general population: Results from a statewide survey in Karnataka, India. medRxiv. 2020.

India, Karnataka	Mohanan M, Malani A, Krishnan K, Acharya A. Prevalence of SARS-CoV-2 in Karnataka, India. JAMA. 2021; 325(10): 1001-3.
India, Kerala	Department of Health and Family Welfare, Government of Kerala (India). India - Kerala COVID-19 ICMR Sero Surveillance 2nd Round August 2020.
India, Kerala	*
India, Kerala	Mordani S. Seropositivity highest in Madhya Pradesh, lowest in Kerala, finds ICMR's national sero-survey. India Today [Internet]. 2021 Jul 28.
India, Madhya Pradesh	*
India, Madhya Pradesh	Mordani S. Seropositivity highest in Madhya Pradesh, lowest in Kerala, finds ICMR's national sero-survey. India Today [Internet]. 2021 Jul 28.
India, Maharashtra	*
India, Maharashtra	Mordani S. Seropositivity highest in Madhya Pradesh, lowest in Kerala, finds ICMR's national sero-survey. India Today [Internet]. 2021 Jul 28.
India, Odisha	*
India, Odisha	Mordani S. Seropositivity highest in Madhya Pradesh, lowest in Kerala, finds ICMR's national sero-survey. India Today [Internet]. 2021 Jul 28.
India, Punjab	*
India, Punjab	Mordani S. Seropositivity highest in Madhya Pradesh, lowest in Kerala, finds ICMR's national sero-survey. India Today [Internet]. 2021 Jul 28.
India, Rajasthan	*
India, Rajasthan	Mordani S. Seropositivity highest in Madhya Pradesh, lowest in Kerala, finds ICMR's national sero-survey. India Today [Internet]. 2021 Jul 28.
India, Tamil Nadu	*
India, Tamil Nadu	Mordani S. Seropositivity highest in Madhya Pradesh, lowest in Kerala, finds ICMR's national sero-survey. India Today [Internet]. 2021 Jul 28.
India, Tamil Nadu	Hindustan Times (India). TN serosurvey finds Covid antibodies in 23% population. India: Hindustan Times (India), 2021.
India, Tamil Nadu	Malani A, Ramachandran S, Tandel V, Parasa R, Sudharshini S, Prakash V, Yogananth Y, Raju S, Selvavinayagam TS. SARS-CoV-2 Seroprevalence in Tamil Nadu in October-November 2020. Preprint. medRxiv. 2021.
India, Telangana	*
India, Telangana	Mordani S. Seropositivity highest in Madhya Pradesh, lowest in Kerala, finds ICMR's national sero-survey. India Today [Internet]. 2021 Jul 28.
India, Tripura	Deb D. 33.98% of Tripura has COVID-19 antibody in them, shows sero survey. The Indian Express [Internet]. 2020 Nov 12; North East India.
India, Uttar Pradesh	*
India, Uttar Pradesh	Mordani S. Seropositivity highest in Madhya Pradesh, lowest in Kerala, finds ICMR's national sero-survey. India Today [Internet]. 2021 Jul 28.
India, Uttarakhand	Mordani S. Seropositivity highest in Madhya Pradesh, lowest in Kerala, finds ICMR's national sero-survey. India Today [Internet]. 2021 Jul 28.
India, West Bengal	Kolkata: One in Four People Have Covid Antibodies. The Statesman [Internet]. 2020 Sept 10.
India, West Bengal	Gautham, K. One in Three Exposed to Covid Virus, Reveals Second Chennai Sero Survey. The Times of India [Internet]. 2020 Oct 22.
India, West Bengal	*

India, West Bengal	Mordani S. Seropositivity highest in Madhya Pradesh, lowest in Kerala, finds ICMR's national sero-survey. India Today [Internet]. 2021 Jul 28.
Iran	Khalagi K, Gharibzadeh S, Khalili D, Mansournia MA, Mirab Samiee S, Aghamohamadi S, Mir-Mohammad-Ali Roodaki M, Hashemi SM, Tayeri K, Namdari Tabar H, Azadmanesh K, Tabrizi JS, Mohammad K, Hajipour F, Namaki S, Raeisi A, Ostovar A. Prevalence of COVID-19 in Iran: results of the first survey of the Iranian COVID-19 Serological Surveillance programme. Clin Microbiol Infect. 2021.
Ireland	Health Service Executive (HSE) (Ireland), University College Dublin. Ireland Study to Investigate COVID-19 Infection in People Living in Ireland (SCOPI) 2020. Dublin, Ireland: Health Protection Surveillance Centre (HPSC) (Ireland), 2020.
Israel	Ministry of Health (Israel). Israel - Press Releases Results of the National Serological Survey for Novel Coronavirus. Jerusalem, Israel: Ministry of Health (Israel), 2020.
Israel	Reicher S, Ratzon R, Ben-Sahar S, Hermoni-Alon S, Mossinson D, Shenhar Y, Friger M, Lustig Y, Alroy-Preis S, Anis E, Sadetzki S, Kaliner E. Nationwide seroprevalence of antibodies against SARS-CoV-2 in Israel. Eur J Epidemiol. 2021.
Italy	Ministry of Health (Italy), National Institute of Statistics (Italy). Italy SARS-CoV-2 Seroprevalence Survey, First Results 2020.
Italy, Abruzzo	Ministry of Health (Italy), National Institute of Statistics (Italy). Italy SARS-CoV-2 Seroprevalence Survey, First Results 2020.
Italy, Basilicata	Ministry of Health (Italy), National Institute of Statistics (Italy). Italy SARS-CoV-2 Seroprevalence Survey, First Results 2020.
Italy, Calabria	Ministry of Health (Italy), National Institute of Statistics (Italy). Italy SARS-CoV-2 Seroprevalence Survey, First Results 2020.
Italy, Campania	Ministry of Health (Italy), National Institute of Statistics (Italy). Italy SARS-CoV-2 Seroprevalence Survey, First Results 2020.
Italy, Emilia-Romagna	Ministry of Health (Italy), National Institute of Statistics (Italy). Italy SARS-CoV-2 Seroprevalence Survey, First Results 2020.
Italy, Friuli-Venezia Giulia	Ministry of Health (Italy), National Institute of Statistics (Italy). Italy SARS-CoV-2 Seroprevalence Survey, First Results 2020.
Italy, Lazio	Ministry of Health (Italy), National Institute of Statistics (Italy). Italy SARS-CoV-2 Seroprevalence Survey, First Results 2020.
Italy, Liguria	Ministry of Health (Italy), National Institute of Statistics (Italy). Italy SARS-CoV-2 Seroprevalence Survey, First Results 2020.
Italy, Lombardia	Ministry of Health (Italy), National Institute of Statistics (Italy). Italy SARS-CoV-2 Seroprevalence Survey, First Results 2020.
Italy, Marche	Ministry of Health (Italy), National Institute of Statistics (Italy). Italy SARS-CoV-2 Seroprevalence Survey, First Results 2020.
Italy, Molise	Ministry of Health (Italy), National Institute of Statistics (Italy). Italy SARS-CoV-2 Seroprevalence Survey, First Results 2020.
Italy, Piemonte	Ministry of Health (Italy), National Institute of Statistics (Italy). Italy SARS-CoV-2 Seroprevalence Survey, First Results 2020.
Italy, Prov. autonoma di Bolzano	Ministry of Health (Italy), National Institute of Statistics (Italy). Italy SARS-CoV-2 Seroprevalence Survey, First Results 2020.
Italy, Prov. autonoma di Trento	Ministry of Health (Italy), National Institute of Statistics (Italy). Italy SARS-CoV-2 Seroprevalence Survey, First Results 2020.
Italy, Puglia	Ministry of Health (Italy), National Institute of Statistics (Italy). Italy SARS-CoV-2 Seroprevalence Survey, First Results 2020.
Italy, Sardegna	Ministry of Health (Italy), National Institute of Statistics (Italy). Italy SARS-CoV-2 Seroprevalence Survey, First Results 2020.
Italy, Sicilia	Ministry of Health (Italy), National Institute of Statistics (Italy). Italy SARS-CoV-2 Seroprevalence Survey, First Results 2020.

Italy, Toscana	Ministry of Health (Italy), National Institute of Statistics (Italy). Italy SARS-CoV-2 Seroprevalence Survey, First Results 2020.
Italy, Umbria	Ministry of Health (Italy), National Institute of Statistics (Italy). Italy SARS-CoV-2 Seroprevalence Survey, First Results 2020.
Italy, Valle d'Aosta	Ministry of Health (Italy), National Institute of Statistics (Italy). Italy SARS-CoV-2 Seroprevalence Survey, First Results 2020.
Italy, Veneto	Ministry of Health (Italy), National Institute of Statistics (Italy). Italy SARS-CoV-2 Seroprevalence Survey, First Results 2020.
Jordan	Bellizzi S, Alsawalha L, Sheikh Ali S, Sharkas G, Muthu N, Ghazo M, Hayajneh W, Profili MC, Obeidat NM. A three-phase population based sero-epidemiological study: Assessing the trend in prevalence of SARS-CoV-2 during COVID-19 pandemic in Jordan. One Health. 2021; 13: 100292.
Jordan	Sughayer MA, Mansour A, Nuirat AA, Souan L, Ghanem M, Siag M, Alhassoon S. Dramatic Rise of Seroprevalence Rates of SARS-CoV-2 Antibodies among Healthy Blood Donors: The evolution of a Pandemic. Preprint. medRxiv. 2021.
Jordan	Sughayer MA, Mansour A, Nuirat AA, Souan L, Abdel-Razeq R, Siag M. A second dramatic rise in seroprevalence rates of SARS-CoV-2 antibodies among adult healthy blood donors in Jordan; have we achieved herd immunity? Preprint. medRxiv. 2021.
Kazakhstan	*
Kenya	Uyoga S, Adetifa IMO, Karanja HK, Nyagwange J, Tuju J, Wanjiku P, Aman R, Mwangangi M, Amoth P, Kasera K, Ng'ang'a W, Rombo C, Yegon CK, Kithi K, Odhiambo E, Rotich T, Orgut I, Kihara S, Otiende M, Bottomley C, Mupe ZN, Kagucia EW, Gallagher K, Etyang A, Voller S, Gitonga J, Mugo D, Agoti CN, Otieno E, Ndwiga L, Lambe T, Wright D, Barasa E, Tsofa B, Bejon P, Ochola-Oyier LI, Agweyu A, Scott AGJ, Warimwe GM. Seroprevalence of anti-SARS-CoV-2 IgG antibodies in Kenyan blood donors. Preprint. medRxiv. 2020.
Kenya	Adetifa IMO, Uyoga S, Gitonga JN, Mugo D, Otiende M, Nyagwange J, Karanja HK, Tuju J, Wanjiku P, Aman R, Mwangangi M, Amoth P, Kasera K, Nganga W, Rombo C, Yegon C, Kithi K, Odhiambo E, Rotich T, Orgut I, Kihara S, Bottomley C, Kagucia EW, Gallagher KE, Etyang A, Voller S, Lambe T, Wright D, Barasa E, Tsofa B, Bejon P, Ochola-Oyier LI, Agweyu A, Scott JAG, Warimwe GM. Temporal trends of SARS-CoV-2 seroprevalence in transfusion blood donors during the first wave of the COVID-19 epidemic in Kenya. medRxiv. 2021.
Kenya	Uyoga S, Adetifa IMO, Otiende M, Gitonga JN, Mugo D, Nyagwange J, Karanja HK, Tuju J, Makale J, Aman R, Mwangangi M, Amoth P, Kasera K, Nganga W, Kilonzo N, Chege E, Yegon C, Odhiambo E, Rotich T, Orgut I, Kihara S, Bottomley C, Kagucia EW, Gallagher KE, Etyang A, Voller S, Lambe T, Wright D, Barasa E, Tsofa B, Mwangangi J, Bejon P, Ochola-Oyier LI, Warimwe GM, Agweyu A, Scott JAG. Prevalence of SARS-CoV-2 Antibodies from a one-year National Serosurveillance of Kenyan Blood Transfusion Donors. Preprint. medRxiv. 2021.
Kenya	Ngere IA, Dawa J, Hunsperger E, Otieno N, Masika M, Amoth P, Makayotto L, Nasimiyu C, Gunn BM, Nyawanda B, Oluga O, Ngunu C, Mirieri H, Gachohi J, Marwanga D, Munywoki P, Odhiambo D, Alando MD, Breiman R, Anzala O, Njenga MK, Bulterys M, Herman-Roloff A, Osoro E. High Seroprevalence of SARS-CoV-2 Eight Months After Introduction in Nairobi, Kenya. Preprint. Lancet. 2021.
Luxembourg	Snoeck CJ, Vaillant M, Abdelrahman T, Satagopam VP, Turner JD, Beaumont K, Gomes CPC, Fritz JV, Schröder VE, Kaysen A, Pavelka L, Stute L, Meyers GR, Pauly L, Hansen M, Pauly C, Aguayo GA, Perquin M, Hanff AM, Ghosh S, Gantenbein M, Huiart L, Ollert M, Krüger R. Prevalence of SARS-CoV-2 infection in the Luxembourgish population: the CON-VINCE study. Preprint. medRxiv. 2020.
Mexico	Health Secretary, National Institute of Public Health (Mexico). Preliminary results of the National Health and Nutrition Survey COVID-19. Communiqué 255, 2020 Dec 16.
Mexico	Muñoz-Medina JE, Grajales-Muñiz C, Salas-Lais AG, Fernandes-Matano L, López-Macías C, Monroy-Muñoz IE, Santos Coy-Arechavaleta A, Palomec-Nava ID, Duque-Molina C, Madera-Sandoval RL, Rivero-Arredondo V, González-Ibarra J, Alvarado-Yaah JE, Rojas-Mendoza T, Santacruz-Tinoco CE, González-Bonilla CR, Borja-Aburto VH. SARS-CoV-2 IgG Antibodies

	Seroprevalence and Sera Neutralizing Activity in MEXICO: A National Cross-Sectional Study during 2020. Microorganisms. 2021; 9(4).
Mexico, Aguascalientes	Government of Mexico, National Institute of Public Health (Mexico). Mexico National Health and Nutrition Survey (ENSANUT) about COVID-19 2020. Cuernavaca, Mexico: National Institute of Public Health (Mexico), 2020.
Mexico, Baja California	Government of Mexico, National Institute of Public Health (Mexico). Mexico National Health and Nutrition Survey (ENSANUT) about COVID-19 2020. Cuernavaca, Mexico: National Institute of Public Health (Mexico), 2020.
Mexico, Baja California Sur	Government of Mexico, National Institute of Public Health (Mexico). Mexico National Health and Nutrition Survey (ENSANUT) about COVID-19 2020. Cuernavaca, Mexico: National Institute of Public Health (Mexico), 2020.
Mexico, Campeche	Government of Mexico, National Institute of Public Health (Mexico). Mexico National Health and Nutrition Survey (ENSANUT) about COVID-19 2020. Cuernavaca, Mexico: National Institute of Public Health (Mexico), 2020.
Mexico, Chiapas	Government of Mexico, National Institute of Public Health (Mexico). Mexico National Health and Nutrition Survey (ENSANUT) about COVID-19 2020. Cuernavaca, Mexico: National Institute of Public Health (Mexico), 2020.
Mexico, Chihuahua	Government of Mexico, National Institute of Public Health (Mexico). Mexico National Health and Nutrition Survey (ENSANUT) about COVID-19 2020. Cuernavaca, Mexico: National Institute of Public Health (Mexico), 2020.
Mexico, Coahuila	Government of Mexico, National Institute of Public Health (Mexico). Mexico National Health and Nutrition Survey (ENSANUT) about COVID-19 2020. Cuernavaca, Mexico: National Institute of Public Health (Mexico), 2020.
Mexico, Colima	Government of Mexico, National Institute of Public Health (Mexico). Mexico National Health and Nutrition Survey (ENSANUT) about COVID-19 2020. Cuernavaca, Mexico: National Institute of Public Health (Mexico), 2020.
Mexico, Durango	Government of Mexico, National Institute of Public Health (Mexico). Mexico National Health and Nutrition Survey (ENSANUT) about COVID-19 2020. Cuernavaca, Mexico: National Institute of Public Health (Mexico), 2020.
Mexico, Guanajuato	Government of Mexico, National Institute of Public Health (Mexico). Mexico National Health and Nutrition Survey (ENSANUT) about COVID-19 2020. Cuernavaca, Mexico: National Institute of Public Health (Mexico), 2020.
Mexico, Guerrero	Government of Mexico, National Institute of Public Health (Mexico). Mexico National Health and Nutrition Survey (ENSANUT) about COVID-19 2020. Cuernavaca, Mexico: National Institute of Public Health (Mexico), 2020.
Mexico, Hidalgo	Government of Mexico, National Institute of Public Health (Mexico). Mexico National Health and Nutrition Survey (ENSANUT) about COVID-19 2020. Cuernavaca, Mexico: National Institute of Public Health (Mexico), 2020.
Mexico, Jalisco	Government of Mexico, National Institute of Public Health (Mexico). Mexico National Health and Nutrition Survey (ENSANUT) about COVID-19 2020. Cuernavaca, Mexico: National Institute of Public Health (Mexico), 2020.
Mexico, México	Government of Mexico, National Institute of Public Health (Mexico). Mexico National Health and Nutrition Survey (ENSANUT) about COVID-19 2020. Cuernavaca, Mexico: National Institute of Public Health (Mexico), 2020.
Mexico, Mexico City	Government of Mexico, National Institute of Public Health (Mexico). Mexico National Health and Nutrition Survey (ENSANUT) about COVID-19 2020. Cuernavaca, Mexico: National Institute of Public Health (Mexico), 2020.
Mexico, Michoacán de Ocampo	Government of Mexico, National Institute of Public Health (Mexico). Mexico National Health and Nutrition Survey (ENSANUT) about COVID-19 2020. Cuernavaca, Mexico: National Institute of Public Health (Mexico), 2020.
Mexico, Morelos	Government of Mexico, National Institute of Public Health (Mexico). Mexico National Health and Nutrition Survey (ENSANUT) about COVID-19 2020. Cuernavaca, Mexico: National Institute of Public Health (Mexico), 2020.

Mexico, Nayarit	Government of Mexico, National Institute of Public Health (Mexico). Mexico National Health and Nutrition Survey (ENSANUT) about COVID-19 2020. Cuernavaca, Mexico: National Institute of Public Health (Mexico), 2020.
Mexico, Nuevo León	Government of Mexico, National Institute of Public Health (Mexico). Mexico National Health and Nutrition Survey (ENSANUT) about COVID-19 2020. Cuernavaca, Mexico: National Institute of Public Health (Mexico), 2020.
Mexico, Oaxaca	Government of Mexico, National Institute of Public Health (Mexico). Mexico National Health and Nutrition Survey (ENSANUT) about COVID-19 2020. Cuernavaca, Mexico: National Institute of Public Health (Mexico), 2020.
Mexico, Puebla	Government of Mexico, National Institute of Public Health (Mexico). Mexico National Health and Nutrition Survey (ENSANUT) about COVID-19 2020. Cuernavaca, Mexico: National Institute of Public Health (Mexico), 2020.
Mexico, Querétaro	Government of Mexico, National Institute of Public Health (Mexico). Mexico National Health and Nutrition Survey (ENSANUT) about COVID-19 2020. Cuernavaca, Mexico: National Institute of Public Health (Mexico), 2020.
Mexico, Quintana Roo	Government of Mexico, National Institute of Public Health (Mexico). Mexico National Health and Nutrition Survey (ENSANUT) about COVID-19 2020. Cuernavaca, Mexico: National Institute of Public Health (Mexico), 2020.
Mexico, San Luis Potosí	Government of Mexico, National Institute of Public Health (Mexico). Mexico National Health and Nutrition Survey (ENSANUT) about COVID-19 2020. Cuernavaca, Mexico: National Institute of Public Health (Mexico), 2020.
Mexico, Sinaloa	Government of Mexico, National Institute of Public Health (Mexico). Mexico National Health and Nutrition Survey (ENSANUT) about COVID-19 2020. Cuernavaca, Mexico: National Institute of Public Health (Mexico), 2020.
Mexico, Sonora	Government of Mexico, National Institute of Public Health (Mexico). Mexico National Health and Nutrition Survey (ENSANUT) about COVID-19 2020. Cuernavaca, Mexico: National Institute of Public Health (Mexico), 2020.
Mexico, Tabasco	Government of Mexico, National Institute of Public Health (Mexico). Mexico National Health and Nutrition Survey (ENSANUT) about COVID-19 2020. Cuernavaca, Mexico: National Institute of Public Health (Mexico), 2020.
Mexico, Tamaulipas	Government of Mexico, National Institute of Public Health (Mexico). Mexico National Health and Nutrition Survey (ENSANUT) about COVID-19 2020. Cuernavaca, Mexico: National Institute of Public Health (Mexico), 2020.
Mexico, Tlaxcala	Government of Mexico, National Institute of Public Health (Mexico). Mexico National Health and Nutrition Survey (ENSANUT) about COVID-19 2020. Cuernavaca, Mexico: National Institute of Public Health (Mexico), 2020.
Mexico, Veracruz de Ignacio de la Llave	Government of Mexico, National Institute of Public Health (Mexico). Mexico National Health and Nutrition Survey (ENSANUT) about COVID-19 2020. Cuernavaca, Mexico: National Institute of Public Health (Mexico), 2020.
Mexico, Yucatán	Government of Mexico, National Institute of Public Health (Mexico). Mexico National Health and Nutrition Survey (ENSANUT) about COVID-19 2020. Cuernavaca, Mexico: National Institute of Public Health (Mexico), 2020.
Mexico, Zacatecas	Government of Mexico, National Institute of Public Health (Mexico). Mexico National Health and Nutrition Survey (ENSANUT) about COVID-19 2020. Cuernavaca, Mexico: National Institute of Public Health (Mexico), 2020.
Monaco	La Gazette de Monaco. Covid 19: around 35 000 people have been tested. La Gazette de Monaco [Internet]. 2020 Jun 15; Politics.
Mozambique	Ministry of Health (Mozambique). Serum-epidemiological Survey of SARS-CoV-2, City of Pemba (InCOVID 2020) - Preliminary Results. Maputo, Mozambique: Ministry of Health (Mozambique).
Mozambique	Ministry of Health (Mozambique). Serum-epidemiological Survey of SARS-CoV-2, City of Quelimane (InCOVID 2020) - Preliminary Results. Maputo, Mozambique: Ministry of Health (Mozambique).

Mozambique	Ministry of Health (Mozambique). Serum-epidemiological Survey of SARS-CoV-2, City of Maputo (InCOVID 2020) - Preliminary Results. Maputo, Mozambique: Ministry of Health (Mozambique).
Mozambique	Ministry of Health (Mozambique). Serum-epidemiological Survey of SARS-CoV-2, City of Beira (InCOVID 2020) - Preliminary Results. Maputo, Mozambique: Ministry of Health (Mozambique).
Mozambique	Ministry of Health (Mozambique). Serum-epidemiological Survey of SARS-CoV-2, City of Lichinga (InCOVID 2020) - Preliminary Results. Maputo, Mozambique: Ministry of Health (Mozambique).
Mozambique	Ministry of Health (Mozambique). Serum-epidemiological Survey of SARS-CoV-2, City of Tete (InCOVID 2020) - Preliminary Results. Maputo, Mozambique: Ministry of Health (Mozambique).
Mozambique	Ministry of Health (Mozambique). Serum-epidemiological Survey of SARS-CoV-2, Cities of Xai-Xai and Chókwè (InCOVID 2020) - Preliminary Results. Maputo, Mozambique: Ministry of Health (Mozambique).
Mozambique	Ministry of Health (Mozambique). Serum-epidemiological Survey of SARS-CoV-2, City of Chimoio (InCOVID 2020) - Preliminary Results . Maputo, Mozambique: Ministry of Health (Mozambique).
Nepal	Ministry of Health and Population (Nepal), National Public Health Laboratory (Nepal), World Health Organization (WHO), World Health Organization Regional Office for South-East Asia (SEARO). Nepal National Sero-Prevalence Survey for COVID-19 October 2020.
Netherlands	Vos ERA, den Hartog G, Schepp RM, Kaaijk P, van Vliet J, Helm K, Smits G, Wijmenga-Monsuur A, Verberk JDM, van Boven M, van Binnendijk RS, de Melker HE, Mollema L, van der Klis FRM. Nationwide seroprevalence of SARS-CoV-2 and identification of risk factors in the general population of the Netherlands during the first epidemic wave. J Epidemiol Community Health. 2020.
Netherlands	Vos ERA, van Boven M, den Hartog G, Backer JA, Klinkenberg D, van Hagen CCE, Boshuizen H, van Binnendijk RS, Mollema L, van der Klis FRM, de Melker HE. Associations between measures of social distancing and SARS-CoV-2 seropositivity: a nationwide population-based study in the Netherlands. Clin Infect Dis. 2021.
Netherlands	Slot E, Hogema BM, Reusken CBEM, Reimerink JH, Molier M, Karregat JHM, IJlst J, Novotný VMJ, van Lier RAW, Zaaijer HL. Low SARS-CoV-2 seroprevalence in blood donors in the early COVID-19 epidemic in the Netherlands. Nat Commun. 2020; 11(1): 5744.
Netherlands	Sanquin (Netherlands). Netherlands - Coronavirus antibodies in an average of 18.6% of the donors. Sanquin (Netherlands) [Internet]. 17 Mar 2021; News.
New Zealand	Carlton LH, Chen T, Whitcombe AL, McGregor R, Scheurich G, Sheen CR, Dickson JM, Bullen C, Chiang A, Exeter DJ, Paynter J, Baker MG, Charlewood R, Moreland NJ. Charting Elimination in the Pandemic: A SARS-CoV-2 Serosurvey of Blood Donors in New Zealand. Preprint. medRxiv. 2021.
Nigeria	Nigeria Centre for Disease Control. Federal Ministry of Health Implements Massive Distribution of Personal Protective Equipment to Primary Healthcare Centres in Nigeria. Nigeria Centre for Disease Control [Internet]. 28 May 2021; News.
Norway	Norwegian Institute of Public Health. Seroprevalence of SARS-CoV-2 in the Norwegian population measured in residual sera collected in April/May 2020 and August 2019. Oslo, Norway: Norwegian Institute of Public Health, 2020.
Norway	Tunheim G., Kran, AB., Rø, G., Hungnes O., Lund-Johansen, F., Tran, T., Andersen JT., Vaage, JT. "Seroprevalence of SARS-CoV-2 in the Norwegian population measured in residual sera collected in late summer 2020". [Seroprevalens av SARS-CoV-2 i den norske befolkningen, målt i restsera samlet inn på sensommeren 2020] Report 2020. Oslo: Norwegian Institute of Public Health, 2020.
Norway	Anda EE, Braaten T, Borch KB, Nøst TH, Chen SLF, Lukic M, Lund E, Forland F, Leon D, Winje BA, Kran A-MB, Kalager M, Johansen FL, Sandanger TM. Seroprevalence of antibodies against SARS-CoV-2 virus in the adult Norwegian population, winter 2020/2021: pre-vaccination period. Preprint. medRxiv. 2021.

Oman	Ministry of Health (Oman). Oman COVID-19 National Serological Survey Phase 1 2020.
Pakistan, Khyber Pakhtunkhwa	Haq M, Rehman A, Ahmad J, Zafar U, Ahmed S, Khan MA, Naveed A, Rajab H, Muhammad F, Naushad W, Aman M, Rehman HU, Ahmad S, Anwar S, Haq NU. SARS-CoV-2: big seroprevalence data from Pakistan-is herd immunity at hand?. Infection. 2021.
Pakistan, Punjab	Haq M, Rehman A, Ahmad J, Zafar U, Ahmed S, Khan MA, Naveed A, Rajab H, Muhammad F, Naushad W, Aman M, Rehman HU, Ahmad S, Anwar S, Haq NU. SARS-CoV-2: big seroprevalence data from Pakistan-is herd immunity at hand?. Infection. 2021.
Pakistan, Sindh	Haq M, Rehman A, Ahmad J, Zafar U, Ahmed S, Khan MA, Naveed A, Rajab H, Muhammad F, Naushad W, Aman M, Rehman HU, Ahmad S, Anwar S, Haq NU. SARS-CoV-2: big seroprevalence data from Pakistan-is herd immunity at hand?. Infection. 2021.
Peru	Huamaní C, Velásquez L, Montes S, Mayanga-Herrera A, Bernabé-Ortiz A. Population-based seroprevalence of SARS-CoV-2 antibodies in a high-altitude setting in Peru. Preprint.medRxiv. 2021.
Poland	Zejda JE, Brożek GM, Kowalska M, Barański K, Kaleta-Pilarska A, Nowakowski A, Xia Y, Buszman P. Seroprevalence of Anti-SARS-CoV-2 Antibodies in a Random Sample of Inhabitants of the Katowice Region, Poland. Int J Environ Res Public Health. 2021; 18(6).
Portugal	Ministry of Health (Portugal). Portugal National Serological Survey COVID-19 Preliminary results 2020. Lisbon, Portugal: Ministry of Health (Portugal), 2020.
Portugal	Ministry of Health (Portugal). Portugal National Serological Survey COVID-19 (2nd phase) 2021. Lisbon, Portugal: Ministry of Health (Portugal), 2020.
Portugal	Castro L, Gomes A, et al. Longitudinal SARS-CoV-2 seroprevalence in Portugal and antibody maintenance 12 months after the start of the COVID-19 pandemic. Preprint. Res Sq. 2021.
Puerto Rico	Puerto Rico Public Health Trust (PRPHT). Puerto Rico Community Assessment for Response to Public Health Emergencies (CASPER) 2020.
Puerto Rico	Jones JM, Stone M, Sulaeman H, Fink RV, Dave H, Levy ME, Di Germanio C, Green V, Notari E, Saa P, Biggerstaff BJ, Strauss D, Kessler D, Vassallo R, Reik R, Rossmann S, Destree M, Nguyen KA, Sayers M, Lough C, Bougie DW, Ritter M, Latoni G, Weales B, Sime S, Gorlin J, Brown NE, Gould CV, Berney K, Benoit TJ, Miller MJ, Freeman D, Kartik D, Fry AM, Azziz-Baumgartner E, Hall AJ, MacNeil A, Gundlapalli AV, Basavaraju SV, Gerber SI, Patton ME, Custer B, Williamson P, Simmons G, Thornburg NJ, Kleinman S, Stramer SL, Opsomer J, Busch MP. Estimated US Infection- and Vaccine-Induced SARS-CoV-2 Seroprevalence Based on Blood Donations, July 2020-May 2021. JAMA. 2021.
Puerto Rico	Centers for Disease Control and Prevention (CDC). CDC COVID Data Tracker Commercial Laboratory Seroprevalence Survey Data. Atlanta, United States of America: Centers for Disease Control and Prevention (CDC).
Qatar	Abu-Raddad LJ, Chemaitelly H, Ayoub HH, Al Kanaani Z, Al Khal A, Al Kuwari E, Butt AA, Coyle P, Jeremijenko A, Kaleeckal AH, Latif AN, Owen RC, Rahim HFA, Al Abdulla SA, Al Kuwari MG, Kandy MC, Saeb H, Ahmed SNN, Al Romaihi HE, Bansal D, Dalton L, Al-Thani MH, Bertollini R. Characterizing the Qatar advanced-phase SARS-CoV-2 epidemic. Sci Rep. 2021; 11(1): 6233.
Russia	Popova AY, Andreeva EE, Babura EA, Balakhonov SV, Bashketova NS, Bulanov MV, Valeullina NN, Goryaev DV, Detkovskaya NN, Ezhlova EB, Zaitseva NN, Istorik OA, Kovalchuk IV, Kozlovskikh DN, Kombarova SV, Kurganova OP, Kutyrev VV, Lomovtsev AE, Lukicheva LA, Lyalina LV, Melnikova AA, Mikailova OM, Noskov AK, Noskova LN, Oglezneva EE, Osmolovskay TP, Patyashina MA, Penkovskaya NA, Samoilova LV, Smirnov VS, Stepanova TF, Trotsenko OE, Totolyan AA. [Features of developing SARS-CoV-2 nucleocapsid protein population-based seroprevalence during the first wave of the COVID-19 epidemic in the Russian Federation]. Infektsiia Immun. 2021; 11(2): 297-323.
Russia	Interfax. Popova declared immunity to coronavirus in 14% of those tested. Interfax [Internet]. 2020 Jun 10.
Sierra Leone	Barrie MB, Lakoh S, Kelly JD, Kanu JS, Squire J, Koroma Z, Bah S, Sankoh O, Brima A, Ansumana R, Goldberg SA, Chitre S, Osuagwu C, Maeda J, Barekye B, Numbere T-W, Abdulaziz M, Mounts A, Blanton C, Singh T, Samai M, Vandi MA, Richardson ET. SARS-

	CoV-2 antibody prevalence in Sierra Leone, March 2021: a cross-sectional, nationally representative, age-stratified serosurvey. Preprint. medRxiv. 2021.
Slovenia	Poljak M, Oštrbenk Valenčak A, Štrumbelj E, Maver Vodičar P, Vehovar V, Resman Rus K, Korva M, Knap N, Seme K, Petrovec M, Zupan B, Demšar J, Kurdija S, Avšič Županc T. Seroprevalence of severe acute respiratory syndrome coronavirus 2 in Slovenia: results of two rounds of a nationwide population study on a probability-based sample, challenges and lessons learned. Clin Microbiol Infect. 2021.
South Africa	Sykes W, Mhlanga L, Swanevelder R, Glatt TN, Grebe E, Coleman C, Pieterson N, Cable R, Welte A, van den Berg K, Vermeulen M. Prevalence of anti-SARS-CoV-2 antibodies among blood donors in Northern Cape, KwaZulu-Natal, Eastern Cape, and Free State provinces of South Africa in January 2021. Preprint. Res Sq. 2021.
South Korea	Lee K, Jo S, Lee J. Seroprevalence of SARS-CoV-2 antibodies in South Korea. J Korean Stat Soc. 2021; 1-14.
South Korea	Nah EH, Cho S, Park H, Hwang I, Cho HI. Nationwide seroprevalence of antibodies to SARS-CoV-2 in asymptomatic population in South Korea: a cross-sectional study. BMJ Open. 2021; 11(4): e049837.
Spain	Government of Spain, Institute of Health Carlos III (Spain). Spain - National Study of Sero-epidemiology of Infection by Sars-Cov-2.
Spain, Andalusia	Government of Spain, Institute of Health Carlos III (Spain). Spain - National Study of Sero-epidemiology of Infection by Sars-Cov-2.
Spain, Aragon	Government of Spain, Institute of Health Carlos III (Spain). Spain - National Study of Sero-epidemiology of Infection by Sars-Cov-2.
Spain, Asturias	Government of Spain, Institute of Health Carlos III (Spain). Spain - National Study of Sero-epidemiology of Infection by Sars-Cov-2.
Spain, Balearic Islands	Government of Spain, Institute of Health Carlos III (Spain). Spain - National Study of Sero-epidemiology of Infection by Sars-Cov-2.
Spain, Basque Country	Government of Spain, Institute of Health Carlos III (Spain). Spain - National Study of Sero-epidemiology of Infection by Sars-Cov-2.
Spain, Canary Islands	Government of Spain, Institute of Health Carlos III (Spain). Spain - National Study of Sero-epidemiology of Infection by Sars-Cov-2.
Spain, Cantabria	Iruzubieta P, Fernández-Lanas T, Rasines L, Cayon L, Álvarez-Cancelo A, Santos-Laso A, García-Blanco A, Curiel-Olmo S, Cabezas J, Wallmann R, Fábrega E, Martínez-Taboada VM, Hernández JL, López-Hoyos M, Lazarus JV, Crespo J. Feasibility of large-scale population testing for SARS-CoV-2 detection by self-testing at home. Sci Rep. 2021; 11(1): 9819.
Spain, Cantabria	Government of Spain, Institute of Health Carlos III (Spain). Spain - National Study of Sero-epidemiology of Infection by Sars-Cov-2.
Spain, Castile and León	Government of Spain, Institute of Health Carlos III (Spain). Spain - National Study of Sero-epidemiology of Infection by Sars-Cov-2.
Spain, Castilla–La Mancha	Government of Spain, Institute of Health Carlos III (Spain). Spain - National Study of Sero-epidemiology of Infection by Sars-Cov-2.
Spain, Catalonia	Government of Spain, Institute of Health Carlos III (Spain). Spain - National Study of Sero-epidemiology of Infection by Sars-Cov-2.
Spain, Ceuta	Government of Spain, Institute of Health Carlos III (Spain). Spain - National Study of Sero-epidemiology of Infection by Sars-Cov-2.
Spain, Community of Madrid	Government of Spain, Institute of Health Carlos III (Spain). Spain - National Study of Sero-epidemiology of Infection by Sars-Cov-2.
Spain, Extremadura	Government of Spain, Institute of Health Carlos III (Spain). Spain - National Study of Sero-epidemiology of Infection by Sars-Cov-2.
Spain, Galicia	Government of Spain, Institute of Health Carlos III (Spain). Spain - National Study of Sero-epidemiology of Infection by Sars-Cov-2.

Spain, La Rioja	Government of Spain, Institute of Health Carlos III (Spain). Spain - National Study of Sero-epidemiology of Infection by Sars-Cov-2.
Spain, Melilla	Government of Spain, Institute of Health Carlos III (Spain). Spain - National Study of Sero-epidemiology of Infection by Sars-Cov-2.
Spain, Murcia	Government of Spain, Institute of Health Carlos III (Spain). Spain - National Study of Sero-epidemiology of Infection by Sars-Cov-2.
Spain, Navarre	Government of Spain, Institute of Health Carlos III (Spain). Spain - National Study of Sero-epidemiology of Infection by Sars-Cov-2.
Spain, Valencian Community	Government of Spain, Institute of Health Carlos III (Spain). Spain - National Study of Sero-epidemiology of Infection by Sars-Cov-2.
Sweden	Public Health Agency of Sweden. Presence of antibodies to SARS-CoV-2 in Sweden, 26 April - 9 May 2021. Östersund, Sweden: Public Health Agency of Sweden.
Sweden	Public Health Agency of Sweden. Detection of Antibodies After Review of Covid-19 in Blood Donors (Sub-report 2). Östersund, Sweden: Public Health Agency of Sweden.
UK, England	Office for National Statistics (ONS) (United Kingdom). England - Coronavirus (COVID-19) Infection Survey: Antibody Data, January 2021. Newport, United Kingdom: Office for National Statistics (ONS) (United Kingdom), 2021.
UK, England	National Health Service (United Kingdom), Public Health England. United Kingdom - England Weekly National COVID-19 Surveillance Report. London, United Kingdom: Government of the United Kingdom.
UK, England	Ward H, Atchison C, Whitaker M, Ainslie KEC, Elliott J, Okell L, Redd R, Ashby D, Donnelly CA, Barclay W, Darzi A, Cooke G, Riley S, Elliott P. SARS-CoV-2 antibody prevalence in England following the first peak of the pandemic. Nat Commun. 2021; 12(905).
UK, England	Ward H, Cooke G, Atchison C, Whitaker M, Elliott J, Moshe M, Brown JC, Flower B, Daunt A, Ainslie K, Ashby D, Donnelly C, Riley S, Darzi A, Barclay W, Elliott P, for the React study team. Declining prevalence of antibody positivity to SARS-CoV-2: a community study of 365,000 adults. Preprint. medRxiv. 2020.
UK, England	Office for National Statistics (ONS) (United Kingdom). United Kingdom Coronavirus (COVID-19) Infection Survey Pilot 2020. Newport, United Kingdom: Office for National Statistics (ONS) (United Kingdom), 2020.
UK, England	Office for National Statistics (ONS) (United Kingdom). United Kingdom Coronavirus (COVID-19) Infection Survey. Newport, United Kingdom: Office for National Statistics (ONS) (United Kingdom), 2020.
UK, Northern Ireland	Office for National Statistics (ONS) (United Kingdom). England - Coronavirus (COVID-19) Infection Survey: Antibody Data, January 2021. Newport, United Kingdom: Office for National Statistics (ONS) (United Kingdom), 2021.
UK, Scotland	Office for National Statistics (ONS) (United Kingdom). England - Coronavirus (COVID-19) Infection Survey: Antibody Data, January 2021. Newport, United Kingdom: Office for National Statistics (ONS) (United Kingdom), 2021.
UK, Scotland	Public Health Scotland, Scottish National Blood Transfusion Service (SNBTS), The Centre for Virus Research (CVR) at the University of Glasgow. Scotland Enhanced Surveillance of Covid-19 Dashboard. Edinburgh, Scotland: Public Health Scotland.
UK, Scotland	Thompson CP, Grayson N, Paton R, Bolton JS, Lourenço J, Penman B, Lee LN, Odon V, Mongkolsapaya J, Chinnakannan S, Dejnirattisai W, Edmans M, Fyfe A, Imlach C, Kooblall K, Lim N, Liu C, Lopez-Camacho C, McInally CA, Ramamurthy N, Ratcliff J, Supasa P, Wang B, Mentzer AJ, Turner M, Sampson O, Semple C, Baillie JK, ISARIC4C Investigators, Harvala H, Screaton G, Temperton N, Klenerman P, Jarvis L, Gupta S, Simmonds P. Detection of neutralising antibodies to SARS coronavirus 2 to determine population exposure in Scottish blood donors between March and May 2020. Preprint. medRxiv. 2020.
UK, Wales	Office for National Statistics (ONS) (United Kingdom). England - Coronavirus (COVID-19) Infection Survey: Antibody Data, January 2021. Newport, United Kingdom: Office for National Statistics (ONS) (United Kingdom), 2021.

USA, Alabama	Jones JM, Stone M, Sulaeman H, Fink RV, Dave H, Levy ME, Di Germanio C, Green V, Notari E, Saa P, Biggerstaff BJ, Strauss D, Kessler D, Vassallo R, Reik R, Rossmann S, Destree M, Nguyen KA, Sayers M, Lough C, Bougie DW, Ritter M, Latoni G, Weales B, Sime S, Gorlin J, Brown NE, Gould CV, Berney K, Benoit TJ, Miller MJ, Freeman D, Kartik D, Fry AM, Azziz-Baumgartner E, Hall AJ, MacNeil A, Gundlapalli AV, Basavaraju SV, Gerber SI, Patton ME, Custer B, Williamson P, Simmons G, Thornburg NJ, Kleinman S, Stramer SL, Opsomer J, Busch MP. Estimated US Infection- and Vaccine-Induced SARS-CoV-2 Seroprevalence Based on Blood Donations, July 2020-May 2021. JAMA. 2021.
USA, Alabama	Centers for Disease Control and Prevention (CDC). CDC COVID Data Tracker Commercial Laboratory Seroprevalence Survey Data. Atlanta, United States of America: Centers for Disease Control and Prevention (CDC).
USA, Alabama	Anand S, Montez-Rath M, Han J, Bozeman J, Kerschmann R, Beyer P, Parsonnet J, Chertow GM. Prevalence of SARS-CoV-2 antibodies in a large nationwide sample of patients on dialysis in the USA: a cross-sectional study. Lancet. 2020.
USA, Alaska	Jones JM, Stone M, Sulaeman H, Fink RV, Dave H, Levy ME, Di Germanio C, Green V, Notari E, Saa P, Biggerstaff BJ, Strauss D, Kessler D, Vassallo R, Reik R, Rossmann S, Destree M, Nguyen KA, Sayers M, Lough C, Bougie DW, Ritter M, Latoni G, Weales B, Sime S, Gorlin J, Brown NE, Gould CV, Berney K, Benoit TJ, Miller MJ, Freeman D, Kartik D, Fry AM, Azziz-Baumgartner E, Hall AJ, MacNeil A, Gundlapalli AV, Basavaraju SV, Gerber SI, Patton ME, Custer B, Williamson P, Simmons G, Thornburg NJ, Kleinman S, Stramer SL, Opsomer J, Busch MP. Estimated US Infection- and Vaccine-Induced SARS-CoV-2 Seroprevalence Based on Blood Donations, July 2020-May 2021. JAMA. 2021.
USA, Alaska	Centers for Disease Control and Prevention (CDC). CDC COVID Data Tracker Commercial Laboratory Seroprevalence Survey Data. Atlanta, United States of America: Centers for Disease Control and Prevention (CDC).
USA, Alaska	Anand S, Montez-Rath M, Han J, Bozeman J, Kerschmann R, Beyer P, Parsonnet J, Chertow GM. Prevalence of SARS-CoV-2 antibodies in a large nationwide sample of patients on dialysis in the USA: a cross-sectional study. Lancet. 2020.
USA, Arizona	Jones JM, Stone M, Sulaeman H, Fink RV, Dave H, Levy ME, Di Germanio C, Green V, Notari E, Saa P, Biggerstaff BJ, Strauss D, Kessler D, Vassallo R, Reik R, Rossmann S, Destree M, Nguyen KA, Sayers M, Lough C, Bougie DW, Ritter M, Latoni G, Weales B, Sime S, Gorlin J, Brown NE, Gould CV, Berney K, Benoit TJ, Miller MJ, Freeman D, Kartik D, Fry AM, Azziz-Baumgartner E, Hall AJ, MacNeil A, Gundlapalli AV, Basavaraju SV, Gerber SI, Patton ME, Custer B, Williamson P, Simmons G, Thornburg NJ, Kleinman S, Stramer SL, Opsomer J, Busch MP. Estimated US Infection- and Vaccine-Induced SARS-CoV-2 Seroprevalence Based on Blood Donations, July 2020-May 2021. JAMA. 2021.
USA, Arizona	Centers for Disease Control and Prevention (CDC). CDC COVID Data Tracker Commercial Laboratory Seroprevalence Survey Data. Atlanta, United States of America: Centers for Disease Control and Prevention (CDC).
USA, Arizona	Anand S, Montez-Rath M, Han J, Bozeman J, Kerschmann R, Beyer P, Parsonnet J, Chertow GM. Prevalence of SARS-CoV-2 antibodies in a large nationwide sample of patients on dialysis in the USA: a cross-sectional study. Lancet. 2020.
USA, Arkansas	Jones JM, Stone M, Sulaeman H, Fink RV, Dave H, Levy ME, Di Germanio C, Green V, Notari E, Saa P, Biggerstaff BJ, Strauss D, Kessler D, Vassallo R, Reik R, Rossmann S, Destree M, Nguyen KA, Sayers M, Lough C, Bougie DW, Ritter M, Latoni G, Weales B, Sime S, Gorlin J, Brown NE, Gould CV, Berney K, Benoit TJ, Miller MJ, Freeman D, Kartik D, Fry AM, Azziz-Baumgartner E, Hall AJ, MacNeil A, Gundlapalli AV, Basavaraju SV, Gerber SI, Patton ME, Custer B, Williamson P, Simmons G, Thornburg NJ, Kleinman S, Stramer SL, Opsomer J, Busch MP. Estimated US Infection- and Vaccine-Induced SARS-CoV-2 Seroprevalence Based on Blood Donations, July 2020-May 2021. JAMA. 2021.
USA, Arkansas	Centers for Disease Control and Prevention (CDC). CDC COVID Data Tracker Commercial Laboratory Seroprevalence Survey Data. Atlanta, United States of America: Centers for Disease Control and Prevention (CDC).

USA, Arkansas	Anand S, Montez-Rath M, Han J, Bozeman J, Kerschmann R, Beyer P, Parsonnet J, Chertow GM. Prevalence of SARS-CoV-2 antibodies in a large nationwide sample of patients on dialysis in the USA: a cross-sectional study. Lancet. 2020.
USA, California	Jones JM, Stone M, Sulaeman H, Fink RV, Dave H, Levy ME, Di Germanio C, Green V, Notari E, Saa P, Biggerstaff BJ, Strauss D, Kessler D, Vassallo R, Reik R, Rossmann S, Destree M, Nguyen KA, Sayers M, Lough C, Bougie DW, Ritter M, Latoni G, Weales B, Sime S, Gorlin J, Brown NE, Gould CV, Berney K, Benoit TJ, Miller MJ, Freeman D, Kartik D, Fry AM, Azziz-Baumgartner E, Hall AJ, MacNeil A, Gundlapalli AV, Basavaraju SV, Gerber SI, Patton ME, Custer B, Williamson P, Simmons G, Thornburg NJ, Kleinman S, Stramer SL, Opsomer J, Busch MP. Estimated US Infection- and Vaccine-Induced SARS-CoV-2 Seroprevalence Based on Blood Donations, July 2020-May 2021. JAMA. 2021.
USA, California	Centers for Disease Control and Prevention (CDC). CDC COVID Data Tracker Commercial Laboratory Seroprevalence Survey Data. Atlanta, United States of America: Centers for Disease Control and Prevention (CDC).
USA, California	Anand S, Montez-Rath M, Han J, Bozeman J, Kerschmann R, Beyer P, Parsonnet J, Chertow GM. Prevalence of SARS-CoV-2 antibodies in a large nationwide sample of patients on dialysis in the USA: a cross-sectional study. Lancet. 2020.
USA, Colorado	Jones JM, Stone M, Sulaeman H, Fink RV, Dave H, Levy ME, Di Germanio C, Green V, Notari E, Saa P, Biggerstaff BJ, Strauss D, Kessler D, Vassallo R, Reik R, Rossmann S, Destree M, Nguyen KA, Sayers M, Lough C, Bougie DW, Ritter M, Latoni G, Weales B, Sime S, Gorlin J, Brown NE, Gould CV, Berney K, Benoit TJ, Miller MJ, Freeman D, Kartik D, Fry AM, Azziz-Baumgartner E, Hall AJ, MacNeil A, Gundlapalli AV, Basavaraju SV, Gerber SI, Patton ME, Custer B, Williamson P, Simmons G, Thornburg NJ, Kleinman S, Stramer SL, Opsomer J, Busch MP. Estimated US Infection- and Vaccine-Induced SARS-CoV-2 Seroprevalence Based on Blood Donations, July 2020-May 2021. JAMA. 2021.
USA, Colorado	Centers for Disease Control and Prevention (CDC). CDC COVID Data Tracker Commercial Laboratory Seroprevalence Survey Data. Atlanta, United States of America: Centers for Disease Control and Prevention (CDC).
USA, Connecticut	Jones JM, Stone M, Sulaeman H, Fink RV, Dave H, Levy ME, Di Germanio C, Green V, Notari E, Saa P, Biggerstaff BJ, Strauss D, Kessler D, Vassallo R, Reik R, Rossmann S, Destree M, Nguyen KA, Sayers M, Lough C, Bougie DW, Ritter M, Latoni G, Weales B, Sime S, Gorlin J, Brown NE, Gould CV, Berney K, Benoit TJ, Miller MJ, Freeman D, Kartik D, Fry AM, Azziz-Baumgartner E, Hall AJ, MacNeil A, Gundlapalli AV, Basavaraju SV, Gerber SI, Patton ME, Custer B, Williamson P, Simmons G, Thornburg NJ, Kleinman S, Stramer SL, Opsomer J, Busch MP. Estimated US Infection- and Vaccine-Induced SARS-CoV-2 Seroprevalence Based on Blood Donations, July 2020-May 2021. JAMA. 2021.
USA, Connecticut	Havers FP, Reed C, Lim T, Montgomery JM, Klena JD, Hall AJ, Fry AM, Cannon DL, Chiang CF, Gibbons A, Krapiunaya I, Morales-Betoulle M, Roguski K, Rasheed MAU, Freeman B, Lester S, Mills L, Carroll DS, Owen SM, Johnson JA, Semenova V, Blackmore C, Blog D, Chai SJ, Dunn A, Hand J, Jain S, Lindquist S, Lynfield R, Pritchard S, Sokol T, Sosa L, Turabelidze G, Watkins SM, Wiesman J, Williams RW, Yendell S, Schiffer J, Thornburg NJ. Seroprevalence of Antibodies to SARS-CoV-2 in 10 Sites in the United States, March 23-May 12, 2020. JAMA Intern Med. 2020.
USA, Connecticut	Centers for Disease Control and Prevention (CDC). CDC COVID Data Tracker Commercial Laboratory Seroprevalence Survey Data. Atlanta, United States of America: Centers for Disease Control and Prevention (CDC).
USA, Connecticut	Anand S, Montez-Rath M, Han J, Bozeman J, Kerschmann R, Beyer P, Parsonnet J, Chertow GM. Prevalence of SARS-CoV-2 antibodies in a large nationwide sample of patients on dialysis in the USA: a cross-sectional study. Lancet. 2020.
USA, Delaware	Jones JM, Stone M, Sulaeman H, Fink RV, Dave H, Levy ME, Di Germanio C, Green V, Notari E, Saa P, Biggerstaff BJ, Strauss D, Kessler D, Vassallo R, Reik R, Rossmann S, Destree M, Nguyen KA, Sayers M, Lough C, Bougie DW, Ritter M, Latoni G, Weales B, Sime S, Gorlin J, Brown NE, Gould CV, Berney K, Benoit TJ, Miller MJ, Freeman D, Kartik D, Fry AM, Azziz-Baumgartner E, Hall AJ, MacNeil A, Gundlapalli AV, Basavaraju SV, Gerber SI, Patton ME, Custer B, Williamson P, Simmons G, Thornburg NJ, Kleinman S, Stramer SL, Opsomer J,

	Busch MP. Estimated US Infection- and Vaccine-Induced SARS-CoV-2 Seroprevalence Based on Blood Donations, July 2020-May 2021. JAMA. 2021.
USA, Delaware	Centers for Disease Control and Prevention (CDC). CDC COVID Data Tracker Commercial Laboratory Seroprevalence Survey Data. Atlanta, United States of America: Centers for Disease Control and Prevention (CDC).
USA, Delaware	Anand S, Montez-Rath M, Han J, Bozeman J, Kerschmann R, Beyer P, Parsonnet J, Chertow GM. Prevalence of SARS-CoV-2 antibodies in a large nationwide sample of patients on dialysis in the USA: a cross-sectional study. Lancet. 2020.
USA, Florida	Jones JM, Stone M, Sulaeman H, Fink RV, Dave H, Levy ME, Di Germanio C, Green V, Notari E, Saa P, Biggerstaff BJ, Strauss D, Kessler D, Vassallo R, Reik R, Rossmann S, Destree M, Nguyen KA, Sayers M, Lough C, Bougie DW, Ritter M, Latoni G, Weales B, Sime S, Gorlin J, Brown NE, Gould CV, Berney K, Benoit TJ, Miller MJ, Freeman D, Kartik D, Fry AM, Azziz-Baumgartner E, Hall AJ, MacNeil A, Gundlapalli AV, Basavaraju SV, Gerber SI, Patton ME, Custer B, Williamson P, Simmons G, Thornburg NJ, Kleinman S, Stramer SL, Opsomer J, Busch MP. Estimated US Infection- and Vaccine-Induced SARS-CoV-2 Seroprevalence Based on Blood Donations, July 2020-May 2021. JAMA. 2021.
USA, Florida	Centers for Disease Control and Prevention (CDC). CDC COVID Data Tracker Commercial Laboratory Seroprevalence Survey Data. Atlanta, United States of America: Centers for Disease Control and Prevention (CDC).
USA, Florida	Anand S, Montez-Rath M, Han J, Bozeman J, Kerschmann R, Beyer P, Parsonnet J, Chertow GM. Prevalence of SARS-CoV-2 antibodies in a large nationwide sample of patients on dialysis in the USA: a cross-sectional study. Lancet. 2020.
USA, Georgia	Jones JM, Stone M, Sulaeman H, Fink RV, Dave H, Levy ME, Di Germanio C, Green V, Notari E, Saa P, Biggerstaff BJ, Strauss D, Kessler D, Vassallo R, Reik R, Rossmann S, Destree M, Nguyen KA, Sayers M, Lough C, Bougie DW, Ritter M, Latoni G, Weales B, Sime S, Gorlin J, Brown NE, Gould CV, Berney K, Benoit TJ, Miller MJ, Freeman D, Kartik D, Fry AM, Azziz-Baumgartner E, Hall AJ, MacNeil A, Gundlapalli AV, Basavaraju SV, Gerber SI, Patton ME, Custer B, Williamson P, Simmons G, Thornburg NJ, Kleinman S, Stramer SL, Opsomer J, Busch MP. Estimated US Infection- and Vaccine-Induced SARS-CoV-2 Seroprevalence Based on Blood Donations, July 2020-May 2021. JAMA. 2021.
USA, Georgia	Centers for Disease Control and Prevention (CDC). CDC COVID Data Tracker Commercial Laboratory Seroprevalence Survey Data. Atlanta, United States of America: Centers for Disease Control and Prevention (CDC).
USA, Georgia	Anand S, Montez-Rath M, Han J, Bozeman J, Kerschmann R, Beyer P, Parsonnet J, Chertow GM. Prevalence of SARS-CoV-2 antibodies in a large nationwide sample of patients on dialysis in the USA: a cross-sectional study. Lancet. 2020.
USA, Hawaii	Jones JM, Stone M, Sulaeman H, Fink RV, Dave H, Levy ME, Di Germanio C, Green V, Notari E, Saa P, Biggerstaff BJ, Strauss D, Kessler D, Vassallo R, Reik R, Rossmann S, Destree M, Nguyen KA, Sayers M, Lough C, Bougie DW, Ritter M, Latoni G, Weales B, Sime S, Gorlin J, Brown NE, Gould CV, Berney K, Benoit TJ, Miller MJ, Freeman D, Kartik D, Fry AM, Azziz-Baumgartner E, Hall AJ, MacNeil A, Gundlapalli AV, Basavaraju SV, Gerber SI, Patton ME, Custer B, Williamson P, Simmons G, Thornburg NJ, Kleinman S, Stramer SL, Opsomer J, Busch MP. Estimated US Infection- and Vaccine-Induced SARS-CoV-2 Seroprevalence Based on Blood Donations, July 2020-May 2021. JAMA. 2021.
USA, Hawaii	Centers for Disease Control and Prevention (CDC). CDC COVID Data Tracker Commercial Laboratory Seroprevalence Survey Data. Atlanta, United States of America: Centers for Disease Control and Prevention (CDC).
USA, Hawaii	Anand S, Montez-Rath M, Han J, Bozeman J, Kerschmann R, Beyer P, Parsonnet J, Chertow GM. Prevalence of SARS-CoV-2 antibodies in a large nationwide sample of patients on dialysis in the USA: a cross-sectional study. Lancet. 2020.
USA, Idaho	Jones JM, Stone M, Sulaeman H, Fink RV, Dave H, Levy ME, Di Germanio C, Green V, Notari E, Saa P, Biggerstaff BJ, Strauss D, Kessler D, Vassallo R, Reik R, Rossmann S, Destree M, Nguyen KA, Sayers M, Lough C, Bougie DW, Ritter M, Latoni G, Weales B, Sime S, Gorlin J, Brown NE, Gould CV, Berney K, Benoit TJ, Miller MJ, Freeman D, Kartik D, Fry AM, Azziz-Baumgartner E, Hall AJ, MacNeil A, Gundlapalli AV, Basavaraju SV, Gerber SI, Patton ME,

	Custer B, Williamson P, Simmons G, Thornburg NJ, Kleinman S, Stramer SL, Opsomer J, Busch MP. Estimated US Infection- and Vaccine-Induced SARS-CoV-2 Seroprevalence Based on Blood Donations, July 2020-May 2021. JAMA. 2021.
USA, Idaho	Centers for Disease Control and Prevention (CDC). CDC COVID Data Tracker Commercial Laboratory Seroprevalence Survey Data. Atlanta, United States of America: Centers for Disease Control and Prevention (CDC).
USA, Illinois	Jones JM, Stone M, Sulaeman H, Fink RV, Dave H, Levy ME, Di Germanio C, Green V, Notari E, Saa P, Biggerstaff BJ, Strauss D, Kessler D, Vassallo R, Reik R, Rossmann S, Destree M, Nguyen KA, Sayers M, Lough C, Bougie DW, Ritter M, Latoni G, Weales B, Sime S, Gorlin J, Brown NE, Gould CV, Berney K, Benoit TJ, Miller MJ, Freeman D, Kartik D, Fry AM, Azziz-Baumgartner E, Hall AJ, MacNeil A, Gundlapalli AV, Basavaraju SV, Gerber SI, Patton ME, Custer B, Williamson P, Simmons G, Thornburg NJ, Kleinman S, Stramer SL, Opsomer J, Busch MP. Estimated US Infection- and Vaccine-Induced SARS-CoV-2 Seroprevalence Based on Blood Donations, July 2020-May 2021. JAMA. 2021.
USA, Illinois	Centers for Disease Control and Prevention (CDC). CDC COVID Data Tracker Commercial Laboratory Seroprevalence Survey Data. Atlanta, United States of America: Centers for Disease Control and Prevention (CDC).
USA, Indiana	Jones JM, Stone M, Sulaeman H, Fink RV, Dave H, Levy ME, Di Germanio C, Green V, Notari E, Saa P, Biggerstaff BJ, Strauss D, Kessler D, Vassallo R, Reik R, Rossmann S, Destree M, Nguyen KA, Sayers M, Lough C, Bougie DW, Ritter M, Latoni G, Weales B, Sime S, Gorlin J, Brown NE, Gould CV, Berney K, Benoit TJ, Miller MJ, Freeman D, Kartik D, Fry AM, Azziz-Baumgartner E, Hall AJ, MacNeil A, Gundlapalli AV, Basavaraju SV, Gerber SI, Patton ME, Custer B, Williamson P, Simmons G, Thornburg NJ, Kleinman S, Stramer SL, Opsomer J, Busch MP. Estimated US Infection- and Vaccine-Induced SARS-CoV-2 Seroprevalence Based on Blood Donations, July 2020-May 2021. JAMA. 2021.
USA, Indiana	Centers for Disease Control and Prevention (CDC). CDC COVID Data Tracker Commercial Laboratory Seroprevalence Survey Data. Atlanta, United States of America: Centers for Disease Control and Prevention (CDC).
USA, Indiana	Menachemi N, Yiannoutsos CT, Dixon BE, Duszynski TJ, Fadel WF, Wools-Kaloustian KK, Unruh Needleman N, Box K, Caine V, Norwood C, Weaver L, Halverson PK. Population Point Prevalence of SARS-CoV-2 Infection Based on a Statewide Random Sample - Indiana, April 25-29, 2020. MMWR Morb Mortal Wkly Rep. 2020; 69(29): 960-964.
USA, Indiana	Anand S, Montez-Rath M, Han J, Bozeman J, Kerschmann R, Beyer P, Parsonnet J, Chertow GM. Prevalence of SARS-CoV-2 antibodies in a large nationwide sample of patients on dialysis in the USA: a cross-sectional study. Lancet. 2020.
USA, Iowa	Jones JM, Stone M, Sulaeman H, Fink RV, Dave H, Levy ME, Di Germanio C, Green V, Notari E, Saa P, Biggerstaff BJ, Strauss D, Kessler D, Vassallo R, Reik R, Rossmann S, Destree M, Nguyen KA, Sayers M, Lough C, Bougie DW, Ritter M, Latoni G, Weales B, Sime S, Gorlin J, Brown NE, Gould CV, Berney K, Benoit TJ, Miller MJ, Freeman D, Kartik D, Fry AM, Azziz-Baumgartner E, Hall AJ, MacNeil A, Gundlapalli AV, Basavaraju SV, Gerber SI, Patton ME, Custer B, Williamson P, Simmons G, Thornburg NJ, Kleinman S, Stramer SL, Opsomer J, Busch MP. Estimated US Infection- and Vaccine-Induced SARS-CoV-2 Seroprevalence Based on Blood Donations, July 2020-May 2021. JAMA. 2021.
USA, Iowa	Centers for Disease Control and Prevention (CDC). CDC COVID Data Tracker Commercial Laboratory Seroprevalence Survey Data. Atlanta, United States of America: Centers for Disease Control and Prevention (CDC).
USA, Kansas	Jones JM, Stone M, Sulaeman H, Fink RV, Dave H, Levy ME, Di Germanio C, Green V, Notari E, Saa P, Biggerstaff BJ, Strauss D, Kessler D, Vassallo R, Reik R, Rossmann S, Destree M, Nguyen KA, Sayers M, Lough C, Bougie DW, Ritter M, Latoni G, Weales B, Sime S, Gorlin J, Brown NE, Gould CV, Berney K, Benoit TJ, Miller MJ, Freeman D, Kartik D, Fry AM, Azziz-Baumgartner E, Hall AJ, MacNeil A, Gundlapalli AV, Basavaraju SV, Gerber SI, Patton ME, Custer B, Williamson P, Simmons G, Thornburg NJ, Kleinman S, Stramer SL, Opsomer J, Busch MP. Estimated US Infection- and Vaccine-Induced SARS-CoV-2 Seroprevalence Based on Blood Donations, July 2020-May 2021. JAMA. 2021.

USA, Kansas	Centers for Disease Control and Prevention (CDC). CDC COVID Data Tracker Commercial Laboratory Seroprevalence Survey Data. Atlanta, United States of America: Centers for Disease Control and Prevention (CDC).
USA, Kentucky	Jones JM, Stone M, Sulaeman H, Fink RV, Dave H, Levy ME, Di Germanio C, Green V, Notari E, Saa P, Biggerstaff BJ, Strauss D, Kessler D, Vassallo R, Reik R, Rossmann S, Destree M, Nguyen KA, Sayers M, Lough C, Bougie DW, Ritter M, Latoni G, Weales B, Sime S, Gorlin J, Brown NE, Gould CV, Berney K, Benoit TJ, Miller MJ, Freeman D, Kartik D, Fry AM, Azziz-Baumgartner E, Hall AJ, MacNeil A, Gundlapalli AV, Basavaraju SV, Gerber SI, Patton ME, Custer B, Williamson P, Simmons G, Thornburg NJ, Kleinman S, Stramer SL, Opsomer J, Busch MP. Estimated US Infection- and Vaccine-Induced SARS-CoV-2 Seroprevalence Based on Blood Donations, July 2020-May 2021. JAMA. 2021.
USA, Kentucky	Centers for Disease Control and Prevention (CDC). CDC COVID Data Tracker Commercial Laboratory Seroprevalence Survey Data. Atlanta, United States of America: Centers for Disease Control and Prevention (CDC).
USA, Kentucky	Anand S, Montez-Rath M, Han J, Bozeman J, Kerschmann R, Beyer P, Parsonnet J, Chertow GM. Prevalence of SARS-CoV-2 antibodies in a large nationwide sample of patients on dialysis in the USA: a cross-sectional study. Lancet. 2020.
USA, Louisiana	Jones JM, Stone M, Sulaeman H, Fink RV, Dave H, Levy ME, Di Germanio C, Green V, Notari E, Saa P, Biggerstaff BJ, Strauss D, Kessler D, Vassallo R, Reik R, Rossmann S, Destree M, Nguyen KA, Sayers M, Lough C, Bougie DW, Ritter M, Latoni G, Weales B, Sime S, Gorlin J, Brown NE, Gould CV, Berney K, Benoit TJ, Miller MJ, Freeman D, Kartik D, Fry AM, Azziz-Baumgartner E, Hall AJ, MacNeil A, Gundlapalli AV, Basavaraju SV, Gerber SI, Patton ME, Custer B, Williamson P, Simmons G, Thornburg NJ, Kleinman S, Stramer SL, Opsomer J, Busch MP. Estimated US Infection- and Vaccine-Induced SARS-CoV-2 Seroprevalence Based on Blood Donations, July 2020-May 2021. JAMA. 2021.
USA, Louisiana	Havers FP, Reed C, Lim T, Montgomery JM, Klena JD, Hall AJ, Fry AM, Cannon DL, Chiang CF, Gibbons A, Krapiunaya I, Morales-Betoulle M, Roguski K, Rasheed MAU, Freeman B, Lester S, Mills L, Carroll DS, Owen SM, Johnson JA, Semenova V, Blackmore C, Blog D, Chai SJ, Dunn A, Hand J, Jain S, Lindquist S, Lynfield R, Pritchard S, Sokol T, Sosa L, Turabelidze G, Watkins SM, Wiesman J, Williams RW, Yendell S, Schiffer J, Thornburg NJ. Seroprevalence of Antibodies to SARS-CoV-2 in 10 Sites in the United States, March 23-May 12, 2020. JAMA Intern Med. 2020.
USA, Louisiana	Centers for Disease Control and Prevention (CDC). CDC COVID Data Tracker Commercial Laboratory Seroprevalence Survey Data. Atlanta, United States of America: Centers for Disease Control and Prevention (CDC).
USA, Maine	Jones JM, Stone M, Sulaeman H, Fink RV, Dave H, Levy ME, Di Germanio C, Green V, Notari E, Saa P, Biggerstaff BJ, Strauss D, Kessler D, Vassallo R, Reik R, Rossmann S, Destree M, Nguyen KA, Sayers M, Lough C, Bougie DW, Ritter M, Latoni G, Weales B, Sime S, Gorlin J, Brown NE, Gould CV, Berney K, Benoit TJ, Miller MJ, Freeman D, Kartik D, Fry AM, Azziz-Baumgartner E, Hall AJ, MacNeil A, Gundlapalli AV, Basavaraju SV, Gerber SI, Patton ME, Custer B, Williamson P, Simmons G, Thornburg NJ, Kleinman S, Stramer SL, Opsomer J, Busch MP. Estimated US Infection- and Vaccine-Induced SARS-CoV-2 Seroprevalence Based on Blood Donations, July 2020-May 2021. JAMA. 2021.
USA, Maine	Centers for Disease Control and Prevention (CDC). CDC COVID Data Tracker Commercial Laboratory Seroprevalence Survey Data. Atlanta, United States of America: Centers for Disease Control and Prevention (CDC).
USA, Maryland	Jones JM, Stone M, Sulaeman H, Fink RV, Dave H, Levy ME, Di Germanio C, Green V, Notari E, Saa P, Biggerstaff BJ, Strauss D, Kessler D, Vassallo R, Reik R, Rossmann S, Destree M, Nguyen KA, Sayers M, Lough C, Bougie DW, Ritter M, Latoni G, Weales B, Sime S, Gorlin J, Brown NE, Gould CV, Berney K, Benoit TJ, Miller MJ, Freeman D, Kartik D, Fry AM, Azziz-Baumgartner E, Hall AJ, MacNeil A, Gundlapalli AV, Basavaraju SV, Gerber SI, Patton ME, Custer B, Williamson P, Simmons G, Thornburg NJ, Kleinman S, Stramer SL, Opsomer J, Busch MP. Estimated US Infection- and Vaccine-Induced SARS-CoV-2 Seroprevalence Based on Blood Donations, July 2020-May 2021. JAMA. 2021.

USA, Maryland	Centers for Disease Control and Prevention (CDC). CDC COVID Data Tracker Commercial Laboratory Seroprevalence Survey Data. Atlanta, United States of America: Centers for Disease Control and Prevention (CDC).
USA, Maryland	Anand S, Montez-Rath M, Han J, Bozeman J, Kerschmann R, Beyer P, Parsonnet J, Chertow GM. Prevalence of SARS-CoV-2 antibodies in a large nationwide sample of patients on dialysis in the USA: a cross-sectional study. Lancet. 2020.
USA, Massachusetts	Jones JM, Stone M, Sulaeman H, Fink RV, Dave H, Levy ME, Di Germanio C, Green V, Notari E, Saa P, Biggerstaff BJ, Strauss D, Kessler D, Vassallo R, Reik R, Rossmann S, Destree M, Nguyen KA, Sayers M, Lough C, Bougie DW, Ritter M, Latoni G, Weales B, Sime S, Gorlin J, Brown NE, Gould CV, Berney K, Benoit TJ, Miller MJ, Freeman D, Kartik D, Fry AM, Azziz-Baumgartner E, Hall AJ, MacNeil A, Gundlapalli AV, Basavaraju SV, Gerber SI, Patton ME, Custer B, Williamson P, Simmons G, Thornburg NJ, Kleinman S, Stramer SL, Opsomer J, Busch MP. Estimated US Infection- and Vaccine-Induced SARS-CoV-2 Seroprevalence Based on Blood Donations, July 2020-May 2021. JAMA. 2021.
USA, Massachusetts	Centers for Disease Control and Prevention (CDC). CDC COVID Data Tracker Commercial Laboratory Seroprevalence Survey Data. Atlanta, United States of America: Centers for Disease Control and Prevention (CDC).
USA, Michigan	Jones JM, Stone M, Sulaeman H, Fink RV, Dave H, Levy ME, Di Germanio C, Green V, Notari E, Saa P, Biggerstaff BJ, Strauss D, Kessler D, Vassallo R, Reik R, Rossmann S, Destree M, Nguyen KA, Sayers M, Lough C, Bougie DW, Ritter M, Latoni G, Weales B, Sime S, Gorlin J, Brown NE, Gould CV, Berney K, Benoit TJ, Miller MJ, Freeman D, Kartik D, Fry AM, Azziz-Baumgartner E, Hall AJ, MacNeil A, Gundlapalli AV, Basavaraju SV, Gerber SI, Patton ME, Custer B, Williamson P, Simmons G, Thornburg NJ, Kleinman S, Stramer SL, Opsomer J, Busch MP. Estimated US Infection- and Vaccine-Induced SARS-CoV-2 Seroprevalence Based on Blood Donations, July 2020-May 2021. JAMA. 2021.
USA, Michigan	Centers for Disease Control and Prevention (CDC). CDC COVID Data Tracker Commercial Laboratory Seroprevalence Survey Data. Atlanta, United States of America: Centers for Disease Control and Prevention (CDC).
USA, Michigan	Anand S, Montez-Rath M, Han J, Bozeman J, Kerschmann R, Beyer P, Parsonnet J, Chertow GM. Prevalence of SARS-CoV-2 antibodies in a large nationwide sample of patients on dialysis in the USA: a cross-sectional study. Lancet. 2020.
USA, Minnesota	Jones JM, Stone M, Sulaeman H, Fink RV, Dave H, Levy ME, Di Germanio C, Green V, Notari E, Saa P, Biggerstaff BJ, Strauss D, Kessler D, Vassallo R, Reik R, Rossmann S, Destree M, Nguyen KA, Sayers M, Lough C, Bougie DW, Ritter M, Latoni G, Weales B, Sime S, Gorlin J, Brown NE, Gould CV, Berney K, Benoit TJ, Miller MJ, Freeman D, Kartik D, Fry AM, Azziz-Baumgartner E, Hall AJ, MacNeil A, Gundlapalli AV, Basavaraju SV, Gerber SI, Patton ME, Custer B, Williamson P, Simmons G, Thornburg NJ, Kleinman S, Stramer SL, Opsomer J, Busch MP. Estimated US Infection- and Vaccine-Induced SARS-CoV-2 Seroprevalence Based on Blood Donations, July 2020-May 2021. JAMA. 2021.
USA, Minnesota	Centers for Disease Control and Prevention (CDC). CDC COVID Data Tracker Commercial Laboratory Seroprevalence Survey Data. Atlanta, United States of America: Centers for Disease Control and Prevention (CDC).
USA, Minnesota	Anand S, Montez-Rath M, Han J, Bozeman J, Kerschmann R, Beyer P, Parsonnet J, Chertow GM. Prevalence of SARS-CoV-2 antibodies in a large nationwide sample of patients on dialysis in the USA: a cross-sectional study. Lancet. 2020.
USA, Mississippi	Jones JM, Stone M, Sulaeman H, Fink RV, Dave H, Levy ME, Di Germanio C, Green V, Notari E, Saa P, Biggerstaff BJ, Strauss D, Kessler D, Vassallo R, Reik R, Rossmann S, Destree M, Nguyen KA, Sayers M, Lough C, Bougie DW, Ritter M, Latoni G, Weales B, Sime S, Gorlin J, Brown NE, Gould CV, Berney K, Benoit TJ, Miller MJ, Freeman D, Kartik D, Fry AM, Azziz-Baumgartner E, Hall AJ, MacNeil A, Gundlapalli AV, Basavaraju SV, Gerber SI, Patton ME, Custer B, Williamson P, Simmons G, Thornburg NJ, Kleinman S, Stramer SL, Opsomer J, Busch MP. Estimated US Infection- and Vaccine-Induced SARS-CoV-2 Seroprevalence Based on Blood Donations, July 2020-May 2021. JAMA. 2021.

USA, Mississippi	Centers for Disease Control and Prevention (CDC). CDC COVID Data Tracker Commercial Laboratory Seroprevalence Survey Data. Atlanta, United States of America: Centers for Disease Control and Prevention (CDC).
USA, Mississippi	Anand S, Montez-Rath M, Han J, Bozeman J, Kerschmann R, Beyer P, Parsonnet J, Chertow GM. Prevalence of SARS-CoV-2 antibodies in a large nationwide sample of patients on dialysis in the USA: a cross-sectional study. Lancet. 2020.
USA, Missouri	Jones JM, Stone M, Sulaeman H, Fink RV, Dave H, Levy ME, Di Germanio C, Green V, Notari E, Saa P, Biggerstaff BJ, Strauss D, Kessler D, Vassallo R, Reik R, Rossmann S, Destree M, Nguyen KA, Sayers M, Lough C, Bougie DW, Ritter M, Latoni G, Weales B, Sime S, Gorlin J, Brown NE, Gould CV, Berney K, Benoit TJ, Miller MJ, Freeman D, Kartik D, Fry AM, Azziz-Baumgartner E, Hall AJ, MacNeil A, Gundlapalli AV, Basavaraju SV, Gerber SI, Patton ME, Custer B, Williamson P, Simmons G, Thornburg NJ, Kleinman S, Stramer SL, Opsomer J, Busch MP. Estimated US Infection- and Vaccine-Induced SARS-CoV-2 Seroprevalence Based on Blood Donations, July 2020-May 2021. JAMA. 2021.
USA, Missouri	Havers FP, Reed C, Lim T, Montgomery JM, Klena JD, Hall AJ, Fry AM, Cannon DL, Chiang CF, Gibbons A, Krapiunaya I, Morales-Betoulle M, Roguski K, Rasheed MAU, Freeman B, Lester S, Mills L, Carroll DS, Owen SM, Johnson JA, Semenova V, Blackmore C, Blog D, Chai SJ, Dunn A, Hand J, Jain S, Lindquist S, Lynfield R, Pritchard S, Sokol T, Sosa L, Turabelidze G, Watkins SM, Wiesman J, Williams RW, Yendell S, Schiffer J, Thornburg NJ. Seroprevalence of Antibodies to SARS-CoV-2 in 10 Sites in the United States, March 23-May 12, 2020. JAMA Intern Med. 2020.
USA, Missouri	Centers for Disease Control and Prevention (CDC). CDC COVID Data Tracker Commercial Laboratory Seroprevalence Survey Data. Atlanta, United States of America: Centers for Disease Control and Prevention (CDC).
USA, Missouri	Anand S, Montez-Rath M, Han J, Bozeman J, Kerschmann R, Beyer P, Parsonnet J, Chertow GM. Prevalence of SARS-CoV-2 antibodies in a large nationwide sample of patients on dialysis in the USA: a cross-sectional study. Lancet. 2020.
USA, Montana	Jones JM, Stone M, Sulaeman H, Fink RV, Dave H, Levy ME, Di Germanio C, Green V, Notari E, Saa P, Biggerstaff BJ, Strauss D, Kessler D, Vassallo R, Reik R, Rossmann S, Destree M, Nguyen KA, Sayers M, Lough C, Bougie DW, Ritter M, Latoni G, Weales B, Sime S, Gorlin J, Brown NE, Gould CV, Berney K, Benoit TJ, Miller MJ, Freeman D, Kartik D, Fry AM, Azziz-Baumgartner E, Hall AJ, MacNeil A, Gundlapalli AV, Basavaraju SV, Gerber SI, Patton ME, Custer B, Williamson P, Simmons G, Thornburg NJ, Kleinman S, Stramer SL, Opsomer J, Busch MP. Estimated US Infection- and Vaccine-Induced SARS-CoV-2 Seroprevalence Based on Blood Donations, July 2020-May 2021. JAMA. 2021.
USA, Montana	Centers for Disease Control and Prevention (CDC). CDC COVID Data Tracker Commercial Laboratory Seroprevalence Survey Data. Atlanta, United States of America: Centers for Disease Control and Prevention (CDC).
USA, Nebraska	Jones JM, Stone M, Sulaeman H, Fink RV, Dave H, Levy ME, Di Germanio C, Green V, Notari E, Saa P, Biggerstaff BJ, Strauss D, Kessler D, Vassallo R, Reik R, Rossmann S, Destree M, Nguyen KA, Sayers M, Lough C, Bougie DW, Ritter M, Latoni G, Weales B, Sime S, Gorlin J, Brown NE, Gould CV, Berney K, Benoit TJ, Miller MJ, Freeman D, Kartik D, Fry AM, Azziz-Baumgartner E, Hall AJ, MacNeil A, Gundlapalli AV, Basavaraju SV, Gerber SI, Patton ME, Custer B, Williamson P, Simmons G, Thornburg NJ, Kleinman S, Stramer SL, Opsomer J, Busch MP. Estimated US Infection- and Vaccine-Induced SARS-CoV-2 Seroprevalence Based on Blood Donations, July 2020-May 2021. JAMA. 2021.
USA, Nebraska	Centers for Disease Control and Prevention (CDC). CDC COVID Data Tracker Commercial Laboratory Seroprevalence Survey Data. Atlanta, United States of America: Centers for Disease Control and Prevention (CDC).
USA, Nebraska	Anand S, Montez-Rath M, Han J, Bozeman J, Kerschmann R, Beyer P, Parsonnet J, Chertow GM. Prevalence of SARS-CoV-2 antibodies in a large nationwide sample of patients on dialysis in the USA: a cross-sectional study. Lancet. 2020.
USA, Nevada	Jones JM, Stone M, Sulaeman H, Fink RV, Dave H, Levy ME, Di Germanio C, Green V, Notari E, Saa P, Biggerstaff BJ, Strauss D, Kessler D, Vassallo R, Reik R, Rossmann S, Destree M, Nguyen KA, Sayers M, Lough C, Bougie DW, Ritter M, Latoni G, Weales B, Sime S, Gorlin J,

	Brown NE, Gould CV, Berney K, Benoit TJ, Miller MJ, Freeman D, Kartik D, Fry AM, Azziz-Baumgartner E, Hall AJ, MacNeil A, Gundlapalli AV, Basavaraju SV, Gerber SI, Patton ME, Custer B, Williamson P, Simmons G, Thornburg NJ, Kleinman S, Stramer SL, Opsomer J, Busch MP. Estimated US Infection- and Vaccine-Induced SARS-CoV-2 Seroprevalence Based on Blood Donations, July 2020-May 2021. JAMA. 2021.
USA, Nevada	Centers for Disease Control and Prevention (CDC). CDC COVID Data Tracker Commercial Laboratory Seroprevalence Survey Data. Atlanta, United States of America: Centers for Disease Control and Prevention (CDC).
USA, New Hampshire	Jones JM, Stone M, Sulaeman H, Fink RV, Dave H, Levy ME, Di Germanio C, Green V, Notari E, Saa P, Biggerstaff BJ, Strauss D, Kessler D, Vassallo R, Reik R, Rossmann S, Destree M, Nguyen KA, Sayers M, Lough C, Bougie DW, Ritter M, Latoni G, Weales B, Sime S, Gorlin J, Brown NE, Gould CV, Berney K, Benoit TJ, Miller MJ, Freeman D, Kartik D, Fry AM, Azziz-Baumgartner E, Hall AJ, MacNeil A, Gundlapalli AV, Basavaraju SV, Gerber SI, Patton ME, Custer B, Williamson P, Simmons G, Thornburg NJ, Kleinman S, Stramer SL, Opsomer J, Busch MP. Estimated US Infection- and Vaccine-Induced SARS-CoV-2 Seroprevalence Based on Blood Donations, July 2020-May 2021. JAMA. 2021.
USA, New Hampshire	Centers for Disease Control and Prevention (CDC). CDC COVID Data Tracker Commercial Laboratory Seroprevalence Survey Data. Atlanta, United States of America: Centers for Disease Control and Prevention (CDC).
USA, New Jersey	Jones JM, Stone M, Sulaeman H, Fink RV, Dave H, Levy ME, Di Germanio C, Green V, Notari E, Saa P, Biggerstaff BJ, Strauss D, Kessler D, Vassallo R, Reik R, Rossmann S, Destree M, Nguyen KA, Sayers M, Lough C, Bougie DW, Ritter M, Latoni G, Weales B, Sime S, Gorlin J, Brown NE, Gould CV, Berney K, Benoit TJ, Miller MJ, Freeman D, Kartik D, Fry AM, Azziz-Baumgartner E, Hall AJ, MacNeil A, Gundlapalli AV, Basavaraju SV, Gerber SI, Patton ME, Custer B, Williamson P, Simmons G, Thornburg NJ, Kleinman S, Stramer SL, Opsomer J, Busch MP. Estimated US Infection- and Vaccine-Induced SARS-CoV-2 Seroprevalence Based on Blood Donations, July 2020-May 2021. JAMA. 2021.
USA, New Jersey	Centers for Disease Control and Prevention (CDC). CDC COVID Data Tracker Commercial Laboratory Seroprevalence Survey Data. Atlanta, United States of America: Centers for Disease Control and Prevention (CDC).
USA, New Jersey	Anand S, Montez-Rath M, Han J, Bozeman J, Kerschmann R, Beyer P, Parsonnet J, Chertow GM. Prevalence of SARS-CoV-2 antibodies in a large nationwide sample of patients on dialysis in the USA: a cross-sectional study. Lancet. 2020.
USA, New Mexico	Jones JM, Stone M, Sulaeman H, Fink RV, Dave H, Levy ME, Di Germanio C, Green V, Notari E, Saa P, Biggerstaff BJ, Strauss D, Kessler D, Vassallo R, Reik R, Rossmann S, Destree M, Nguyen KA, Sayers M, Lough C, Bougie DW, Ritter M, Latoni G, Weales B, Sime S, Gorlin J, Brown NE, Gould CV, Berney K, Benoit TJ, Miller MJ, Freeman D, Kartik D, Fry AM, Azziz-Baumgartner E, Hall AJ, MacNeil A, Gundlapalli AV, Basavaraju SV, Gerber SI, Patton ME, Custer B, Williamson P, Simmons G, Thornburg NJ, Kleinman S, Stramer SL, Opsomer J, Busch MP. Estimated US Infection- and Vaccine-Induced SARS-CoV-2 Seroprevalence Based on Blood Donations, July 2020-May 2021. JAMA. 2021.
USA, New Mexico	Centers for Disease Control and Prevention (CDC). CDC COVID Data Tracker Commercial Laboratory Seroprevalence Survey Data. Atlanta, United States of America: Centers for Disease Control and Prevention (CDC).
USA, New Mexico	Anand S, Montez-Rath M, Han J, Bozeman J, Kerschmann R, Beyer P, Parsonnet J, Chertow GM. Prevalence of SARS-CoV-2 antibodies in a large nationwide sample of patients on dialysis in the USA: a cross-sectional study. Lancet. 2020.
USA, New York	Jones JM, Stone M, Sulaeman H, Fink RV, Dave H, Levy ME, Di Germanio C, Green V, Notari E, Saa P, Biggerstaff BJ, Strauss D, Kessler D, Vassallo R, Reik R, Rossmann S, Destree M, Nguyen KA, Sayers M, Lough C, Bougie DW, Ritter M, Latoni G, Weales B, Sime S, Gorlin J, Brown NE, Gould CV, Berney K, Benoit TJ, Miller MJ, Freeman D, Kartik D, Fry AM, Azziz-Baumgartner E, Hall AJ, MacNeil A, Gundlapalli AV, Basavaraju SV, Gerber SI, Patton ME, Custer B, Williamson P, Simmons G, Thornburg NJ, Kleinman S, Stramer SL, Opsomer J, Busch MP. Estimated US Infection- and Vaccine-Induced SARS-CoV-2 Seroprevalence Based on Blood Donations, July 2020-May 2021. JAMA. 2021.

USA, New York	Centers for Disease Control and Prevention (CDC). CDC COVID Data Tracker Commercial Laboratory Seroprevalence Survey Data. Atlanta, United States of America: Centers for Disease Control and Prevention (CDC).
USA, New York	Rosenberg ES, Tesoriero JM, Rosenthal EM, Chung R, Barranco MA, Styer LM, Parker MM, John Leung SY, Morne JE, Greene D, Holtgrave DR, Hoefer D, Kumar J, Udo T, Hutton B, Zucker HA. Cumulative incidence and diagnosis of SARS-CoV-2 infection in New York. Ann Epidemiol. 2020; 48: 23-29e4.
USA, New York	Anand S, Montez-Rath M, Han J, Bozeman J, Kerschmann R, Beyer P, Parsonnet J, Chertow GM. Prevalence of SARS-CoV-2 antibodies in a large nationwide sample of patients on dialysis in the USA: a cross-sectional study. Lancet. 2020.
USA, North Carolina	Jones JM, Stone M, Sulaeman H, Fink RV, Dave H, Levy ME, Di Germanio C, Green V, Notari E, Saa P, Biggerstaff BJ, Strauss D, Kessler D, Vassallo R, Reik R, Rossmann S, Destree M, Nguyen KA, Sayers M, Lough C, Bougie DW, Ritter M, Latoni G, Weales B, Sime S, Gorlin J, Brown NE, Gould CV, Berney K, Benoit TJ, Miller MJ, Freeman D, Kartik D, Fry AM, Azziz-Baumgartner E, Hall AJ, MacNeil A, Gundlapalli AV, Basavaraju SV, Gerber SI, Patton ME, Custer B, Williamson P, Simmons G, Thornburg NJ, Kleinman S, Stramer SL, Opsomer J, Busch MP. Estimated US Infection- and Vaccine-Induced SARS-CoV-2 Seroprevalence Based on Blood Donations, July 2020-May 2021. JAMA. 2021.
USA, North Carolina	Centers for Disease Control and Prevention (CDC). CDC COVID Data Tracker Commercial Laboratory Seroprevalence Survey Data. Atlanta, United States of America: Centers for Disease Control and Prevention (CDC).
USA, North Carolina	Anand S, Montez-Rath M, Han J, Bozeman J, Kerschmann R, Beyer P, Parsonnet J, Chertow GM. Prevalence of SARS-CoV-2 antibodies in a large nationwide sample of patients on dialysis in the USA: a cross-sectional study. Lancet. 2020.
USA, North Dakota	Jones JM, Stone M, Sulaeman H, Fink RV, Dave H, Levy ME, Di Germanio C, Green V, Notari E, Saa P, Biggerstaff BJ, Strauss D, Kessler D, Vassallo R, Reik R, Rossmann S, Destree M, Nguyen KA, Sayers M, Lough C, Bougie DW, Ritter M, Latoni G, Weales B, Sime S, Gorlin J, Brown NE, Gould CV, Berney K, Benoit TJ, Miller MJ, Freeman D, Kartik D, Fry AM, Azziz-Baumgartner E, Hall AJ, MacNeil A, Gundlapalli AV, Basavaraju SV, Gerber SI, Patton ME, Custer B, Williamson P, Simmons G, Thornburg NJ, Kleinman S, Stramer SL, Opsomer J, Busch MP. Estimated US Infection- and Vaccine-Induced SARS-CoV-2 Seroprevalence Based on Blood Donations, July 2020-May 2021. JAMA. 2021.
USA, North Dakota	Centers for Disease Control and Prevention (CDC). CDC COVID Data Tracker Commercial Laboratory Seroprevalence Survey Data. Atlanta, United States of America: Centers for Disease Control and Prevention (CDC).
USA, Ohio	Jones JM, Stone M, Sulaeman H, Fink RV, Dave H, Levy ME, Di Germanio C, Green V, Notari E, Saa P, Biggerstaff BJ, Strauss D, Kessler D, Vassallo R, Reik R, Rossmann S, Destree M, Nguyen KA, Sayers M, Lough C, Bougie DW, Ritter M, Latoni G, Weales B, Sime S, Gorlin J, Brown NE, Gould CV, Berney K, Benoit TJ, Miller MJ, Freeman D, Kartik D, Fry AM, Azziz-Baumgartner E, Hall AJ, MacNeil A, Gundlapalli AV, Basavaraju SV, Gerber SI, Patton ME, Custer B, Williamson P, Simmons G, Thornburg NJ, Kleinman S, Stramer SL, Opsomer J, Busch MP. Estimated US Infection- and Vaccine-Induced SARS-CoV-2 Seroprevalence Based on Blood Donations, July 2020-May 2021. JAMA. 2021.
USA, Ohio	Centers for Disease Control and Prevention (CDC). CDC COVID Data Tracker Commercial Laboratory Seroprevalence Survey Data. Atlanta, United States of America: Centers for Disease Control and Prevention (CDC).
USA, Ohio	Ohio Department of Health, Ohio State University. United States - Ohio Prevalence of Current and Past COVID-19 in Adults July 2020.
USA, Ohio	Anand S, Montez-Rath M, Han J, Bozeman J, Kerschmann R, Beyer P, Parsonnet J, Chertow GM. Prevalence of SARS-CoV-2 antibodies in a large nationwide sample of patients on dialysis in the USA: a cross-sectional study. Lancet. 2020.
USA, Oklahoma	Jones JM, Stone M, Sulaeman H, Fink RV, Dave H, Levy ME, Di Germanio C, Green V, Notari E, Saa P, Biggerstaff BJ, Strauss D, Kessler D, Vassallo R, Reik R, Rossmann S, Destree M, Nguyen KA, Sayers M, Lough C, Bougie DW, Ritter M, Latoni G, Weales B, Sime S, Gorlin J, Brown NE, Gould CV, Berney K, Benoit TJ, Miller MJ, Freeman D, Kartik D, Fry AM, Azziz-

	Baumgartner E, Hall AJ, MacNeil A, Gundlapalli AV, Basavaraju SV, Gerber SI, Patton ME, Custer B, Williamson P, Simmons G, Thornburg NJ, Kleinman S, Stramer SL, Opsomer J, Busch MP. Estimated US Infection- and Vaccine-Induced SARS-CoV-2 Seroprevalence Based on Blood Donations, July 2020-May 2021. JAMA. 2021.
USA, Oklahoma	Centers for Disease Control and Prevention (CDC). CDC COVID Data Tracker Commercial Laboratory Seroprevalence Survey Data. Atlanta, United States of America: Centers for Disease Control and Prevention (CDC).
USA, Oklahoma	Anand S, Montez-Rath M, Han J, Bozeman J, Kerschmann R, Beyer P, Parsonnet J, Chertow GM. Prevalence of SARS-CoV-2 antibodies in a large nationwide sample of patients on dialysis in the USA: a cross-sectional study. Lancet. 2020.
USA, Oregon	Jones JM, Stone M, Sulaeman H, Fink RV, Dave H, Levy ME, Di Germanio C, Green V, Notari E, Saa P, Biggerstaff BJ, Strauss D, Kessler D, Vassallo R, Reik R, Rossmann S, Destree M, Nguyen KA, Sayers M, Lough C, Bougie DW, Ritter M, Latoni G, Weales B, Sime S, Gorlin J, Brown NE, Gould CV, Berney K, Benoit TJ, Miller MJ, Freeman D, Kartik D, Fry AM, Azziz-Baumgartner E, Hall AJ, MacNeil A, Gundlapalli AV, Basavaraju SV, Gerber SI, Patton ME, Custer B, Williamson P, Simmons G, Thornburg NJ, Kleinman S, Stramer SL, Opsomer J, Busch MP. Estimated US Infection- and Vaccine-Induced SARS-CoV-2 Seroprevalence Based on Blood Donations, July 2020-May 2021. JAMA. 2021.
USA, Oregon	Centers for Disease Control and Prevention (CDC). CDC COVID Data Tracker Commercial Laboratory Seroprevalence Survey Data. Atlanta, United States of America: Centers for Disease Control and Prevention (CDC).
USA, Oregon	Anand S, Montez-Rath M, Han J, Bozeman J, Kerschmann R, Beyer P, Parsonnet J, Chertow GM. Prevalence of SARS-CoV-2 antibodies in a large nationwide sample of patients on dialysis in the USA: a cross-sectional study. Lancet. 2020.
USA, Pennsylvania	Jones JM, Stone M, Sulaeman H, Fink RV, Dave H, Levy ME, Di Germanio C, Green V, Notari E, Saa P, Biggerstaff BJ, Strauss D, Kessler D, Vassallo R, Reik R, Rossmann S, Destree M, Nguyen KA, Sayers M, Lough C, Bougie DW, Ritter M, Latoni G, Weales B, Sime S, Gorlin J, Brown NE, Gould CV, Berney K, Benoit TJ, Miller MJ, Freeman D, Kartik D, Fry AM, Azziz-Baumgartner E, Hall AJ, MacNeil A, Gundlapalli AV, Basavaraju SV, Gerber SI, Patton ME, Custer B, Williamson P, Simmons G, Thornburg NJ, Kleinman S, Stramer SL, Opsomer J, Busch MP. Estimated US Infection- and Vaccine-Induced SARS-CoV-2 Seroprevalence Based on Blood Donations, July 2020-May 2021. JAMA. 2021.
USA, Pennsylvania	Centers for Disease Control and Prevention (CDC). CDC COVID Data Tracker Commercial Laboratory Seroprevalence Survey Data. Atlanta, United States of America: Centers for Disease Control and Prevention (CDC).
USA, Pennsylvania	Anand S, Montez-Rath M, Han J, Bozeman J, Kerschmann R, Beyer P, Parsonnet J, Chertow GM. Prevalence of SARS-CoV-2 antibodies in a large nationwide sample of patients on dialysis in the USA: a cross-sectional study. Lancet. 2020.
USA, Rhode Island	Jones JM, Stone M, Sulaeman H, Fink RV, Dave H, Levy ME, Di Germanio C, Green V, Notari E, Saa P, Biggerstaff BJ, Strauss D, Kessler D, Vassallo R, Reik R, Rossmann S, Destree M, Nguyen KA, Sayers M, Lough C, Bougie DW, Ritter M, Latoni G, Weales B, Sime S, Gorlin J, Brown NE, Gould CV, Berney K, Benoit TJ, Miller MJ, Freeman D, Kartik D, Fry AM, Azziz-Baumgartner E, Hall AJ, MacNeil A, Gundlapalli AV, Basavaraju SV, Gerber SI, Patton ME, Custer B, Williamson P, Simmons G, Thornburg NJ, Kleinman S, Stramer SL, Opsomer J, Busch MP. Estimated US Infection- and Vaccine-Induced SARS-CoV-2 Seroprevalence Based on Blood Donations, July 2020-May 2021. JAMA. 2021.
USA, Rhode Island	Centers for Disease Control and Prevention (CDC). CDC COVID Data Tracker Commercial Laboratory Seroprevalence Survey Data. Atlanta, United States of America: Centers for Disease Control and Prevention (CDC).
USA, South Carolina	Jones JM, Stone M, Sulaeman H, Fink RV, Dave H, Levy ME, Di Germanio C, Green V, Notari E, Saa P, Biggerstaff BJ, Strauss D, Kessler D, Vassallo R, Reik R, Rossmann S, Destree M, Nguyen KA, Sayers M, Lough C, Bougie DW, Ritter M, Latoni G, Weales B, Sime S, Gorlin J, Brown NE, Gould CV, Berney K, Benoit TJ, Miller MJ, Freeman D, Kartik D, Fry AM, Azziz-Baumgartner E, Hall AJ, MacNeil A, Gundlapalli AV, Basavaraju SV, Gerber SI, Patton ME, Custer B, Williamson P, Simmons G, Thornburg NJ, Kleinman S, Stramer SL, Opsomer J,

	Busch MP. Estimated US Infection- and Vaccine-Induced SARS-CoV-2 Seroprevalence Based on Blood Donations, July 2020-May 2021. JAMA. 2021.
USA, South Carolina	Centers for Disease Control and Prevention (CDC). CDC COVID Data Tracker Commercial Laboratory Seroprevalence Survey Data. Atlanta, United States of America: Centers for Disease Control and Prevention (CDC).
USA, South Carolina	Anand S, Montez-Rath M, Han J, Bozeman J, Kerschmann R, Beyer P, Parsonnet J, Chertow GM. Prevalence of SARS-CoV-2 antibodies in a large nationwide sample of patients on dialysis in the USA: a cross-sectional study. Lancet. 2020.
USA, South Dakota	Jones JM, Stone M, Sulaeman H, Fink RV, Dave H, Levy ME, Di Germanio C, Green V, Notari E, Saa P, Biggerstaff BJ, Strauss D, Kessler D, Vassallo R, Reik R, Rossmann S, Destree M, Nguyen KA, Sayers M, Lough C, Bougie DW, Ritter M, Latoni G, Weales B, Sime S, Gorlin J, Brown NE, Gould CV, Berney K, Benoit TJ, Miller MJ, Freeman D, Kartik D, Fry AM, Azziz-Baumgartner E, Hall AJ, MacNeil A, Gundlapalli AV, Basavaraju SV, Gerber SI, Patton ME, Custer B, Williamson P, Simmons G, Thornburg NJ, Kleinman S, Stramer SL, Opsomer J, Busch MP. Estimated US Infection- and Vaccine-Induced SARS-CoV-2 Seroprevalence Based on Blood Donations, July 2020-May 2021. JAMA. 2021.
USA, South Dakota	Centers for Disease Control and Prevention (CDC). CDC COVID Data Tracker Commercial Laboratory Seroprevalence Survey Data. Atlanta, United States of America: Centers for Disease Control and Prevention (CDC).
USA, Tennessee	Jones JM, Stone M, Sulaeman H, Fink RV, Dave H, Levy ME, Di Germanio C, Green V, Notari E, Saa P, Biggerstaff BJ, Strauss D, Kessler D, Vassallo R, Reik R, Rossmann S, Destree M, Nguyen KA, Sayers M, Lough C, Bougie DW, Ritter M, Latoni G, Weales B, Sime S, Gorlin J, Brown NE, Gould CV, Berney K, Benoit TJ, Miller MJ, Freeman D, Kartik D, Fry AM, Azziz-Baumgartner E, Hall AJ, MacNeil A, Gundlapalli AV, Basavaraju SV, Gerber SI, Patton ME, Custer B, Williamson P, Simmons G, Thornburg NJ, Kleinman S, Stramer SL, Opsomer J, Busch MP. Estimated US Infection- and Vaccine-Induced SARS-CoV-2 Seroprevalence Based on Blood Donations, July 2020-May 2021. JAMA. 2021.
USA, Tennessee	Centers for Disease Control and Prevention (CDC). CDC COVID Data Tracker Commercial Laboratory Seroprevalence Survey Data. Atlanta, United States of America: Centers for Disease Control and Prevention (CDC).
USA, Tennessee	Anand S, Montez-Rath M, Han J, Bozeman J, Kerschmann R, Beyer P, Parsonnet J, Chertow GM. Prevalence of SARS-CoV-2 antibodies in a large nationwide sample of patients on dialysis in the USA: a cross-sectional study. Lancet. 2020.
USA, Texas	Jones JM, Stone M, Sulaeman H, Fink RV, Dave H, Levy ME, Di Germanio C, Green V, Notari E, Saa P, Biggerstaff BJ, Strauss D, Kessler D, Vassallo R, Reik R, Rossmann S, Destree M, Nguyen KA, Sayers M, Lough C, Bougie DW, Ritter M, Latoni G, Weales B, Sime S, Gorlin J, Brown NE, Gould CV, Berney K, Benoit TJ, Miller MJ, Freeman D, Kartik D, Fry AM, Azziz-Baumgartner E, Hall AJ, MacNeil A, Gundlapalli AV, Basavaraju SV, Gerber SI, Patton ME, Custer B, Williamson P, Simmons G, Thornburg NJ, Kleinman S, Stramer SL, Opsomer J, Busch MP. Estimated US Infection- and Vaccine-Induced SARS-CoV-2 Seroprevalence Based on Blood Donations, July 2020-May 2021. JAMA. 2021.
USA, Texas	Centers for Disease Control and Prevention (CDC). CDC COVID Data Tracker Commercial Laboratory Seroprevalence Survey Data. Atlanta, United States of America: Centers for Disease Control and Prevention (CDC).
USA, Texas	Anand S, Montez-Rath M, Han J, Bozeman J, Kerschmann R, Beyer P, Parsonnet J, Chertow GM. Prevalence of SARS-CoV-2 antibodies in a large nationwide sample of patients on dialysis in the USA: a cross-sectional study. Lancet. 2020.
USA, Utah	Jones JM, Stone M, Sulaeman H, Fink RV, Dave H, Levy ME, Di Germanio C, Green V, Notari E, Saa P, Biggerstaff BJ, Strauss D, Kessler D, Vassallo R, Reik R, Rossmann S, Destree M, Nguyen KA, Sayers M, Lough C, Bougie DW, Ritter M, Latoni G, Weales B, Sime S, Gorlin J, Brown NE, Gould CV, Berney K, Benoit TJ, Miller MJ, Freeman D, Kartik D, Fry AM, Azziz-Baumgartner E, Hall AJ, MacNeil A, Gundlapalli AV, Basavaraju SV, Gerber SI, Patton ME, Custer B, Williamson P, Simmons G, Thornburg NJ, Kleinman S, Stramer SL, Opsomer J, Busch MP. Estimated US Infection- and Vaccine-Induced SARS-CoV-2 Seroprevalence Based on Blood Donations, July 2020-May 2021. JAMA. 2021.

USA, Utah	Havers FP, Reed C, Lim T, Montgomery JM, Klena JD, Hall AJ, Fry AM, Cannon DL, Chiang CF, Gibbons A, Krapiunaya I, Morales-Betoulle M, Roguski K, Rasheed MAU, Freeman B, Lester S, Mills L, Carroll DS, Owen SM, Johnson JA, Semenova V, Blackmore C, Blog D, Chai SJ, Dunn A, Hand J, Jain S, Lindquist S, Lynfield R, Pritchard S, Sokol T, Sosa L, Turabelidze G, Watkins SM, Wiesman J, Williams RW, Yendell S, Schiffer J, Thornburg NJ. Seroprevalence of Antibodies to SARS-CoV-2 in 10 Sites in the United States, March 23-May 12, 2020. JAMA Intern Med. 2020.
USA, Utah	Centers for Disease Control and Prevention (CDC). CDC COVID Data Tracker Commercial Laboratory Seroprevalence Survey Data. Atlanta, United States of America: Centers for Disease Control and Prevention (CDC).
USA, Utah	Anand S, Montez-Rath M, Han J, Bozeman J, Kerschmann R, Beyer P, Parsonnet J, Chertow GM. Prevalence of SARS-CoV-2 antibodies in a large nationwide sample of patients on dialysis in the USA: a cross-sectional study. Lancet. 2020.
USA, Vermont	Jones JM, Stone M, Sulaeman H, Fink RV, Dave H, Levy ME, Di Germanio C, Green V, Notari E, Saa P, Biggerstaff BJ, Strauss D, Kessler D, Vassallo R, Reik R, Rossmann S, Destree M, Nguyen KA, Sayers M, Lough C, Bougie DW, Ritter M, Latoni G, Weales B, Sime S, Gorlin J, Brown NE, Gould CV, Berney K, Benoit TJ, Miller MJ, Freeman D, Kartik D, Fry AM, Azziz-Baumgartner E, Hall AJ, MacNeil A, Gundlapalli AV, Basavaraju SV, Gerber SI, Patton ME, Custer B, Williamson P, Simmons G, Thornburg NJ, Kleinman S, Stramer SL, Opsomer J, Busch MP. Estimated US Infection- and Vaccine-Induced SARS-CoV-2 Seroprevalence Based on Blood Donations, July 2020-May 2021. JAMA. 2021.
USA, Vermont	Centers for Disease Control and Prevention (CDC). CDC COVID Data Tracker Commercial Laboratory Seroprevalence Survey Data. Atlanta, United States of America: Centers for Disease Control and Prevention (CDC).
USA, Virginia	Jones JM, Stone M, Sulaeman H, Fink RV, Dave H, Levy ME, Di Germanio C, Green V, Notari E, Saa P, Biggerstaff BJ, Strauss D, Kessler D, Vassallo R, Reik R, Rossmann S, Destree M, Nguyen KA, Sayers M, Lough C, Bougie DW, Ritter M, Latoni G, Weales B, Sime S, Gorlin J, Brown NE, Gould CV, Berney K, Benoit TJ, Miller MJ, Freeman D, Kartik D, Fry AM, Azziz-Baumgartner E, Hall AJ, MacNeil A, Gundlapalli AV, Basavaraju SV, Gerber SI, Patton ME, Custer B, Williamson P, Simmons G, Thornburg NJ, Kleinman S, Stramer SL, Opsomer J, Busch MP. Estimated US Infection- and Vaccine-Induced SARS-CoV-2 Seroprevalence Based on Blood Donations, July 2020-May 2021. JAMA. 2021.
USA, Virginia	Centers for Disease Control and Prevention (CDC). CDC COVID Data Tracker Commercial Laboratory Seroprevalence Survey Data. Atlanta, United States of America: Centers for Disease Control and Prevention (CDC).
USA, Washington	Jones JM, Stone M, Sulaeman H, Fink RV, Dave H, Levy ME, Di Germanio C, Green V, Notari E, Saa P, Biggerstaff BJ, Strauss D, Kessler D, Vassallo R, Reik R, Rossmann S, Destree M, Nguyen KA, Sayers M, Lough C, Bougie DW, Ritter M, Latoni G, Weales B, Sime S, Gorlin J, Brown NE, Gould CV, Berney K, Benoit TJ, Miller MJ, Freeman D, Kartik D, Fry AM, Azziz-Baumgartner E, Hall AJ, MacNeil A, Gundlapalli AV, Basavaraju SV, Gerber SI, Patton ME, Custer B, Williamson P, Simmons G, Thornburg NJ, Kleinman S, Stramer SL, Opsomer J, Busch MP. Estimated US Infection- and Vaccine-Induced SARS-CoV-2 Seroprevalence Based on Blood Donations, July 2020-May 2021. JAMA. 2021.
USA, Washington	Centers for Disease Control and Prevention (CDC). CDC COVID Data Tracker Commercial Laboratory Seroprevalence Survey Data. Atlanta, United States of America: Centers for Disease Control and Prevention (CDC).
USA, Washington	Anand S, Montez-Rath M, Han J, Bozeman J, Kerschmann R, Beyer P, Parsonnet J, Chertow GM. Prevalence of SARS-CoV-2 antibodies in a large nationwide sample of patients on dialysis in the USA: a cross-sectional study. Lancet. 2020.
USA, Washington	McCulloch DJ, Jackson ML, Hughes JP, Lester S, Mills L, Freeman B, Rasheed MAU, Thornburg NJ, Chu HY. Seroprevalence of SARS-CoV-2 antibodies in Seattle, Washington: October 2019-April 2020. PLoS One. 2021; 16(5): e0252235.
USA, Washington, DC	Jones JM, Stone M, Sulaeman H, Fink RV, Dave H, Levy ME, Di Germanio C, Green V, Notari E, Saa P, Biggerstaff BJ, Strauss D, Kessler D, Vassallo R, Reik R, Rossmann S, Destree M, Nguyen KA, Sayers M, Lough C, Bougie DW, Ritter M, Latoni G, Weales B, Sime S, Gorlin J,

	Brown NE, Gould CV, Berney K, Benoit TJ, Miller MJ, Freeman D, Kartik D, Fry AM, Azziz-Baumgartner E, Hall AJ, MacNeil A, Gundlapalli AV, Basavaraju SV, Gerber SI, Patton ME, Custer B, Williamson P, Simmons G, Thornburg NJ, Kleinman S, Stramer SL, Opsomer J, Busch MP. Estimated US Infection- and Vaccine-Induced SARS-CoV-2 Seroprevalence Based on Blood Donations, July 2020-May 2021. JAMA. 2021.
USA, Washington, DC	Centers for Disease Control and Prevention (CDC). CDC COVID Data Tracker Commercial Laboratory Seroprevalence Survey Data. Atlanta, United States of America: Centers for Disease Control and Prevention (CDC).
USA, West Virginia	Jones JM, Stone M, Sulaeman H, Fink RV, Dave H, Levy ME, Di Germanio C, Green V, Notari E, Saa P, Biggerstaff BJ, Strauss D, Kessler D, Vassallo R, Reik R, Rossmann S, Destree M, Nguyen KA, Sayers M, Lough C, Bougie DW, Ritter M, Latoni G, Weales B, Sime S, Gorlin J, Brown NE, Gould CV, Berney K, Benoit TJ, Miller MJ, Freeman D, Kartik D, Fry AM, Azziz-Baumgartner E, Hall AJ, MacNeil A, Gundlapalli AV, Basavaraju SV, Gerber SI, Patton ME, Custer B, Williamson P, Simmons G, Thornburg NJ, Kleinman S, Stramer SL, Opsomer J, Busch MP. Estimated US Infection- and Vaccine-Induced SARS-CoV-2 Seroprevalence Based on Blood Donations, July 2020-May 2021. JAMA. 2021.
USA, West Virginia	Centers for Disease Control and Prevention (CDC). CDC COVID Data Tracker Commercial Laboratory Seroprevalence Survey Data. Atlanta, United States of America: Centers for Disease Control and Prevention (CDC).
USA, Wisconsin	Jones JM, Stone M, Sulaeman H, Fink RV, Dave H, Levy ME, Di Germanio C, Green V, Notari E, Saa P, Biggerstaff BJ, Strauss D, Kessler D, Vassallo R, Reik R, Rossmann S, Destree M, Nguyen KA, Sayers M, Lough C, Bougie DW, Ritter M, Latoni G, Weales B, Sime S, Gorlin J, Brown NE, Gould CV, Berney K, Benoit TJ, Miller MJ, Freeman D, Kartik D, Fry AM, Azziz-Baumgartner E, Hall AJ, MacNeil A, Gundlapalli AV, Basavaraju SV, Gerber SI, Patton ME, Custer B, Williamson P, Simmons G, Thornburg NJ, Kleinman S, Stramer SL, Opsomer J, Busch MP. Estimated US Infection- and Vaccine-Induced SARS-CoV-2 Seroprevalence Based on Blood Donations, July 2020-May 2021. JAMA. 2021.
USA, Wisconsin	Centers for Disease Control and Prevention (CDC). CDC COVID Data Tracker Commercial Laboratory Seroprevalence Survey Data. Atlanta, United States of America: Centers for Disease Control and Prevention (CDC).
USA, Wisconsin	Anand S, Montez-Rath M, Han J, Bozeman J, Kerschmann R, Beyer P, Parsonnet J, Chertow GM. Prevalence of SARS-CoV-2 antibodies in a large nationwide sample of patients on dialysis in the USA: a cross-sectional study. Lancet. 2020.
USA, Wyoming	Jones JM, Stone M, Sulaeman H, Fink RV, Dave H, Levy ME, Di Germanio C, Green V, Notari E, Saa P, Biggerstaff BJ, Strauss D, Kessler D, Vassallo R, Reik R, Rossmann S, Destree M, Nguyen KA, Sayers M, Lough C, Bougie DW, Ritter M, Latoni G, Weales B, Sime S, Gorlin J, Brown NE, Gould CV, Berney K, Benoit TJ, Miller MJ, Freeman D, Kartik D, Fry AM, Azziz-Baumgartner E, Hall AJ, MacNeil A, Gundlapalli AV, Basavaraju SV, Gerber SI, Patton ME, Custer B, Williamson P, Simmons G, Thornburg NJ, Kleinman S, Stramer SL, Opsomer J, Busch MP. Estimated US Infection- and Vaccine-Induced SARS-CoV-2 Seroprevalence Based on Blood Donations, July 2020-May 2021. JAMA. 2021.
USA, Wyoming	Centers for Disease Control and Prevention (CDC). CDC COVID Data Tracker Commercial Laboratory Seroprevalence Survey Data. Atlanta, United States of America: Centers for Disease Control and Prevention (CDC).
Uzbekistan	Rakhimov RA, Ibadullaeva NS, Khikmatullaeva AS, Abdukadirova MA, Sadirova SS, Lokteva LM, Rakhimov RR, Bayjanov AK, Samatova IR. Formation of Herd Immunity to SARS-CoV-2 in the Regions of Uzbekistan. European Journal of Molecular Clinical Medicine. 2021; 8(3): 574-81.
Yemen	*
Zambia	Mulenga LB, Hines JZ, Fwoloshi S, Chirwa L, Siwingwa M, Yingst S, Wolkon A, Barradas DT, Favaloro J, Zulu JE, Banda D, Nikoi KI, Kampamba D, Banda N, Chilopa B, Hanunka B, Stevens TL Jr, Shibemba A, Mwale C, Sivile S, Zyambo KD, Makupe A, Kapina M, Mweemba A, Sinyange N, Kapata N, Zulu PM, Chanda D, Mupeta F, Chilufya C, Mukonka V, Agolory S,

	Malama K. Prevalence of SARS-CoV-2 in six districts in Zambia in July, 2020: a cross-sectional cluster sample survey. Lancet Glob Health. 2021.

Section 4: Age-stratified seroprevalence sources

Figure S4: Age-stratified seroprevalence data coverage by location

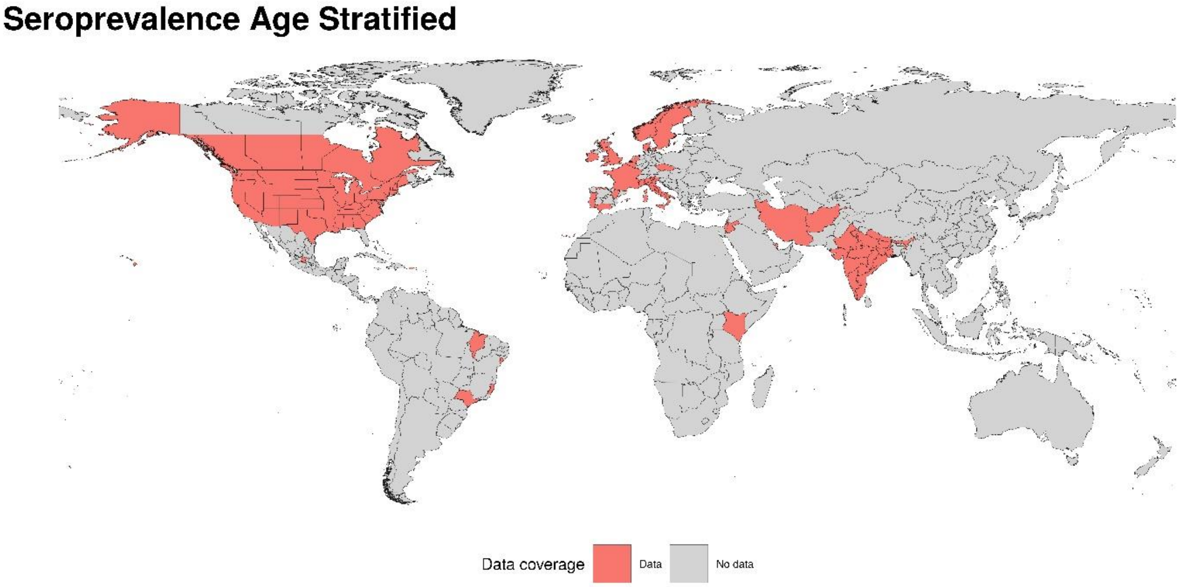

Table S4. Age-stratified seroprevalence data sources by location

Location	Source
Afghanistan	World Health Organization (WHO). Prevalence of COVID-19 and its Related Deaths in Afghanistan: A Nationwide, Population-Based Seroepidemiological Study July 2020. Kabul, Afghanistan: Ministry of Public Health (Afghanistan), 2021.
Andorra	Royo-Cebrecos C, Vilanova D, López J, Arroyo V, Francisco G, Pons M, Carrasco MG, Piqué JM, Sanz S, Dobaño C, García-Basteiro A. Mass SARS-CoV-2 serological screening for the Principality of Andorra. Preprint. Res Sq. 2020.
Belgium	Herzog S, De Bie J, Abrams S, Wouters I, Ekinci E, Patteet L, Coppens A, De Spiegeleer S, Beutels P, Van Damme P, Hens N, Theeten H. Seroprevalence of IgG antibodies against SARS coronavirus 2 in Belgium - a serial prospective cross-sectional nationwide study of residual samples. Preprint. medRxiv. 2020.
Brazil	Menezes AMB, Victora CG, Hartwig FP, Silveira MF, Horta BL, Barros AJD, Mesenburg MA, Wehrmeister FC, Pellanda LC, Dellagostin OA, Struchiner CJ, Burattini MN, Barros FC, Hallal PC. High prevalence of symptoms among Brazilian subjects with antibodies against SARS-CoV-2. Sci Rep. 2021; 11(1): 13279.
Brazil, Espírito Santo	Gomes CC, Cerutti Jr C, Zandonade E, Maciel ELN, Carvalho de Alencar FE, Almada GL, Cardoso OA, Medeiros Jabor P, Zanotti RL, Queiroz Reuter T, Gomes de Andrade VL, Bastos WM, Fernandes de Medeiros Jr N. A population-based study of the prevalence of COVID-19 infection in Espírito Santo, Brazil: methodology and results of the first stage. Preprint. medRxiv. 2020.
Brazil, Maranhão	Silva AAM da, Lima Neto LG, Azevedo C de MP e S de, Costa LMM da, Bragança MLBM, Barros Filho AKD, Wittlin BB, Souza BF de, Oliveira BLCA de, Carvalho CA de, Thomaz ÉBAF, Simões Neto EA, Leite Júnior JF, Cosme LMSS, Campos MAG, Queiroz RC de S, Costa SS, Carvalho VA de, Simóes VMF, Alves MTSS de B e, Santos AM dos. Population-based seroprevalence of SARS-CoV-2 is more than halfway through the herd immunity threshold in the State of Maranhao, Brazil. Preprint. medRxiv. 2020.

Brazil, Maranhão	Secretary of State for Health (Brazil - Maranhão). Prevalence of SARS-CoV-2 virus infection in Maranhão, Brazil, Final Research Report, Phase II, October 2020.
Brazil, São Paulo	Albuquerque JOM, Kamioka GA, Madalosso G, Costa SA, Ferreira PB, Pino FA, Sato APS, Carvalho ACA, Amorim ABP, Aires CC, Kataoka APAG, Savani ESMM, Bessa TAF, Aguiar BS, Failla MA, Santos EA, Brito EMT, Santos MCH, Silva SMS, Caldeira LAV, Zamarco LC, Fonseca SMS, Lima MMC, Marques IA, Silva FEV, Glasser PR, Burihan PCPR, Cavazzana CL, Lara RC, Mello DS, Pellini ACG, Nishio FY, Kian FM, Braga ES, Bertelli NMP, Fracini W, Gonçalves MDA, Zular PS, Piva RS, Masi E de. Prevalence evolution of SARS-CoV-2 infection in the Municipality of São Paulo, 2020 - 2021. medRxiv. 2021.
Brazil, Sergipe	Federal University of Sergipe (Brazil). Brazil - Evolution of the Prevalence of Infection by SARS-CoV-2 in Sergipe. Third Phase Results: February 18 to March 30, 2021.
Canada	Canadian Blood Services. Canada COVID-19 Seroprevalence Report - August 19, 2020. Ottawa, Canada: Canadian Blood Services, 2020.
Canada	Saeed S, Drews SJ, Pambrun C, Yi QL, Osmond L, O'Brien SF. SARS-CoV-2 seroprevalence among blood donors after the first COVID-19 wave in Canada. Transfusion. 2021; 61(3): 862-872.
Canada	Investigators A to beat coronavirus/Action pour battre le coronavirus (Ab-CS, Jha P. COVID Seroprevalence, Symptoms and Mortality During the First Wave of SARS-CoV-2 in Canada. medRxiv. 2021.
Canada	Canadian Blood Services. Canada COVID-19 Seroprevalence Report - December 18, 2020. Ottawa, Canada: Canadian Blood Services, 2020.
Canada	Canadian Blood Services. Canada COVID-19 Seroprevalence Report - January 12, 2021. Ottawa, Canada: Canadian Blood Services, 2021.
Canada	Canadian Blood Services. Canada COVID-19 Seroprevalence Report - April 2020 to January 2021. Ottawa, Canada: Canadian Blood Services, 2021.
Canada, Alberta	Canadian Blood Services. Canada COVID-19 Seroprevalence Report - December 18, 2020. Ottawa, Canada: Canadian Blood Services, 2020.
Canada, British Columbia	Canadian Blood Services. Canada COVID-19 Seroprevalence Report - December 18, 2020. Ottawa, Canada: Canadian Blood Services, 2020.
Canada, Manitoba	Canadian Blood Services. Canada COVID-19 Seroprevalence Report - December 18, 2020. Ottawa, Canada: Canadian Blood Services, 2020.
Canada, Ontario	Government of Ontario (Canada), Public Health Ontario (Canada). Canada COVID-19 Seroprevalence in Ontario: March 27, 2020 to June 30, 2020. Canada: Public Health Ontario (Canada), 2020.
Canada, Ontario	Canadian Blood Services. Canada COVID-19 Seroprevalence Report - December 18, 2020. Ottawa, Canada: Canadian Blood Services, 2020.
Canada, Ontario	Canadian Blood Services. Canada COVID-19 Seroprevalence Report - January 12, 2021. Ottawa, Canada: Canadian Blood Services, 2021.
Canada, Quebec	Hema-Quebec (Canada), Quebec National Institute of Public Health (Canada). Canada - Quebec COVID-19 First Wave Blood Donor Seroprevalence Study 2020. Canada: Hema-Quebec (Canada).
Canada, Saskatchewan	Canadian Blood Services. Canada COVID-19 Seroprevalence Report - December 18, 2020. Ottawa, Canada: Canadian Blood Services, 2020.
Czechia	Piler P, Thon V, Andrýsková L, Doležel K, Kostka D, Pavlík T, Dušek L, Pikhart H, Bobák M, Matic S, Klánová J. Dynamics of seroconversion of anti-SARS-CoV-2 IgG antibodies in the Czech unvaccinated population: nationwide prospective seroconversion (PROSECO) study. Preprint. medRxiv. 2021.

Denmark	Erikstrup C, Hother CE, Pedersen OBV, Mølbak K, Skov RL, Holm DK, Sækmose SG, Nilsson AC, Brooks PT, Boldsen JK, Mikkelsen C, Gybel-Brask M, Sørensen E, Dinh KM, Mikkelsen S, Møller BK, Haunstrup T, Harritshøj L, Jensen BA, Hjalgrim H, Lillevang ST, Ullum H. Estimation of SARS-CoV-2 infection fatality rate by real-time antibody screening of blood donors. Clin Infect Dis. 2020.
Denmark	Espenhain L, Tribler S, Jørgensen CS, Hansen CH, Sönksen UW, Ethelberg S. Prevalence of SARS-CoV-2 antibodies in Denmark 2020: results from nationwide, population-based sero-epidemiological surveys. Preprint. medRxiv. 2021.
France	Vu SL, Jones G, Anna F, Rose T, Richard J-B, Bernard-Stoecklin S, Goyard S, Demeret C, Helynck O, Robin C, Monnet V, Facci LP de, Ungeheuer M-N, Léon L, Guillois Y, Filleul L, Charneau P, Lévy-Bruhl D, van der Werf S, Noel H. Prevalence of SARS-CoV-2 antibodies in France: results from nationwide serological surveillance. Preprint. medRxiv. 2020.
India	Murhekar MV, Bhatnagar T, Selvaraju S, Rade K, Saravanakumar V, Vivian Thangaraj JW, Kumar MS, Shah N, Sabarinathan R, Turuk A, Anand PK, Asthana S, Balachandar R, Bangar SD, Bansal AK, Bhat J, Chakraborty D, Rangaraju C, Chopra V, Das D, Deb AK, Devi KR, Dwivedi GR, Salim Khan SM, Haq I, Kumar MS, Laxmaiah A, Madhuka, Mahapatra A, Mitra A, Nirmala AR, Pagdhune A, Qurieshi MA, Ramarao T, Sahay S, Sharma YK, Shrinivasa MB, Shukla VK, Singh PK, Viramgami A, Wilson VC, Yadav R, Girish Kumar CP, Luke HE, Ranganathan UD, Babu S, Sekar K, Yadav PD, Sapkal GN, Das A, Das P, Dutta S, Hemalatha R, Kumar A, Narain K, Narasimhaiah S, Panda S, Pati S, Patil S, Sarkar K, Singh S, Kant R, Tripathy S, Toteja GS, Babu GR, Kant S, Muliyil JP, Pandey RM, Sarkar S, Singh SK, Zodpey S, Gangakhedkar RR, S Reddy DC, Bhargava B. Prevalence of SARS-CoV-2 infection in India: Findings from the national serosurvey, May-June 2020. Indian J Med Res. 2020; 152(1,2): 48-60.
India	Murhekar MV, Bhatnagar T, Selvaraju S, Saravanakumar V, Thangaraj JWV, Shah N, Kumar MS, Rade K, Sabarinathan R, Asthana S, Balachandar R, Bangar SD, Bansal AK, Bhat J, Chopra V, Das D, Deb AK, Devi KR, Dwivedi GR, Khan SMS, Kumar CPG, Kumar MS, Laxmaiah A, Madhukar M, Mahapatra A, Mohanty SS, Rangaraju C, Turuk A, Baradwaj DK, Chahal AS, Debnath F, Haq I, Kalliath A, Kanungo S, Kshatri JS, Lakshmi GGJN, Mitra A, Nirmala AR, Prasad GV, Qurieshi MA, Sahay S, Sangwan RK, Sekar K, Shukla VK, Singh PK, Singh P, Singh R, Varma DS, Viramgami A, Panda S, Reddy DCS, Bhargava B, ICMR Serosurveillance Group. SARS-CoV-2 antibody seroprevalence in India, August-September, 2020: findings from the second nationwide household serosurvey. Lancet Glob Health. 2021; 9(3): e257-e266.
India	Murhekar MV, Bhatnagar T, Thangaraj J, Saravanakumar V, Kumar MS, Selvaraju S, Rade K, Kumar C, Sabarinathan R, Turuk A, Asthana S, Balachandar R, Bangar SD, Bansal AK, Chopra V, Das D, Deb AK, Devi KR, Dhikav V, Dwivedi GR, ICMR Serosurveillance Group (2021). I SARS-CoV-2 seroprevalence among the general population and healthcare workers in India, December 2020-January 2021. 2021; 108: 145-155.
India, Andhra Pradesh	*
India, Assam	*
India, Bihar	*
India, Chhattisgarh	*
India, Gujarat	*
India, Haryana	Health Department, Haryana (India). India - Haryana Covid-19 Sero Survey Round 2, October 2020.
India, Jharkhand	*
India, Karnataka	*
India, Kerala	*
India, Madhya Pradesh	*
India, Maharashtra	*

India, Odisha	*
India, Punjab	*
India, Rajasthan	*
India, Tamil Nadu	*
India, Telangana	*
India, Uttar Pradesh	*
India, West Bengal	*
Iran	Khalagi K, Gharibzadeh S, Khalili D, Mansournia MA, Mirab Samiee S, Aghamohamadi S, Mir-Mohammad-Ali Roodaki M, Hashemi SM, Tayeri K, Namdari Tabar H, Azadmanesh K, Tabrizi JS, Mohammad K, Hajipour F, Namaki S, Raeisi A, Ostovar A. Prevalence of COVID-19 in Iran: results of the first survey of the Iranian COVID-19 Serological Surveillance programme. Clin Microbiol Infect. 2021.
Ireland	Health Service Executive (HSE) (Ireland), University College Dublin. Ireland Study to Investigate COVID-19 Infection in People Living in Ireland (SCOPI) 2020. Dublin, Ireland: Health Protection Surveillance Centre (HPSC) (Ireland), 2020.
Israel	Reicher S, Ratzon R, Ben-Sahar S, Hermoni-Alon S, Mossinson D, Shenhar Y, Friger M, Lustig Y, Alroy-Preis S, Anis E, Sadetzki S, Kaliner E. Nationwide seroprevalence of antibodies against SARS-CoV-2 in Israel. Eur J Epidemiol. 2021.
Italy	Ministry of Health (Italy), National Institute of Statistics (Italy). Italy SARS-CoV-2 Seroprevalence Survey, First Results 2020.
Jordan	Bellizzi S, Alsawalha L, Sheikh Ali S, Sharkas G, Muthu N, Ghazo M, Hayajneh W, Profili MC, Obeidat NM. A three-phase population based sero-epidemiological study: Assessing the trend in prevalence of SARS-CoV-2 during COVID-19 pandemic in Jordan. One Health. 2021; 13: 100292.
Jordan	Sughayer MA, Mansour A, Nuirat AA, Souan L, Ghanem M, Siag M, Alhassoon S. Dramatic Rise of Seroprevalence Rates of SARS-CoV-2 Antibodies among Healthy Blood Donors: The evolution of a Pandemic. Preprint. medRxiv. 2021.
Kenya	Uyoga S, Adetifa IMO, Karanja HK, Nyagwange J, Tuju J, Wanjiku P, Aman R, Mwangangi M, Amoth P, Kasera K, Ng'ang'a W, Rombo C, Yegon CK, Kithi K, Odhiambo E, Rotich T, Orgut I, Kihara S, Otiende M, Bottomley C, Mupe ZN, Kagucia EW, Gallagher K, Etyang A, Voller S, Gitonga J, Mugo D, Agoti CN, Otieno E, Ndwiga L, Lambe T, Wright D, Barasa E, Tsofa B, Bejon P, Ochola-Oyier LI, Agweyu A, Scott AGJ, Warimwe GM. Seroprevalence of anti-SARS-CoV-2 IgG antibodies in Kenyan blood donors. Preprint. medRxiv. 2020.
Kenya	Adetifa IMO, Uyoga S, Gitonga JN, Mugo D, Otiende M, Nyagwange J, Karanja HK, Tuju J, Wanjiku P, Aman R, Mwangangi M, Amoth P, Kasera K, Nganga W, Rombo C, Yegon C, Kithi K, Odhiambo E, Rotich T, Orgut I, Kihara S, Bottomley C, Kagucia EW, Gallagher KE, Etyang A, Voller S, Lambe T, Wright D, Barasa E, Tsofa B, Bejon P, Ochola-Oyier LI, Agweyu A, Scott JAG, Warimwe GM. Temporal trends of SARS-CoV-2 seroprevalence in transfusion blood donors during the first wave of the COVID-19 epidemic in Kenya. medRxiv. 2021.
Mexico	Government of Mexico, National Institute of Public Health (Mexico). Mexico National Health and Nutrition Survey (ENSANUT) about COVID-19 2020. Cuernavaca, Mexico: National Institute of Public Health (Mexico), 2020.
Mexico	Muñoz-Medina JE, Grajales-Muñiz C, Salas-Lais AG, Fernandes-Matano L, López-Macías C, Monroy-Muñoz IE, Santos Coy-Arechavaleta A, Palomec-Nava ID, Duque-Molina C, Madera-Sandoval RL, Rivero-Arredondo V, González-Ibarra J, Alvarado-Yaah JE, Rojas-Mendoza T, Santacruz-Tinoco CE, González-Bonilla CR, Borja-Aburto VH. SARS-CoV-2 IgG Antibodies Seroprevalence and Sera Neutralizing Activity in MEXICO: A National Cross-Sectional Study during 2020. Microorganisms. 2021; 9(4).

Mexico, Guanajuato	Government of Mexico, National Institute of Public Health (Mexico). Mexico National Health and Nutrition Survey (ENSANUT) about COVID-19 2020. Cuernavaca, Mexico: National Institute of Public Health (Mexico), 2020.
Nepal	Ministry of Health and Population (Nepal), National Public Health Laboratory (Nepal), World Health Organization (WHO), World Health Organization Regional Office for South-East Asia (SEARO). Nepal National Sero-Prevalence Survey for COVID-19 October 2020.
Netherlands	Slot E, Hogema BM, Reusken CBEM, Reimerink JH, Molier M, Karregat JHM, IJlst J, Novotný VMJ, van Lier RAW, Zaaijer HL. Low SARS-CoV-2 seroprevalence in blood donors in the early COVID-19 epidemic in the Netherlands. Nat Commun. 2020; 11(1): 5744.
Netherlands	Vos ERA, den Hartog G, Schepp RM, Kaaijk P, van Vliet J, Helm K, Smits G, Wijmenga-Monsuur A, Verberk JDM, van Boven M, van Binnendijk RS, de Melker HE, Mollema L, van der Klis FRM. Nationwide seroprevalence of SARS-CoV-2 and identification of risk factors in the general population of the Netherlands during the first epidemic wave. J Epidemiol Community Health. 2020.
Norway	Norwegian Institute of Public Health. Seroprevalence of SARS-CoV-2 in the Norwegian population measured in residual sera collected in April/May 2020 and August 2019. Oslo, Norway: Norwegian Institute of Public Health, 2020.
Norway	Tunheim G., Kran, AB., Rø, G., Hungnes O., Lund-Johansen, F., Tran, T., Andersen JT., Vaage, JT. "Seroprevalence of SARS-CoV-2 in the Norwegian population measured in residual sera collected in late summer 2020". [Seroprevalens av SARS-CoV-2 i den norske befolkningen, målt i restsera samlet inn på sensommeren 2020] Report 2020. Oslo: Norwegian Institute of Public Health, 2020.
Portugal	Ministry of Health (Portugal). Portugal National Serological Survey COVID-19 Preliminary results 2020. Lisbon, Portugal: Ministry of Health (Portugal), 2020.
Puerto Rico	Centers for Disease Control and Prevention (CDC). CDC COVID Data Tracker Commercial Laboratory Seroprevalence Survey Data. Atlanta, United States of America: Centers for Disease Control and Prevention (CDC).
Puerto Rico	Jones JM, Stone M, Sulaeman H, Fink RV, Dave H, Levy ME, Di Germanio C, Green V, Notari E, Saa P, Biggerstaff BJ, Strauss D, Kessler D, Vassallo R, Reik R, Rossmann S, Destree M, Nguyen KA, Sayers M, Lough C, Bougie DW, Ritter M, Latoni G, Weales B, Sime S, Gorlin J, Brown NE, Gould CV, Berney K, Benoit TJ, Miller MJ, Freeman D, Kartik D, Fry AM, Azziz-Baumgartner E, Hall AJ, MacNeil A, Gundlapalli AV, Basavaraju SV, Gerber SI, Patton ME, Custer B, Williamson P, Simmons G, Thornburg NJ, Kleinman S, Stramer SL, Opsomer J, Busch MP. Estimated US Infection- and Vaccine-Induced SARS-CoV-2 Seroprevalence Based on Blood Donations, July 2020-May 2021. JAMA. 2021.
Qatar	Abu-Raddad LJ, Chemaitelly H, Ayoub HH, Al Kanaani Z, Al Khal A, Al Kuwari E, Butt AA, Coyle P, Jeremijenko A, Kaleeckal AH, Latif AN, Owen RC, Rahim HFA, Al Abdulla SA, Al Kuwari MG, Kandy MC, Saeb H, Ahmed SNN, Al Romaihi HE, Bansal D, Dalton L, Al-Thani MH, Bertollini R. Characterizing the Qatar advanced-phase SARS-CoV-2 epidemic. Sci Rep. 2021; 11(1): 6233.
Spain	Government of Spain, Institute of Health Carlos III (Spain). Spain - National Study of Sero-epidemiology of Infection by Sars-Cov-2.
Spain, Andalusia	Government of Spain, Institute of Health Carlos III (Spain). Spain - National Study of Sero-epidemiology of Infection by Sars-Cov-2.
Spain, Aragon	Government of Spain, Institute of Health Carlos III (Spain). Spain - National Study of Sero-epidemiology of Infection by Sars-Cov-2.
Spain, Asturias	Government of Spain, Institute of Health Carlos III (Spain). Spain - National Study of Sero-epidemiology of Infection by Sars-Cov-2.

Spain, Balearic Islands	Government of Spain, Institute of Health Carlos III (Spain). Spain - National Study of Sero-epidemiology of Infection by Sars-Cov-2.
Spain, Canary Islands	Government of Spain, Institute of Health Carlos III (Spain). Spain - National Study of Sero-epidemiology of Infection by Sars-Cov-2.
Spain, Cantabria	Iruzubieta P, Fernández-Lanas T, Rasines L, Cayon L, Álvarez-Cancelo A, Santos-Laso A, García-Blanco A, Curiel-Olmo S, Cabezas J, Wallmann R, Fábrega E, Martínez-Taboada VM, Hernández JL, López-Hoyos M, Lazarus JV, Crespo J. Feasibility of large-scale population testing for SARS-CoV-2 detection by self-testing at home. Sci Rep. 2021; 11(1): 9819.
Sweden	Public Health Agency of Sweden. Detection of Antibodies After Review of Covid-19 in Blood Donors (Sub-report 2). Östersund, Sweden: Public Health Agency of Sweden.
UK, England	National Health Service (United Kingdom), Public Health England. United Kingdom - England Weekly National COVID-19 Surveillance Report. London, United Kingdom: Government of the United Kingdom.
UK, England	Ward H, Atchison CJ, Whitaker M, Ainslie KEC, Elliott J, Okell LC, Redd R, Ashby D, Donnelly CA, Barclay W, Darzi A, Cooke G, Riley S, Elliott P. Antibody prevalence for SARS-CoV-2 in England following first peak of the pandemic: REACT2 study in 100,000 adults. Preprint. medRxiv. 2020.
UK, England	Office for National Statistics (ONS) (United Kingdom). United Kingdom Coronavirus (COVID-19) Infection Survey. Newport, United Kingdom: Office for National Statistics (ONS) (United Kingdom), 2020.
UK, England	Ward H, Cooke G, Atchison C, Whitaker M, Elliott J, Moshe M, Brown JC, Flower B, Daunt A, Ainslie K, Ashby D, Donnelly C, Riley S, Darzi A, Barclay W, Elliott P, for the React study team. Declining prevalence of antibody positivity to SARS-CoV-2: a community study of 365,000 adults. Preprint. medRxiv. 2020.
UK, England	Office for National Statistics (ONS) (United Kingdom). England - Coronavirus (COVID-19) Infection Survey: Antibody Data, January 2021. Newport, United Kingdom: Office for National Statistics (ONS) (United Kingdom), 2021.
UK, Scotland	Public Health Scotland, Scottish National Blood Transfusion Service (SNBTS), The Centre for Virus Research (CVR) at the University of Glasgow. Scotland Enhanced Surveillance of Covid-19 Dashboard. Edinburgh, Scotland: Public Health Scotland.
UK, Scotland	Office for National Statistics (ONS) (United Kingdom). England - Coronavirus (COVID-19) Infection Survey: Antibody Data, January 2021. Newport, United Kingdom: Office for National Statistics (ONS) (United Kingdom), 2021.
UK, Wales	Office for National Statistics (ONS) (United Kingdom). England - Coronavirus (COVID-19) Infection Survey: Antibody Data, January 2021. Newport, United Kingdom: Office for National Statistics (ONS) (United Kingdom), 2021.
USA, Alabama	Centers for Disease Control and Prevention (CDC). CDC COVID Data Tracker Commercial Laboratory Seroprevalence Survey Data. Atlanta, United States of America: Centers for Disease Control and Prevention (CDC).
USA, Alabama	Jones JM, Stone M, Sulaeman H, Fink RV, Dave H, Levy ME, Di Germanio C, Green V, Notari E, Saa P, Biggerstaff BJ, Strauss D, Kessler D, Vassallo R, Reik R, Rossmann S, Destree M, Nguyen KA, Sayers M, Lough C, Bougie DW, Ritter M, Latoni G, Weales B, Sime S, Gorlin J, Brown NE, Gould CV, Berney K, Benoit TJ, Miller MJ, Freeman D, Kartik D, Fry AM, Azziz-Baumgartner E, Hall AJ, MacNeil A, Gundlapalli AV, Basavaraju SV, Gerber SI, Patton ME, Custer B, Williamson P, Simmons G, Thornburg NJ, Kleinman S, Stramer SL, Opsomer J, Busch MP. Estimated US Infection- and Vaccine-Induced SARS-CoV-2 Seroprevalence Based on Blood Donations, July 2020-May 2021. JAMA. 2021.
USA, Alaska	Centers for Disease Control and Prevention (CDC). CDC COVID Data Tracker Commercial Laboratory Seroprevalence Survey Data. Atlanta, United States of America: Centers for Disease Control and Prevention (CDC).

USA, Alaska	Jones JM, Stone M, Sulaeman H, Fink RV, Dave H, Levy ME, Di Germanio C, Green V, Notari E, Saa P, Biggerstaff BJ, Strauss D, Kessler D, Vassallo R, Reik R, Rossmann S, Destree M, Nguyen KA, Sayers M, Lough C, Bougie DW, Ritter M, Latoni G, Weales B, Sime S, Gorlin J, Brown NE, Gould CV, Berney K, Benoit TJ, Miller MJ, Freeman D, Kartik D, Fry AM, Azziz-Baumgartner E, Hall AJ, MacNeil A, Gundlapalli AV, Basavaraju SV, Gerber SI, Patton ME, Custer B, Williamson P, Simmons G, Thornburg NJ, Kleinman S, Stramer SL, Opsomer J, Busch MP. Estimated US Infection- and Vaccine-Induced SARS-CoV-2 Seroprevalence Based on Blood Donations, July 2020-May 2021. JAMA. 2021.
USA, Arizona	Centers for Disease Control and Prevention (CDC). CDC COVID Data Tracker Commercial Laboratory Seroprevalence Survey Data. Atlanta, United States of America: Centers for Disease Control and Prevention (CDC).
USA, Arkansas	Centers for Disease Control and Prevention (CDC). CDC COVID Data Tracker Commercial Laboratory Seroprevalence Survey Data. Atlanta, United States of America: Centers for Disease Control and Prevention (CDC).
USA, California	Centers for Disease Control and Prevention (CDC). CDC COVID Data Tracker Commercial Laboratory Seroprevalence Survey Data. Atlanta, United States of America: Centers for Disease Control and Prevention (CDC).
USA, Colorado	Centers for Disease Control and Prevention (CDC). CDC COVID Data Tracker Commercial Laboratory Seroprevalence Survey Data. Atlanta, United States of America: Centers for Disease Control and Prevention (CDC).
USA, Colorado	Jones JM, Stone M, Sulaeman H, Fink RV, Dave H, Levy ME, Di Germanio C, Green V, Notari E, Saa P, Biggerstaff BJ, Strauss D, Kessler D, Vassallo R, Reik R, Rossmann S, Destree M, Nguyen KA, Sayers M, Lough C, Bougie DW, Ritter M, Latoni G, Weales B, Sime S, Gorlin J, Brown NE, Gould CV, Berney K, Benoit TJ, Miller MJ, Freeman D, Kartik D, Fry AM, Azziz-Baumgartner E, Hall AJ, MacNeil A, Gundlapalli AV, Basavaraju SV, Gerber SI, Patton ME, Custer B, Williamson P, Simmons G, Thornburg NJ, Kleinman S, Stramer SL, Opsomer J, Busch MP. Estimated US Infection- and Vaccine-Induced SARS-CoV-2 Seroprevalence Based on Blood Donations, July 2020-May 2021. JAMA. 2021.
USA, Connecticut	Havers FP, Reed C, Lim T, Montgomery JM, Klena JD, Hall AJ, Fry AM, Cannon DL, Chiang CF, Gibbons A, Krapiunaya I, Morales-Betoulle M, Roguski K, Rasheed MAU, Freeman B, Lester S, Mills L, Carroll DS, Owen SM, Johnson JA, Semenova V, Blackmore C, Blog D, Chai SJ, Dunn A, Hand J, Jain S, Lindquist S, Lynfield R, Pritchard S, Sokol T, Sosa L, Turabelidze G, Watkins SM, Wiesman J, Williams RW, Yendell S, Schiffer J, Thornburg NJ. Seroprevalence of Antibodies to SARS-CoV-2 in 10 Sites in the United States, March 23-May 12, 2020. JAMA Intern Med. 2020.
USA, Connecticut	Centers for Disease Control and Prevention (CDC). CDC COVID Data Tracker Commercial Laboratory Seroprevalence Survey Data. Atlanta, United States of America: Centers for Disease Control and Prevention (CDC).
USA, Connecticut	Jones JM, Stone M, Sulaeman H, Fink RV, Dave H, Levy ME, Di Germanio C, Green V, Notari E, Saa P, Biggerstaff BJ, Strauss D, Kessler D, Vassallo R, Reik R, Rossmann S, Destree M, Nguyen KA, Sayers M, Lough C, Bougie DW, Ritter M, Latoni G, Weales B, Sime S, Gorlin J, Brown NE, Gould CV, Berney K, Benoit TJ, Miller MJ, Freeman D, Kartik D, Fry AM, Azziz-Baumgartner E, Hall AJ, MacNeil A, Gundlapalli AV, Basavaraju SV, Gerber SI, Patton ME, Custer B, Williamson P, Simmons G, Thornburg NJ, Kleinman S, Stramer SL, Opsomer J, Busch MP. Estimated US Infection- and Vaccine-Induced SARS-CoV-2 Seroprevalence Based on Blood Donations, July 2020-May 2021. JAMA. 2021.
USA, Delaware	Centers for Disease Control and Prevention (CDC). CDC COVID Data Tracker Commercial Laboratory Seroprevalence Survey Data. Atlanta, United States of America: Centers for Disease Control and Prevention (CDC).
USA, Georgia	Centers for Disease Control and Prevention (CDC). CDC COVID Data Tracker Commercial Laboratory Seroprevalence Survey Data. Atlanta, United States of America: Centers for Disease Control and Prevention (CDC).

USA, Georgia	Jones JM, Stone M, Sulaeman H, Fink RV, Dave H, Levy ME, Di Germanio C, Green V, Notari E, Saa P, Biggerstaff BJ, Strauss D, Kessler D, Vassallo R, Reik R, Rossmann S, Destree M, Nguyen KA, Sayers M, Lough C, Bougie DW, Ritter M, Latoni G, Weales B, Sime S, Gorlin J, Brown NE, Gould CV, Berney K, Benoit TJ, Miller MJ, Freeman D, Kartik D, Fry AM, Azziz-Baumgartner E, Hall AJ, MacNeil A, Gundlapalli AV, Basavaraju SV, Gerber SI, Patton ME, Custer B, Williamson P, Simmons G, Thornburg NJ, Kleinman S, Stramer SL, Opsomer J, Busch MP. Estimated US Infection- and Vaccine-Induced SARS-CoV-2 Seroprevalence Based on Blood Donations, July 2020-May 2021. JAMA. 2021.
USA, Hawaii	Centers for Disease Control and Prevention (CDC). CDC COVID Data Tracker Commercial Laboratory Seroprevalence Survey Data. Atlanta, United States of America: Centers for Disease Control and Prevention (CDC).
USA, Hawaii	Jones JM, Stone M, Sulaeman H, Fink RV, Dave H, Levy ME, Di Germanio C, Green V, Notari E, Saa P, Biggerstaff BJ, Strauss D, Kessler D, Vassallo R, Reik R, Rossmann S, Destree M, Nguyen KA, Sayers M, Lough C, Bougie DW, Ritter M, Latoni G, Weales B, Sime S, Gorlin J, Brown NE, Gould CV, Berney K, Benoit TJ, Miller MJ, Freeman D, Kartik D, Fry AM, Azziz-Baumgartner E, Hall AJ, MacNeil A, Gundlapalli AV, Basavaraju SV, Gerber SI, Patton ME, Custer B, Williamson P, Simmons G, Thornburg NJ, Kleinman S, Stramer SL, Opsomer J, Busch MP. Estimated US Infection- and Vaccine-Induced SARS-CoV-2 Seroprevalence Based on Blood Donations, July 2020-May 2021. JAMA. 2021.
USA, Idaho	Centers for Disease Control and Prevention (CDC). CDC COVID Data Tracker Commercial Laboratory Seroprevalence Survey Data. Atlanta, United States of America: Centers for Disease Control and Prevention (CDC).
USA, Idaho	Jones JM, Stone M, Sulaeman H, Fink RV, Dave H, Levy ME, Di Germanio C, Green V, Notari E, Saa P, Biggerstaff BJ, Strauss D, Kessler D, Vassallo R, Reik R, Rossmann S, Destree M, Nguyen KA, Sayers M, Lough C, Bougie DW, Ritter M, Latoni G, Weales B, Sime S, Gorlin J, Brown NE, Gould CV, Berney K, Benoit TJ, Miller MJ, Freeman D, Kartik D, Fry AM, Azziz-Baumgartner E, Hall AJ, MacNeil A, Gundlapalli AV, Basavaraju SV, Gerber SI, Patton ME, Custer B, Williamson P, Simmons G, Thornburg NJ, Kleinman S, Stramer SL, Opsomer J, Busch MP. Estimated US Infection- and Vaccine-Induced SARS-CoV-2 Seroprevalence Based on Blood Donations, July 2020-May 2021. JAMA. 2021.
USA, Illinois	Centers for Disease Control and Prevention (CDC). CDC COVID Data Tracker Commercial Laboratory Seroprevalence Survey Data. Atlanta, United States of America: Centers for Disease Control and Prevention (CDC).
USA, Indiana	Menachemi N, Yiannoutsos CT, Dixon BE, Duszynski TJ, Fadel WF, Wools-Kaloustian KK, Unruh Needleman N, Box K, Caine V, Norwood C, Weaver L, Halverson PK. Population Point Prevalence of SARS-CoV-2 Infection Based on a Statewide Random Sample - Indiana, April 25-29, 2020. MMWR Morb Mortal Wkly Rep. 2020; 69(29): 960-964.
USA, Indiana	Centers for Disease Control and Prevention (CDC). CDC COVID Data Tracker Commercial Laboratory Seroprevalence Survey Data. Atlanta, United States of America: Centers for Disease Control and Prevention (CDC).
USA, Indiana	Jones JM, Stone M, Sulaeman H, Fink RV, Dave H, Levy ME, Di Germanio C, Green V, Notari E, Saa P, Biggerstaff BJ, Strauss D, Kessler D, Vassallo R, Reik R, Rossmann S, Destree M, Nguyen KA, Sayers M, Lough C, Bougie DW, Ritter M, Latoni G, Weales B, Sime S, Gorlin J, Brown NE, Gould CV, Berney K, Benoit TJ, Miller MJ, Freeman D, Kartik D, Fry AM, Azziz-Baumgartner E, Hall AJ, MacNeil A, Gundlapalli AV, Basavaraju SV, Gerber SI, Patton ME, Custer B, Williamson P, Simmons G, Thornburg NJ, Kleinman S, Stramer SL, Opsomer J, Busch MP. Estimated US Infection- and Vaccine-Induced SARS-CoV-2 Seroprevalence Based on Blood Donations, July 2020-May 2021. JAMA. 2021.
USA, Iowa	Centers for Disease Control and Prevention (CDC). CDC COVID Data Tracker Commercial Laboratory Seroprevalence Survey Data. Atlanta, United States of America: Centers for Disease Control and Prevention (CDC).

USA, Iowa	Jones JM, Stone M, Sulaeman H, Fink RV, Dave H, Levy ME, Di Germanio C, Green V, Notari E, Saa P, Biggerstaff BJ, Strauss D, Kessler D, Vassallo R, Reik R, Rossmann S, Destree M, Nguyen KA, Sayers M, Lough C, Bougie DW, Ritter M, Latoni G, Weales B, Sime S, Gorlin J, Brown NE, Gould CV, Berney K, Benoit TJ, Miller MJ, Freeman D, Kartik D, Fry AM, Azziz-Baumgartner E, Hall AJ, MacNeil A, Gundlapalli AV, Basavaraju SV, Gerber SI, Patton ME, Custer B, Williamson P, Simmons G, Thornburg NJ, Kleinman S, Stramer SL, Opsomer J, Busch MP. Estimated US Infection- and Vaccine-Induced SARS-CoV-2 Seroprevalence Based on Blood Donations, July 2020-May 2021. JAMA. 2021.
USA, Kansas	Centers for Disease Control and Prevention (CDC). CDC COVID Data Tracker Commercial Laboratory Seroprevalence Survey Data. Atlanta, United States of America: Centers for Disease Control and Prevention (CDC).
USA, Kentucky	Centers for Disease Control and Prevention (CDC). CDC COVID Data Tracker Commercial Laboratory Seroprevalence Survey Data. Atlanta, United States of America: Centers for Disease Control and Prevention (CDC).
USA, Louisiana	Havers FP, Reed C, Lim T, Montgomery JM, Klena JD, Hall AJ, Fry AM, Cannon DL, Chiang CF, Gibbons A, Krapiunaya I, Morales-Betoulle M, Roguski K, Rasheed MAU, Freeman B, Lester S, Mills L, Carroll DS, Owen SM, Johnson JA, Semenova V, Blackmore C, Blog D, Chai SJ, Dunn A, Hand J, Jain S, Lindquist S, Lynfield R, Pritchard S, Sokol T, Sosa L, Turabelidze G, Watkins SM, Wiesman J, Williams RW, Yendell S, Schiffer J, Thornburg NJ. Seroprevalence of Antibodies to SARS-CoV-2 in 10 Sites in the United States, March 23-May 12, 2020. JAMA Intern Med. 2020.
USA, Louisiana	Centers for Disease Control and Prevention (CDC). CDC COVID Data Tracker Commercial Laboratory Seroprevalence Survey Data. Atlanta, United States of America: Centers for Disease Control and Prevention (CDC).
USA, Louisiana	Jones JM, Stone M, Sulaeman H, Fink RV, Dave H, Levy ME, Di Germanio C, Green V, Notari E, Saa P, Biggerstaff BJ, Strauss D, Kessler D, Vassallo R, Reik R, Rossmann S, Destree M, Nguyen KA, Sayers M, Lough C, Bougie DW, Ritter M, Latoni G, Weales B, Sime S, Gorlin J, Brown NE, Gould CV, Berney K, Benoit TJ, Miller MJ, Freeman D, Kartik D, Fry AM, Azziz-Baumgartner E, Hall AJ, MacNeil A, Gundlapalli AV, Basavaraju SV, Gerber SI, Patton ME, Custer B, Williamson P, Simmons G, Thornburg NJ, Kleinman S, Stramer SL, Opsomer J, Busch MP. Estimated US Infection- and Vaccine-Induced SARS-CoV-2 Seroprevalence Based on Blood Donations, July 2020-May 2021. JAMA. 2021.
USA, Maine	Centers for Disease Control and Prevention (CDC). CDC COVID Data Tracker Commercial Laboratory Seroprevalence Survey Data. Atlanta, United States of America: Centers for Disease Control and Prevention (CDC).
USA, Maine	Jones JM, Stone M, Sulaeman H, Fink RV, Dave H, Levy ME, Di Germanio C, Green V, Notari E, Saa P, Biggerstaff BJ, Strauss D, Kessler D, Vassallo R, Reik R, Rossmann S, Destree M, Nguyen KA, Sayers M, Lough C, Bougie DW, Ritter M, Latoni G, Weales B, Sime S, Gorlin J, Brown NE, Gould CV, Berney K, Benoit TJ, Miller MJ, Freeman D, Kartik D, Fry AM, Azziz-Baumgartner E, Hall AJ, MacNeil A, Gundlapalli AV, Basavaraju SV, Gerber SI, Patton ME, Custer B, Williamson P, Simmons G, Thornburg NJ, Kleinman S, Stramer SL, Opsomer J, Busch MP. Estimated US Infection- and Vaccine-Induced SARS-CoV-2 Seroprevalence Based on Blood Donations, July 2020-May 2021. JAMA. 2021.
USA, Maryland	Centers for Disease Control and Prevention (CDC). CDC COVID Data Tracker Commercial Laboratory Seroprevalence Survey Data. Atlanta, United States of America: Centers for Disease Control and Prevention (CDC).
USA, Massachusetts	Centers for Disease Control and Prevention (CDC). CDC COVID Data Tracker Commercial Laboratory Seroprevalence Survey Data. Atlanta, United States of America: Centers for Disease Control and Prevention (CDC).

USA, Massachusetts	Jones JM, Stone M, Sulaeman H, Fink RV, Dave H, Levy ME, Di Germanio C, Green V, Notari E, Saa P, Biggerstaff BJ, Strauss D, Kessler D, Vassallo R, Reik R, Rossmann S, Destree M, Nguyen KA, Sayers M, Lough C, Bougie DW, Ritter M, Latoni G, Weales B, Sime S, Gorlin J, Brown NE, Gould CV, Berney K, Benoit TJ, Miller MJ, Freeman D, Kartik D, Fry AM, Azziz-Baumgartner E, Hall AJ, MacNeil A, Gundlapalli AV, Basavaraju SV, Gerber SI, Patton ME, Custer B, Williamson P, Simmons G, Thornburg NJ, Kleinman S, Stramer SL, Opsomer J, Busch MP. Estimated US Infection- and Vaccine-Induced SARS-CoV-2 Seroprevalence Based on Blood Donations, July 2020-May 2021. JAMA. 2021.
USA, Michigan	Centers for Disease Control and Prevention (CDC). CDC COVID Data Tracker Commercial Laboratory Seroprevalence Survey Data. Atlanta, United States of America: Centers for Disease Control and Prevention (CDC).
USA, Michigan	Jones JM, Stone M, Sulaeman H, Fink RV, Dave H, Levy ME, Di Germanio C, Green V, Notari E, Saa P, Biggerstaff BJ, Strauss D, Kessler D, Vassallo R, Reik R, Rossmann S, Destree M, Nguyen KA, Sayers M, Lough C, Bougie DW, Ritter M, Latoni G, Weales B, Sime S, Gorlin J, Brown NE, Gould CV, Berney K, Benoit TJ, Miller MJ, Freeman D, Kartik D, Fry AM, Azziz-Baumgartner E, Hall AJ, MacNeil A, Gundlapalli AV, Basavaraju SV, Gerber SI, Patton ME, Custer B, Williamson P, Simmons G, Thornburg NJ, Kleinman S, Stramer SL, Opsomer J, Busch MP. Estimated US Infection- and Vaccine-Induced SARS-CoV-2 Seroprevalence Based on Blood Donations, July 2020-May 2021. JAMA. 2021.
USA, Minnesota	Centers for Disease Control and Prevention (CDC). CDC COVID Data Tracker Commercial Laboratory Seroprevalence Survey Data. Atlanta, United States of America: Centers for Disease Control and Prevention (CDC).
USA, Mississippi	Centers for Disease Control and Prevention (CDC). CDC COVID Data Tracker Commercial Laboratory Seroprevalence Survey Data. Atlanta, United States of America: Centers for Disease Control and Prevention (CDC).
USA, Mississippi	Jones JM, Stone M, Sulaeman H, Fink RV, Dave H, Levy ME, Di Germanio C, Green V, Notari E, Saa P, Biggerstaff BJ, Strauss D, Kessler D, Vassallo R, Reik R, Rossmann S, Destree M, Nguyen KA, Sayers M, Lough C, Bougie DW, Ritter M, Latoni G, Weales B, Sime S, Gorlin J, Brown NE, Gould CV, Berney K, Benoit TJ, Miller MJ, Freeman D, Kartik D, Fry AM, Azziz-Baumgartner E, Hall AJ, MacNeil A, Gundlapalli AV, Basavaraju SV, Gerber SI, Patton ME, Custer B, Williamson P, Simmons G, Thornburg NJ, Kleinman S, Stramer SL, Opsomer J, Busch MP. Estimated US Infection- and Vaccine-Induced SARS-CoV-2 Seroprevalence Based on Blood Donations, July 2020-May 2021. JAMA. 2021.
USA, Missouri	Havers FP, Reed C, Lim T, Montgomery JM, Klena JD, Hall AJ, Fry AM, Cannon DL, Chiang CF, Gibbons A, Krapiunaya I, Morales-Betoulle M, Roguski K, Rasheed MAU, Freeman B, Lester S, Mills L, Carroll DS, Owen SM, Johnson JA, Semenova V, Blackmore C, Blog D, Chai SJ, Dunn A, Hand J, Jain S, Lindquist S, Lynfield R, Pritchard S, Sokol T, Sosa L, Turabelidze G, Watkins SM, Wiesman J, Williams RW, Yendell S, Schiffer J, Thornburg NJ. Seroprevalence of Antibodies to SARS-CoV-2 in 10 Sites in the United States, March 23-May 12, 2020. JAMA Intern Med. 2020.
USA, Missouri	Centers for Disease Control and Prevention (CDC). CDC COVID Data Tracker Commercial Laboratory Seroprevalence Survey Data. Atlanta, United States of America: Centers for Disease Control and Prevention (CDC).
USA, Montana	Centers for Disease Control and Prevention (CDC). CDC COVID Data Tracker Commercial Laboratory Seroprevalence Survey Data. Atlanta, United States of America: Centers for Disease Control and Prevention (CDC).
USA, Montana	Jones JM, Stone M, Sulaeman H, Fink RV, Dave H, Levy ME, Di Germanio C, Green V, Notari E, Saa P, Biggerstaff BJ, Strauss D, Kessler D, Vassallo R, Reik R, Rossmann S, Destree M, Nguyen KA, Sayers M, Lough C, Bougie DW, Ritter M, Latoni G, Weales B, Sime S, Gorlin J, Brown NE, Gould CV, Berney K, Benoit TJ, Miller MJ, Freeman D, Kartik D, Fry AM, Azziz-Baumgartner E, Hall AJ, MacNeil A, Gundlapalli AV, Basavaraju SV, Gerber SI, Patton ME, Custer B, Williamson P, Simmons G, Thornburg NJ, Kleinman S, Stramer SL, Opsomer J, Busch MP. Estimated US Infection- and Vaccine-Induced SARS-CoV-2 Seroprevalence Based on Blood Donations, July 2020-May 2021. JAMA. 2021.

USA, Nebraska	Centers for Disease Control and Prevention (CDC). CDC COVID Data Tracker Commercial Laboratory Seroprevalence Survey Data. Atlanta, United States of America: Centers for Disease Control and Prevention (CDC).
USA, Nebraska	Jones JM, Stone M, Sulaeman H, Fink RV, Dave H, Levy ME, Di Germanio C, Green V, Notari E, Saa P, Biggerstaff BJ, Strauss D, Kessler D, Vassallo R, Reik R, Rossmann S, Destree M, Nguyen KA, Sayers M, Lough C, Bougie DW, Ritter M, Latoni G, Weales B, Sime S, Gorlin J, Brown NE, Gould CV, Berney K, Benoit TJ, Miller MJ, Freeman D, Kartik D, Fry AM, Azziz-Baumgartner E, Hall AJ, MacNeil A, Gundlapalli AV, Basavaraju SV, Gerber SI, Patton ME, Custer B, Williamson P, Simmons G, Thornburg NJ, Kleinman S, Stramer SL, Opsomer J, Busch MP. Estimated US Infection- and Vaccine-Induced SARS-CoV-2 Seroprevalence Based on Blood Donations, July 2020-May 2021. JAMA. 2021.
USA, Nevada	Centers for Disease Control and Prevention (CDC). CDC COVID Data Tracker Commercial Laboratory Seroprevalence Survey Data. Atlanta, United States of America: Centers for Disease Control and Prevention (CDC).
USA, Nevada	Jones JM, Stone M, Sulaeman H, Fink RV, Dave H, Levy ME, Di Germanio C, Green V, Notari E, Saa P, Biggerstaff BJ, Strauss D, Kessler D, Vassallo R, Reik R, Rossmann S, Destree M, Nguyen KA, Sayers M, Lough C, Bougie DW, Ritter M, Latoni G, Weales B, Sime S, Gorlin J, Brown NE, Gould CV, Berney K, Benoit TJ, Miller MJ, Freeman D, Kartik D, Fry AM, Azziz-Baumgartner E, Hall AJ, MacNeil A, Gundlapalli AV, Basavaraju SV, Gerber SI, Patton ME, Custer B, Williamson P, Simmons G, Thornburg NJ, Kleinman S, Stramer SL, Opsomer J, Busch MP. Estimated US Infection- and Vaccine-Induced SARS-CoV-2 Seroprevalence Based on Blood Donations, July 2020-May 2021. JAMA. 2021.
USA, New Hampshire	Centers for Disease Control and Prevention (CDC). CDC COVID Data Tracker Commercial Laboratory Seroprevalence Survey Data. Atlanta, United States of America: Centers for Disease Control and Prevention (CDC).
USA, New Hampshire	Jones JM, Stone M, Sulaeman H, Fink RV, Dave H, Levy ME, Di Germanio C, Green V, Notari E, Saa P, Biggerstaff BJ, Strauss D, Kessler D, Vassallo R, Reik R, Rossmann S, Destree M, Nguyen KA, Sayers M, Lough C, Bougie DW, Ritter M, Latoni G, Weales B, Sime S, Gorlin J, Brown NE, Gould CV, Berney K, Benoit TJ, Miller MJ, Freeman D, Kartik D, Fry AM, Azziz-Baumgartner E, Hall AJ, MacNeil A, Gundlapalli AV, Basavaraju SV, Gerber SI, Patton ME, Custer B, Williamson P, Simmons G, Thornburg NJ, Kleinman S, Stramer SL, Opsomer J, Busch MP. Estimated US Infection- and Vaccine-Induced SARS-CoV-2 Seroprevalence Based on Blood Donations, July 2020-May 2021. JAMA. 2021.
USA, New Jersey	Centers for Disease Control and Prevention (CDC). CDC COVID Data Tracker Commercial Laboratory Seroprevalence Survey Data. Atlanta, United States of America: Centers for Disease Control and Prevention (CDC).
USA, New Mexico	Centers for Disease Control and Prevention (CDC). CDC COVID Data Tracker Commercial Laboratory Seroprevalence Survey Data. Atlanta, United States of America: Centers for Disease Control and Prevention (CDC).
USA, New York	Rosenberg ES, Tesoriero JM, Rosenthal EM, Chung R, Barranco MA, Styer LM, Parker MM, John Leung SY, Morne JE, Greene D, Holtgrave DR, Hoefer D, Kumar J, Udo T, Hutton B, Zucker HA. Cumulative incidence and diagnosis of SARS-CoV-2 infection in New York. Ann Epidemiol. 2020; 48: 23-29e4.
USA, New York	Centers for Disease Control and Prevention (CDC). CDC COVID Data Tracker Commercial Laboratory Seroprevalence Survey Data. Atlanta, United States of America: Centers for Disease Control and Prevention (CDC).
USA, North Carolina	Centers for Disease Control and Prevention (CDC). CDC COVID Data Tracker Commercial Laboratory Seroprevalence Survey Data. Atlanta, United States of America: Centers for Disease Control and Prevention (CDC).

USA, North Carolina	Jones JM, Stone M, Sulaeman H, Fink RV, Dave H, Levy ME, Di Germanio C, Green V, Notari E, Saa P, Biggerstaff BJ, Strauss D, Kessler D, Vassallo R, Reik R, Rossmann S, Destree M, Nguyen KA, Sayers M, Lough C, Bougie DW, Ritter M, Latoni G, Weales B, Sime S, Gorlin J, Brown NE, Gould CV, Berney K, Benoit TJ, Miller MJ, Freeman D, Kartik D, Fry AM, Azziz-Baumgartner E, Hall AJ, MacNeil A, Gundlapalli AV, Basavaraju SV, Gerber SI, Patton ME, Custer B, Williamson P, Simmons G, Thornburg NJ, Kleinman S, Stramer SL, Opsomer J, Busch MP. Estimated US Infection- and Vaccine-Induced SARS-CoV-2 Seroprevalence Based on Blood Donations, July 2020-May 2021. JAMA. 2021.
USA, North Dakota	Jones JM, Stone M, Sulaeman H, Fink RV, Dave H, Levy ME, Di Germanio C, Green V, Notari E, Saa P, Biggerstaff BJ, Strauss D, Kessler D, Vassallo R, Reik R, Rossmann S, Destree M, Nguyen KA, Sayers M, Lough C, Bougie DW, Ritter M, Latoni G, Weales B, Sime S, Gorlin J, Brown NE, Gould CV, Berney K, Benoit TJ, Miller MJ, Freeman D, Kartik D, Fry AM, Azziz-Baumgartner E, Hall AJ, MacNeil A, Gundlapalli AV, Basavaraju SV, Gerber SI, Patton ME, Custer B, Williamson P, Simmons G, Thornburg NJ, Kleinman S, Stramer SL, Opsomer J, Busch MP. Estimated US Infection- and Vaccine-Induced SARS-CoV-2 Seroprevalence Based on Blood Donations, July 2020-May 2021. JAMA. 2021.
USA, Ohio	Centers for Disease Control and Prevention (CDC). CDC COVID Data Tracker Commercial Laboratory Seroprevalence Survey Data. Atlanta, United States of America: Centers for Disease Control and Prevention (CDC).
USA, Oklahoma	Centers for Disease Control and Prevention (CDC). CDC COVID Data Tracker Commercial Laboratory Seroprevalence Survey Data. Atlanta, United States of America: Centers for Disease Control and Prevention (CDC).
USA, Oklahoma	Jones JM, Stone M, Sulaeman H, Fink RV, Dave H, Levy ME, Di Germanio C, Green V, Notari E, Saa P, Biggerstaff BJ, Strauss D, Kessler D, Vassallo R, Reik R, Rossmann S, Destree M, Nguyen KA, Sayers M, Lough C, Bougie DW, Ritter M, Latoni G, Weales B, Sime S, Gorlin J, Brown NE, Gould CV, Berney K, Benoit TJ, Miller MJ, Freeman D, Kartik D, Fry AM, Azziz-Baumgartner E, Hall AJ, MacNeil A, Gundlapalli AV, Basavaraju SV, Gerber SI, Patton ME, Custer B, Williamson P, Simmons G, Thornburg NJ, Kleinman S, Stramer SL, Opsomer J, Busch MP. Estimated US Infection- and Vaccine-Induced SARS-CoV-2 Seroprevalence Based on Blood Donations, July 2020-May 2021. JAMA. 2021.
USA, Oregon	Centers for Disease Control and Prevention (CDC). CDC COVID Data Tracker Commercial Laboratory Seroprevalence Survey Data. Atlanta, United States of America: Centers for Disease Control and Prevention (CDC).
USA, Pennsylvania	Centers for Disease Control and Prevention (CDC). CDC COVID Data Tracker Commercial Laboratory Seroprevalence Survey Data. Atlanta, United States of America: Centers for Disease Control and Prevention (CDC).
USA, Rhode Island	Centers for Disease Control and Prevention (CDC). CDC COVID Data Tracker Commercial Laboratory Seroprevalence Survey Data. Atlanta, United States of America: Centers for Disease Control and Prevention (CDC).
USA, Rhode Island	Jones JM, Stone M, Sulaeman H, Fink RV, Dave H, Levy ME, Di Germanio C, Green V, Notari E, Saa P, Biggerstaff BJ, Strauss D, Kessler D, Vassallo R, Reik R, Rossmann S, Destree M, Nguyen KA, Sayers M, Lough C, Bougie DW, Ritter M, Latoni G, Weales B, Sime S, Gorlin J, Brown NE, Gould CV, Berney K, Benoit TJ, Miller MJ, Freeman D, Kartik D, Fry AM, Azziz-Baumgartner E, Hall AJ, MacNeil A, Gundlapalli AV, Basavaraju SV, Gerber SI, Patton ME, Custer B, Williamson P, Simmons G, Thornburg NJ, Kleinman S, Stramer SL, Opsomer J, Busch MP. Estimated US Infection- and Vaccine-Induced SARS-CoV-2 Seroprevalence Based on Blood Donations, July 2020-May 2021. JAMA. 2021.
USA, South Carolina	Centers for Disease Control and Prevention (CDC). CDC COVID Data Tracker Commercial Laboratory Seroprevalence Survey Data. Atlanta, United States of America: Centers for Disease Control and Prevention (CDC).

USA, South Carolina	Jones JM, Stone M, Sulaeman H, Fink RV, Dave H, Levy ME, Di Germanio C, Green V, Notari E, Saa P, Biggerstaff BJ, Strauss D, Kessler D, Vassallo R, Reik R, Rossmann S, Destree M, Nguyen KA, Sayers M, Lough C, Bougie DW, Ritter M, Latoni G, Weales B, Sime S, Gorlin J, Brown NE, Gould CV, Berney K, Benoit TJ, Miller MJ, Freeman D, Kartik D, Fry AM, Azziz-Baumgartner E, Hall AJ, MacNeil A, Gundlapalli AV, Basavaraju SV, Gerber SI, Patton ME, Custer B, Williamson P, Simmons G, Thornburg NJ, Kleinman S, Stramer SL, Opsomer J, Busch MP. Estimated US Infection- and Vaccine-Induced SARS-CoV-2 Seroprevalence Based on Blood Donations, July 2020-May 2021. JAMA. 2021.
USA, South Dakota	Jones JM, Stone M, Sulaeman H, Fink RV, Dave H, Levy ME, Di Germanio C, Green V, Notari E, Saa P, Biggerstaff BJ, Strauss D, Kessler D, Vassallo R, Reik R, Rossmann S, Destree M, Nguyen KA, Sayers M, Lough C, Bougie DW, Ritter M, Latoni G, Weales B, Sime S, Gorlin J, Brown NE, Gould CV, Berney K, Benoit TJ, Miller MJ, Freeman D, Kartik D, Fry AM, Azziz-Baumgartner E, Hall AJ, MacNeil A, Gundlapalli AV, Basavaraju SV, Gerber SI, Patton ME, Custer B, Williamson P, Simmons G, Thornburg NJ, Kleinman S, Stramer SL, Opsomer J, Busch MP. Estimated US Infection- and Vaccine-Induced SARS-CoV-2 Seroprevalence Based on Blood Donations, July 2020-May 2021. JAMA. 2021.
USA, Tennessee	Centers for Disease Control and Prevention (CDC). CDC COVID Data Tracker Commercial Laboratory Seroprevalence Survey Data. Atlanta, United States of America: Centers for Disease Control and Prevention (CDC).
USA, Texas	Centers for Disease Control and Prevention (CDC). CDC COVID Data Tracker Commercial Laboratory Seroprevalence Survey Data. Atlanta, United States of America: Centers for Disease Control and Prevention (CDC).
USA, Utah	Havers FP, Reed C, Lim T, Montgomery JM, Klena JD, Hall AJ, Fry AM, Cannon DL, Chiang CF, Gibbons A, Krapiunaya I, Morales-Betoulle M, Roguski K, Rasheed MAU, Freeman B, Lester S, Mills L, Carroll DS, Owen SM, Johnson JA, Semenova V, Blackmore C, Blog D, Chai SJ, Dunn A, Hand J, Jain S, Lindquist S, Lynfield R, Pritchard S, Sokol T, Sosa L, Turabelidze G, Watkins SM, Wiesman J, Williams RW, Yendell S, Schiffer J, Thornburg NJ. Seroprevalence of Antibodies to SARS-CoV-2 in 10 Sites in the United States, March 23-May 12, 2020. JAMA Intern Med. 2020.
USA, Utah	Centers for Disease Control and Prevention (CDC). CDC COVID Data Tracker Commercial Laboratory Seroprevalence Survey Data. Atlanta, United States of America: Centers for Disease Control and Prevention (CDC).
USA, Utah	Jones JM, Stone M, Sulaeman H, Fink RV, Dave H, Levy ME, Di Germanio C, Green V, Notari E, Saa P, Biggerstaff BJ, Strauss D, Kessler D, Vassallo R, Reik R, Rossmann S, Destree M, Nguyen KA, Sayers M, Lough C, Bougie DW, Ritter M, Latoni G, Weales B, Sime S, Gorlin J, Brown NE, Gould CV, Berney K, Benoit TJ, Miller MJ, Freeman D, Kartik D, Fry AM, Azziz-Baumgartner E, Hall AJ, MacNeil A, Gundlapalli AV, Basavaraju SV, Gerber SI, Patton ME, Custer B, Williamson P, Simmons G, Thornburg NJ, Kleinman S, Stramer SL, Opsomer J, Busch MP. Estimated US Infection- and Vaccine-Induced SARS-CoV-2 Seroprevalence Based on Blood Donations, July 2020-May 2021. JAMA. 2021.
USA, Vermont	Centers for Disease Control and Prevention (CDC). CDC COVID Data Tracker Commercial Laboratory Seroprevalence Survey Data. Atlanta, United States of America: Centers for Disease Control and Prevention (CDC).
USA, Vermont	Jones JM, Stone M, Sulaeman H, Fink RV, Dave H, Levy ME, Di Germanio C, Green V, Notari E, Saa P, Biggerstaff BJ, Strauss D, Kessler D, Vassallo R, Reik R, Rossmann S, Destree M, Nguyen KA, Sayers M, Lough C, Bougie DW, Ritter M, Latoni G, Weales B, Sime S, Gorlin J, Brown NE, Gould CV, Berney K, Benoit TJ, Miller MJ, Freeman D, Kartik D, Fry AM, Azziz-Baumgartner E, Hall AJ, MacNeil A, Gundlapalli AV, Basavaraju SV, Gerber SI, Patton ME, Custer B, Williamson P, Simmons G, Thornburg NJ, Kleinman S, Stramer SL, Opsomer J, Busch MP. Estimated US Infection- and Vaccine-Induced SARS-CoV-2 Seroprevalence Based on Blood Donations, July 2020-May 2021. JAMA. 2021.
USA, Virginia	Centers for Disease Control and Prevention (CDC). CDC COVID Data Tracker Commercial Laboratory Seroprevalence Survey Data. Atlanta, United States of America: Centers for Disease Control and Prevention (CDC).

USA, Washington	Centers for Disease Control and Prevention (CDC). CDC COVID Data Tracker Commercial Laboratory Seroprevalence Survey Data. Atlanta, United States of America: Centers for Disease Control and Prevention (CDC).
USA, Washington, DC	Centers for Disease Control and Prevention (CDC). CDC COVID Data Tracker Commercial Laboratory Seroprevalence Survey Data. Atlanta, United States of America: Centers for Disease Control and Prevention (CDC).
USA, West Virginia	Centers for Disease Control and Prevention (CDC). CDC COVID Data Tracker Commercial Laboratory Seroprevalence Survey Data. Atlanta, United States of America: Centers for Disease Control and Prevention (CDC).
USA, Wisconsin	Centers for Disease Control and Prevention (CDC). CDC COVID Data Tracker Commercial Laboratory Seroprevalence Survey Data. Atlanta, United States of America: Centers for Disease Control and Prevention (CDC).
USA, Wyoming	Centers for Disease Control and Prevention (CDC). CDC COVID Data Tracker Commercial Laboratory Seroprevalence Survey Data. Atlanta, United States of America: Centers for Disease Control and Prevention (CDC).
USA, Wyoming	Jones JM, Stone M, Sulaeman H, Fink RV, Dave H, Levy ME, Di Germanio C, Green V, Notari E, Saa P, Biggerstaff BJ, Strauss D, Kessler D, Vassallo R, Reik R, Rossmann S, Destree M, Nguyen KA, Sayers M, Lough C, Bougie DW, Ritter M, Latoni G, Weales B, Sime S, Gorlin J, Brown NE, Gould CV, Berney K, Benoit TJ, Miller MJ, Freeman D, Kartik D, Fry AM, Azziz-Baumgartner E, Hall AJ, MacNeil A, Gundlapalli AV, Basavaraju SV, Gerber SI, Patton ME, Custer B, Williamson P, Simmons G, Thornburg NJ, Kleinman S, Stramer SL, Opsomer J, Busch MP. Estimated US Infection- and Vaccine-Induced SARS-CoV-2 Seroprevalence Based on Blood Donations, July 2020-May 2021. JAMA. 2021.
