# Supplementary material for: Variation in the COVID-19 infection–fatality ratio by age, time, and geography during the pre-vaccine era: a systematic analysis
Source: Lancet. 2022 Apr 16;399(10334):1469–88. doi: 10.1016/S0140-6736(21)02867-1 (PMC8871594; doi:10.1016/S0140-6736(21)02867-1)
Supplement: Supplementary appendix 3 [file mmc3.pdf]

THE LANCET

Supplementary appendix

This appendix formed part of the original submission and has been peer reviewed. We post it as supplied by the authors.

Supplement to: COVID-19 Forecasting Team. Variation in the COVID-19 infection–fatality ratio by age, time, and geography during the pre-vaccine era: a systematic analysis. *Lancet* 2022; published online Feb 24. https://doi.org/10.1016/S0140-6736(21)02867-1.

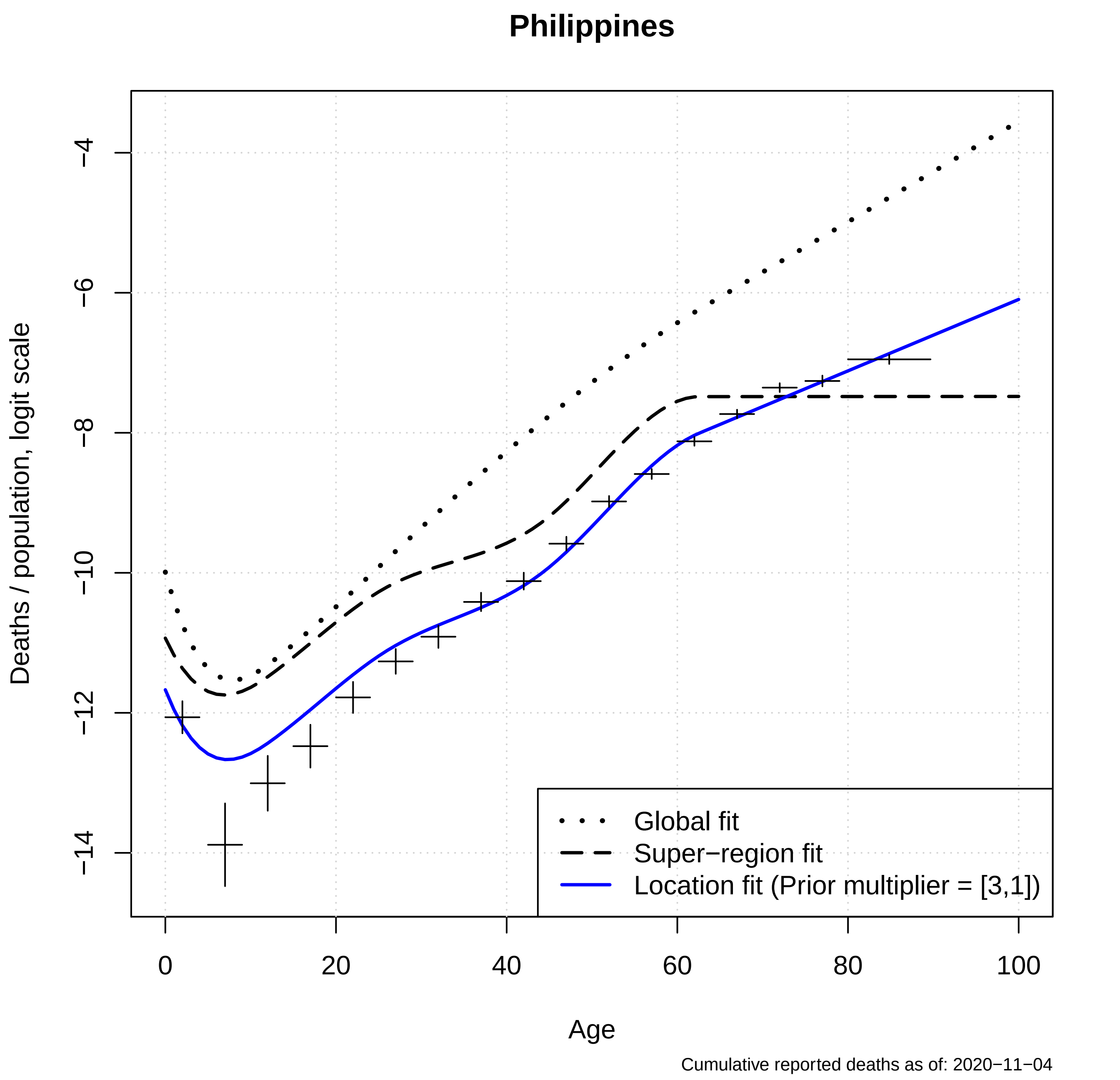

Philippines
Deaths / population, logit scale
−4
−6
−8
−10
−12
−14
0
20
40
60
80
100
Age
Global fit
Super−region fit
Location fit (Prior multiplier = [3,1])
Cumulative reported deaths as of: 2020−11−04

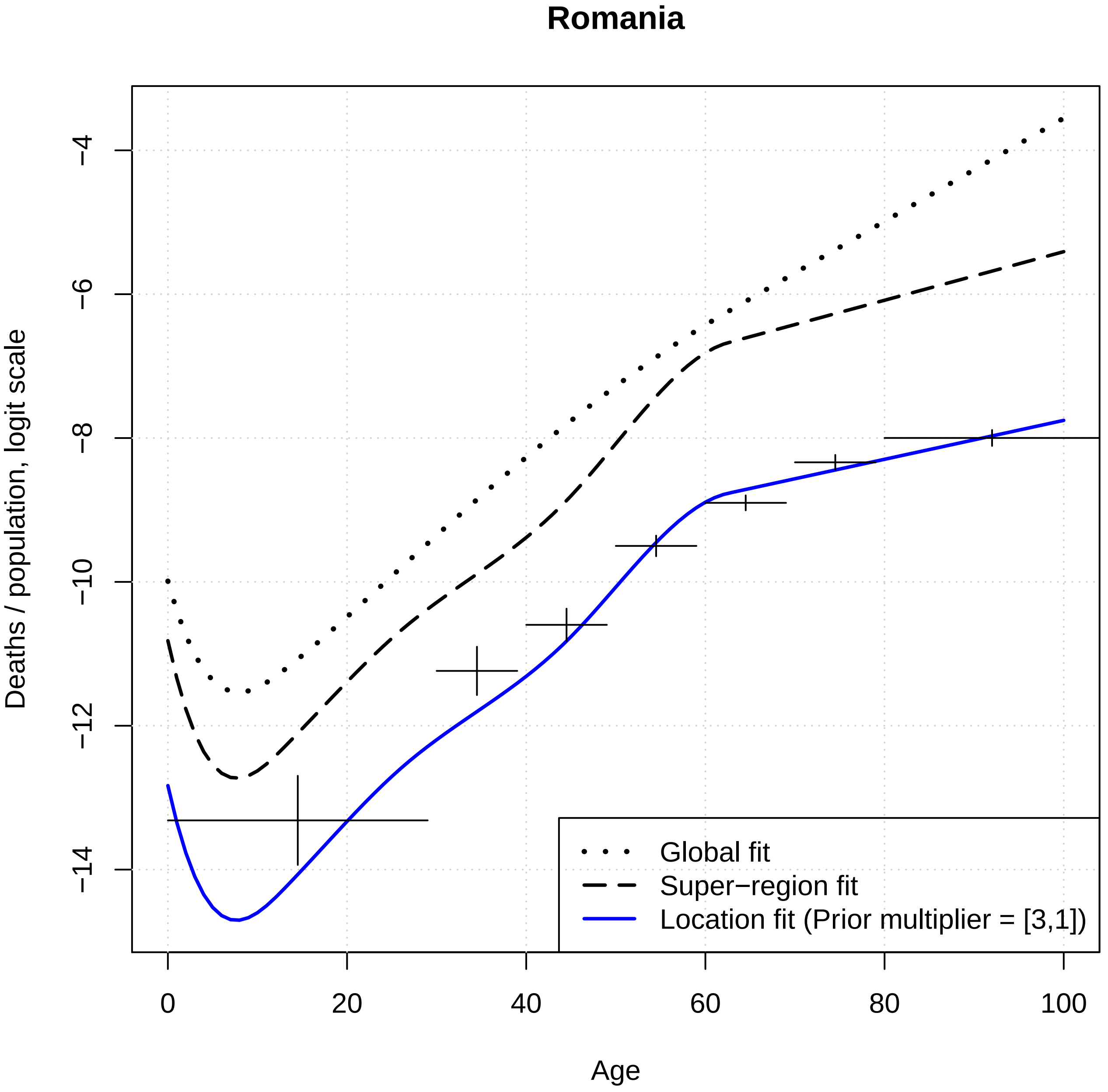

Cumulative reported deaths as of: 2020−06−10

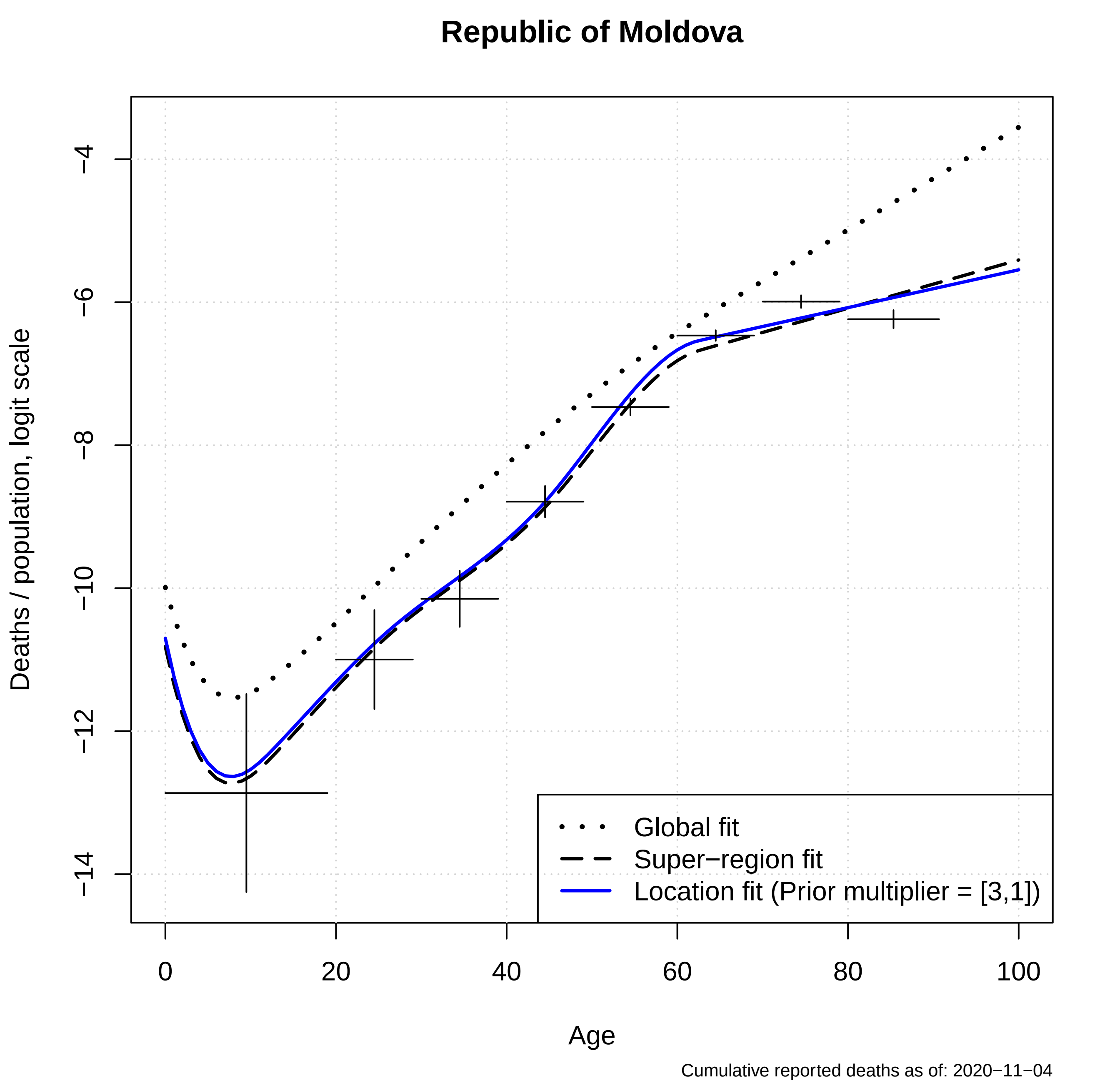

Republic of Moldova
Deaths / population, logit scale
−4
−6
−8
−10
−12
−14
0
20
40
60
80
100
Age
Global fit
Super−region fit
Location fit (Prior multiplier = [3,1])
Cumulative reported deaths as of: 2020−11−04

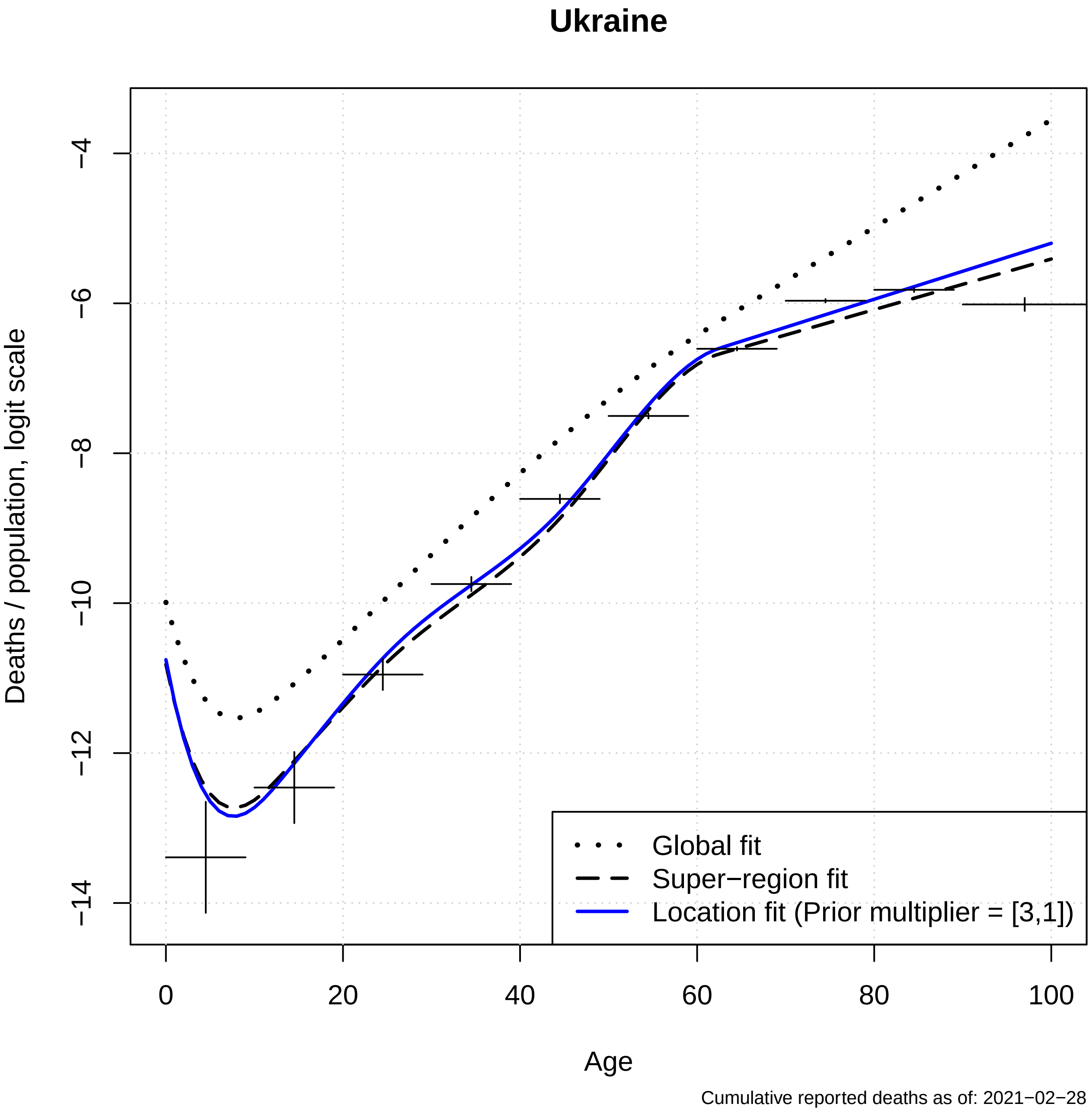

Ukraine
Deaths / population, logit scale
−4
−6
−8
−10
−12
−14
0
20
40
60
80
100
Age
Global fit
Super−region fit
Location fit (Prior multiplier = [3,1])
Cumulative reported deaths as of: 2021−02−28

Figure S3. Age patterns of COVID-19 mortality for select countries and territories in the high-income super-region

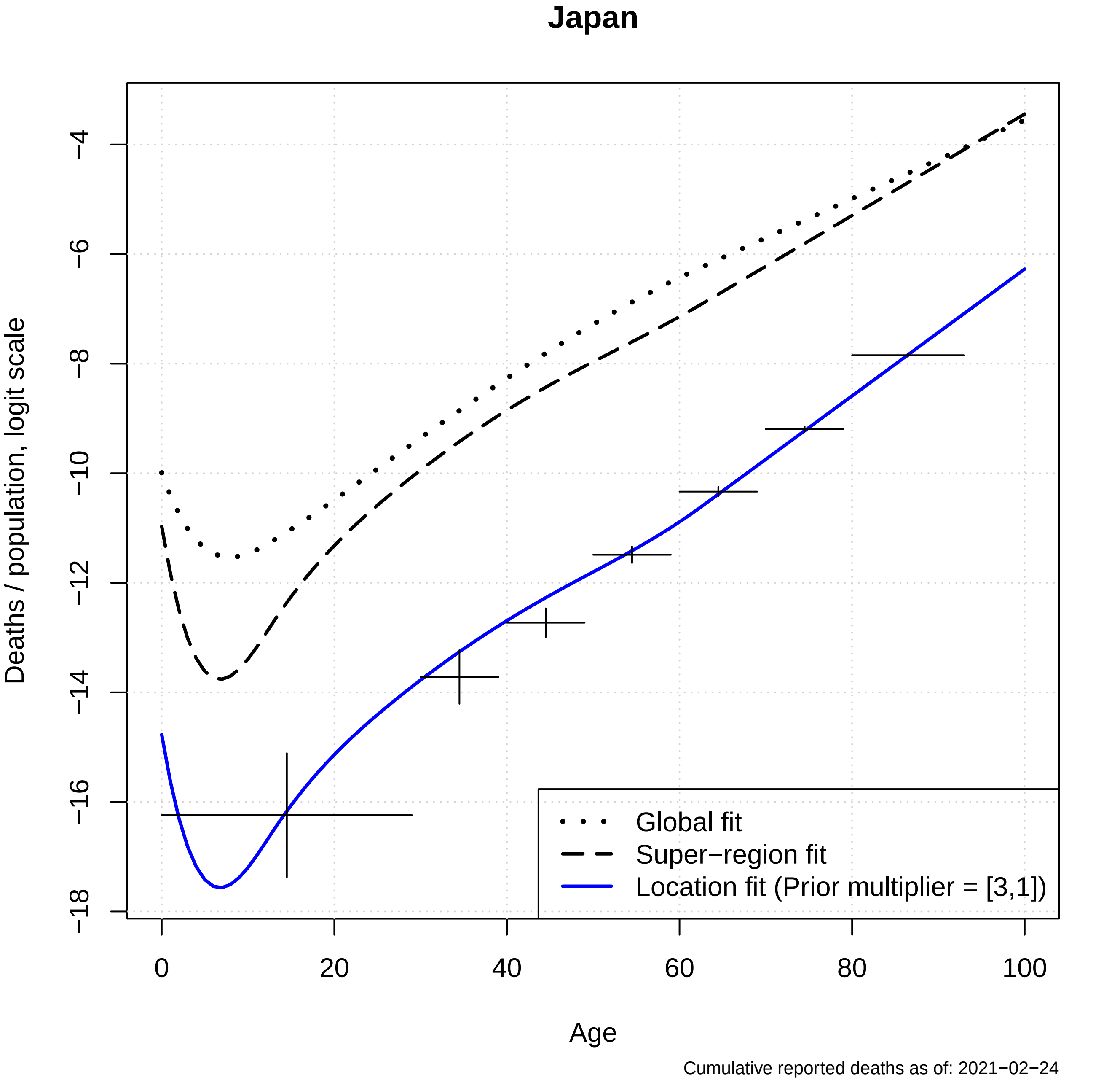

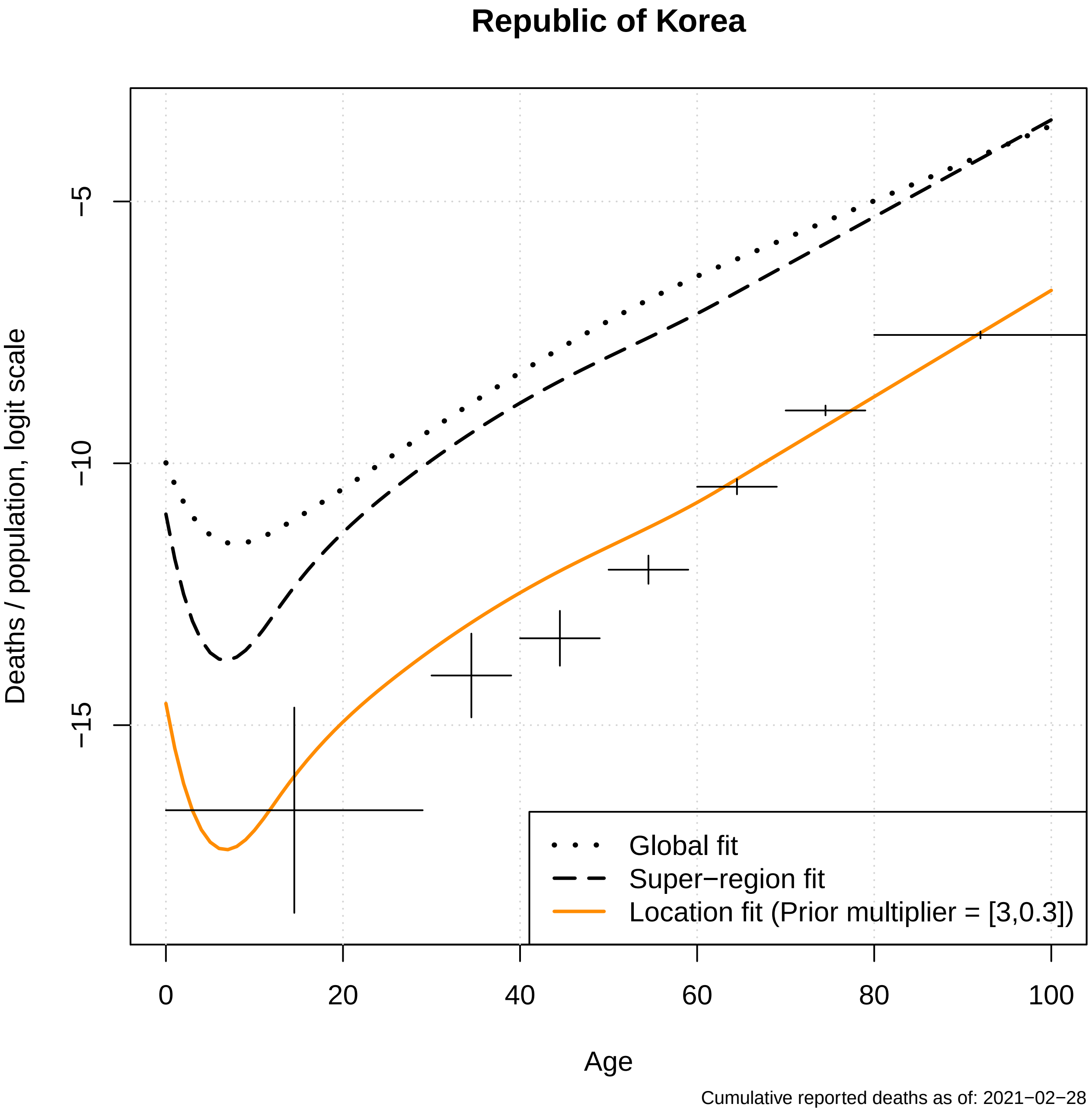
Republic of Korea
Deaths / population, logit scale
−5
−10
−15
0
20
40
60
80
100
Age
Global fit
Super−region fit
Location fit (Prior multiplier = [3,0.3])
Cumulative reported deaths as of: 2021−02−28

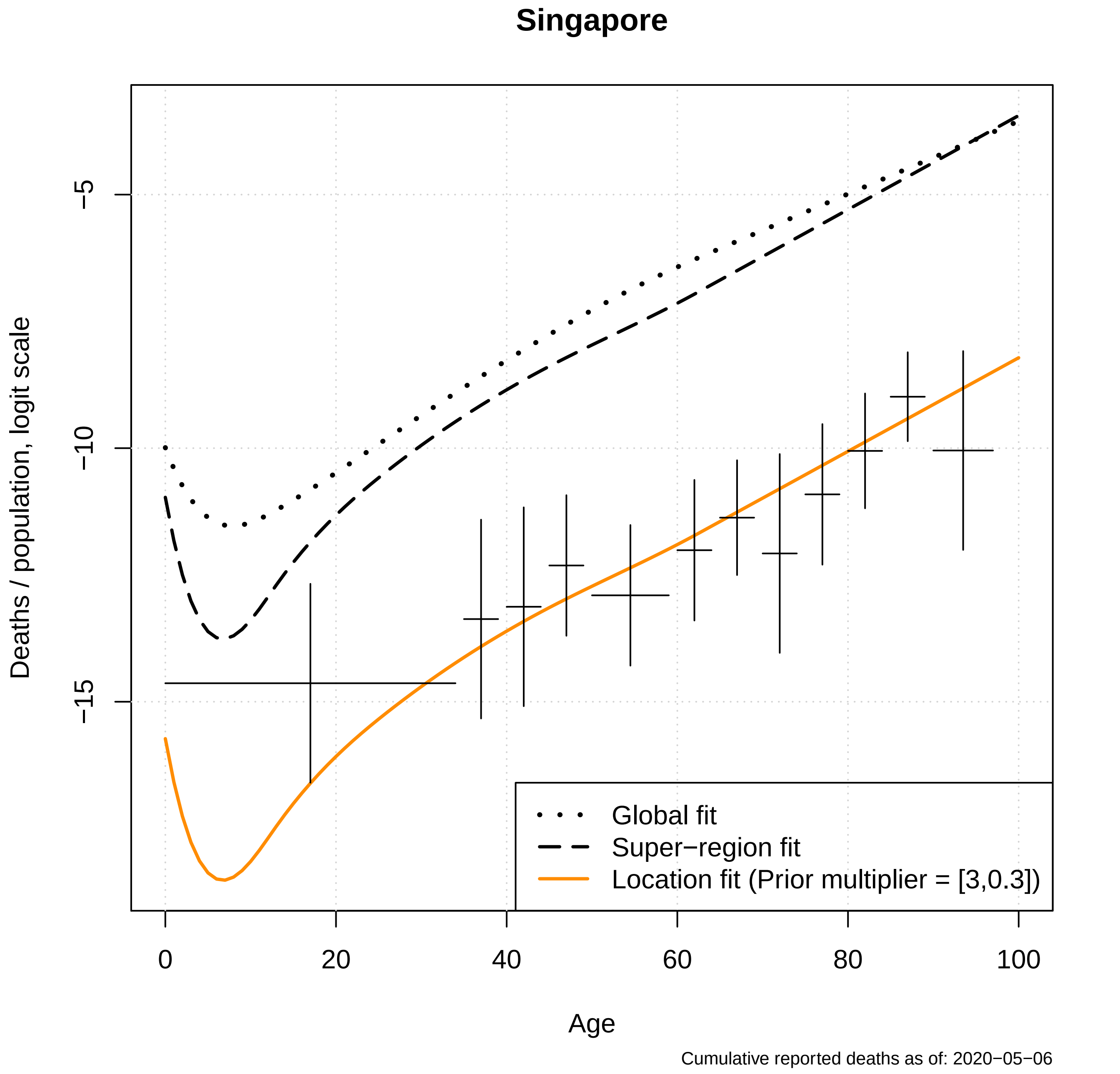
Singapore
Deaths / population, logit scale
−5
−10
−15
0
20
40
60
80
100
Age
Global fit
Super−region fit
Location fit (Prior multiplier = [3,0.3])
Cumulative reported deaths as of: 2020−05−06

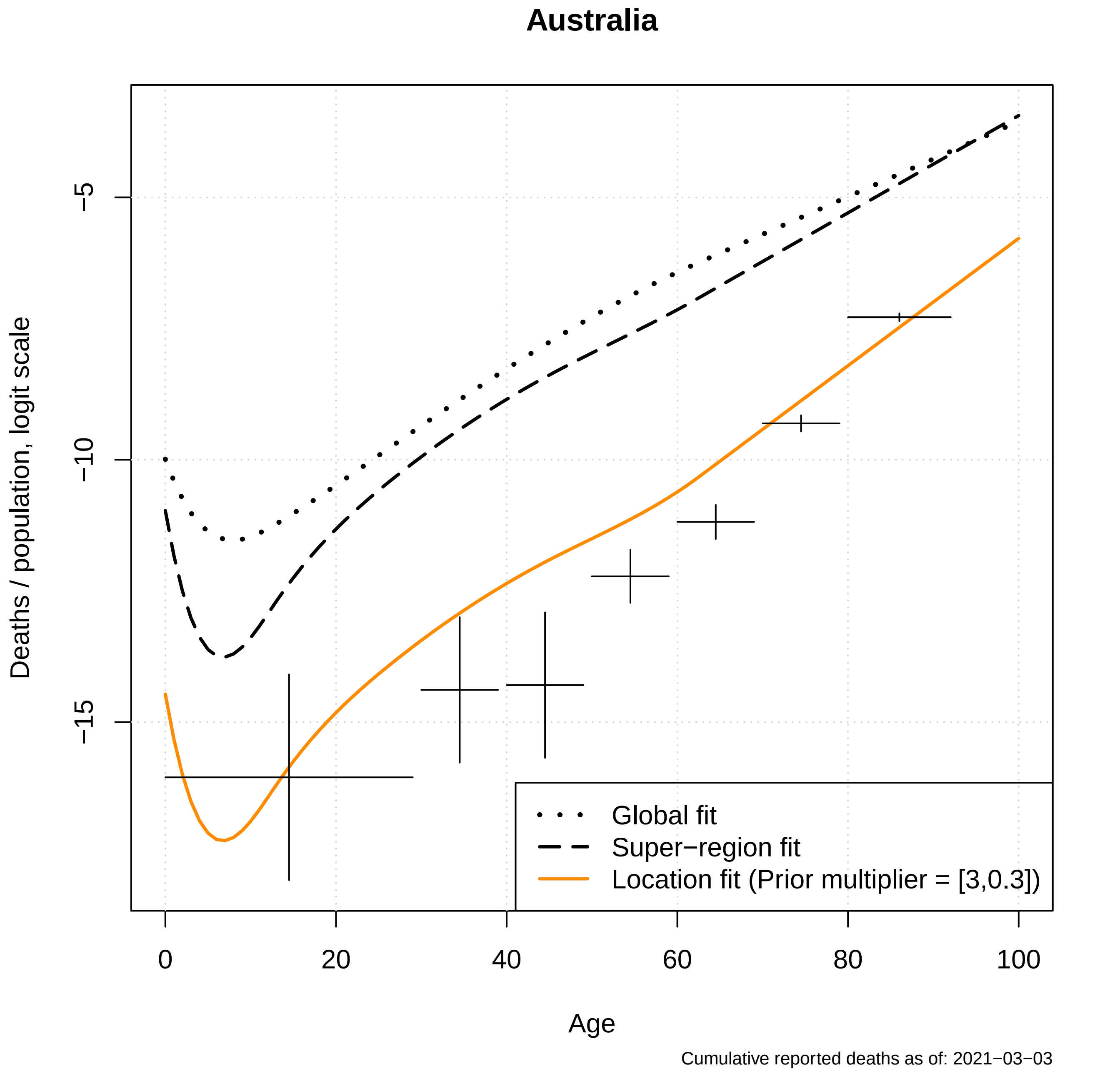
Australia
Deaths / population, logit scale
−5
−10
−15
0
20
40
60
80
100
Age
Global fit
Super−region fit
Location fit (Prior multiplier = [3,0.3])
Cumulative reported deaths as of: 2021−03−03

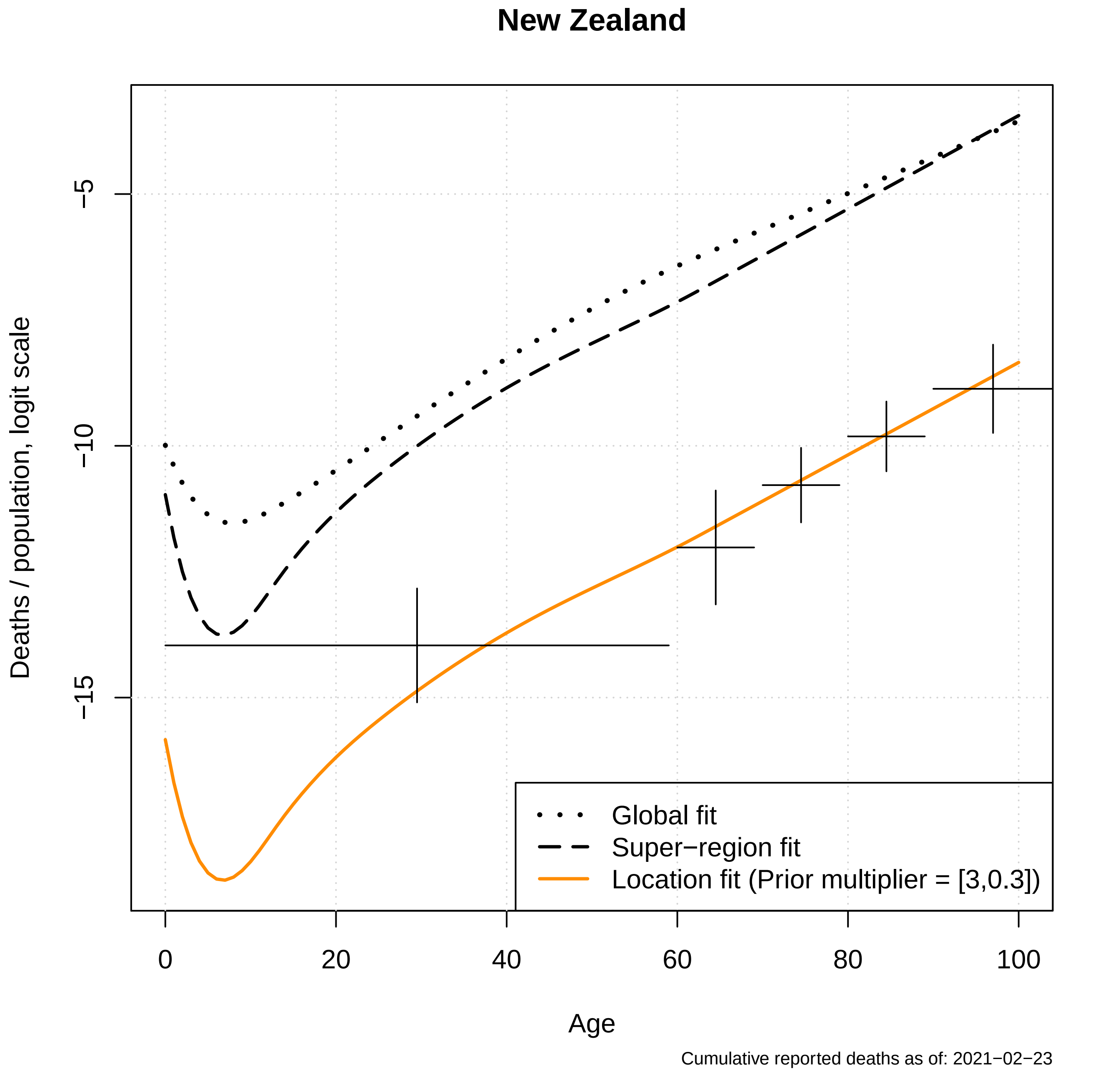

New Zealand
Deaths / population, logit scale
−5
−10
−15
0
20
40
60
80
100
Age
Global fit
Super−region fit
Location fit (Prior multiplier = [3,0.3])
Cumulative reported deaths as of: 2021−02−23

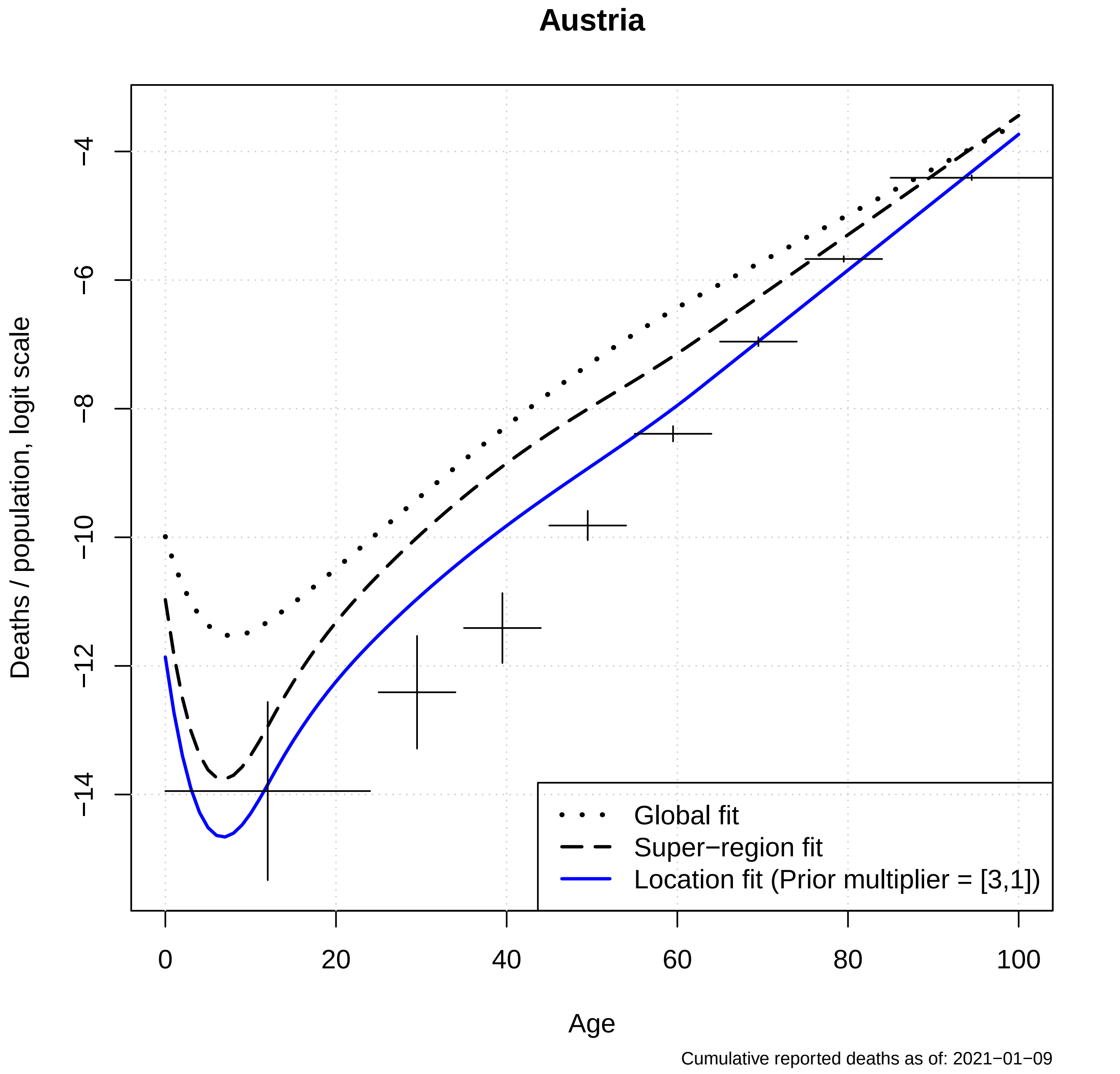

Austria
Deaths / population, logit scale
−4
−6
−8
−10
−12
−14
0
20
40
60
80
100
Age
Global fit
Super−region fit
Location fit (Prior multiplier = [3,1])
Cumulative reported deaths as of: 2021−01−09

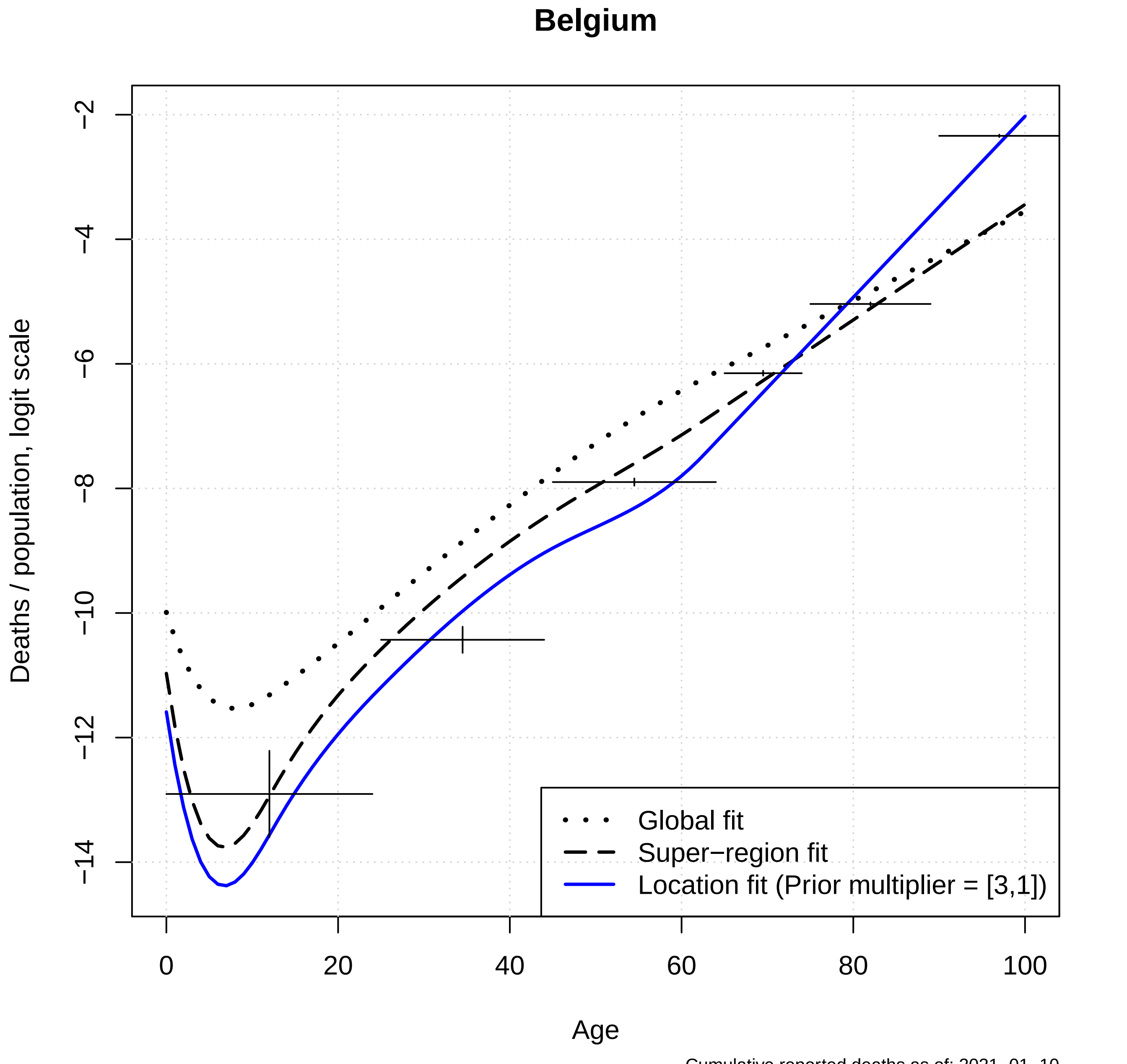

Cumulative reported deaths as of: 2021−01−10

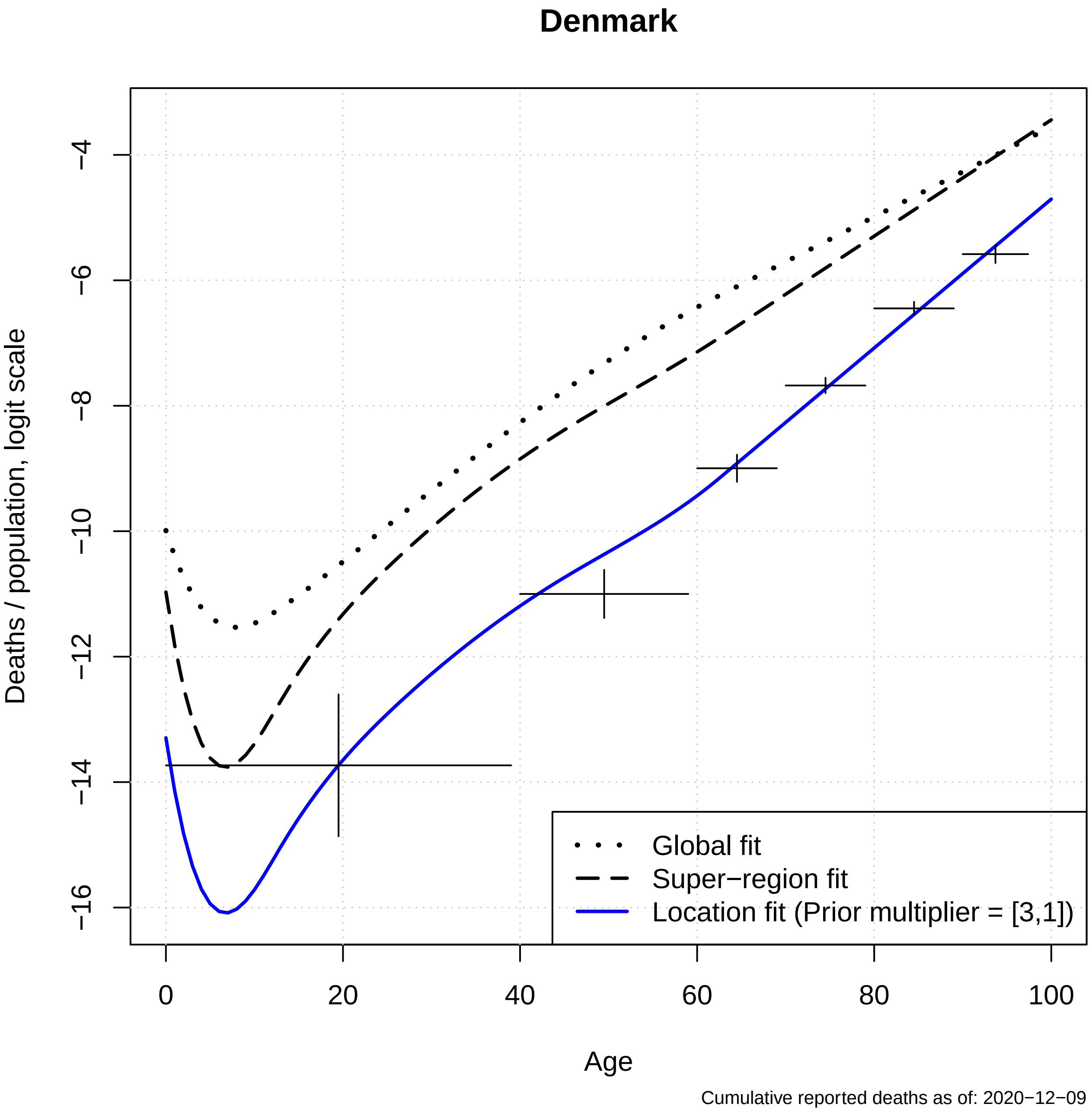

Denmark
Deaths / population, logit scale
−4
−6
−8
−10
−12
−14
−16
0
20
40
60
80
100
Age
Global fit
Super−region fit
Location fit (Prior multiplier = [3,1])
Cumulative reported deaths as of: 2020−12−09

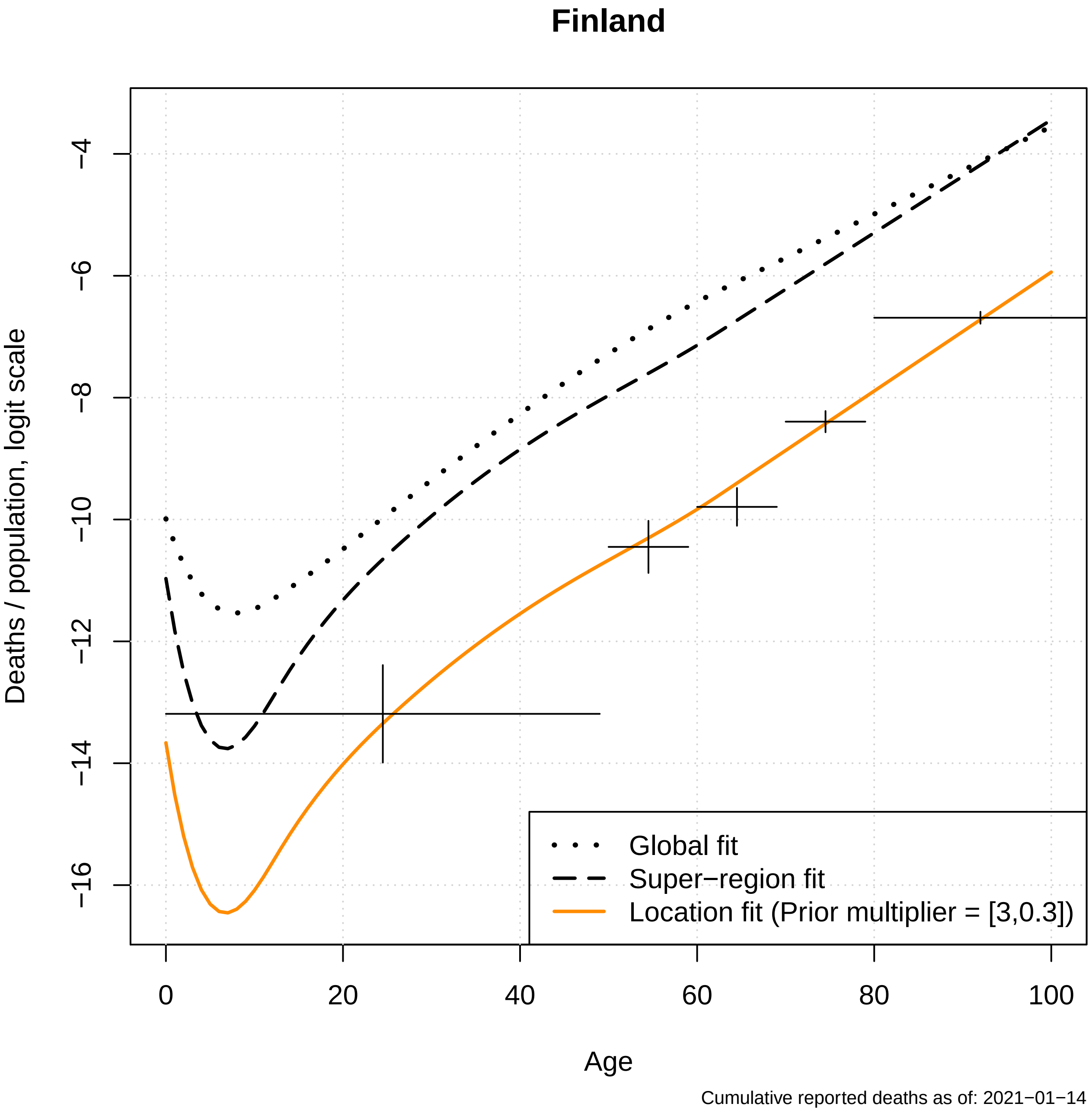
Finland
Deaths / population, logit scale
−4
−6
−8
−10
−12
−14
−16
0
20
40
60
80
100
Age
Global fit
Super−region fit
Location fit (Prior multiplier = [3,0.3])
Cumulative reported deaths as of: 2021−01−14

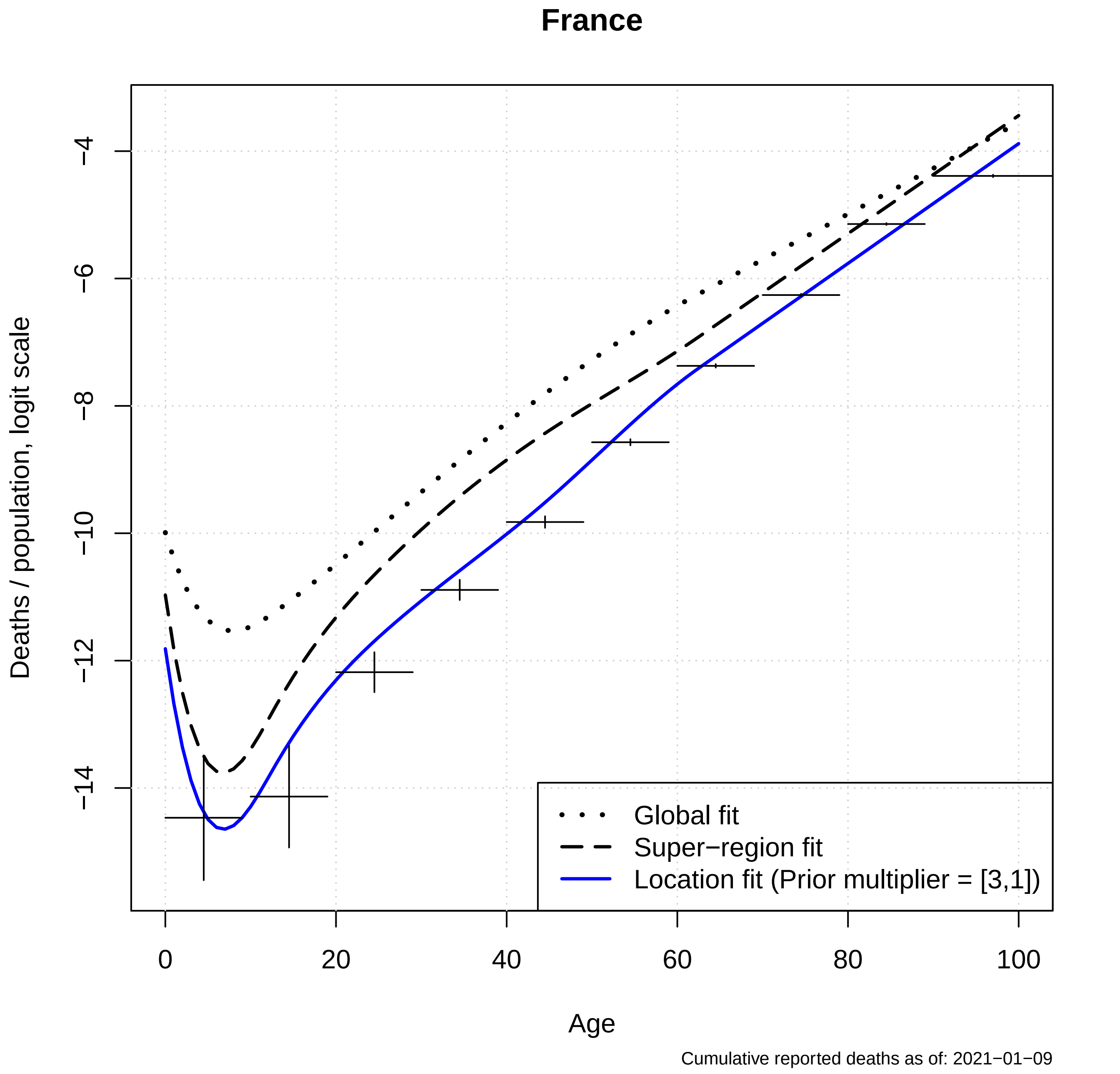
France
Deaths / population, logit scale
−4
−6
−8
−10
−12
−14
0
20
40
60
80
100
Age
Global fit
Super−region fit
Location fit (Prior multiplier = [3,1])
Cumulative reported deaths as of: 2021−01−09

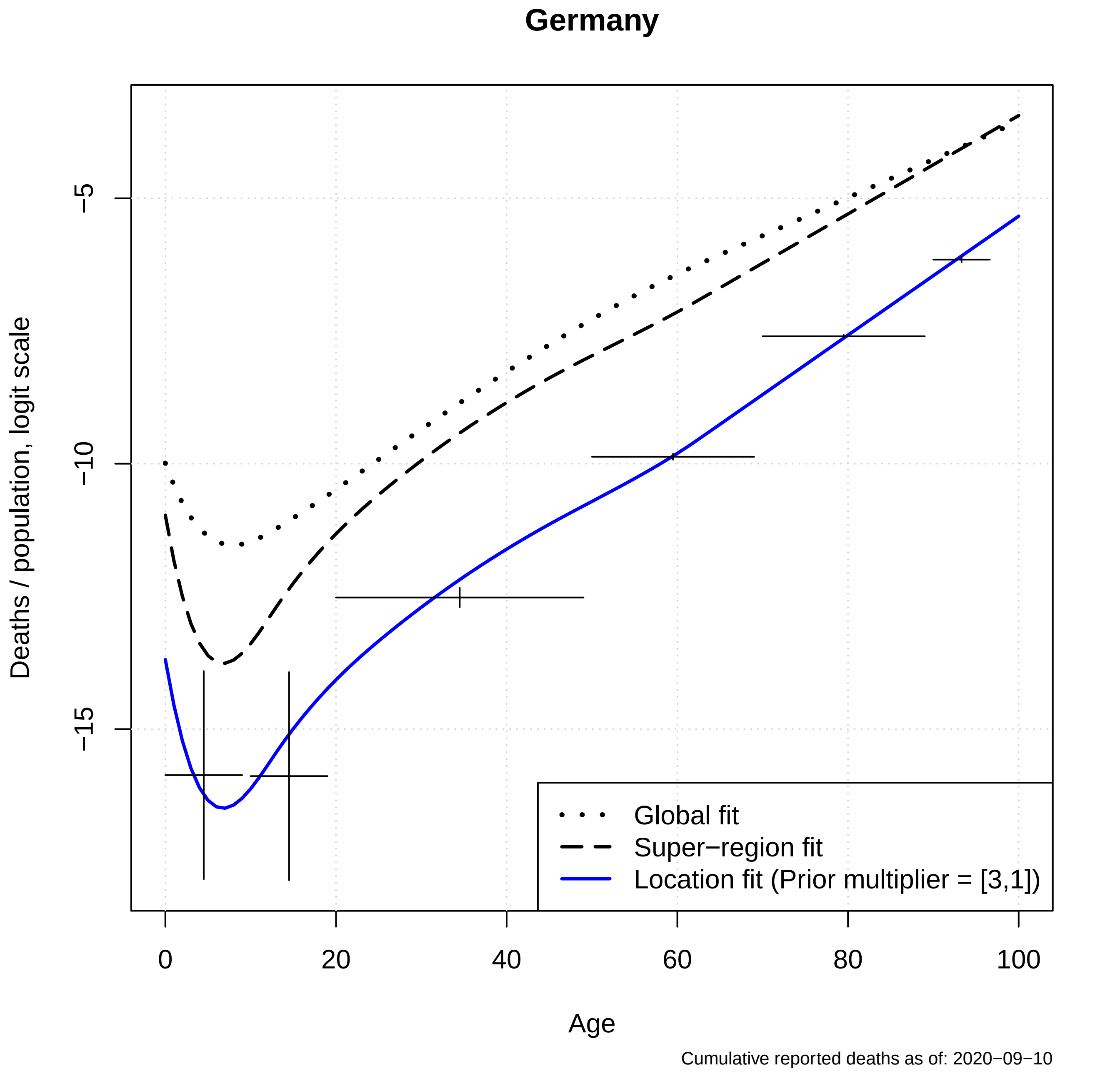

Germany
Deaths / population, logit scale
−5
−10
−15
0
20
40
60
80
100
Age
Global fit
Super−region fit
Location fit (Prior multiplier = [3,1])
Cumulative reported deaths as of: 2020−09−10

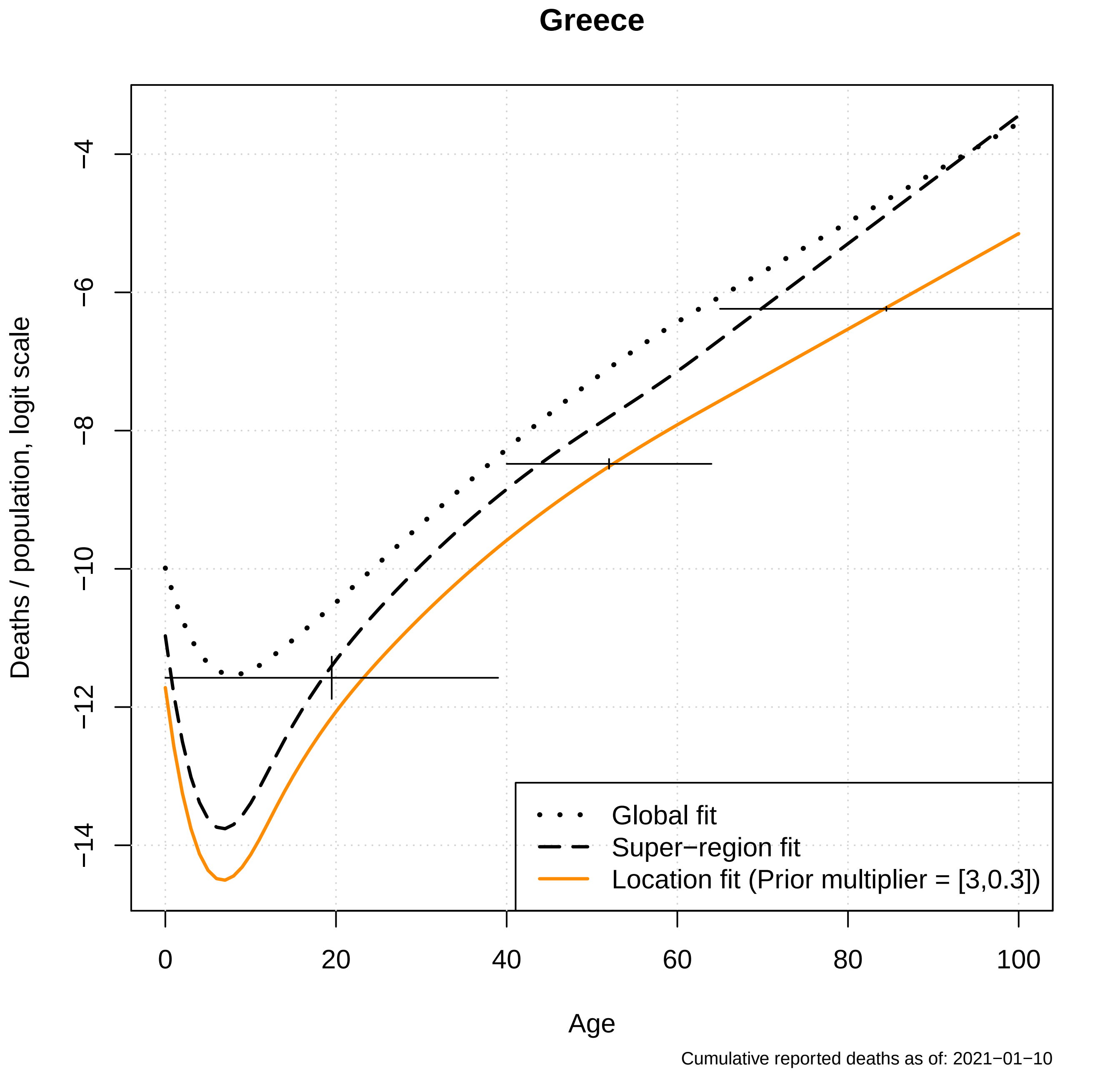
Greece
−4
−6
−8
−10
−12
−14
Deaths / population, logit scale
0
20
40
60
80
100
Age
Global fit
Super−region fit
Location fit (Prior multiplier = [3,0.3])
Cumulative reported deaths as of: 2021−01−10

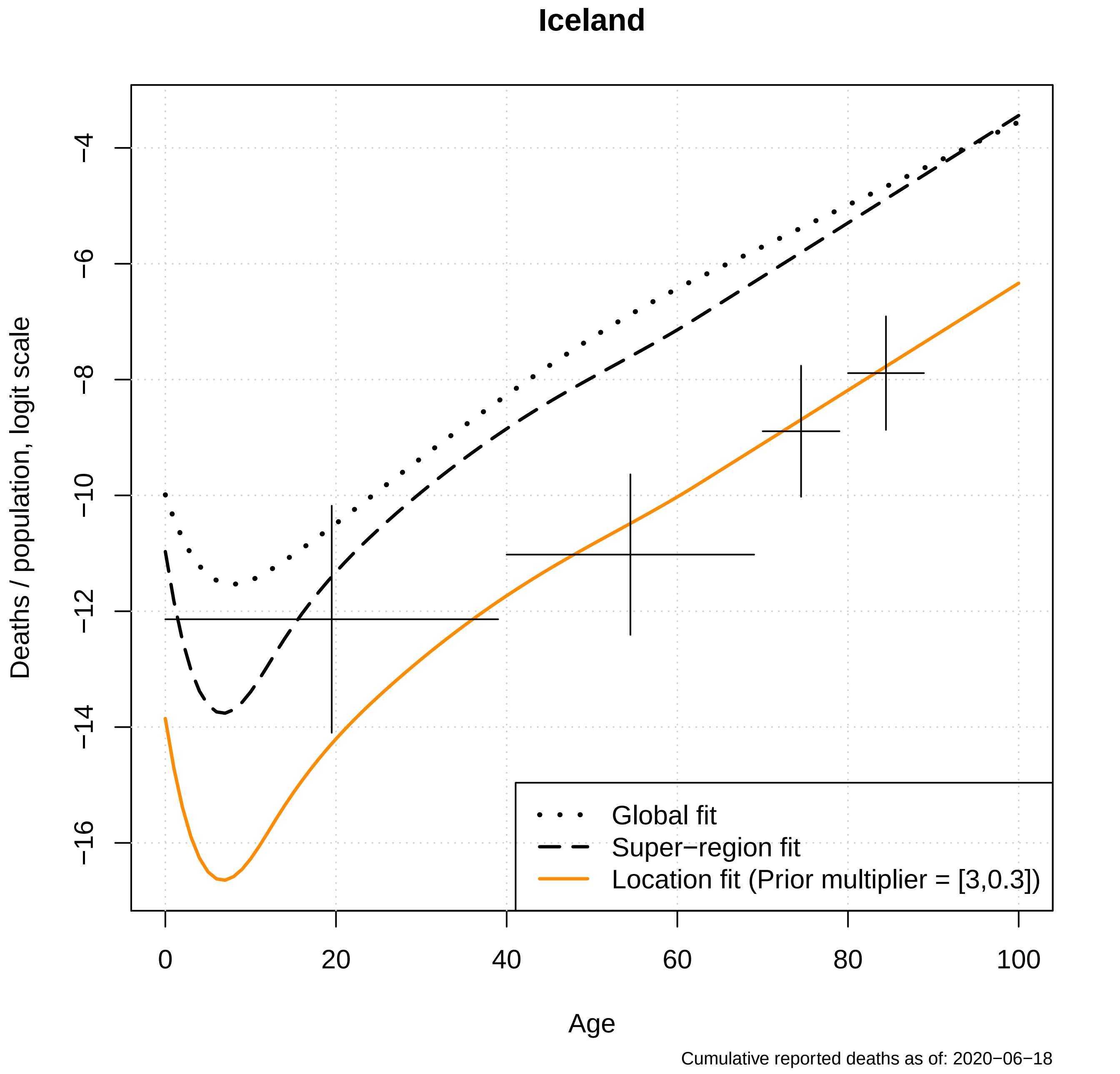
Iceland
Deaths / population, logit scale
−4
−6
−8
−10
−12
−14
−16
0
20
40
60
80
100
Age
Global fit
Super−region fit
Location fit (Prior multiplier = [3,0.3])
Cumulative reported deaths as of: 2020−06−18

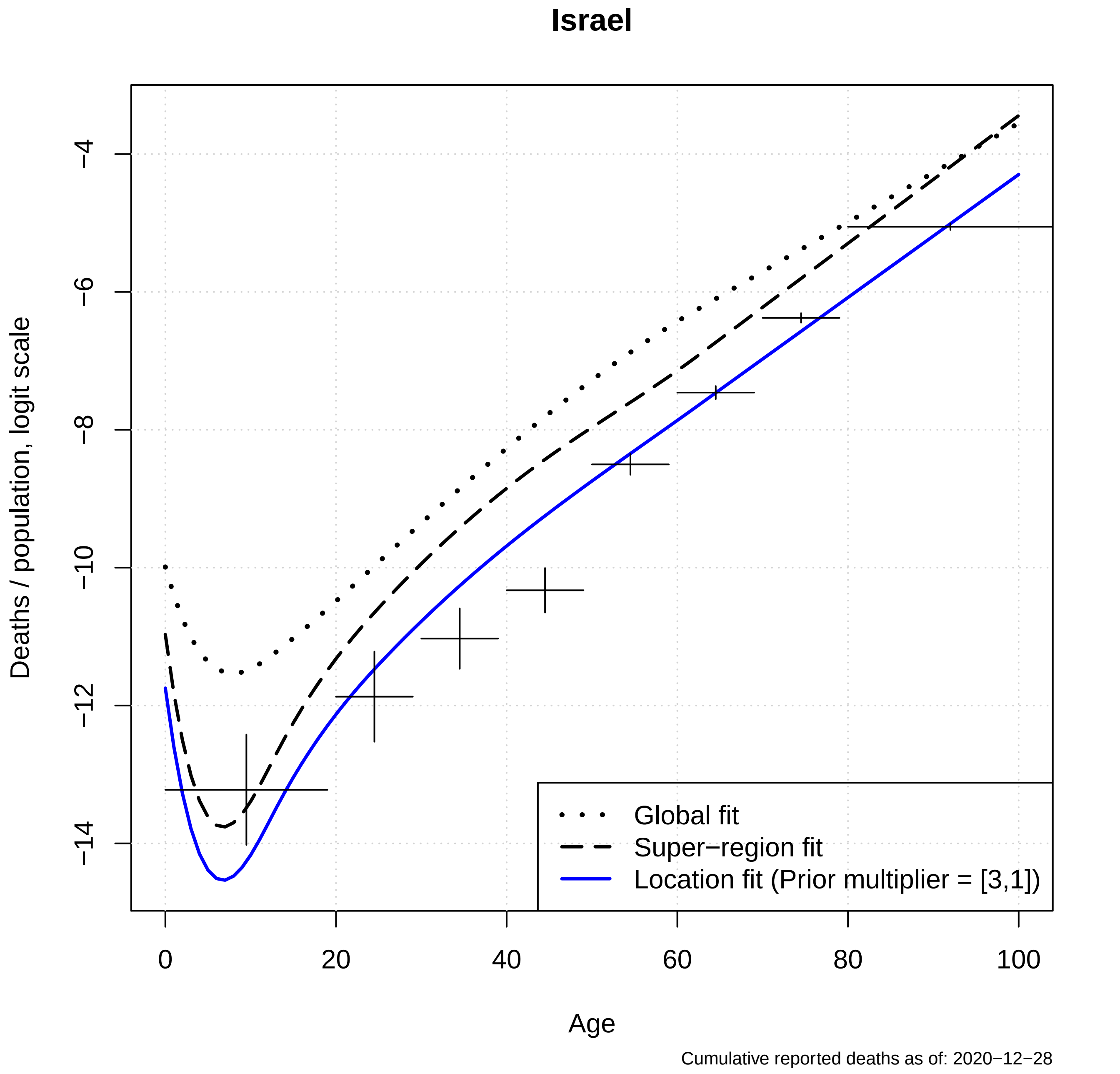
Israel
Deaths / population, logit scale
−4
−6
−8
−10
−12
−14
0
20
40
60
80
100
Age
Global fit
Super−region fit
Location fit (Prior multiplier = [3,1])
Cumulative reported deaths as of: 2020−12−28

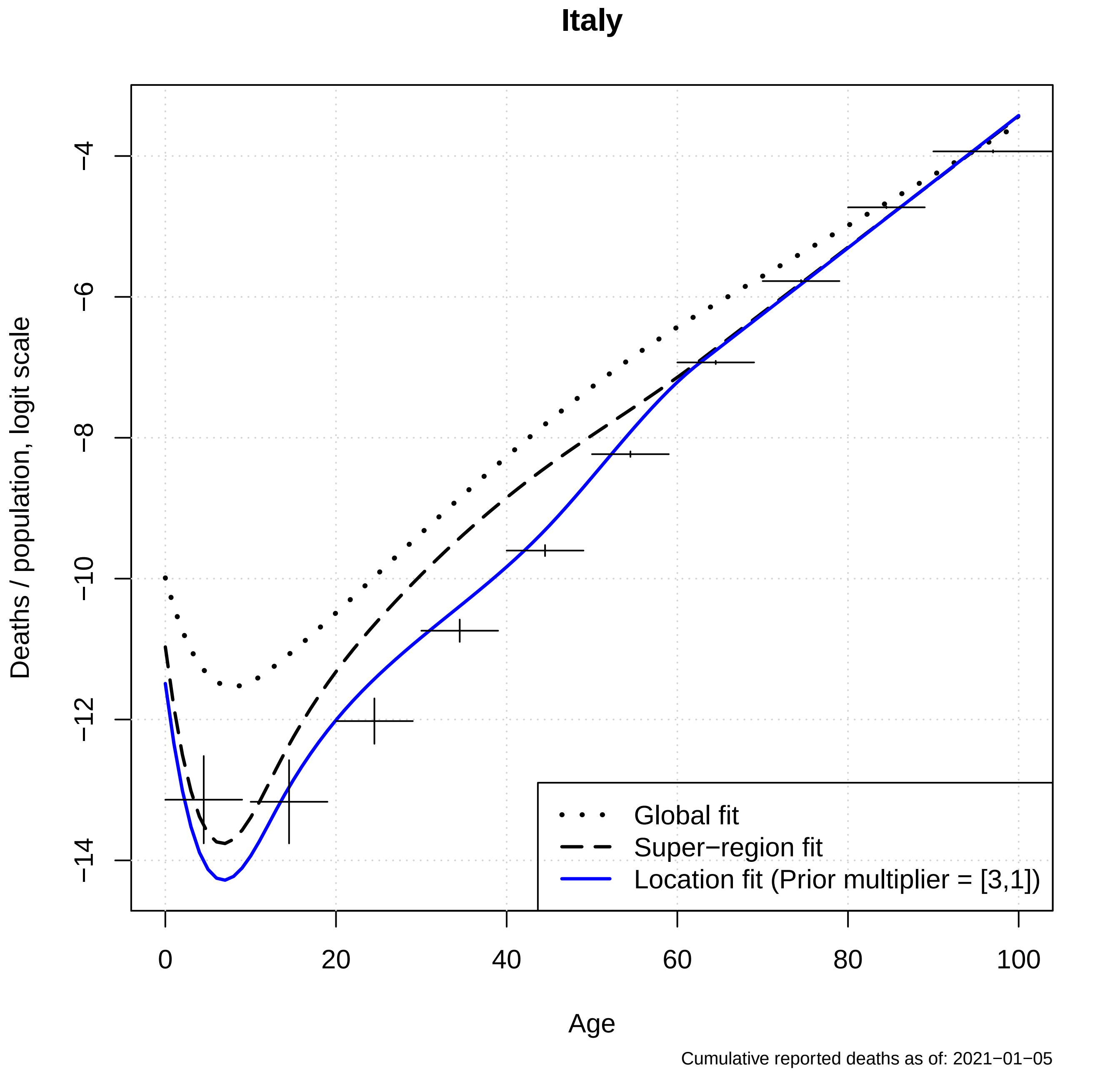
Italy
Deaths / population, logit scale
−4
−6
−8
−10
−12
−14
0
20
40
60
80
100
Age
Global fit
Super−region fit
Location fit (Prior multiplier = [3,1])
Cumulative reported deaths as of: 2021−01−05

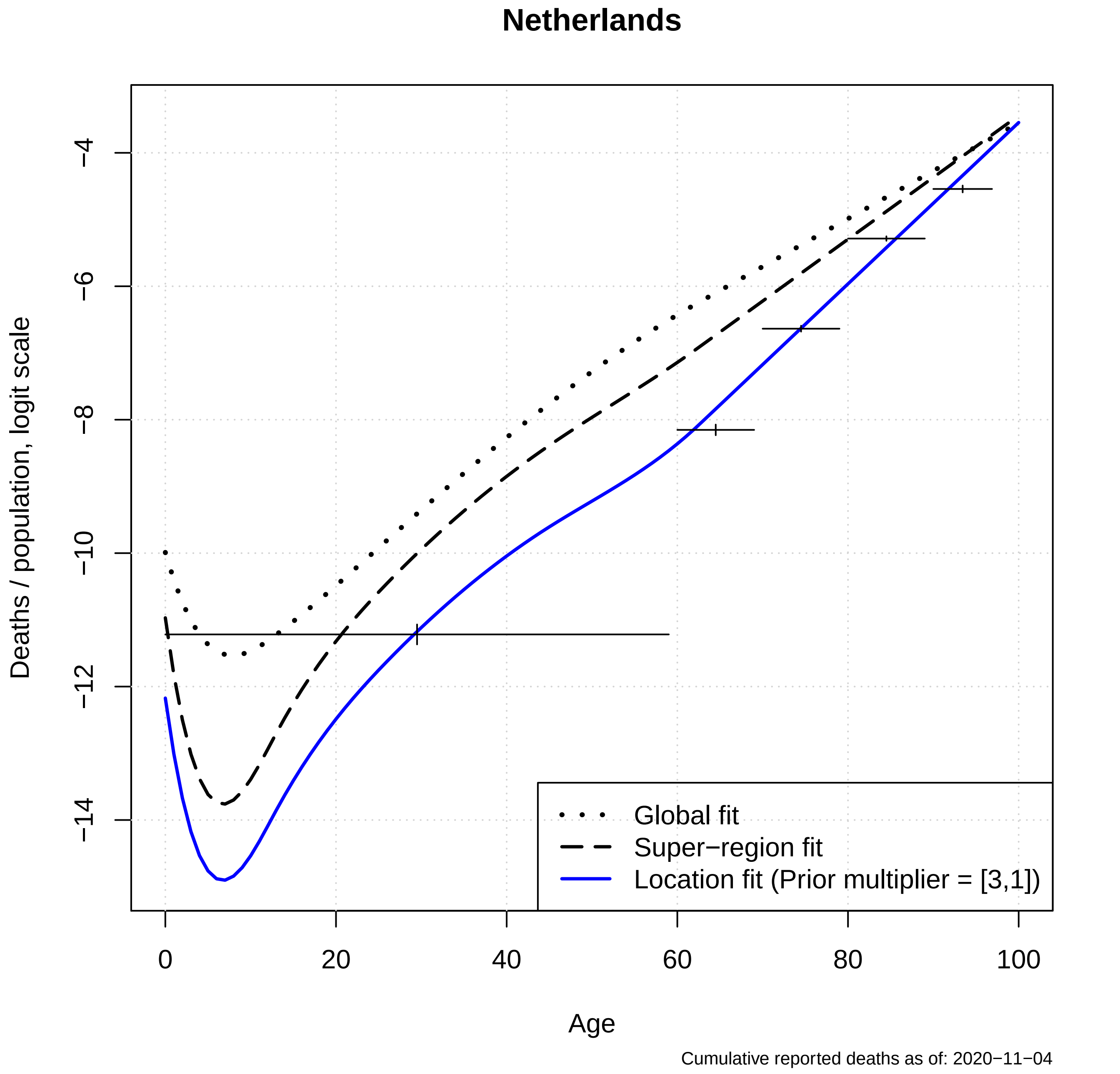
Netherlands
Deaths / population, logit scale
−4
−6
−8
−10
−12
−14
0
20
40
60
80
100
Age
Global fit
Super−region fit
Location fit (Prior multiplier = [3,1])
Cumulative reported deaths as of: 2020−11−04

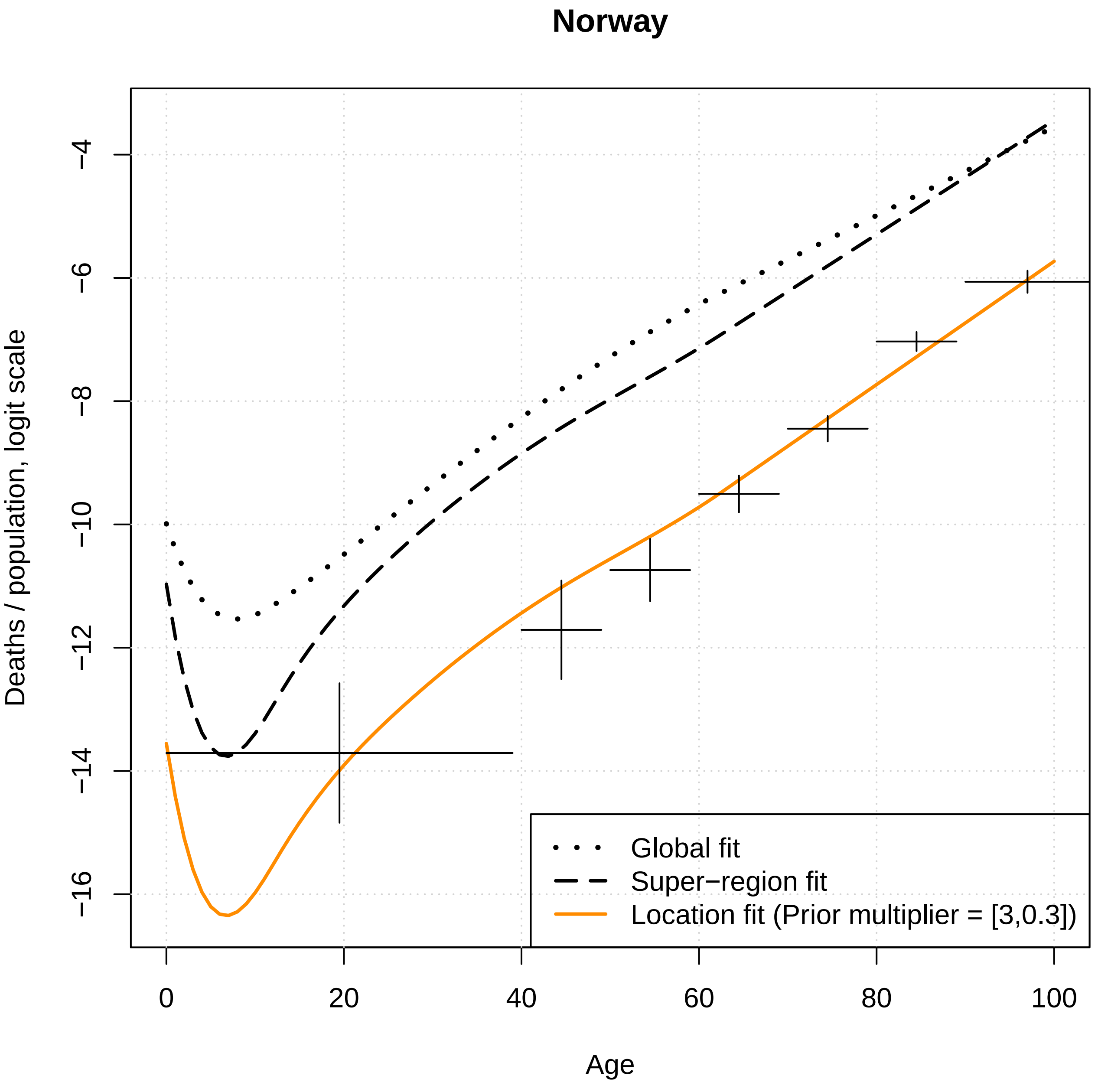

Cumulative reported deaths as of: 2020−12−30

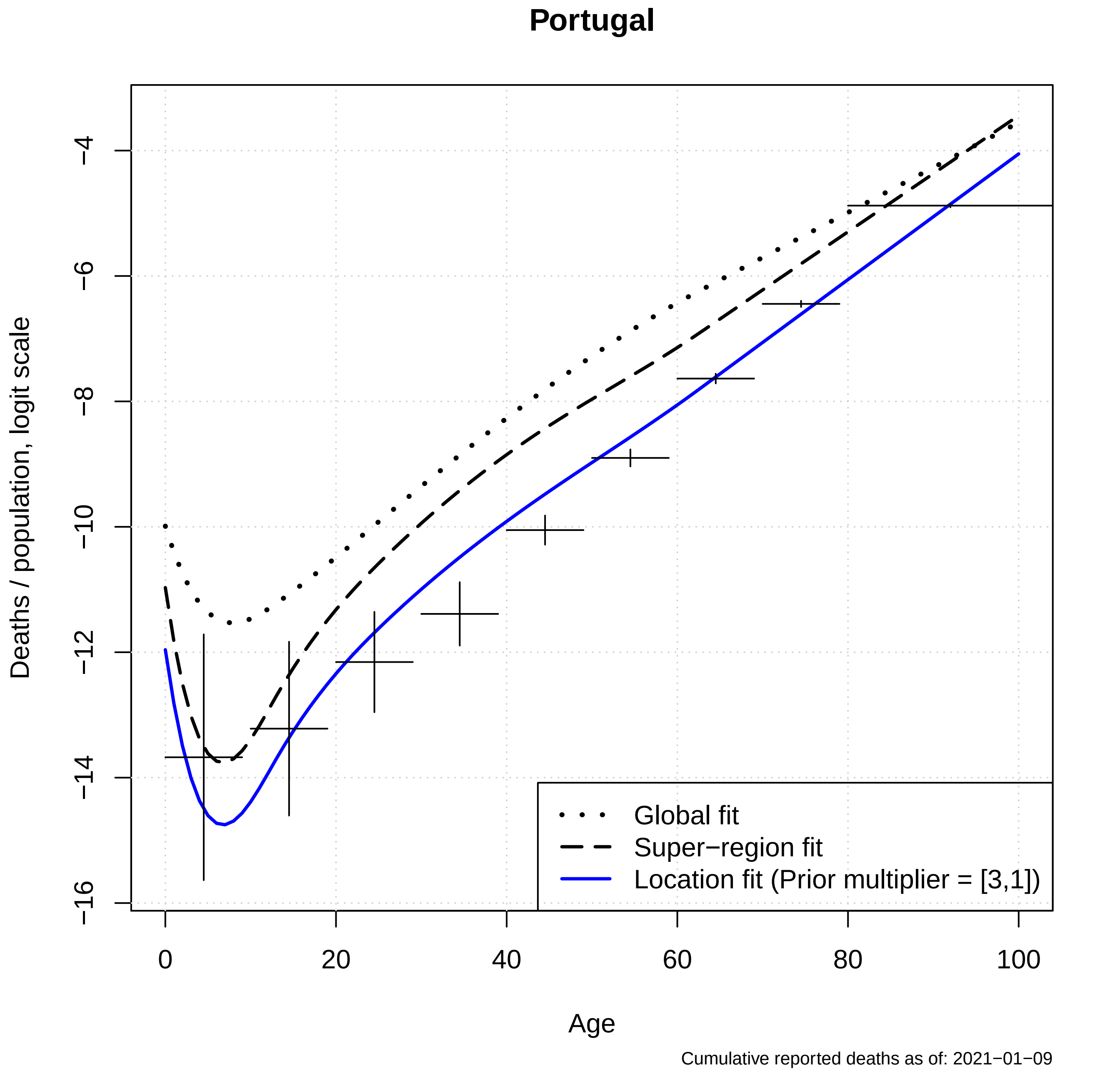
Portugal
Deaths / population, logit scale
−4
−6
−8
−10
−12
−14
−16
0
20
40
60
80
100
Age
Global fit
Super−region fit
Location fit (Prior multiplier = [3,1])
Cumulative reported deaths as of: 2021−01−09

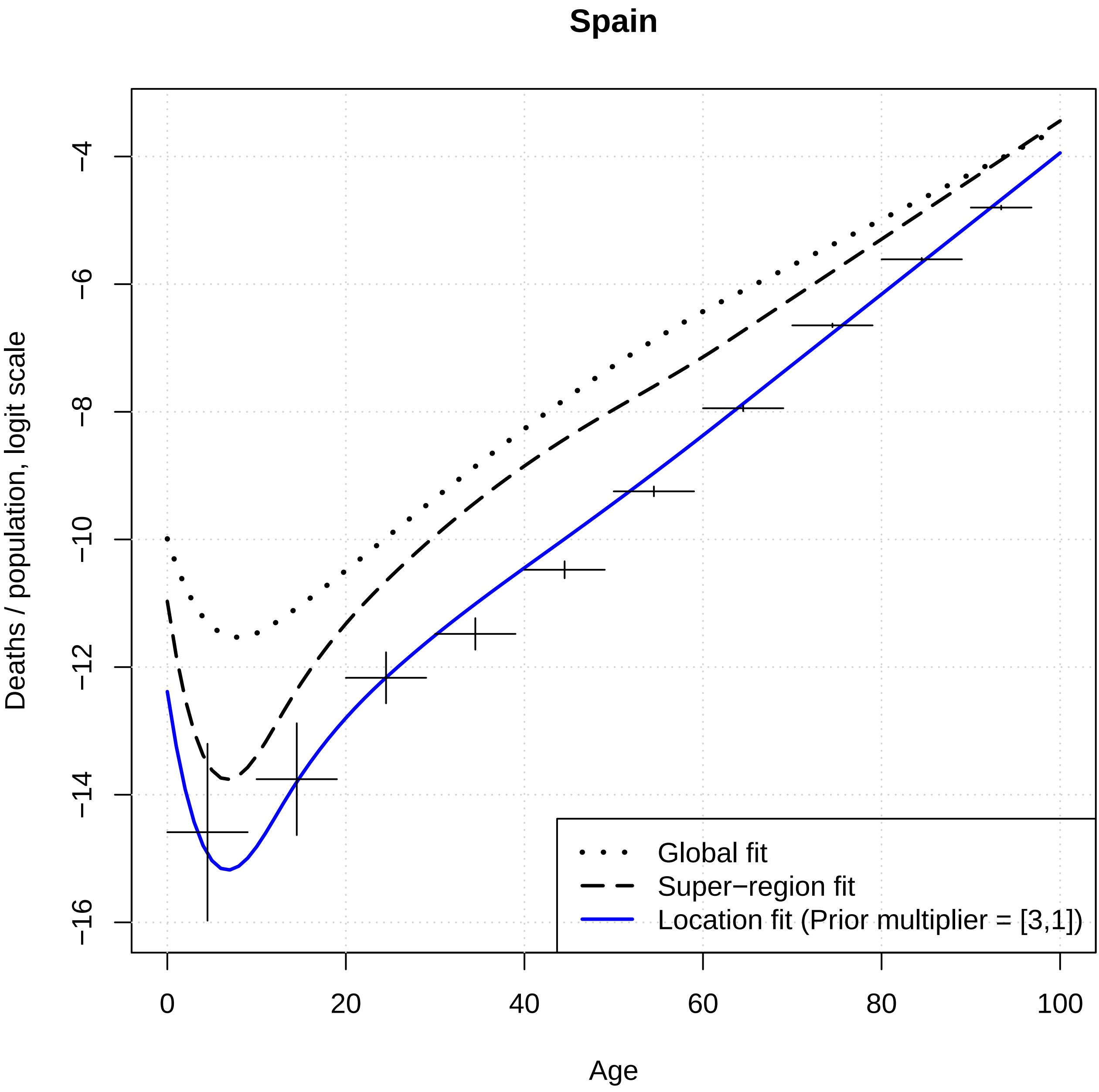

Cumulative reported deaths as of: 2020−05−21

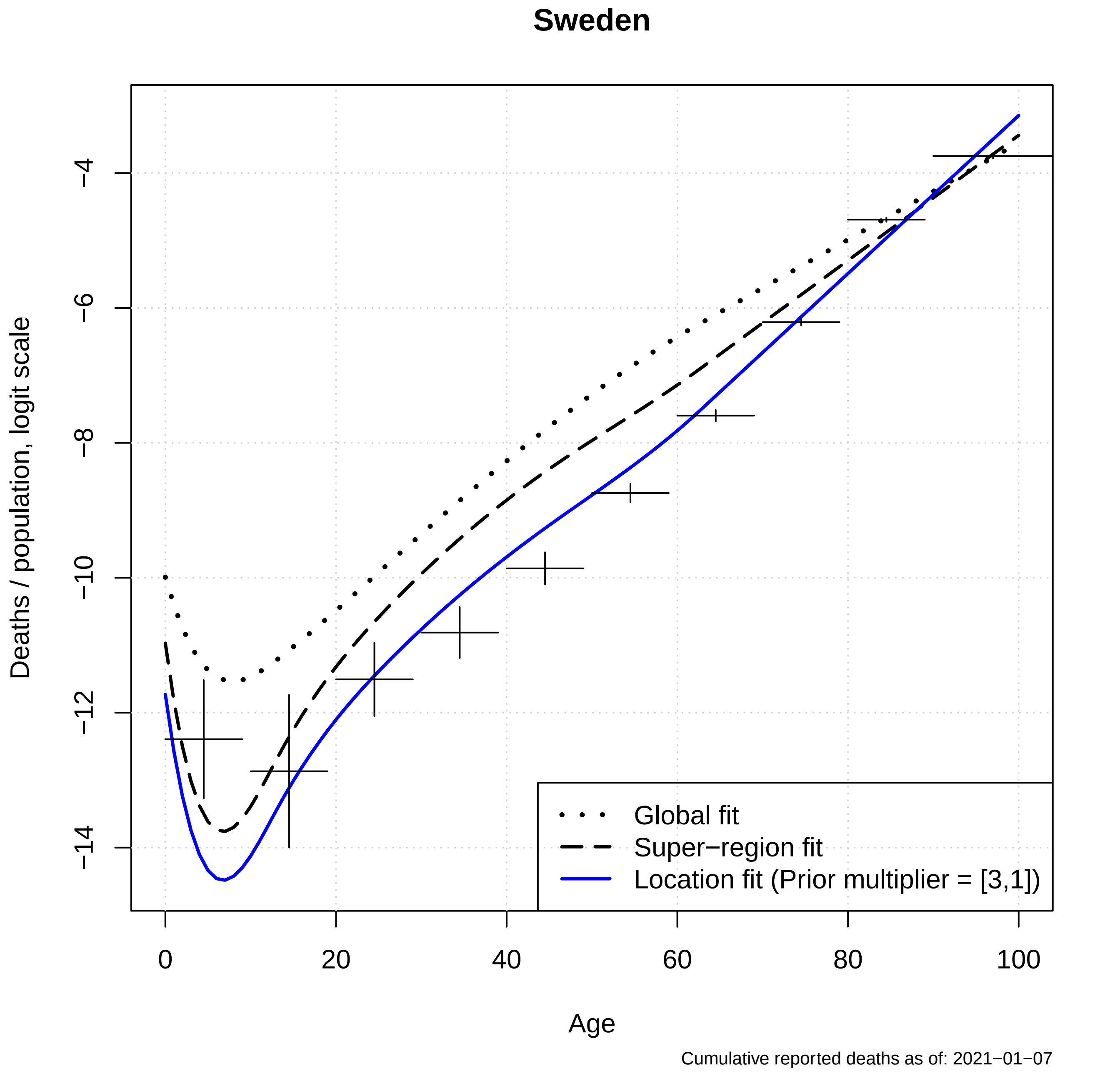

Sweden
Deaths / population, logit scale
−4
−6
−8
−10
−12
−14
0
20
40
60
80
100
Age
Global fit
Super−region fit
Location fit (Prior multiplier = [3,1])
Cumulative reported deaths as of: 2021−01−07

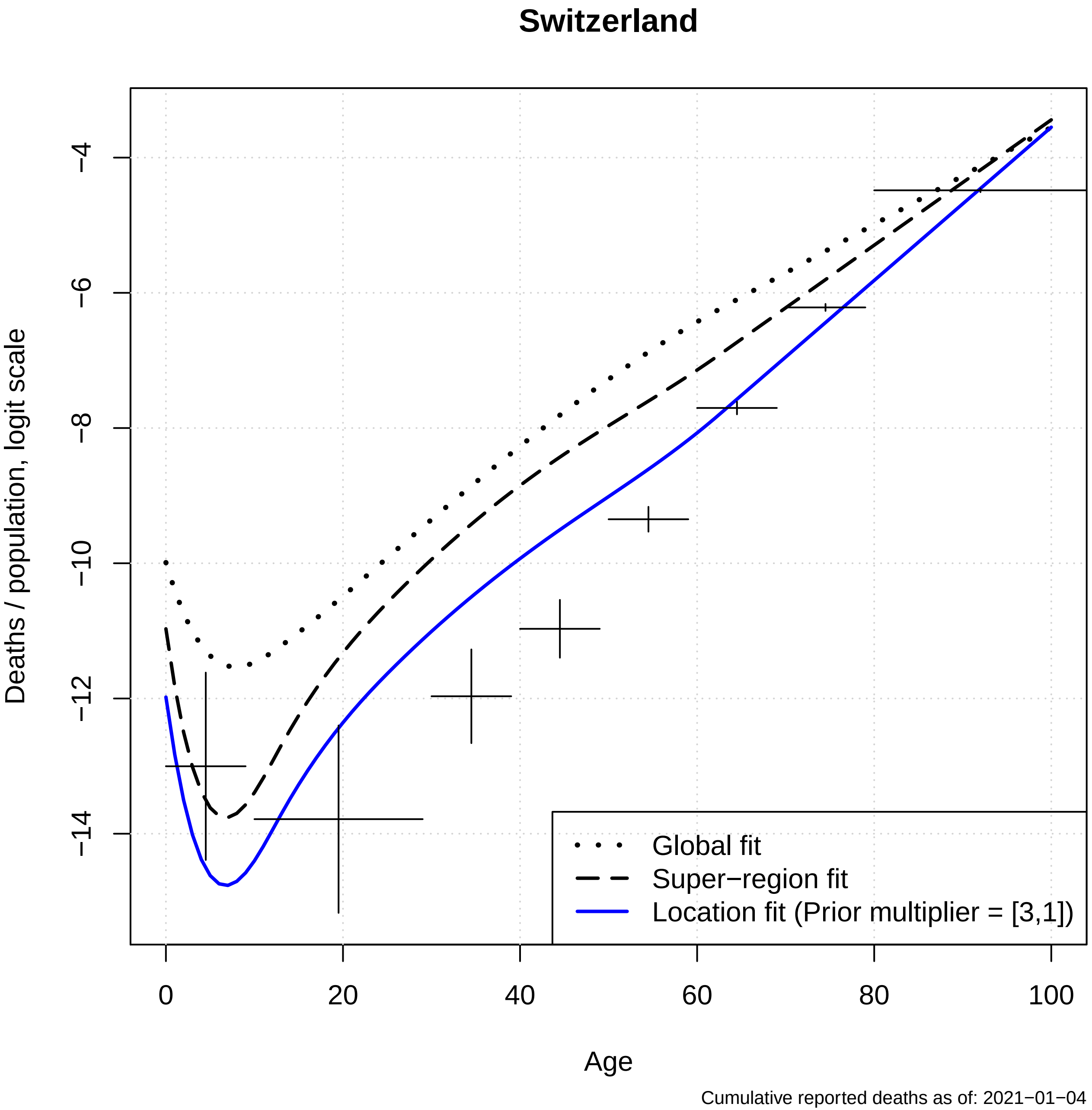
Switzerland
Deaths / population, logit scale
−4
−6
−8
−10
−12
−14
0
20
40
60
80
100
Age
Global fit
Super−region fit
Location fit (Prior multiplier = [3,1])
Cumulative reported deaths as of: 2021−01−04

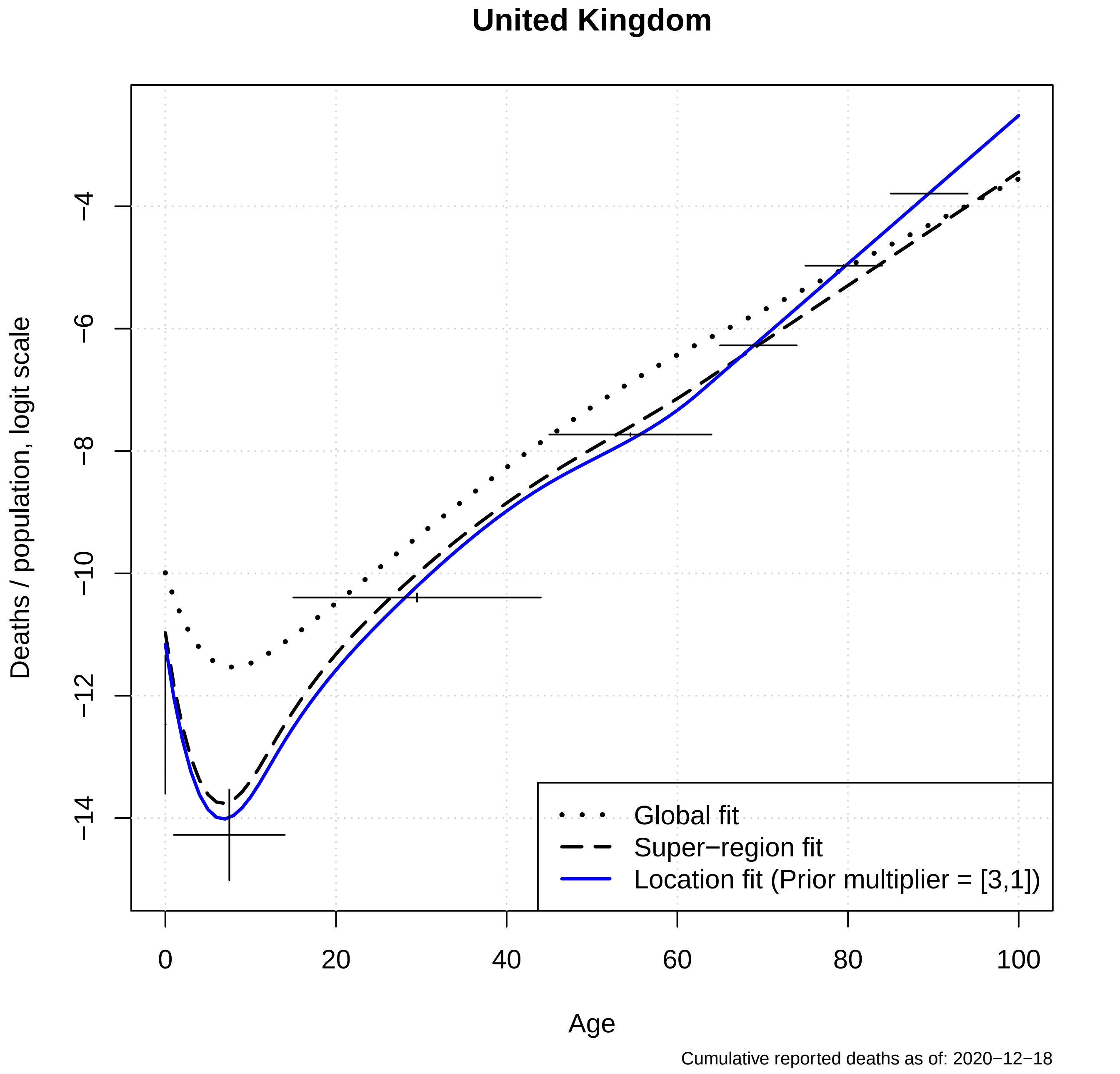
United Kingdom
Deaths / population, logit scale
−4
−6
−8
−10
−12
−14
0
20
40
60
80
100
Age
Global fit
Super−region fit
Location fit (Prior multiplier = [3,1])
Cumulative reported deaths as of: 2020−12−18

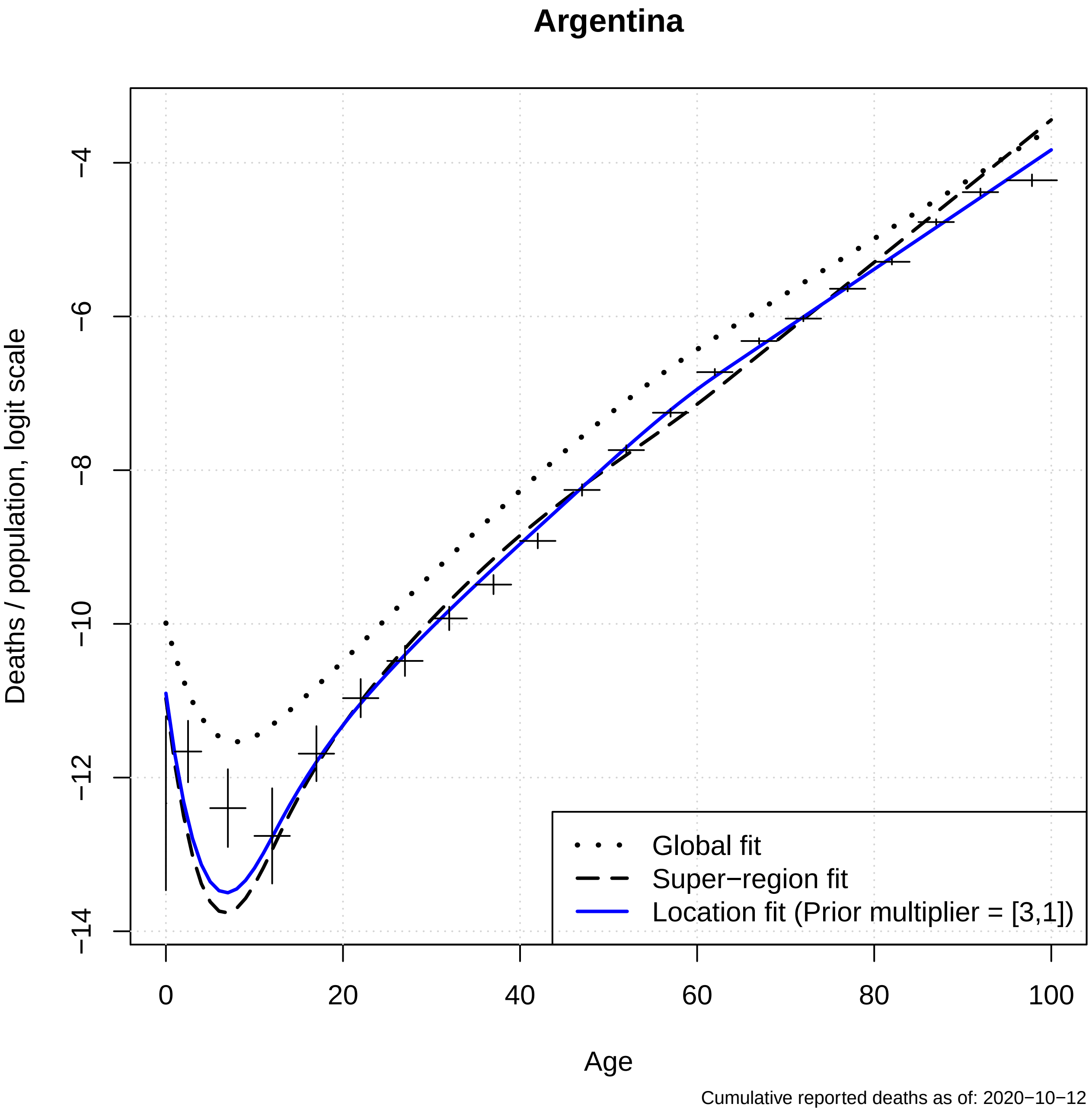
Argentina
Deaths / population, logit scale
−4
−6
−8
−10
−12
−14
0
20
40
60
80
100
Age
Global fit
Super−region fit
Location fit (Prior multiplier = [3,1])
Cumulative reported deaths as of: 2020−10−12

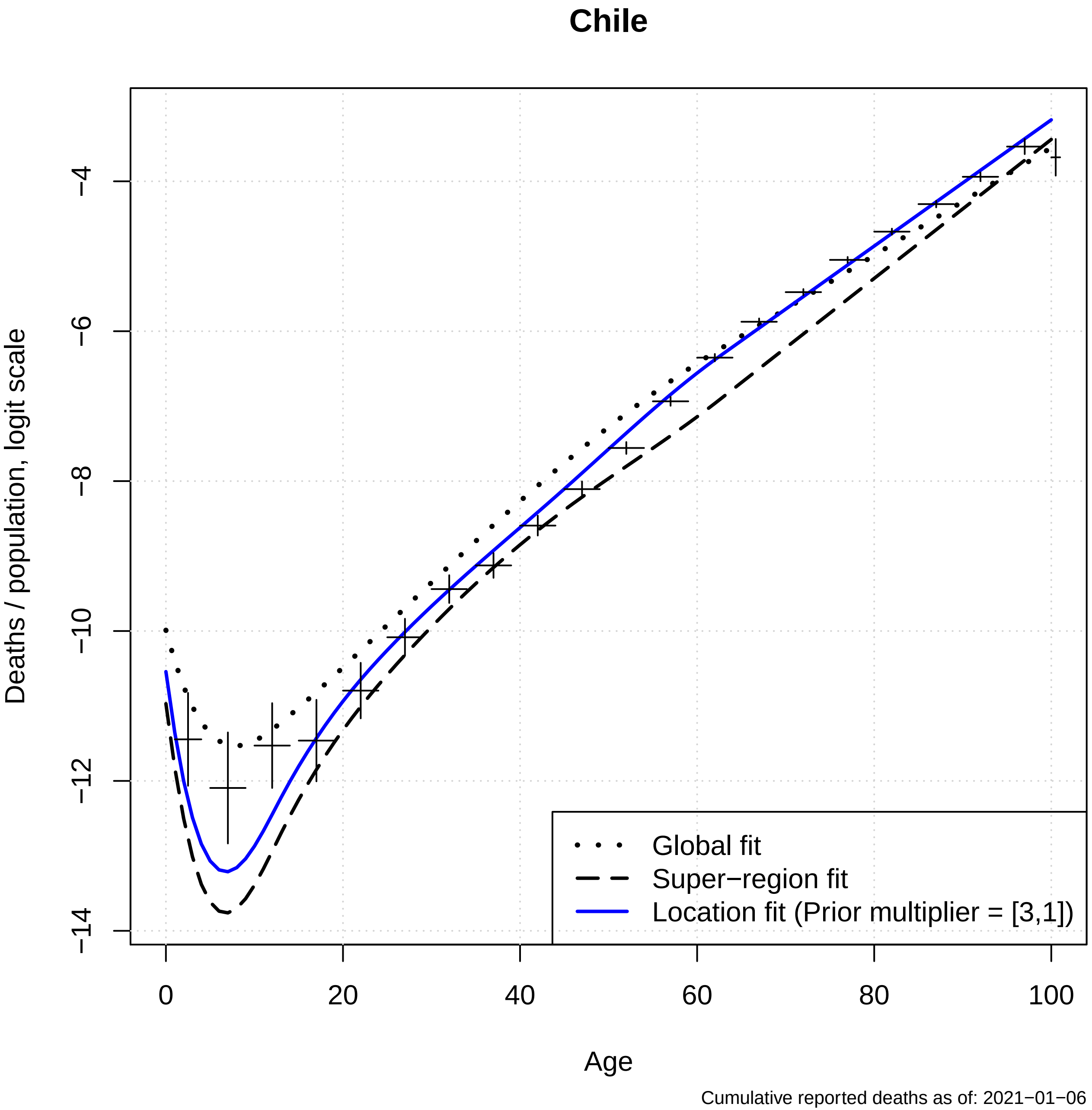

Chile
Deaths / population, logit scale
−4
−6
−8
−10
−12
−14
0
20
40
60
80
100
Age
Global fit
Super−region fit
Location fit (Prior multiplier = [3,1])
Cumulative reported deaths as of: 2021−01−06

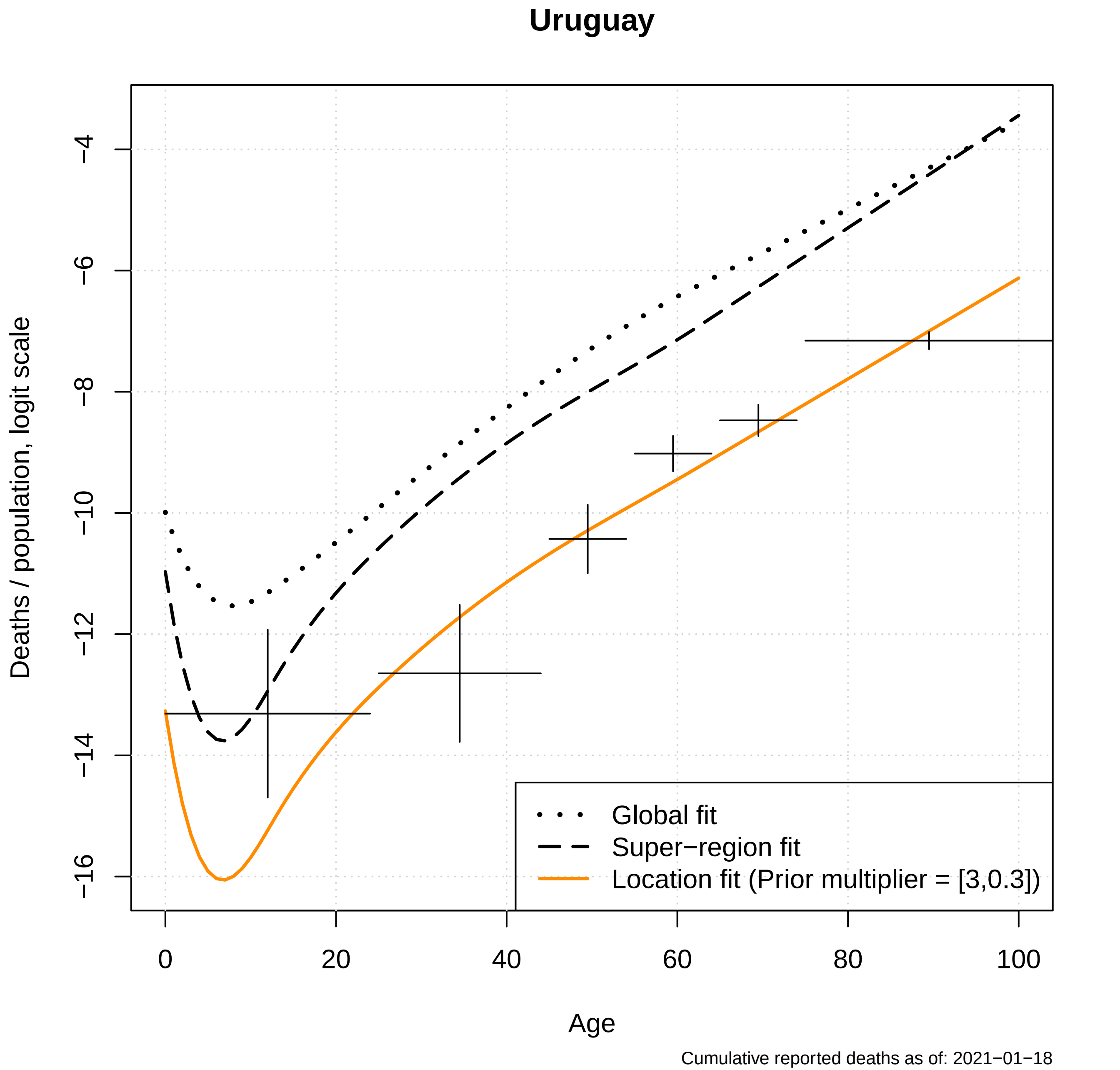
Uruguay
Deaths / population, logit scale
−4
−6
−8
−10
−12
−14
−16
0
20
40
60
80
100
Age
Global fit
Super−region fit
Location fit (Prior multiplier = [3,0.3])
Cumulative reported deaths as of: 2021−01−18

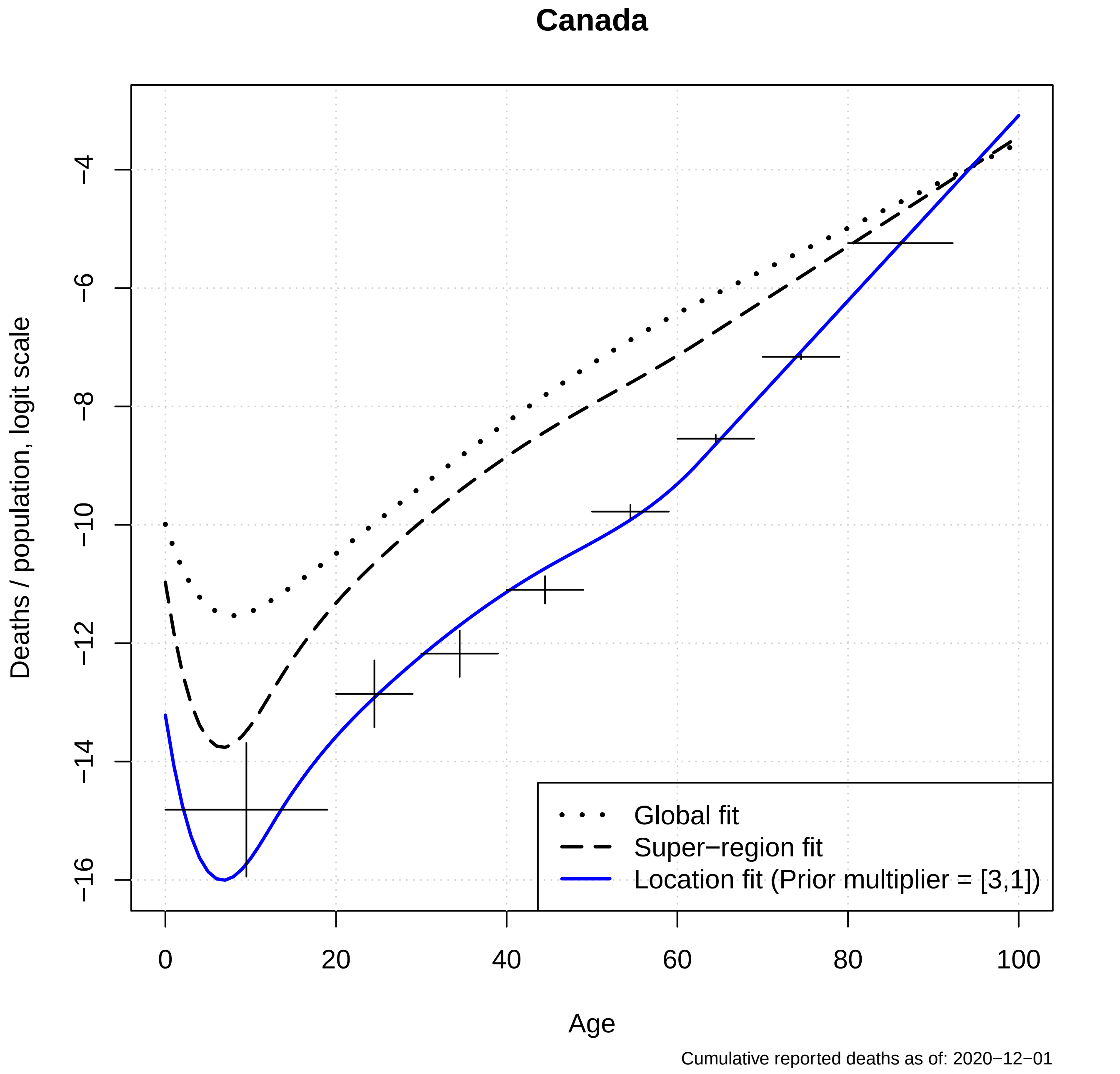

Canada
Deaths / population, logit scale
−4
−6
−8
−10
−12
−14
−16
0
20
40
60
80
100
Age
Global fit
Super−region fit
Location fit (Prior multiplier = [3,1])
Cumulative reported deaths as of: 2020−12−01

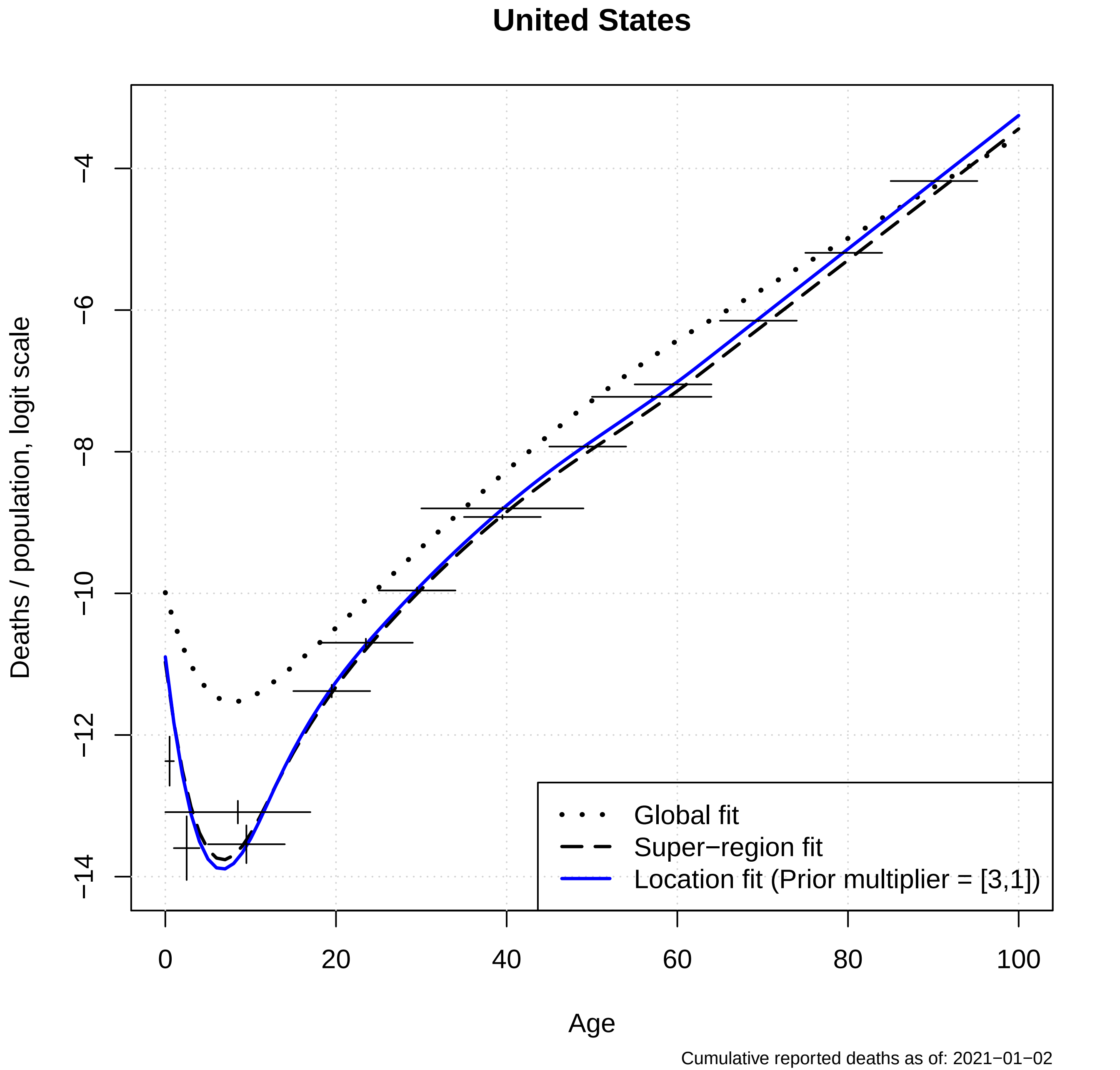

United States
Deaths / population, logit scale
−4
−6
−8
−10
−12
−14
0
20
40
60
80
100
Age
Global fit
Super−region fit
Location fit (Prior multiplier = [3,1])
Cumulative reported deaths as of: 2021−01−02

Figure S4. Age patterns of COVID-19 mortality for select countries and territories in Latin America and the Caribbean

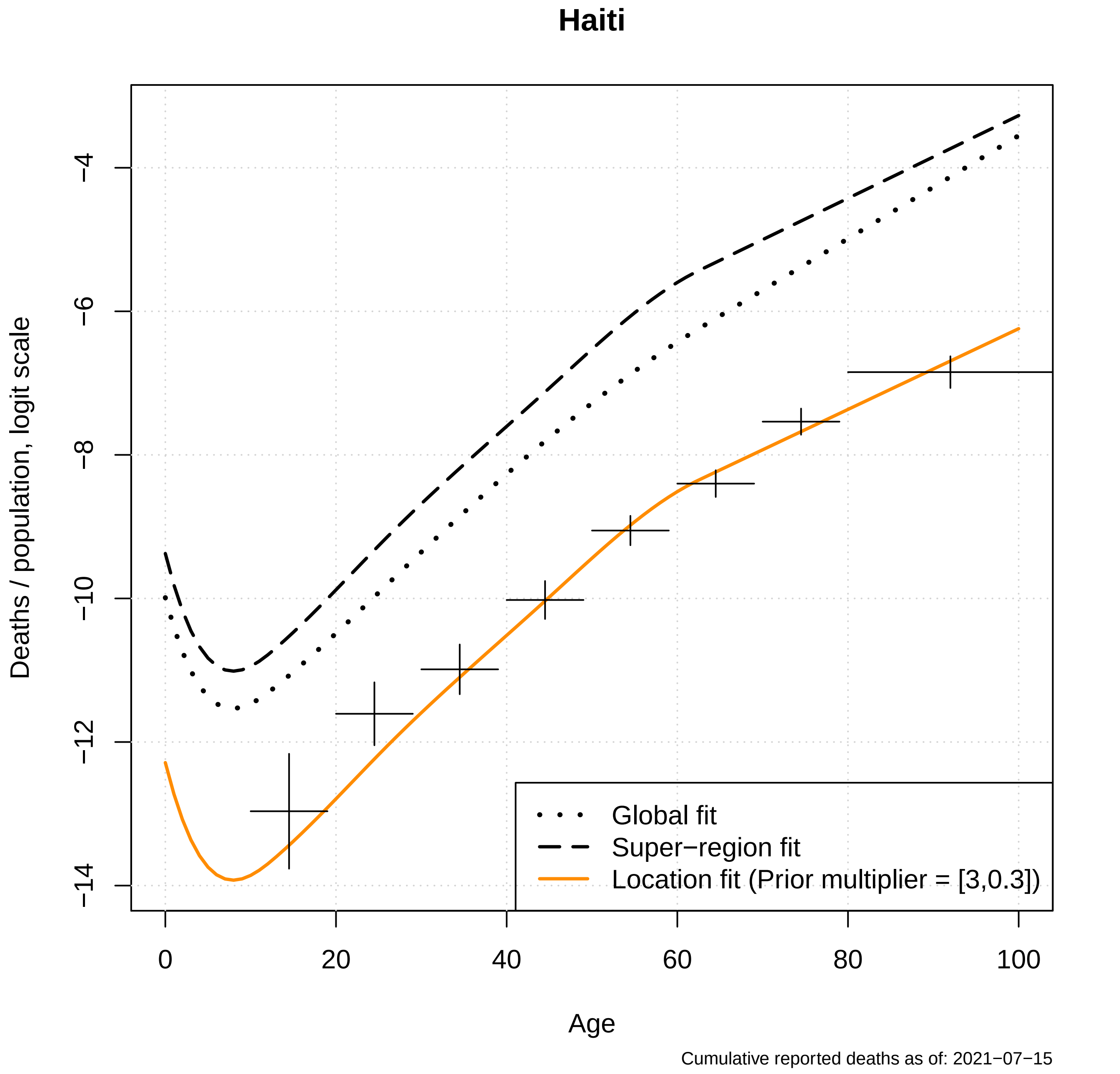

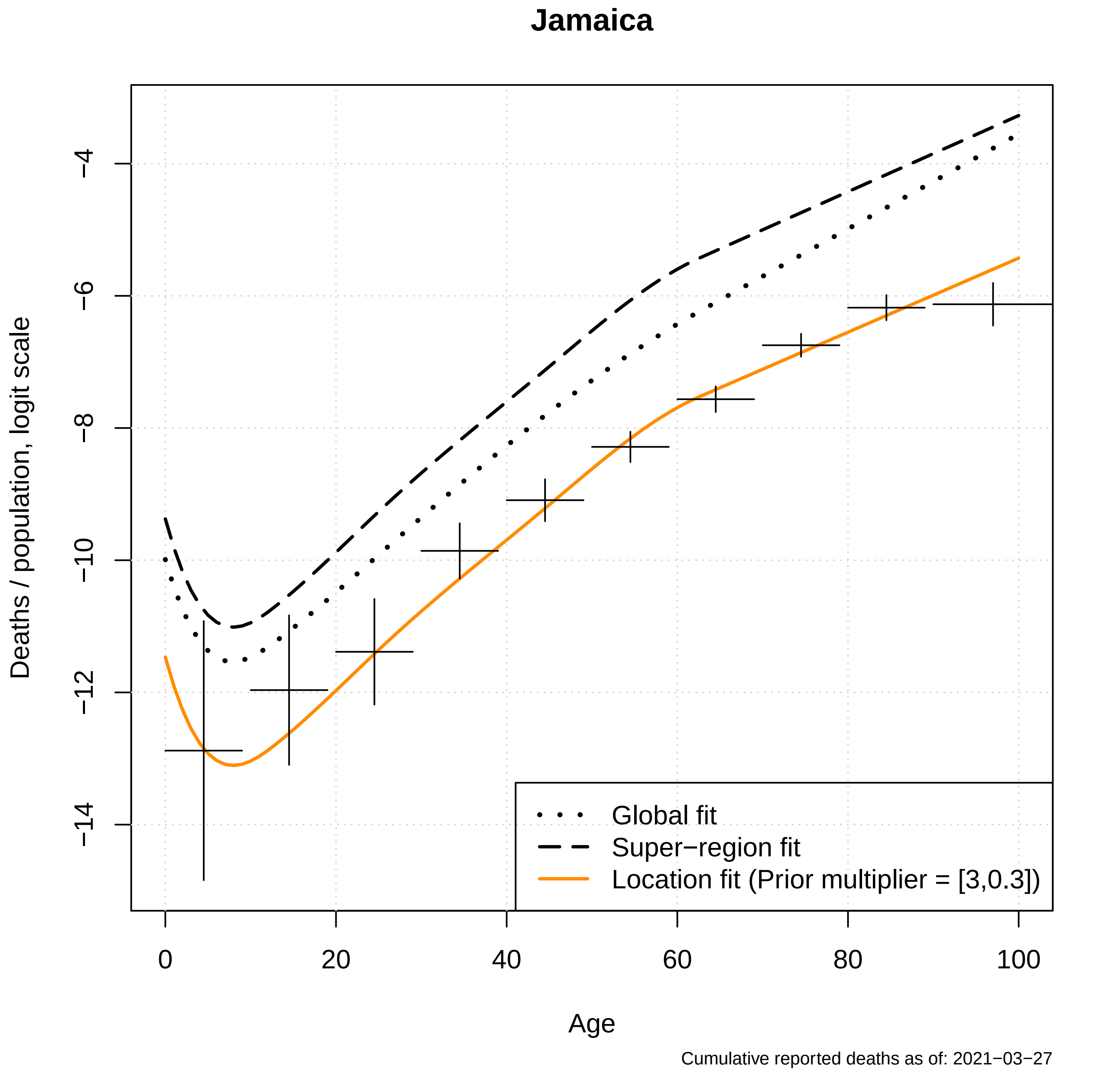

Jamaica
Deaths / population, logit scale
−4
−6
−8
−10
−12
−14
0
20
40
60
80
100
Age
Global fit
Super−region fit
Location fit (Prior multiplier = [3,0.3])
Cumulative reported deaths as of: 2021−03−27

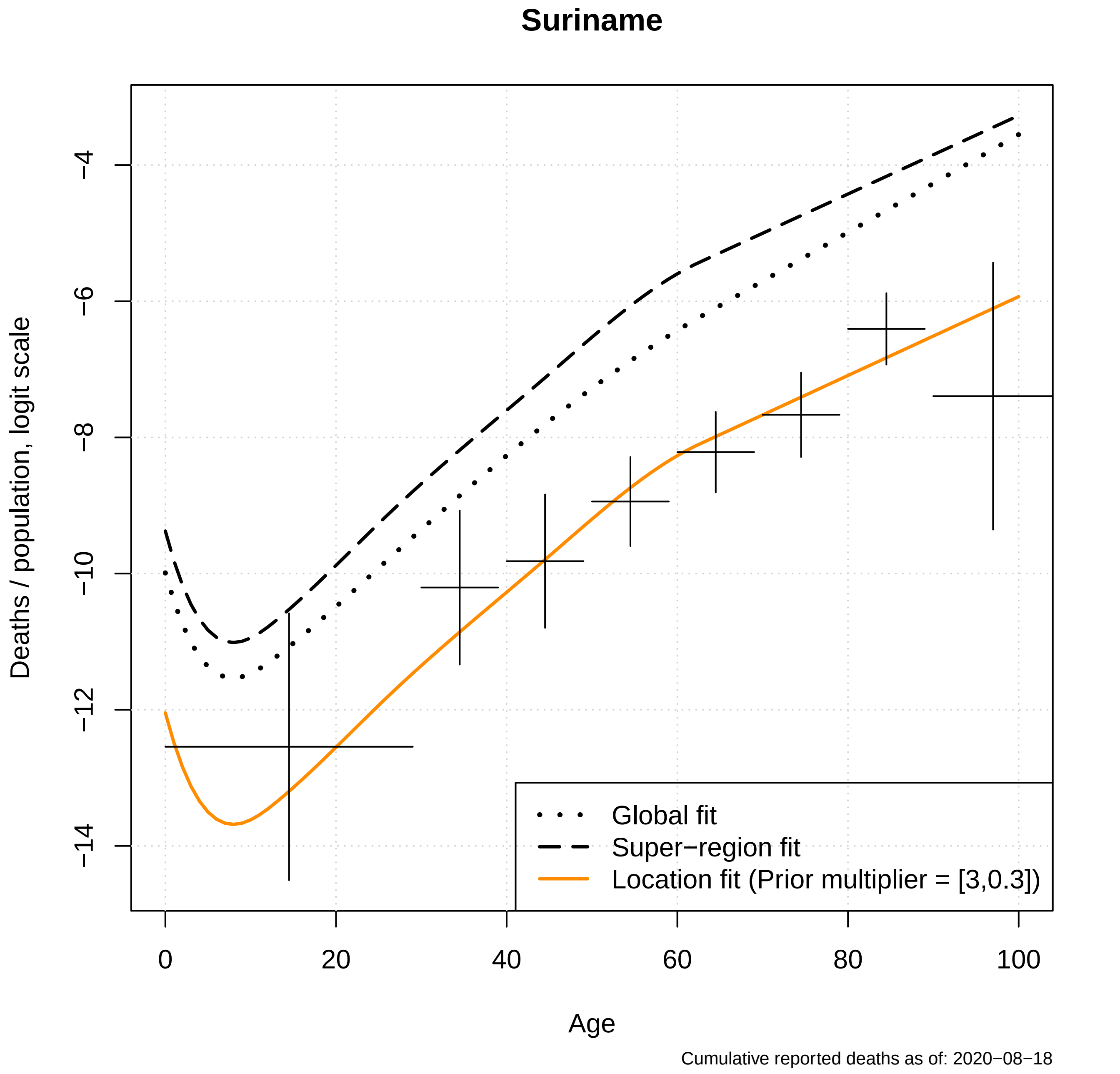

Suriname
Deaths / population, logit scale
−4
−6
−8
−10
−12
−14
0
20
40
60
80
100
Age
Global fit
Super−region fit
Location fit (Prior multiplier = [3,0.3])
Cumulative reported deaths as of: 2020−08−18

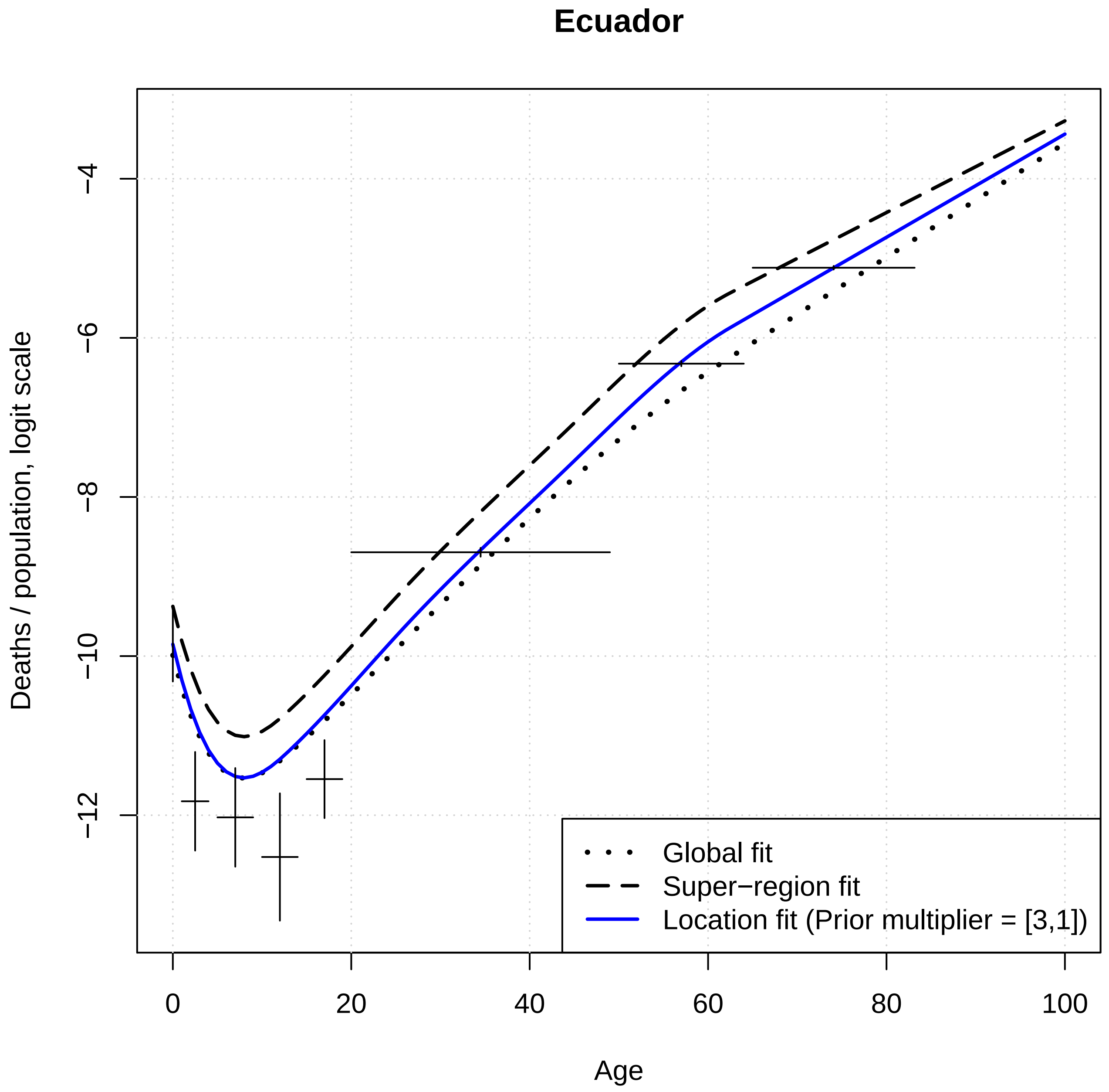

Cumulative reported deaths as of: 2020−11−04

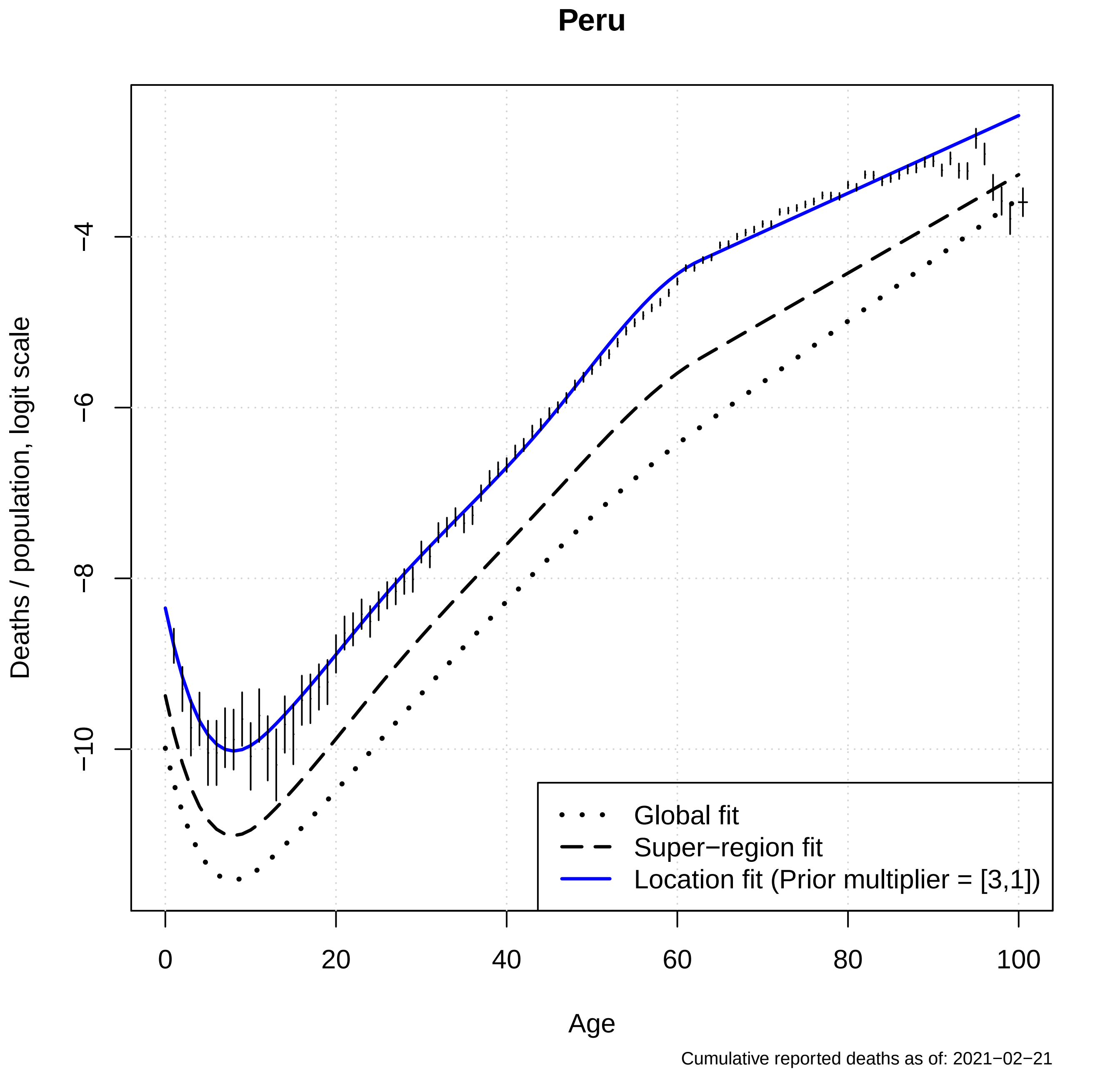

Peru
Deaths / population, logit scale
−4
−6
−8
−10
0
20
40
60
80
100
Age
Global fit
Super−region fit
Location fit (Prior multiplier = [3,1])
Cumulative reported deaths as of: 2021−02−21

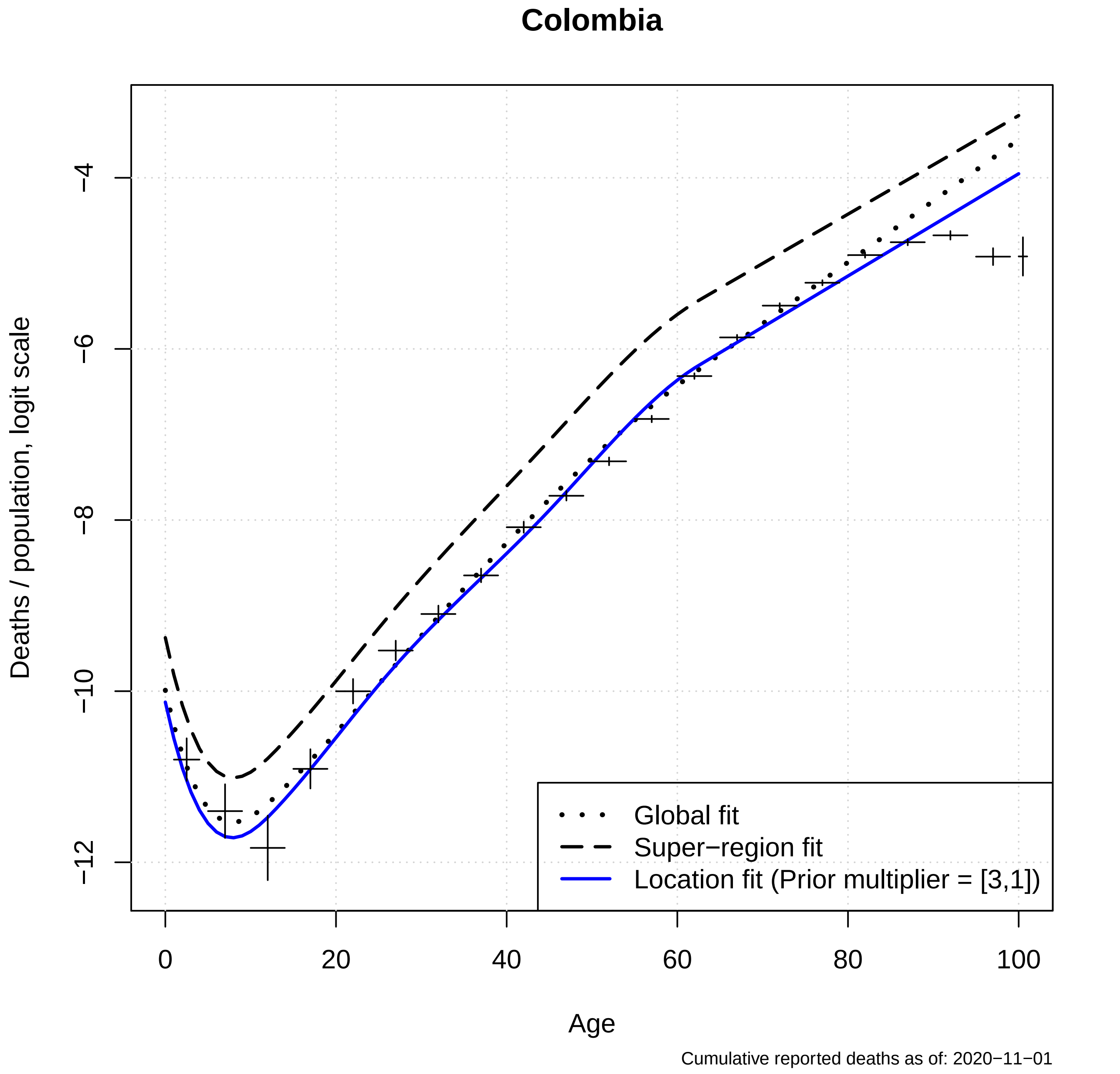

Colombia
Deaths / population, logit scale
−4
−6
−8
−10
−12
0
20
40
60
80
100
Age
Global fit
Super−region fit
Location fit (Prior multiplier = [3,1])
Cumulative reported deaths as of: 2020−11−01

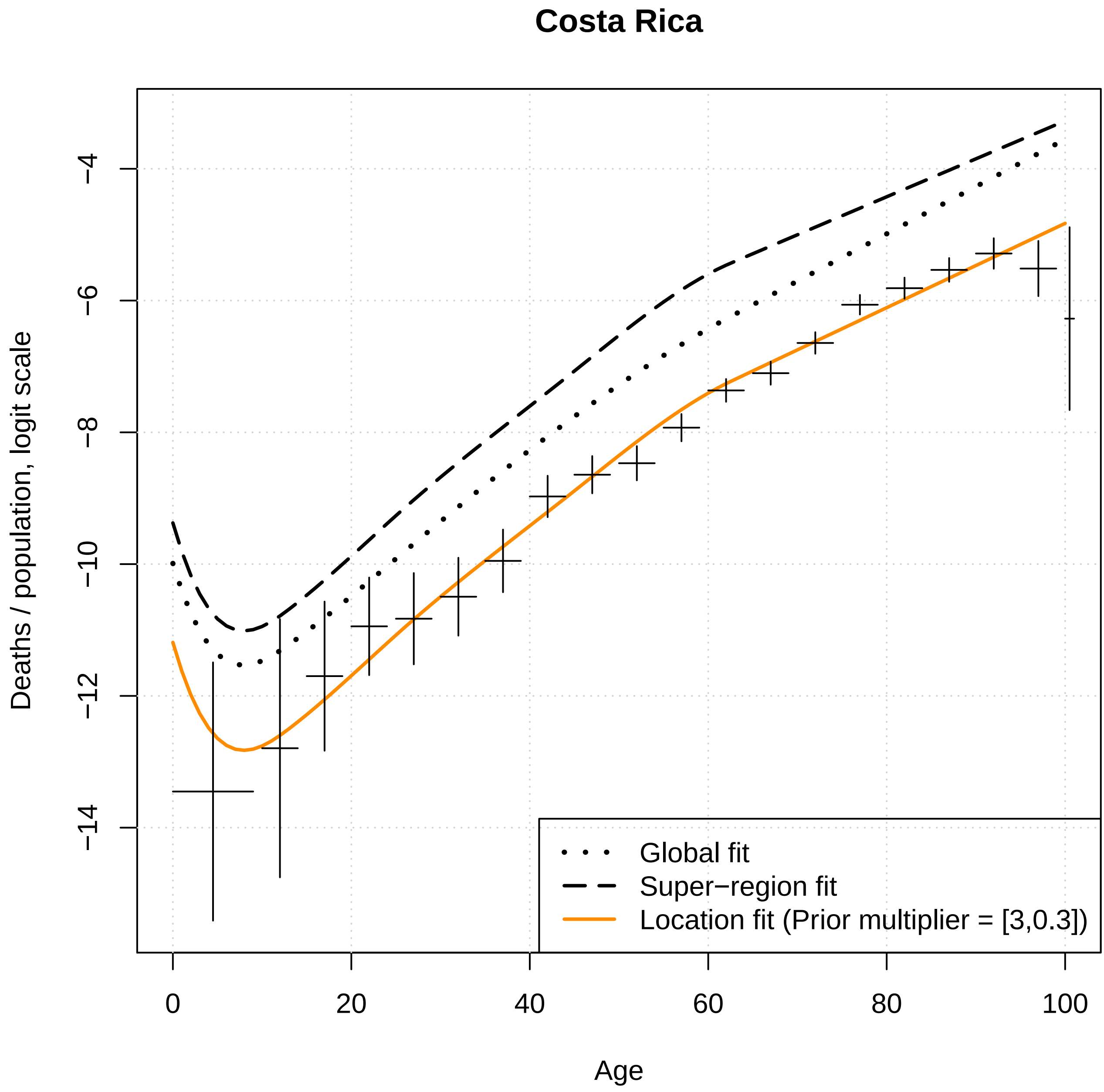

Cumulative reported deaths as of: 2020−10−20

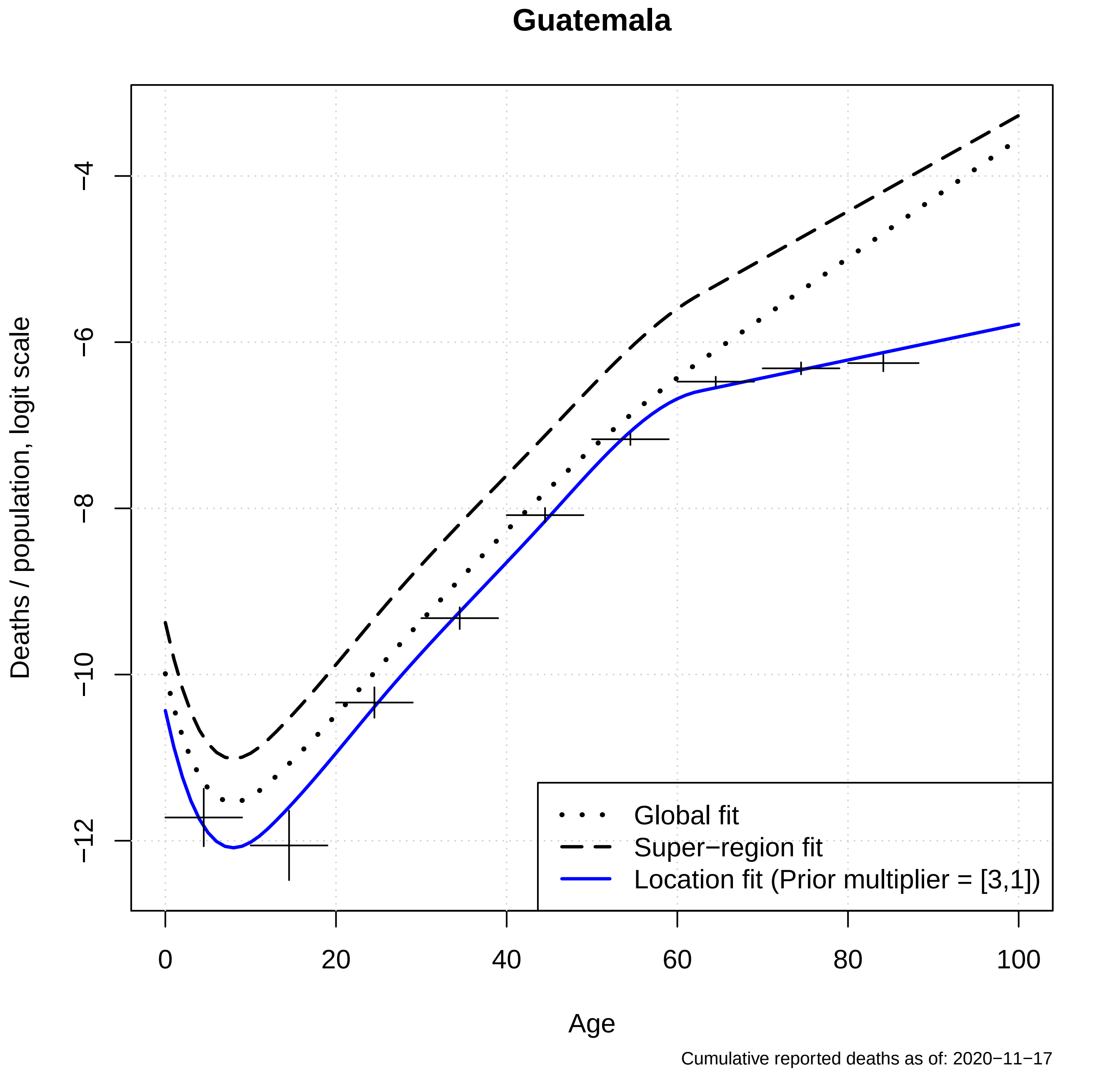

Guatemala
Deaths / population, logit scale
−4
−6
−8
−10
−12
0
20
40
60
80
100
Age
Global fit
Super−region fit
Location fit (Prior multiplier = [3,1])
Cumulative reported deaths as of: 2020−11−17

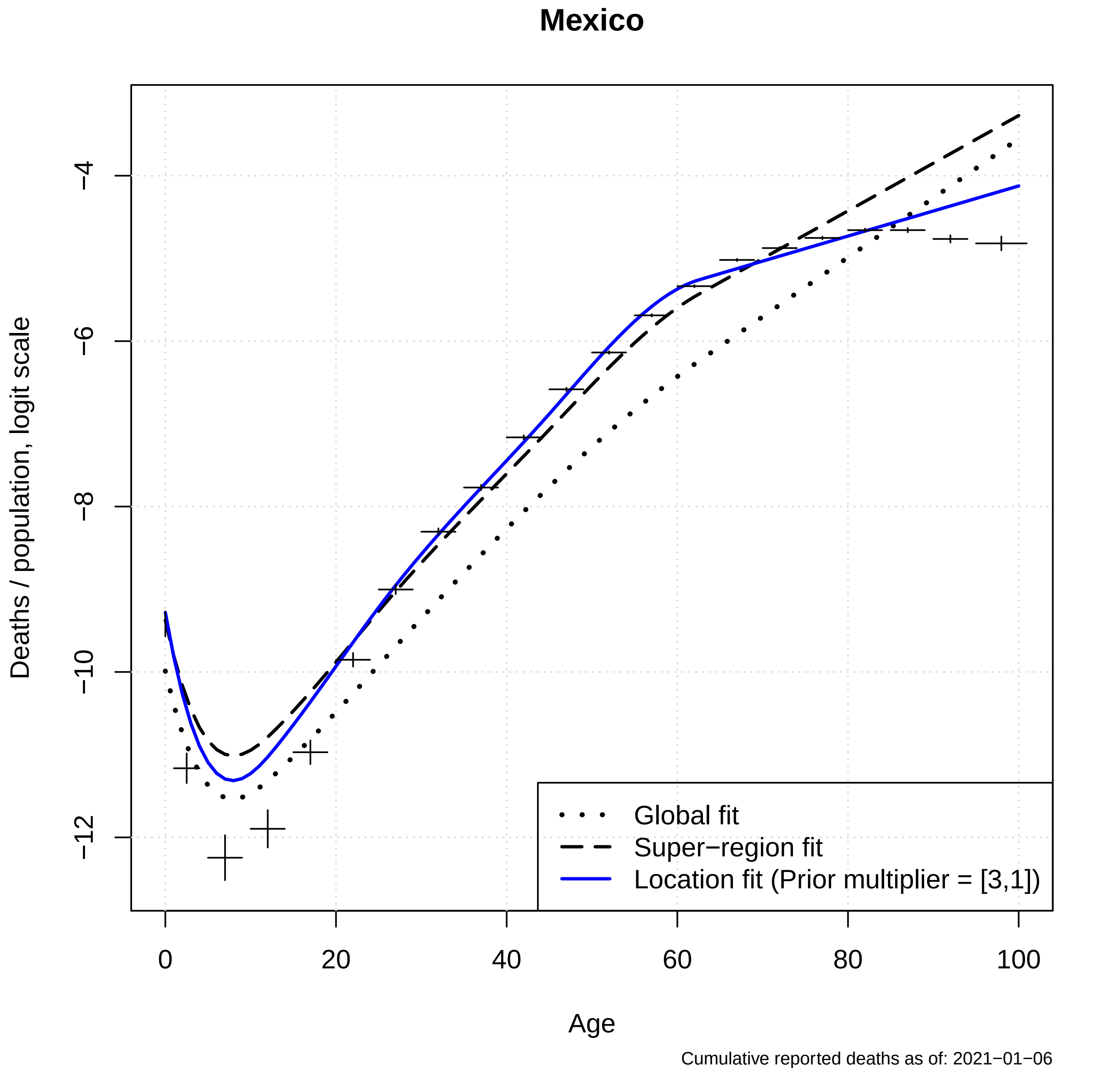

Mexico
Deaths / population, logit scale
−4
−6
−8
−10
−12
0
20
40
60
80
100
Age
Global fit
Super−region fit
Location fit (Prior multiplier = [3,1])
Cumulative reported deaths as of: 2021−01−06

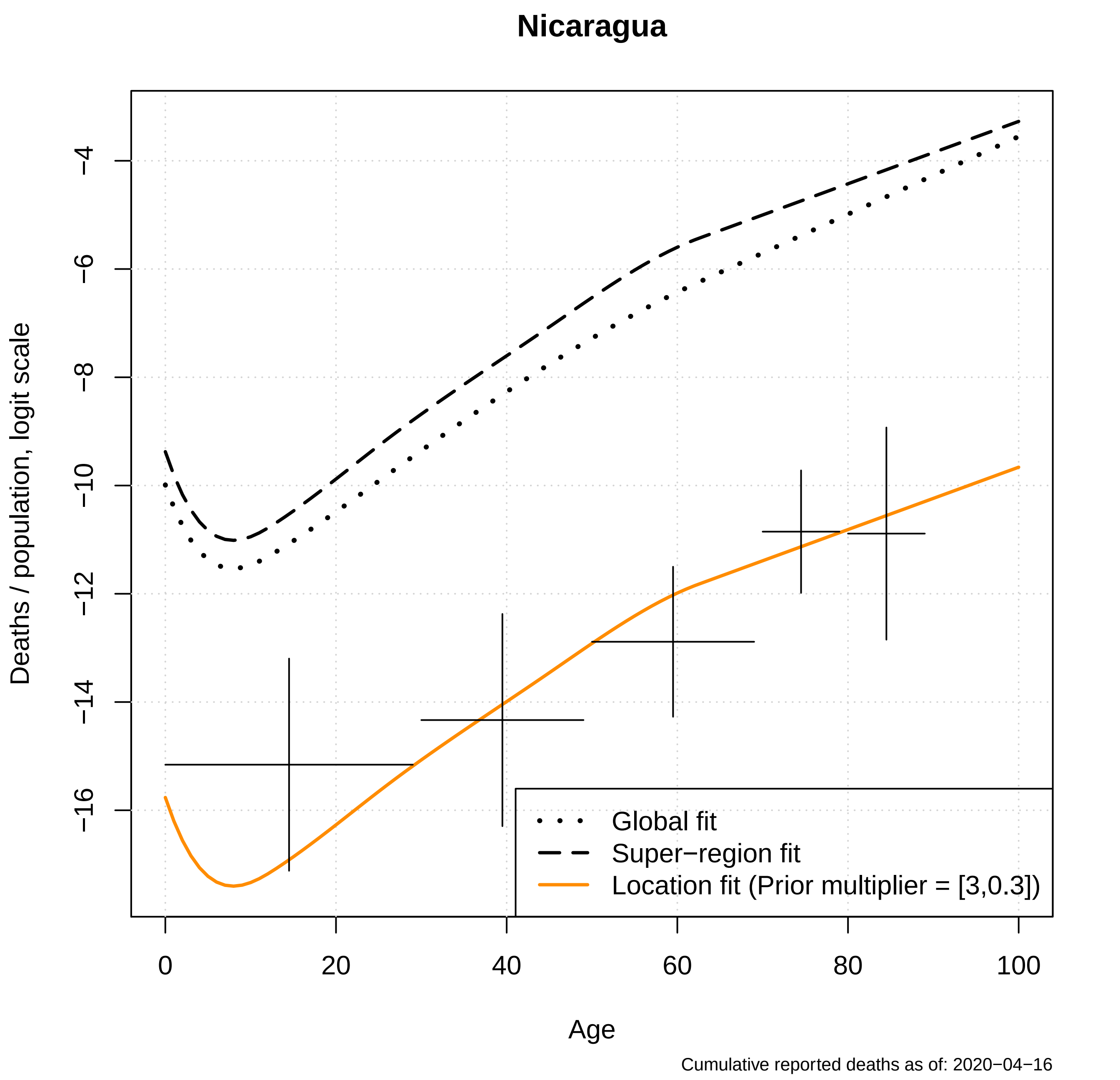

Nicaragua
Deaths / population, logit scale
−4
−6
−8
−10
−12
−14
−16
0
20
40
60
80
100
Age
Global fit
Super−region fit
Location fit (Prior multiplier = [3,0.3])
Cumulative reported deaths as of: 2020−04−16

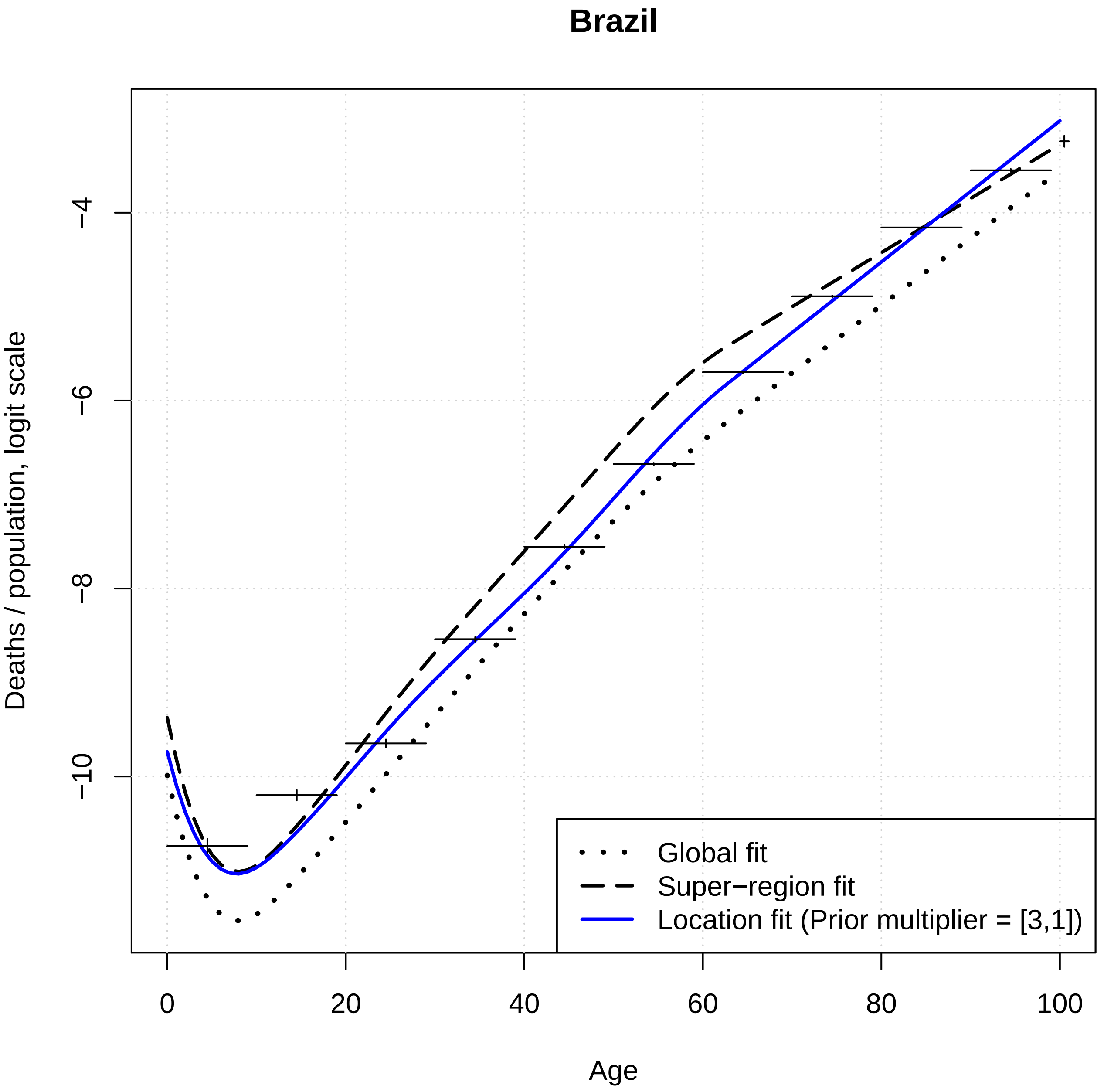

Cumulative reported deaths as of: 2021−02−21

Figure S5. Age patterns of COVID-19 mortality for select countries and territories in north Africa and the Middle East

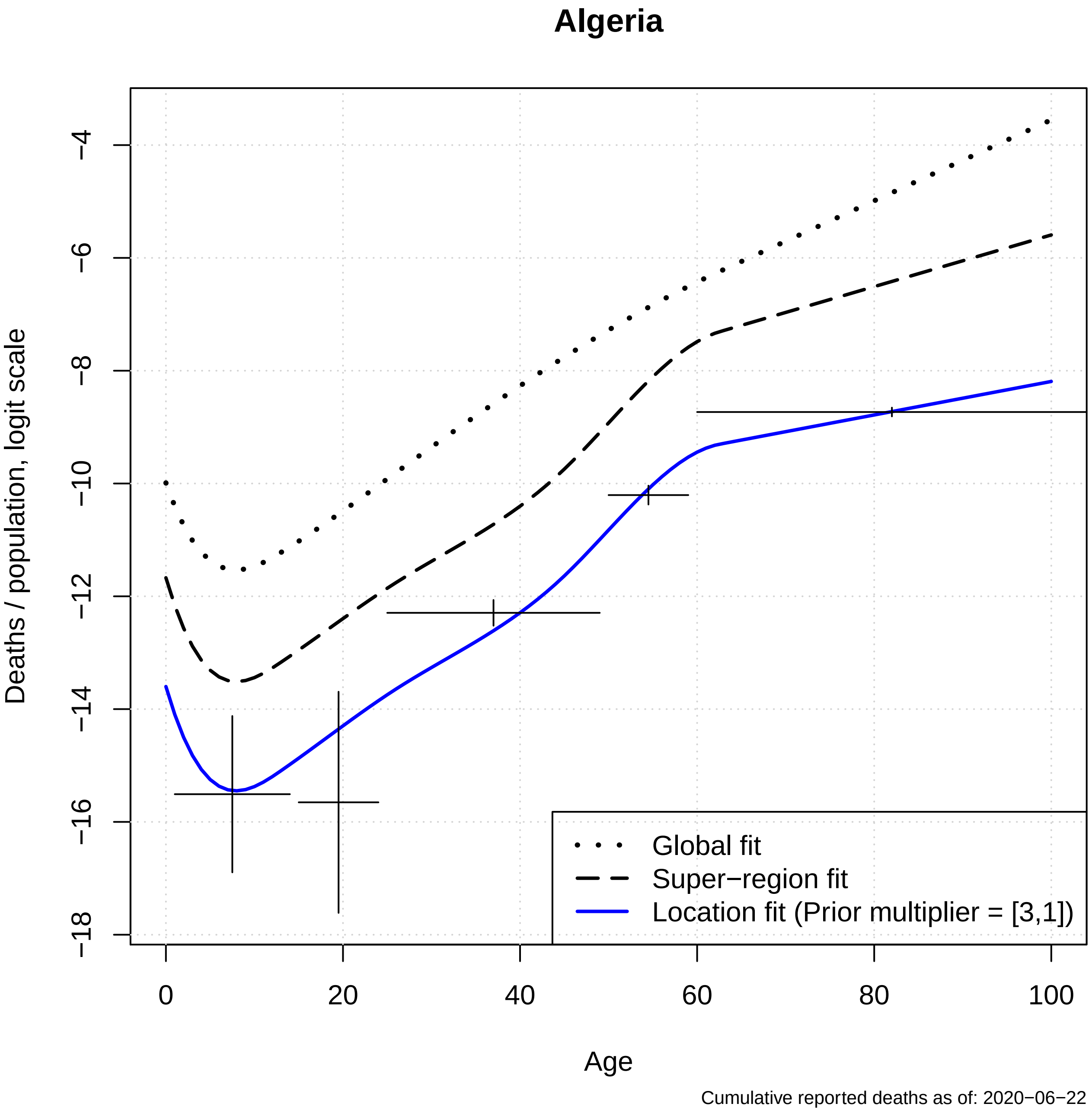

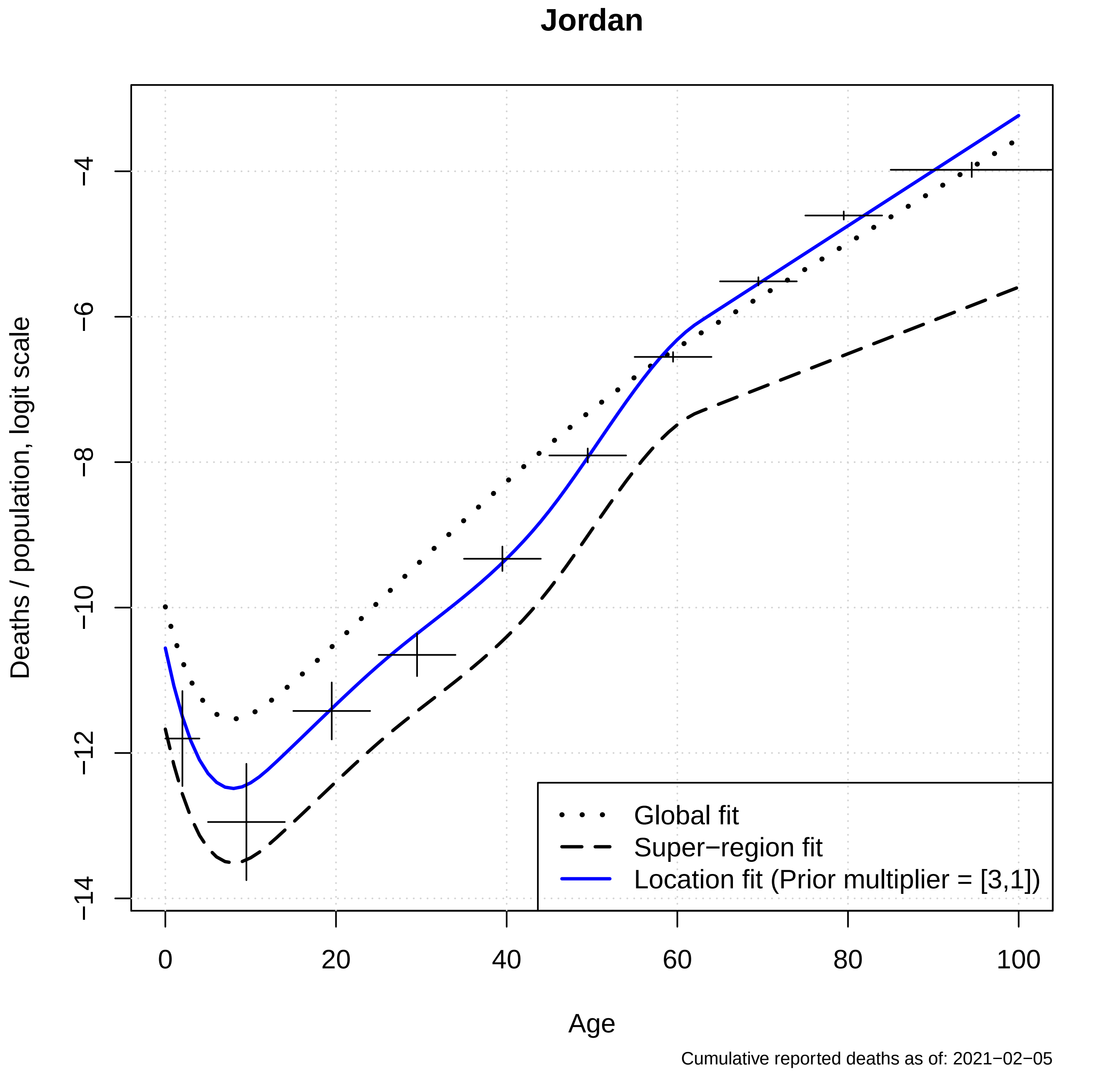
Jordan
Deaths / population, logit scale
−4
−6
−8
−10
−12
−14
0
20
40
60
80
100
Age
Global fit
Super−region fit
Location fit (Prior multiplier = [3,1])
Cumulative reported deaths as of: 2021−02−05

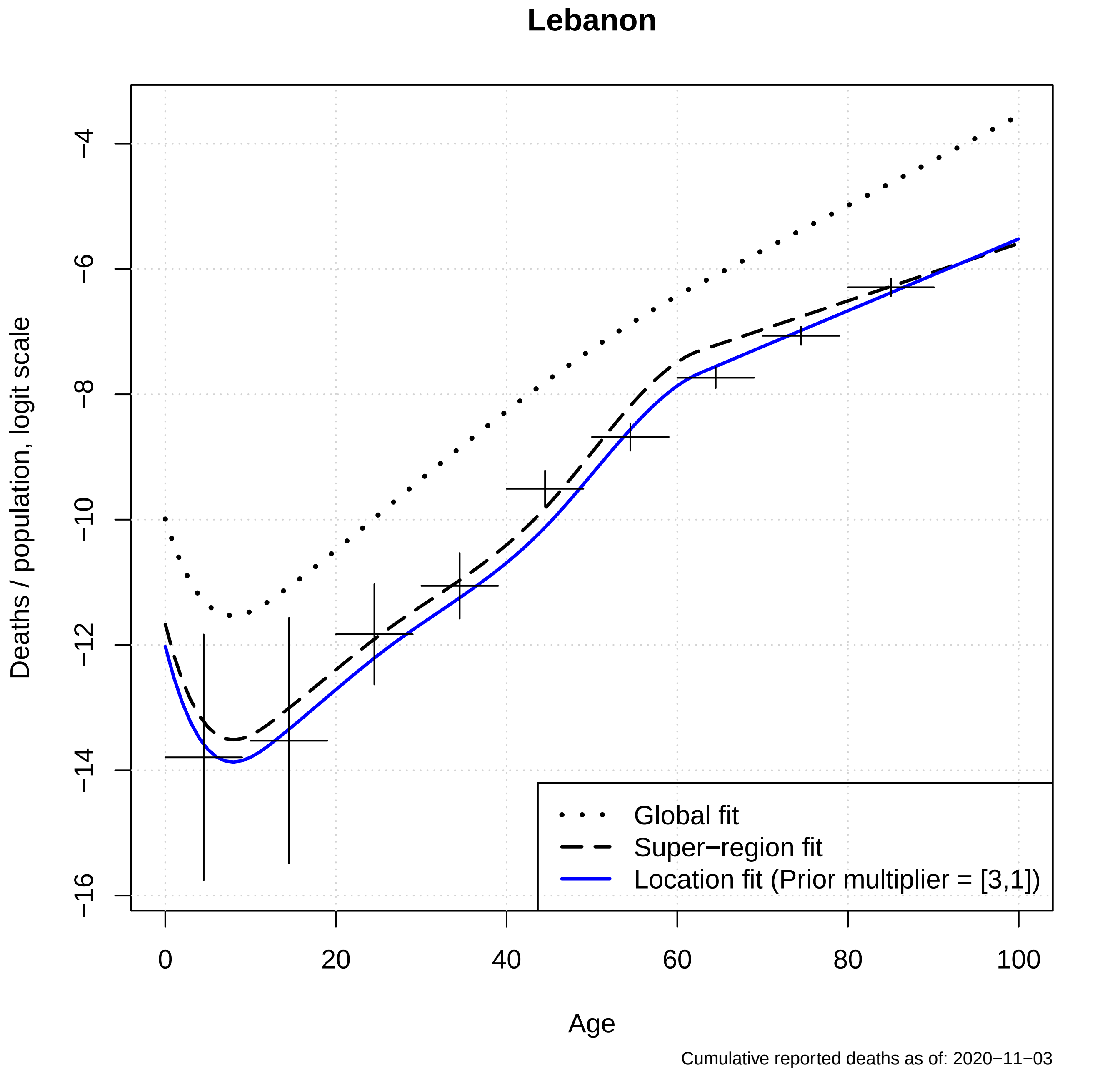

Lebanon
Deaths / population, logit scale
−4
−6
−8
−10
−12
−14
−16
0
20
40
60
80
100
Age
Global fit
Super−region fit
Location fit (Prior multiplier = [3,1])
Cumulative reported deaths as of: 2020−11−03

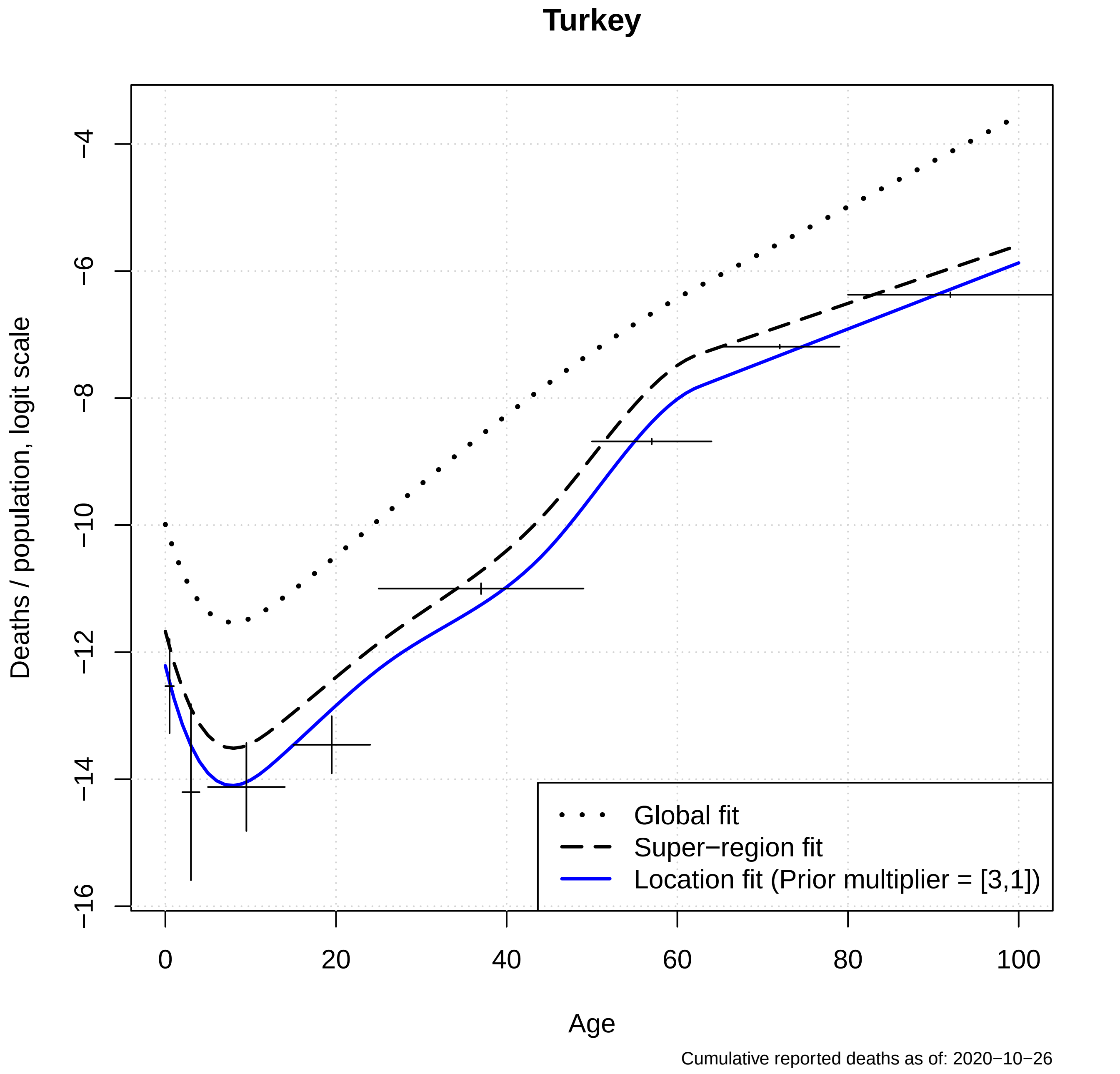

Turkey
Deaths / population, logit scale
−4
−6
−8
−10
−12
−14
−16
0
20
40
60
80
100
Age
Global fit
Super−region fit
Location fit (Prior multiplier = [3,1])
Cumulative reported deaths as of: 2020−10−26

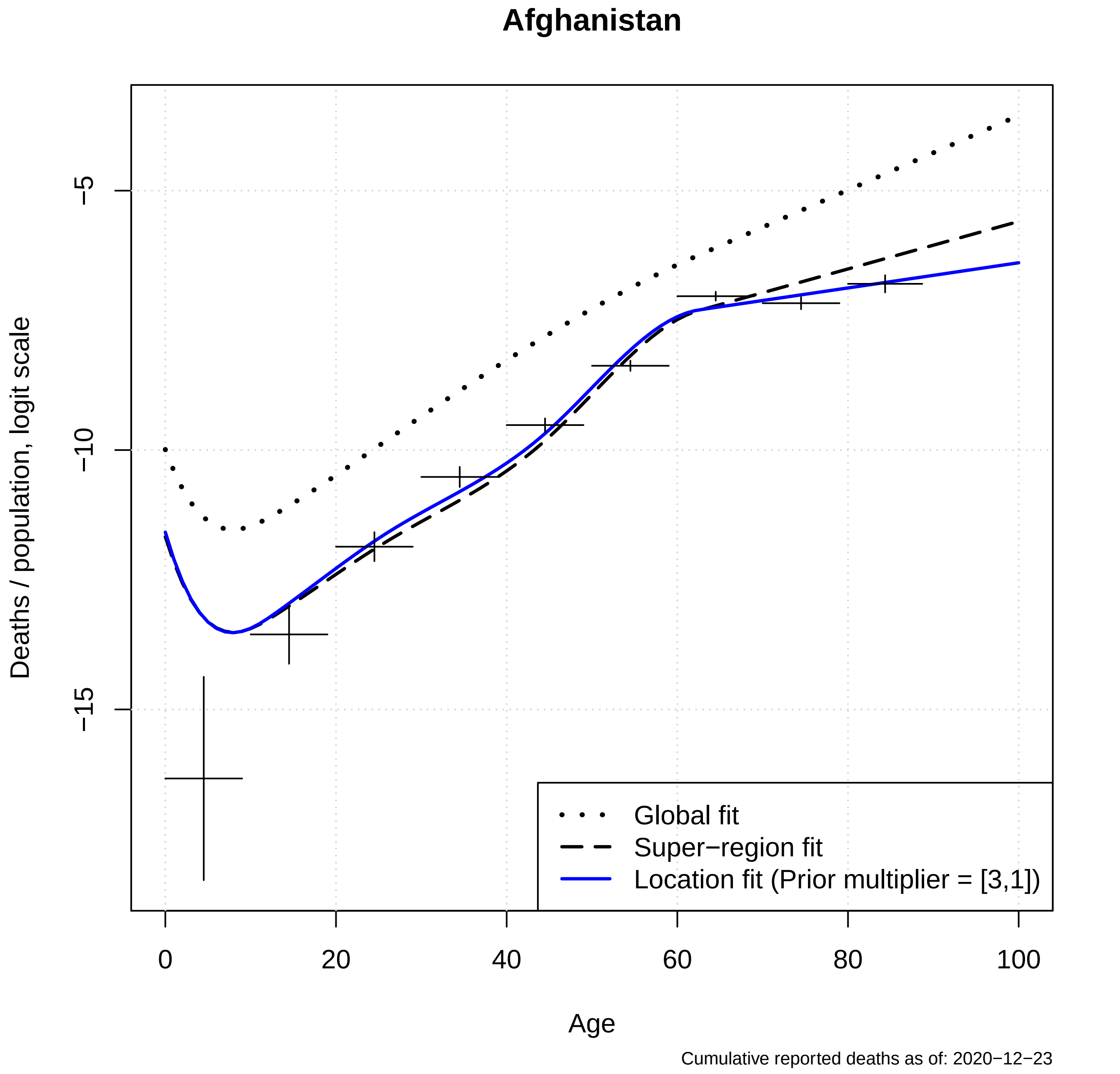
Afghanistan
Deaths / population, logit scale
−5
−10
−15
0
20
40
60
80
100
Age
Global fit
Super−region fit
Location fit (Prior multiplier = [3,1])
Cumulative reported deaths as of: 2020−12−23

Figure S6. Age patterns of COVID-19 mortality for select countries and territories in south Asia

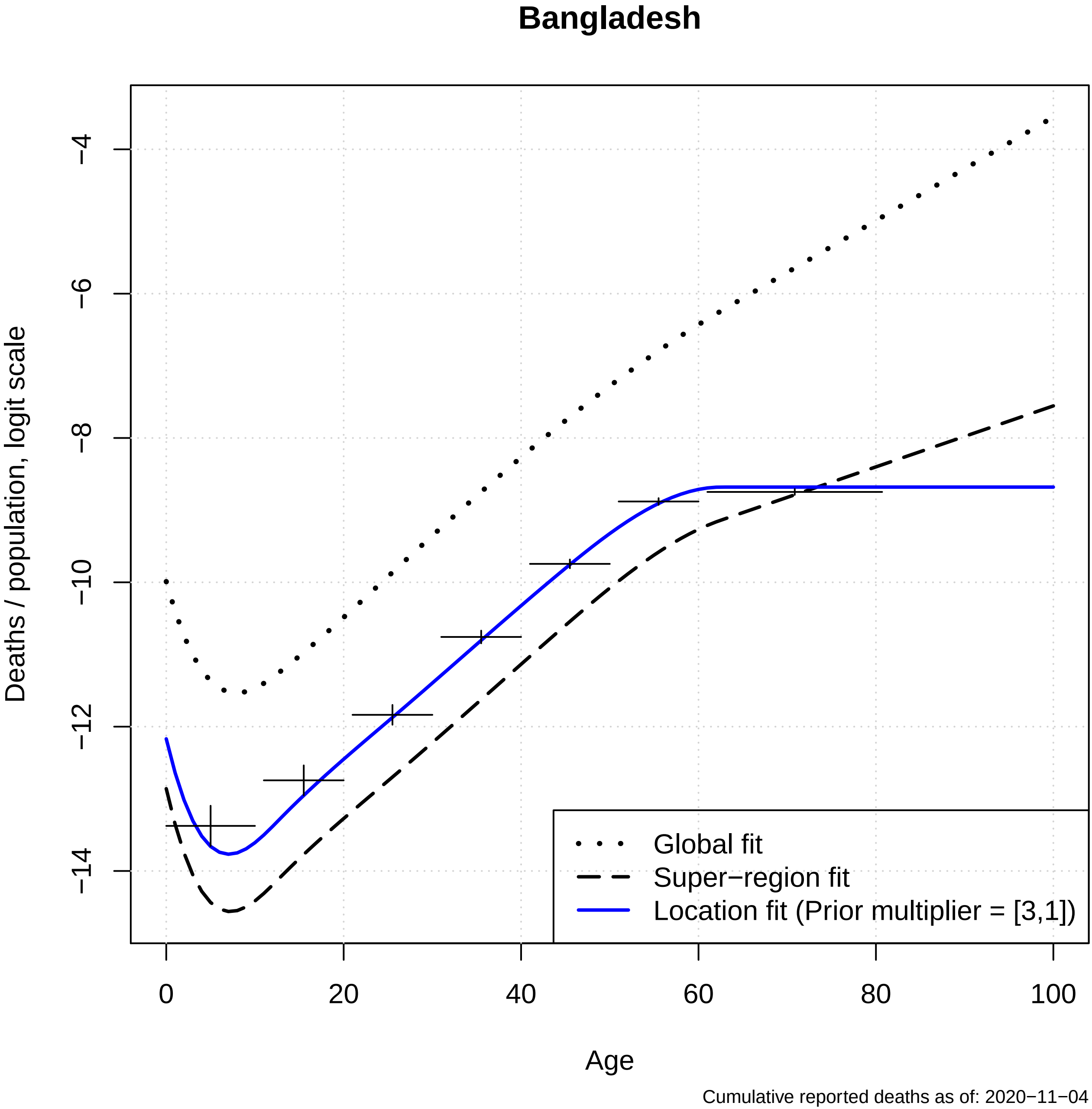

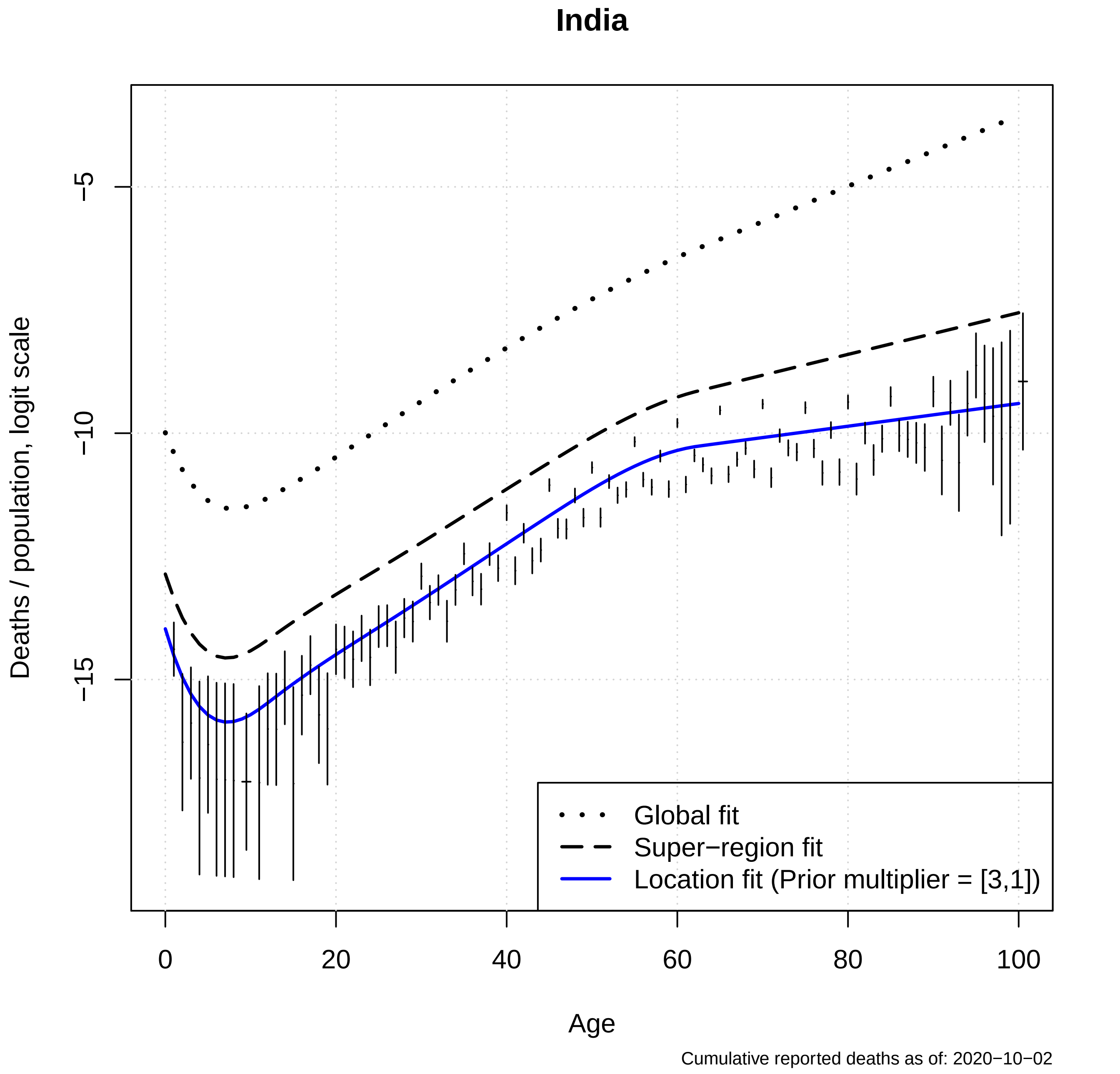

India
Deaths / population, logit scale
−5
−10
−15
0
20
40
60
80
100
Age
Global fit
Super−region fit
Location fit (Prior multiplier = [3,1])
Cumulative reported deaths as of: 2020−10−02

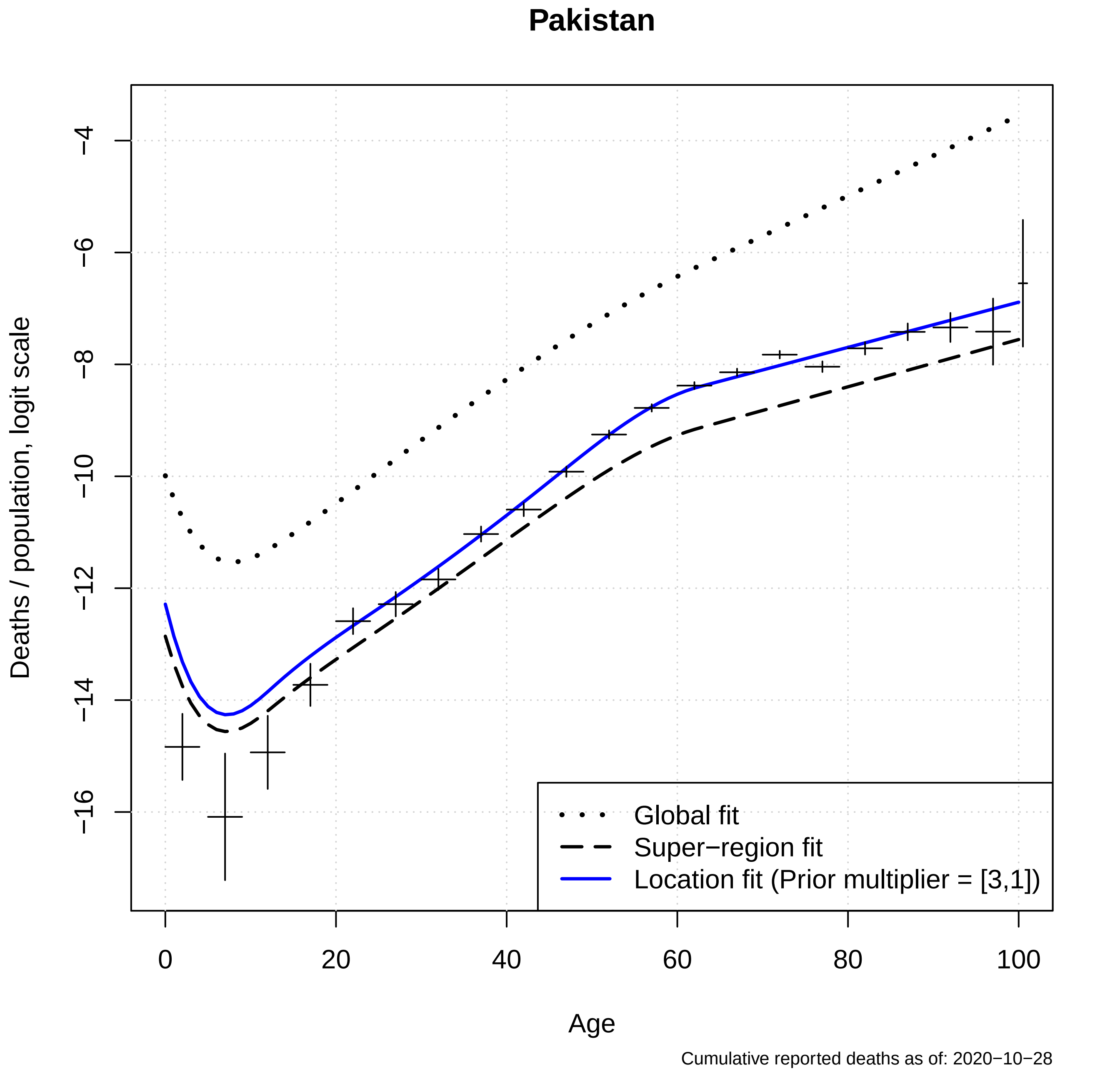
Pakistan
Deaths / population, logit scale
−4
−6
−8
−10
−12
−14
−16
0
20
40
60
80
100
Age
Global fit
Super−region fit
Location fit (Prior multiplier = [3,1])
Cumulative reported deaths as of: 2020−10−28

Figure S7. Age patterns of COVID-19 mortality for select countries and territories in sub-Saharan Africa

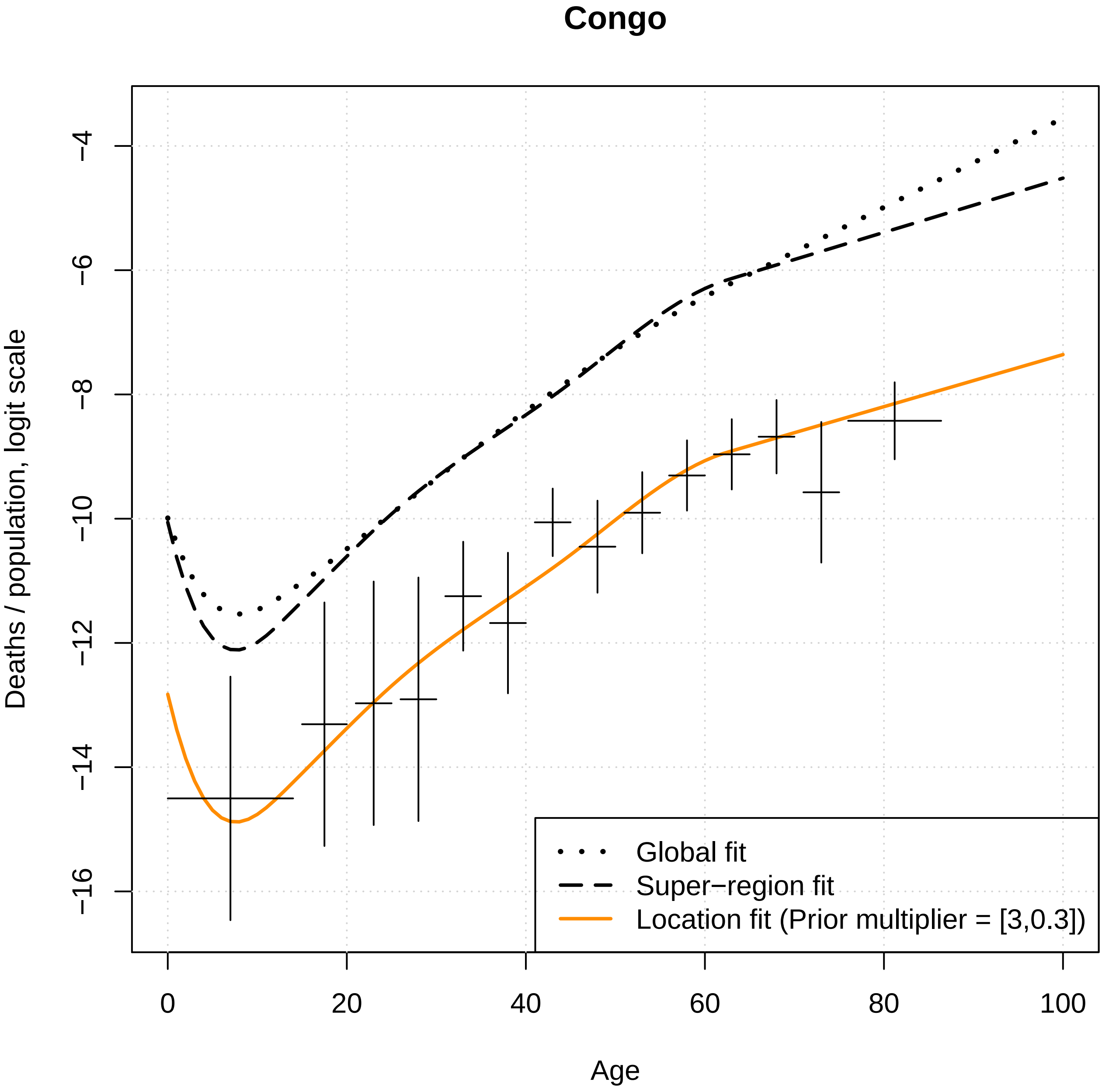

Cumulative reported deaths as of: 2020−09−21

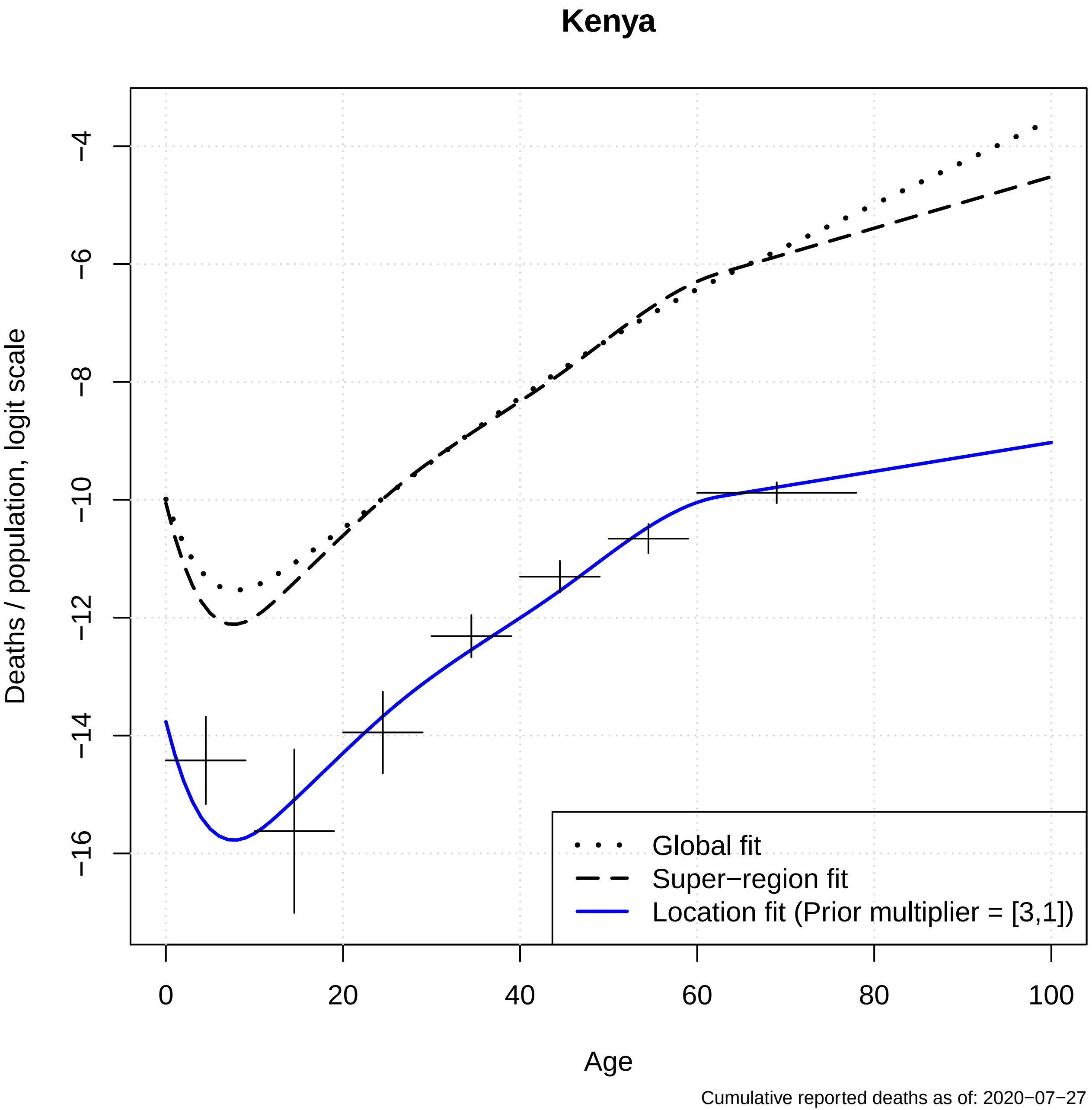
Kenya
Deaths / population, logit scale
−4
−6
−8
−10
−12
−14
−16
0
20
40
60
80
100
Age
Global fit
Super−region fit
Location fit (Prior multiplier = [3,1])
Cumulative reported deaths as of: 2020−07−27

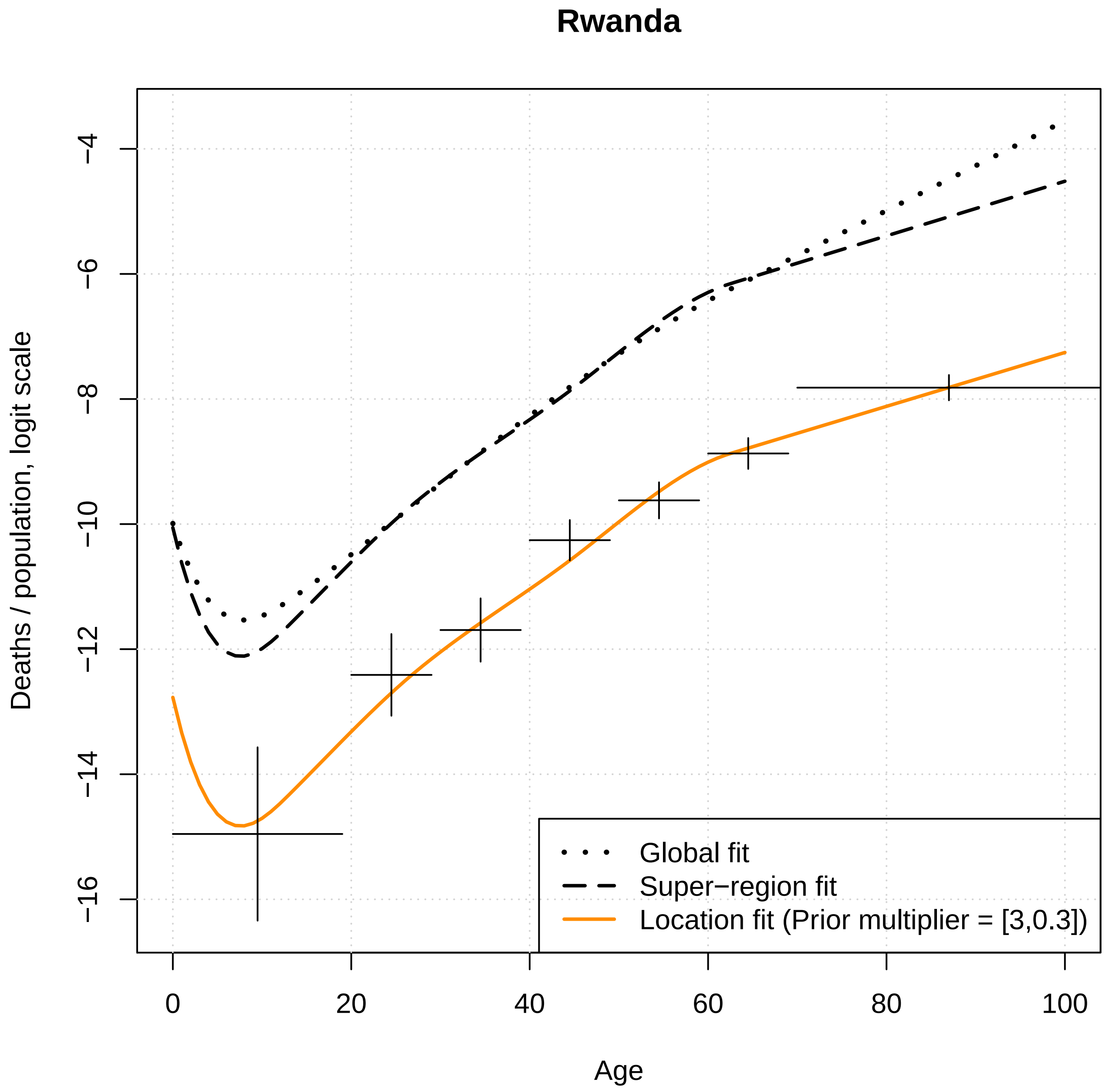

Cumulative reported deaths as of: 2021−03−04

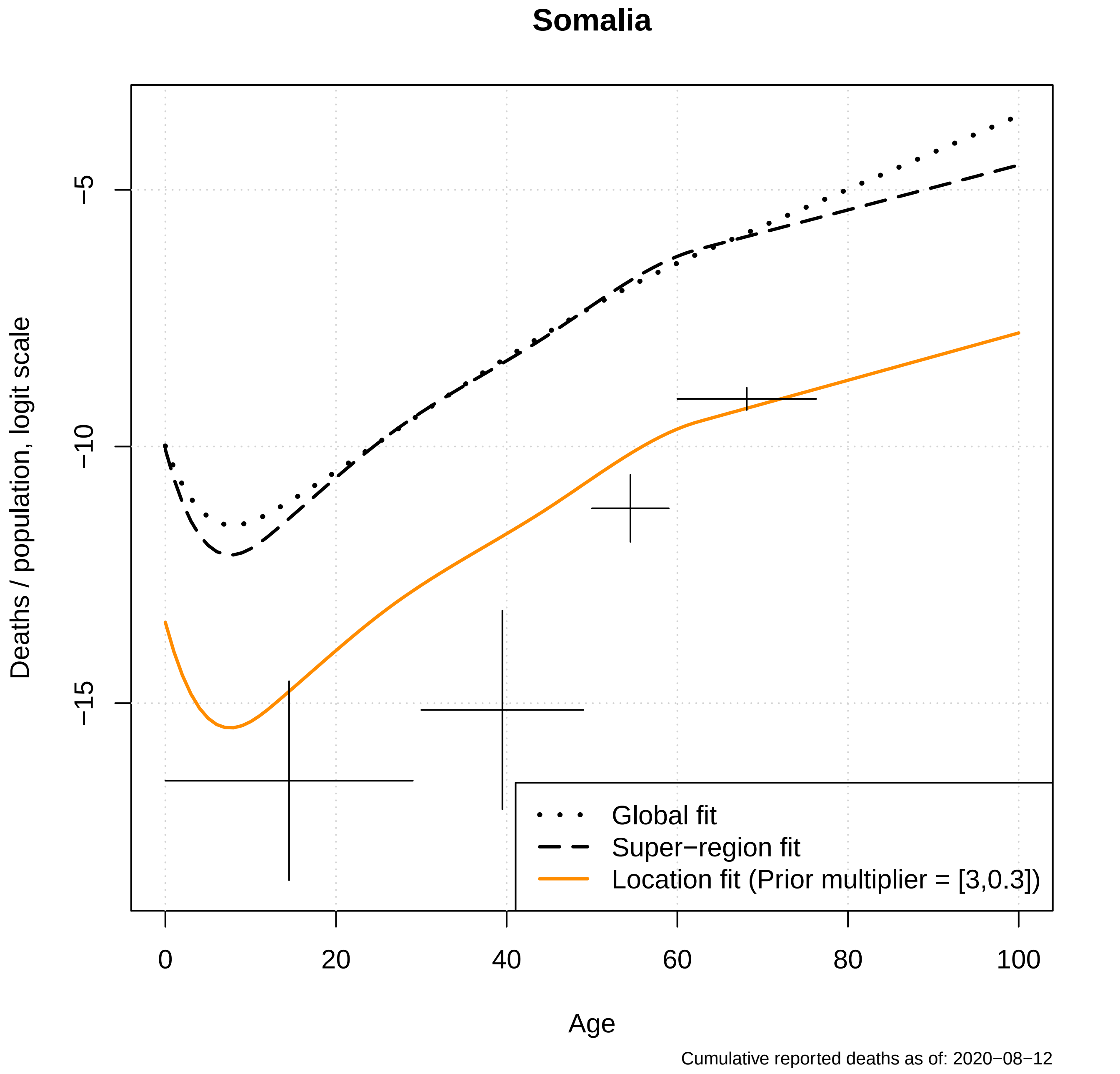
Somalia
Deaths / population, logit scale
−5
−10
−15
0
20
40
60
80
100
Age
Global fit
Super−region fit
Location fit (Prior multiplier = [3,0.3])
Cumulative reported deaths as of: 2020−08−12

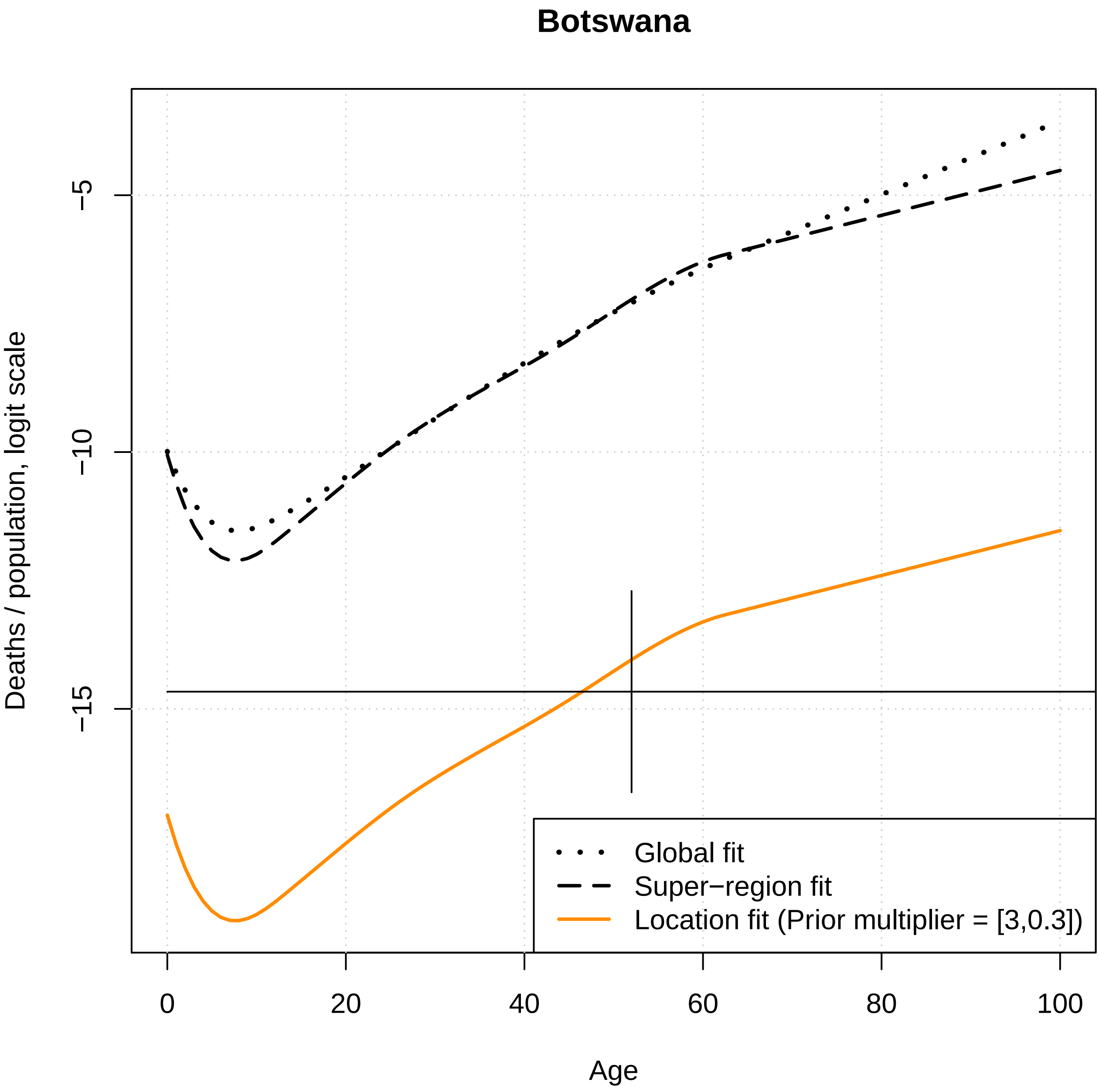

Cumulative reported deaths as of: 2020−06−17

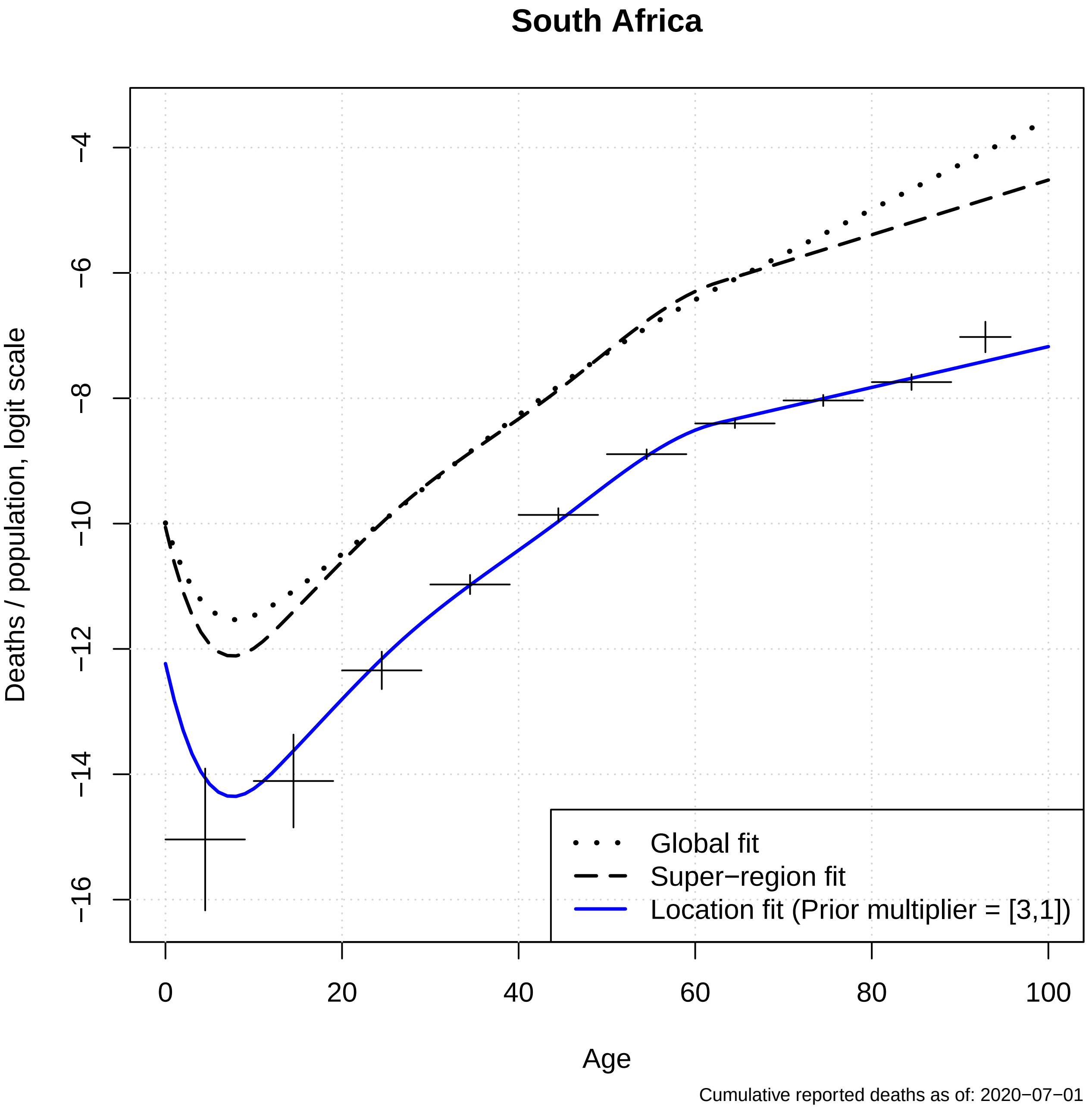

South Africa
Deaths / population, logit scale
−4
−6
−8
−10
−12
−14
−16
0
20
40
60
80
100
Age
Global fit
Super−region fit
Location fit (Prior multiplier = [3,1])
Cumulative reported deaths as of: 2020−07−01

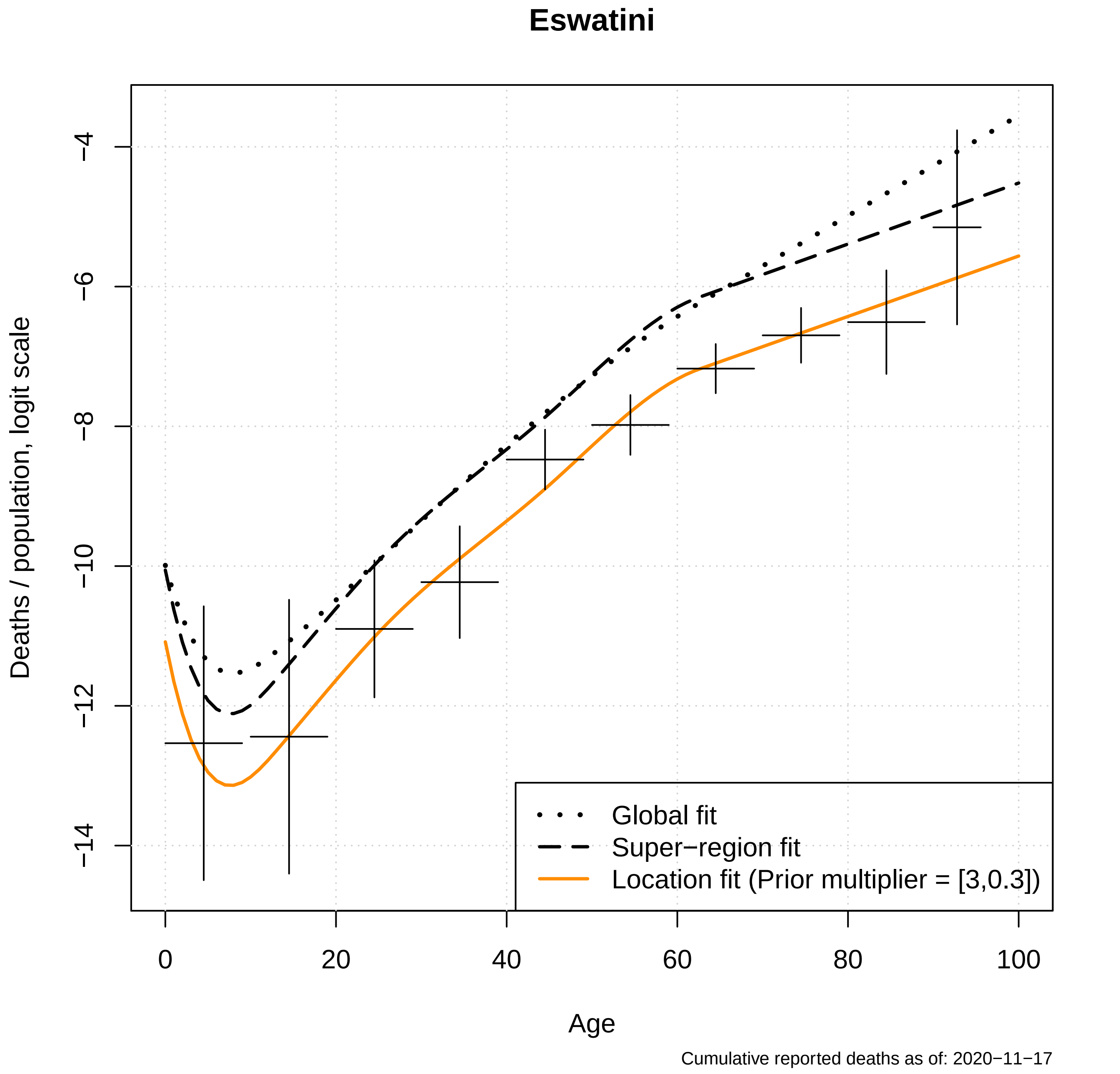

Eswatini
Deaths / population, logit scale
−4
−6
−8
−10
−12
−14
0
20
40
60
80
100
Age
Global fit
Super−region fit
Location fit (Prior multiplier = [3,0.3])
Cumulative reported deaths as of: 2020−11−17

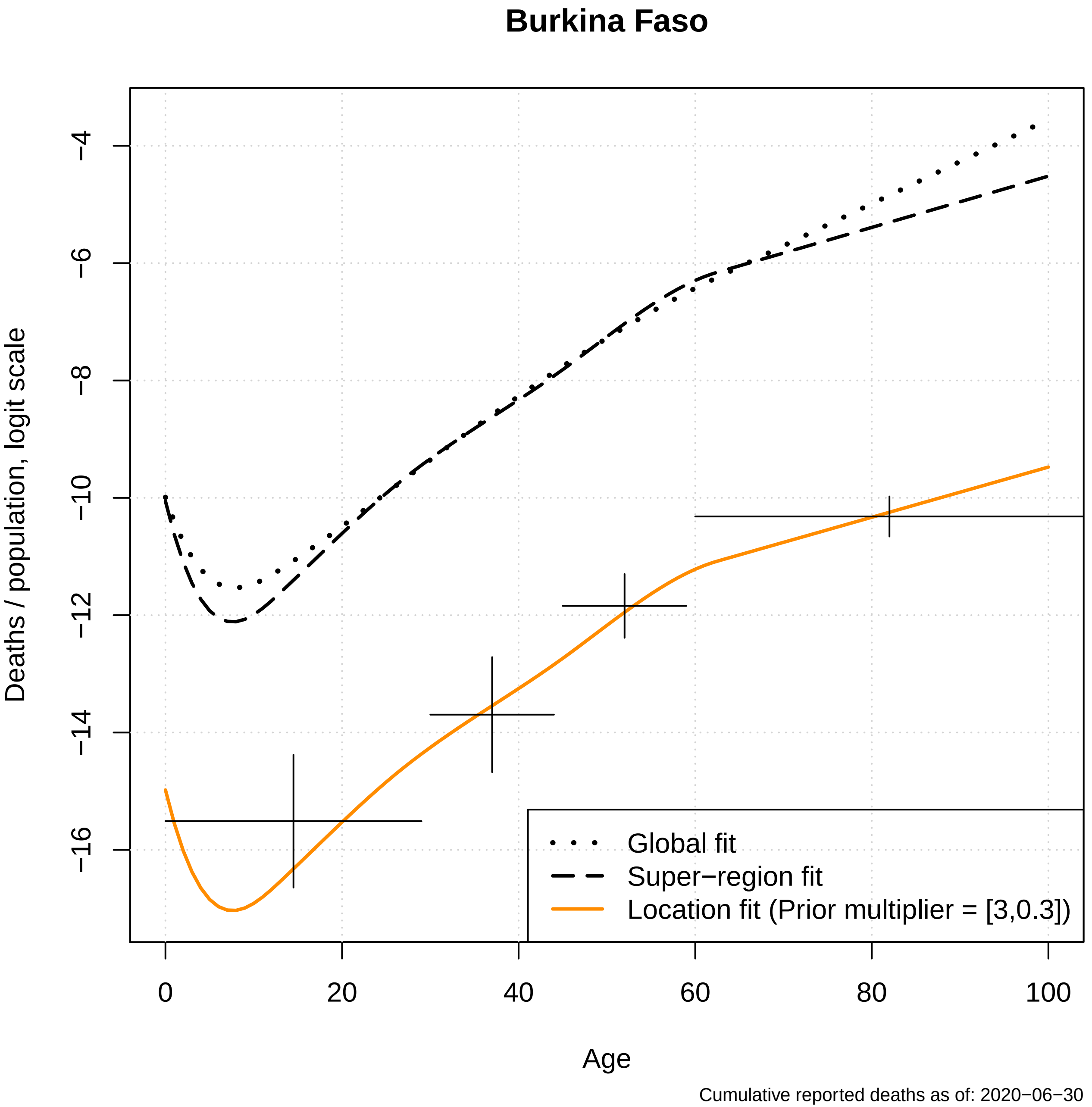

Burkina Faso
Deaths / population, logit scale
Age
−4
−6
−8
−10
−12
−14
−16
0
20
40
60
80
100
Global fit
Super−region fit
Location fit (Prior multiplier = [3,0.3])
Cumulative reported deaths as of: 2020−06−30

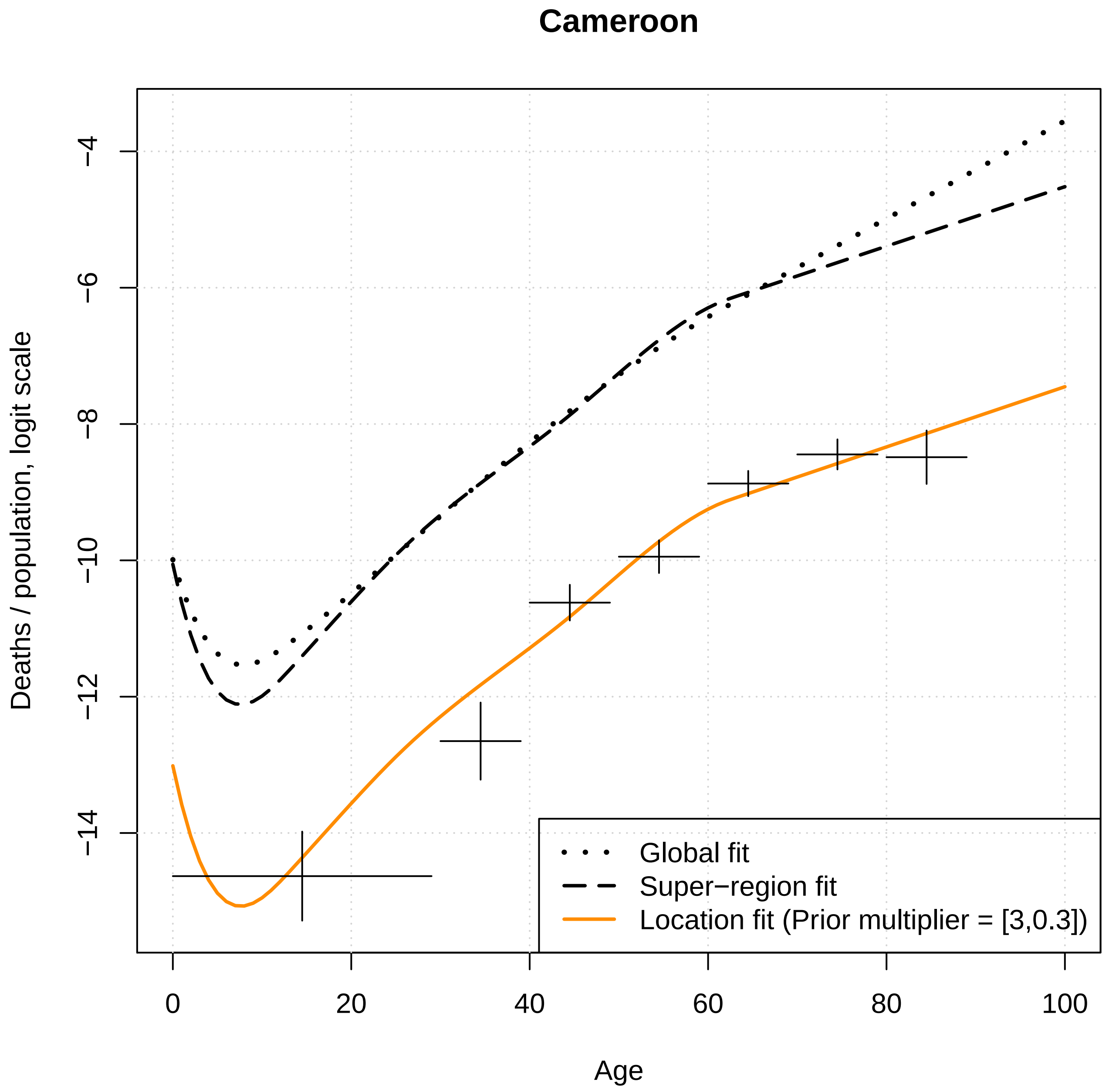

Cumulative reported deaths as of: 2020−09−23

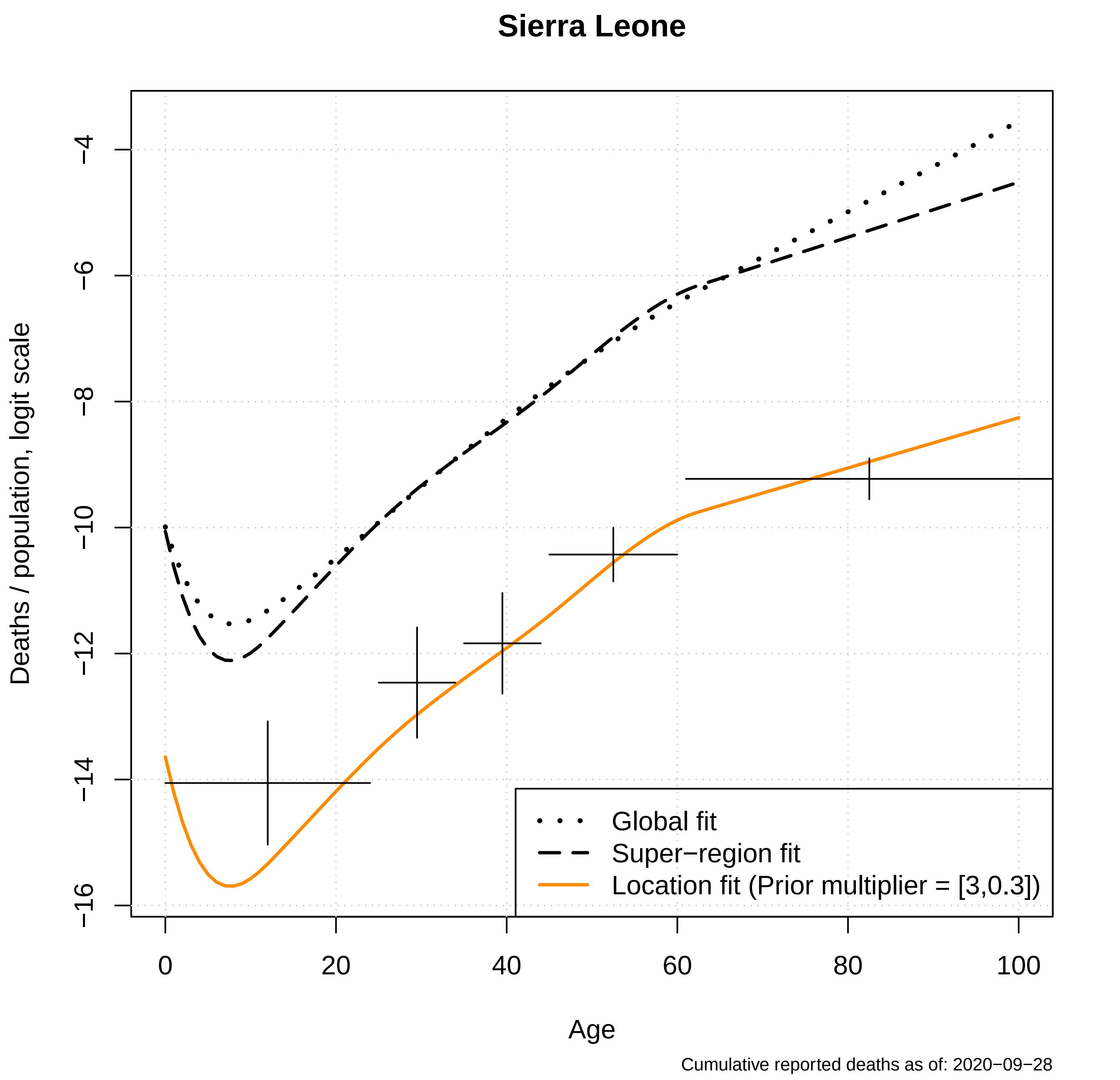

Sierra Leone
Deaths / population, logit scale
−4
−6
−8
−10
−12
−14
−16
0
20
40
60
80
100
Age
Global fit
Super−region fit
Location fit (Prior multiplier = [3,0.3])
Cumulative reported deaths as of: 2020−09−28

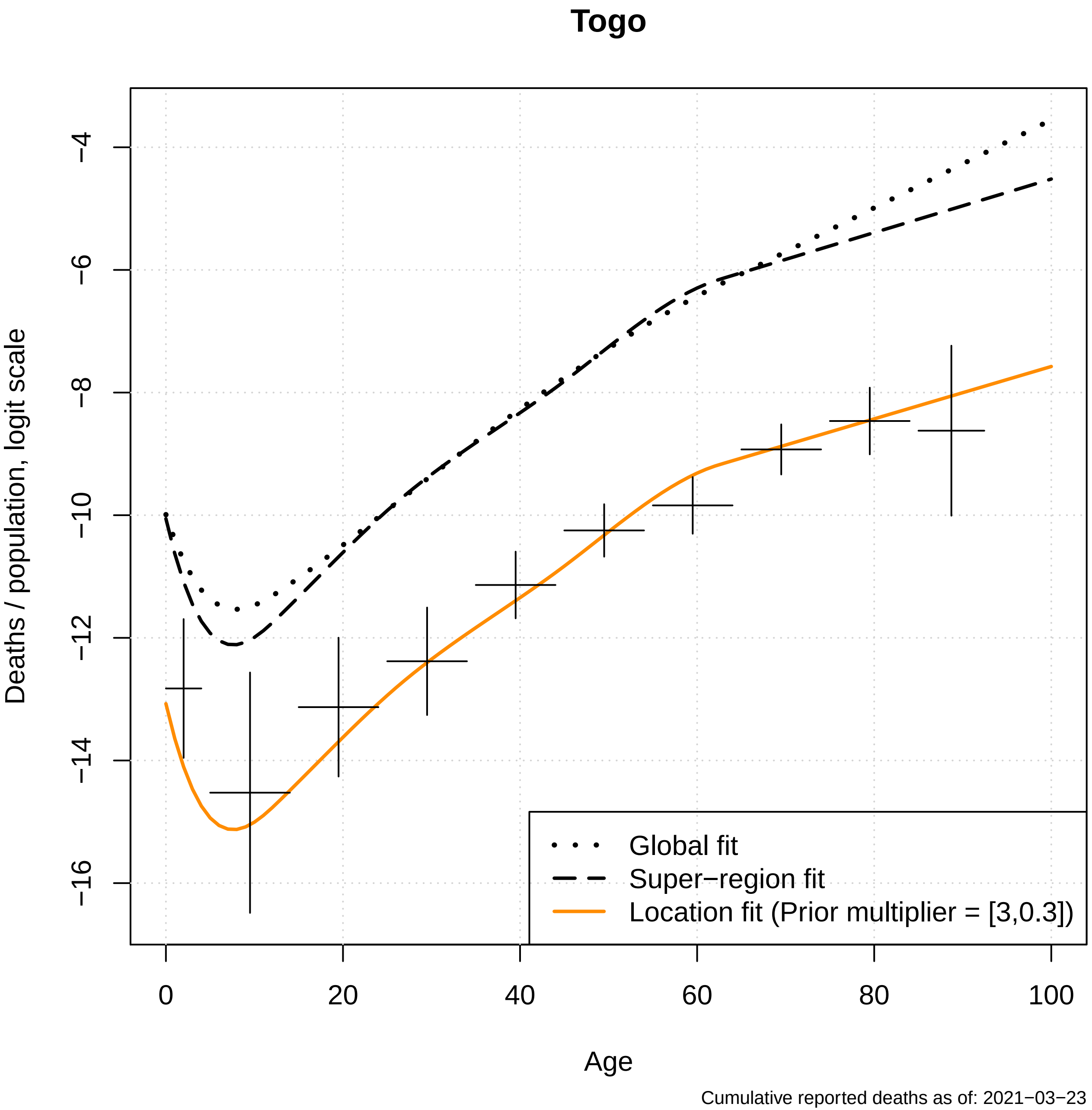

Togo
Deaths / population, logit scale
−4
−6
−8
−10
−12
−14
−16
0
20
40
60
80
100
Age
Global fit
Super−region fit
Location fit (Prior multiplier = [3,0.3])
Cumulative reported deaths as of: 2021−03−23

Figure S8. Age patterns of COVID-19 mortality for select subnational locations

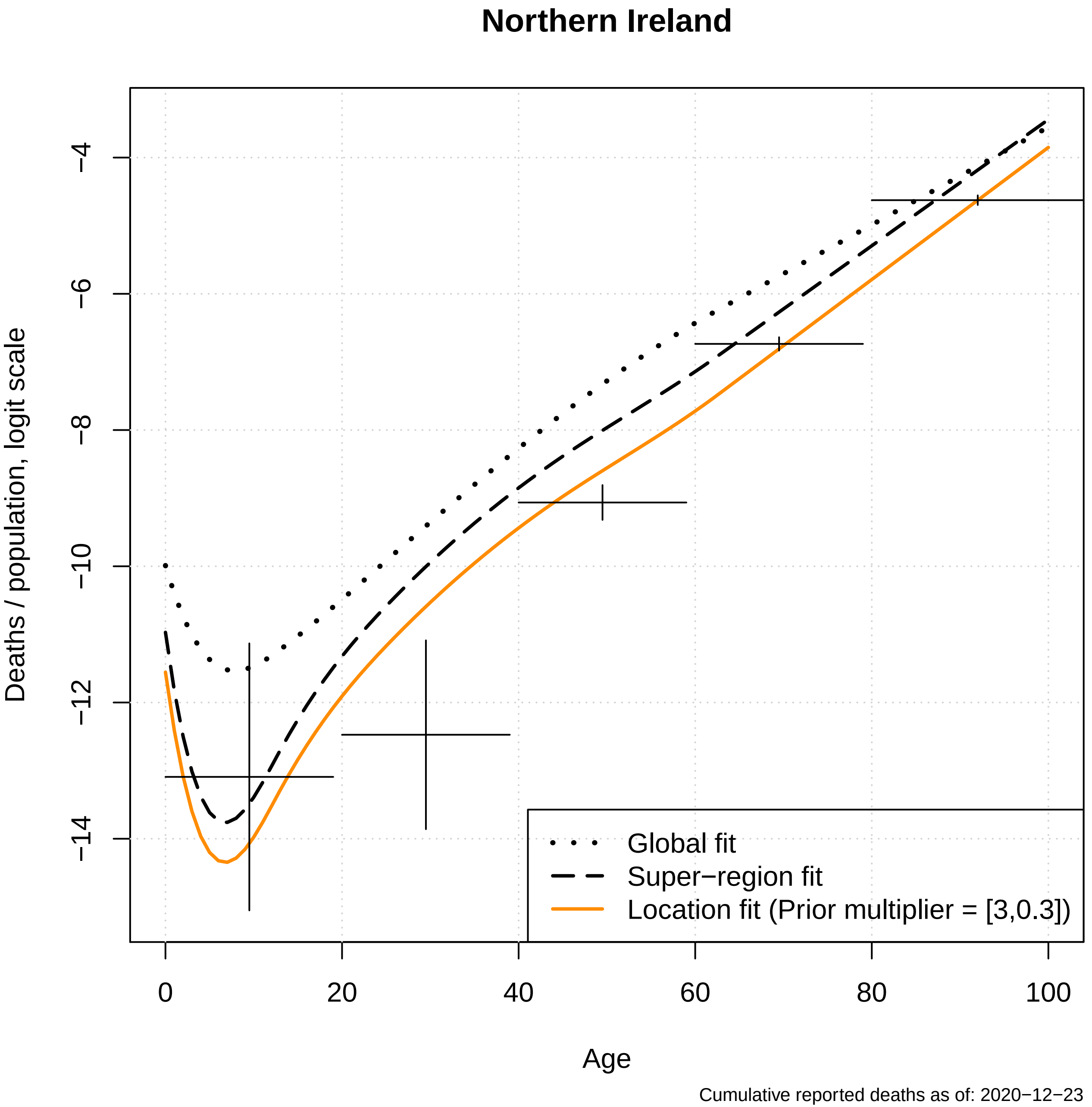

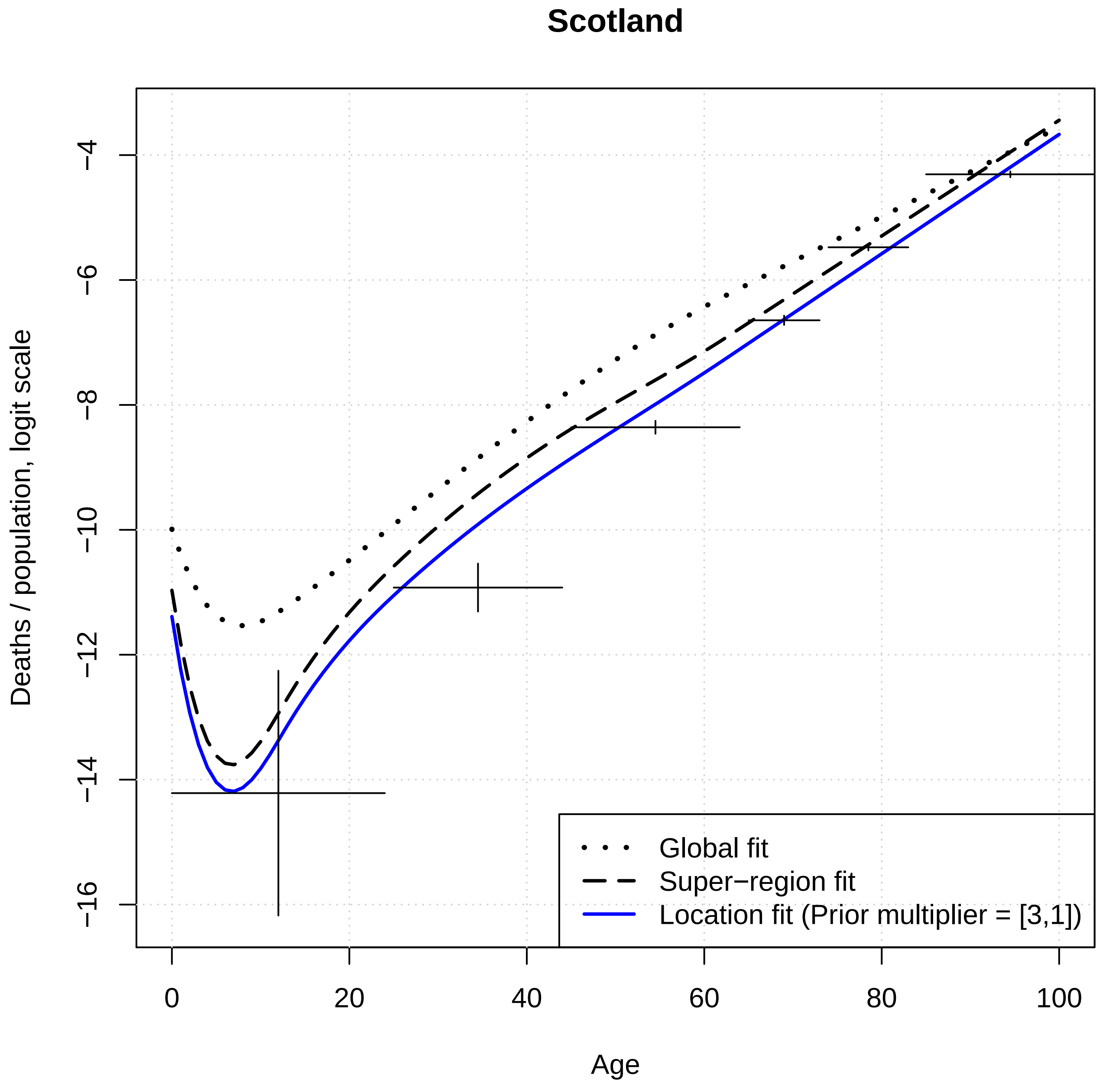

Cumulative reported deaths as of: 2020−12−21

Figure S8B. South Africa subnationals

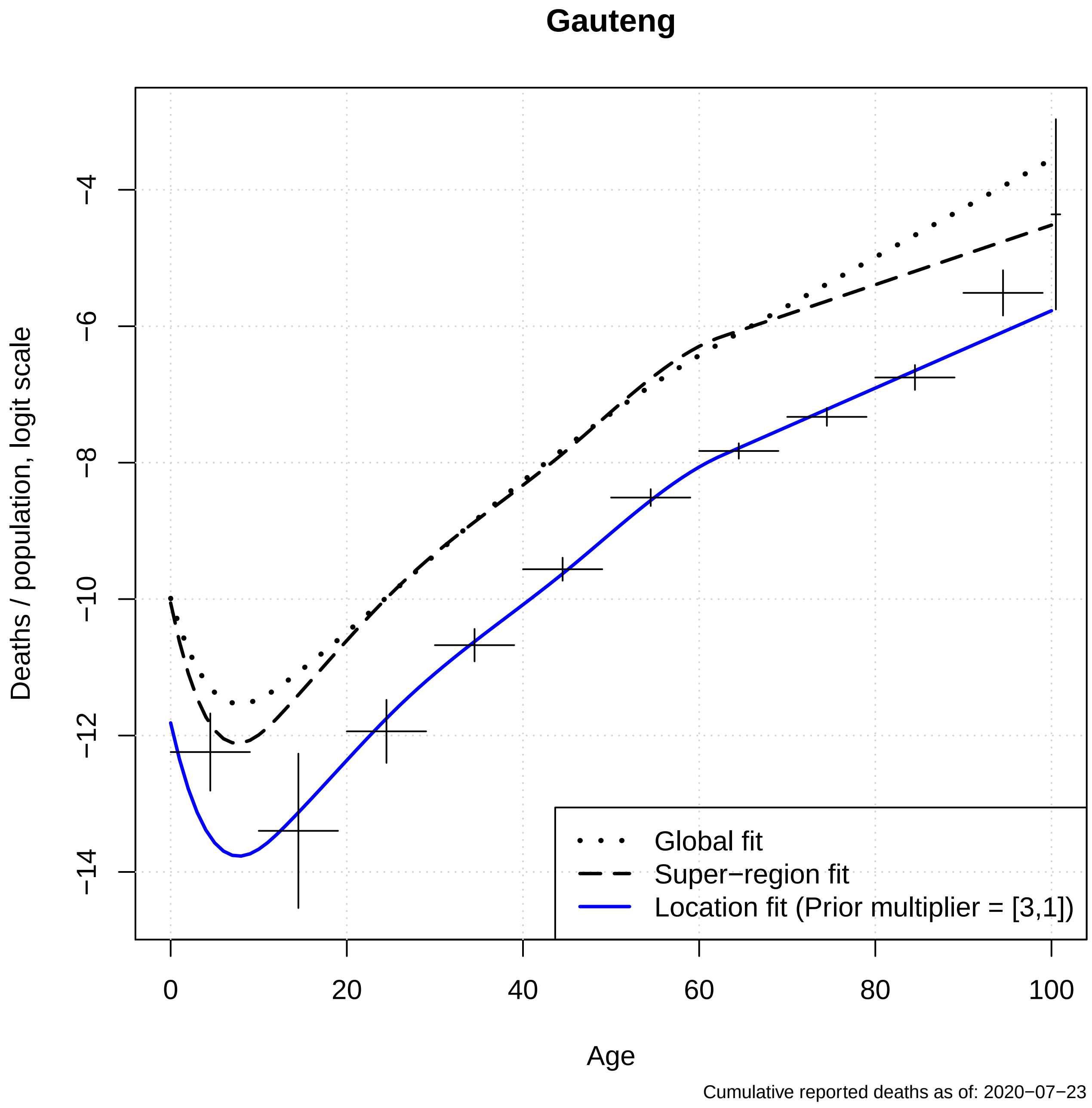

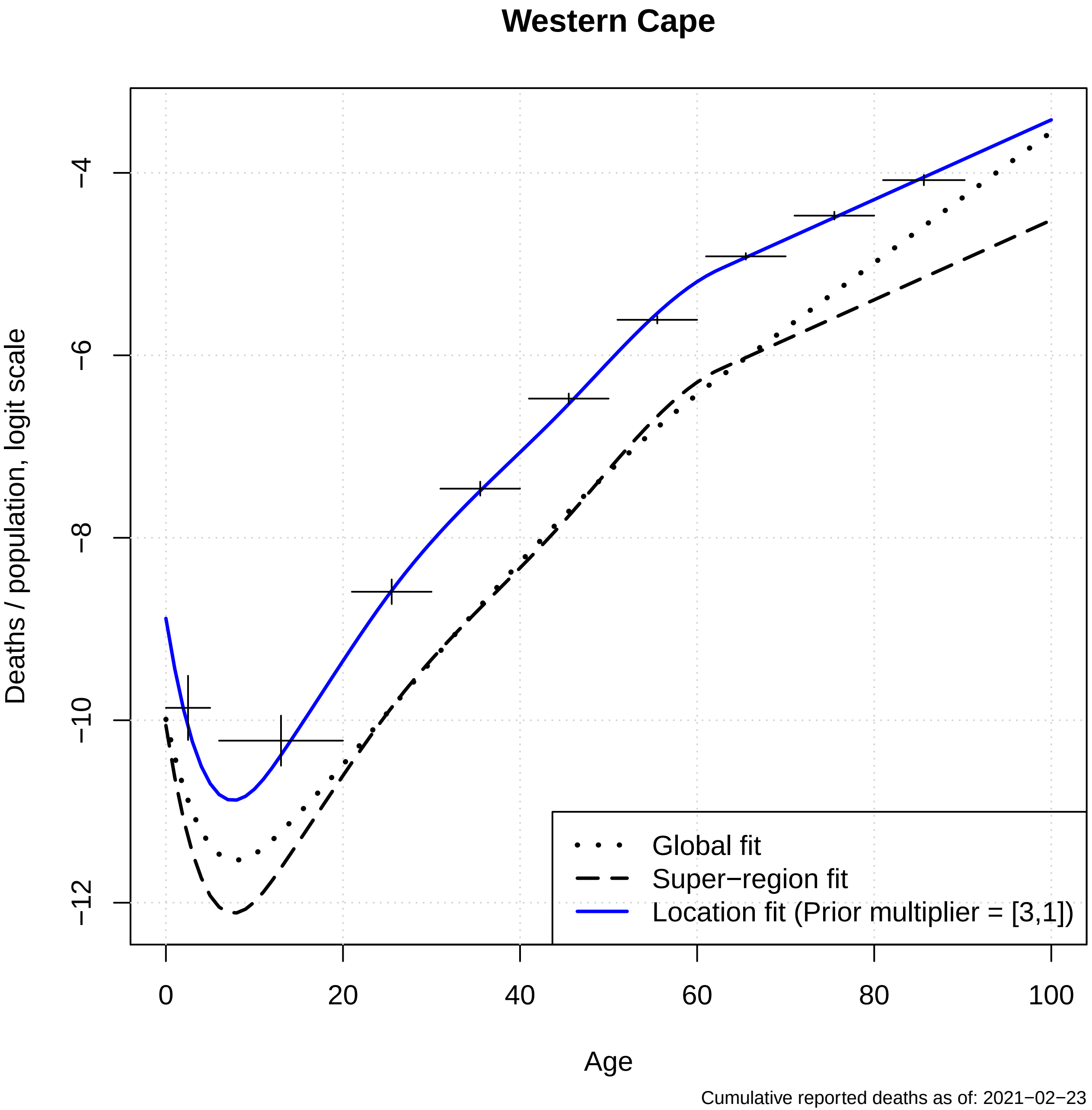

Western Cape
Deaths / population, logit scale
−4
−6
−8
−10
−12
0
20
40
60
80
100
Age
Global fit
Super−region fit
Location fit (Prior multiplier = [3,1])
Cumulative reported deaths as of: 2021−02−23

Figure S8C. USA subnationals

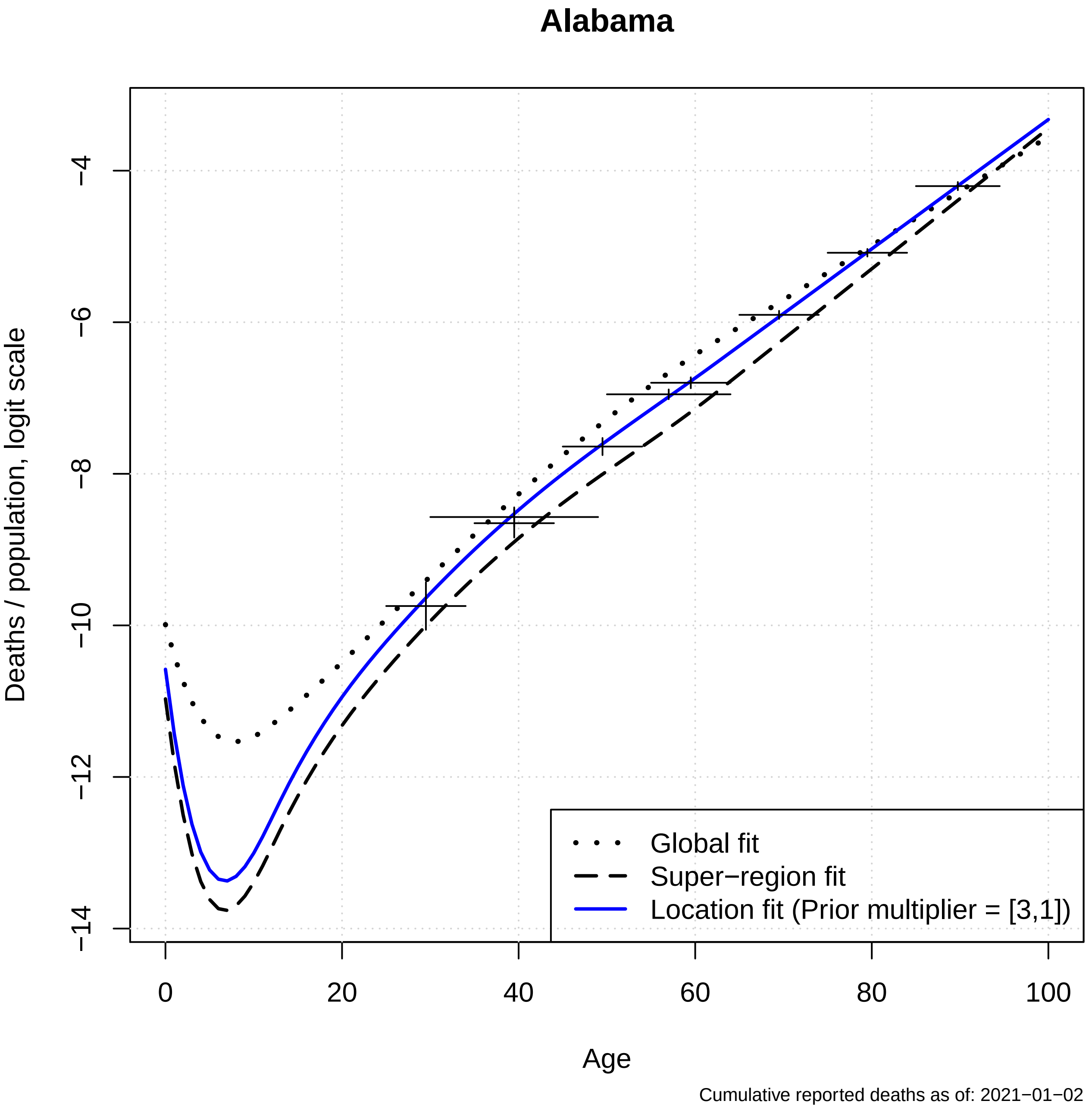

Alaska

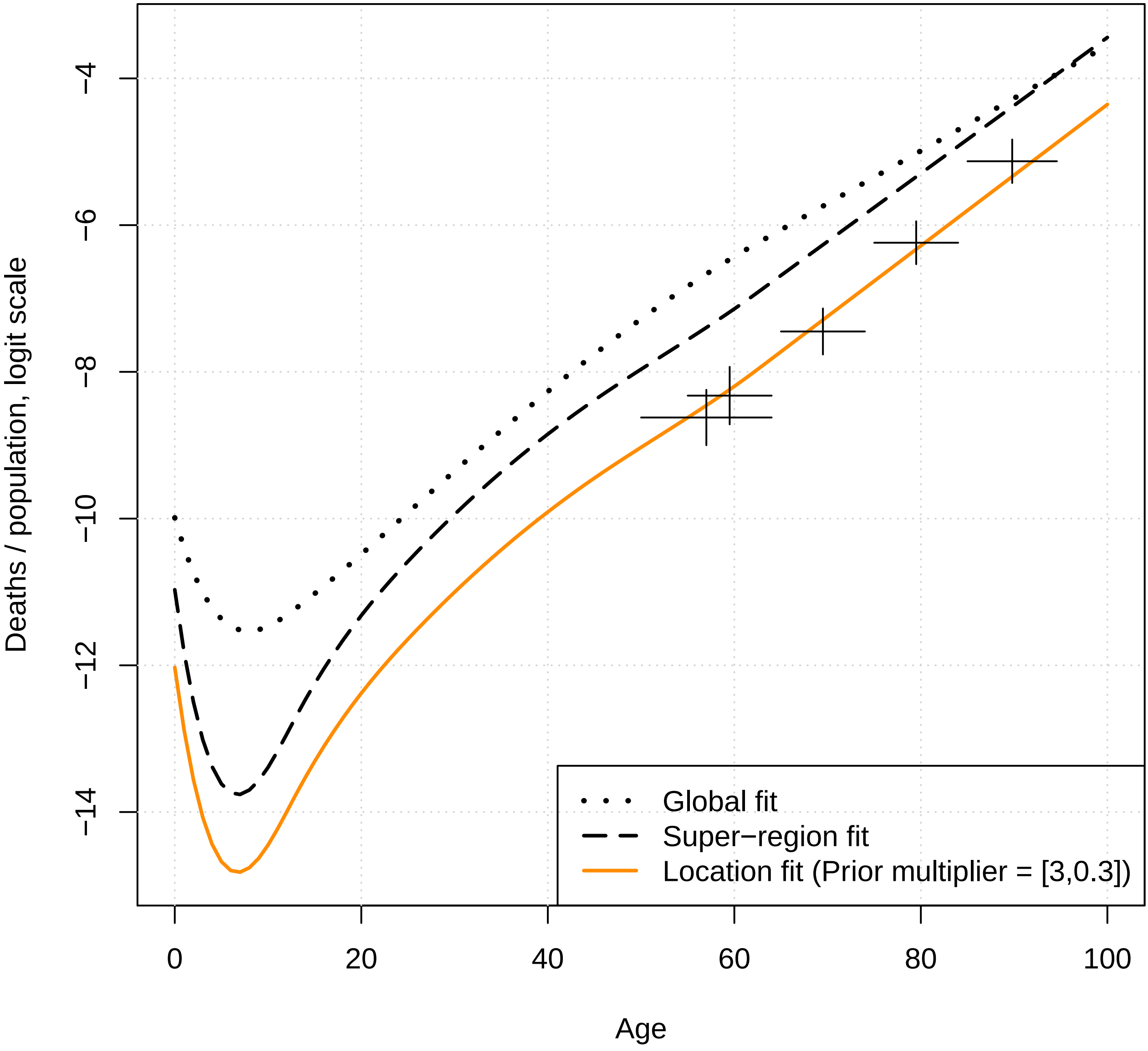

Cumulative reported deaths as of: 2021−01−02

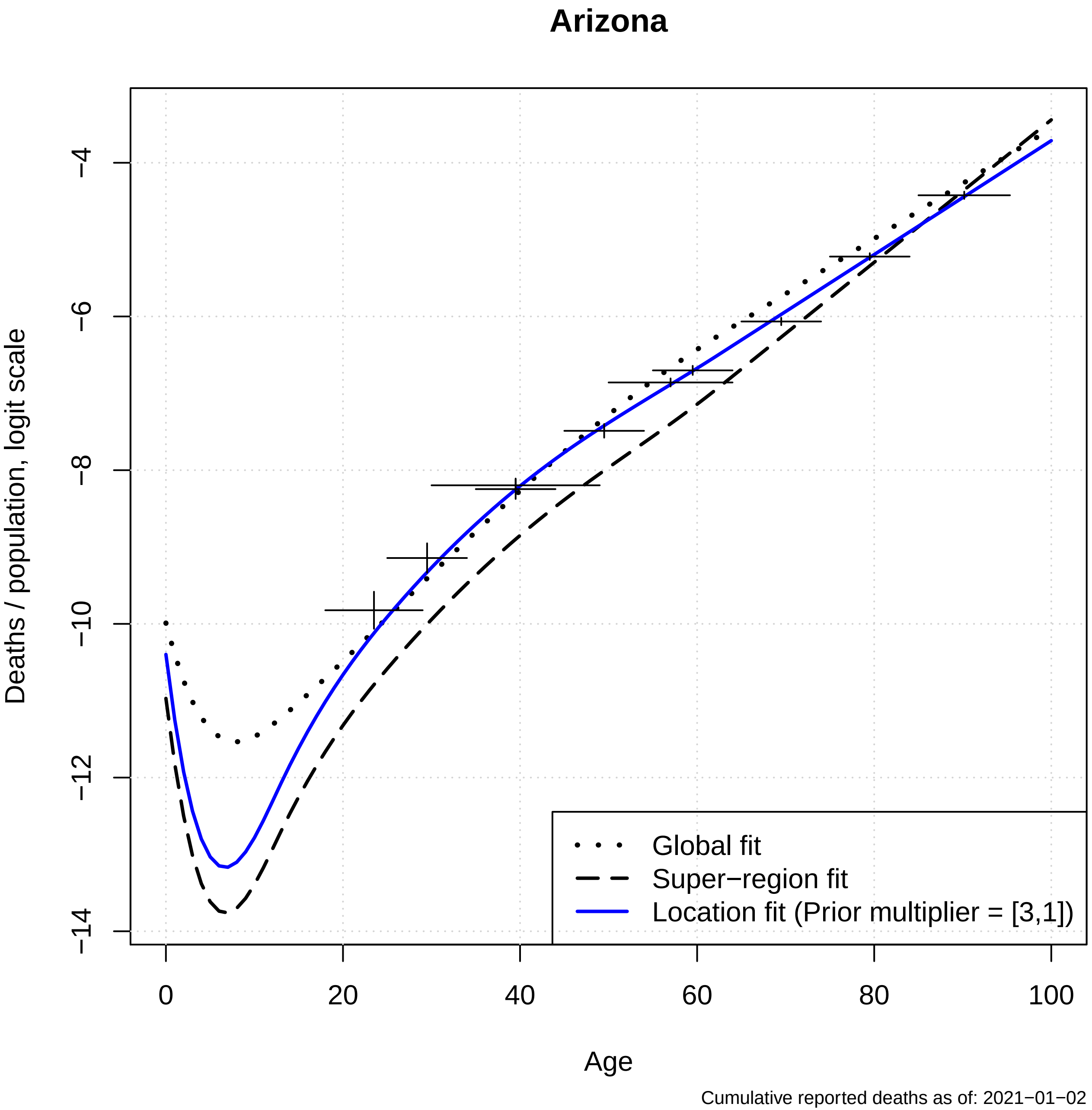

Arizona
Deaths / population, logit scale
−4
−6
−8
−10
−12
−14
0
20
40
60
80
100
Age
Global fit
Super−region fit
Location fit (Prior multiplier = [3,1])
Cumulative reported deaths as of: 2021−01−02

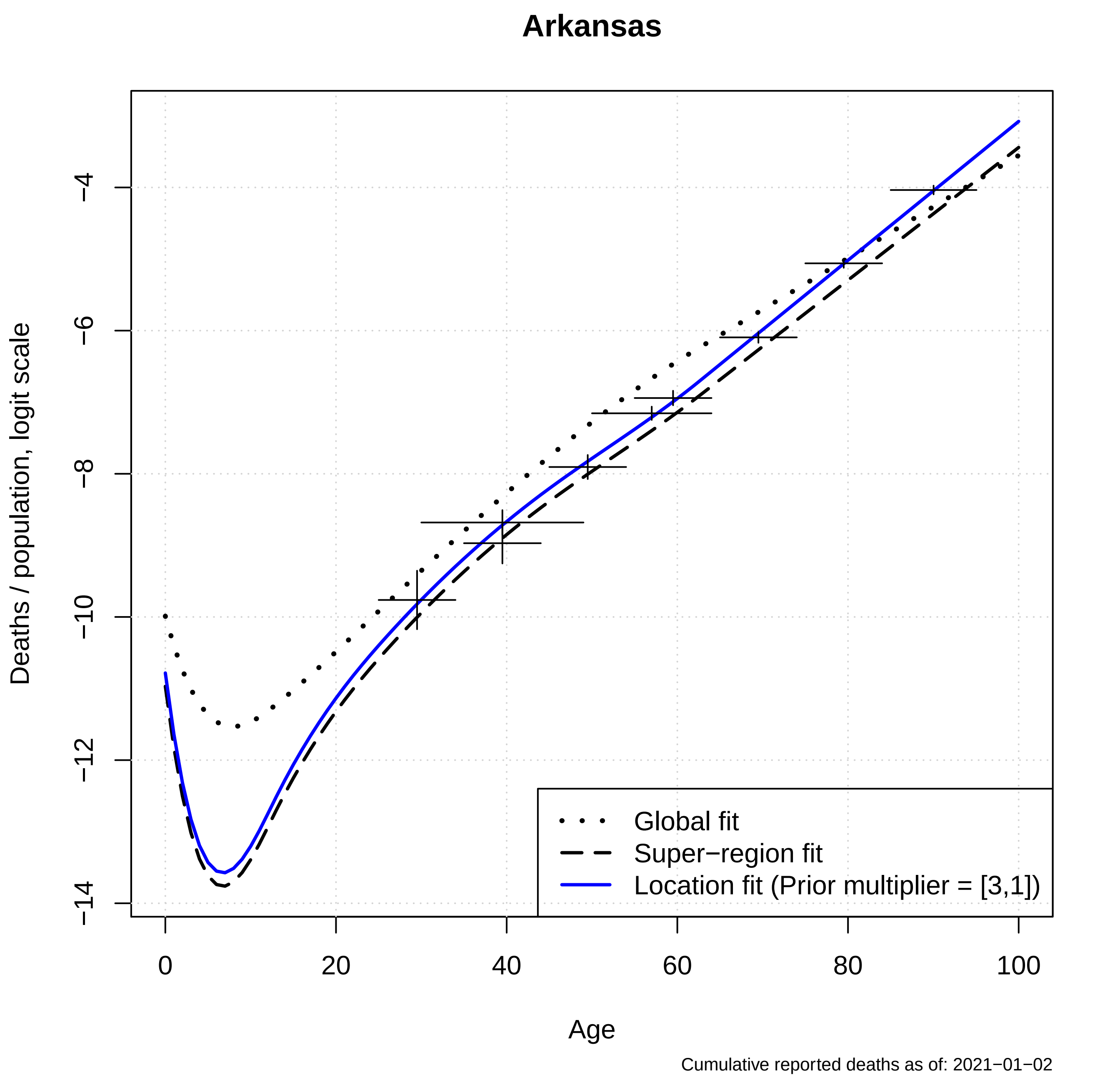
Arkansas
Deaths / population, logit scale
−4
−6
−8
−10
−12
−14
0
20
40
60
80
100
Age
Global fit
Super−region fit
Location fit (Prior multiplier = [3,1])
Cumulative reported deaths as of: 2021−01−02

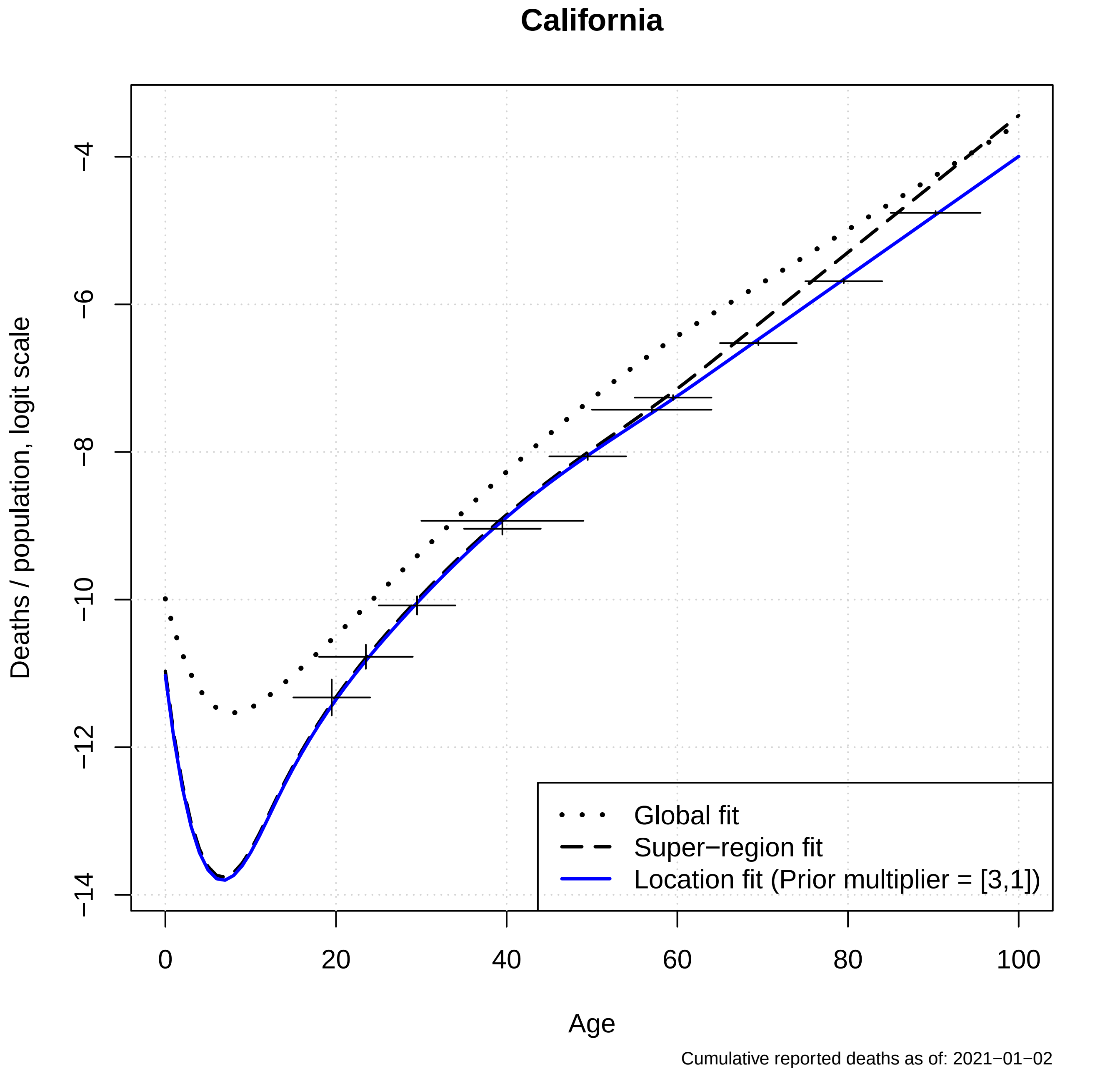
California
Deaths / population, logit scale
−4
−6
−8
−10
−12
−14
0
20
40
60
80
100
Age
Global fit
Super−region fit
Location fit (Prior multiplier = [3,1])
Cumulative reported deaths as of: 2021−01−02

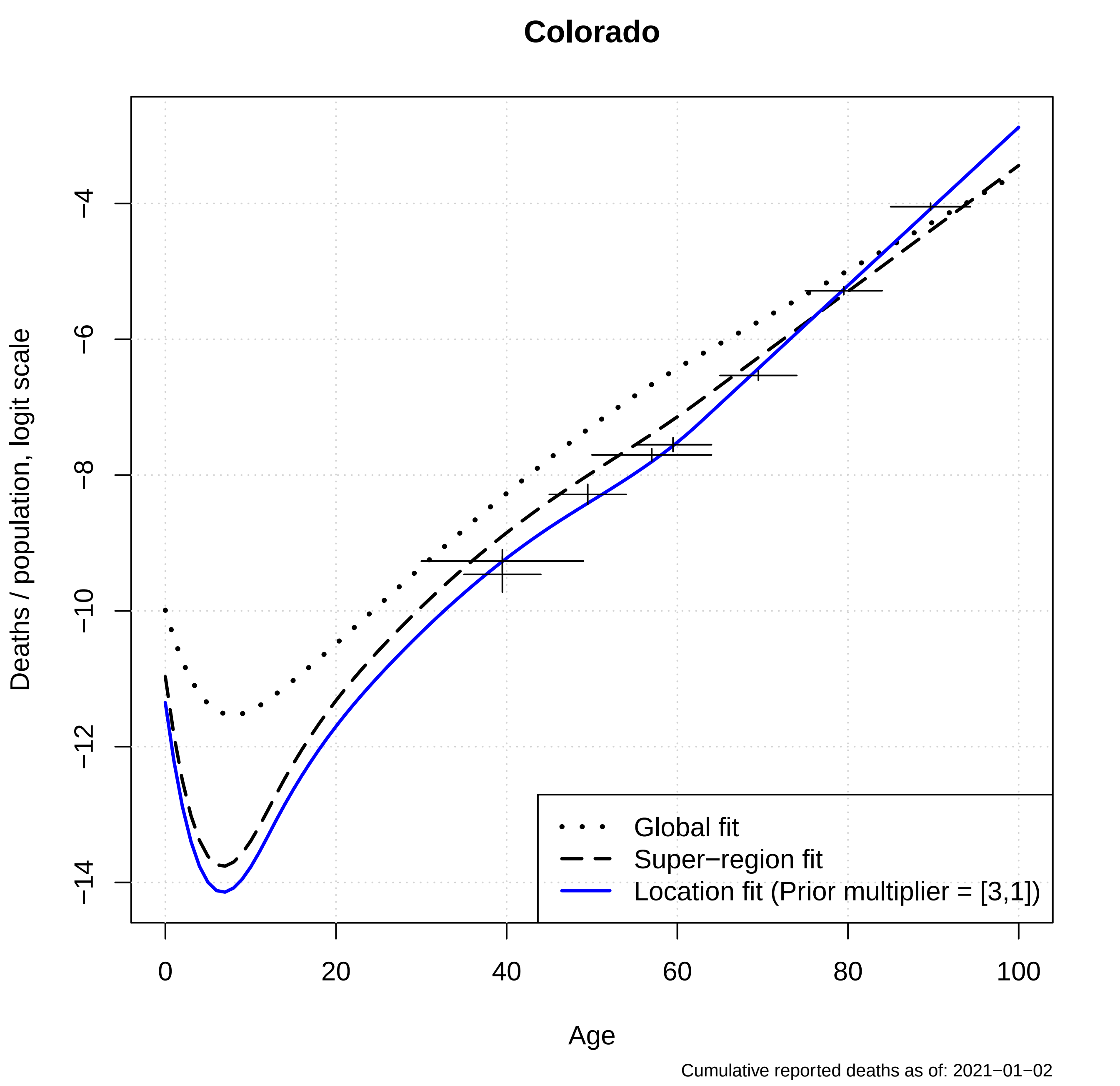

Colorado
Deaths / population, logit scale
−4
−6
−8
−10
−12
−14
0
20
40
60
80
100
Age
Global fit
Super−region fit
Location fit (Prior multiplier = [3,1])
Cumulative reported deaths as of: 2021−01−02

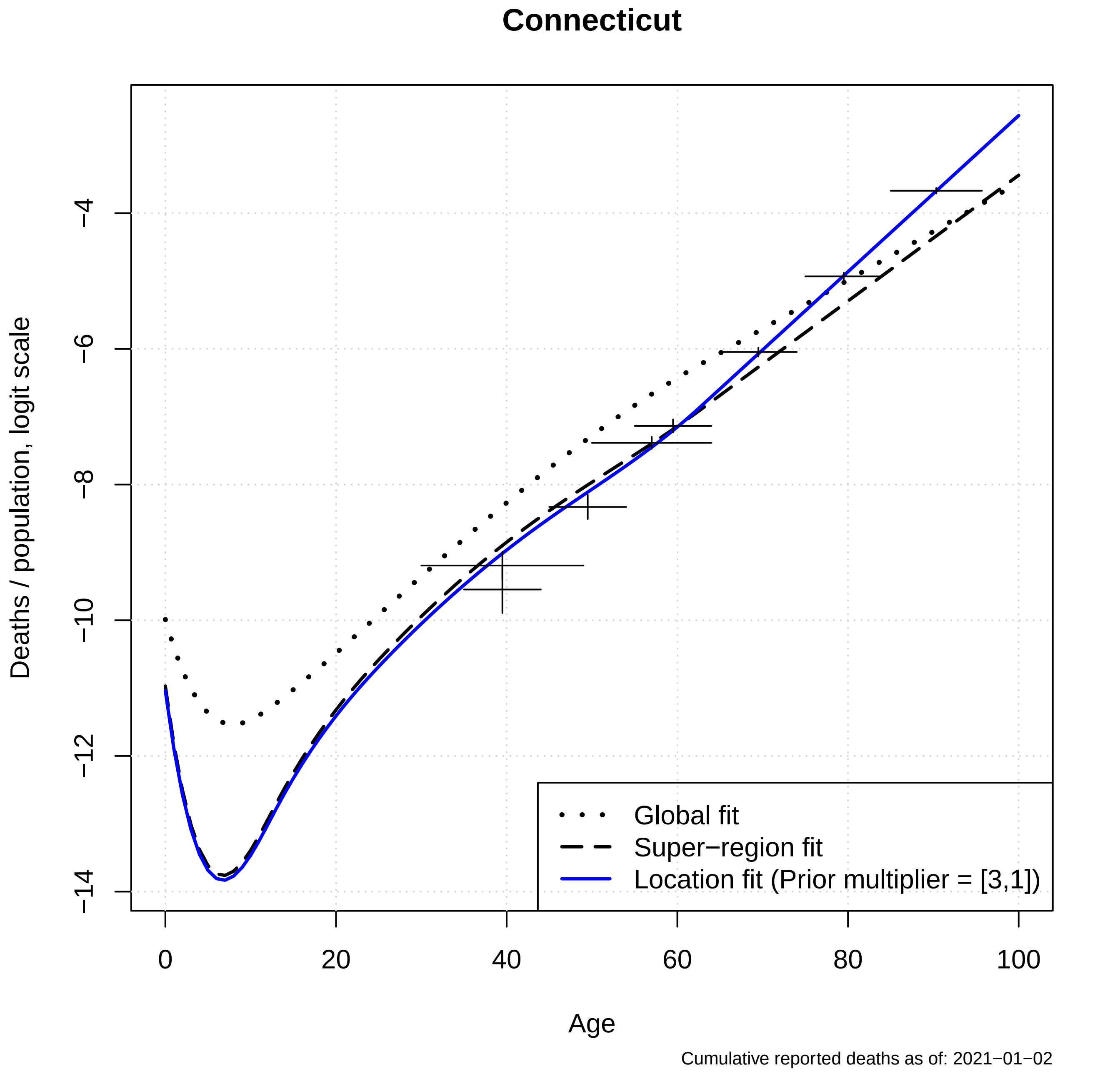

Connecticut
Deaths / population, logit scale
−4
−6
−8
−10
−12
−14
0
20
40
60
80
100
Age
Global fit
Super−region fit
Location fit (Prior multiplier = [3,1])
Cumulative reported deaths as of: 2021−01−02

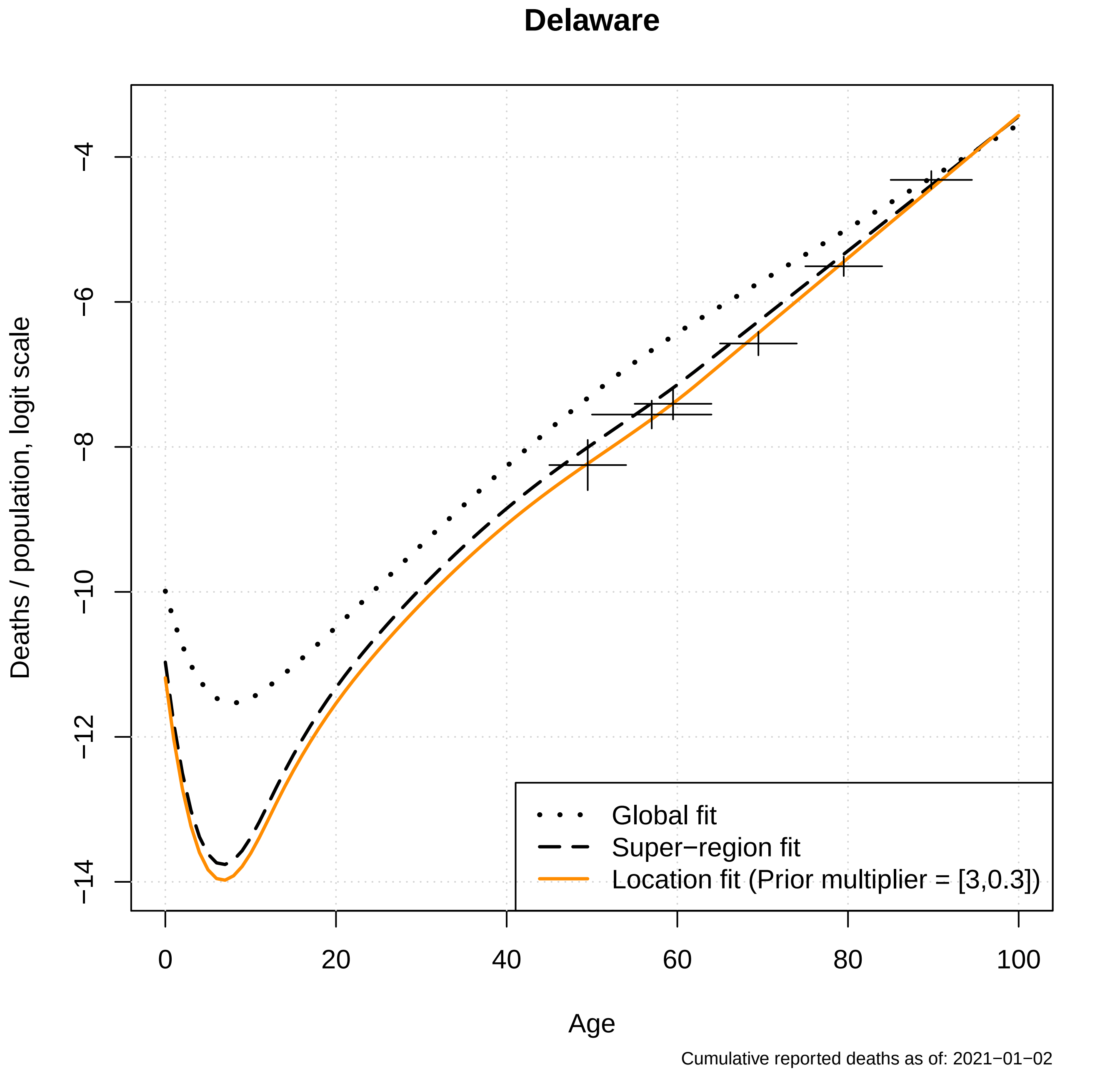

Delaware
Deaths / population, logit scale
−4
−6
−8
−10
−12
−14
0
20
40
60
80
100
Age
Global fit
Super−region fit
Location fit (Prior multiplier = [3,0.3])
Cumulative reported deaths as of: 2021−01−02

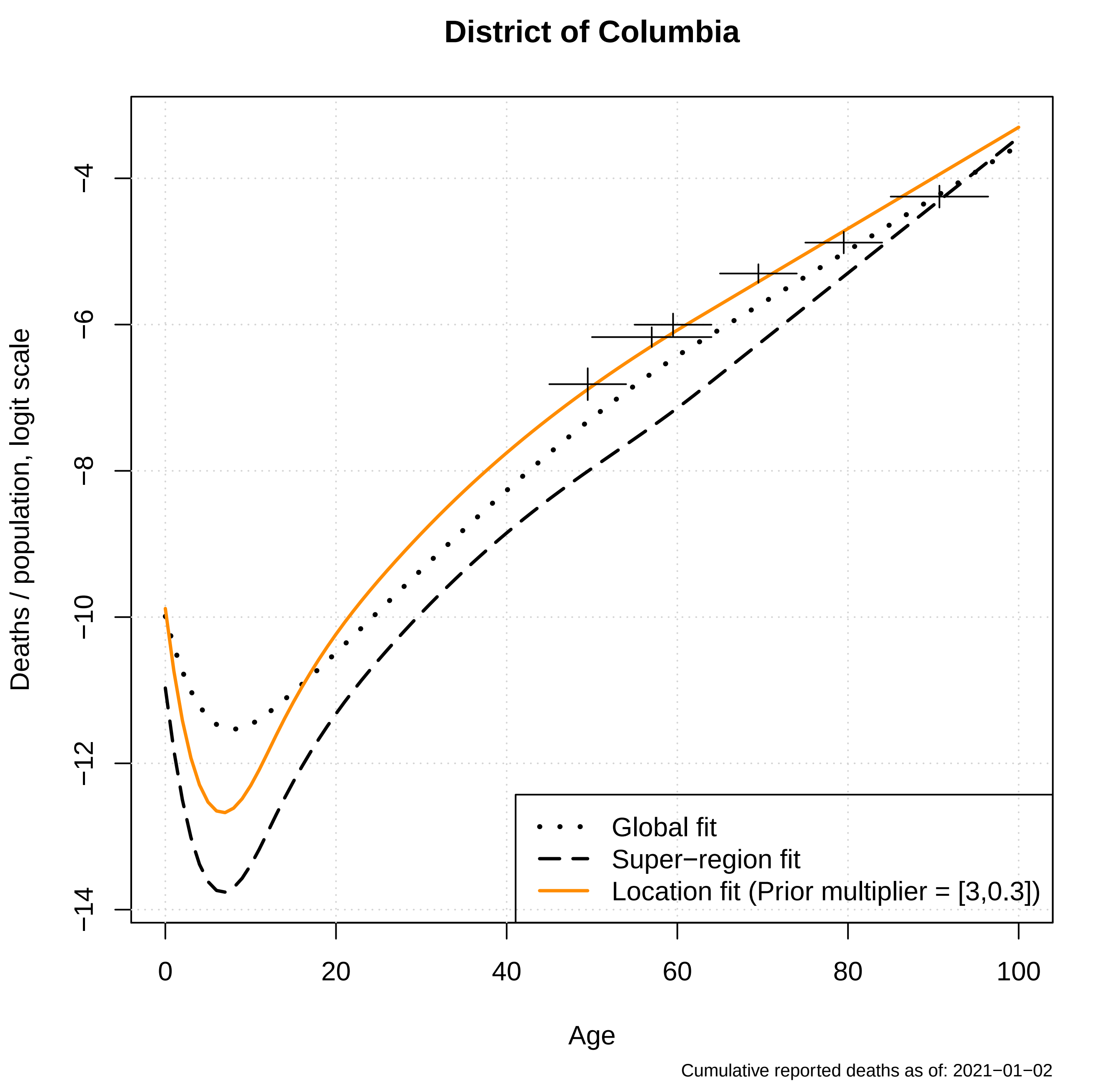

District of Columbia
Deaths / population, logit scale
−4
−6
−8
−10
−12
−14
0
20
40
60
80
100
Age
Global fit
Super−region fit
Location fit (Prior multiplier = [3,0.3])
Cumulative reported deaths as of: 2021−01−02

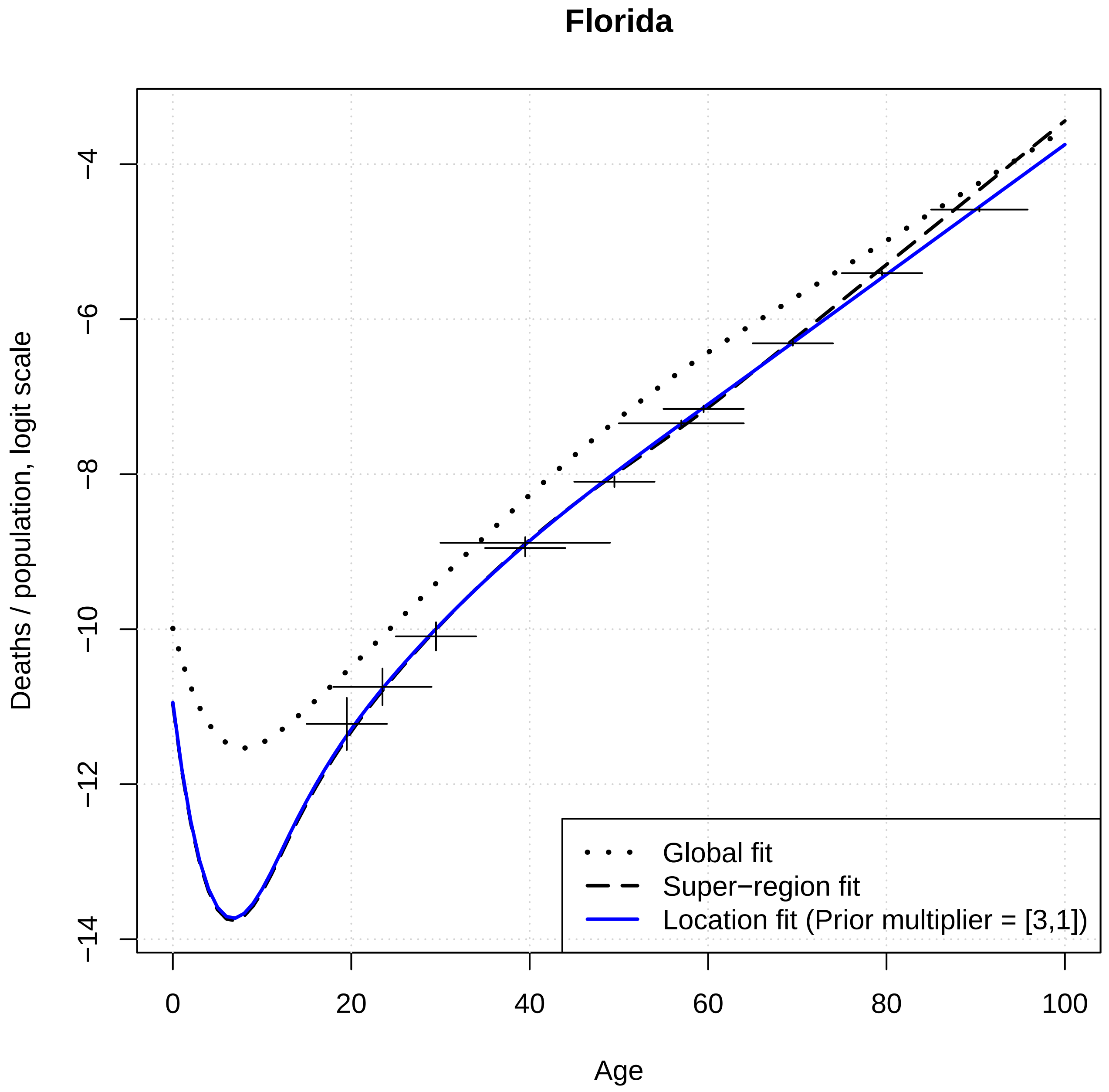

Cumulative reported deaths as of: 2021−01−02

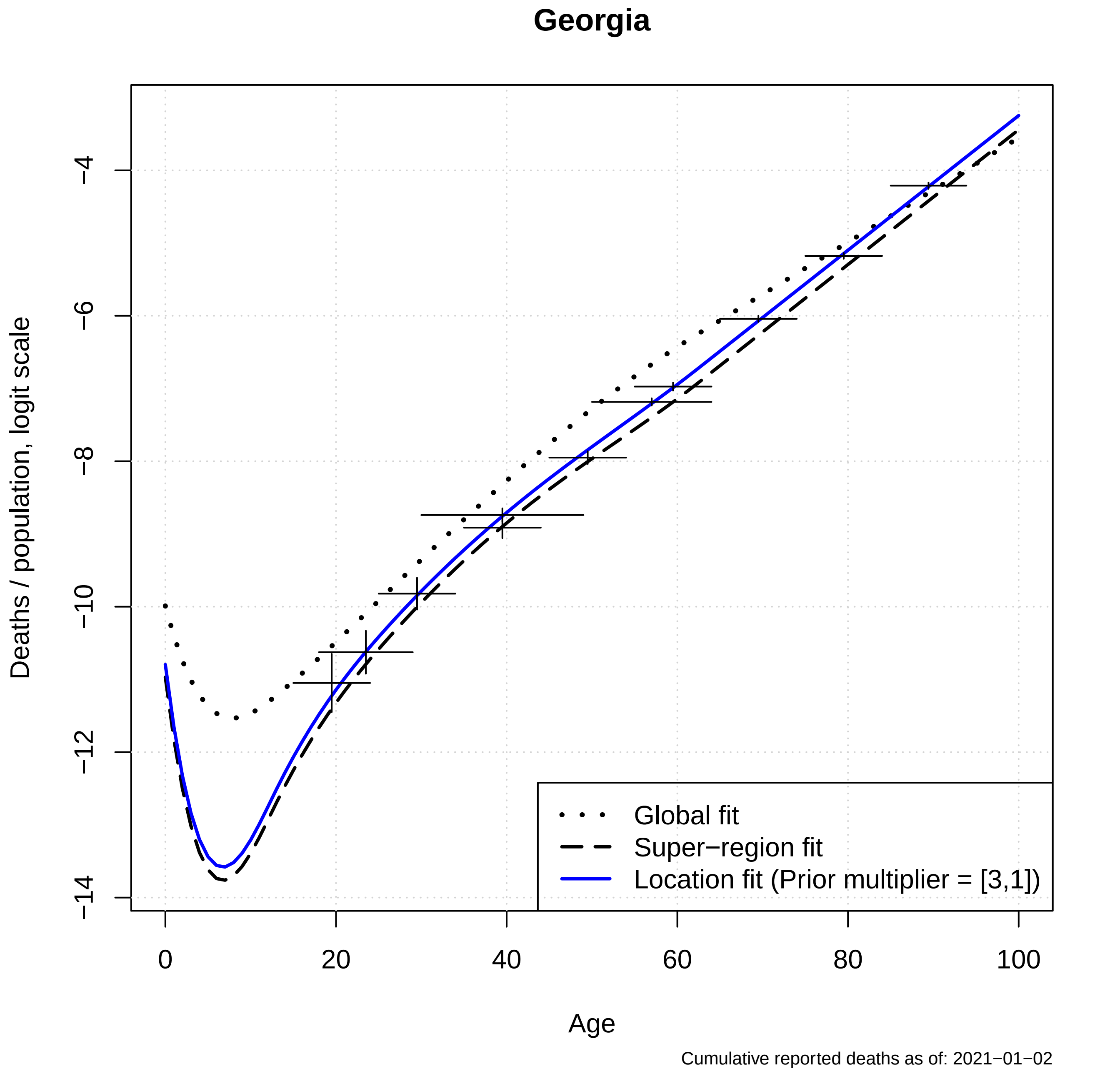

Georgia
Deaths / population, logit scale
−4
−6
−8
−10
−12
−14
0
20
40
60
80
100
Age
Global fit
Super−region fit
Location fit (Prior multiplier = [3,1])
Cumulative reported deaths as of: 2021−01−02

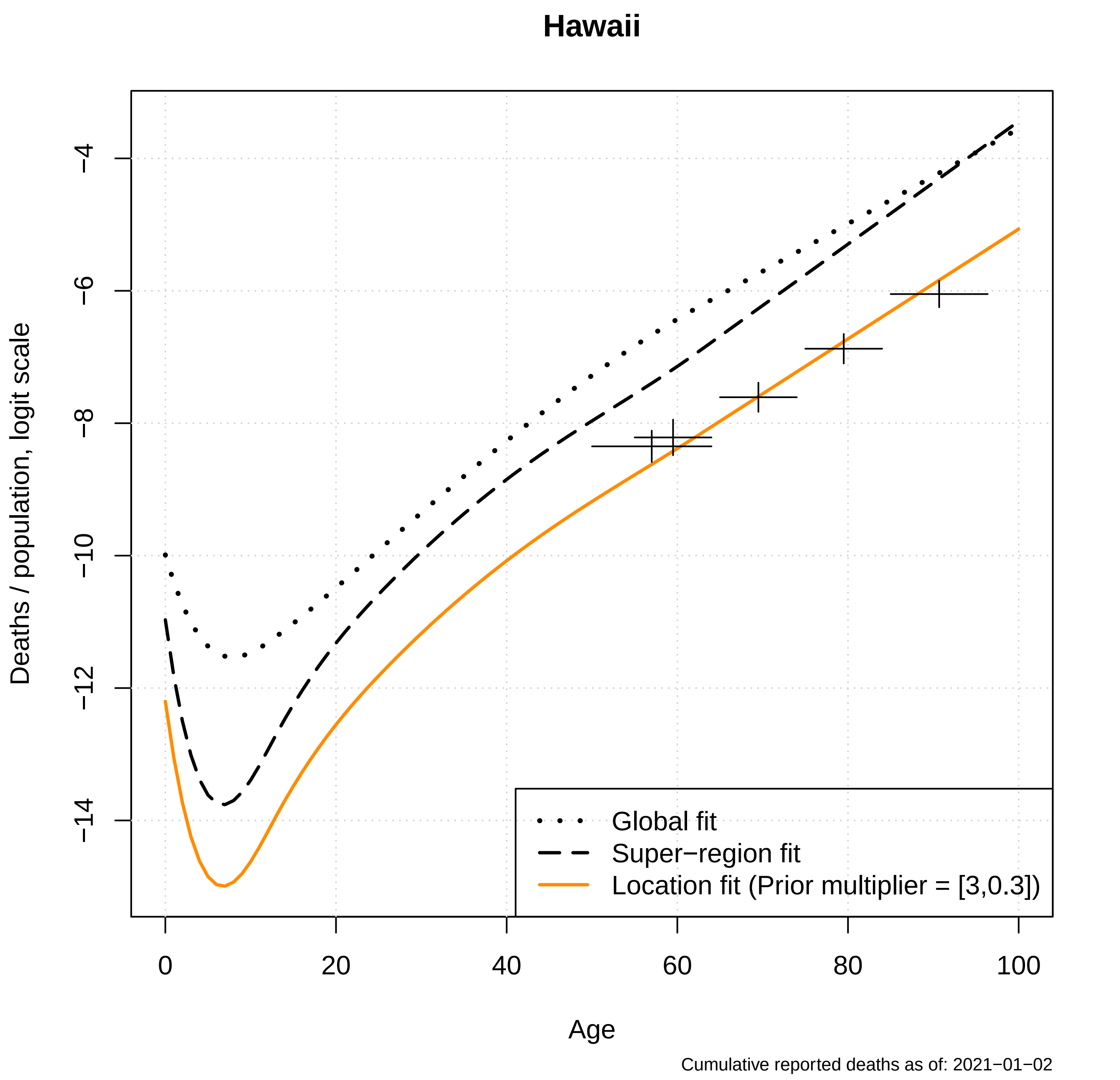
Hawaii
Deaths / population, logit scale
−4
−6
−8
−10
−12
−14
0
20
40
60
80
100
Age
Global fit
Super−region fit
Location fit (Prior multiplier = [3,0.3])
Cumulative reported deaths as of: 2021−01−02

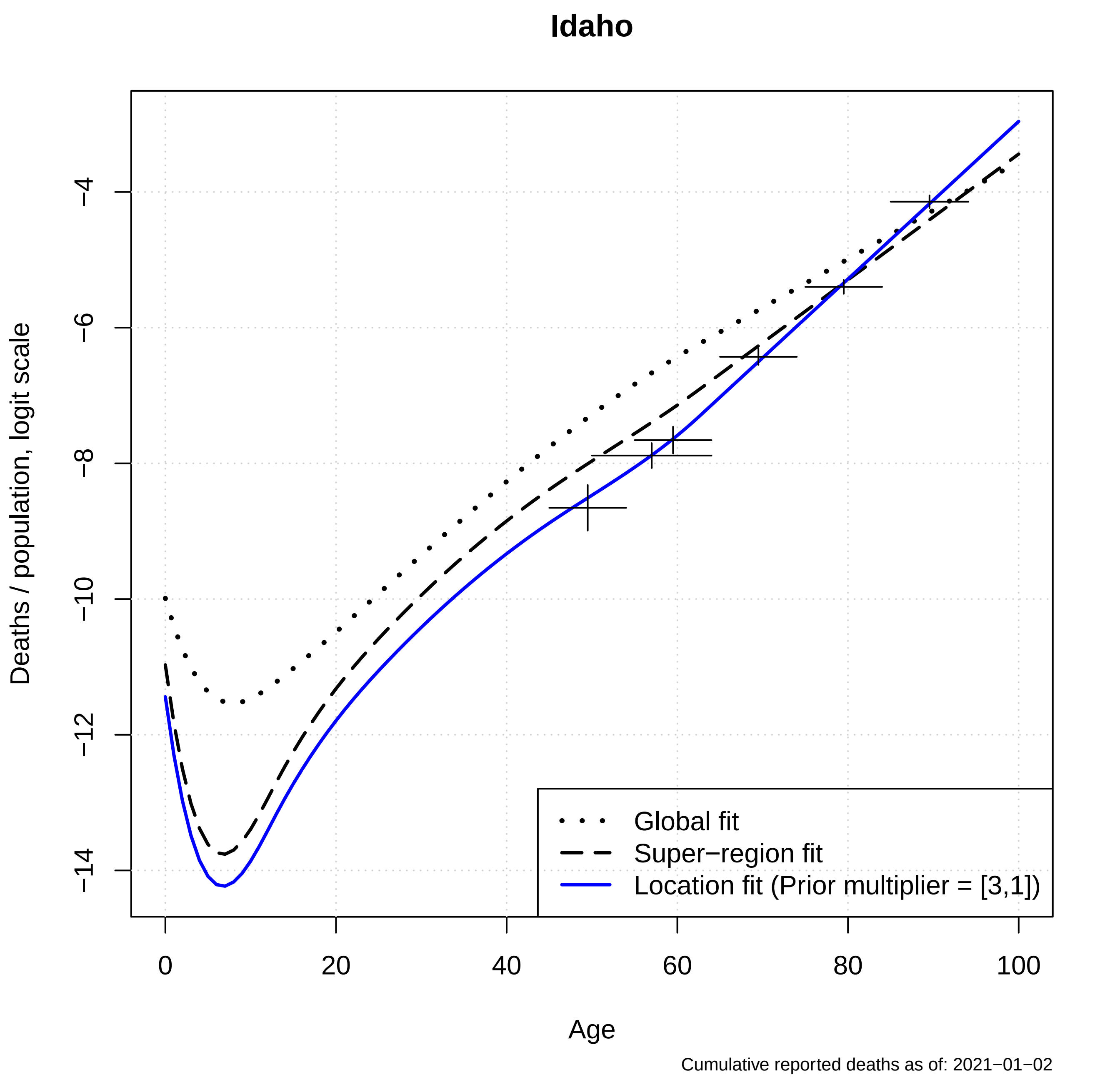

Idaho
Deaths / population, logit scale
−4
−6
−8
−10
−12
−14
0
20
40
60
80
100
Age
Global fit
Super−region fit
Location fit (Prior multiplier = [3,1])
Cumulative reported deaths as of: 2021−01−02

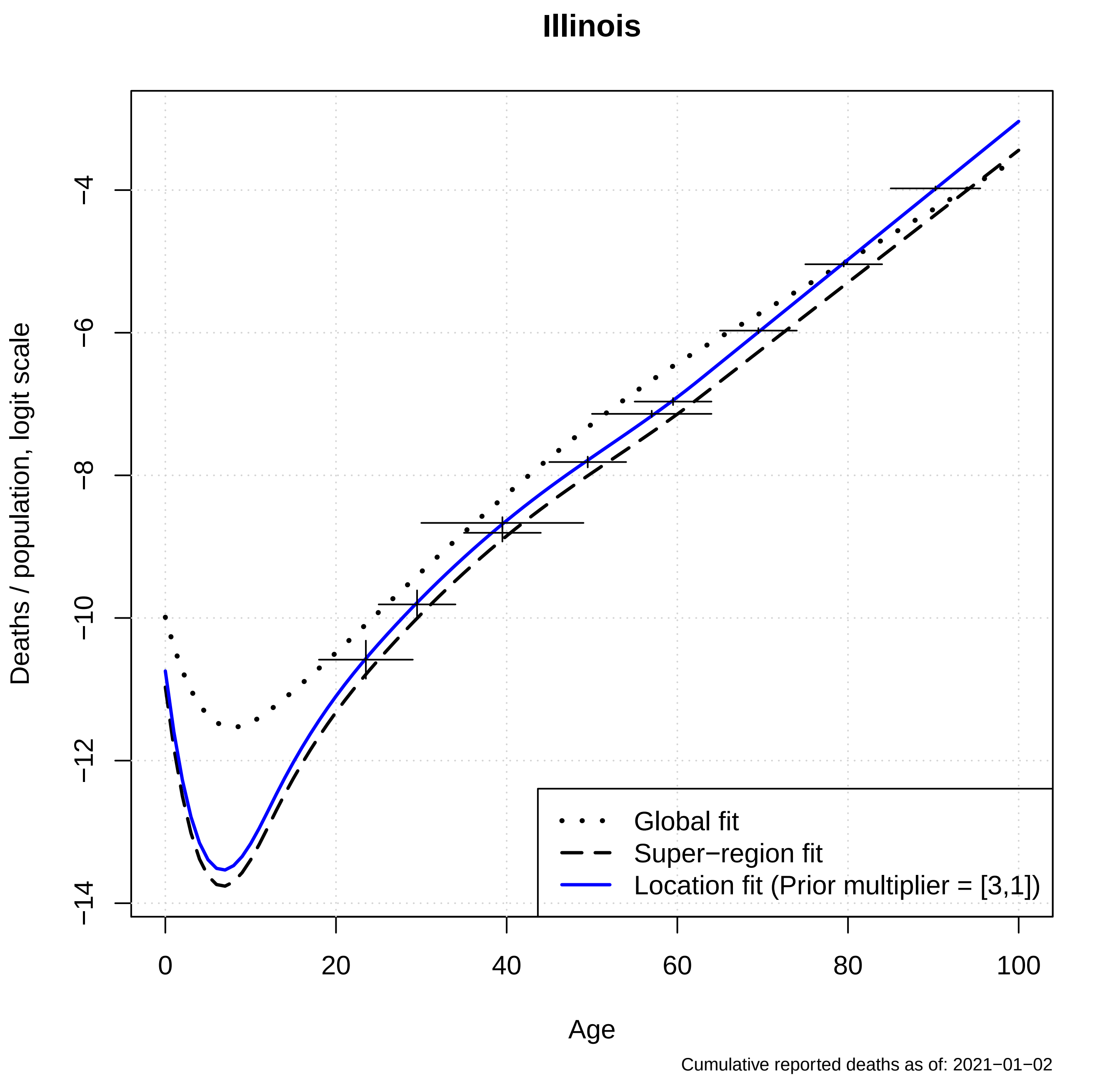

Illinois
Deaths / population, logit scale
−4
−6
−8
−10
−12
−14
0
20
40
60
80
100
Age
Global fit
Super−region fit
Location fit (Prior multiplier = [3,1])
Cumulative reported deaths as of: 2021−01−02

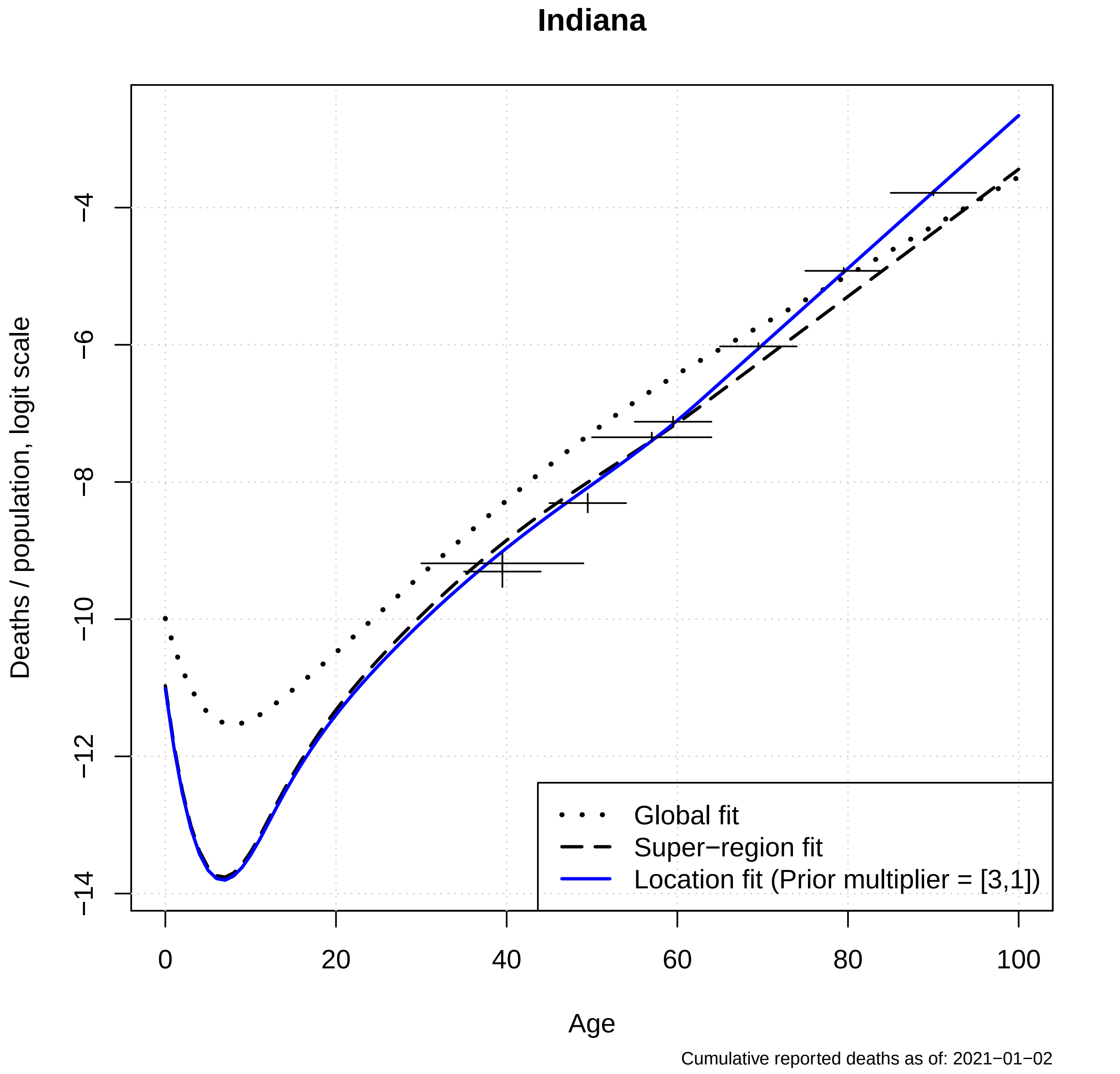
Indiana
Deaths / population, logit scale
−4
−6
−8
−10
−12
−14
0
20
40
60
80
100
Age
Global fit
Super−region fit
Location fit (Prior multiplier = [3,1])
Cumulative reported deaths as of: 2021−01−02

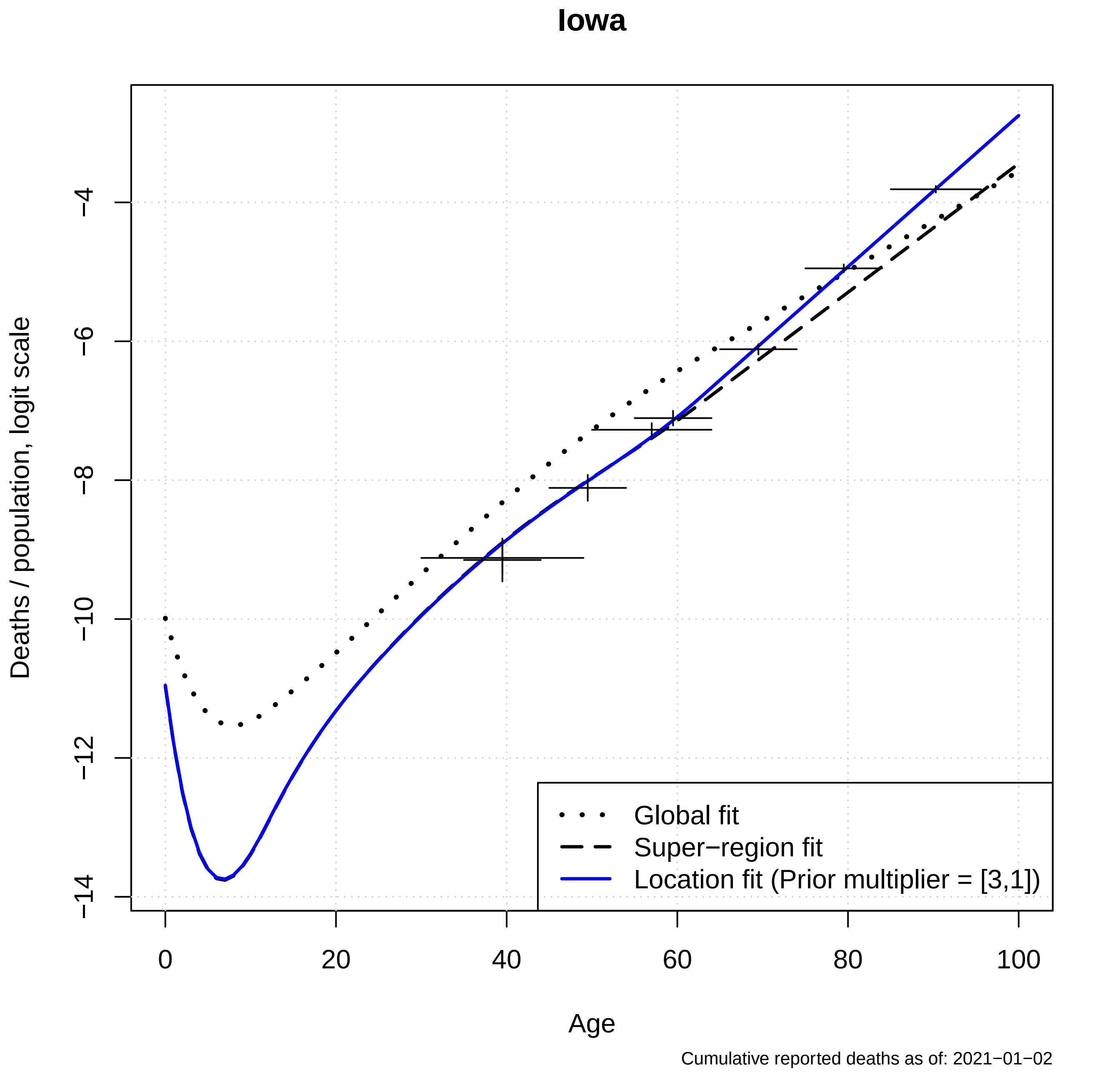
Iowa
Deaths / population, logit scale
−4
−6
−8
−10
−12
−14
0
20
40
60
80
100
Age
Global fit
Super−region fit
Location fit (Prior multiplier = [3,1])
Cumulative reported deaths as of: 2021−01−02

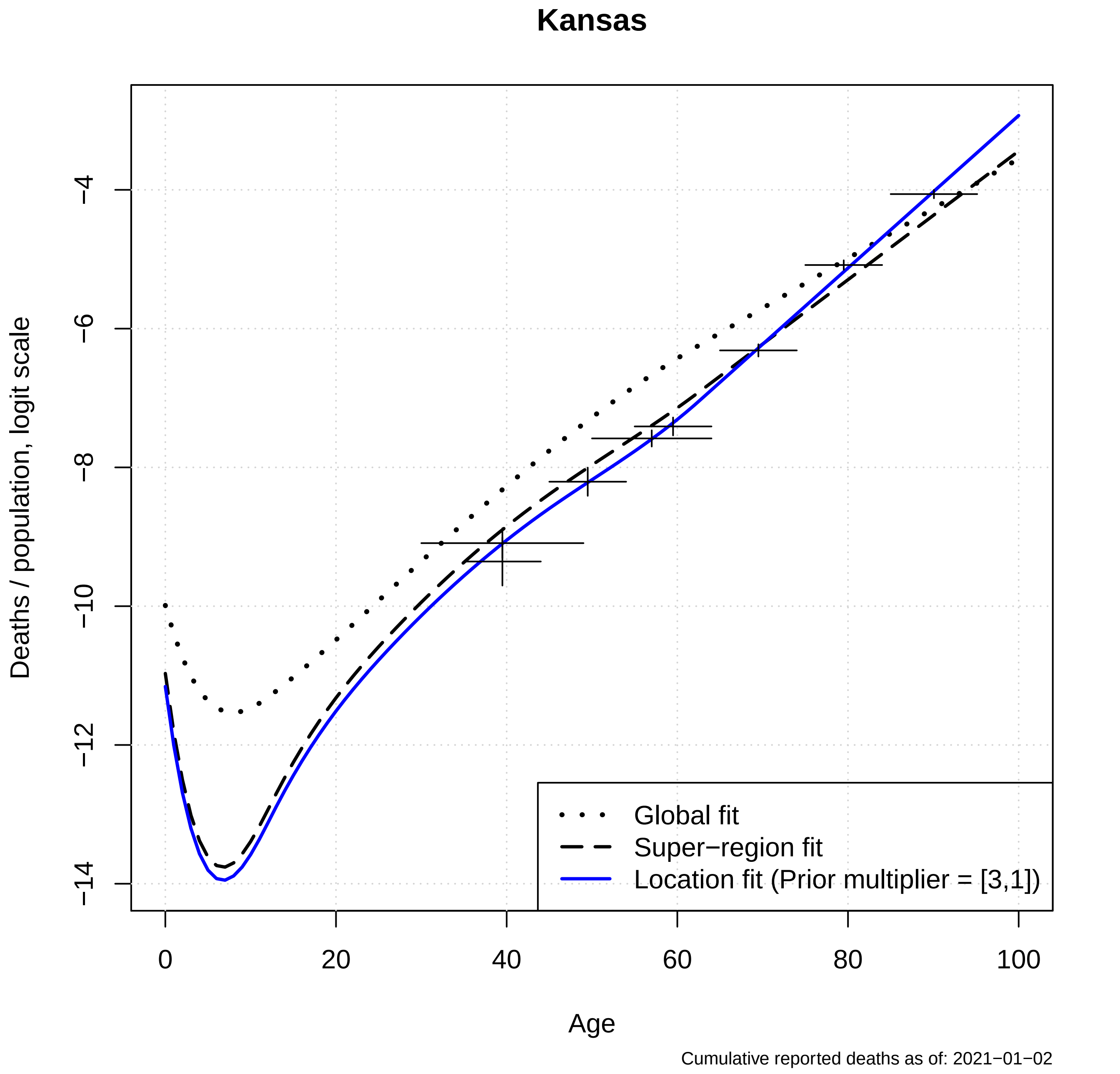

Kansas
Deaths / population, logit scale
−4
−6
−8
−10
−12
−14
0
20
40
60
80
100
Age
Global fit
Super−region fit
Location fit (Prior multiplier = [3,1])
Cumulative reported deaths as of: 2021−01−02

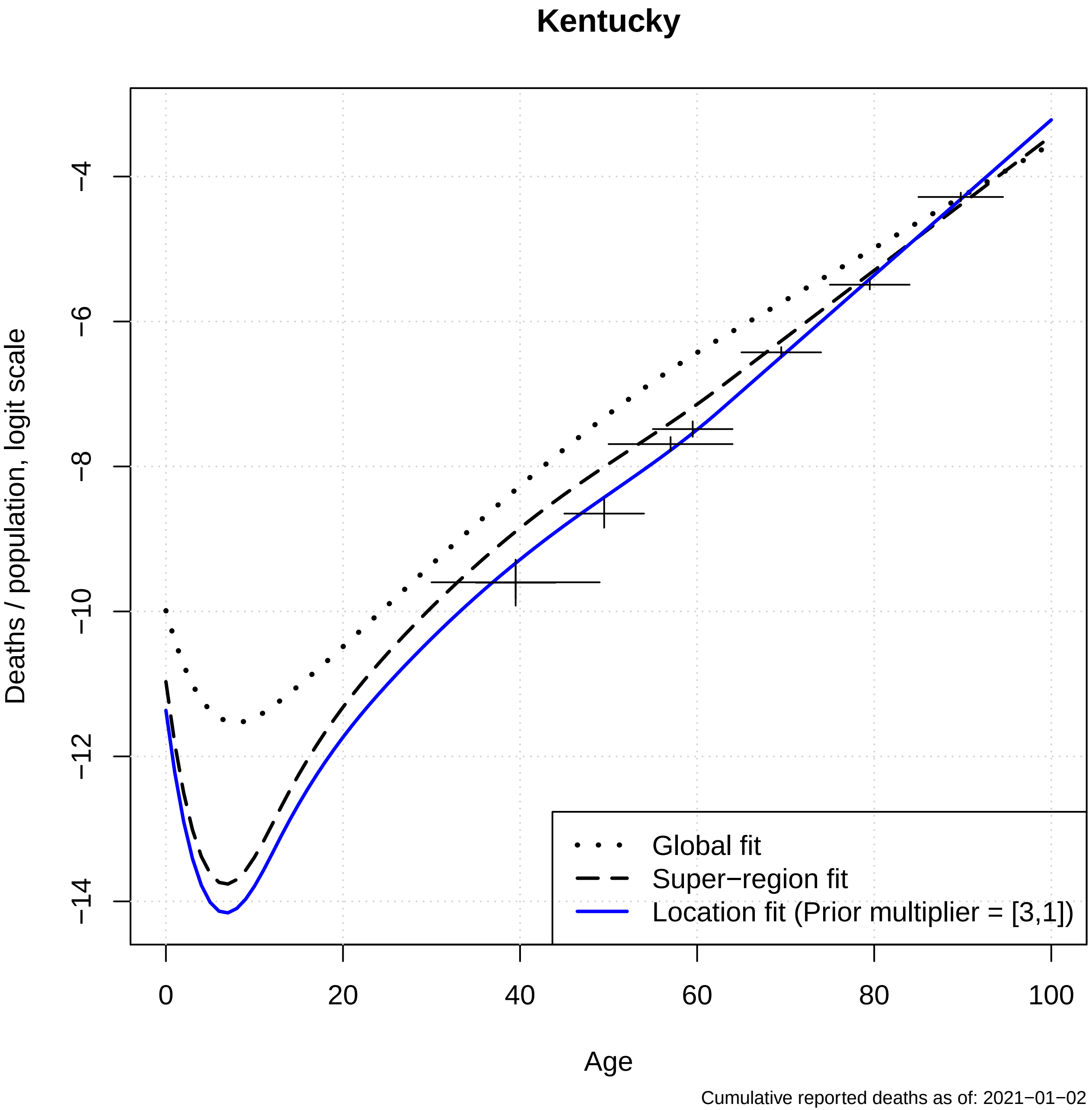

Kentucky
Deaths / population, logit scale
−4
−6
−8
−10
−12
−14
0
20
40
60
80
100
Age
Global fit
Super−region fit
Location fit (Prior multiplier = [3,1])
Cumulative reported deaths as of: 2021−01−02

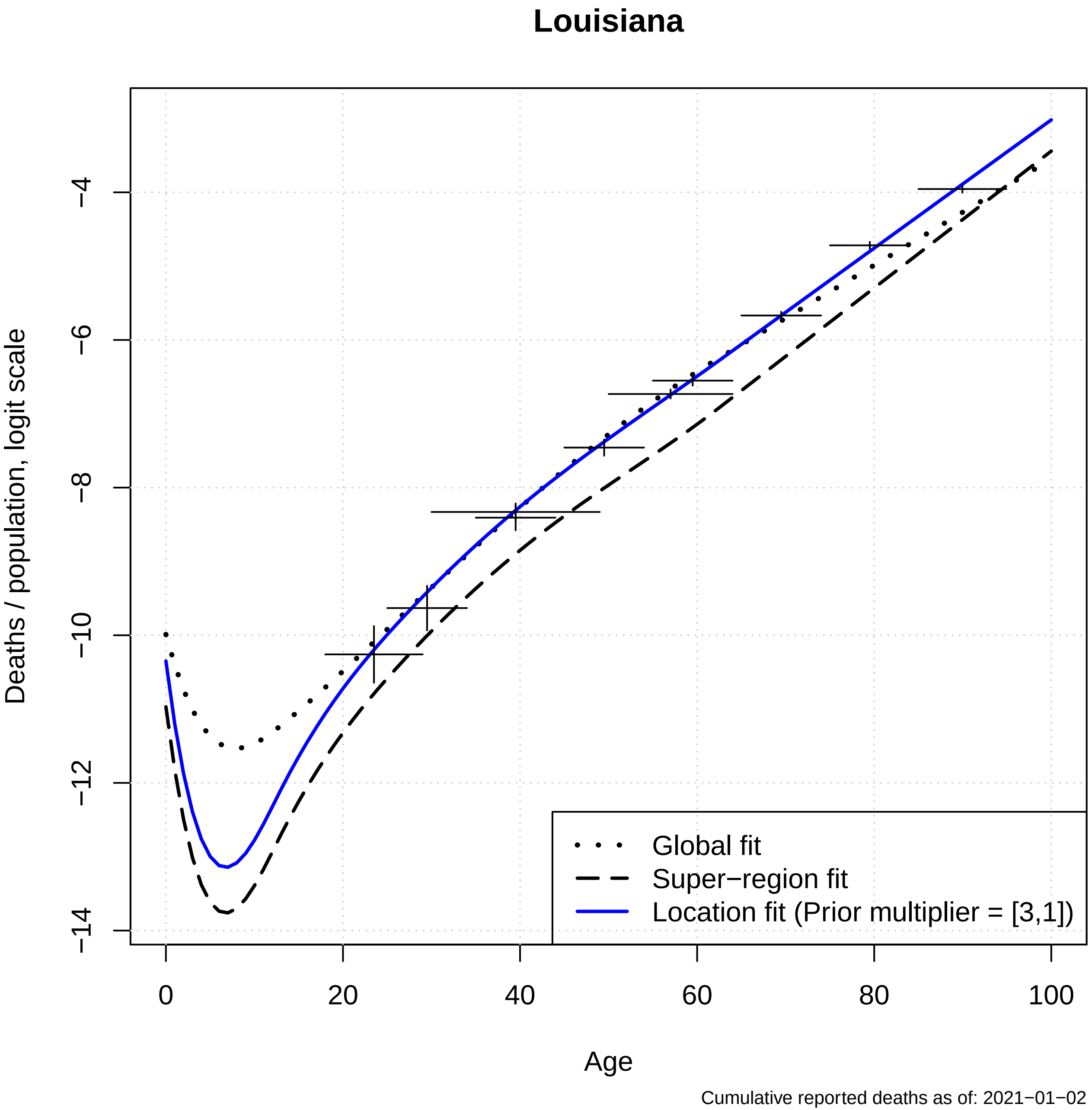
Louisiana
Deaths / population, logit scale
−4
−6
−8
−10
−12
−14
0
20
40
60
80
100
Age
Global fit
Super−region fit
Location fit (Prior multiplier = [3,1])
Cumulative reported deaths as of: 2021−01−02

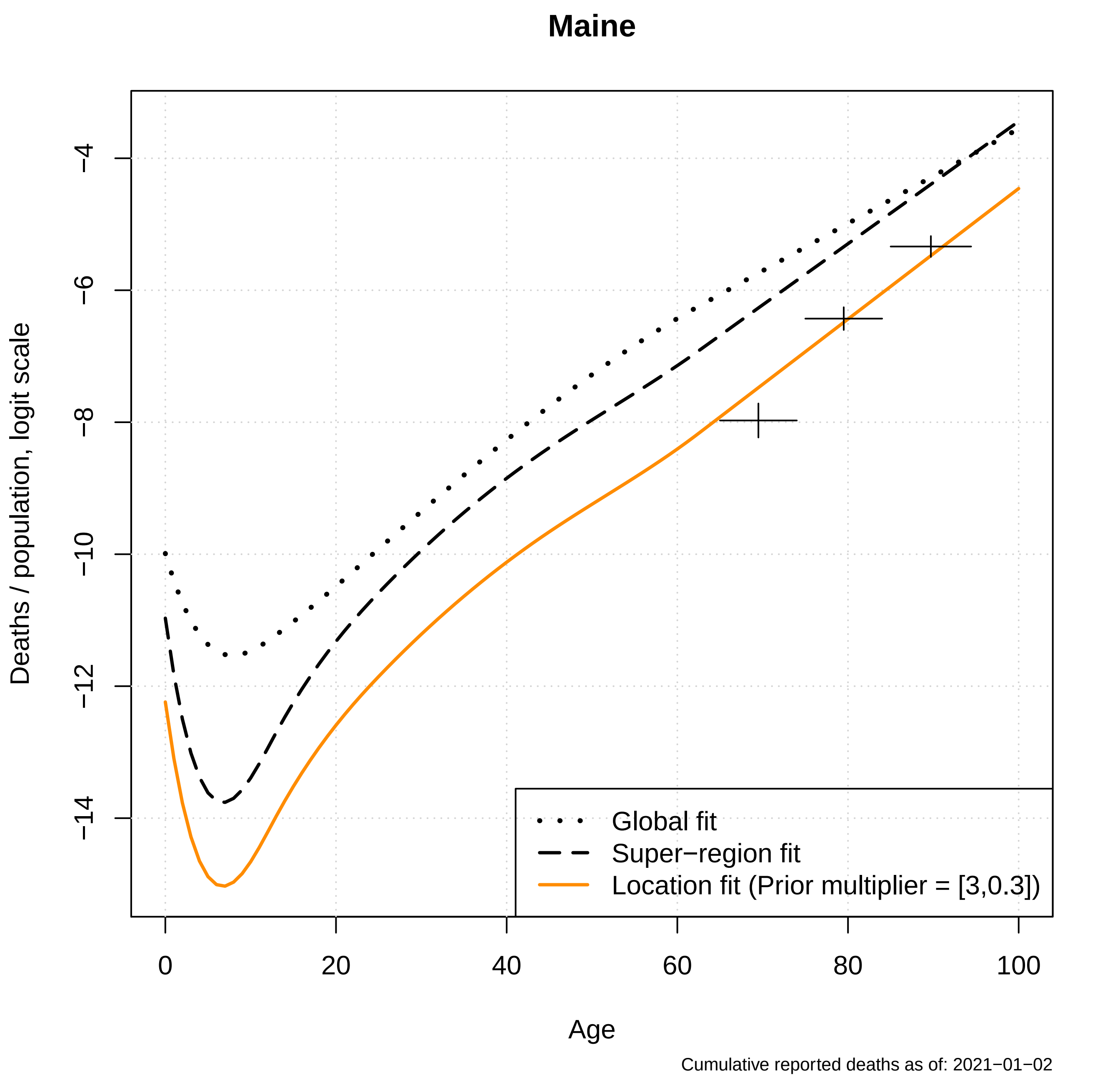

Maine
Deaths / population, logit scale
−4
−6
−8
−10
−12
−14
0
20
40
60
80
100
Age
Global fit
Super−region fit
Location fit (Prior multiplier = [3,0.3])
Cumulative reported deaths as of: 2021−01−02

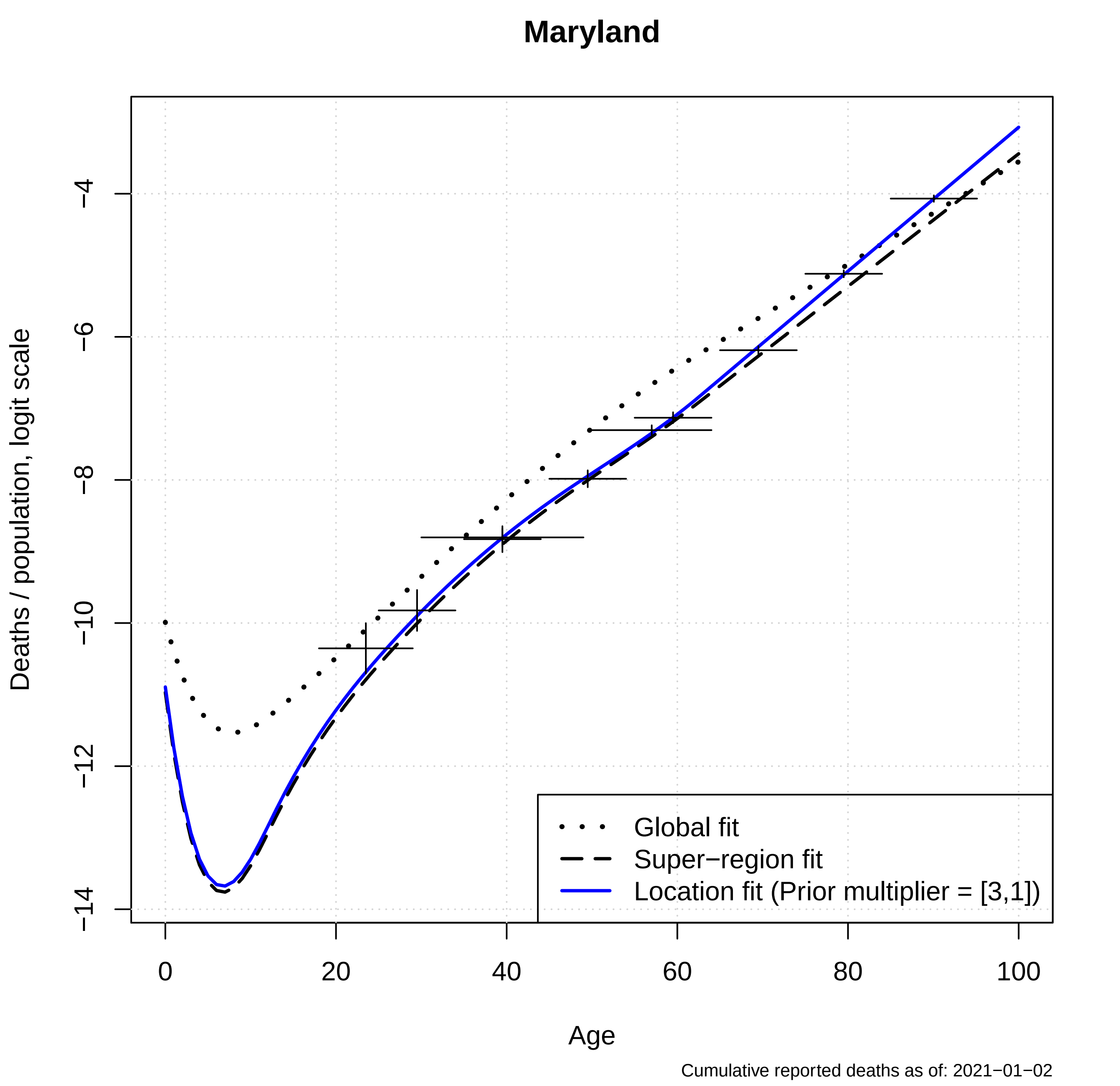

Maryland
Deaths / population, logit scale
−4
−6
−8
−10
−12
−14
0
20
40
60
80
100
Age
Global fit
Super−region fit
Location fit (Prior multiplier = [3,1])
Cumulative reported deaths as of: 2021−01−02

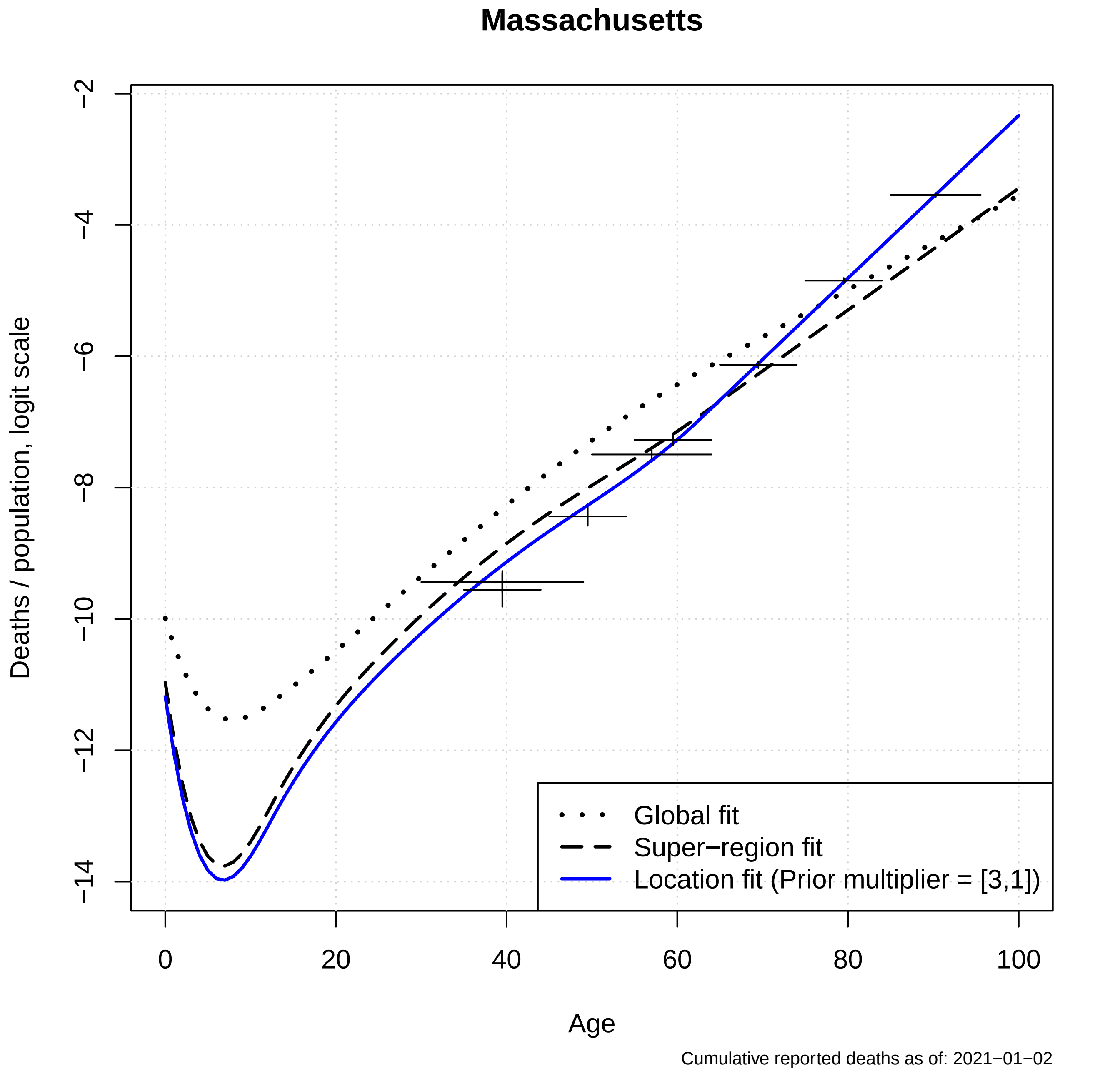
Massachusetts
Deaths / population, logit scale
−2
−4
−6
−8
−10
−12
−14
0
20
40
60
80
100
Age
Global fit
Super−region fit
Location fit (Prior multiplier = [3,1])
Cumulative reported deaths as of: 2021−01−02

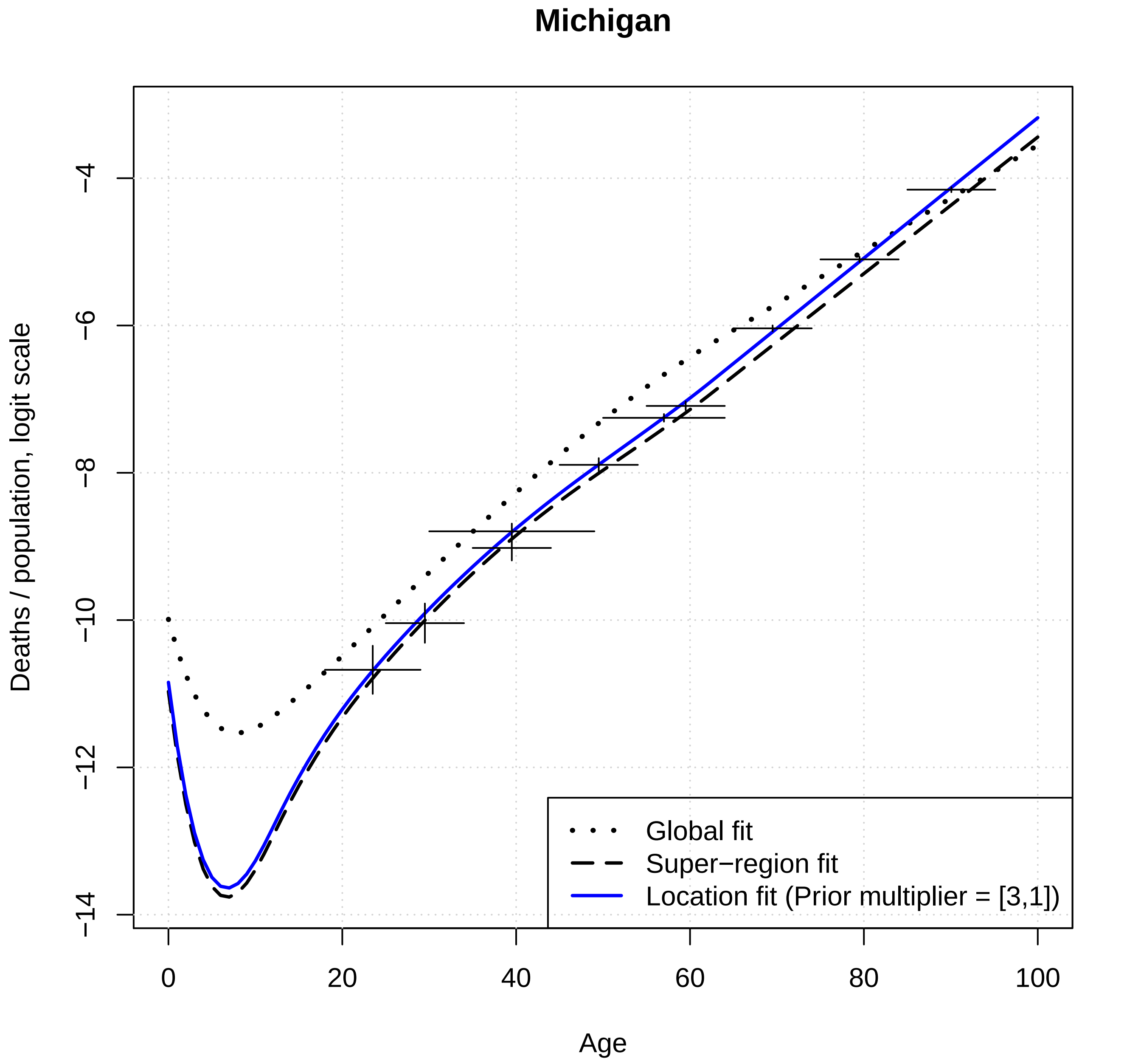

Cumulative reported deaths as of: 2021−01−02

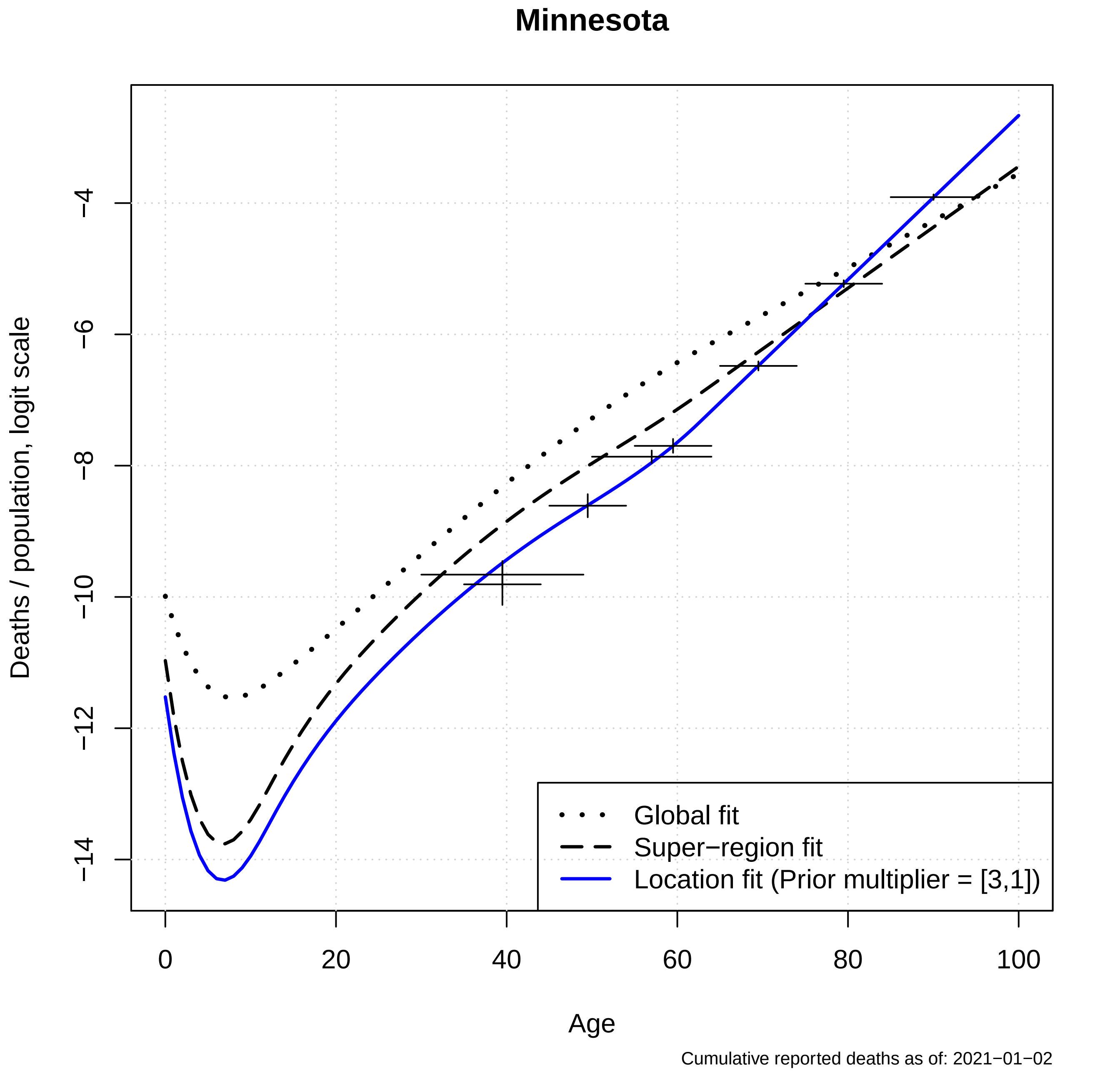

Minnesota
Deaths / population, logit scale
−4
−6
−8
−10
−12
−14
0
20
40
60
80
100
Age
Global fit
Super−region fit
Location fit (Prior multiplier = [3,1])
Cumulative reported deaths as of: 2021−01−02

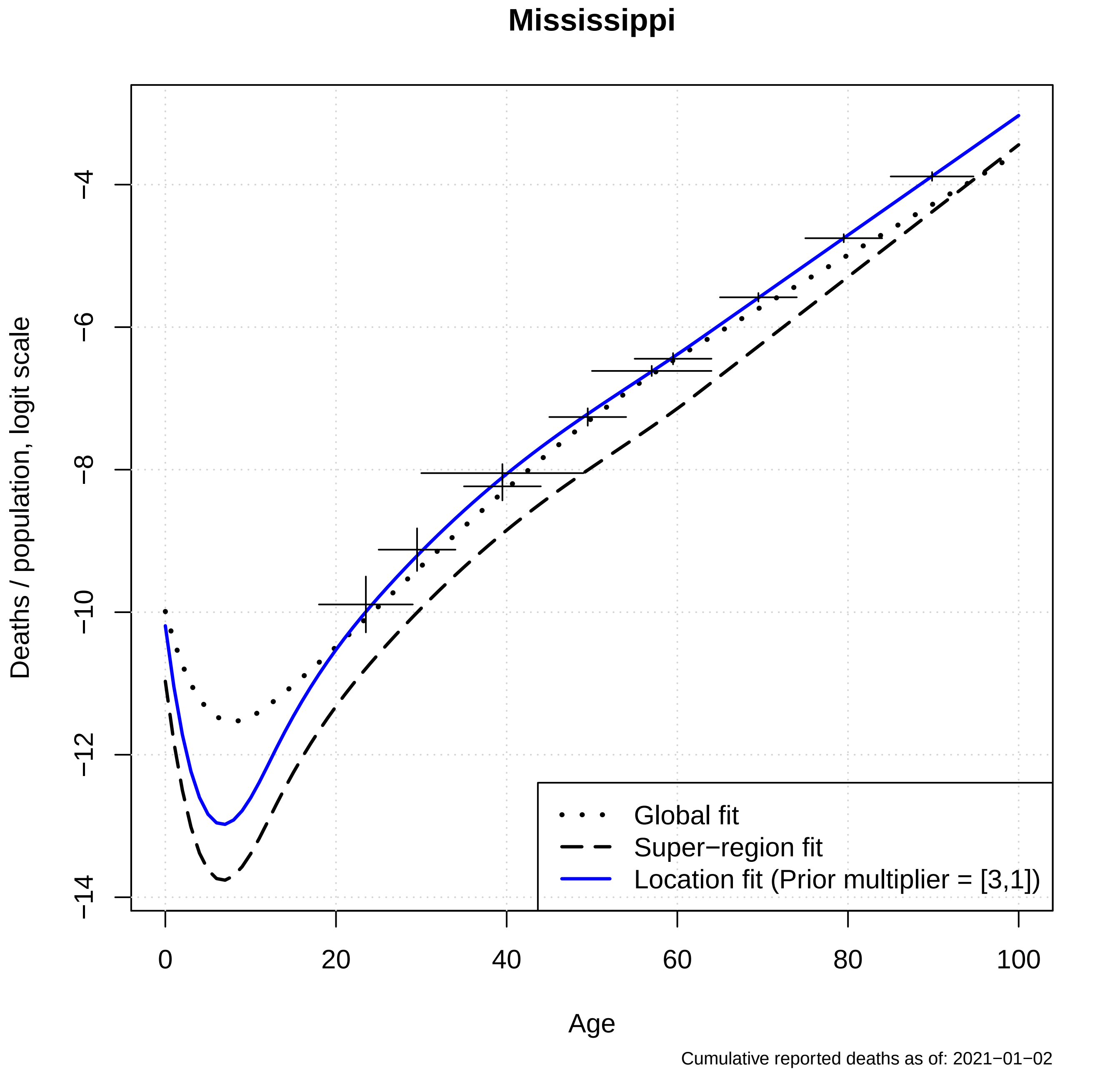

Mississippi
Deaths / population, logit scale
−4
−6
−8
−10
−12
−14
0
20
40
60
80
100
Age
Global fit
Super−region fit
Location fit (Prior multiplier = [3,1])
Cumulative reported deaths as of: 2021−01−02

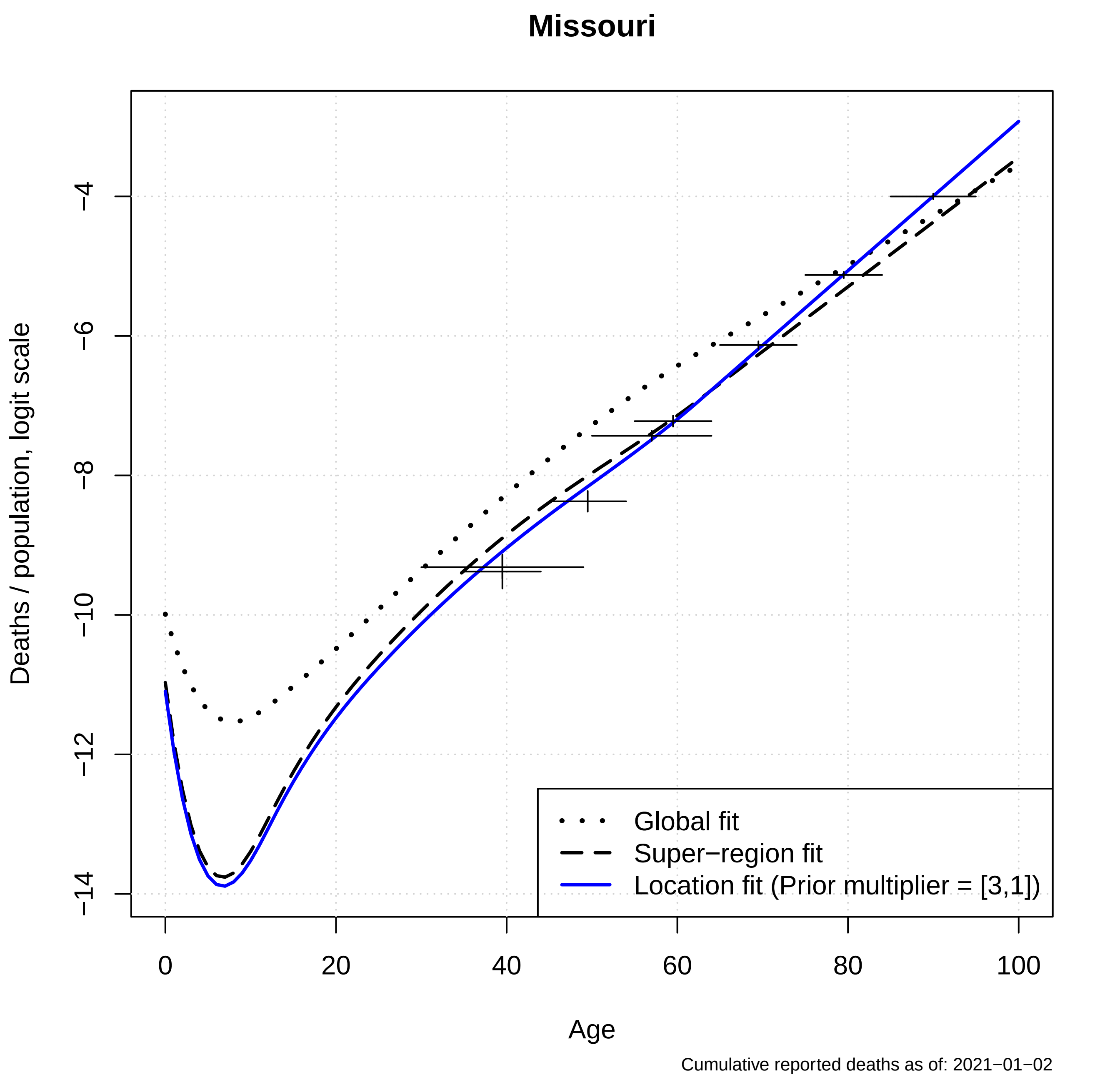

Missouri
Deaths / population, logit scale
−4
−6
−8
−10
−12
−14
0
20
40
60
80
100
Age
Global fit
Super−region fit
Location fit (Prior multiplier = [3,1])
Cumulative reported deaths as of: 2021−01−02

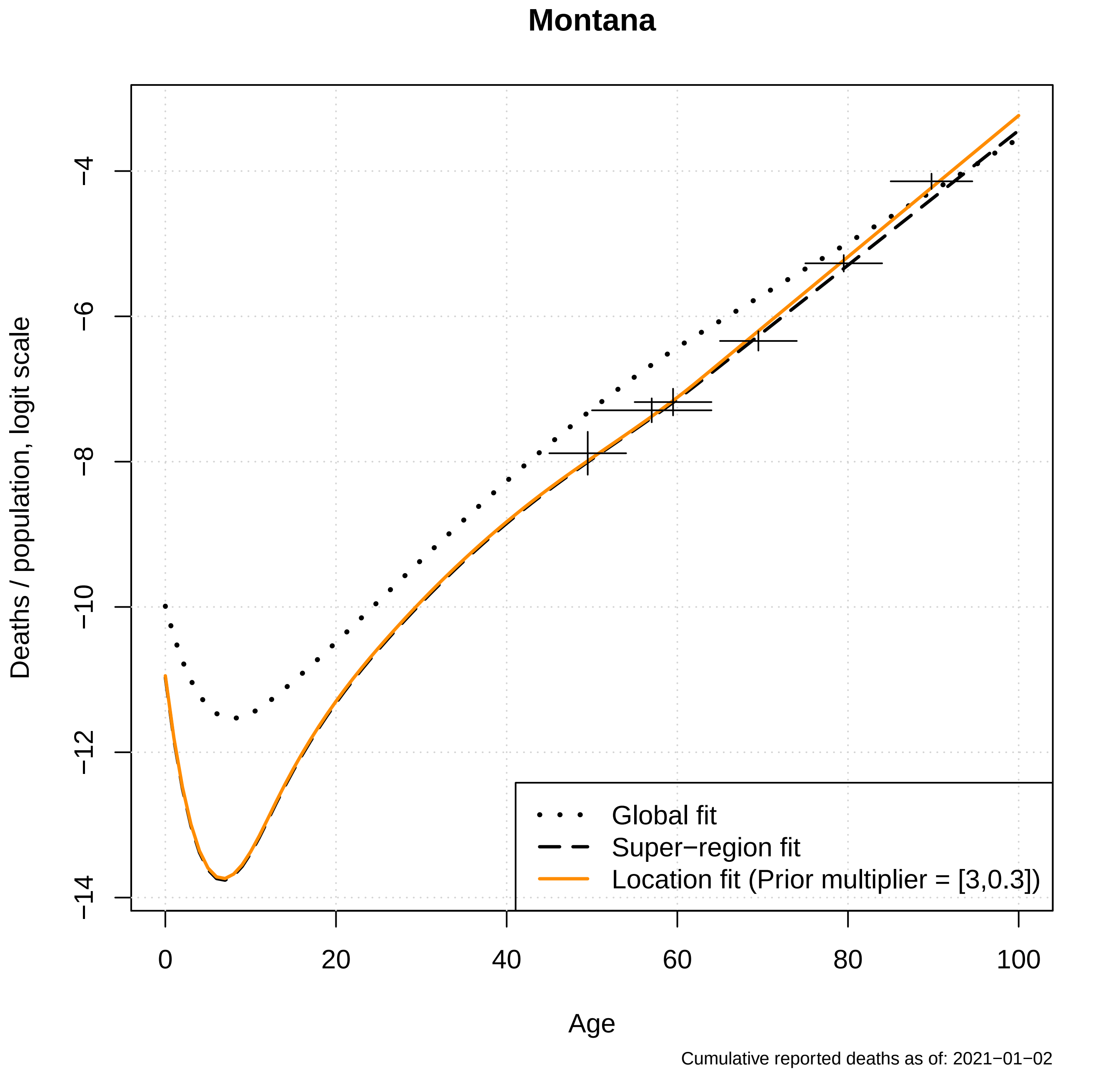

Montana
Deaths / population, logit scale
−4
−6
−8
−10
−12
−14
0
20
40
60
80
100
Age
Global fit
Super−region fit
Location fit (Prior multiplier = [3,0.3])
Cumulative reported deaths as of: 2021−01−02

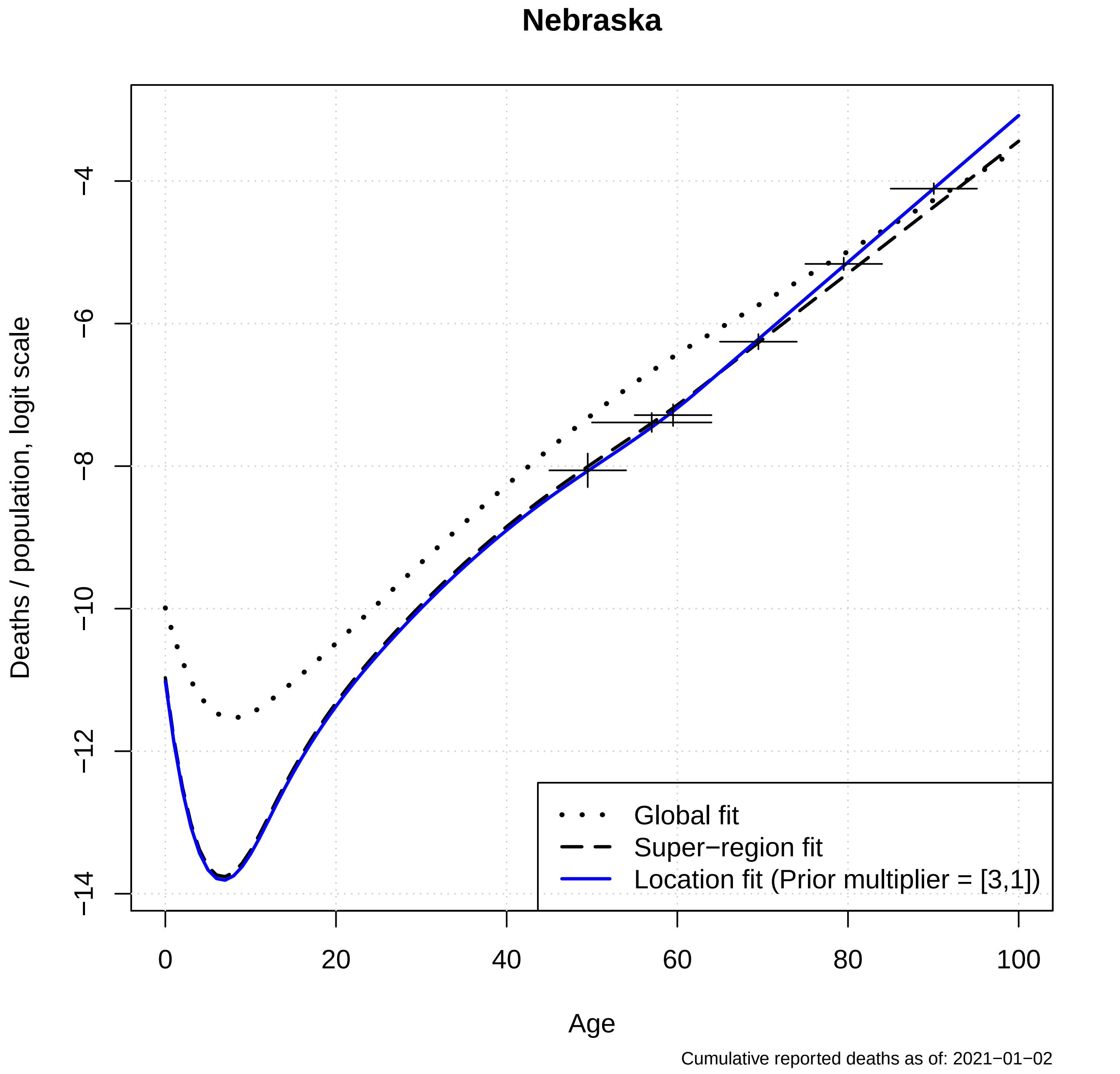

Nebraska
Deaths / population, logit scale
−4
−6
−8
−10
−12
−14
0
20
40
60
80
100
Age
Global fit
Super−region fit
Location fit (Prior multiplier = [3,1])
Cumulative reported deaths as of: 2021−01−02

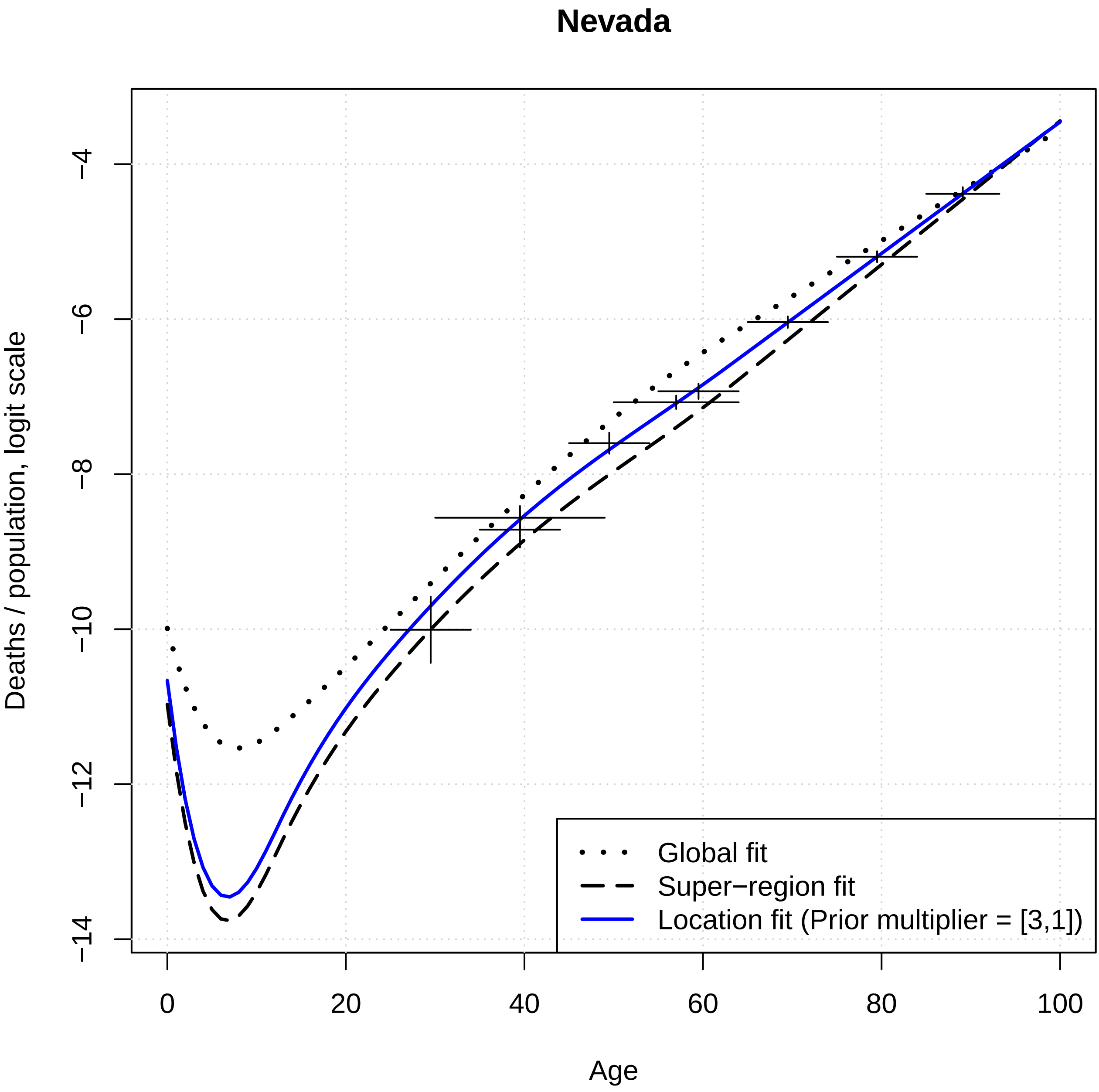

Cumulative reported deaths as of: 2021−01−02

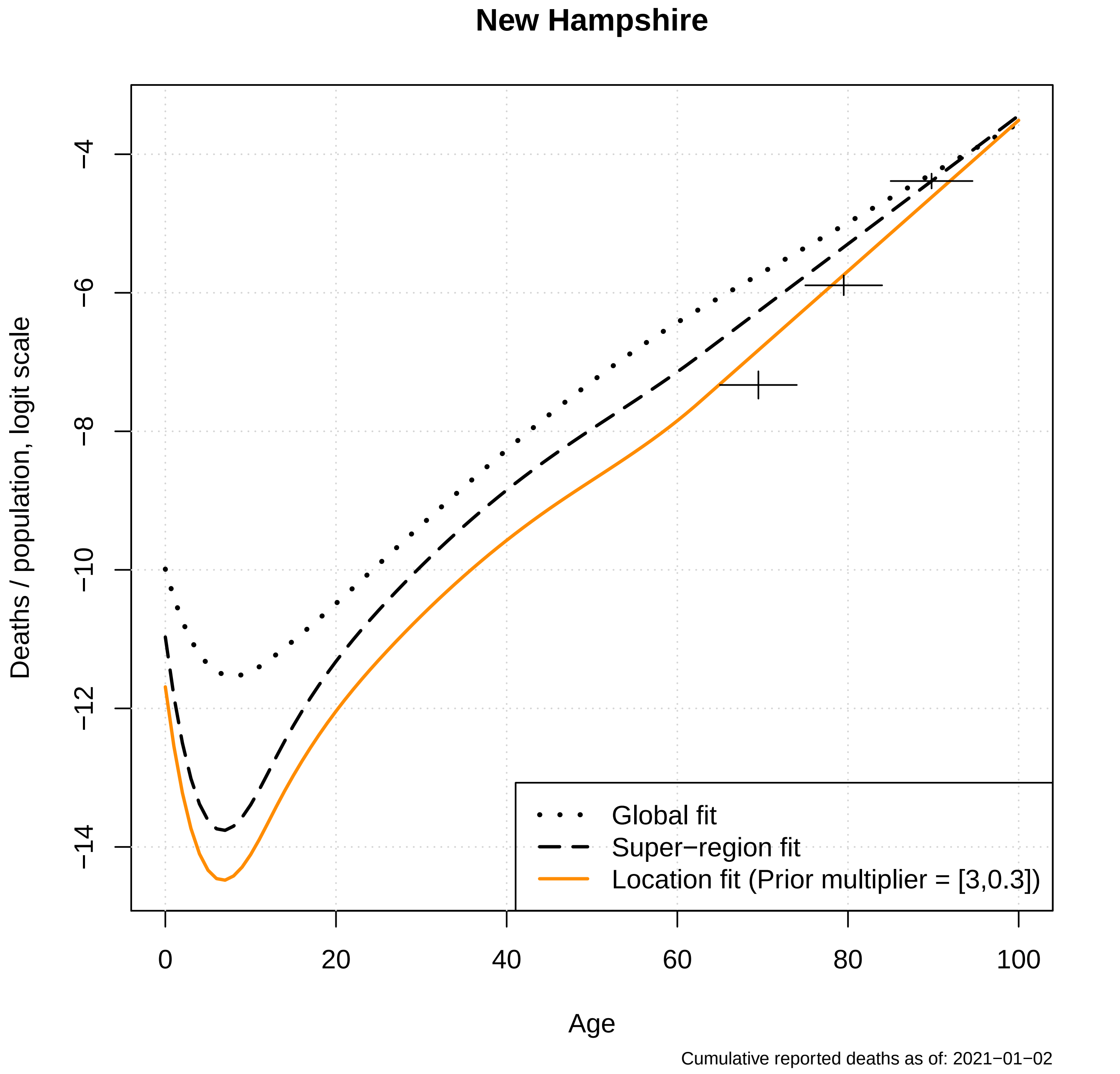
New Hampshire
Deaths / population, logit scale
−4
−6
−8
−10
−12
−14
0
20
40
60
80
100
Age
Global fit
Super−region fit
Location fit (Prior multiplier = [3,0.3])
Cumulative reported deaths as of: 2021−01−02

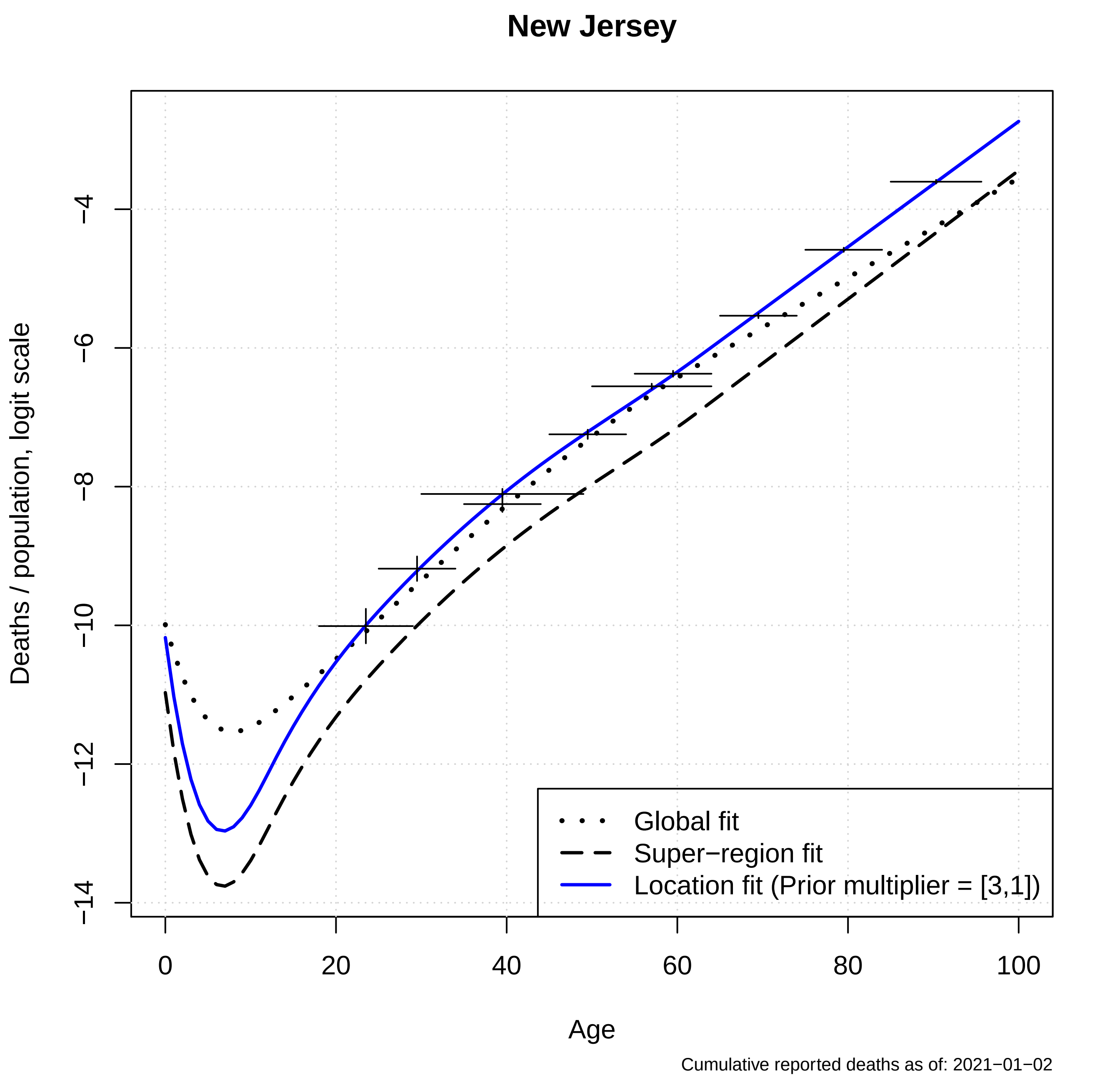
New Jersey
Deaths / population, logit scale
−4
−6
−8
−10
−12
−14
0
20
40
60
80
100
Age
Global fit
Super−region fit
Location fit (Prior multiplier = [3,1])
Cumulative reported deaths as of: 2021−01−02

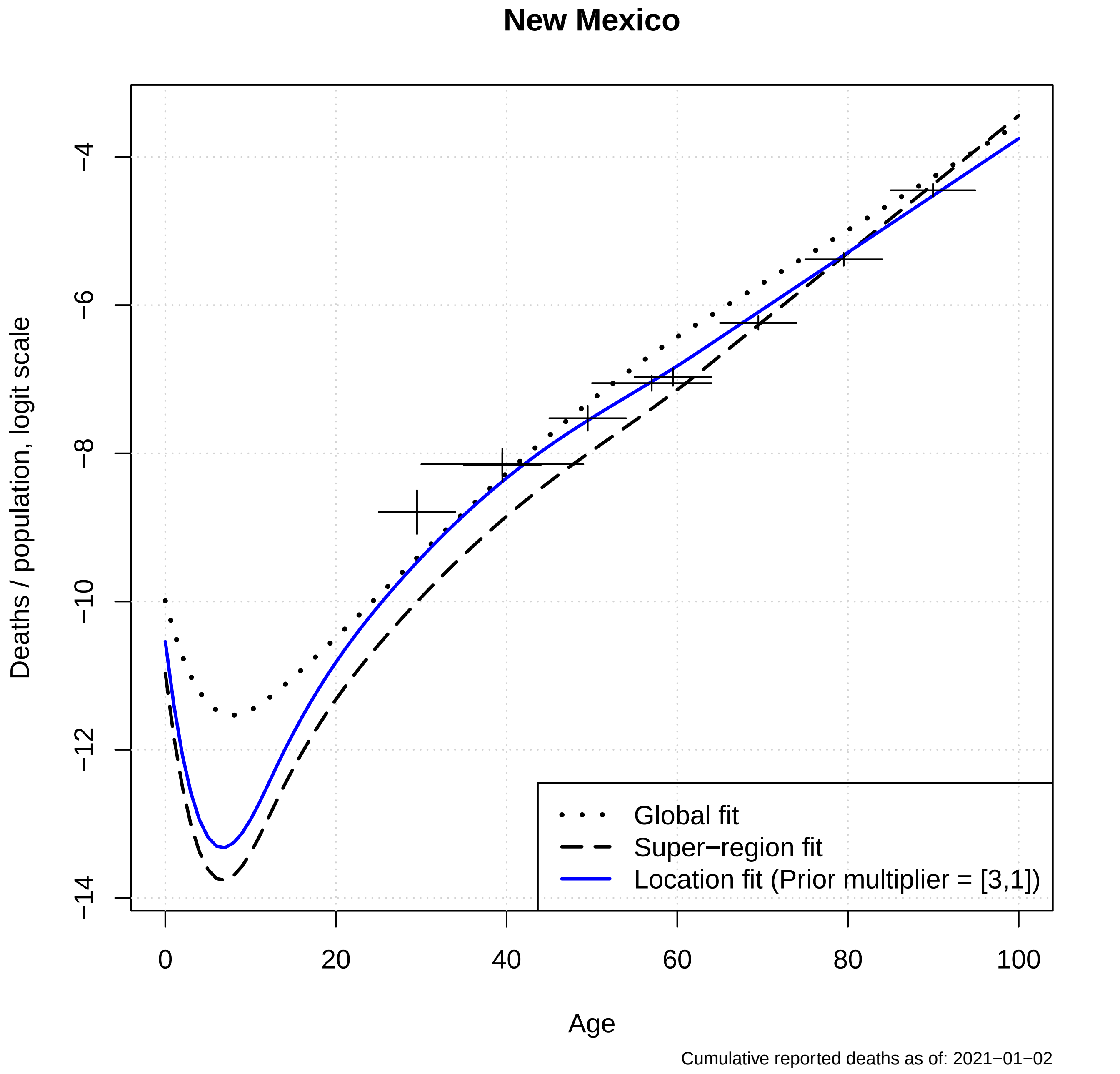
New Mexico
Deaths / population, logit scale
−4
−6
−8
−10
−12
−14
0
20
40
60
80
100
Age
Global fit
Super−region fit
Location fit (Prior multiplier = [3,1])
Cumulative reported deaths as of: 2021−01−02

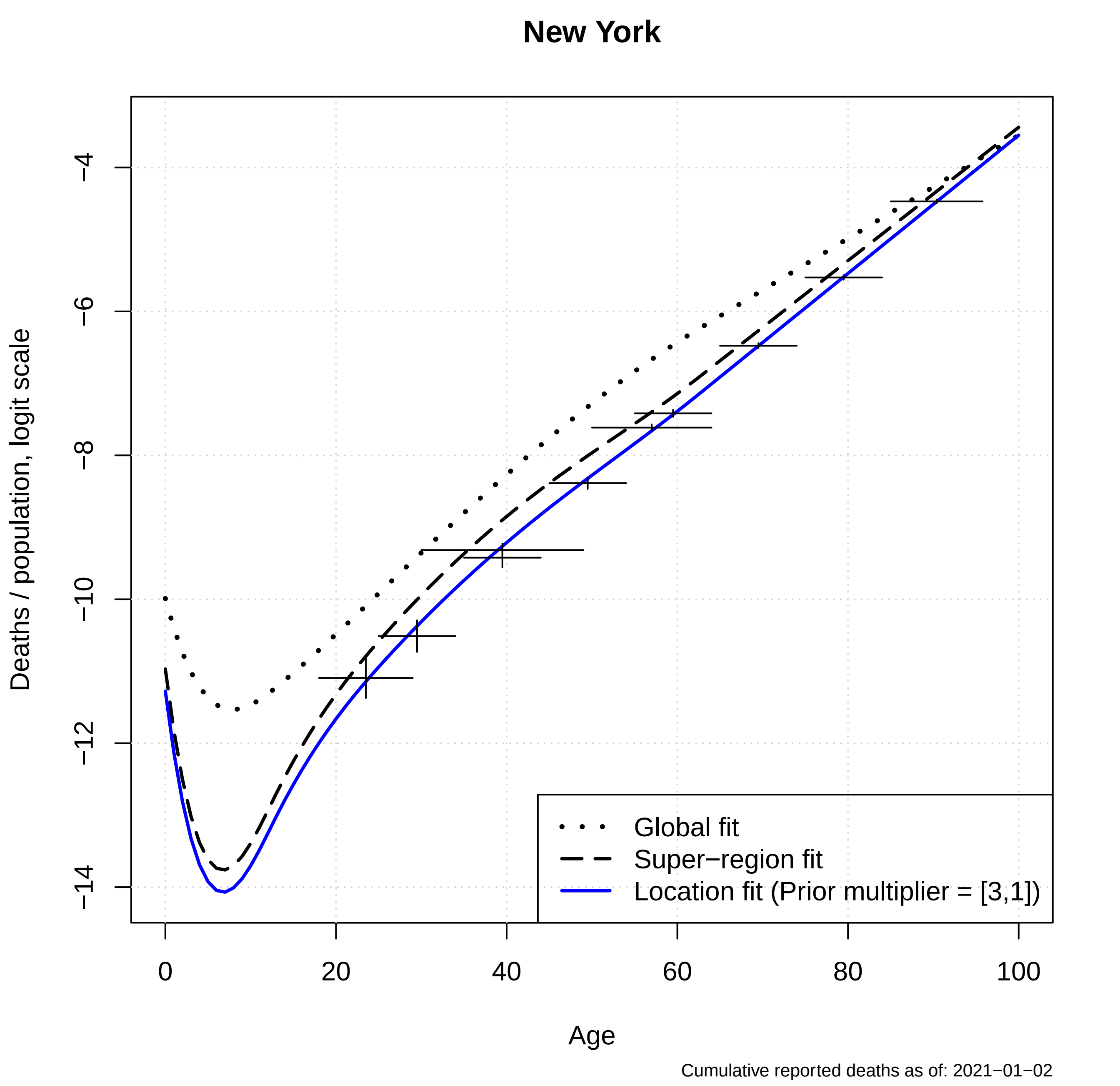
New York
Deaths / population, logit scale
−4
−6
−8
−10
−12
−14
0
20
40
60
80
100
Age
Global fit
Super−region fit
Location fit (Prior multiplier = [3,1])
Cumulative reported deaths as of: 2021−01−02

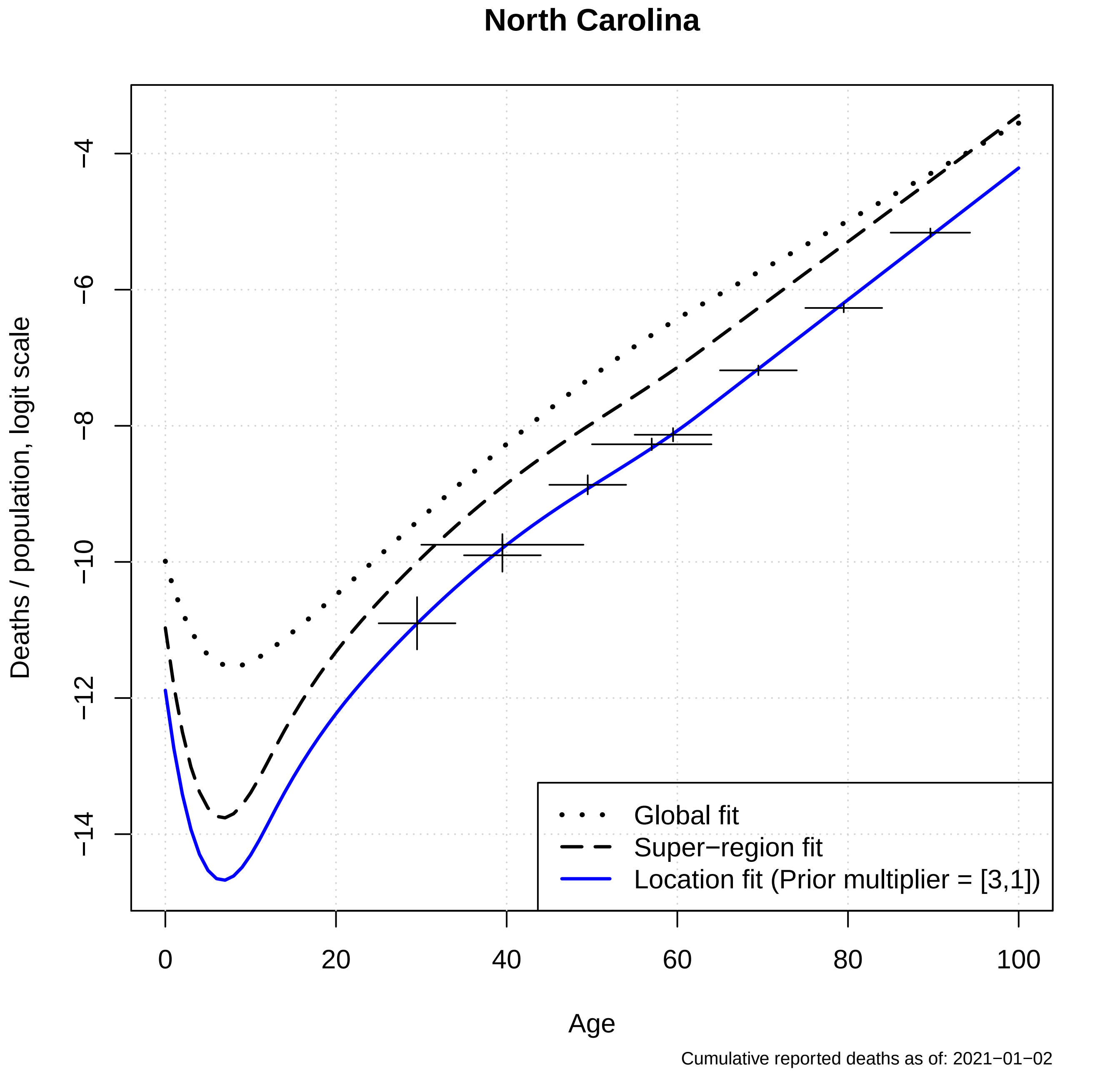

North Carolina
Deaths / population, logit scale
−4
−6
−8
−10
−12
−14
0
20
40
60
80
100
Age
Global fit
Super−region fit
Location fit (Prior multiplier = [3,1])
Cumulative reported deaths as of: 2021−01−02

North Dakota

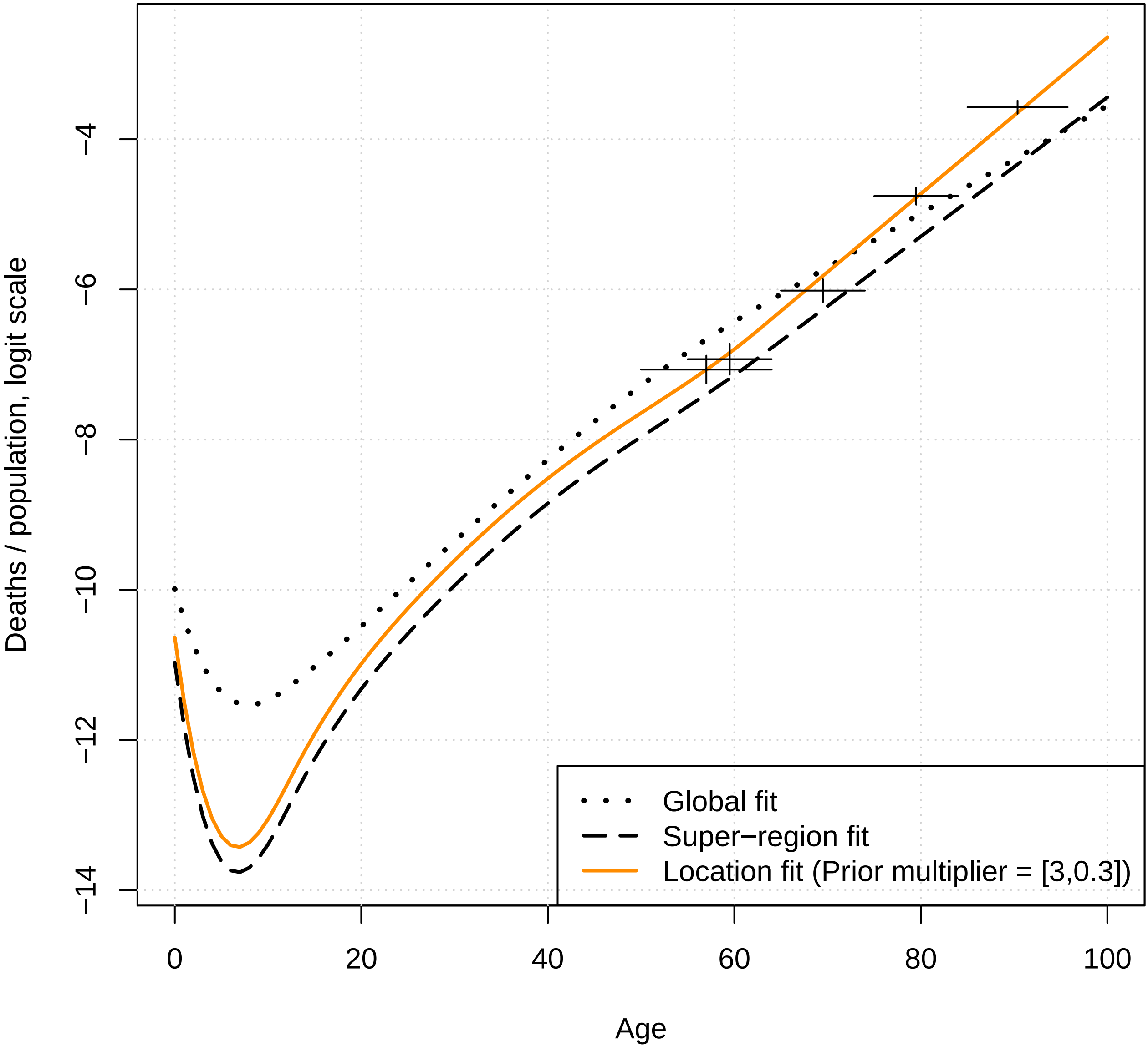

Cumulative reported deaths as of: 2021−01−02

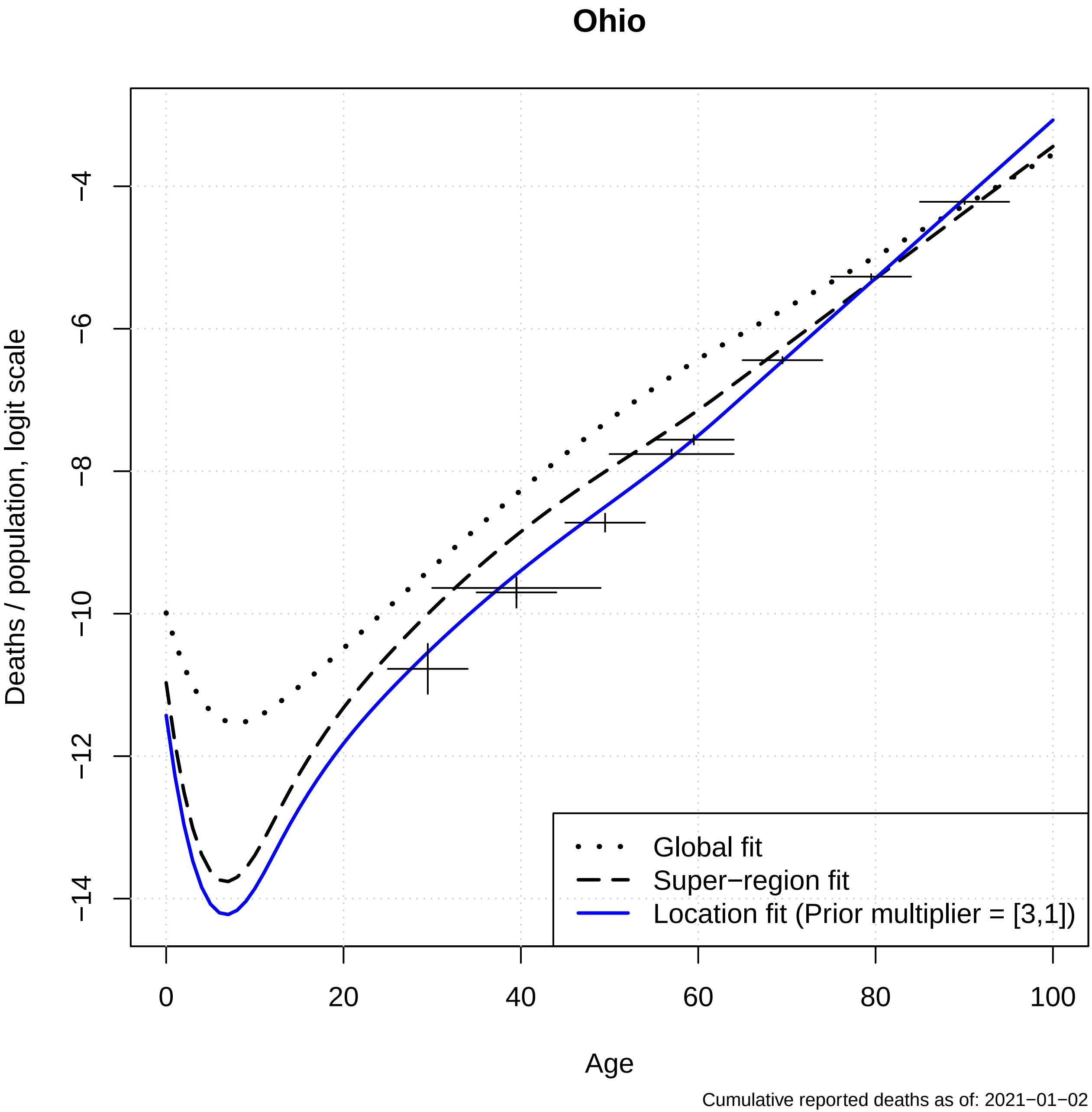

Ohio
Deaths / population, logit scale
−4
−6
−8
−10
−12
−14
0
20
40
60
80
100
Age
Global fit
Super−region fit
Location fit (Prior multiplier = [3,1])
Cumulative reported deaths as of: 2021−01−02

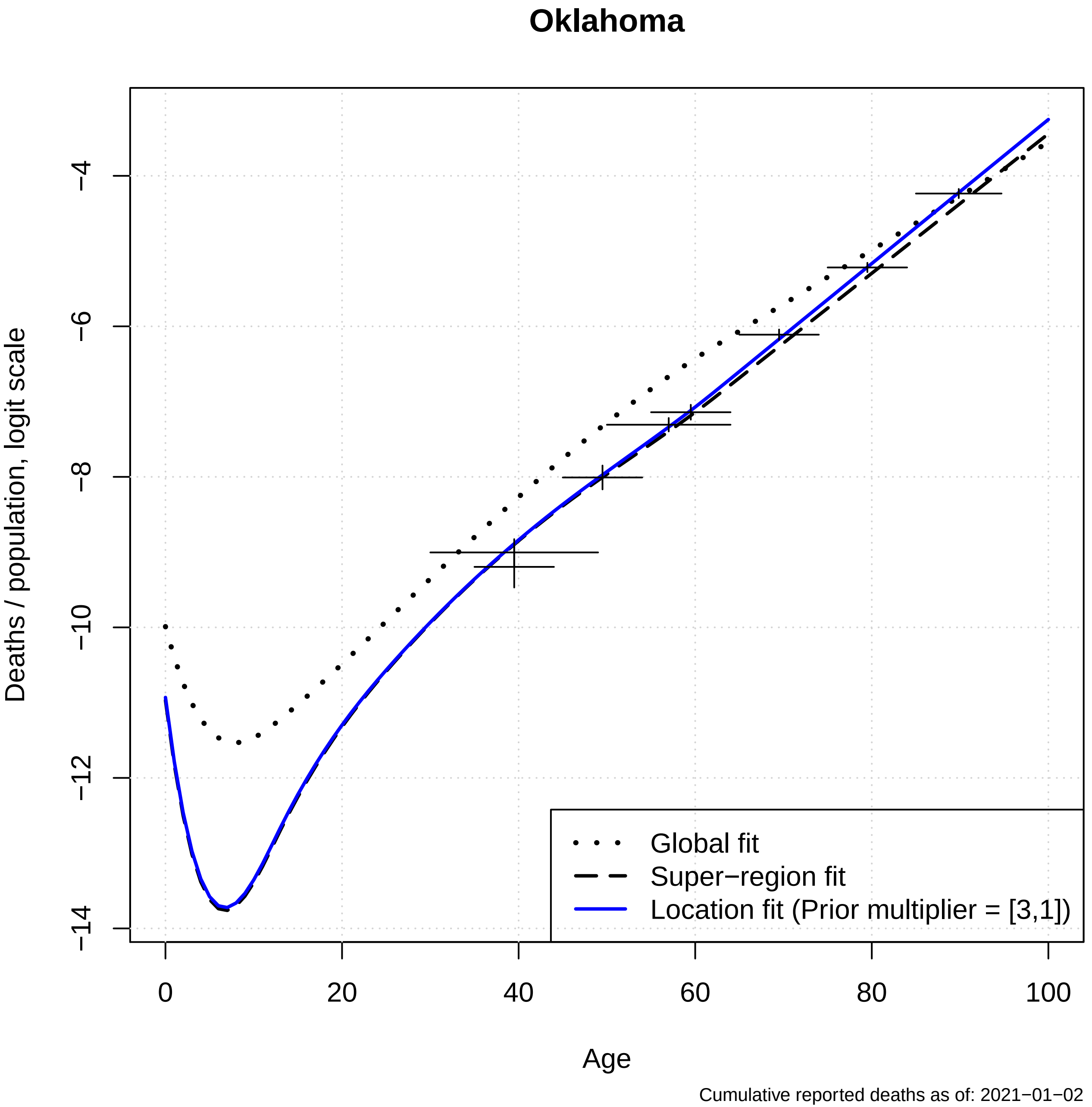
Oklahoma
Deaths / population, logit scale
−4
−6
−8
−10
−12
−14
0
20
40
60
80
100
Age
Global fit
Super−region fit
Location fit (Prior multiplier = [3,1])
Cumulative reported deaths as of: 2021−01−02

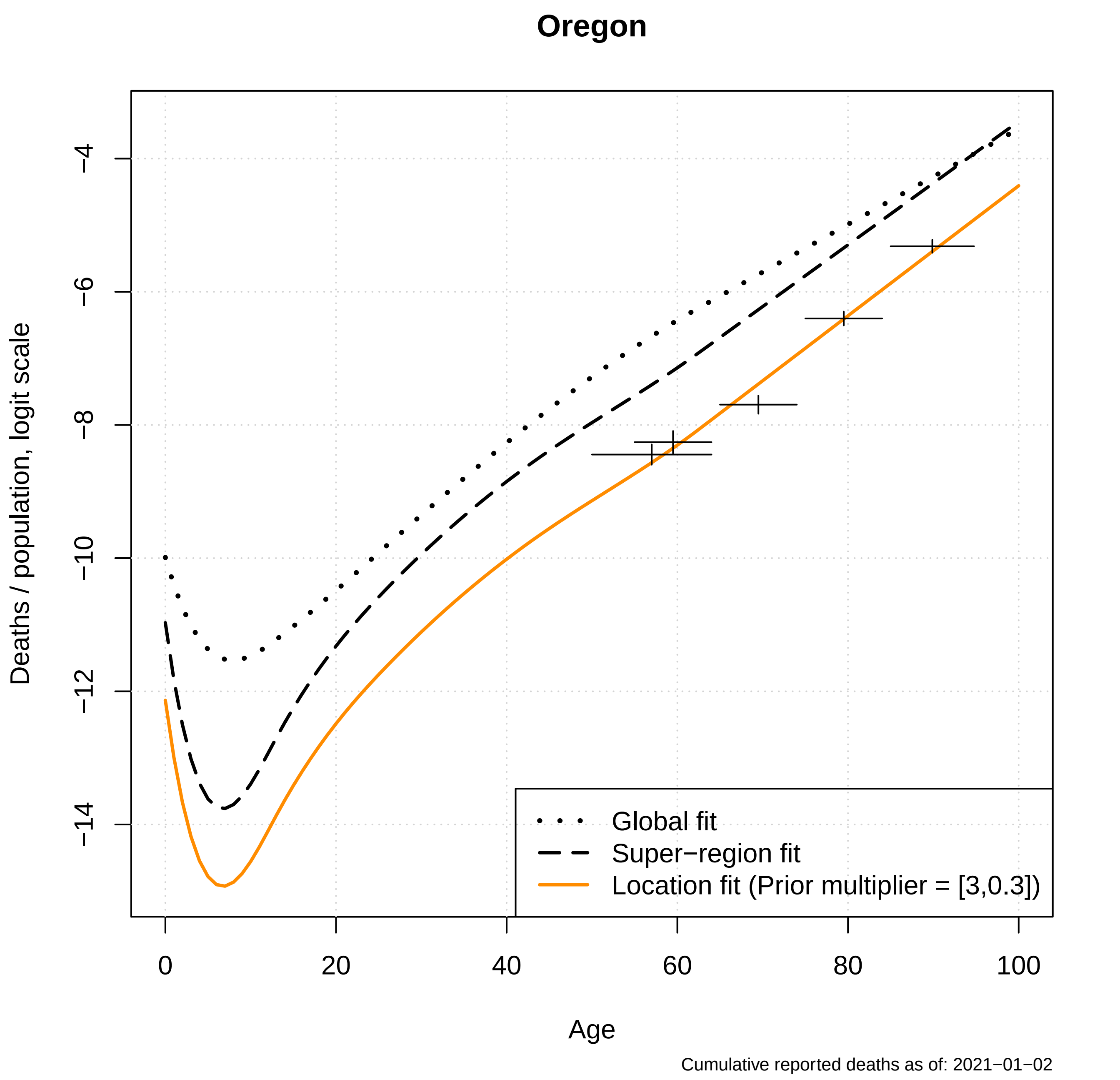
Oregon
Deaths / population, logit scale
−4
−6
−8
−10
−12
−14
0
20
40
60
80
100
Age
Global fit
Super−region fit
Location fit (Prior multiplier = [3,0.3])
Cumulative reported deaths as of: 2021−01−02

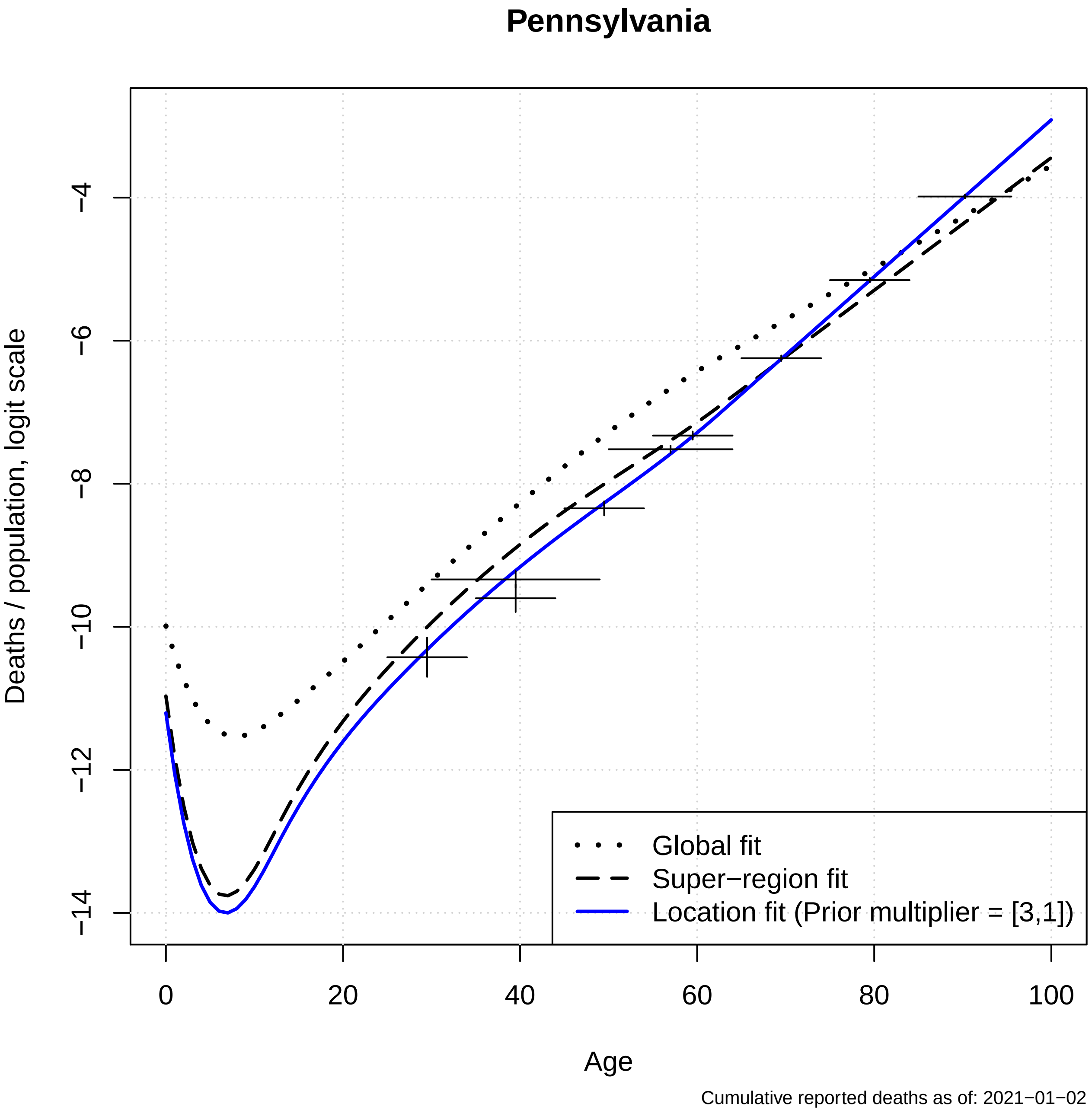
Pennsylvania
Deaths / population, logit scale
Age
−4
−6
−8
−10
−12
−14
0
20
40
60
80
100
Global fit
Super−region fit
Location fit (Prior multiplier = [3,1])
Cumulative reported deaths as of: 2021−01−02

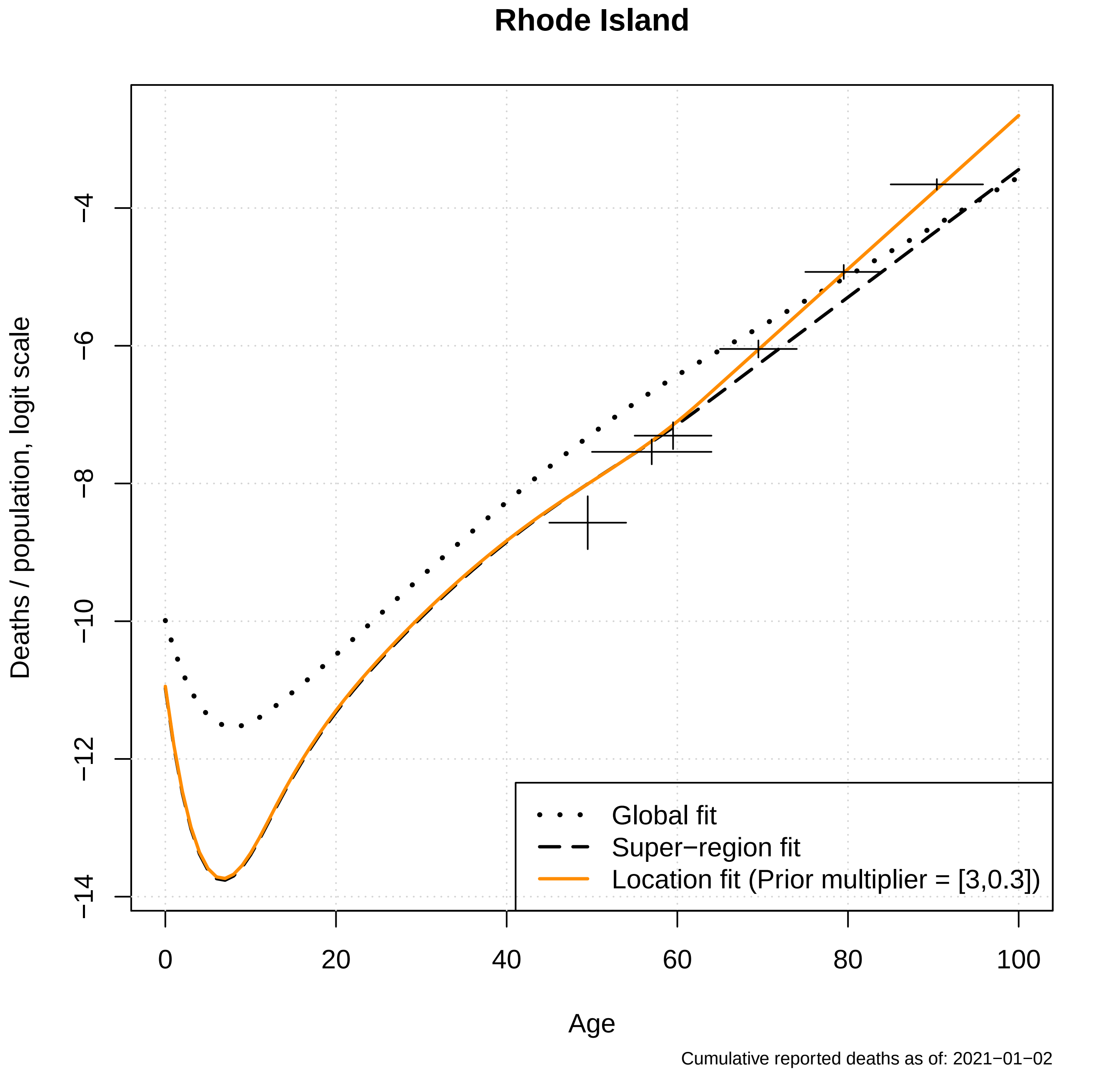

Rhode Island
Deaths / population, logit scale
−4
−6
−8
−10
−12
−14
0
20
40
60
80
100
Age
Global fit
Super−region fit
Location fit (Prior multiplier = [3,0.3])
Cumulative reported deaths as of: 2021−01−02

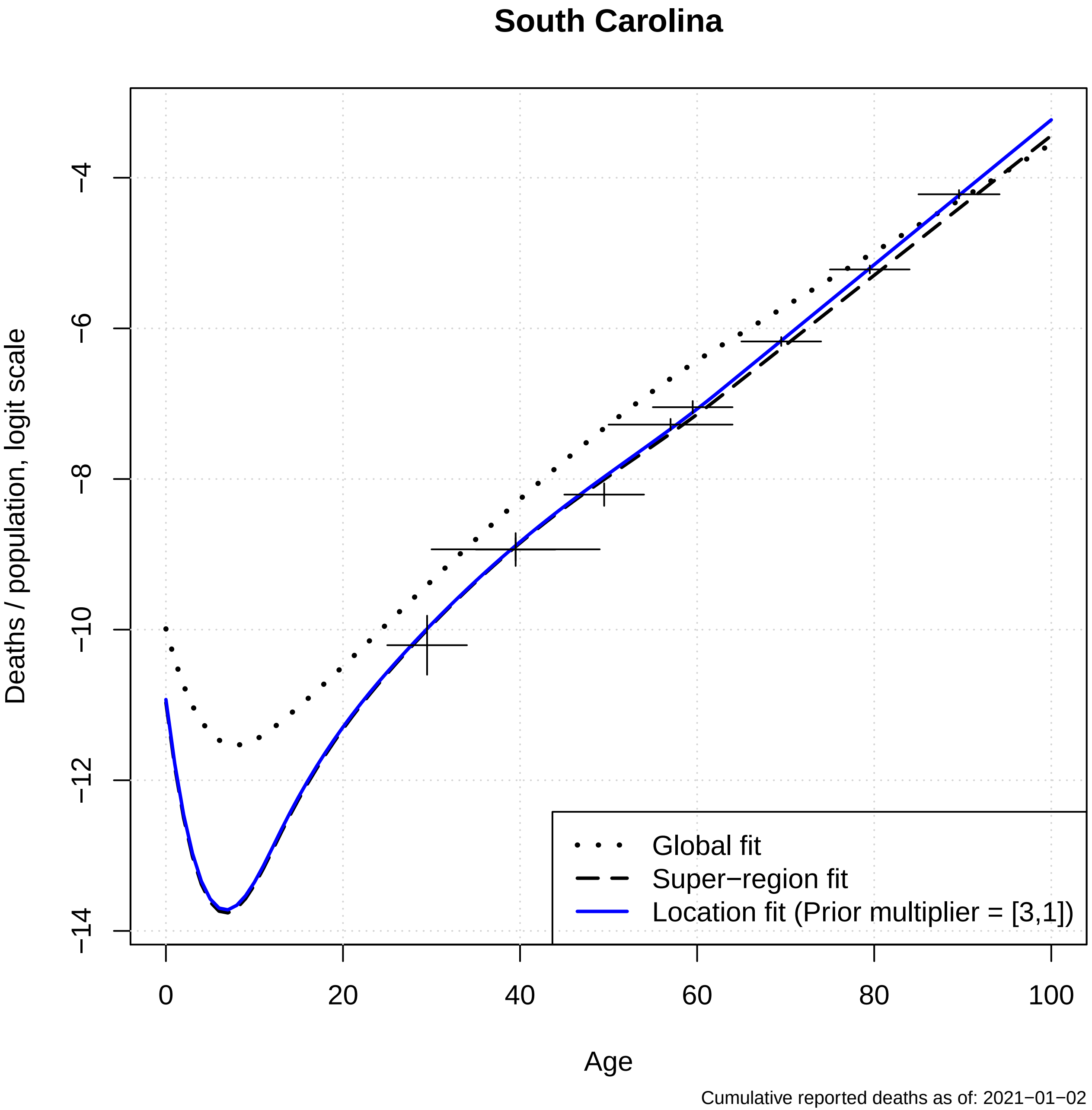
South Carolina
Deaths / population, logit scale
−4
−6
−8
−10
−12
−14
0
20
40
60
80
100
Age
Global fit
Super−region fit
Location fit (Prior multiplier = [3,1])
Cumulative reported deaths as of: 2021−01−02

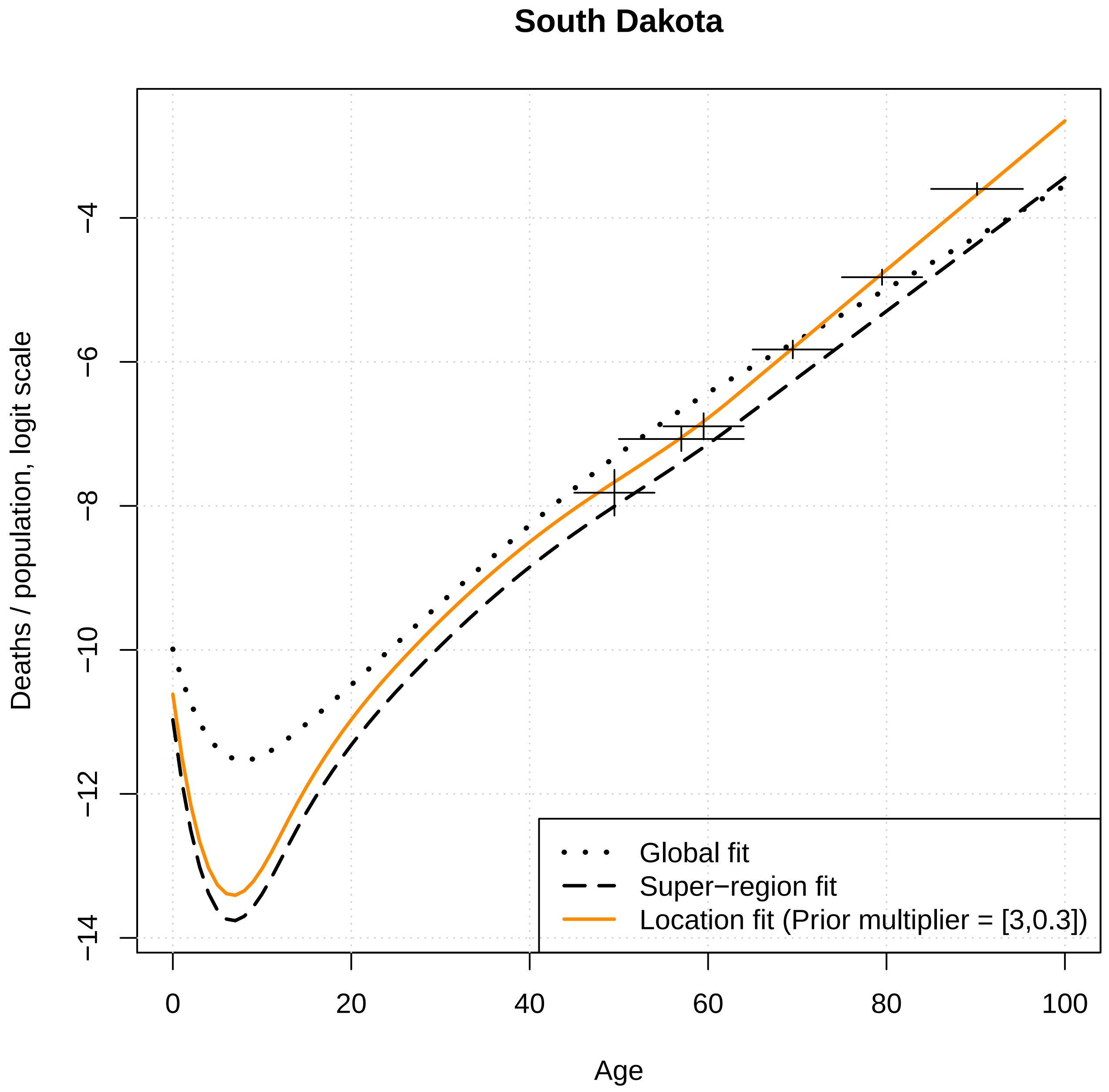

Cumulative reported deaths as of: 2021−01−02

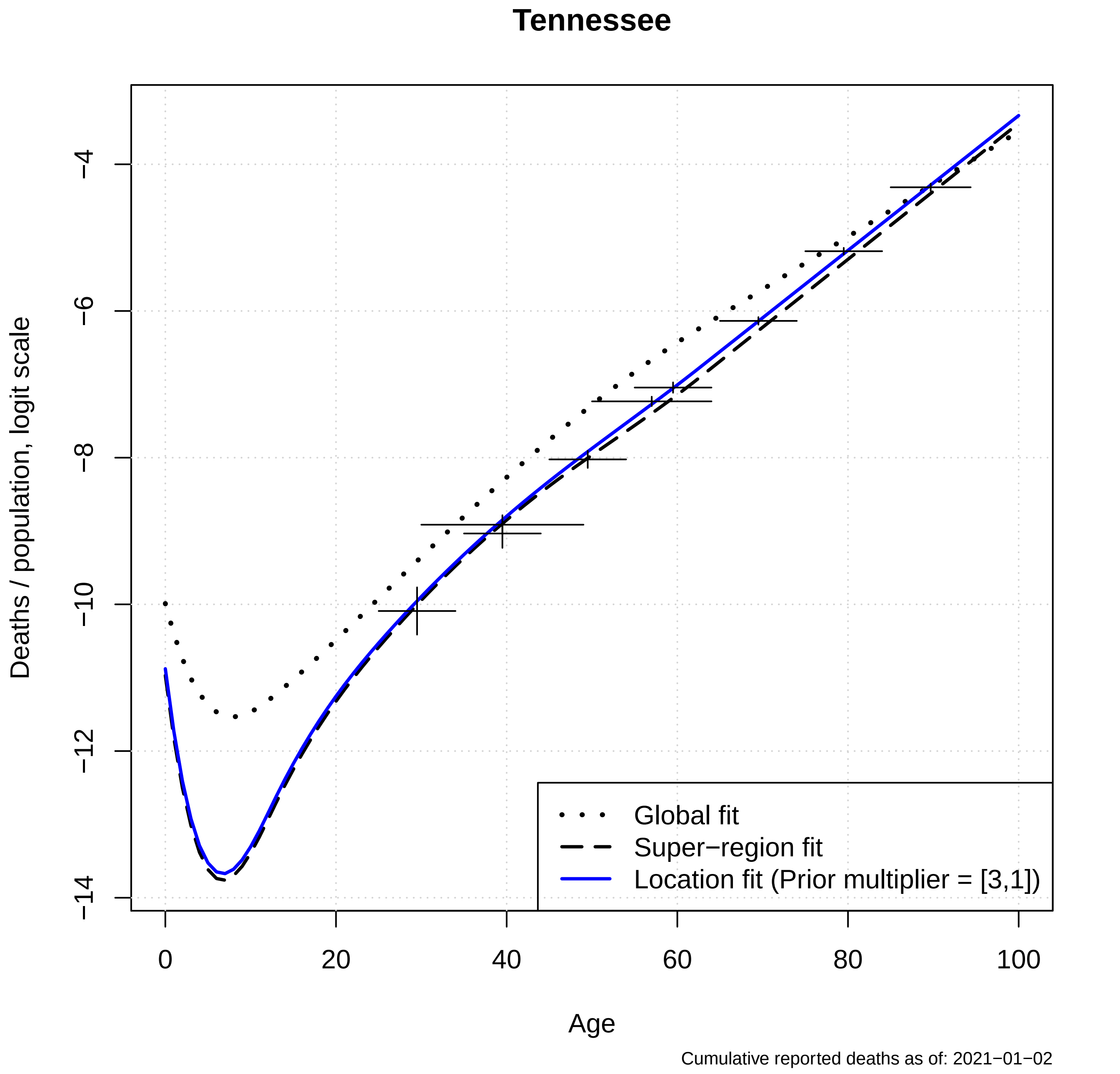

Tennessee
Deaths / population, logit scale
−4
−6
−8
−10
−12
−14
0
20
40
60
80
100
Age
Global fit
Super−region fit
Location fit (Prior multiplier = [3,1])
Cumulative reported deaths as of: 2021−01−02

Texas

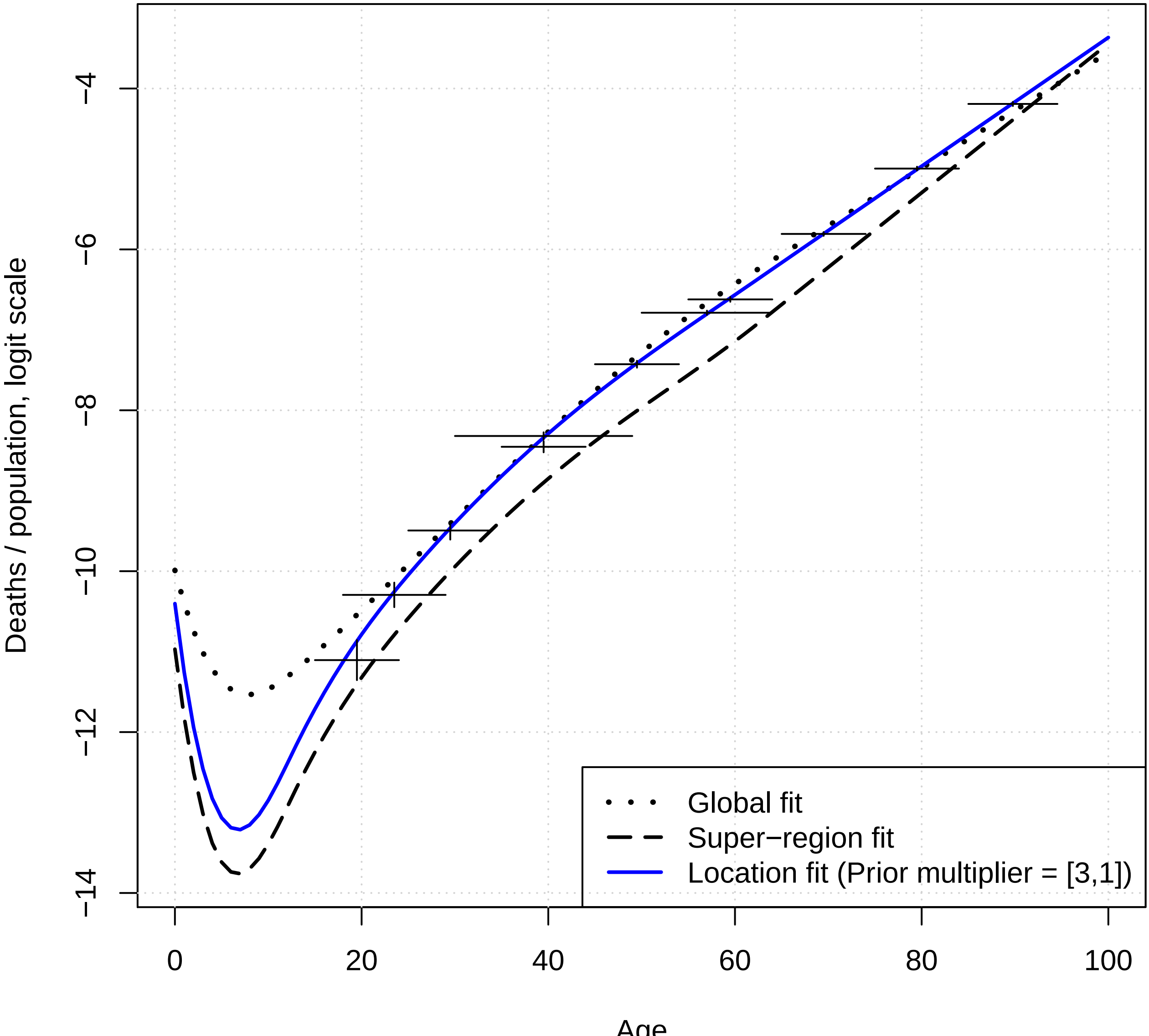

Cumulative reported deaths as of: 2021−01−02

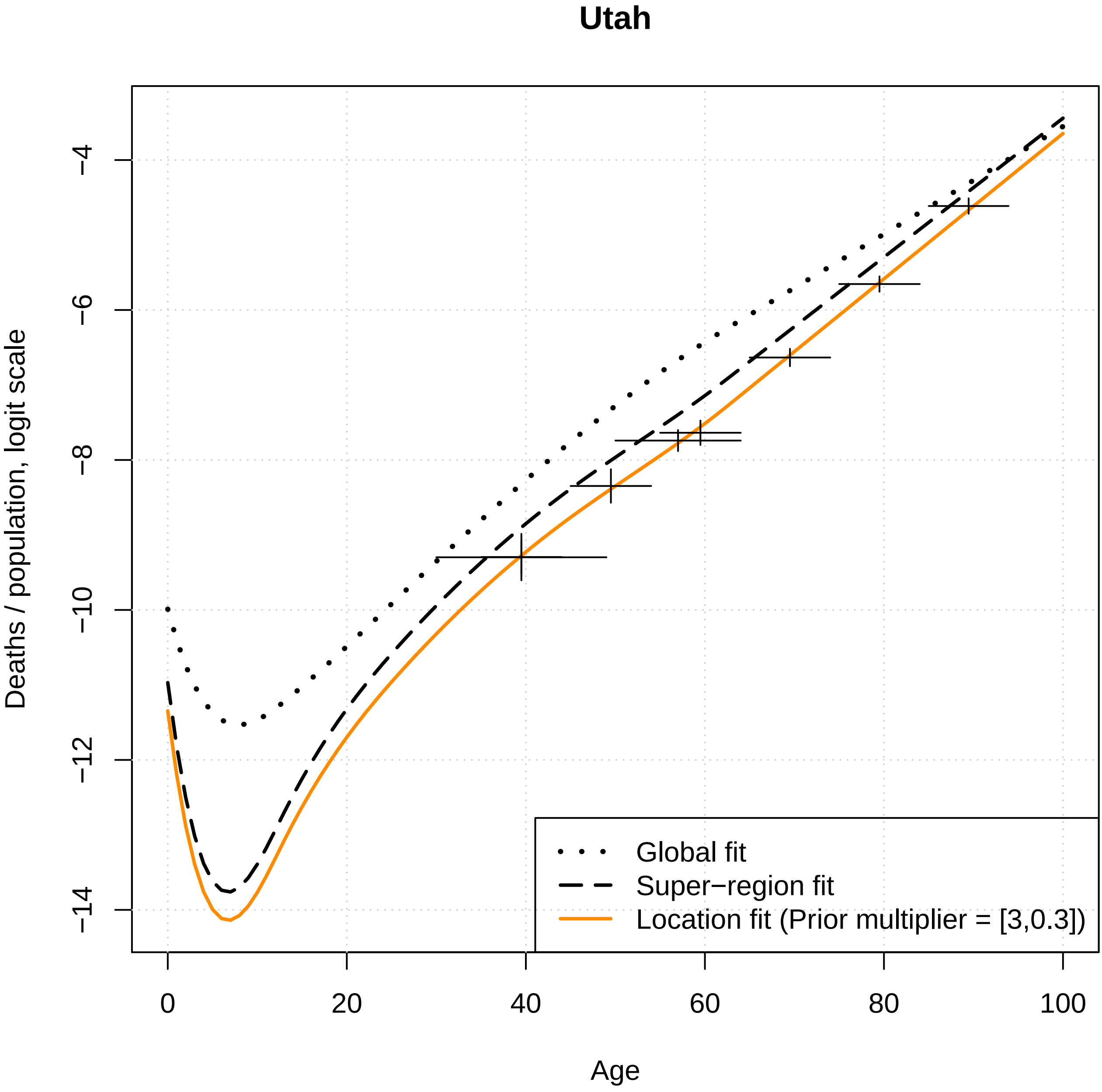

Cumulative reported deaths as of: 2021−01−02

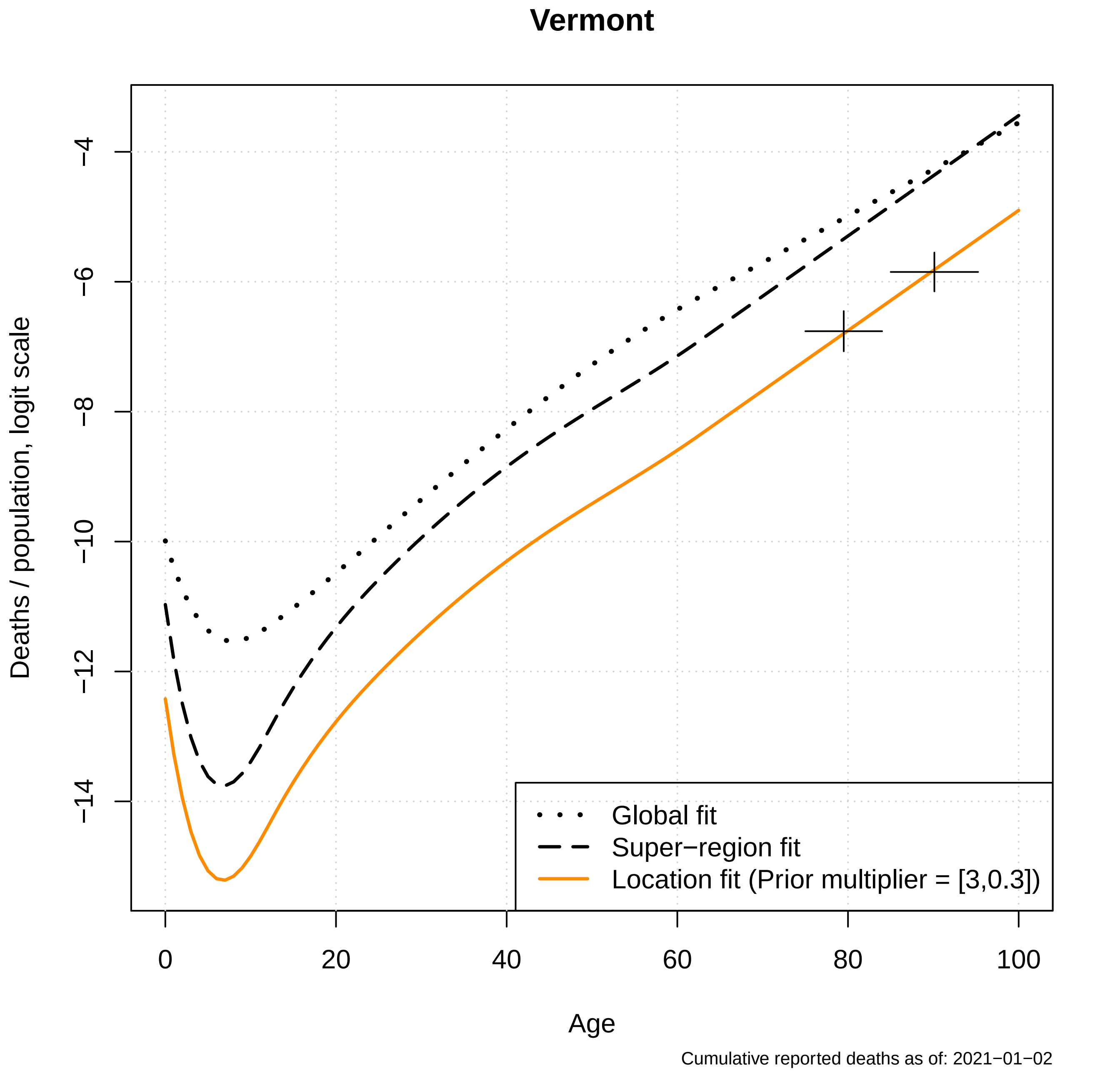

Vermont
Deaths / population, logit scale
−4
−6
−8
−10
−12
−14
0
20
40
60
80
100
Age
Global fit
Super−region fit
Location fit (Prior multiplier = [3,0.3])
Cumulative reported deaths as of: 2021−01−02

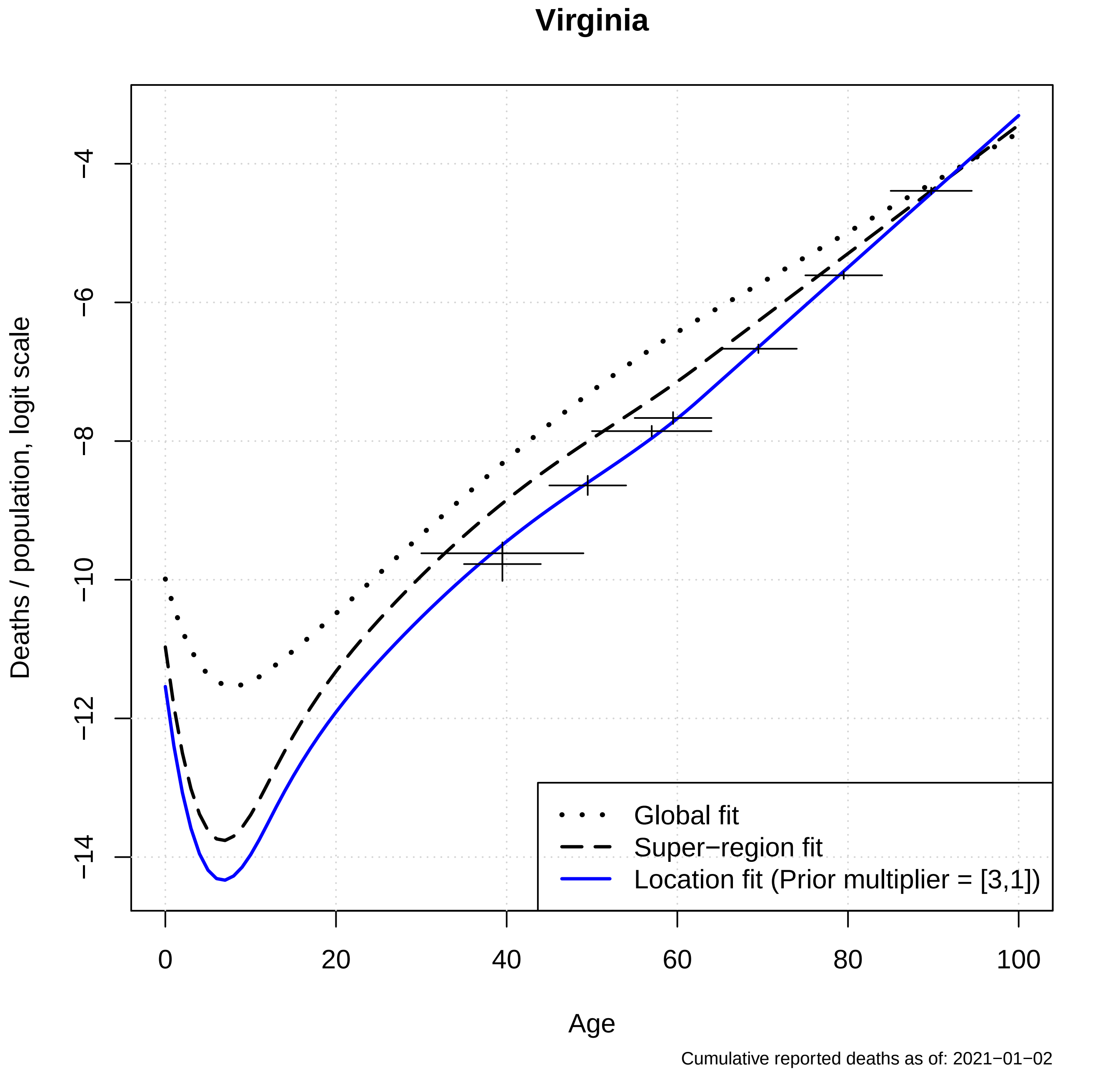
Virginia
Deaths / population, logit scale
−4
−6
−8
−10
−12
−14
0
20
40
60
80
100
Age
Global fit
Super−region fit
Location fit (Prior multiplier = [3,1])
Cumulative reported deaths as of: 2021−01−02

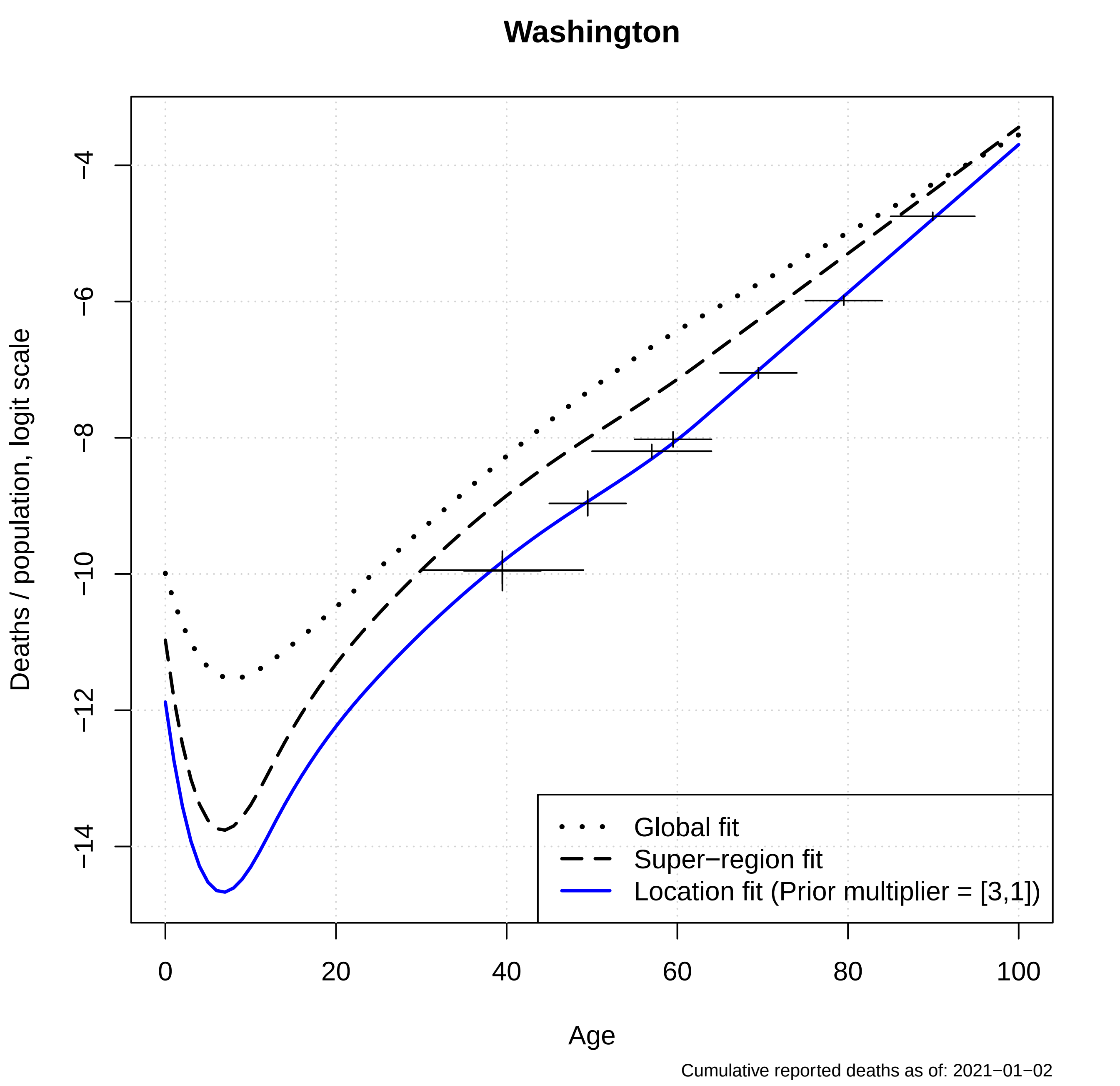

Washington
Deaths / population, logit scale
−4
−6
−8
−10
−12
−14
0
20
40
60
80
100
Age
Global fit
Super−region fit
Location fit (Prior multiplier = [3,1])
Cumulative reported deaths as of: 2021−01−02

West Virginia

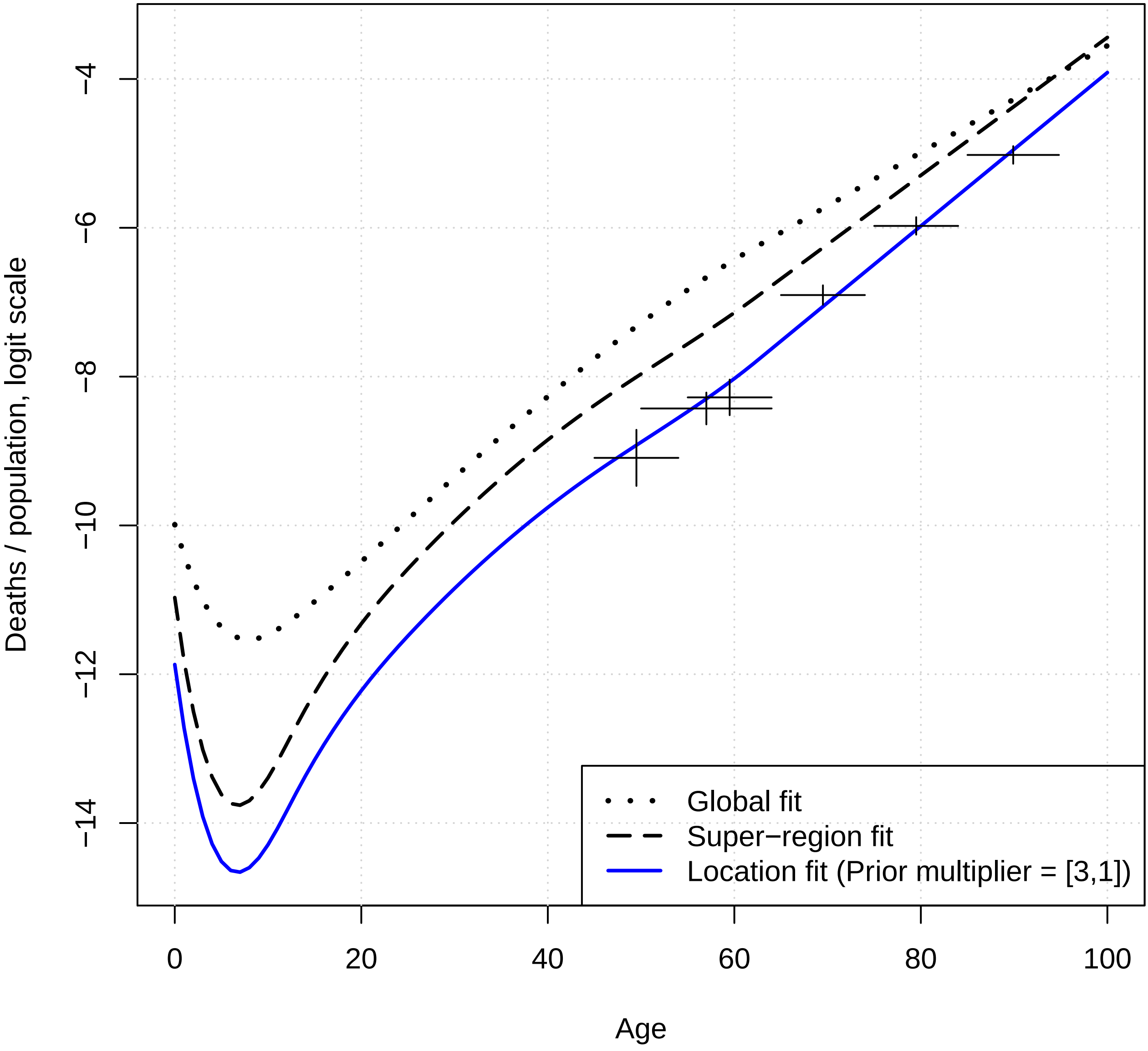

Cumulative reported deaths as of: 2021−01−02

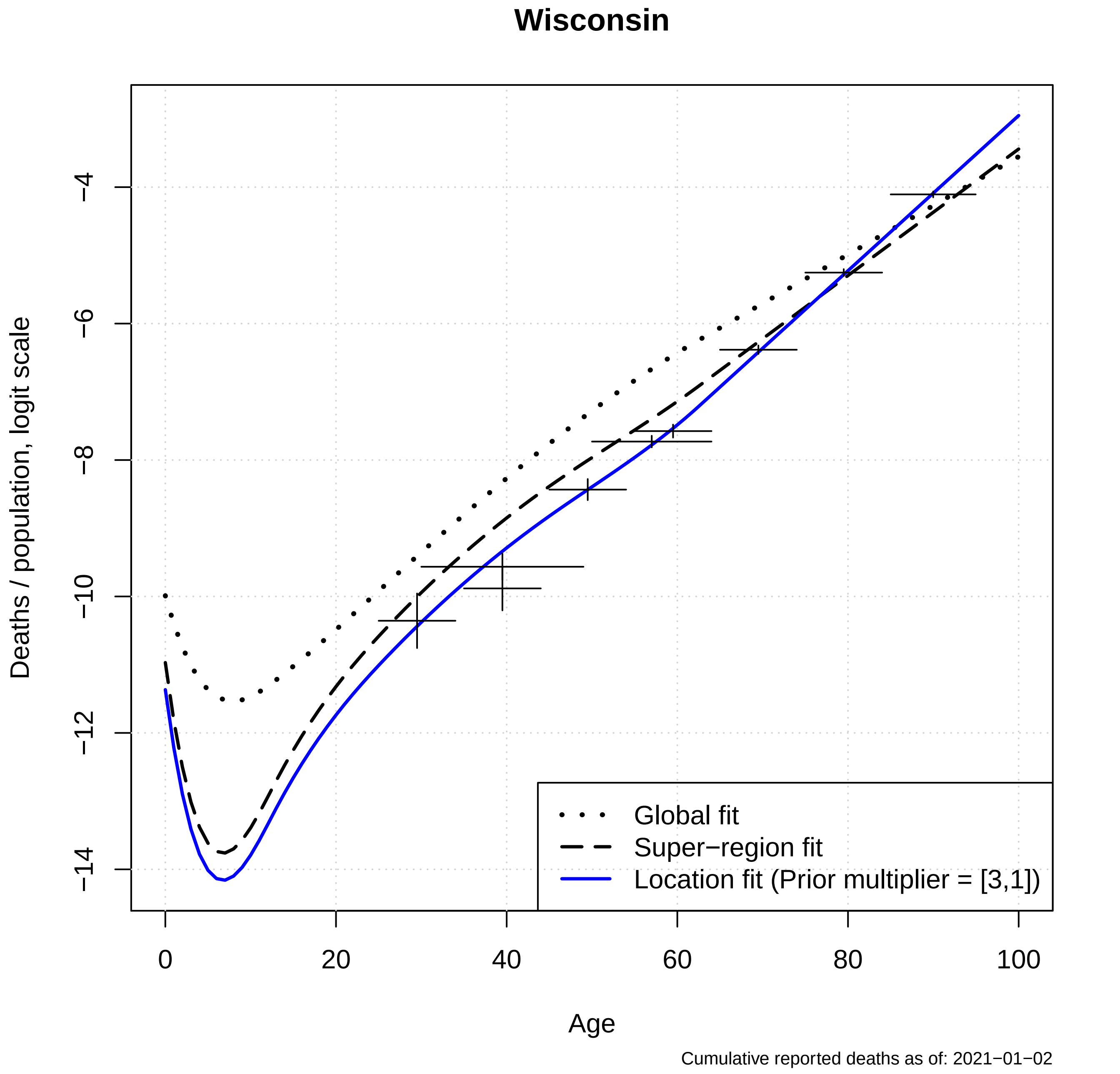
Wisconsin
Deaths / population, logit scale
−4
−6
−8
−10
−12
−14
0
20
40
60
80
100
Age
Global fit
Super−region fit
Location fit (Prior multiplier = [3,1])
Cumulative reported deaths as of: 2021−01−02

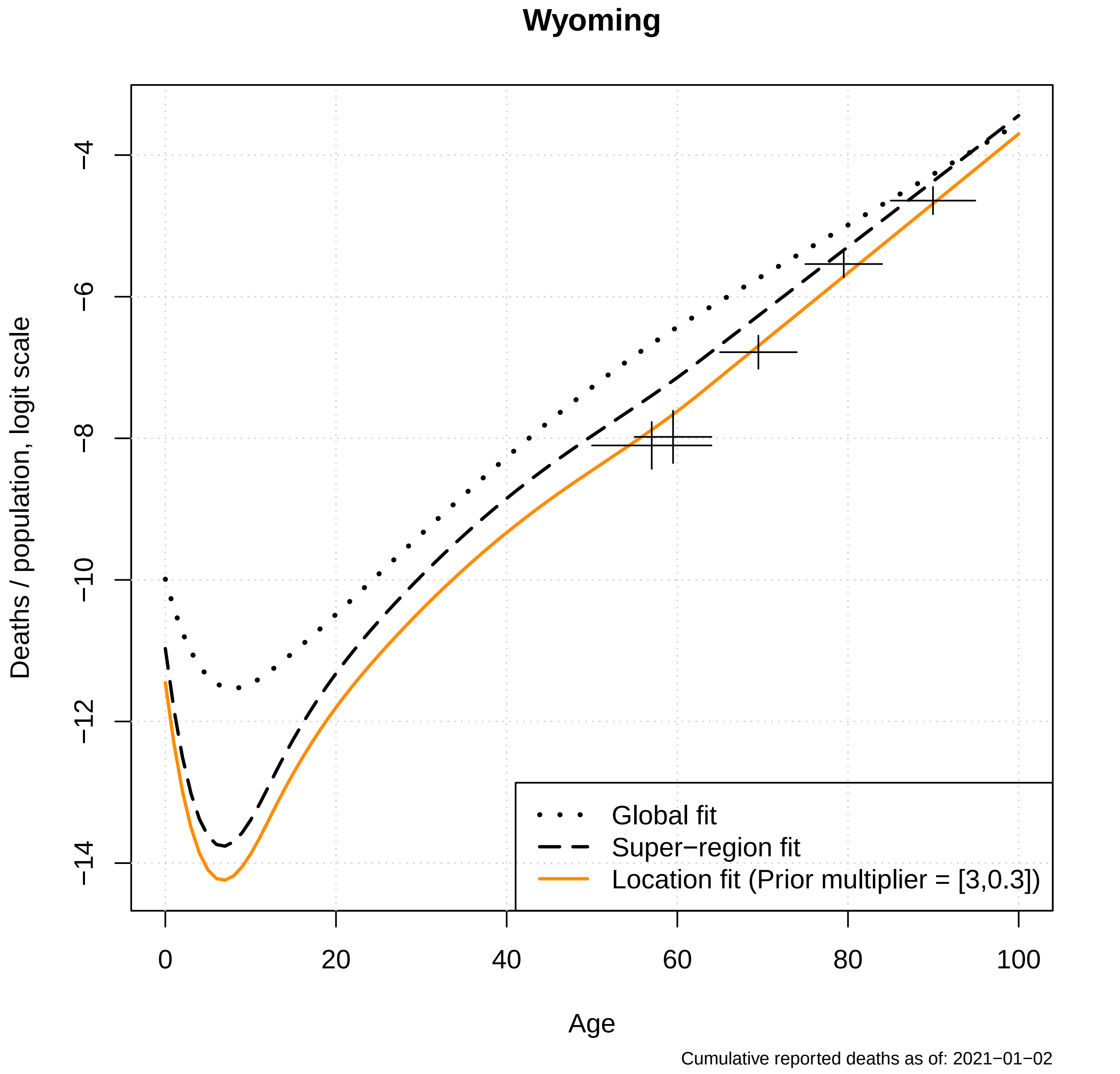
Wyoming
Deaths / population, logit scale
−4
−6
−8
−10
−12
−14
0
20
40
60
80
100
Age
Global fit
Super−region fit
Location fit (Prior multiplier = [3,0.3])
Cumulative reported deaths as of: 2021−01−02

Figure S8D. Mexico subnationals

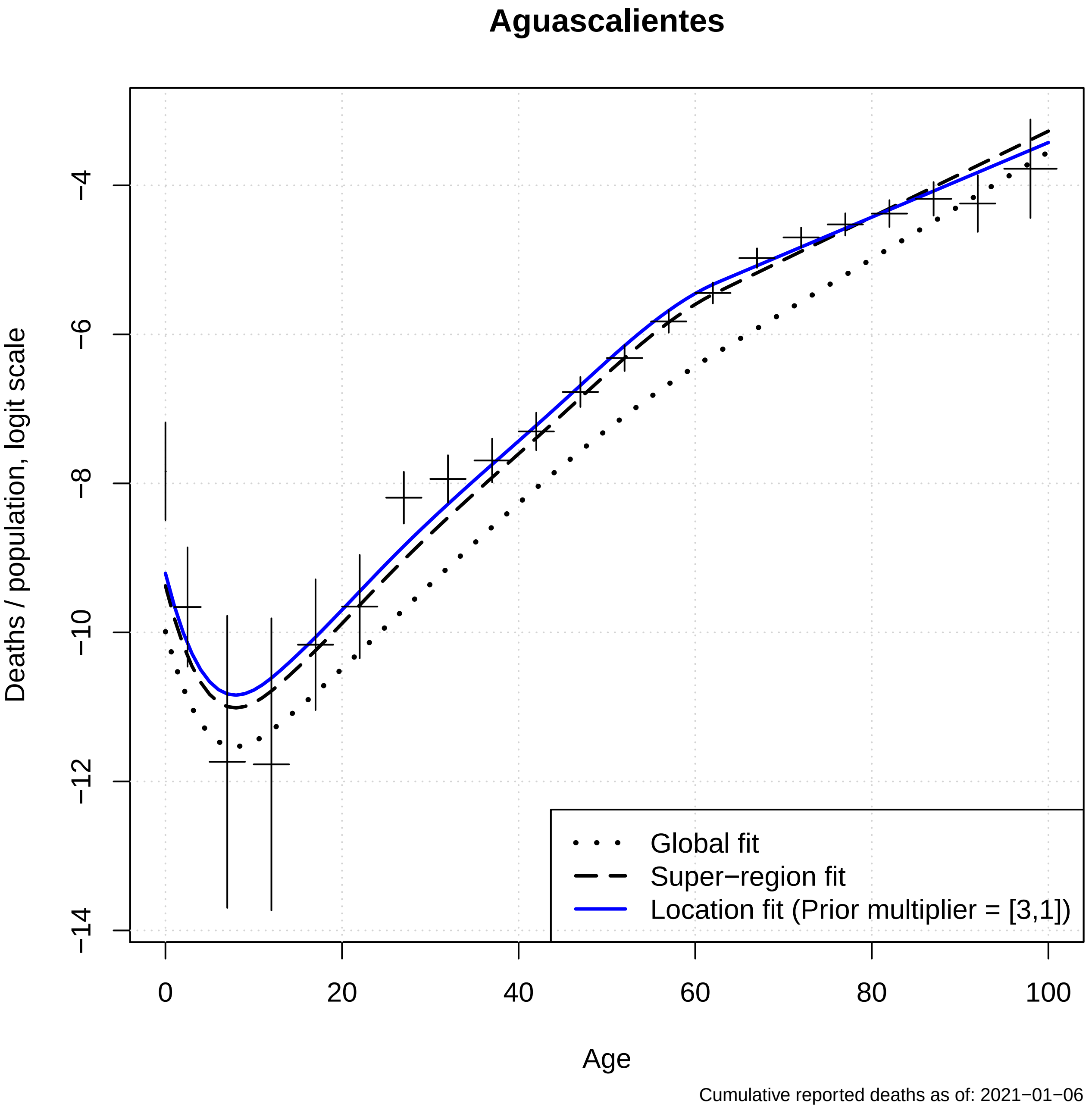

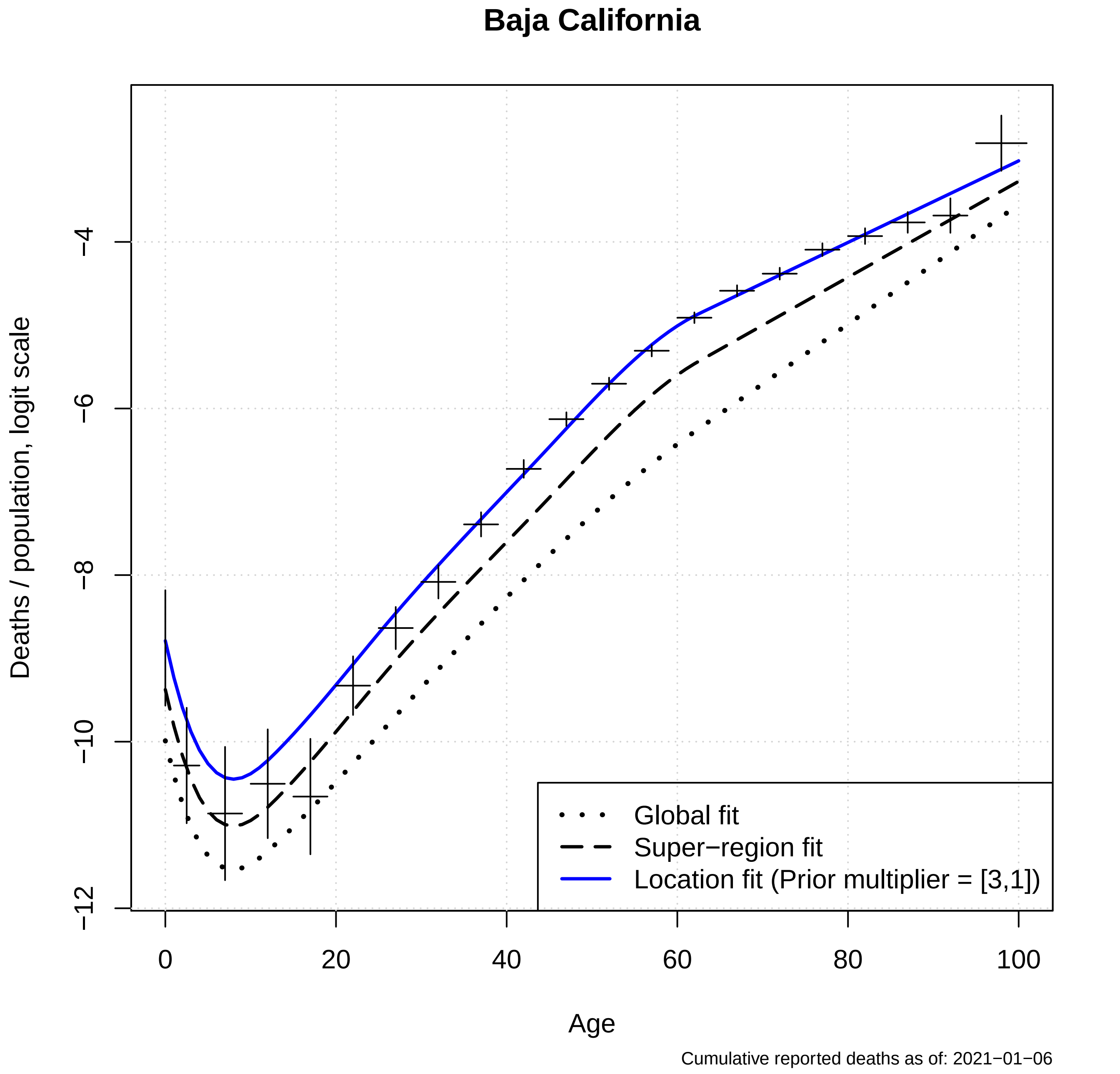
Baja California
Deaths / population, logit scale
−12
−10
−8
−6
−4
0
20
40
60
80
100
Age
Global fit
Super−region fit
Location fit (Prior multiplier = [3,1])
Cumulative reported deaths as of: 2021−01−06

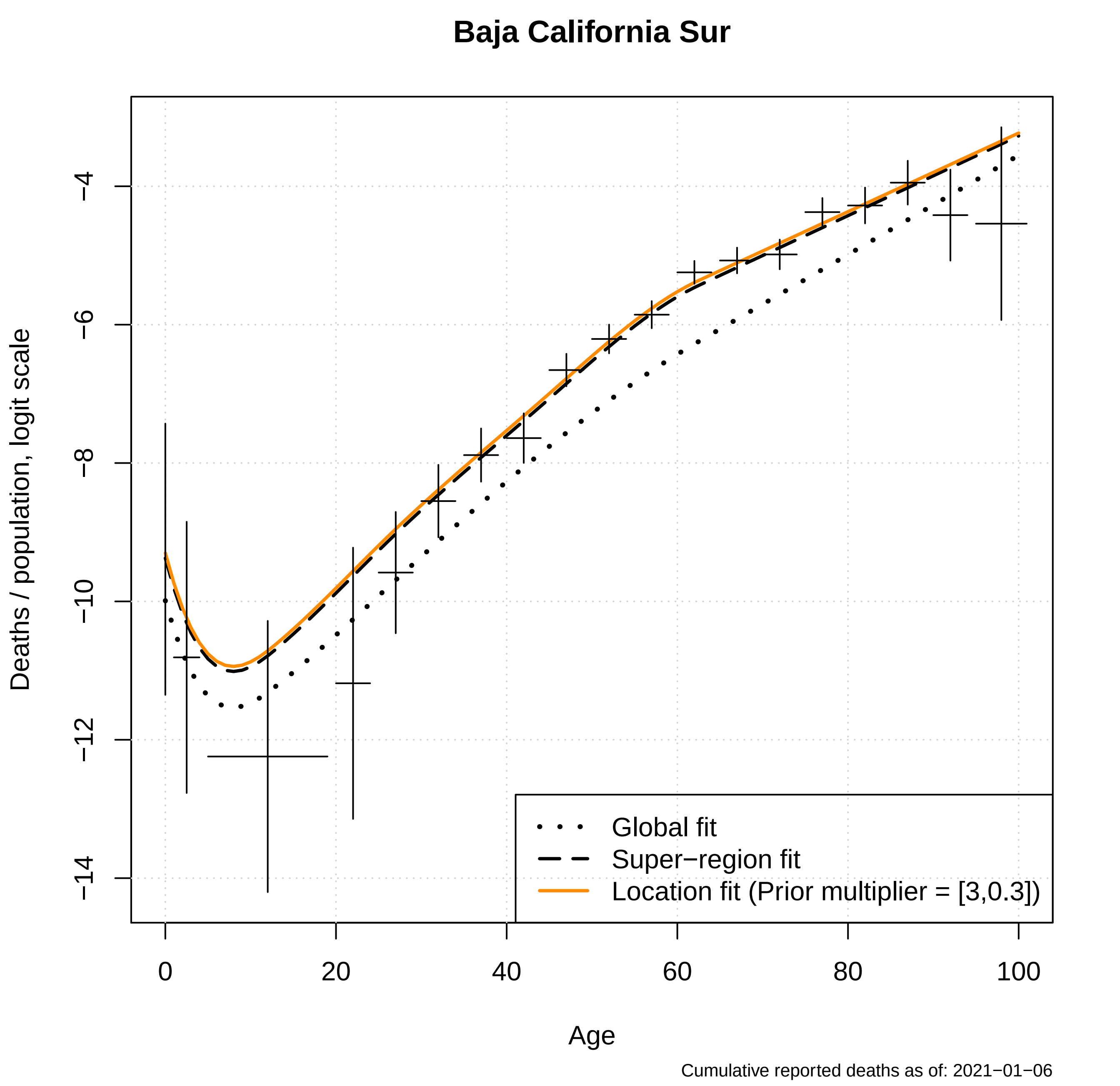
Baja California Sur
Deaths / population, logit scale
−4
−6
−8
−10
−12
−14
0
20
40
60
80
100
Age
Global fit
Super−region fit
Location fit (Prior multiplier = [3,0.3])
Cumulative reported deaths as of: 2021−01−06

Campeche

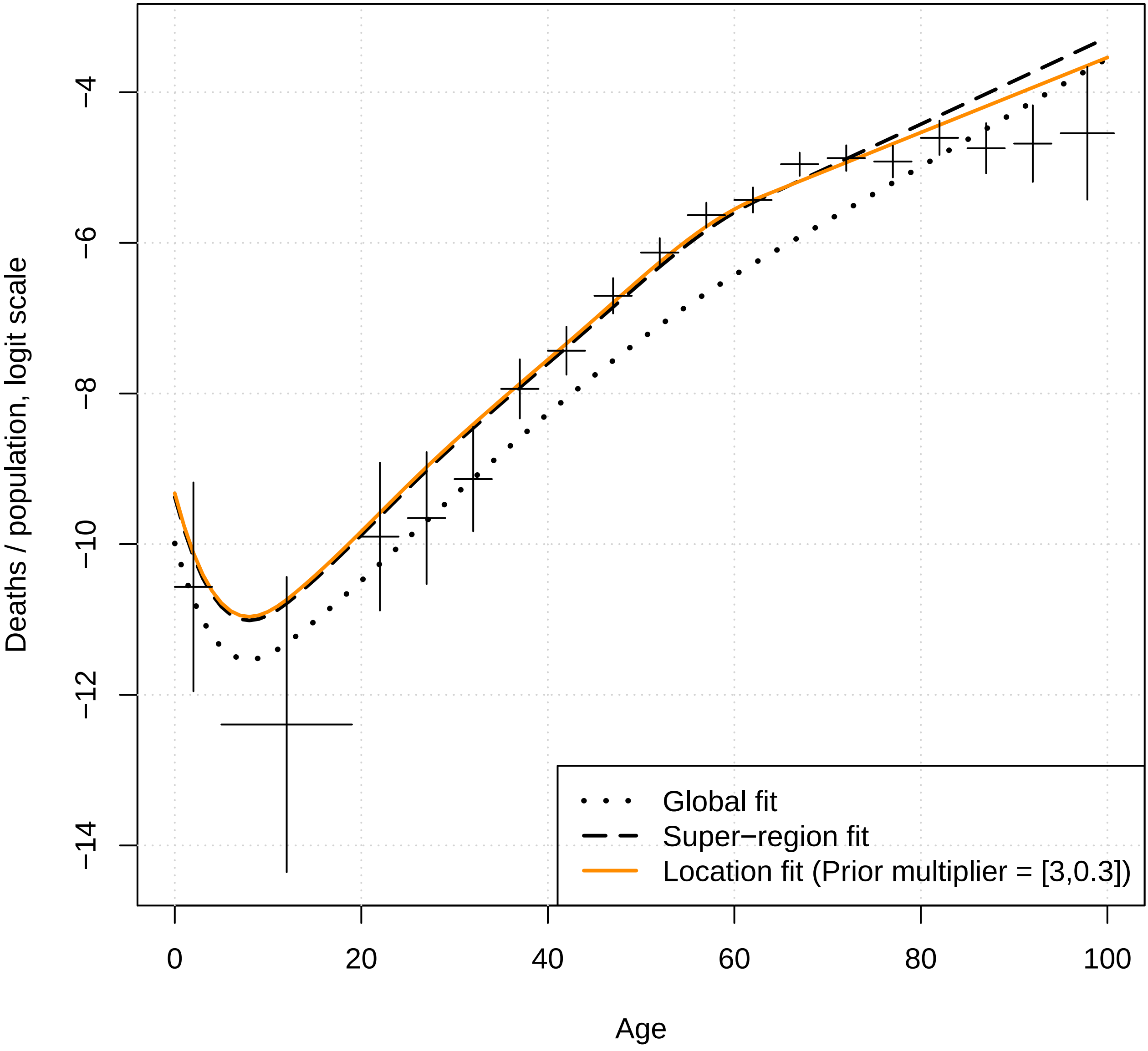

Cumulative reported deaths as of: 2021−01−06

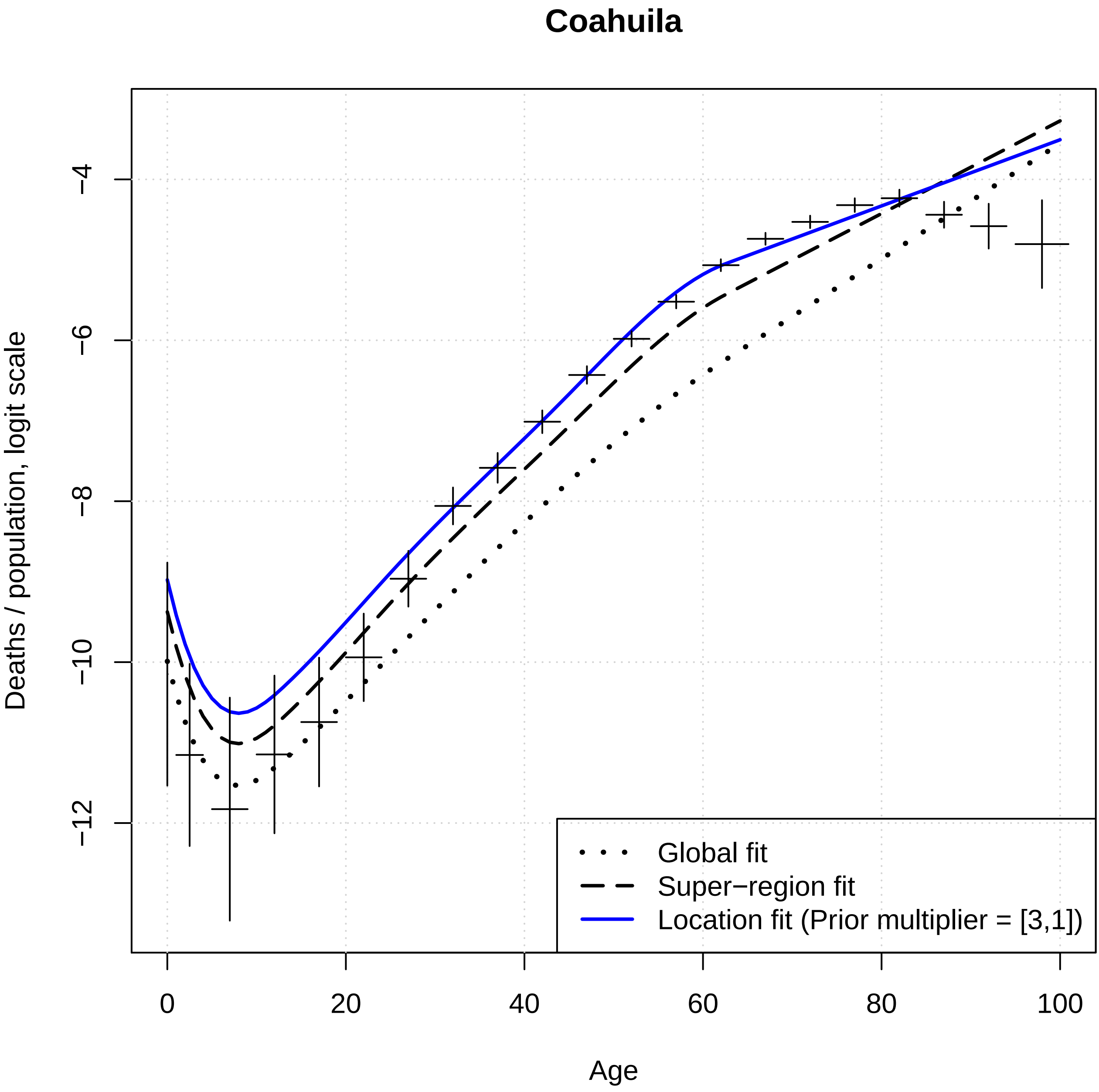

Cumulative reported deaths as of: 2021−01−06

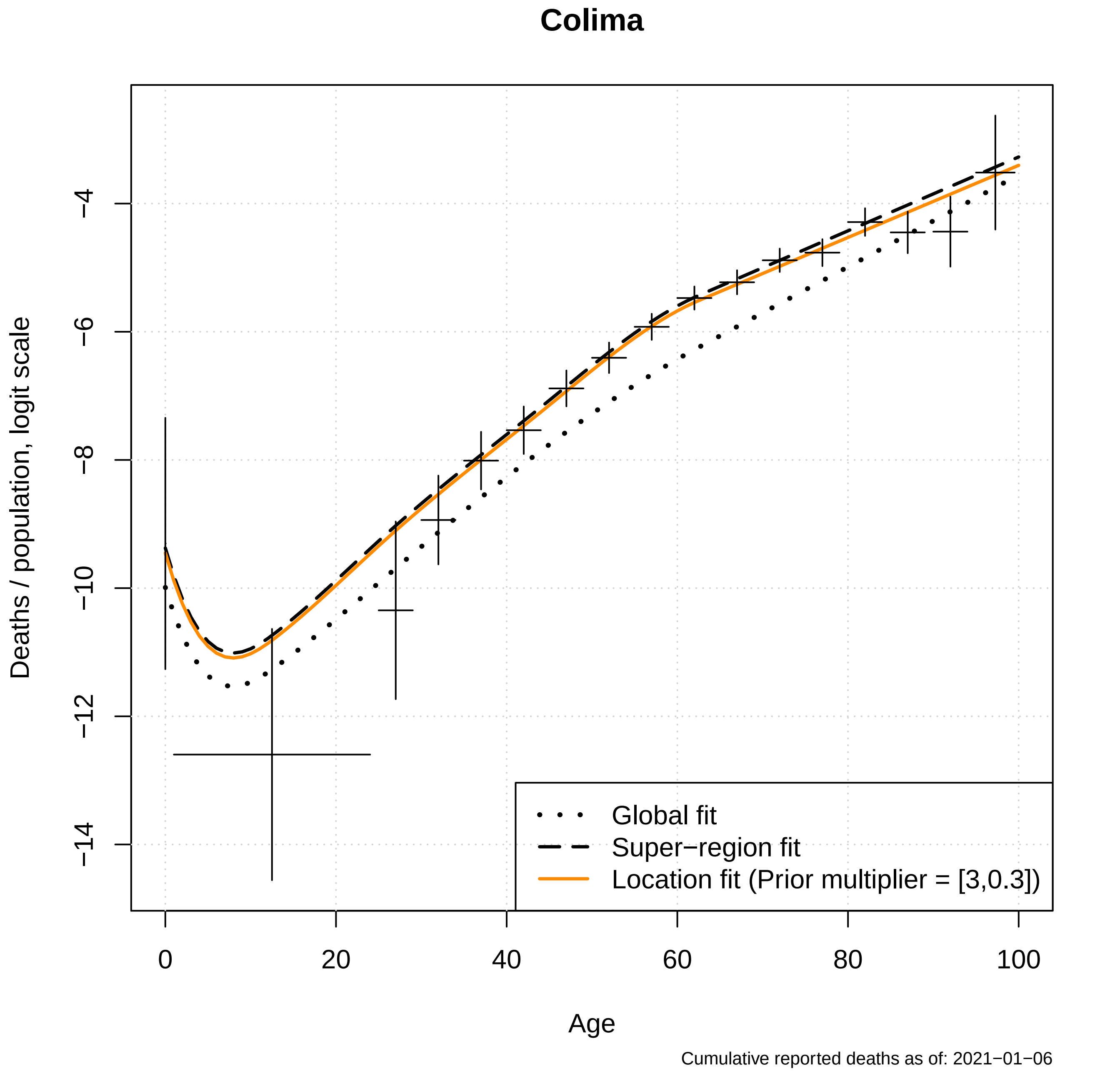

Colima
Deaths / population, logit scale
−4
−6
−8
−10
−12
−14
0
20
40
60
80
100
Age
Global fit
Super−region fit
Location fit (Prior multiplier = [3,0.3])
Cumulative reported deaths as of: 2021−01−06

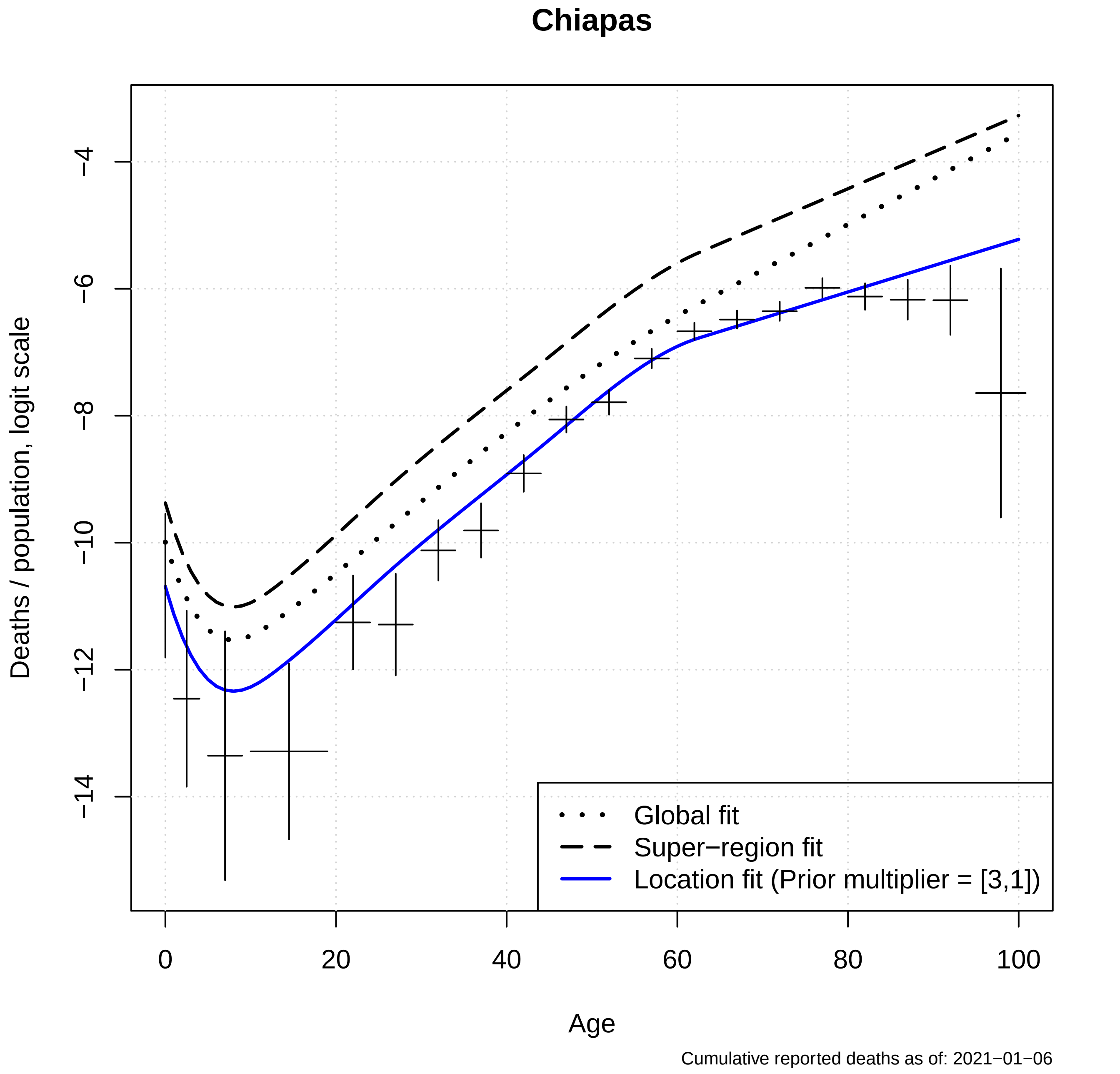

Chiapas
−4
−6
−8
−10
−12
−14
Deaths / population, logit scale
0
20
40
60
80
100
Age
Global fit
Super−region fit
Location fit (Prior multiplier = [3,1])
Cumulative reported deaths as of: 2021−01−06

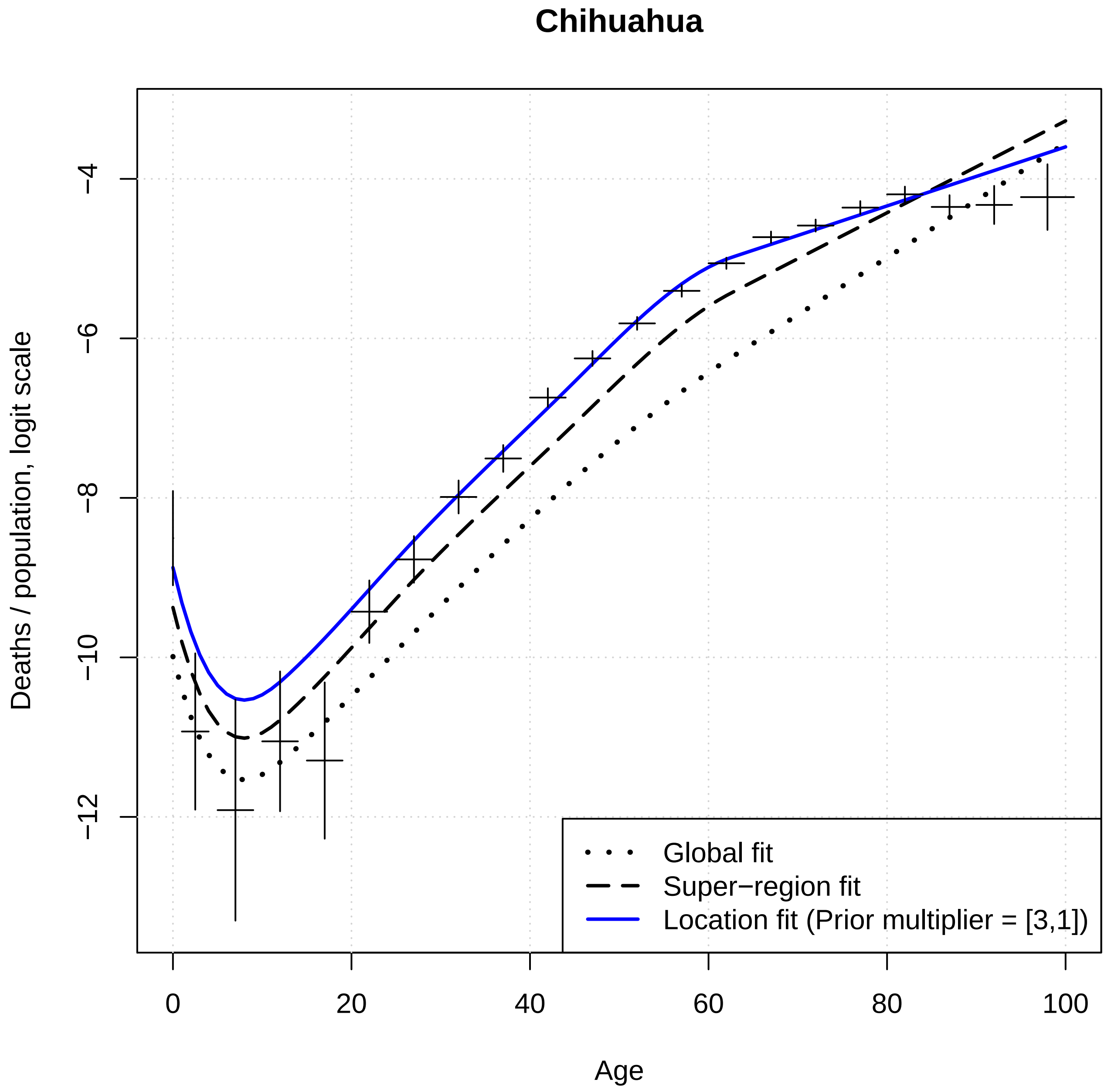

Cumulative reported deaths as of: 2021−01−06

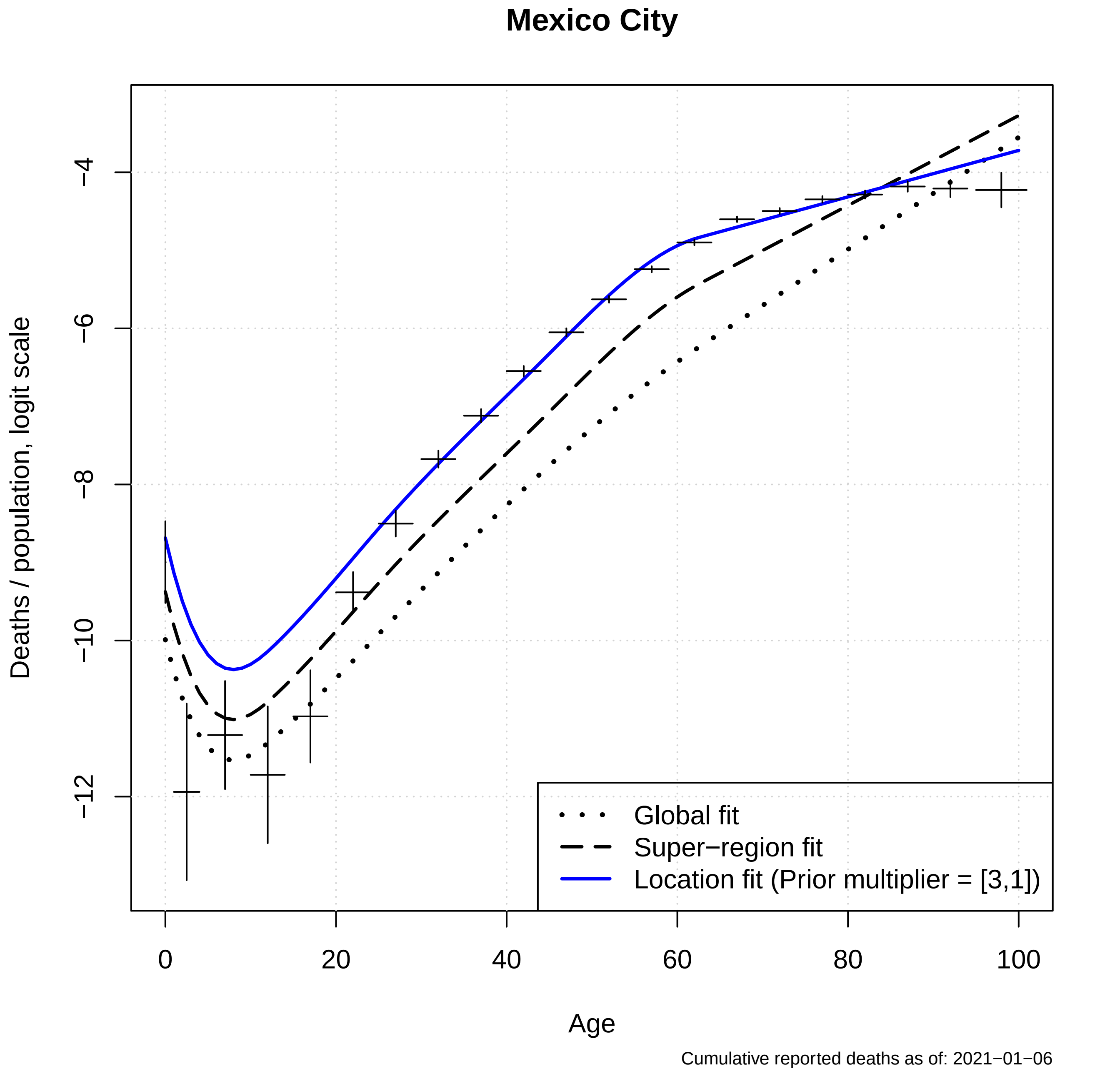

Mexico City
Deaths / population, logit scale
−4
−6
−8
−10
−12
0
20
40
60
80
100
Age
Global fit
Super−region fit
Location fit (Prior multiplier = [3,1])
Cumulative reported deaths as of: 2021−01−06

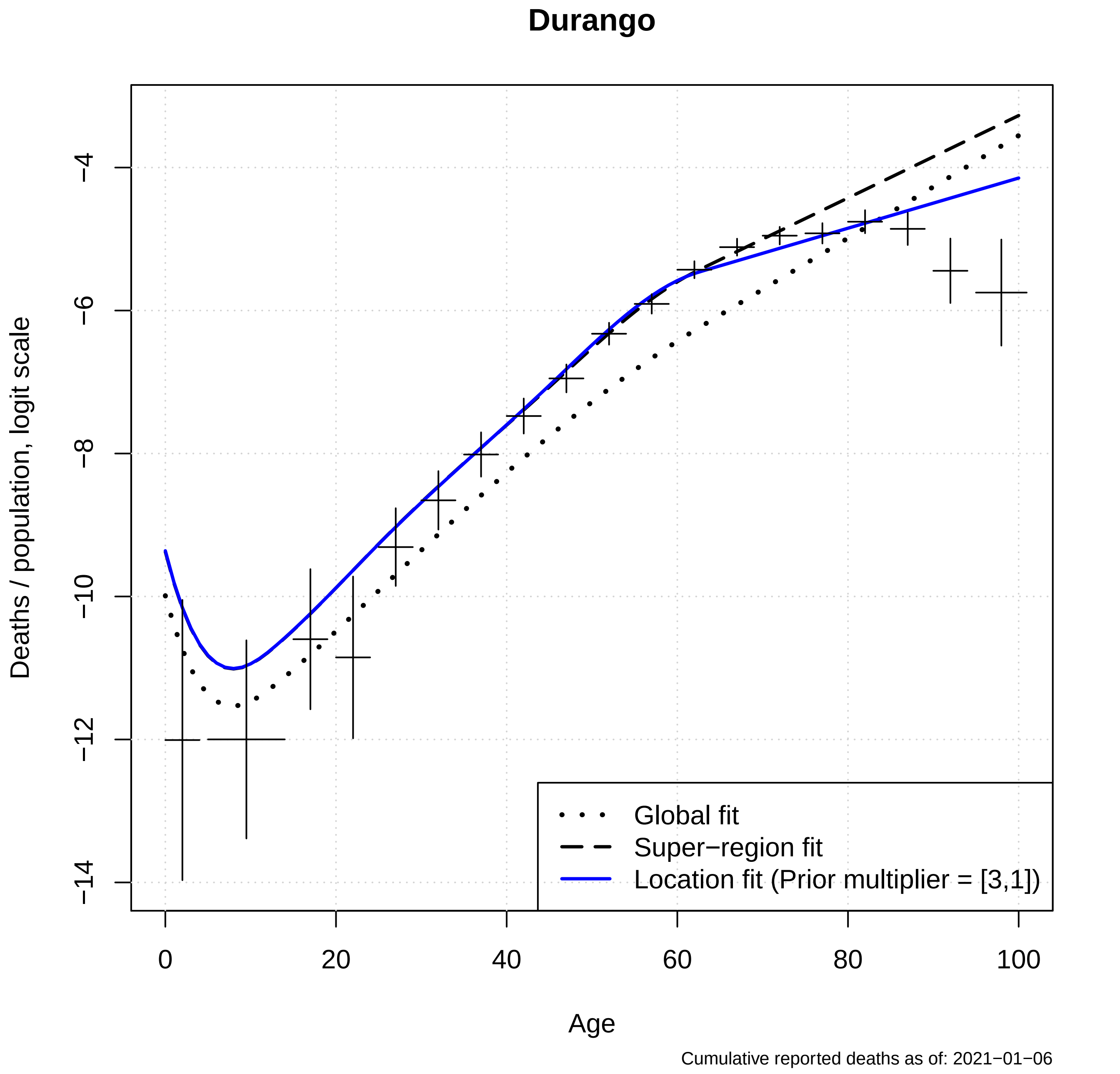

Durango
Deaths / population, logit scale
−4
−6
−8
−10
−12
−14
0
20
40
60
80
100
Age
Global fit
Super−region fit
Location fit (Prior multiplier = [3,1])
Cumulative reported deaths as of: 2021−01−06

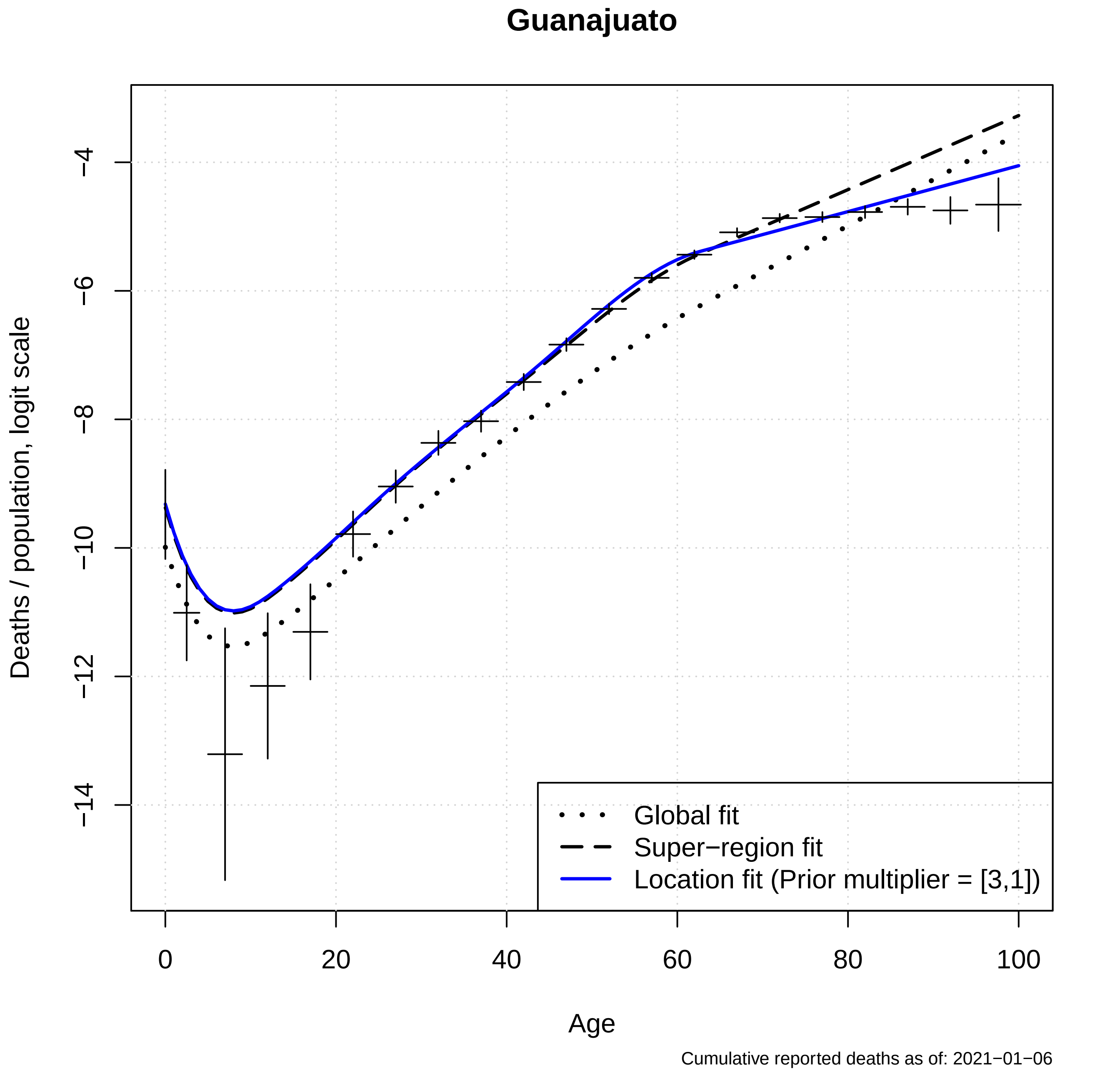

Guanajuato
Deaths / population, logit scale
−4
−6
−8
−10
−12
−14
0
20
40
60
80
100
Age
Global fit
Super−region fit
Location fit (Prior multiplier = [3,1])
Cumulative reported deaths as of: 2021−01−06

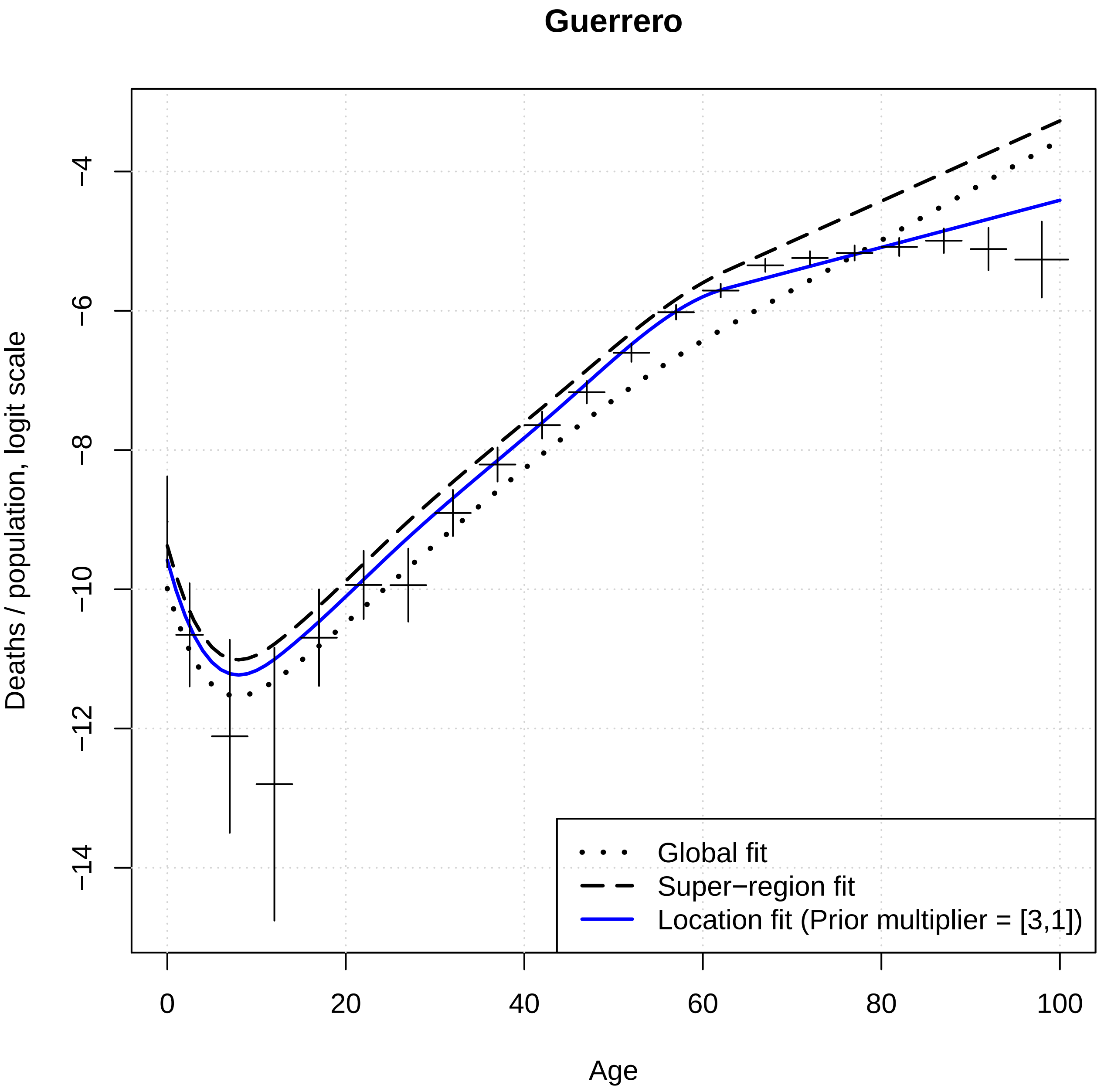

Cumulative reported deaths as of: 2021−01−06

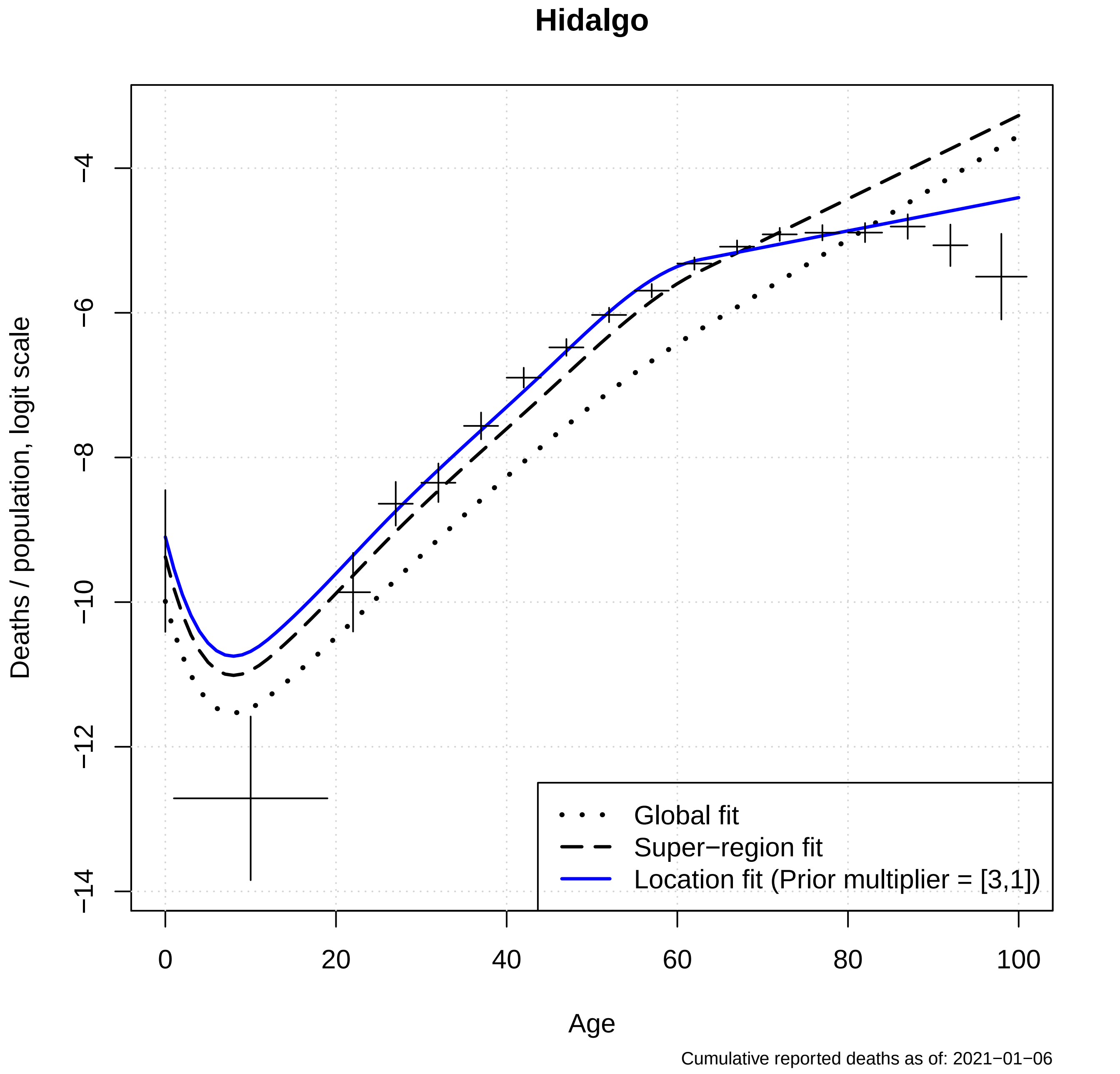

Hidalgo
Deaths / population, logit scale
−4
−6
−8
−10
−12
−14
0
20
40
60
80
100
Age
Global fit
Super−region fit
Location fit (Prior multiplier = [3,1])
Cumulative reported deaths as of: 2021−01−06

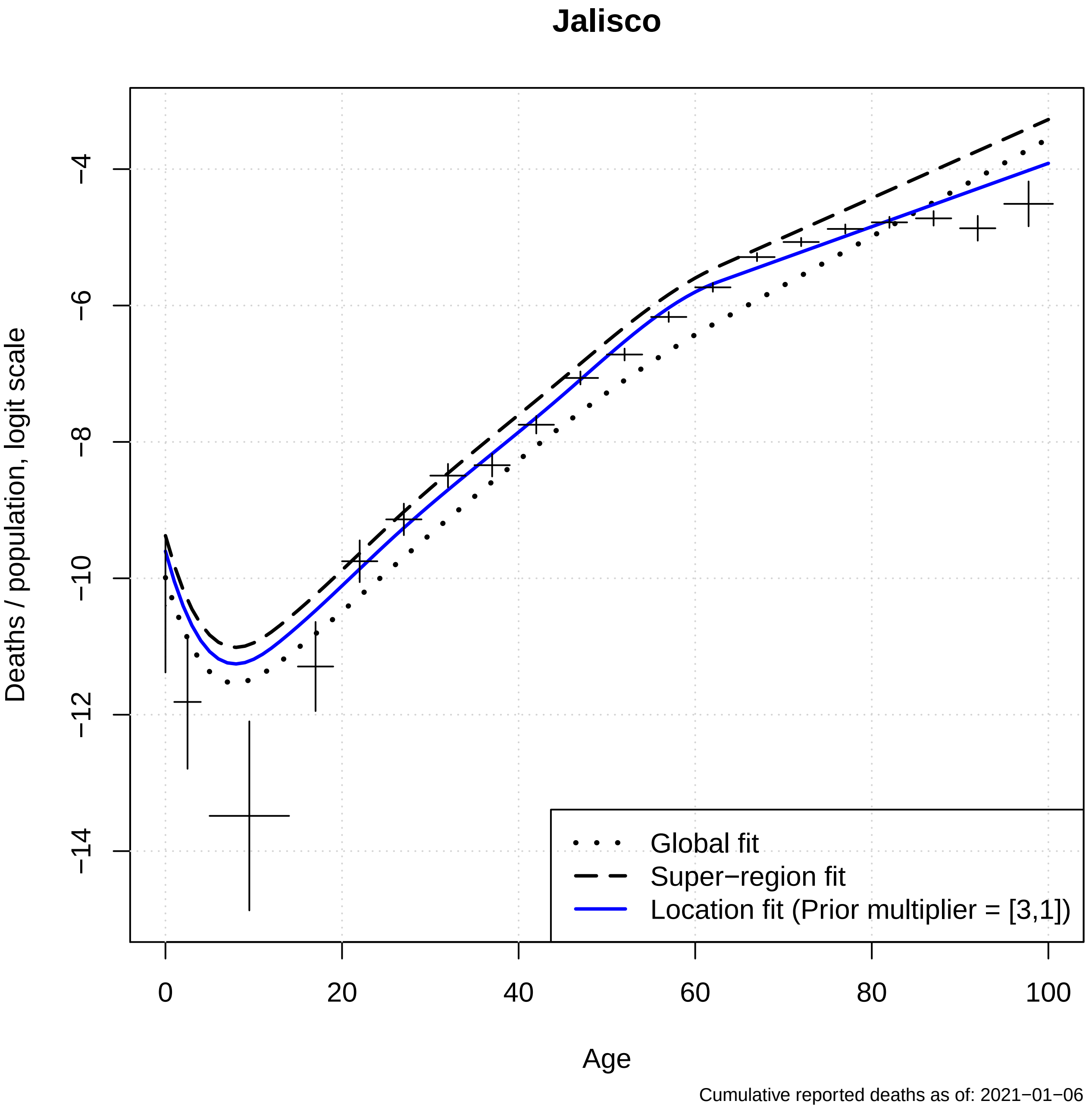

Jalisco
Deaths / population, logit scale
−4
−6
−8
−10
−12
−14
0
20
40
60
80
100
Age
Global fit
Super−region fit
Location fit (Prior multiplier = [3,1])
Cumulative reported deaths as of: 2021−01−06

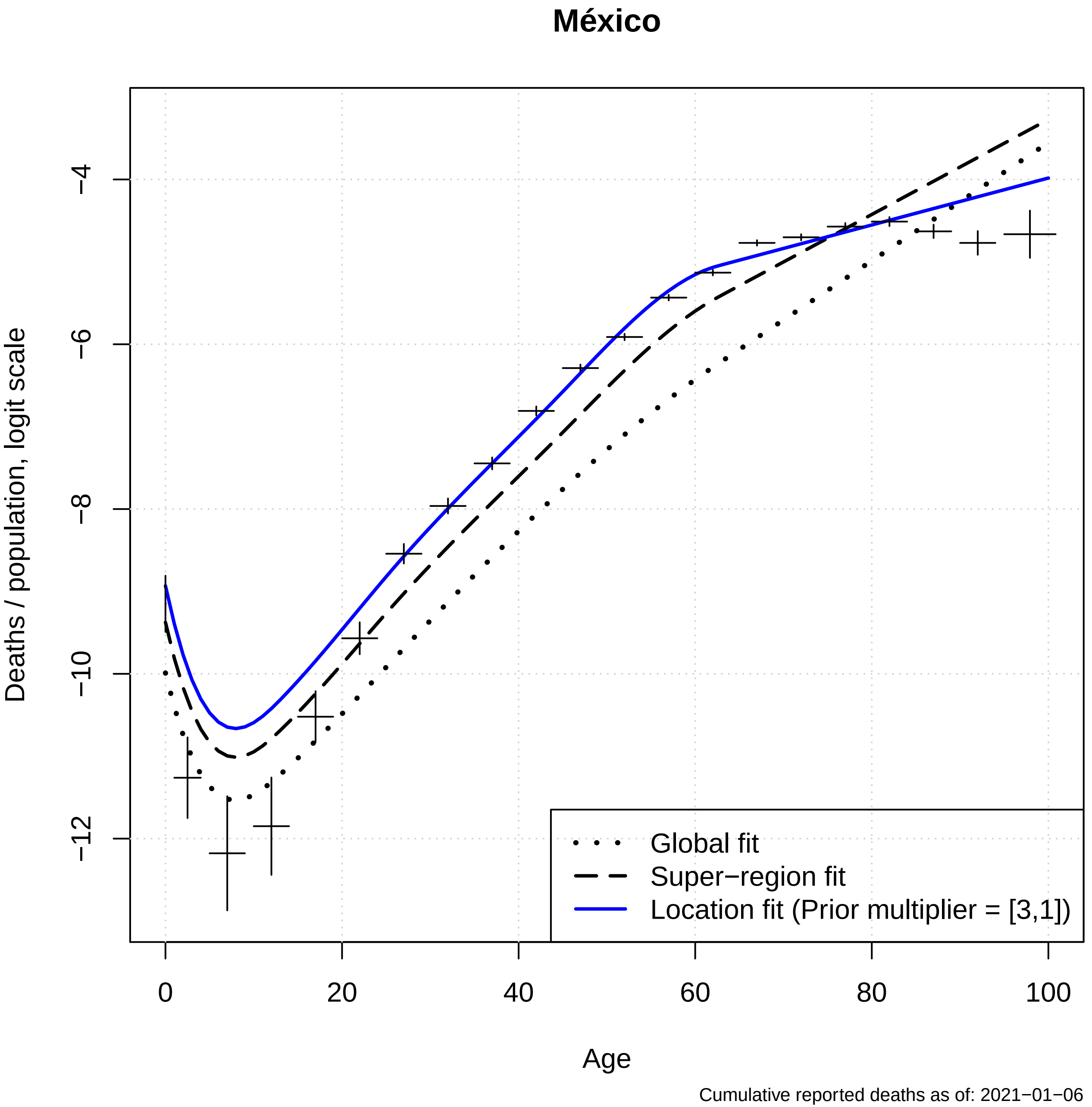
México
Deaths / population, logit scale
−4
−6
−8
−10
−12
0
20
40
60
80
100
Age
Global fit
Super−region fit
Location fit (Prior multiplier = [3,1])
Cumulative reported deaths as of: 2021−01−06

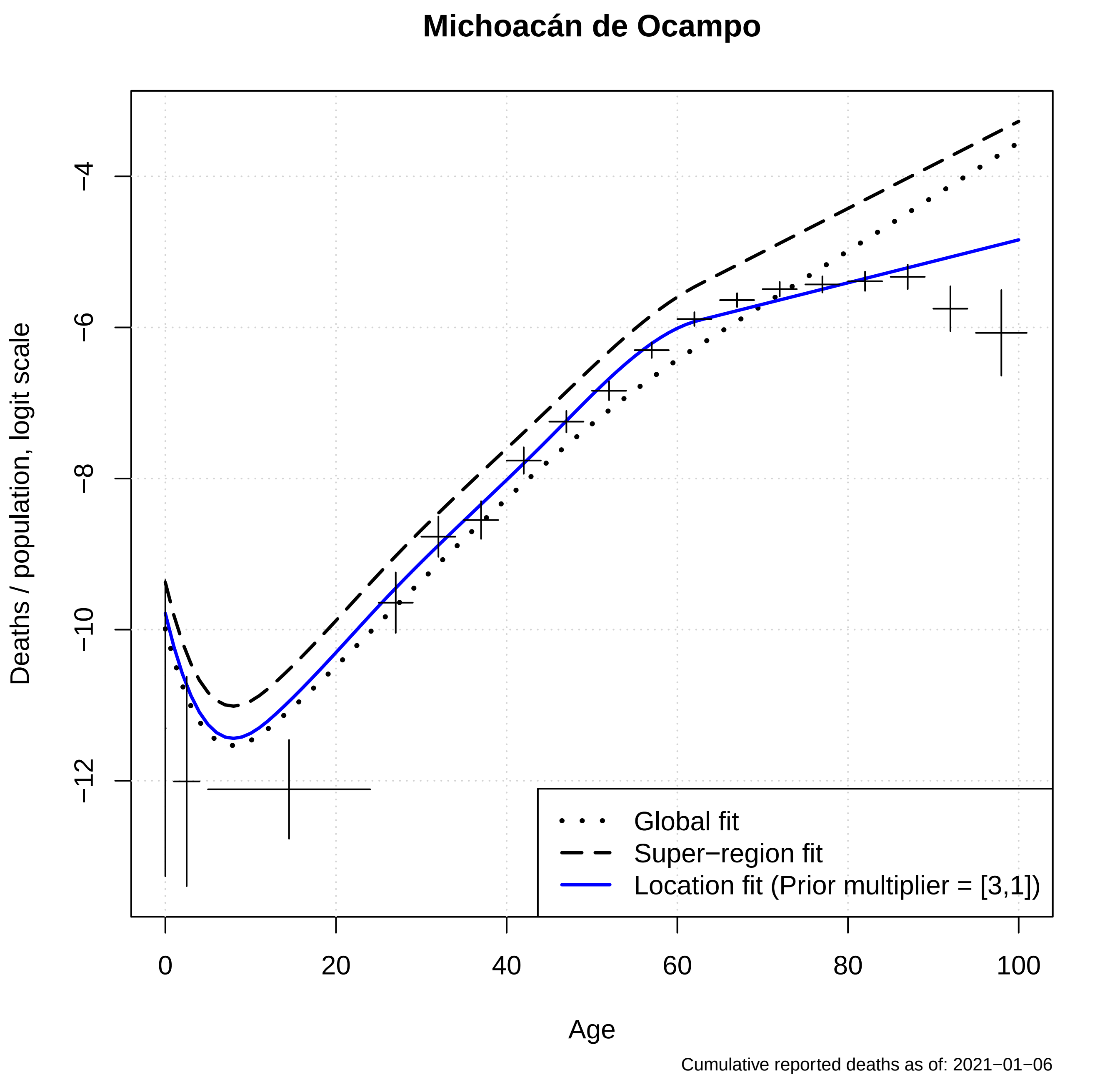
Michoacán de Ocampo
Deaths / population, logit scale
−4
−6
−8
−10
−12
0
20
40
60
80
100
Age
Global fit
Super−region fit
Location fit (Prior multiplier = [3,1])
Cumulative reported deaths as of: 2021−01−06

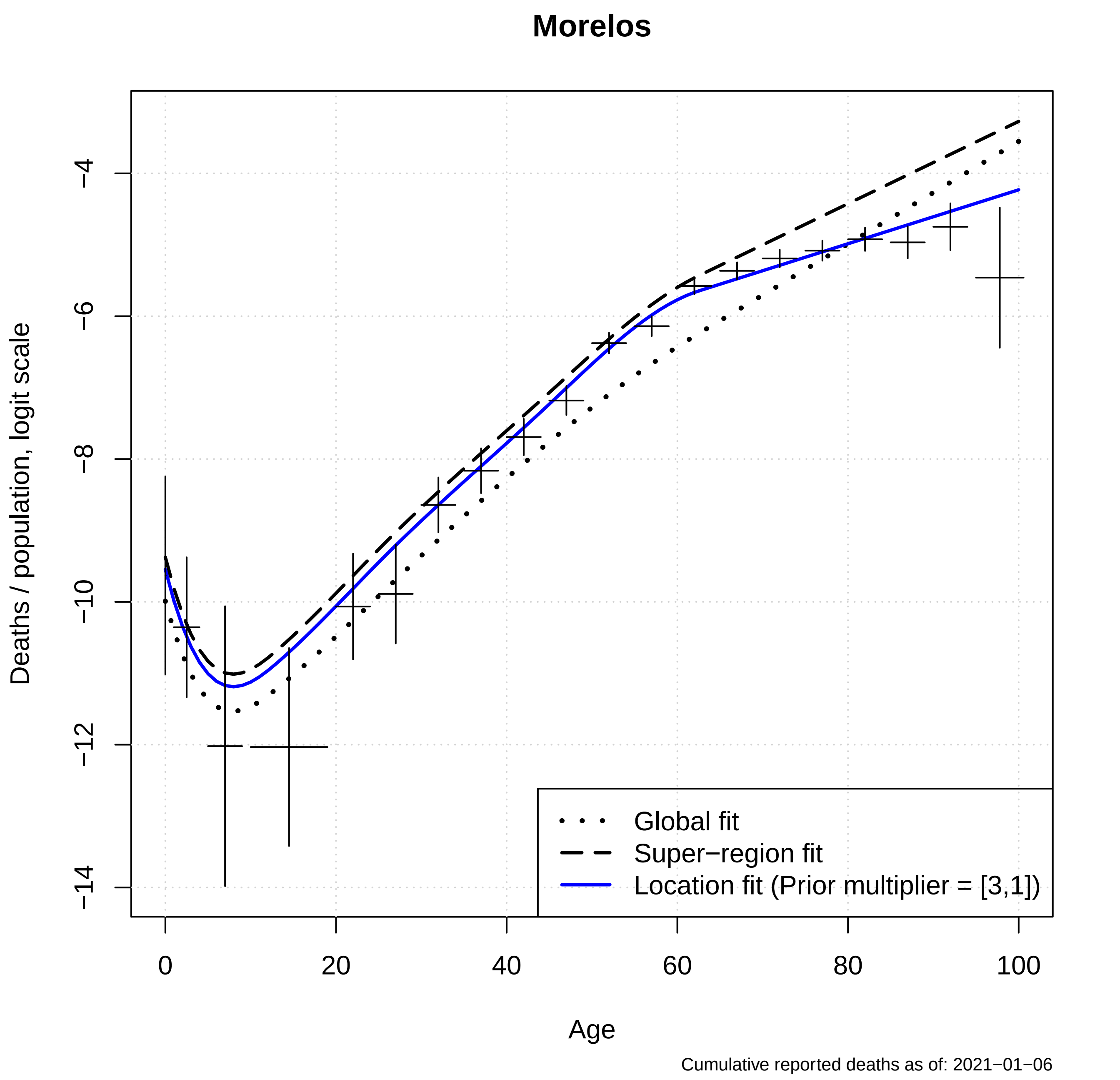

Morelos
Deaths / population, logit scale
−4
−6
−8
−10
−12
−14
0
20
40
60
80
100
Age
Global fit
Super−region fit
Location fit (Prior multiplier = [3,1])
Cumulative reported deaths as of: 2021−01−06

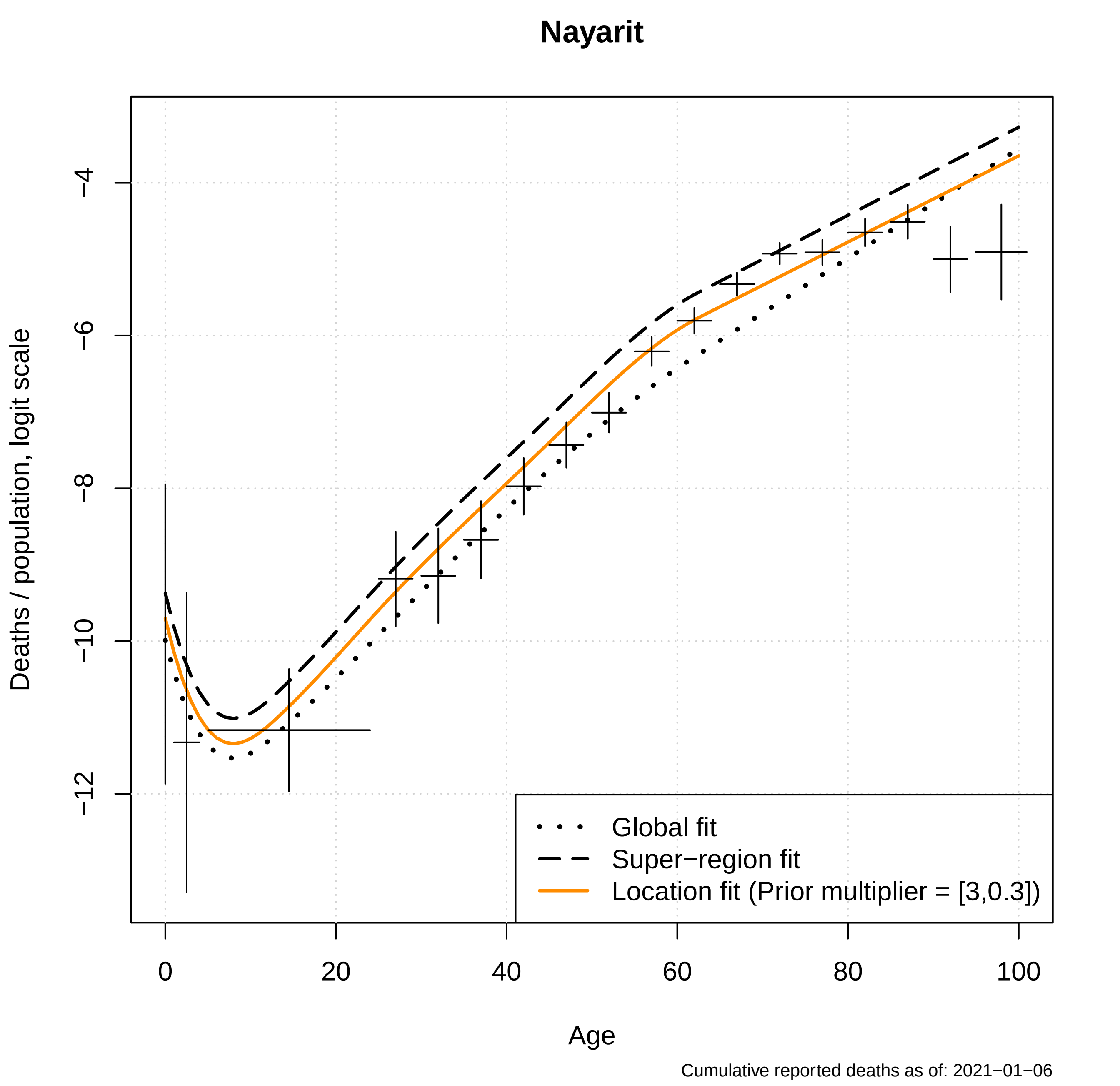
Nayarit
Deaths / population, logit scale
−4
−6
−8
−10
−12
0
20
40
60
80
100
Age
Global fit
Super−region fit
Location fit (Prior multiplier = [3,0.3])
Cumulative reported deaths as of: 2021−01−06

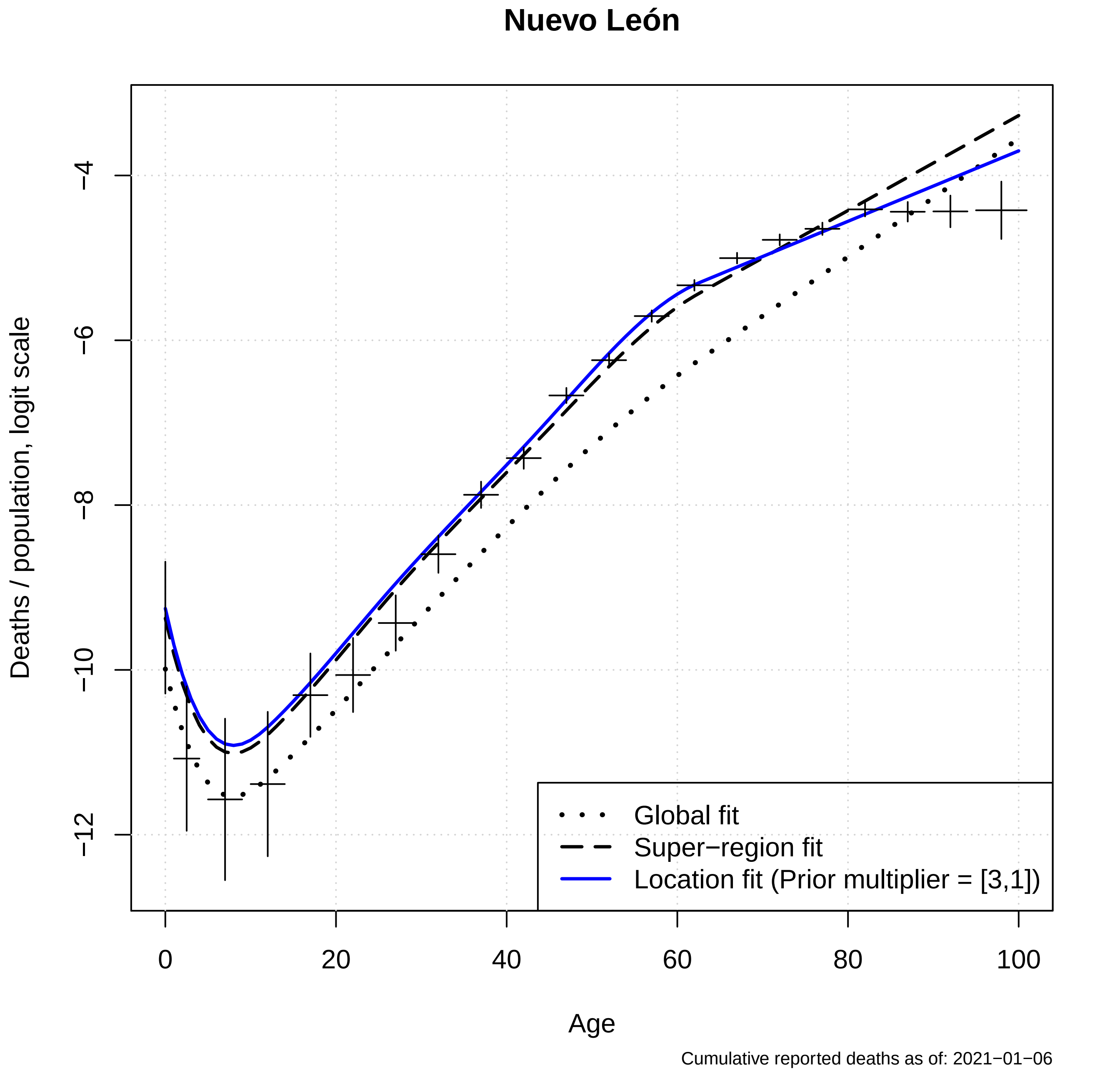

Nuevo León
Deaths / population, logit scale
−4
−6
−8
−10
−12
0
20
40
60
80
100
Age
Global fit
Super−region fit
Location fit (Prior multiplier = [3,1])
Cumulative reported deaths as of: 2021−01−06

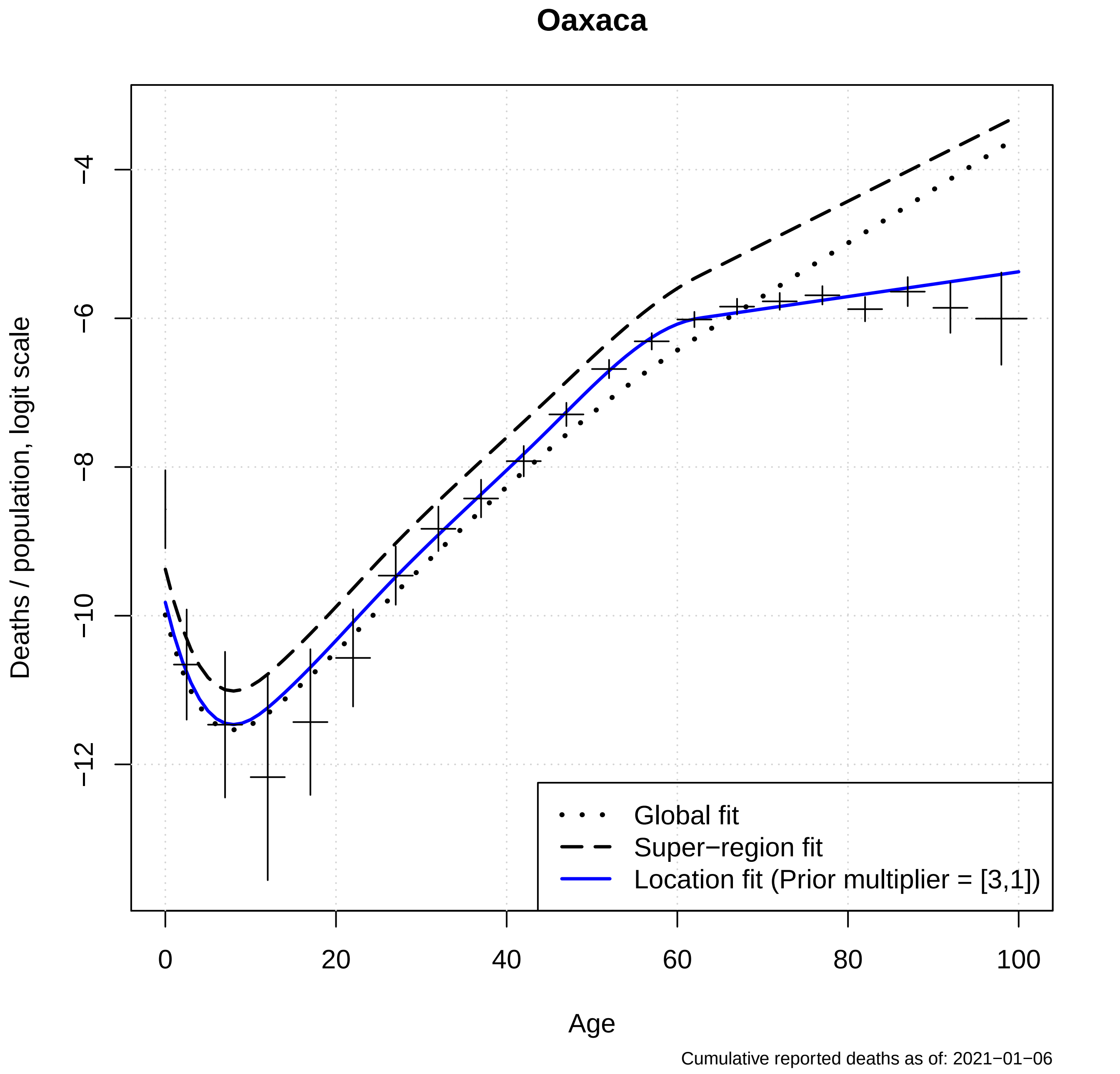

Oaxaca
Deaths / population, logit scale
−4
−6
−8
−10
−12
0
20
40
60
80
100
Age
Global fit
Super−region fit
Location fit (Prior multiplier = [3,1])
Cumulative reported deaths as of: 2021−01−06

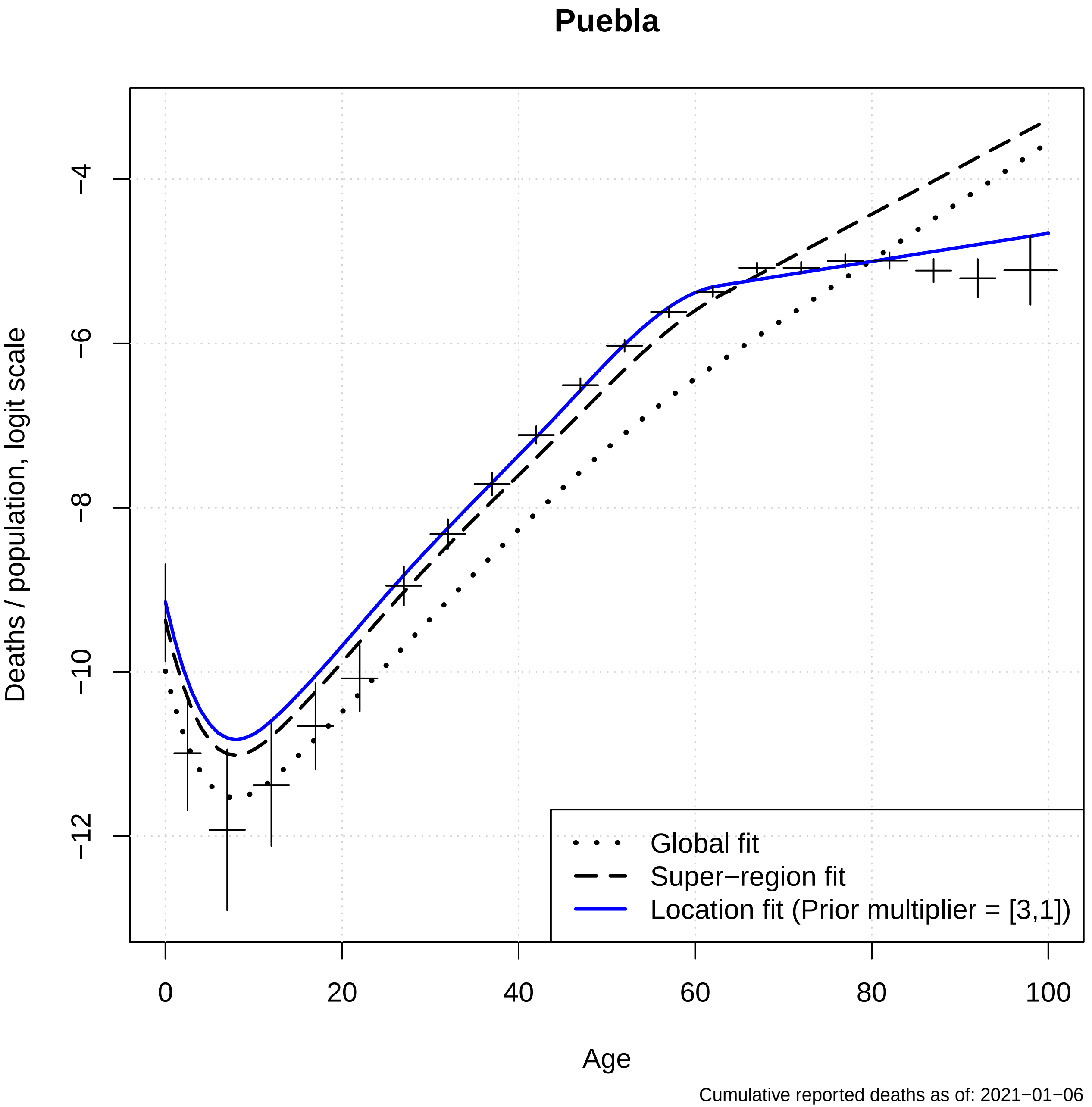
Puebla
Deaths / population, logit scale
−4
−6
−8
−10
−12
0
20
40
60
80
100
Age
Global fit
Super−region fit
Location fit (Prior multiplier = [3,1])
Cumulative reported deaths as of: 2021−01−06

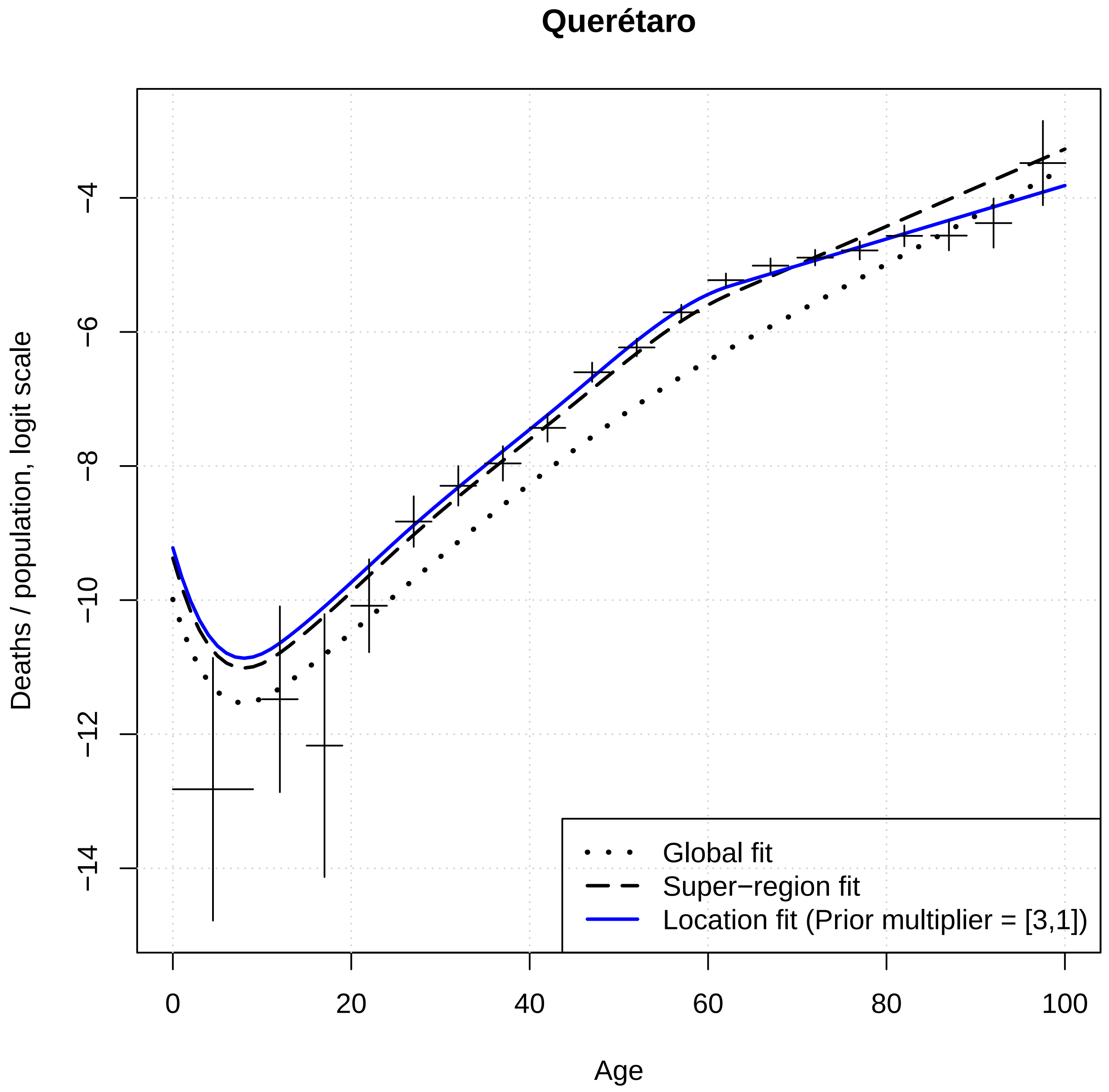

Cumulative reported deaths as of: 2021−01−06

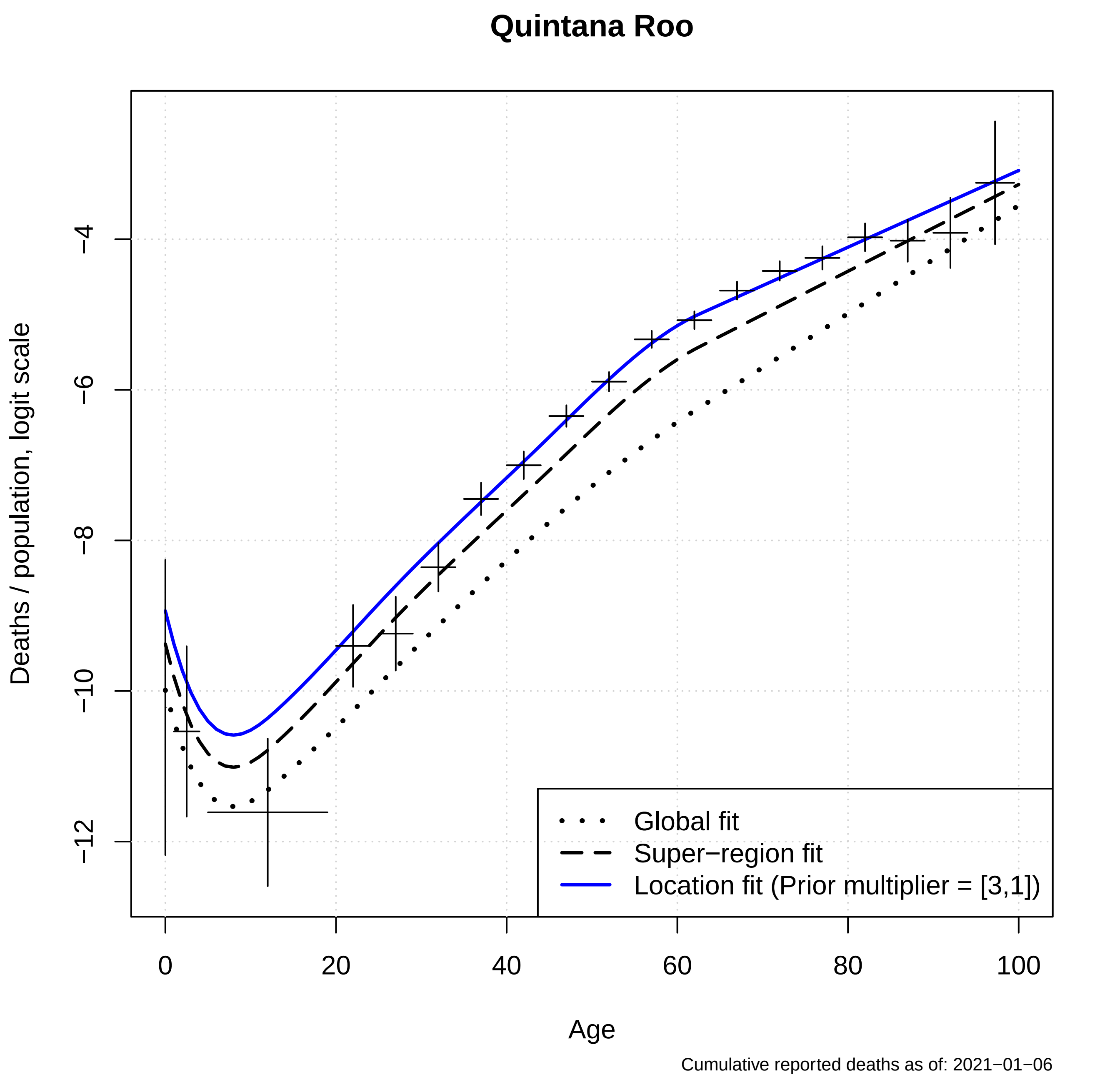

Quintana Roo
Deaths / population, logit scale
Age
−4
−6
−8
−10
−12
0
20
40
60
80
100
Global fit
Super−region fit
Location fit (Prior multiplier = [3,1])
Cumulative reported deaths as of: 2021−01−06

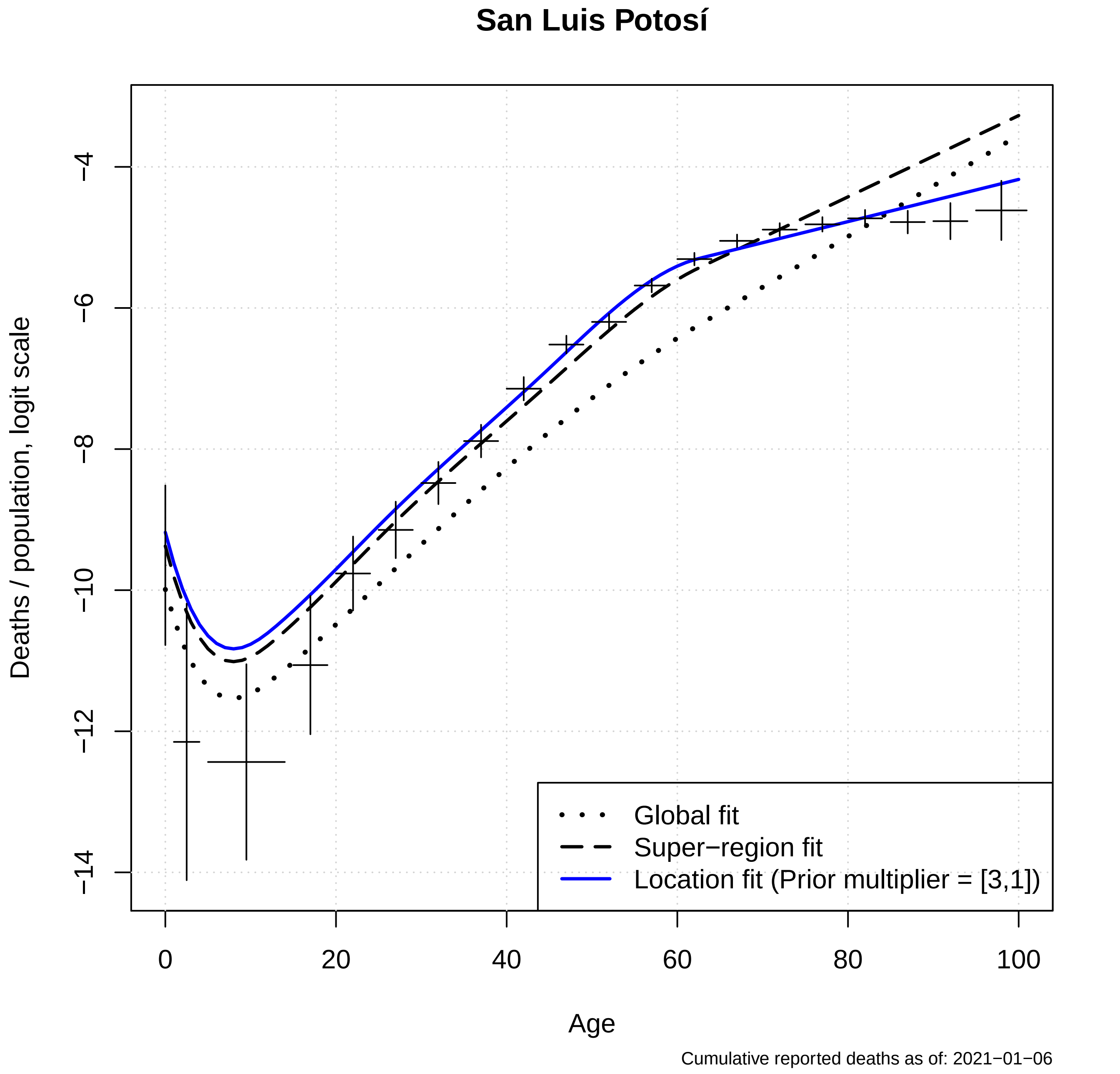
San Luis Potosí
Deaths / population, logit scale
−4
−6
−8
−10
−12
−14
0
20
40
60
80
100
Age
Global fit
Super−region fit
Location fit (Prior multiplier = [3,1])
Cumulative reported deaths as of: 2021−01−06

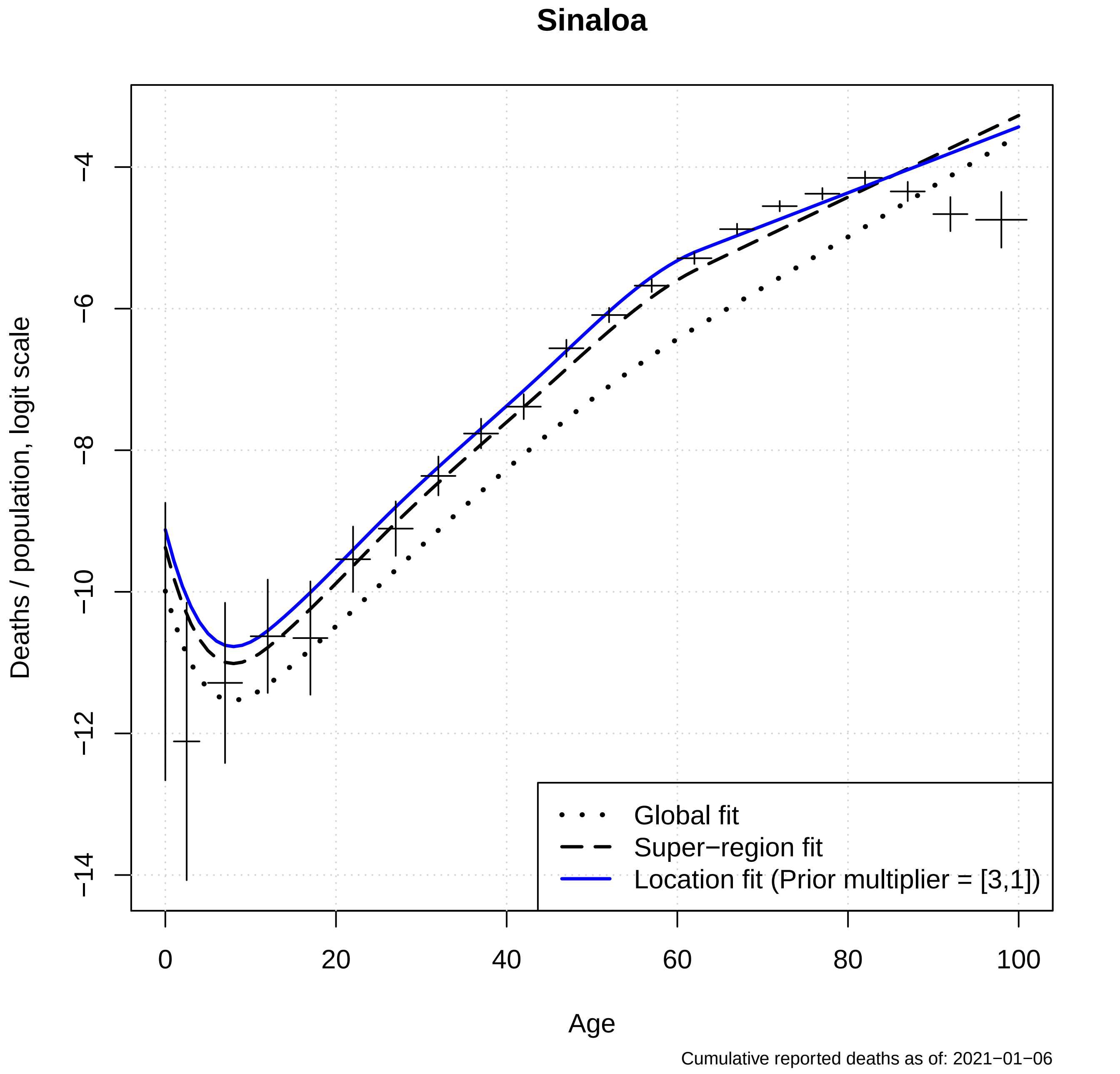
Sinaloa
Deaths / population, logit scale
−4
−6
−8
−10
−12
−14
0
20
40
60
80
100
Age
Global fit
Super−region fit
Location fit (Prior multiplier = [3,1])
Cumulative reported deaths as of: 2021−01−06

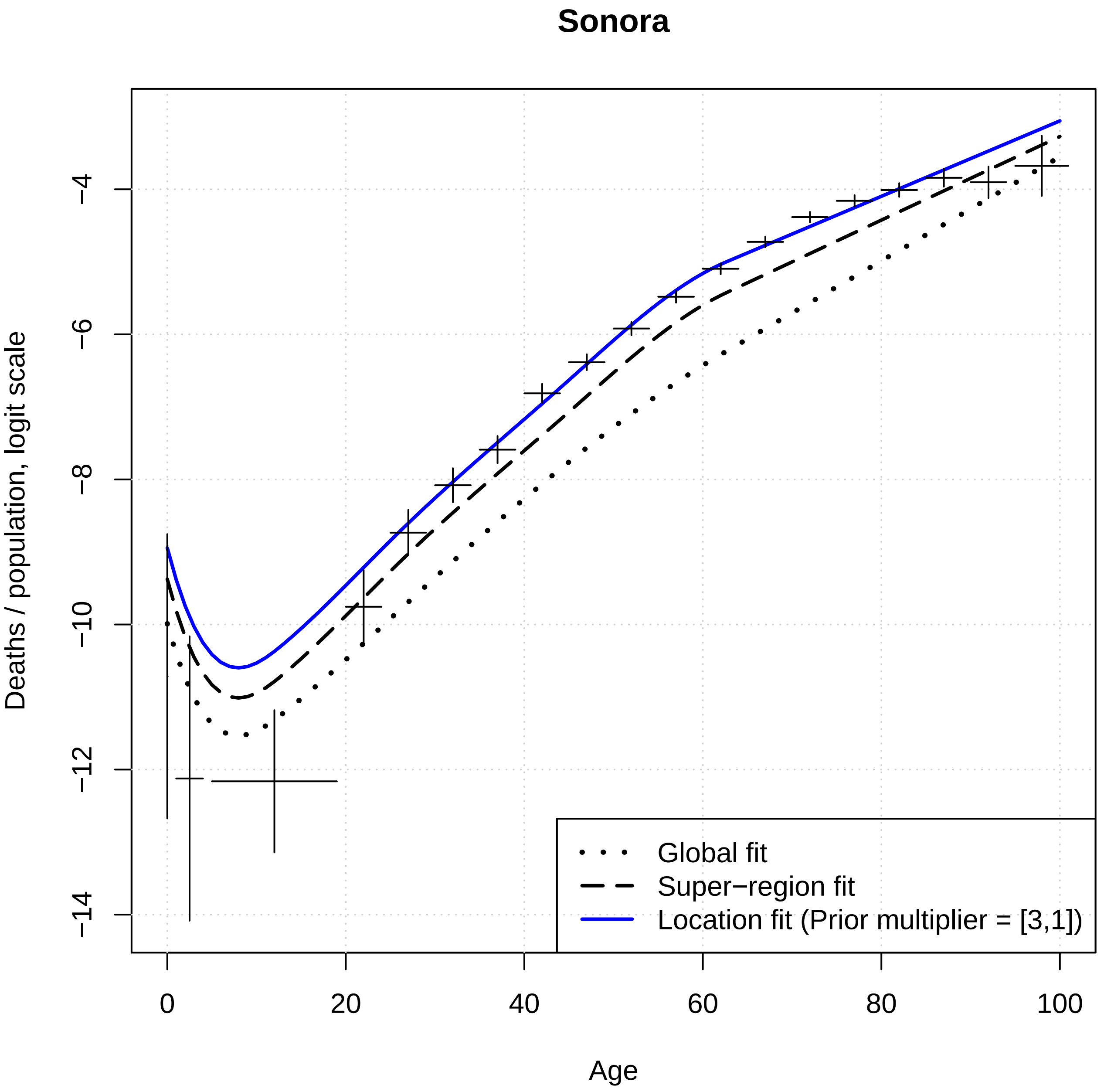

Cumulative reported deaths as of: 2021−01−06

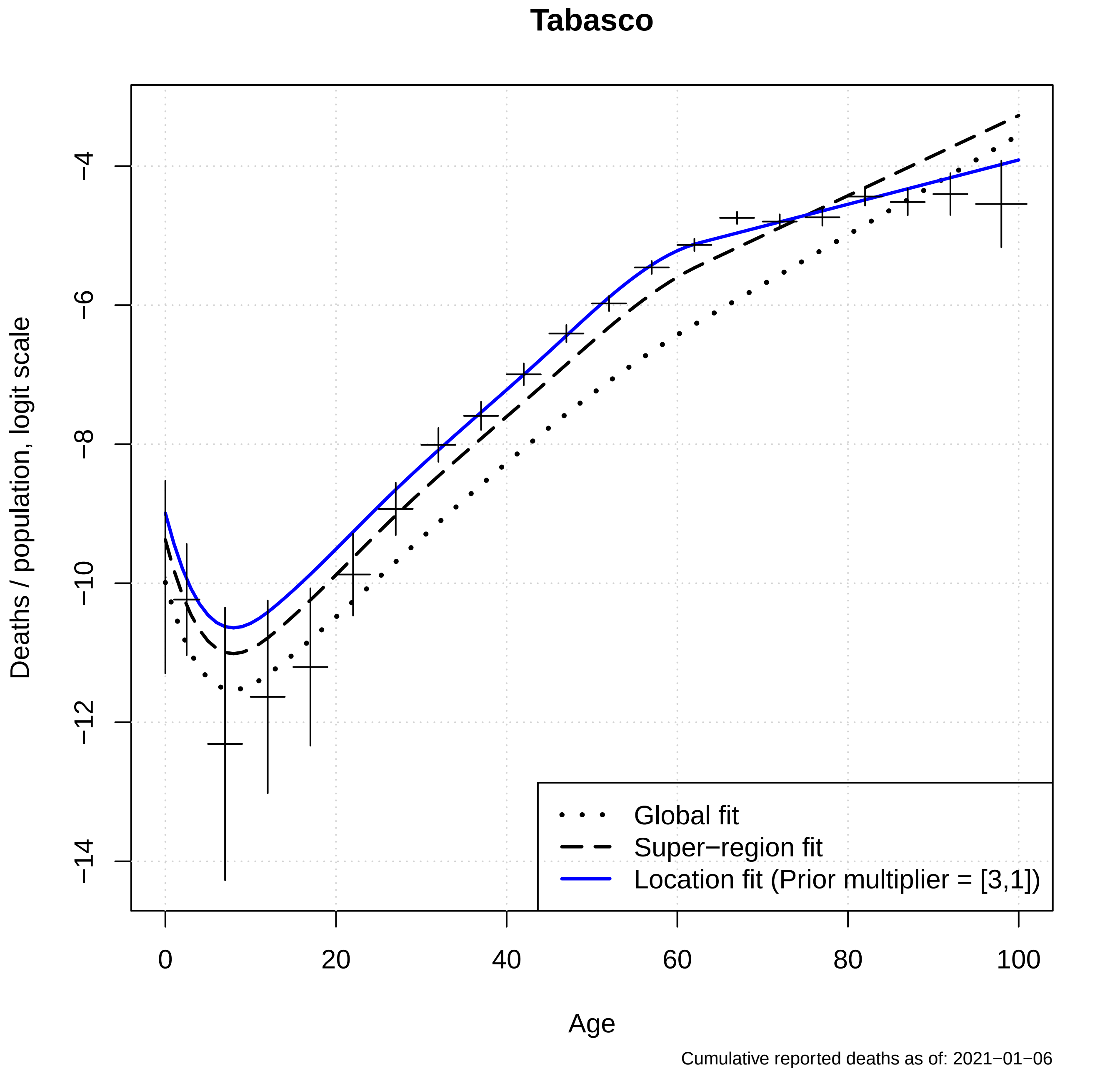

Tabasco
Deaths / population, logit scale
−4
−6
−8
−10
−12
−14
0
20
40
60
80
100
Age
Global fit
Super−region fit
Location fit (Prior multiplier = [3,1])
Cumulative reported deaths as of: 2021−01−06

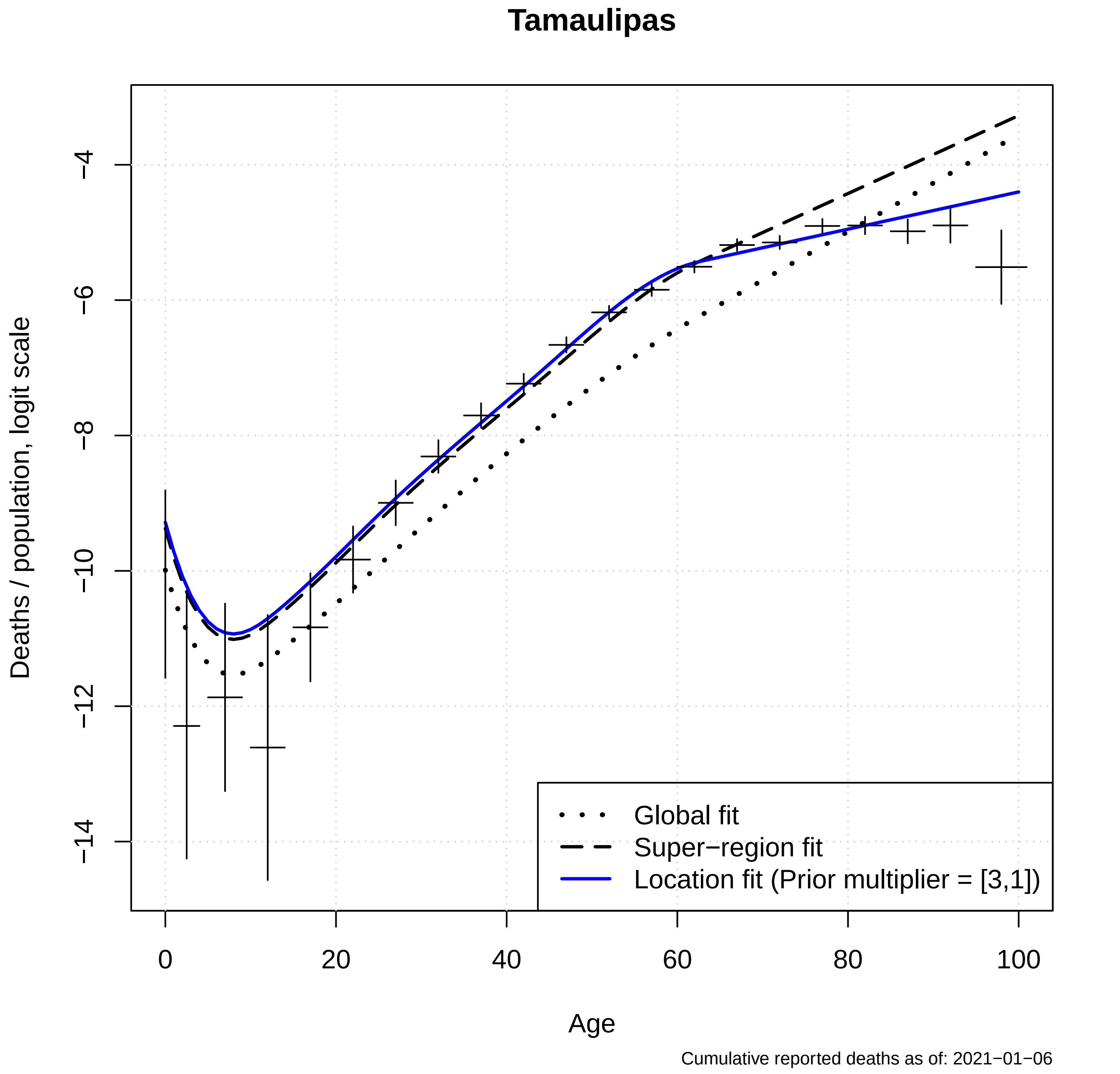
Tamaulipas
Deaths / population, logit scale
−4
−6
−8
−10
−12
−14
0
20
40
60
80
100
Age
Global fit
Super−region fit
Location fit (Prior multiplier = [3,1])
Cumulative reported deaths as of: 2021−01−06

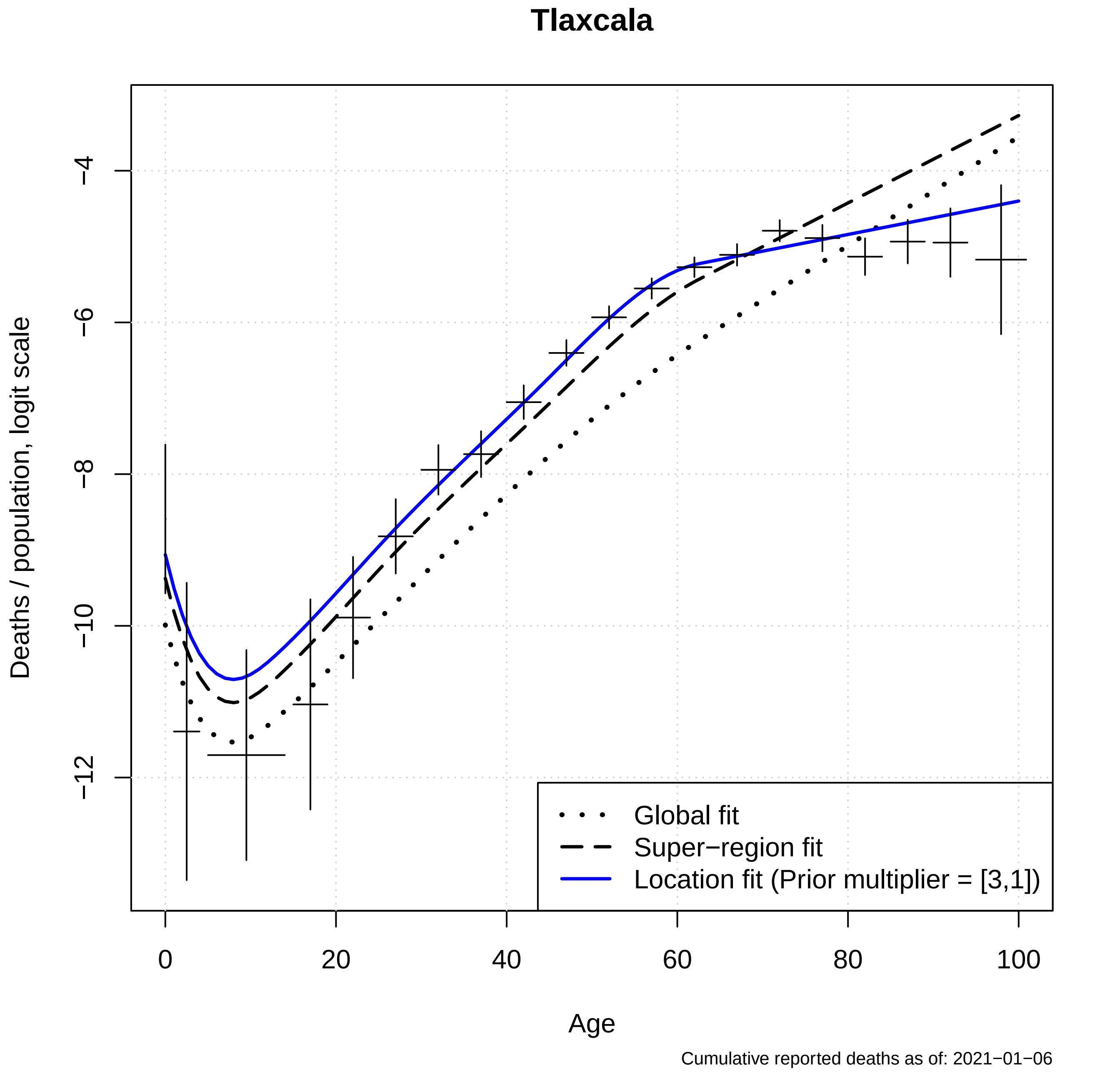

Tlaxcala
Deaths / population, logit scale
−4
−6
−8
−10
−12
0
20
40
60
80
100
Age
Global fit
Super−region fit
Location fit (Prior multiplier = [3,1])
Cumulative reported deaths as of: 2021−01−06

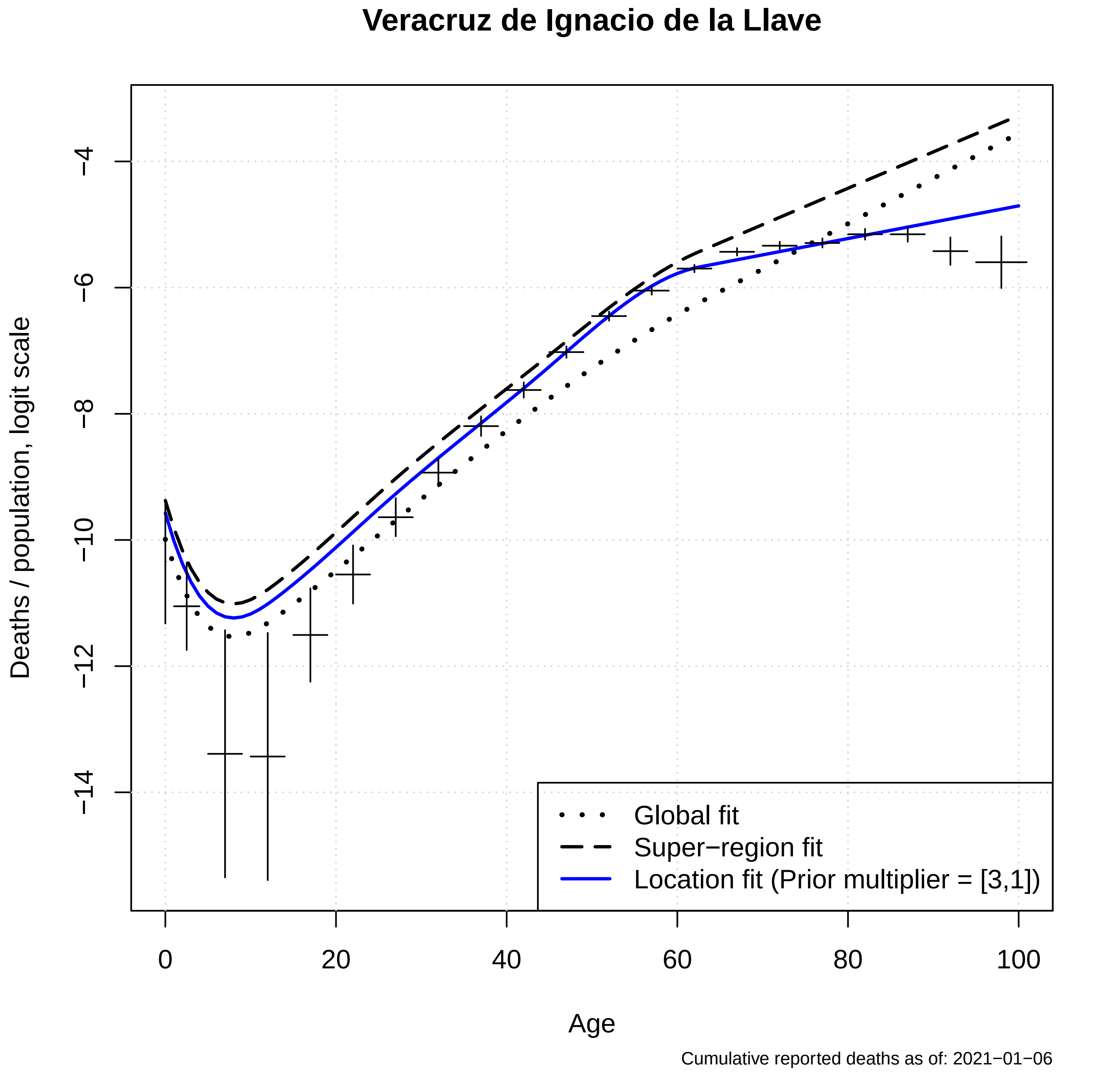

Veracruz de Ignacio de la Llave
Deaths / population, logit scale
−4
−6
−8
−10
−12
−14
0
20
40
60
80
100
Age
Global fit
Super−region fit
Location fit (Prior multiplier = [3,1])
Cumulative reported deaths as of: 2021−01−06

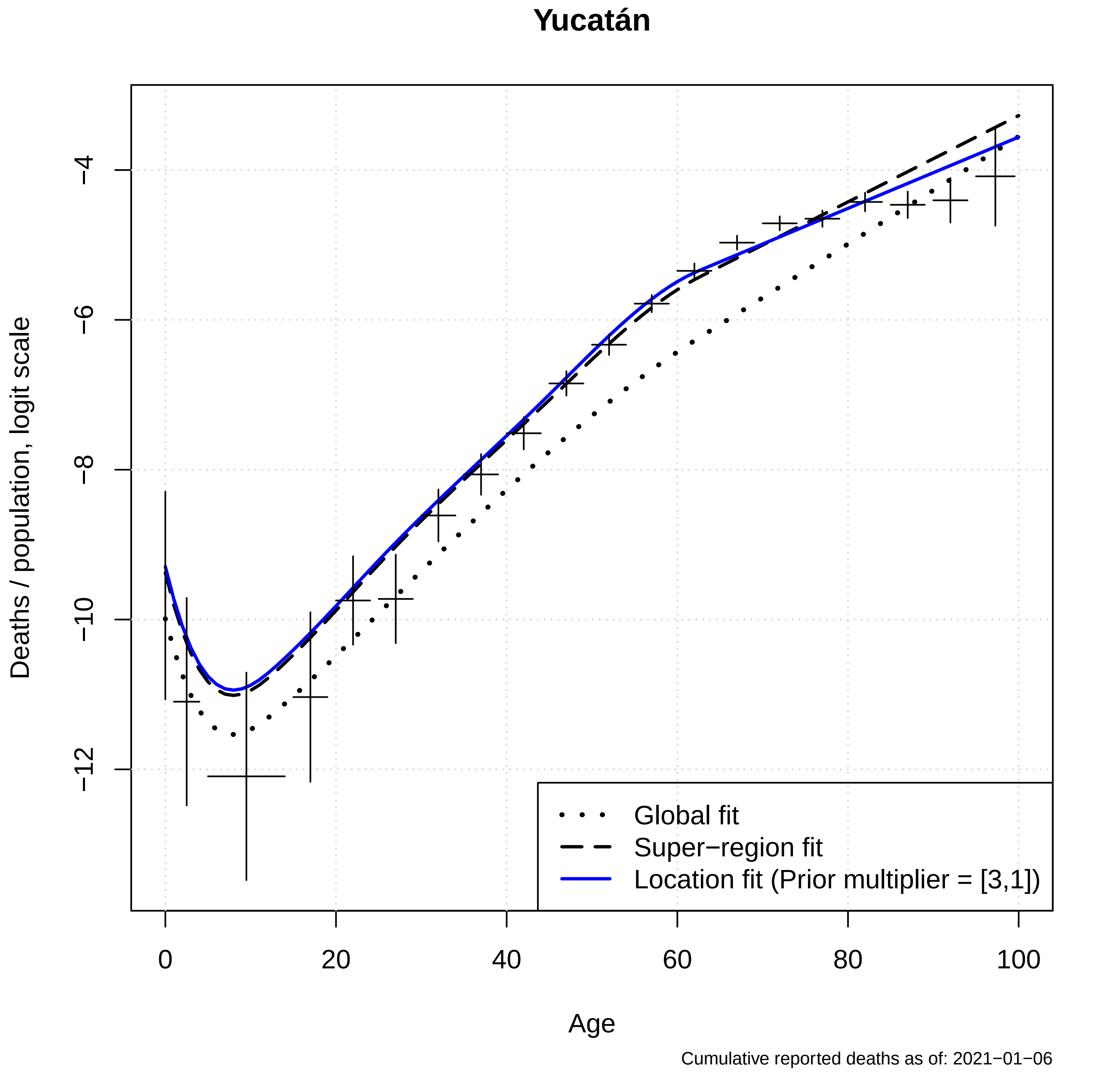

Yucatán
Deaths / population, logit scale
−4
−6
−8
−10
−12
0
20
40
60
80
100
Age
Global fit
Super−region fit
Location fit (Prior multiplier = [3,1])
Cumulative reported deaths as of: 2021−01−06

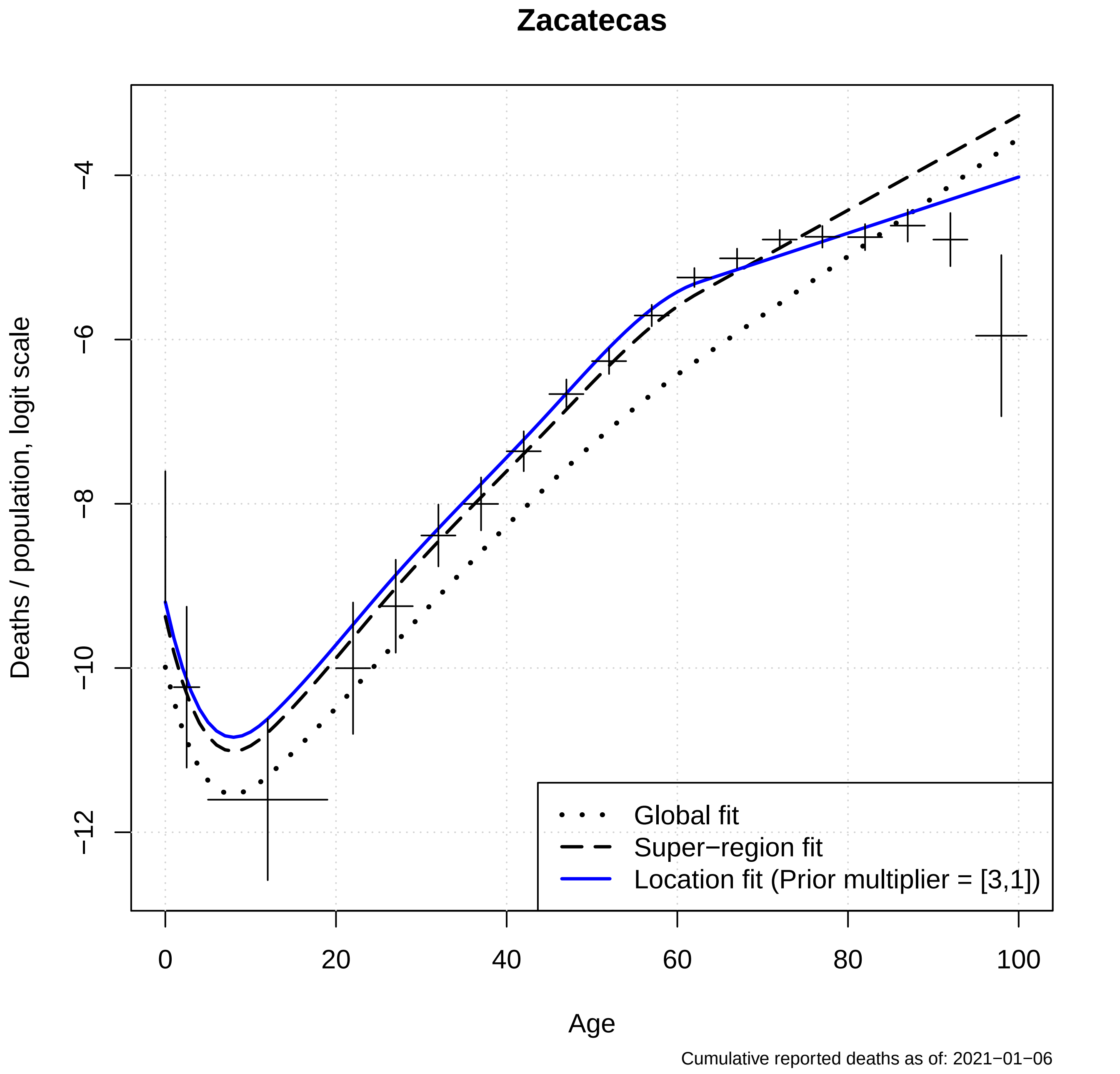

Zacatecas
Deaths / population, logit scale
−4
−6
−8
−10
−12
0
20
40
60
80
100
Age
Global fit
Super−region fit
Location fit (Prior multiplier = [3,1])
Cumulative reported deaths as of: 2021−01−06

Figure S8E. Indonesia subnationals

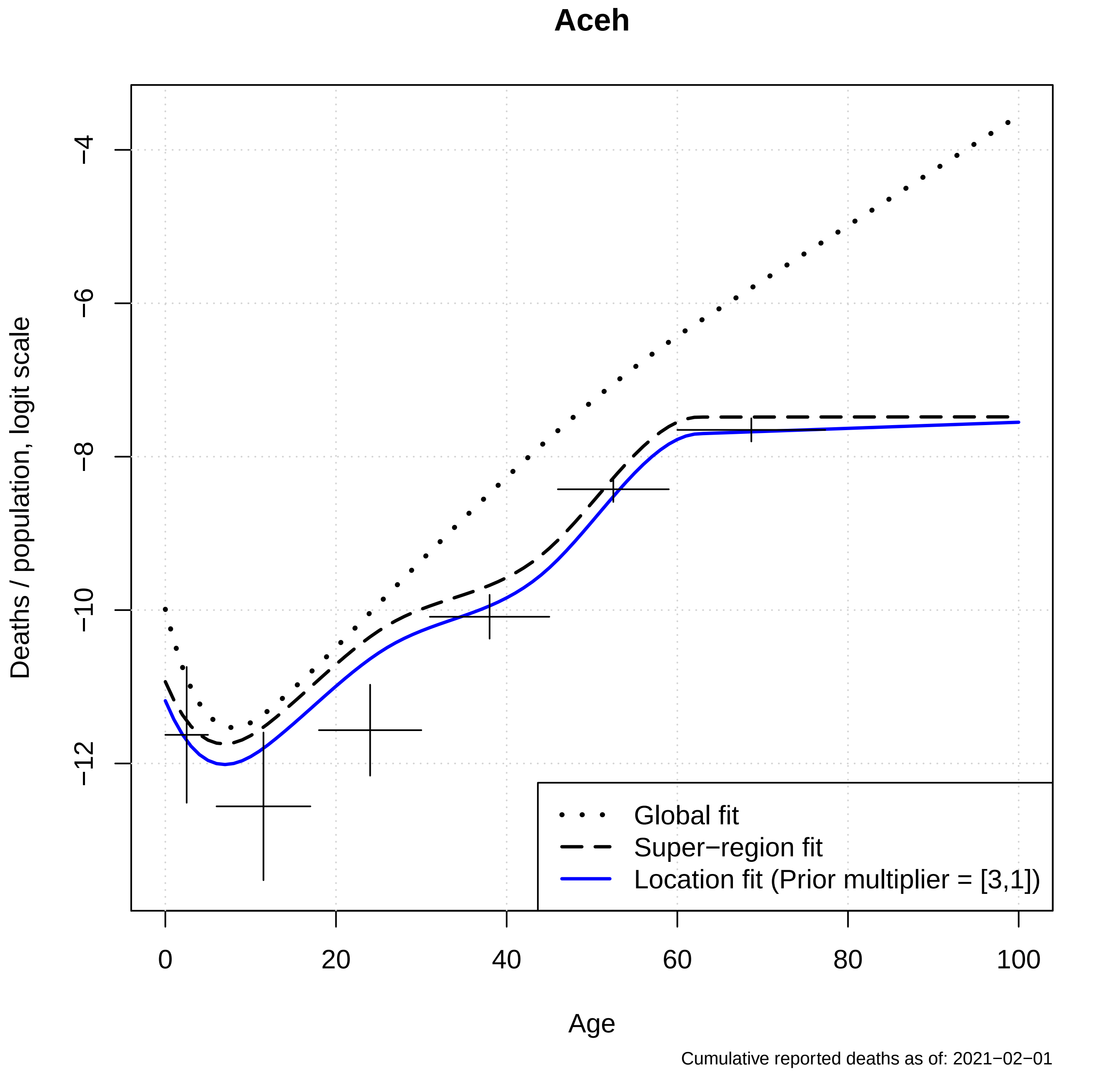

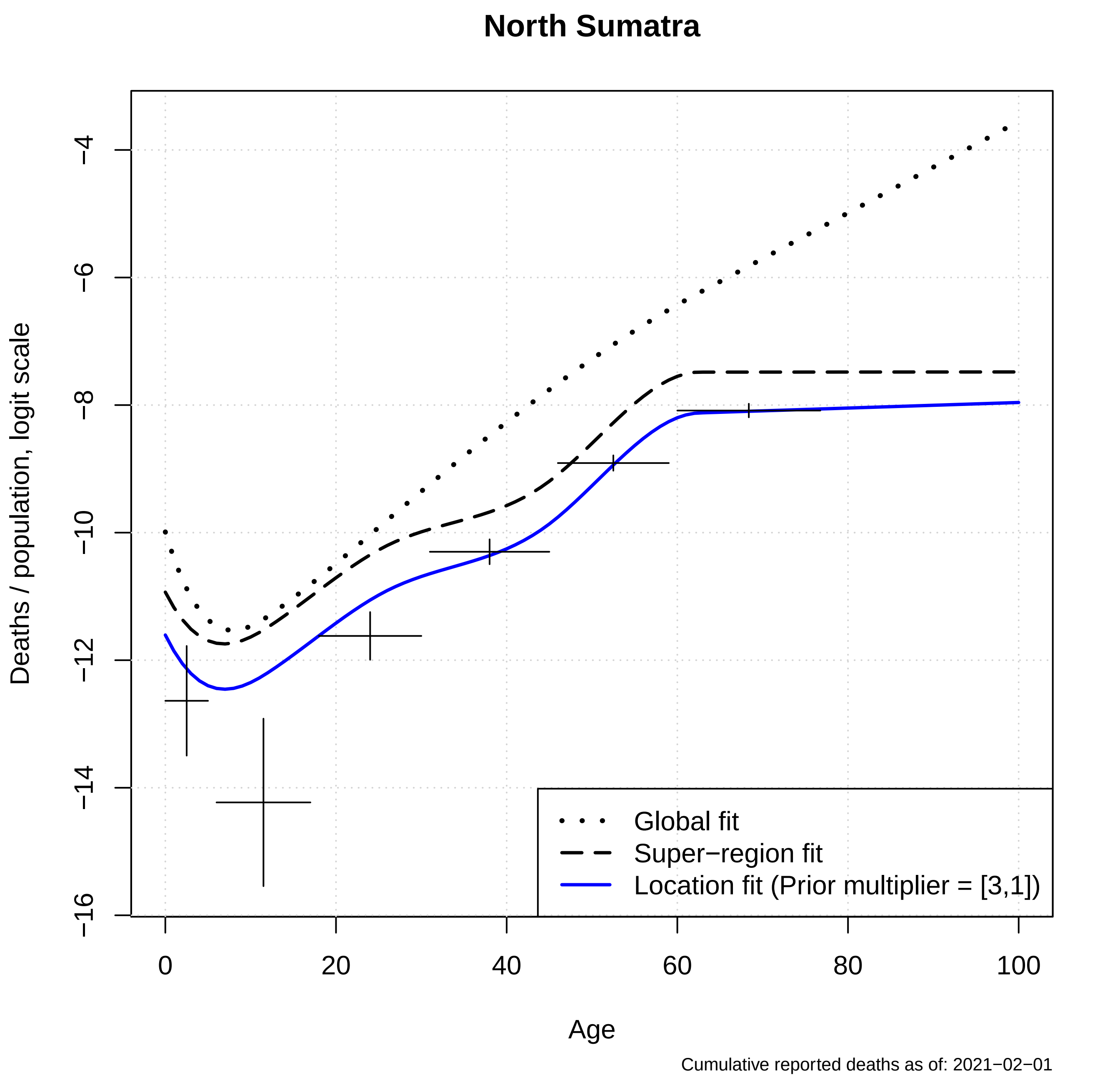

North Sumatra
Deaths / population, logit scale
−4
−6
−8
−10
−12
−14
−16
0
20
40
60
80
100
Age
Global fit
Super−region fit
Location fit (Prior multiplier = [3,1])
Cumulative reported deaths as of: 2021−02−01

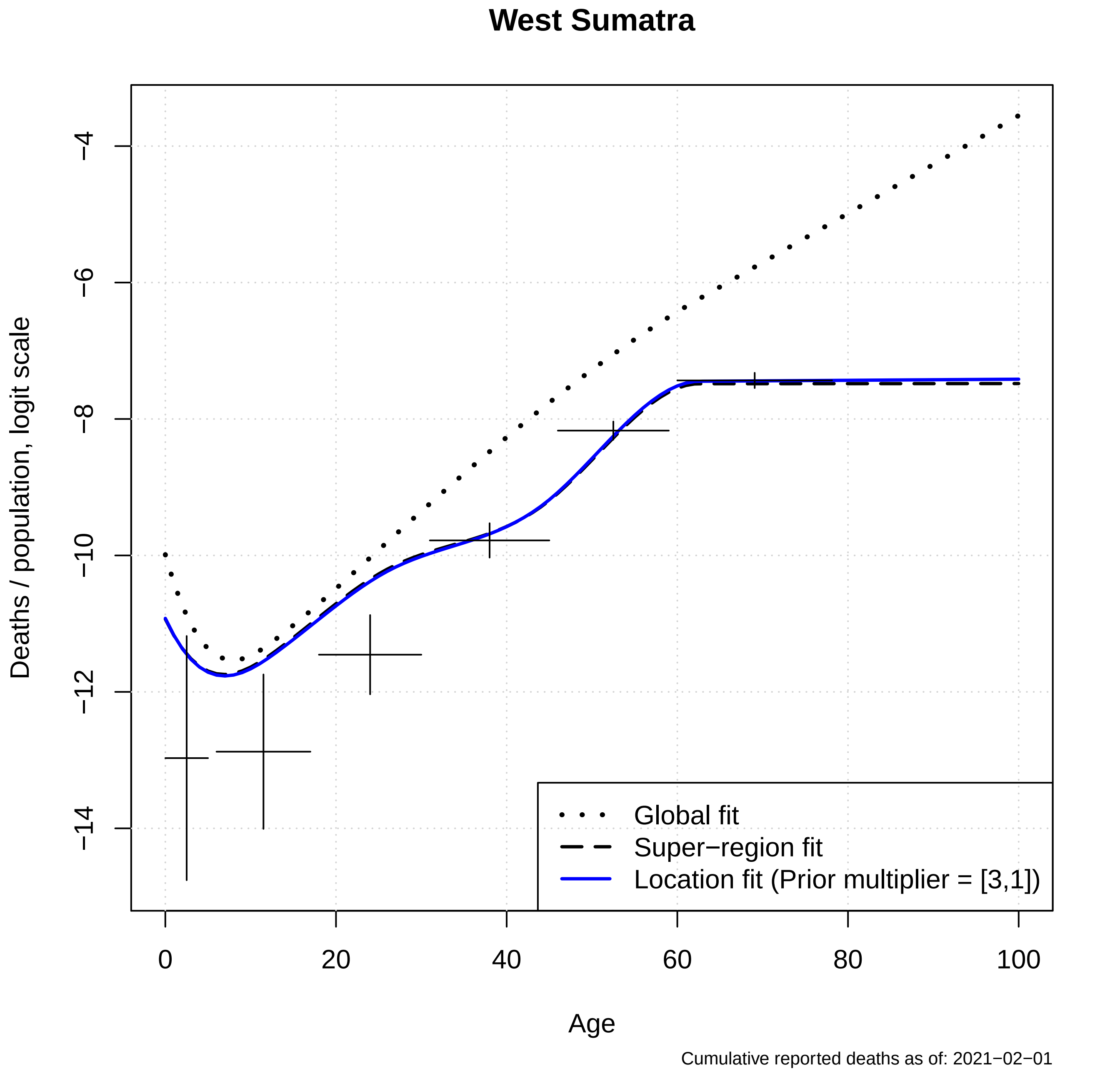
West Sumatra
Deaths / population, logit scale
−4
−6
−8
−10
−12
−14
0
20
40
60
80
100
Age
Global fit
Super−region fit
Location fit (Prior multiplier = [3,1])
Cumulative reported deaths as of: 2021−02−01

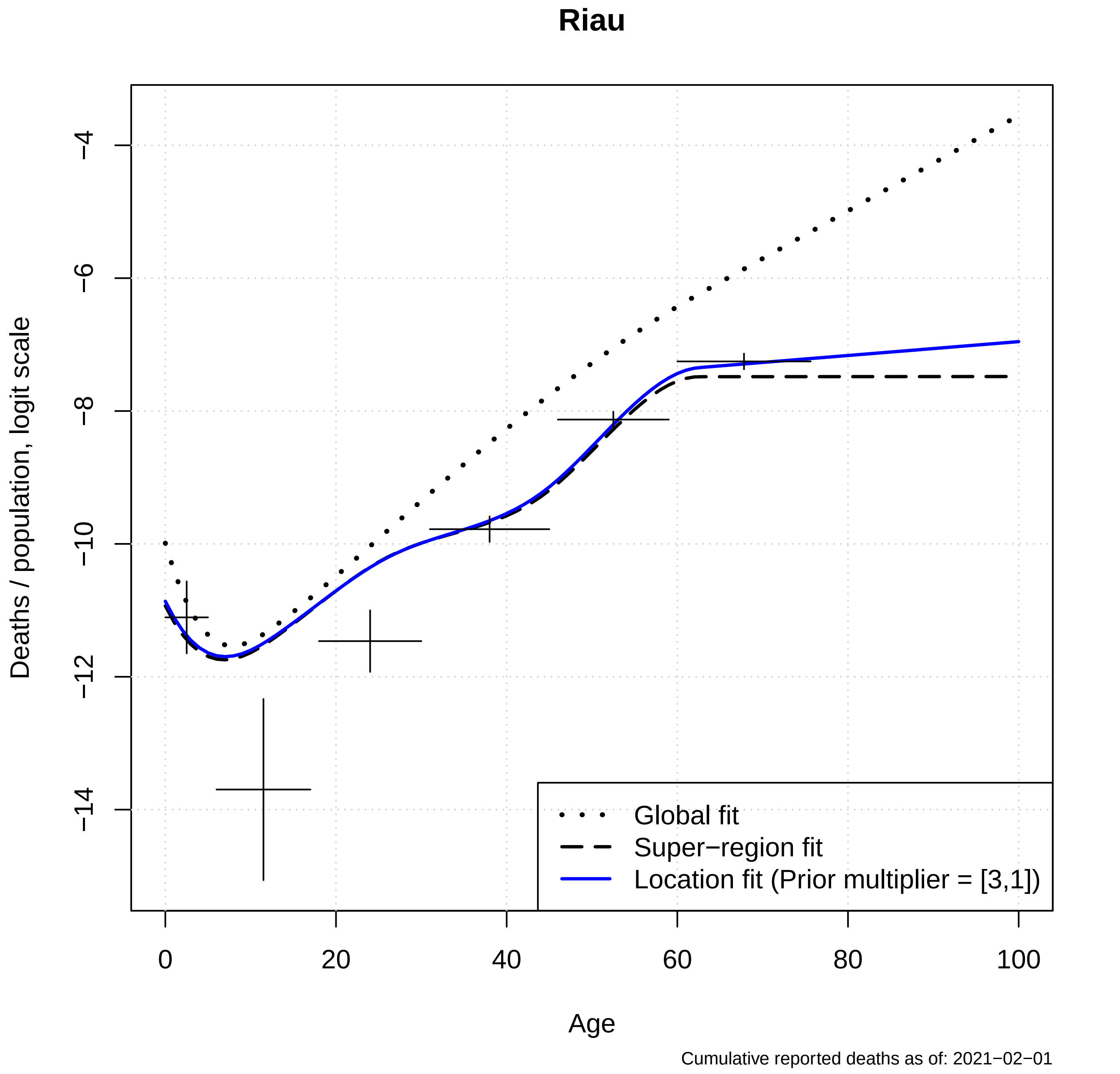
Riau
Deaths / population, logit scale
−4
−6
−8
−10
−12
−14
0
20
40
60
80
100
Age
Global fit
Super−region fit
Location fit (Prior multiplier = [3,1])
Cumulative reported deaths as of: 2021−02−01

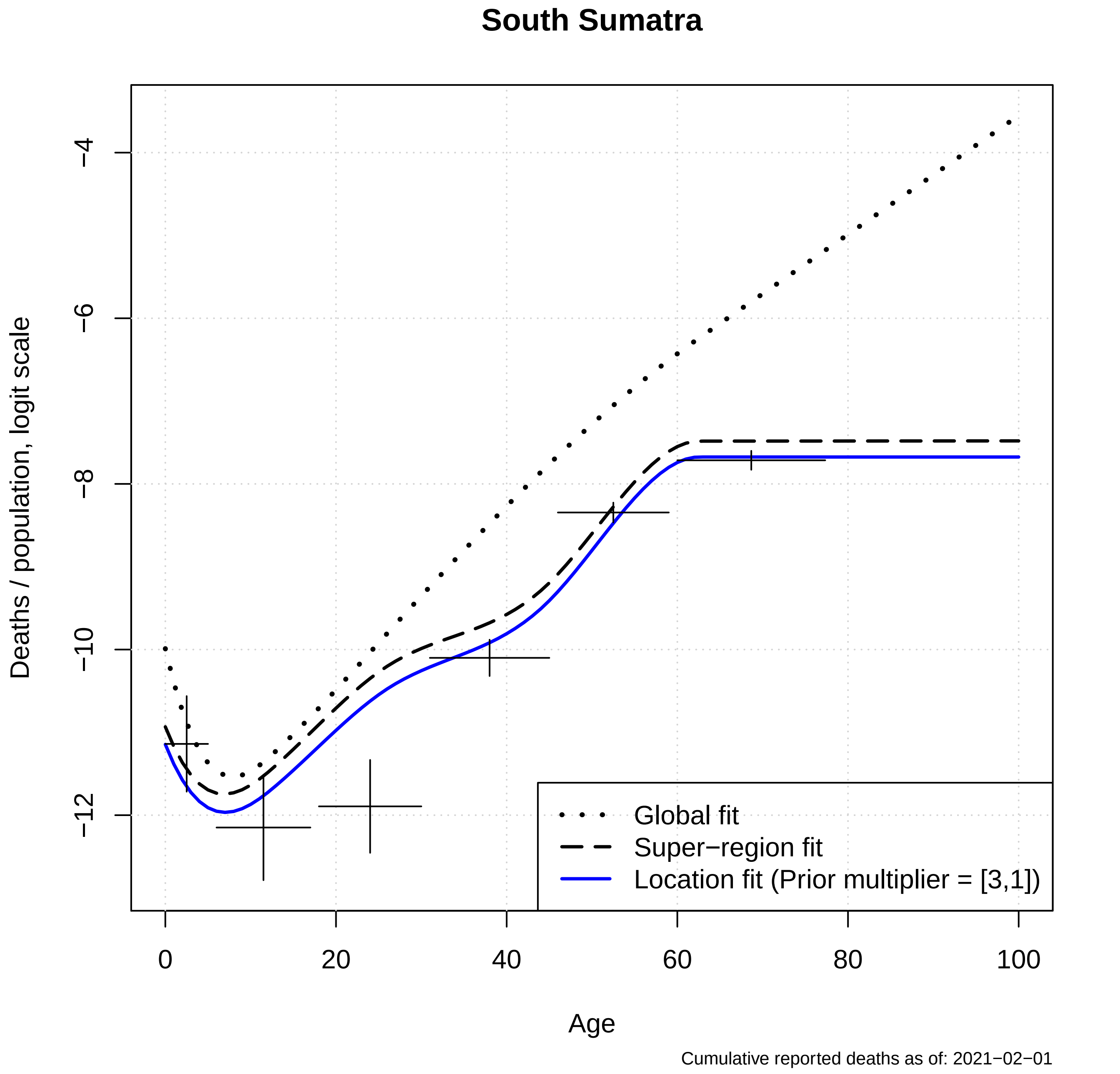

South Sumatra
Deaths / population, logit scale
−4
−6
−8
−10
−12
0
20
40
60
80
100
Age
Global fit
Super−region fit
Location fit (Prior multiplier = [3,1])
Cumulative reported deaths as of: 2021−02−01

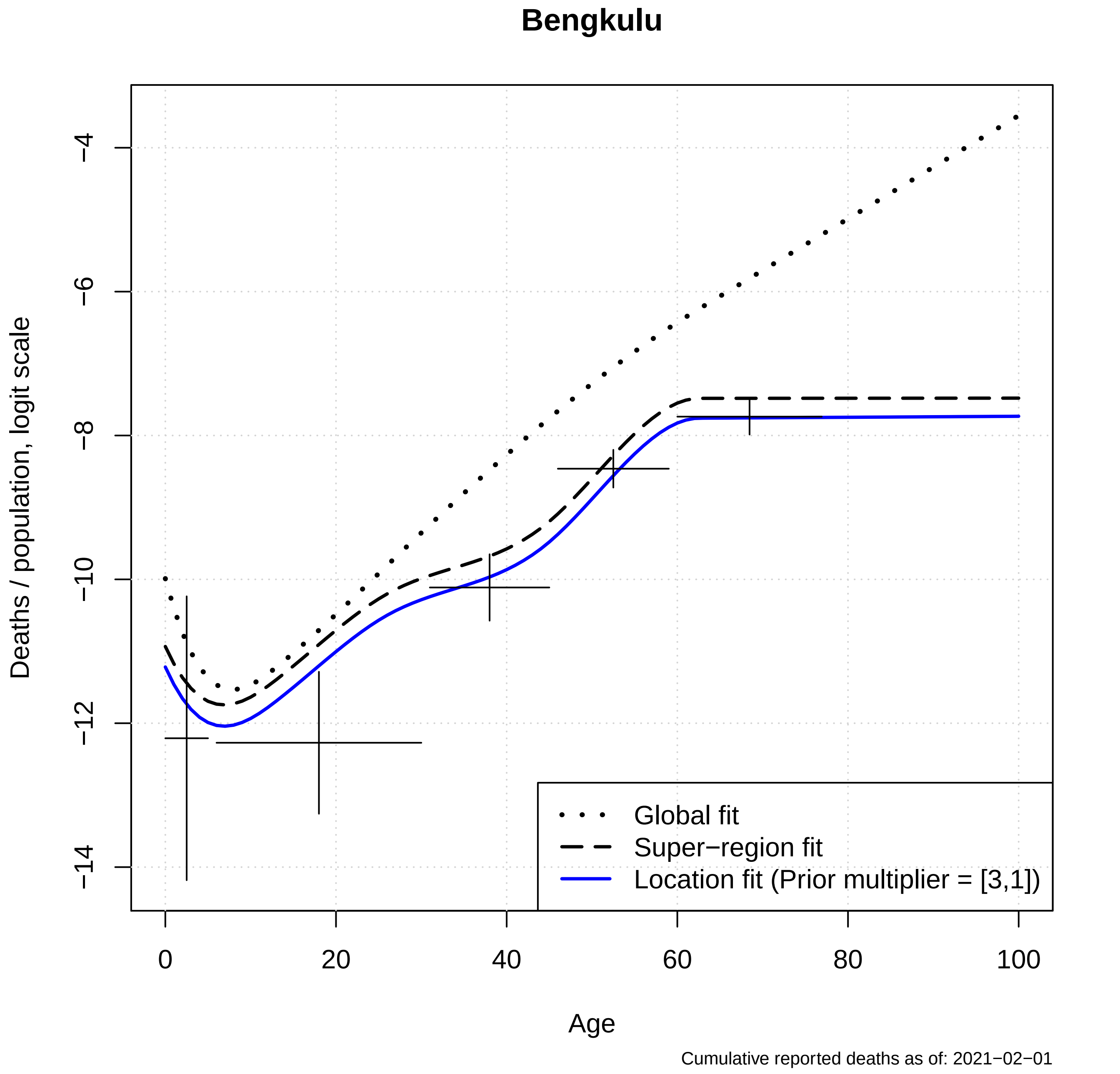
Bengkulu
Deaths / population, logit scale
−4
−6
−8
−10
−12
−14
0
20
40
60
80
100
Age
Global fit
Super−region fit
Location fit (Prior multiplier = [3,1])
Cumulative reported deaths as of: 2021−02−01

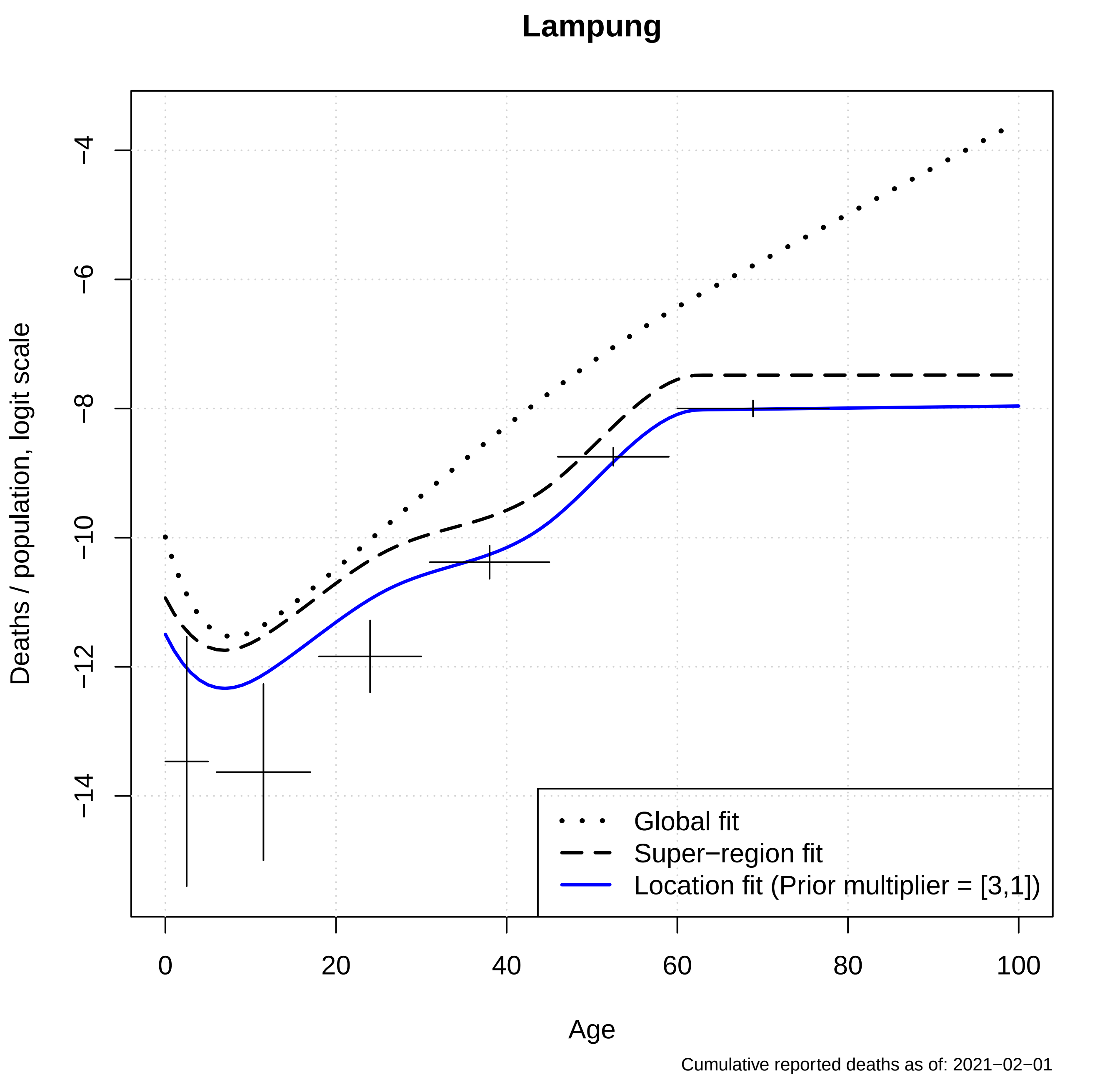
Lampung
Deaths / population, logit scale
−4
−6
−8
−10
−12
−14
0
20
40
60
80
100
Age
Global fit
Super−region fit
Location fit (Prior multiplier = [3,1])
Cumulative reported deaths as of: 2021−02−01

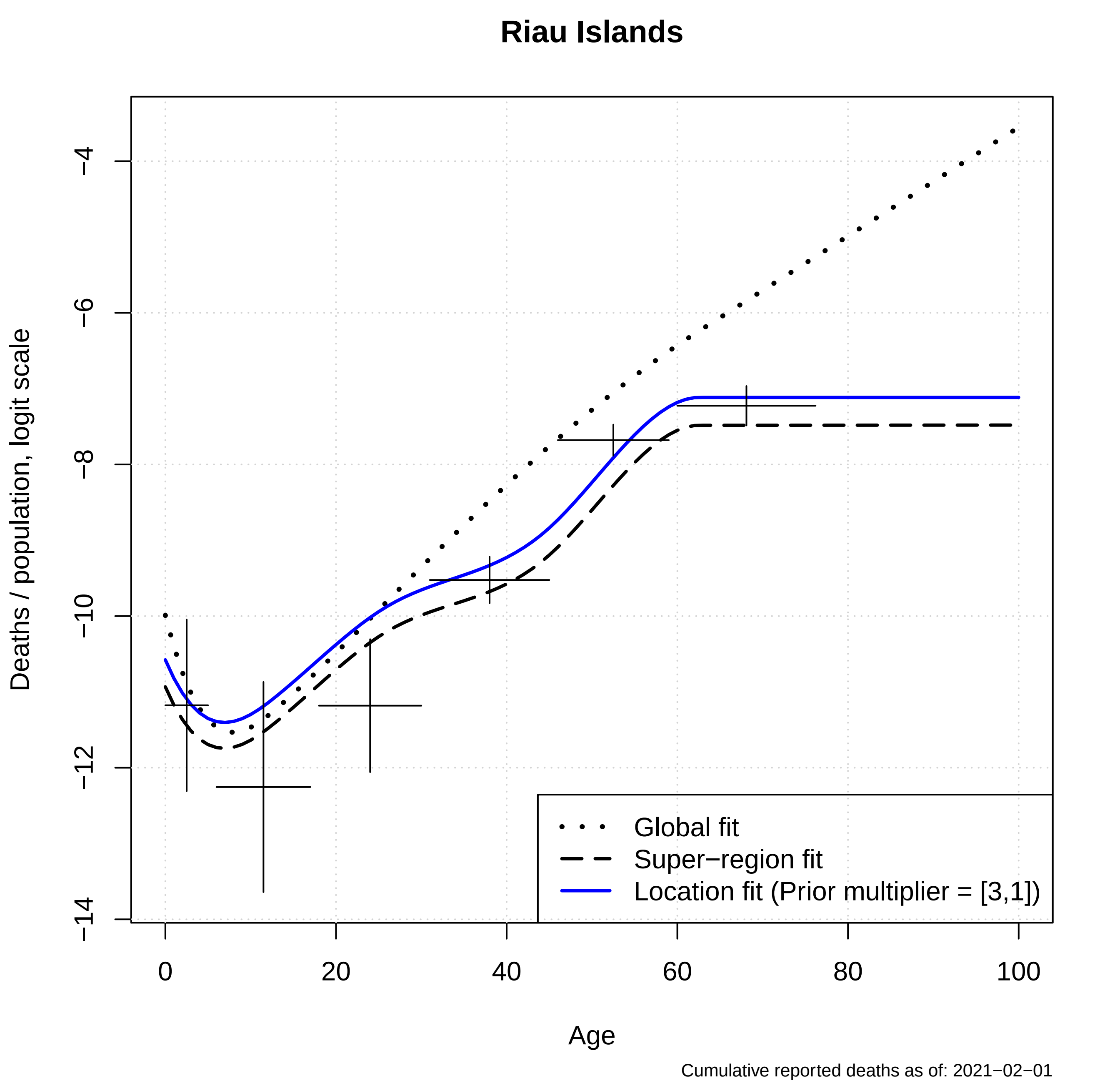

Riau Islands
Deaths / population, logit scale
−4
−6
−8
−10
−12
−14
0
20
40
60
80
100
Age
Global fit
Super−region fit
Location fit (Prior multiplier = [3,1])
Cumulative reported deaths as of: 2021−02−01

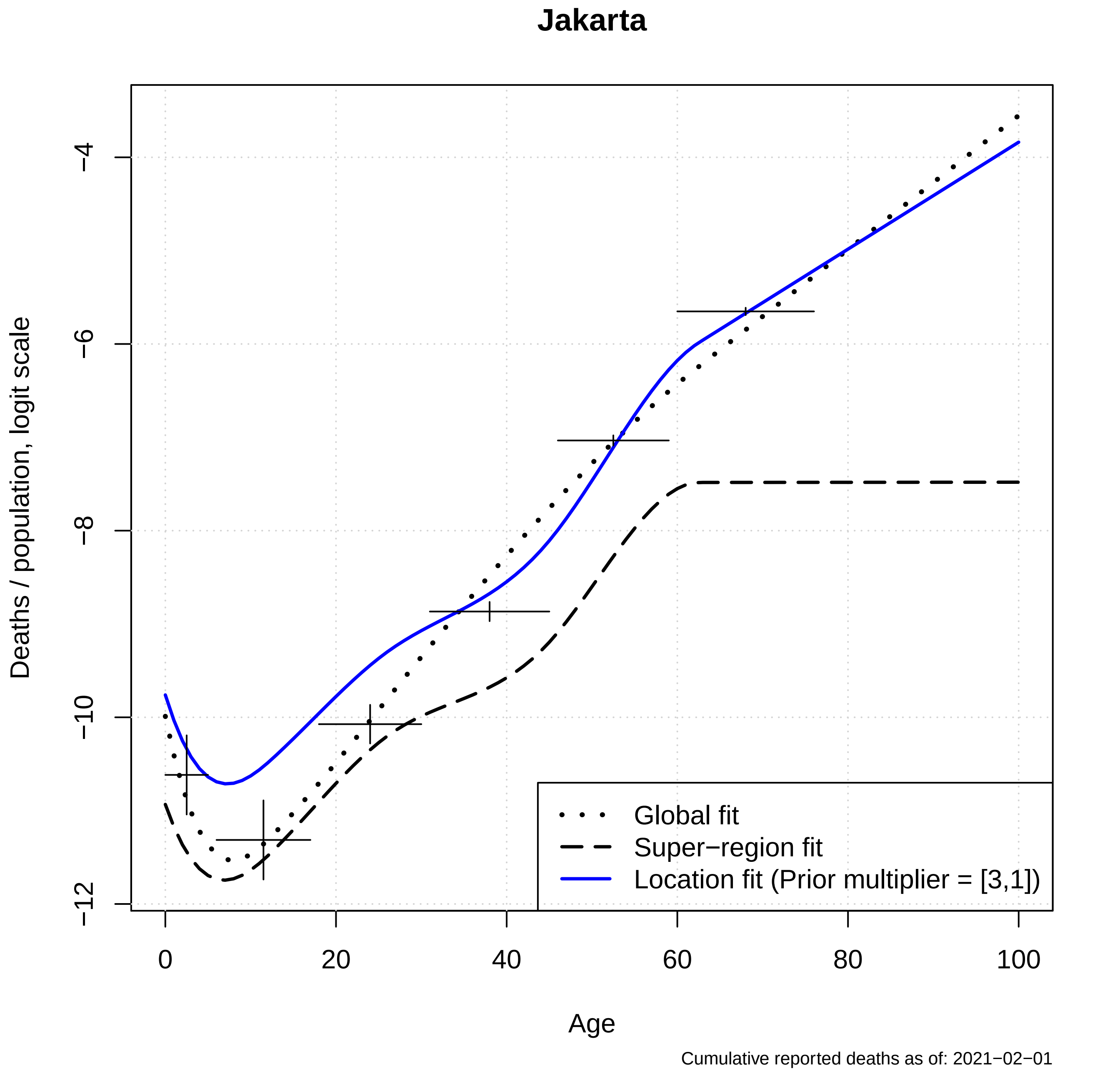

Jakarta
Deaths / population, logit scale
−4
−6
−8
−10
−12
0
20
40
60
80
100
Age
Global fit
Super−region fit
Location fit (Prior multiplier = [3,1])
Cumulative reported deaths as of: 2021−02−01

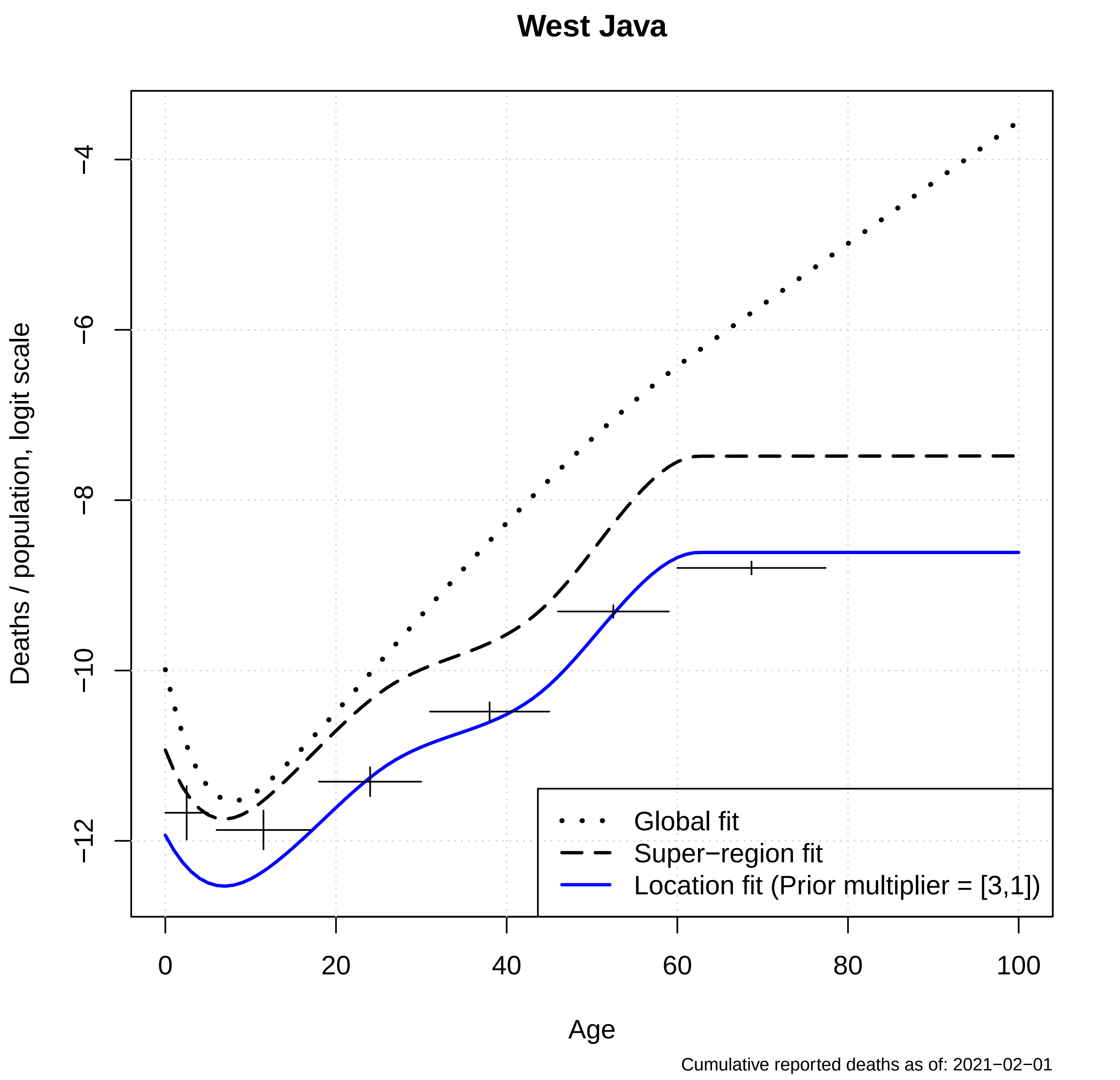
West Java
Deaths / population, logit scale
−4
−6
−8
−10
−12
0
20
40
60
80
100
Age
Global fit
Super−region fit
Location fit (Prior multiplier = [3,1])
Cumulative reported deaths as of: 2021−02−01

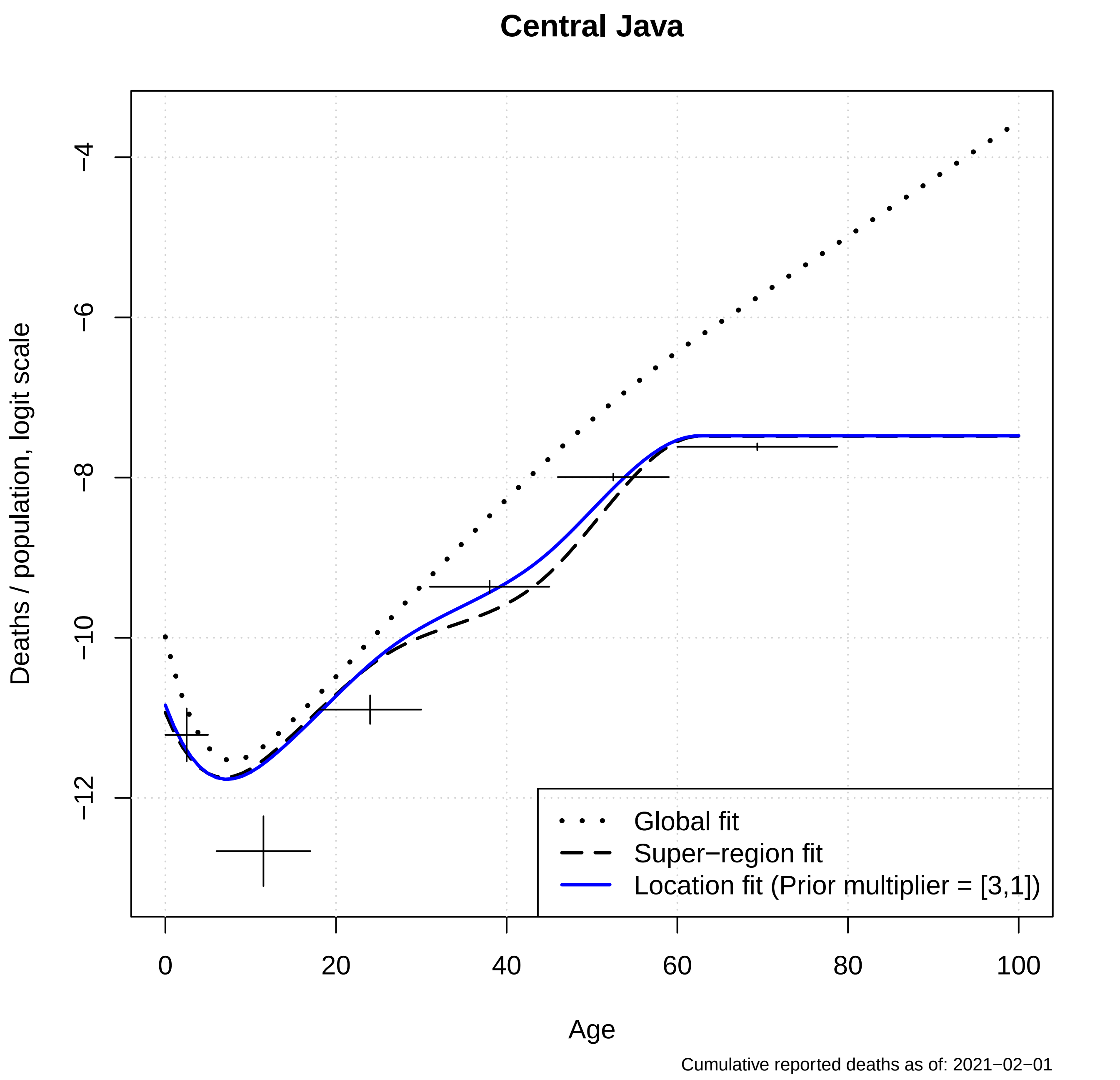

Central Java
Deaths / population, logit scale
−4
−6
−8
−10
−12
0
20
40
60
80
100
Age
Global fit
Super−region fit
Location fit (Prior multiplier = [3,1])
Cumulative reported deaths as of: 2021−02−01

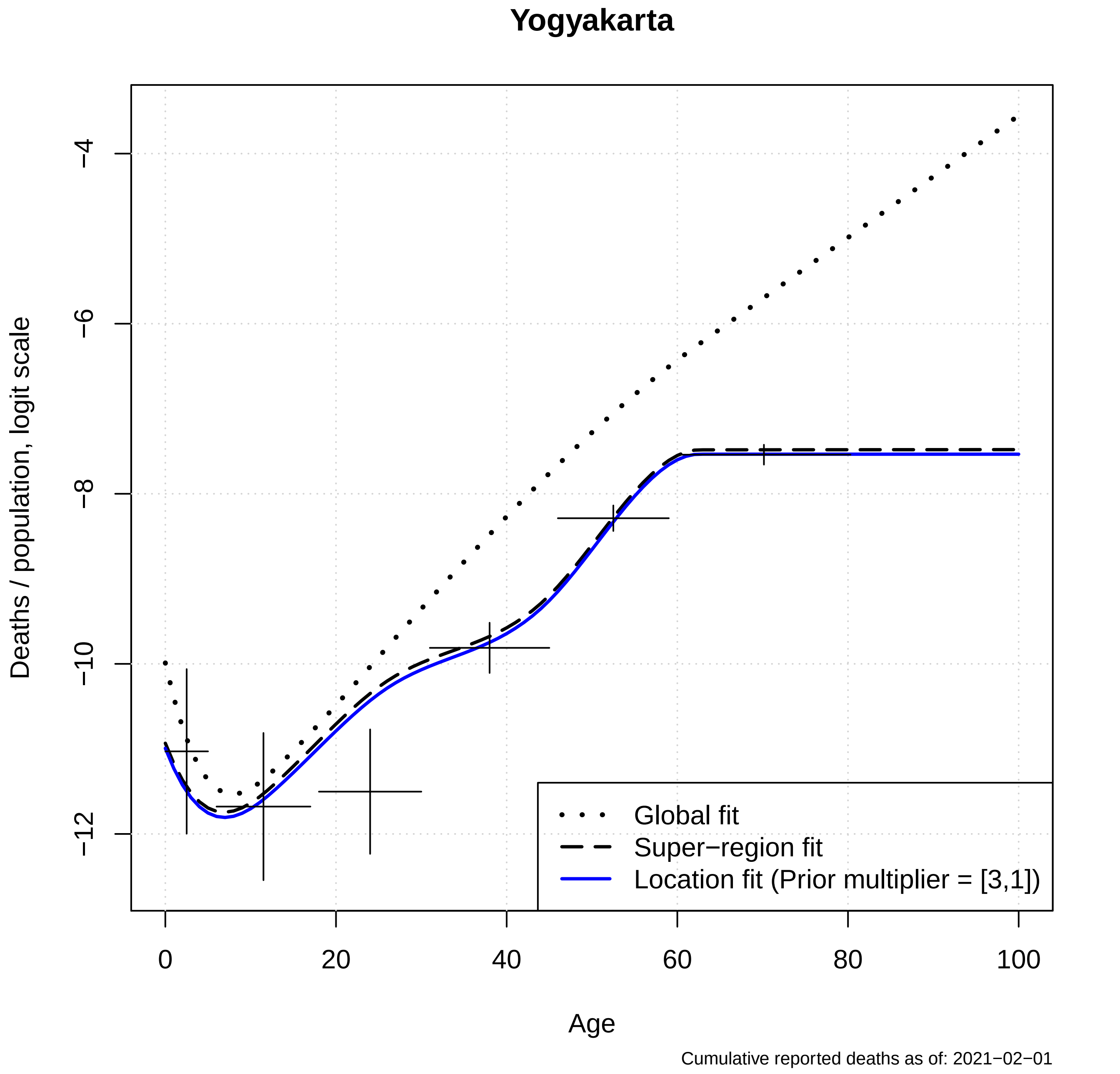

Yogyakarta
Deaths / population, logit scale
−4
−6
−8
−10
−12
0
20
40
60
80
100
Age
Global fit
Super−region fit
Location fit (Prior multiplier = [3,1])
Cumulative reported deaths as of: 2021−02−01

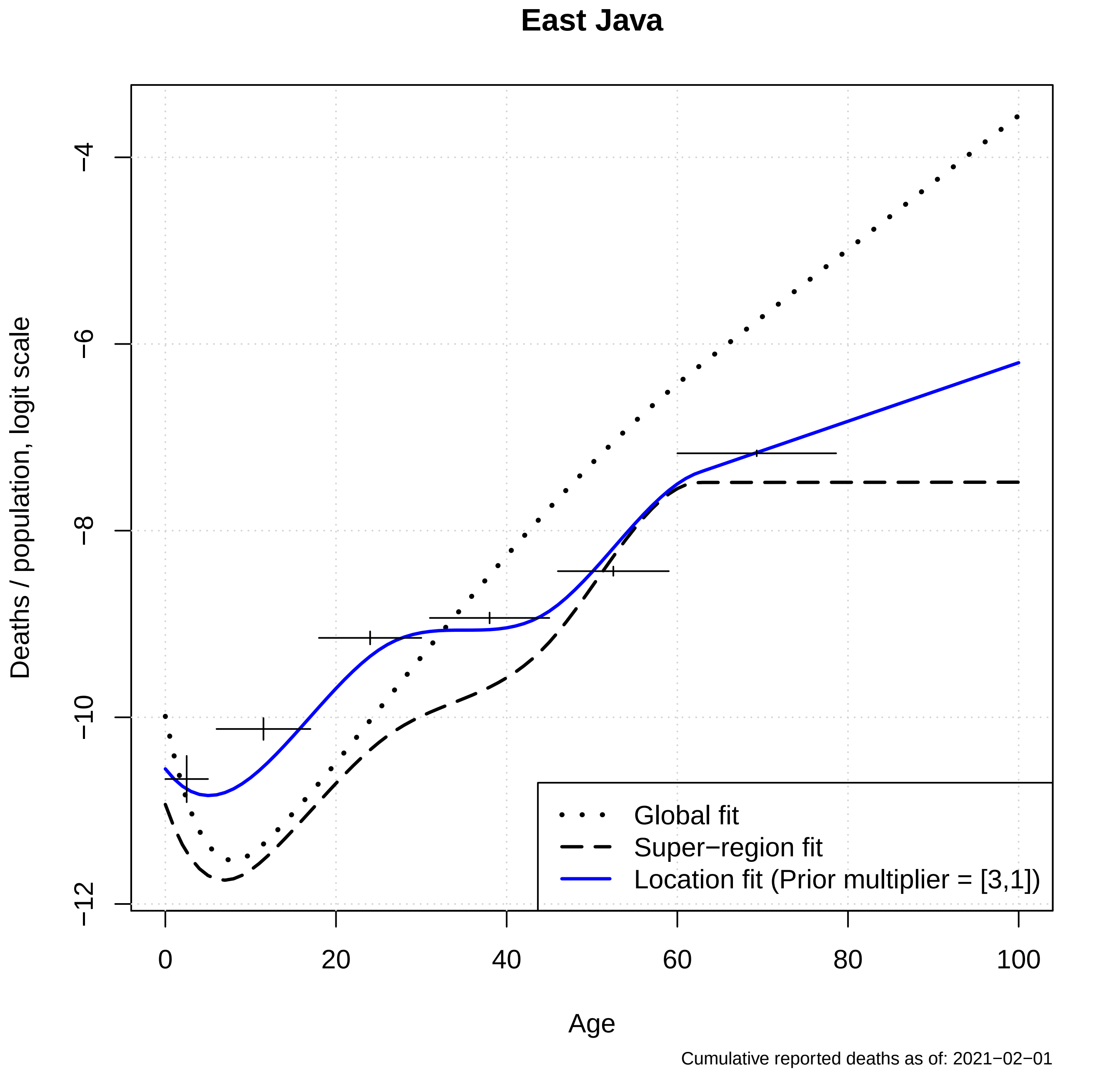
East Java
Deaths / population, logit scale
−4
−6
−8
−10
−12
Age
0
20
40
60
80
100
Global fit
Super−region fit
Location fit (Prior multiplier = [3,1])
Cumulative reported deaths as of: 2021−02−01

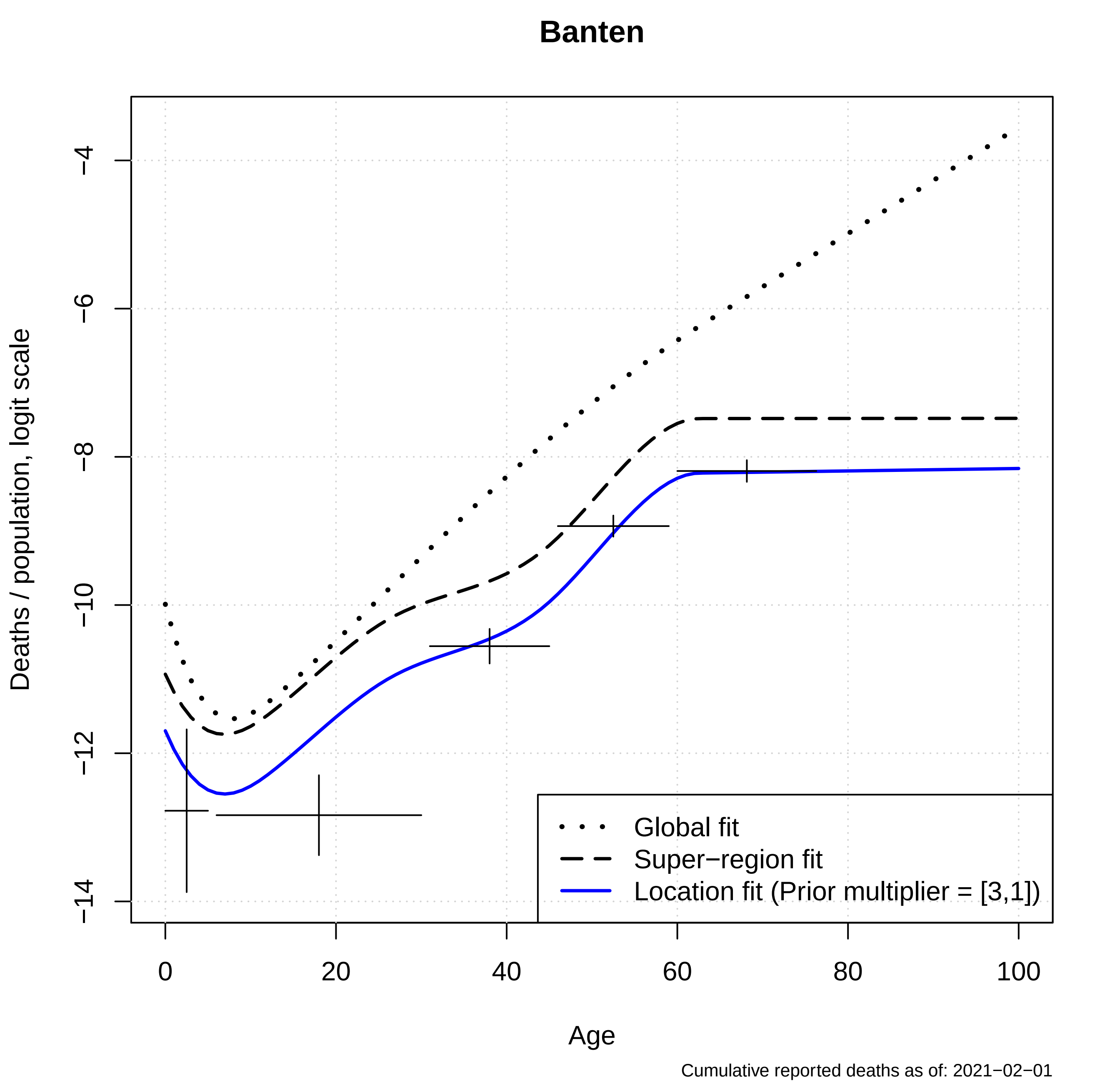

Banten
Deaths / population, logit scale
−4
−6
−8
−10
−12
−14
0
20
40
60
80
100
Age
Global fit
Super−region fit
Location fit (Prior multiplier = [3,1])
Cumulative reported deaths as of: 2021−02−01

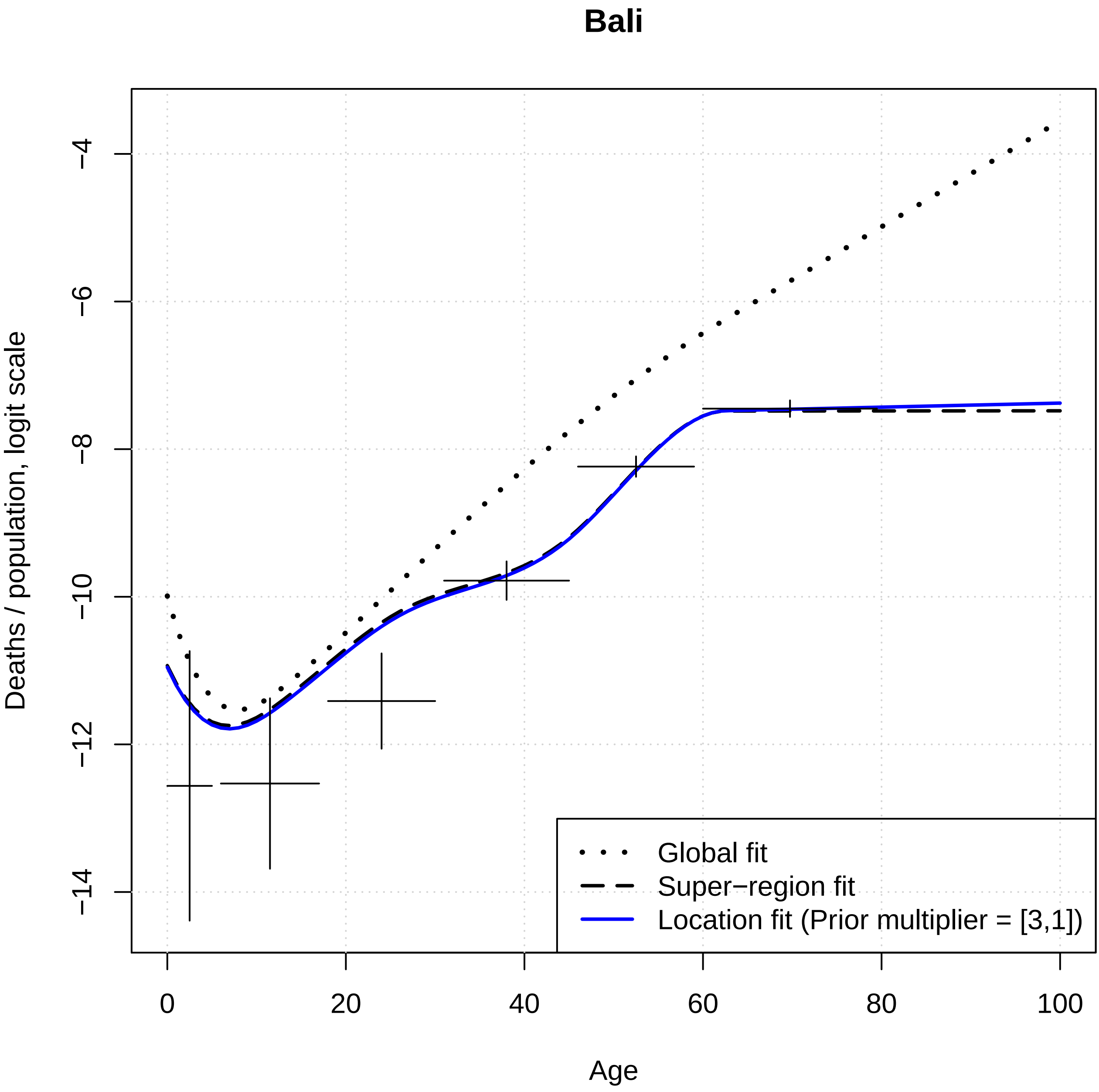

Cumulative reported deaths as of: 2021−02−01

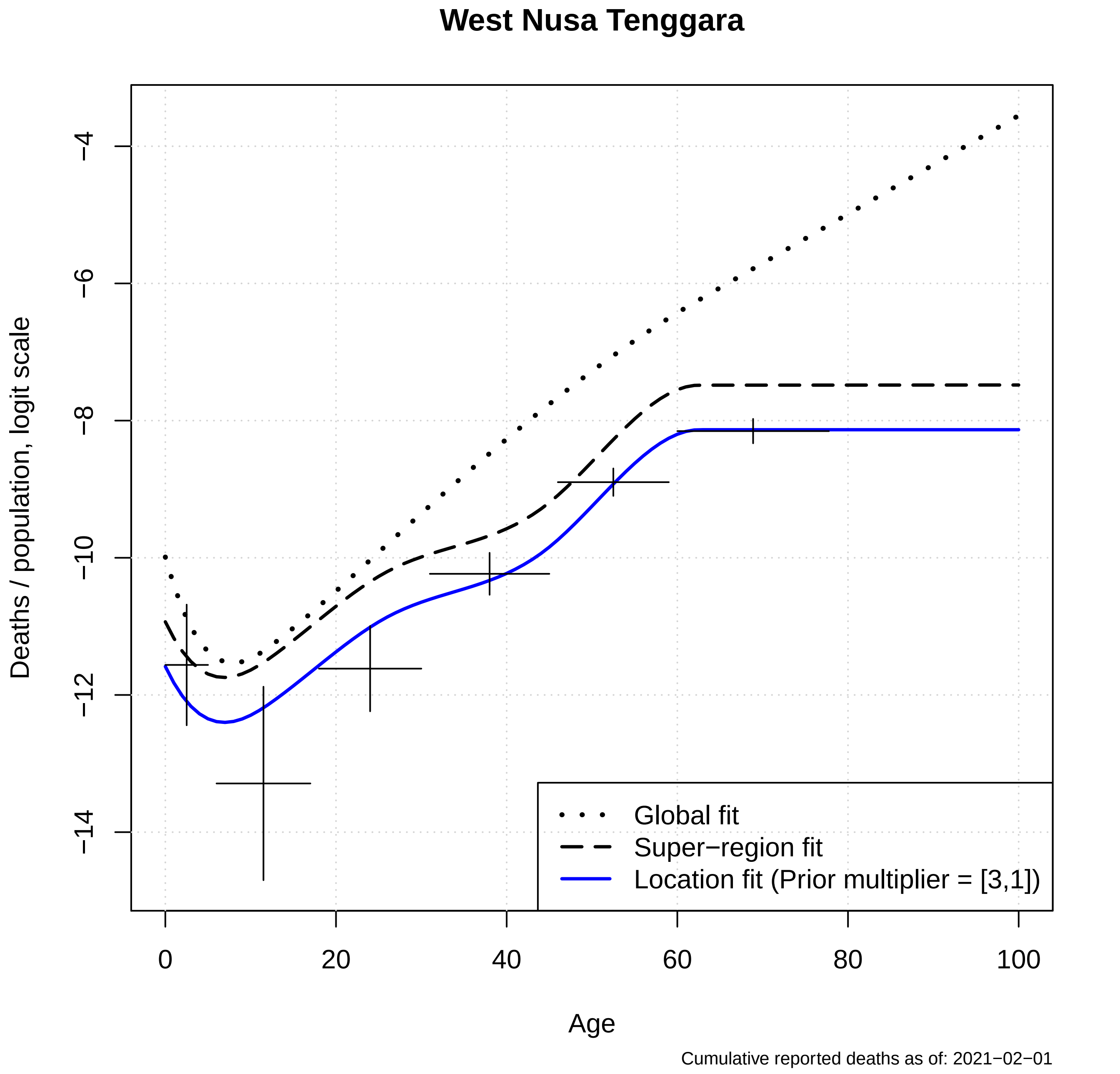
West Nusa Tenggara
Deaths / population, logit scale
−4
−6
−8
−10
−12
−14
0
20
40
60
80
100
Age
Global fit
Super−region fit
Location fit (Prior multiplier = [3,1])
Cumulative reported deaths as of: 2021−02−01

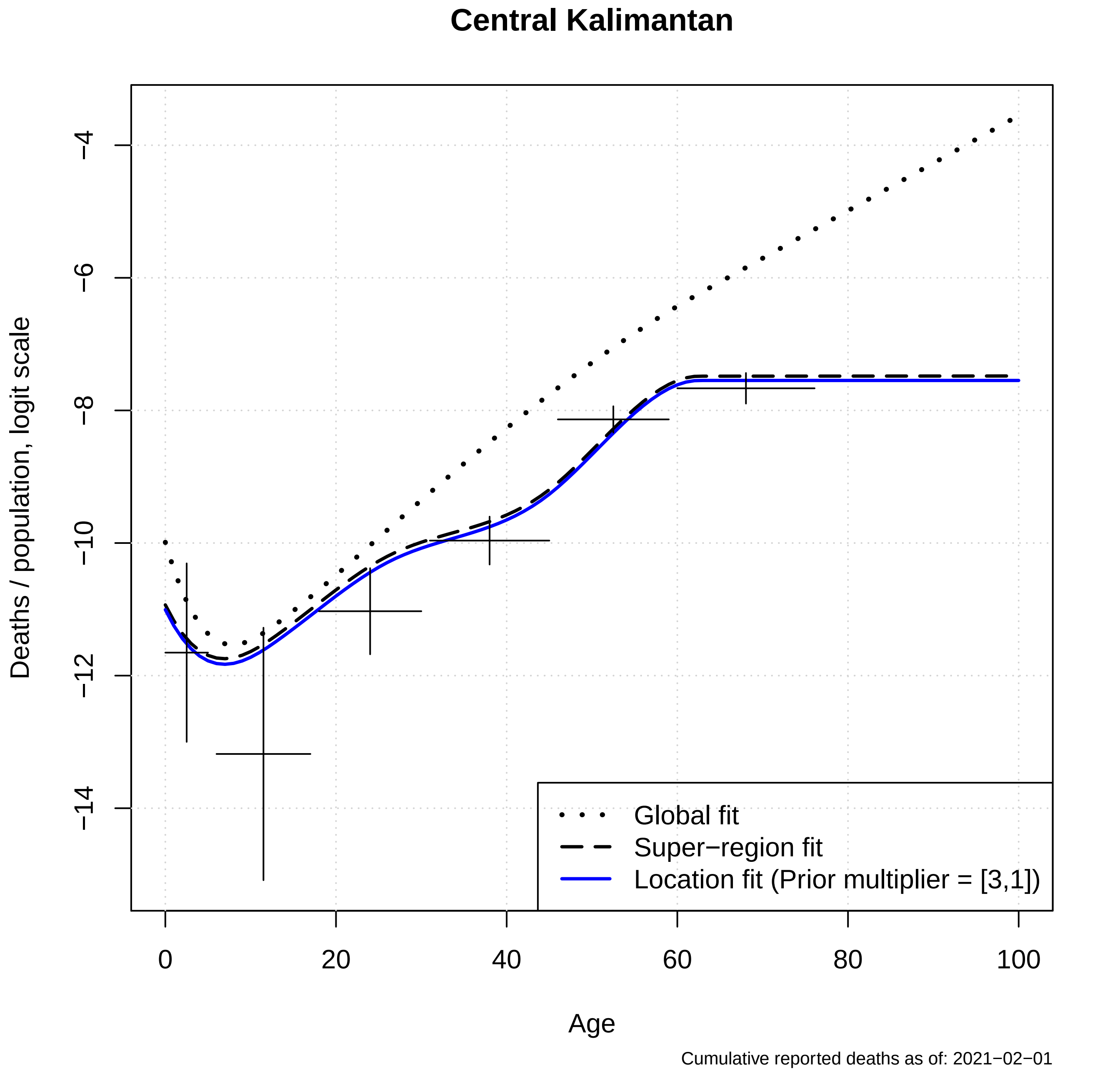

Central Kalimantan
Deaths / population, logit scale
−4
−6
−8
−10
−12
−14
0
20
40
60
80
100
Age
Global fit
Super−region fit
Location fit (Prior multiplier = [3,1])
Cumulative reported deaths as of: 2021−02−01

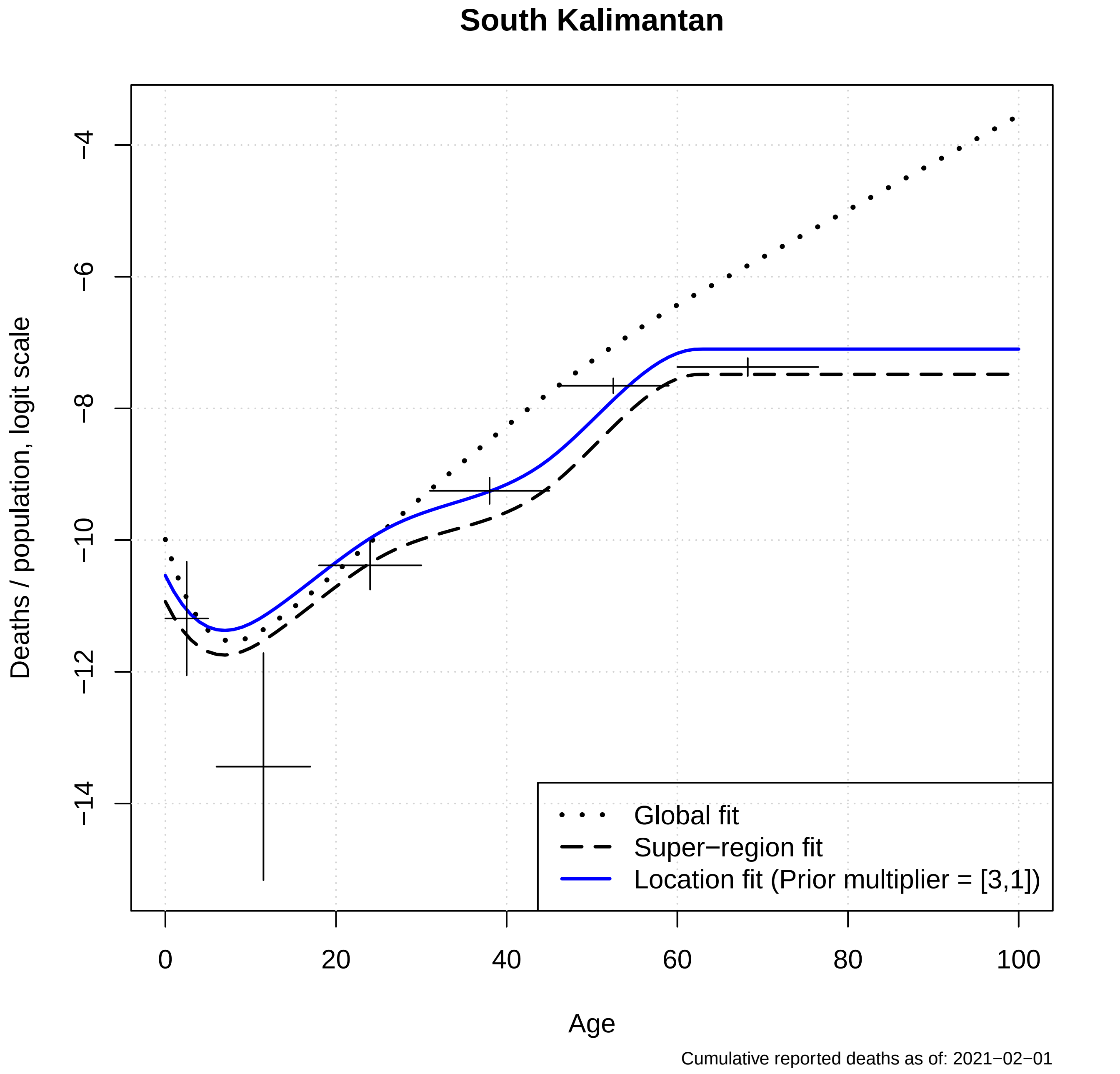
South Kalimantan
Deaths / population, logit scale
−4
−6
−8
−10
−12
−14
0
20
40
60
80
100
Age
Global fit
Super−region fit
Location fit (Prior multiplier = [3,1])
Cumulative reported deaths as of: 2021−02−01

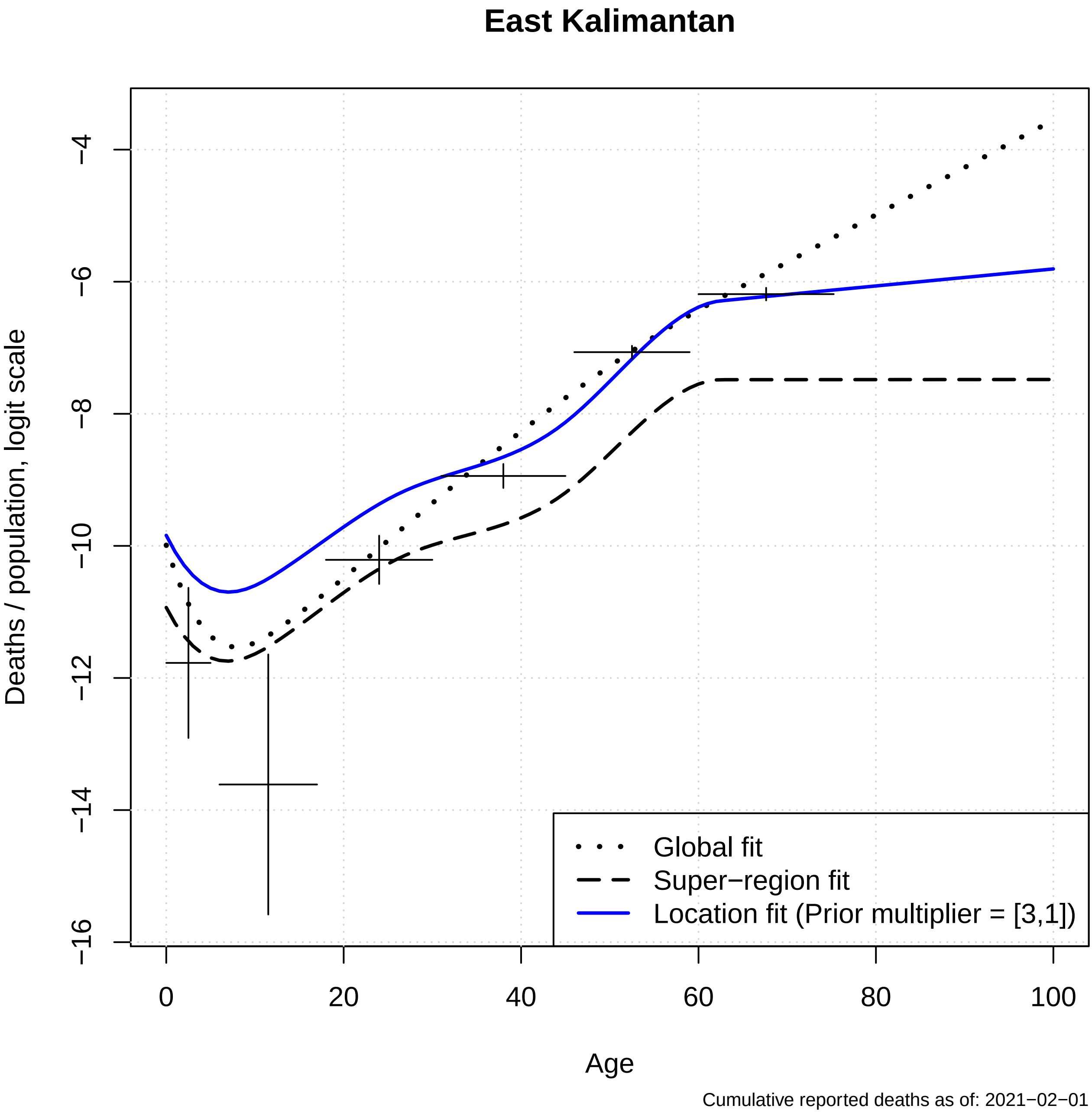
East Kalimantan
Deaths / population, logit scale
−4
−6
−8
−10
−12
−14
−16
0
20
40
60
80
100
Age
Global fit
Super−region fit
Location fit (Prior multiplier = [3,1])
Cumulative reported deaths as of: 2021−02−01

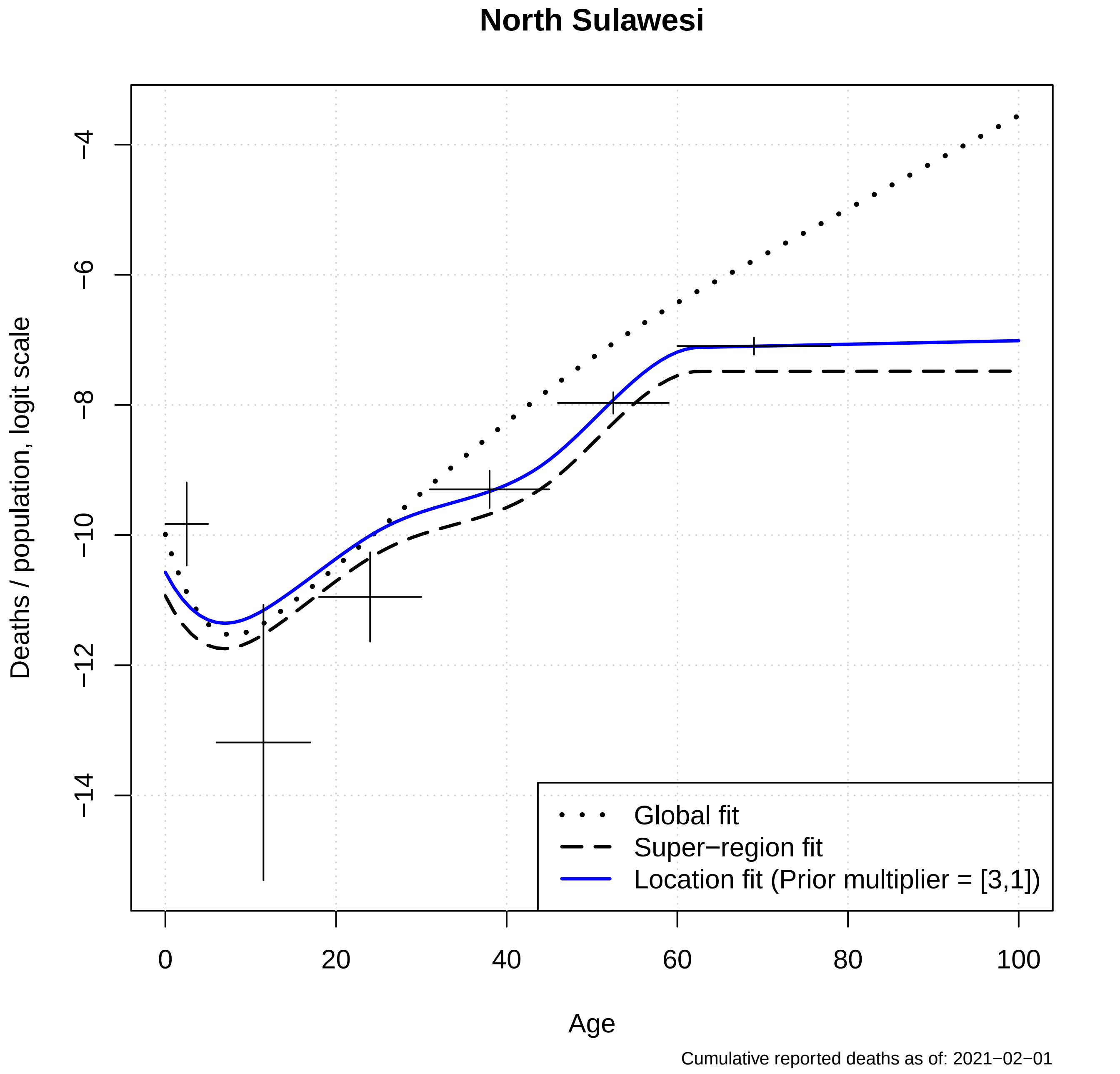

North Sulawesi
Deaths / population, logit scale
−4
−6
−8
−10
−12
−14
0
20
40
60
80
100
Age
Global fit
Super−region fit
Location fit (Prior multiplier = [3,1])
Cumulative reported deaths as of: 2021−02−01

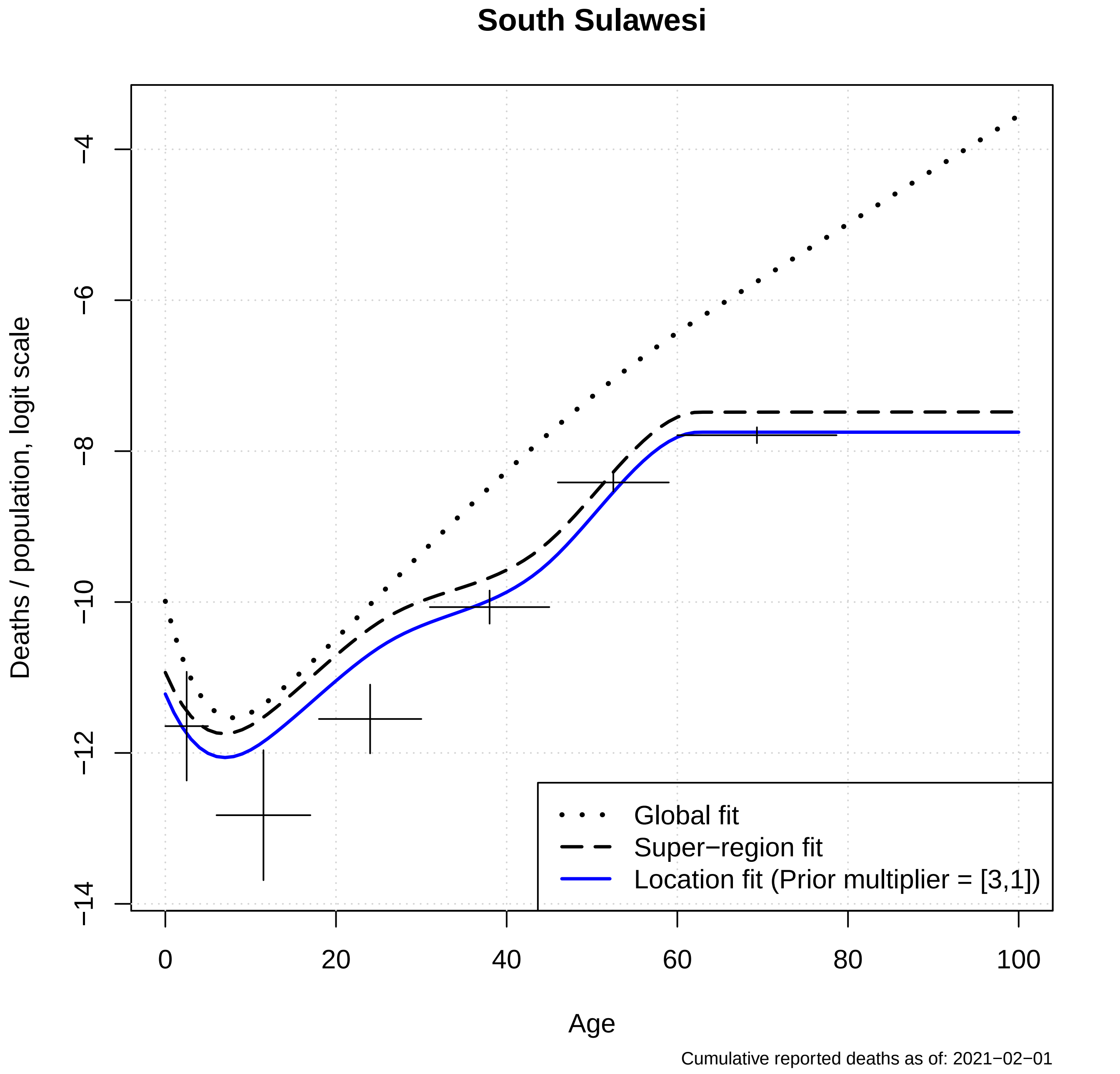
South Sulawesi
Deaths / population, logit scale
−4
−6
−8
−10
−12
−14
0
20
40
60
80
100
Age
Global fit
Super−region fit
Location fit (Prior multiplier = [3,1])
Cumulative reported deaths as of: 2021−02−01

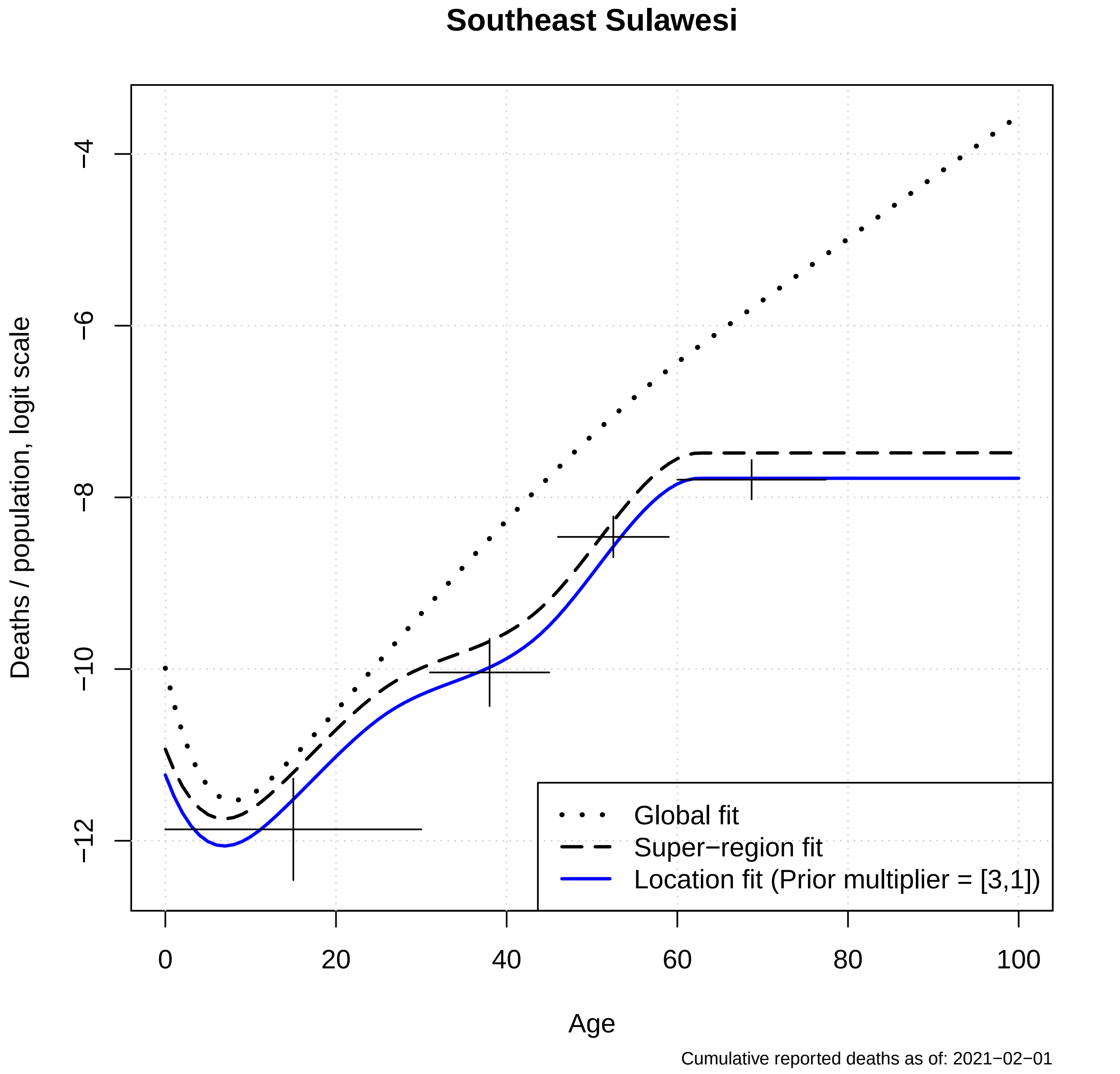
Southeast Sulawesi
Deaths / population, logit scale
−4
−6
−8
−10
−12
0
20
40
60
80
100
Age
Global fit
Super−region fit
Location fit (Prior multiplier = [3,1])
Cumulative reported deaths as of: 2021−02−01

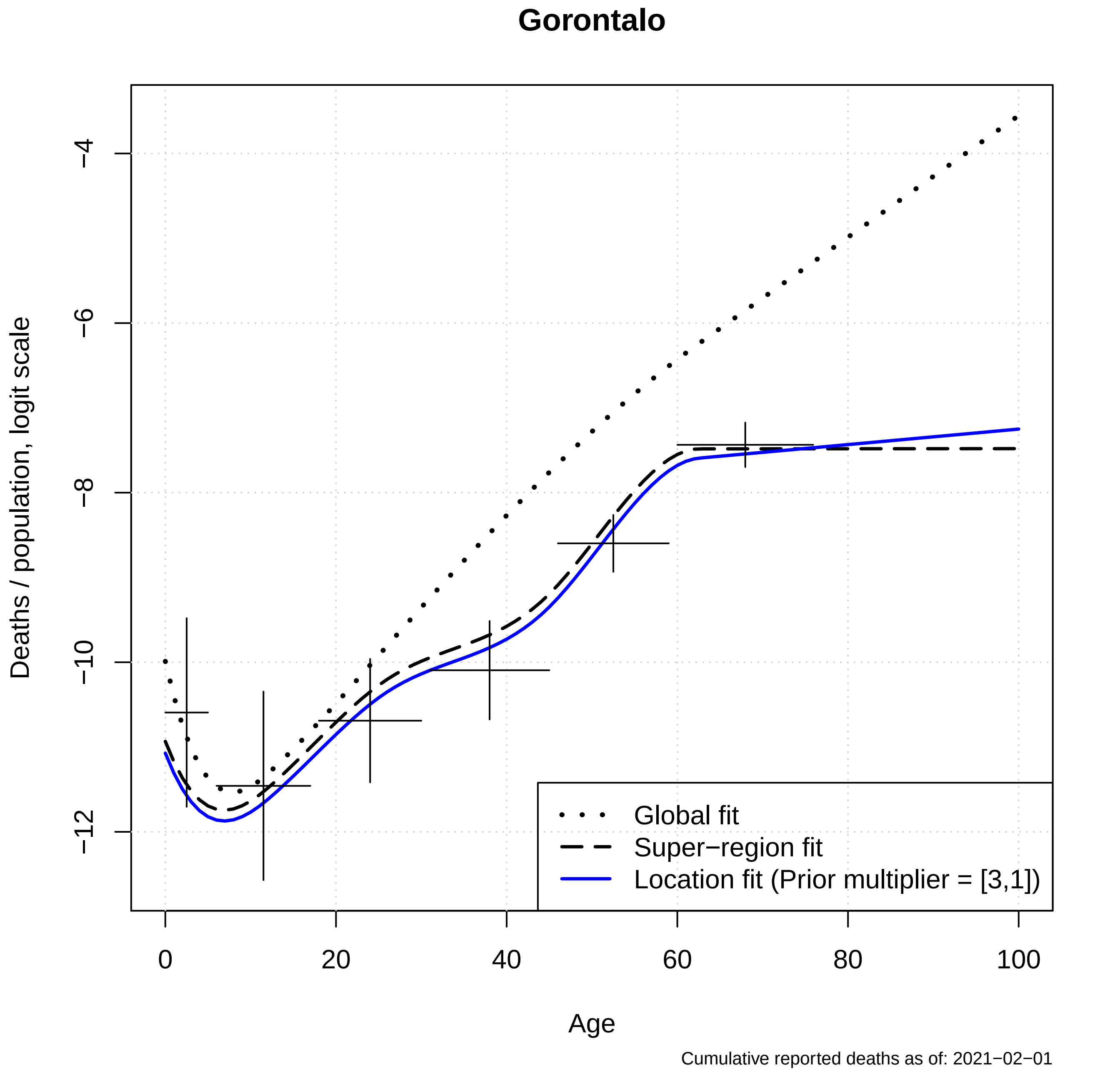

Gorontalo
Deaths / population, logit scale
−4
−6
−8
−10
−12
0
20
40
60
80
100
Age
Global fit
Super−region fit
Location fit (Prior multiplier = [3,1])
Cumulative reported deaths as of: 2021−02−01

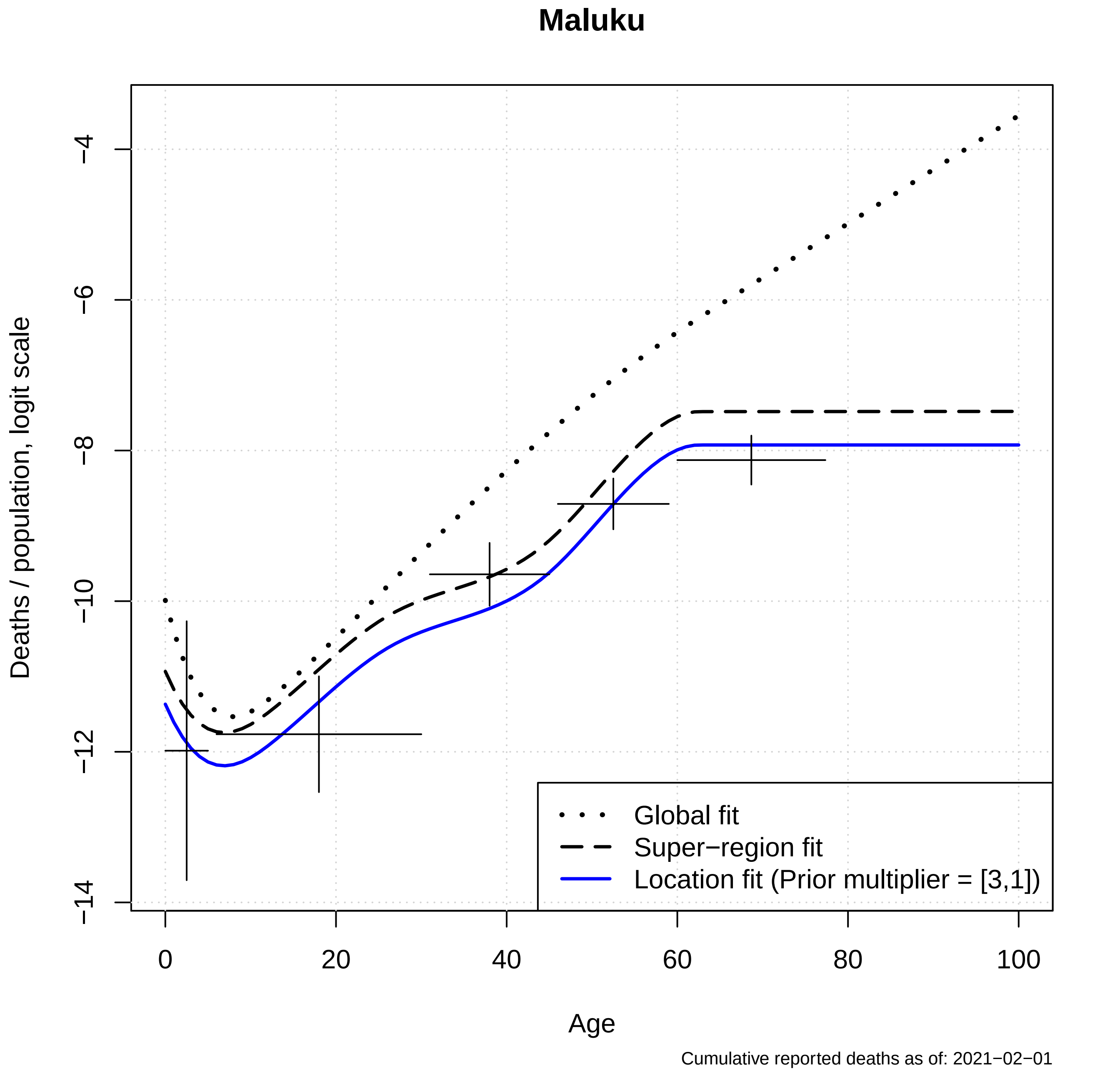

Maluku
Deaths / population, logit scale
−4
−6
−8
−10
−12
−14
0
20
40
60
80
100
Age
Global fit
Super−region fit
Location fit (Prior multiplier = [3,1])
Cumulative reported deaths as of: 2021−02−01

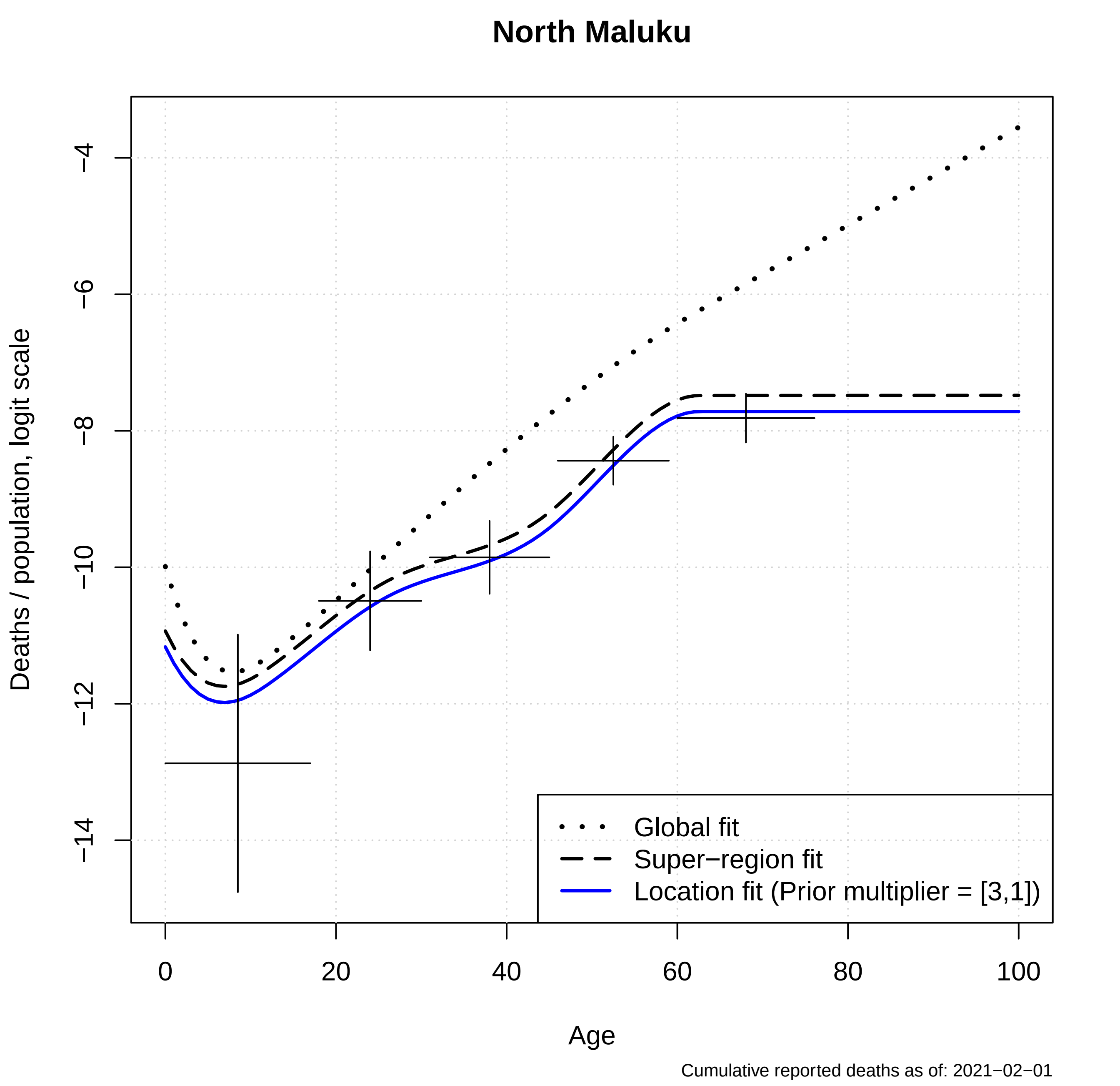
North Maluku
Deaths / population, logit scale
−4
−6
−8
−10
−12
−14
0
20
40
60
80
100
Age
Global fit
Super−region fit
Location fit (Prior multiplier = [3,1])
Cumulative reported deaths as of: 2021−02−01

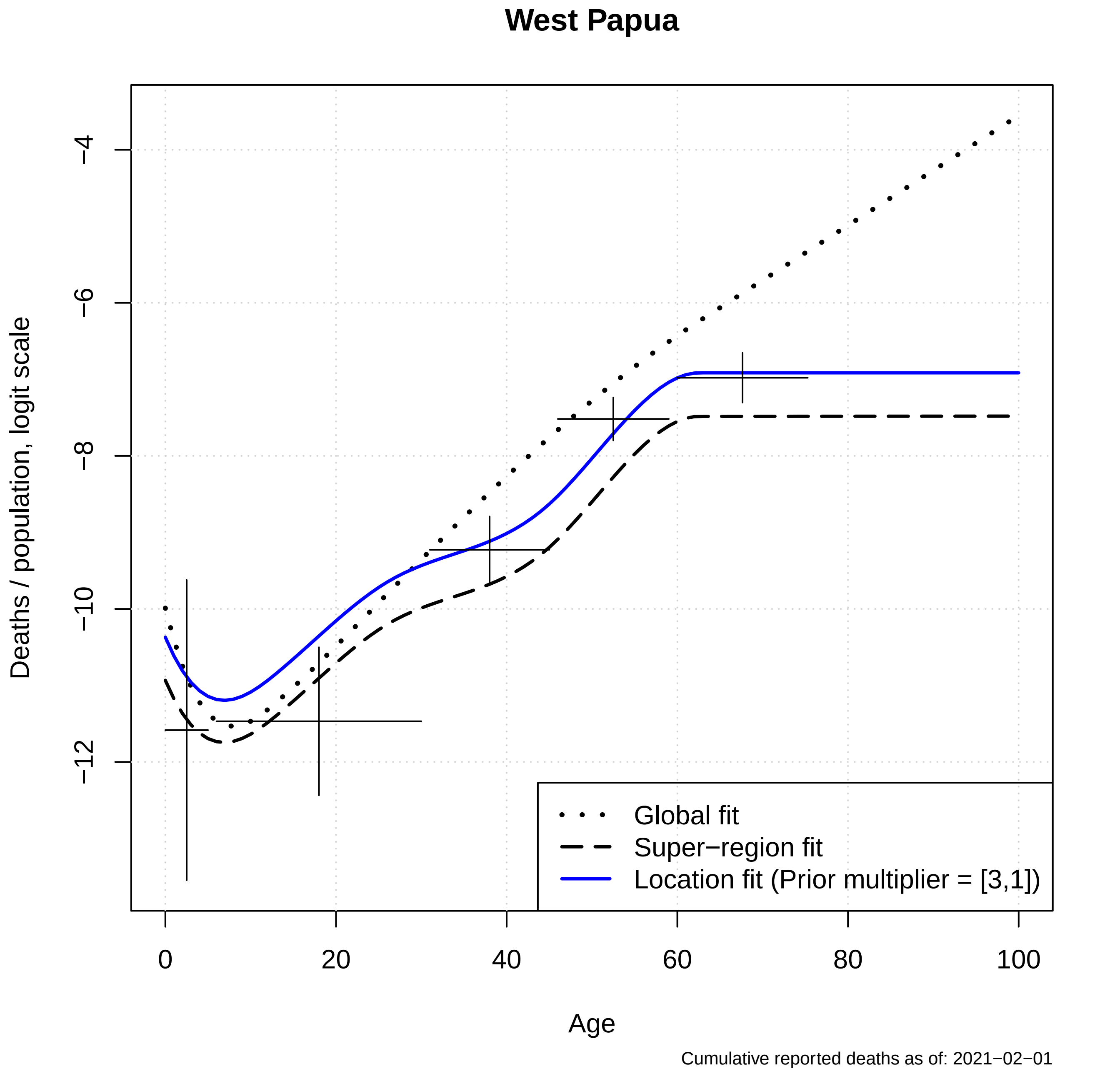
West Papua
Deaths / population, logit scale
−4
−6
−8
−10
−12
0
20
40
60
80
100
Age
Global fit
Super−region fit
Location fit (Prior multiplier = [3,1])
Cumulative reported deaths as of: 2021−02−01

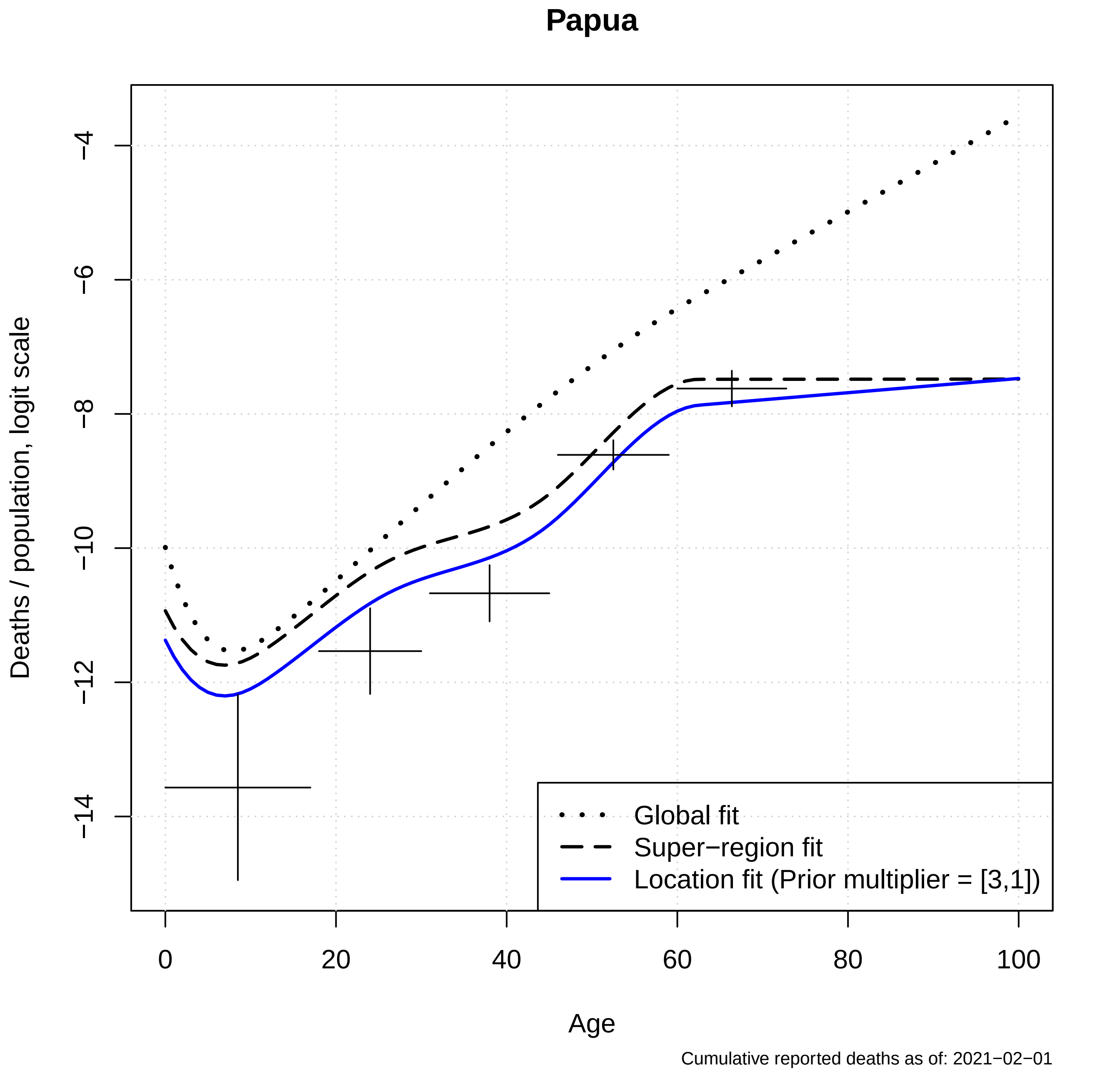

Papua
−4
−6
−8
−10
−12
−14
Deaths / population, logit scale
0
20
40
60
80
100
Age
Global fit
Super−region fit
Location fit (Prior multiplier = [3,1])
Cumulative reported deaths as of: 2021−02−01

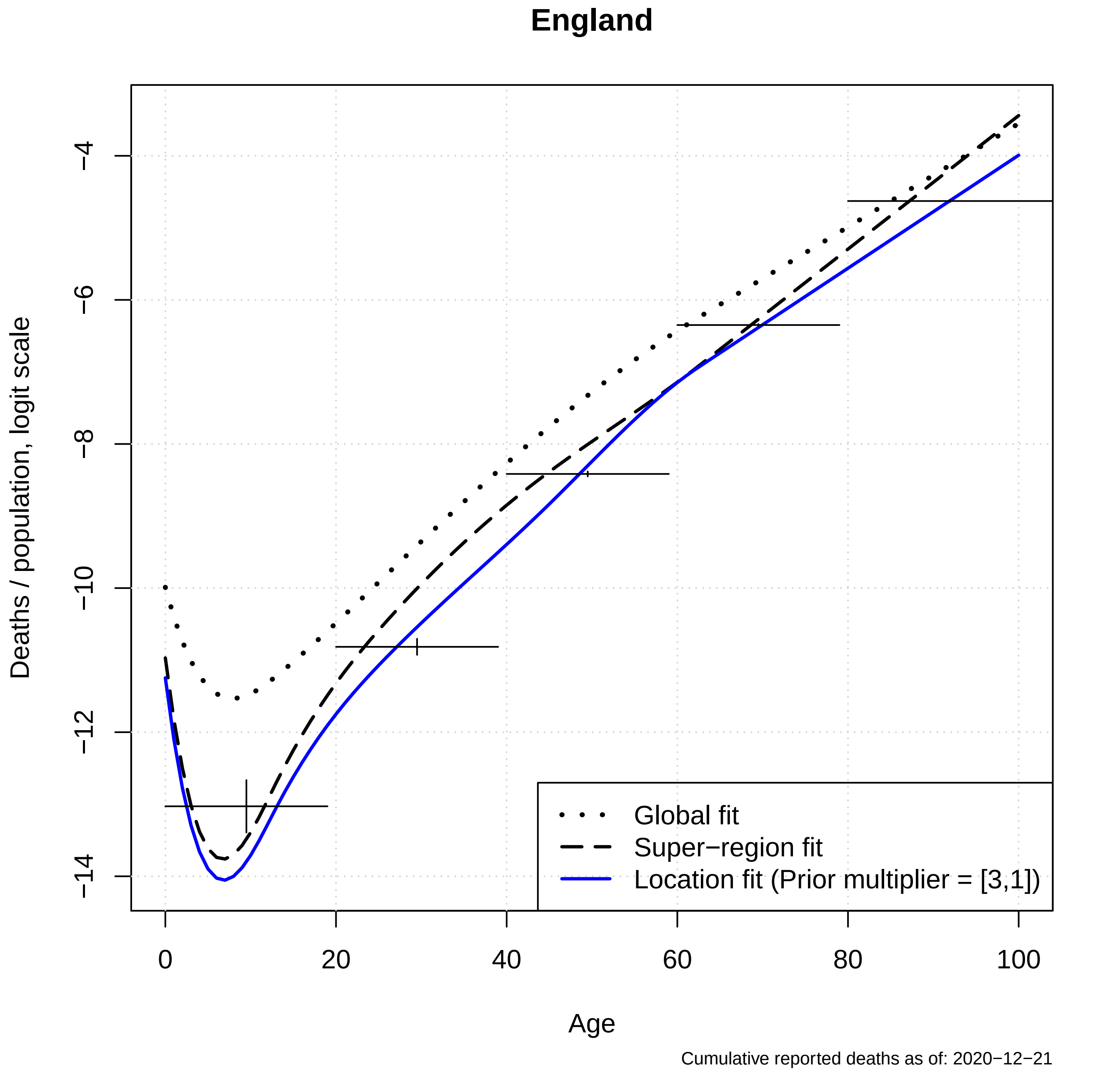
England
Deaths / population, logit scale
−4
−6
−8
−10
−12
−14
0
20
40
60
80
100
Age
Global fit
Super−region fit
Location fit (Prior multiplier = [3,1])
Cumulative reported deaths as of: 2020−12−21

Figure S8F. Brazil subnationals

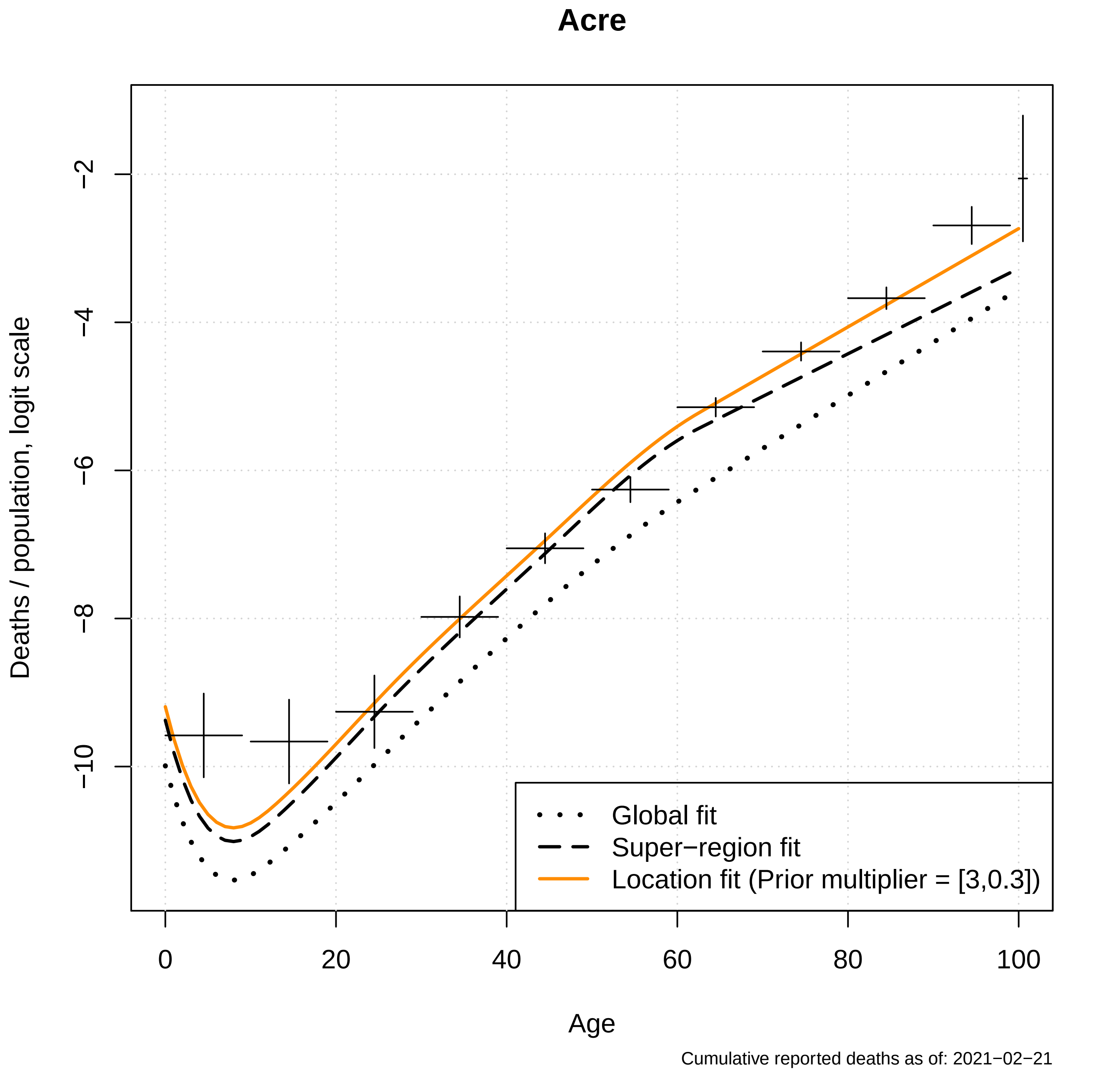

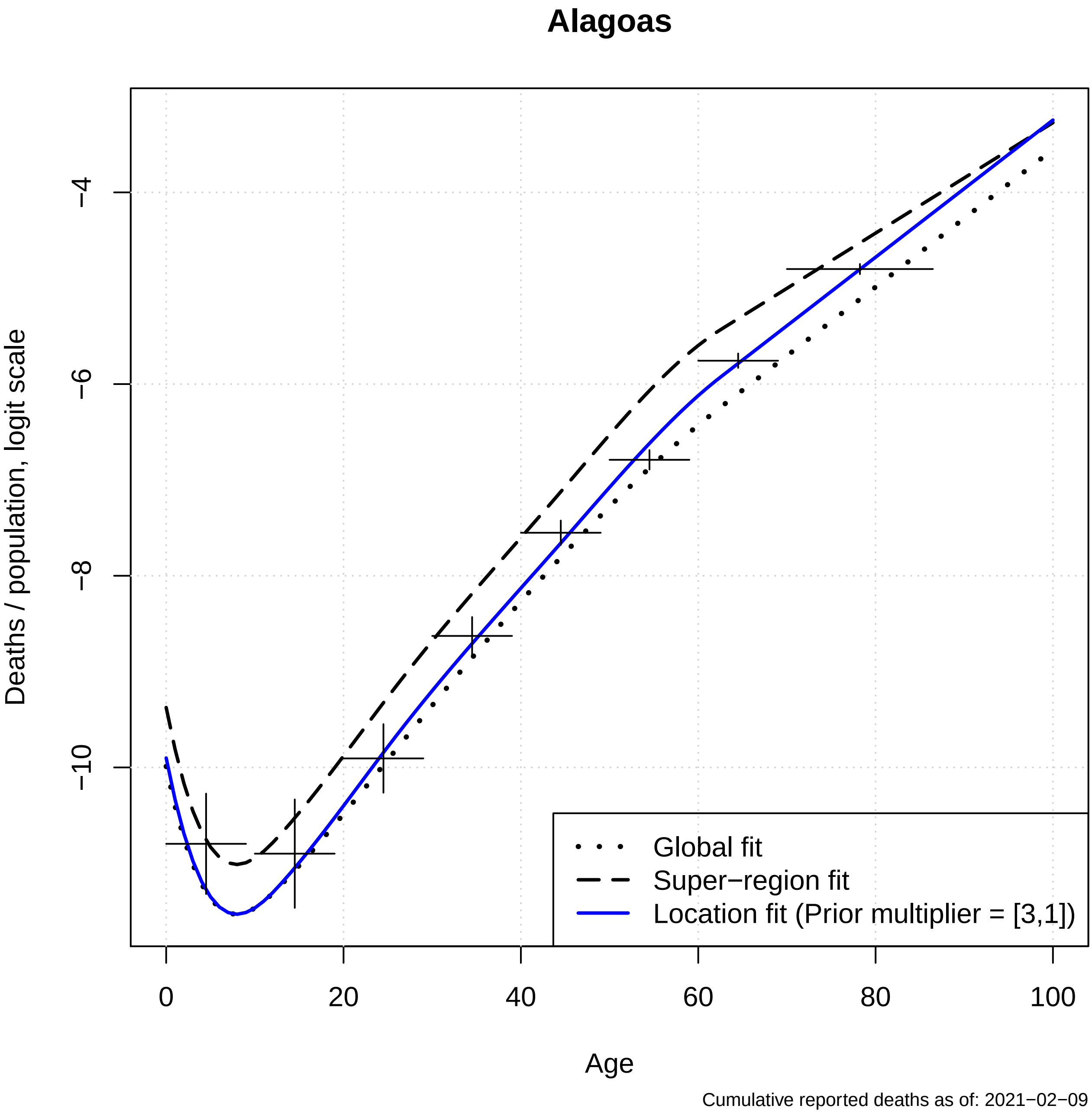

Alagoas
Deaths / population, logit scale
−4
−6
−8
−10
0
20
40
60
80
100
Age
Global fit
Super−region fit
Location fit (Prior multiplier = [3,1])
Cumulative reported deaths as of: 2021−02−09

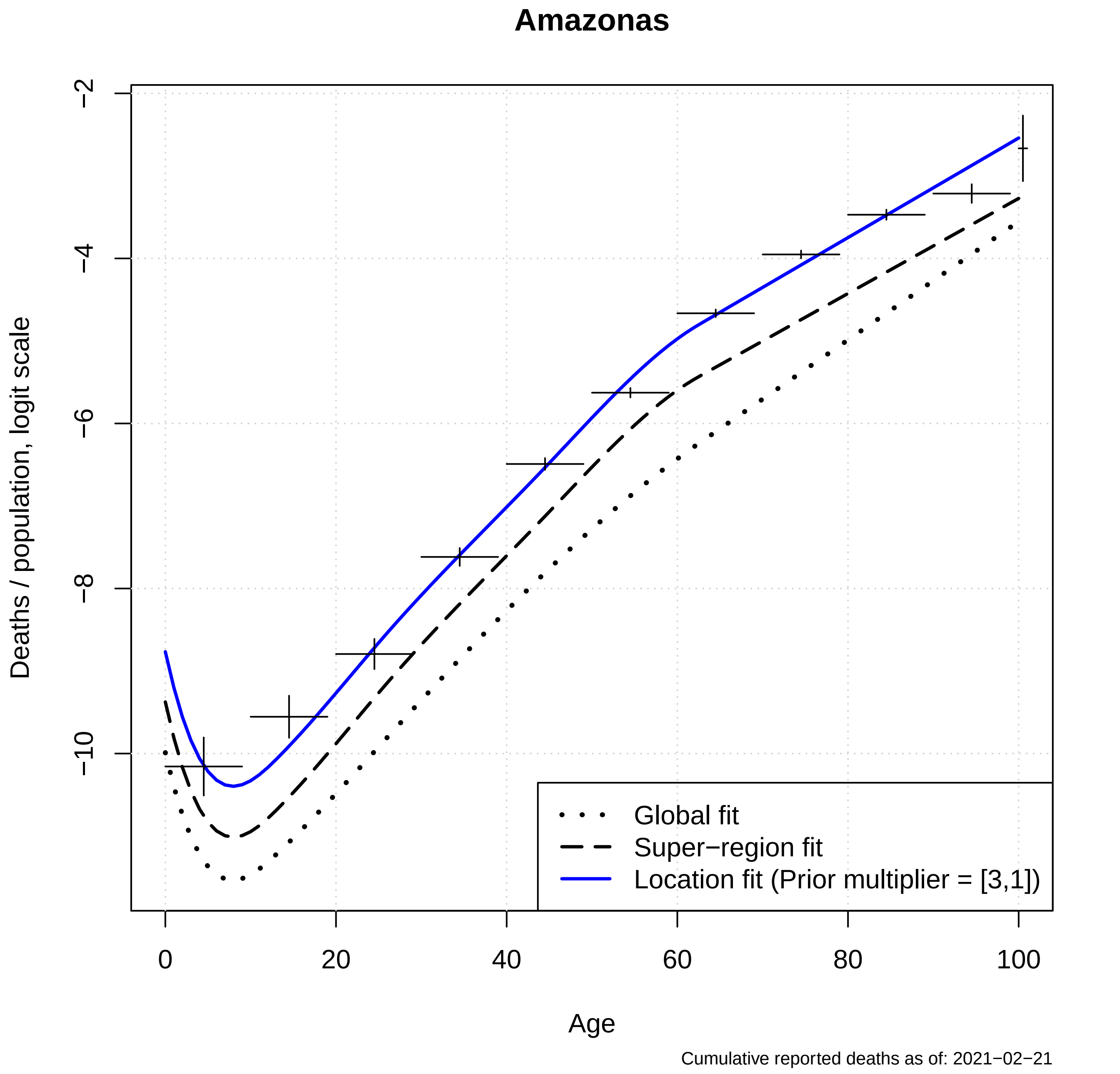
Amazonas
Deaths / population, logit scale
−2
−4
−6
−8
−10
0
20
40
60
80
100
Age
Global fit
Super−region fit
Location fit (Prior multiplier = [3,1])
Cumulative reported deaths as of: 2021−02−21

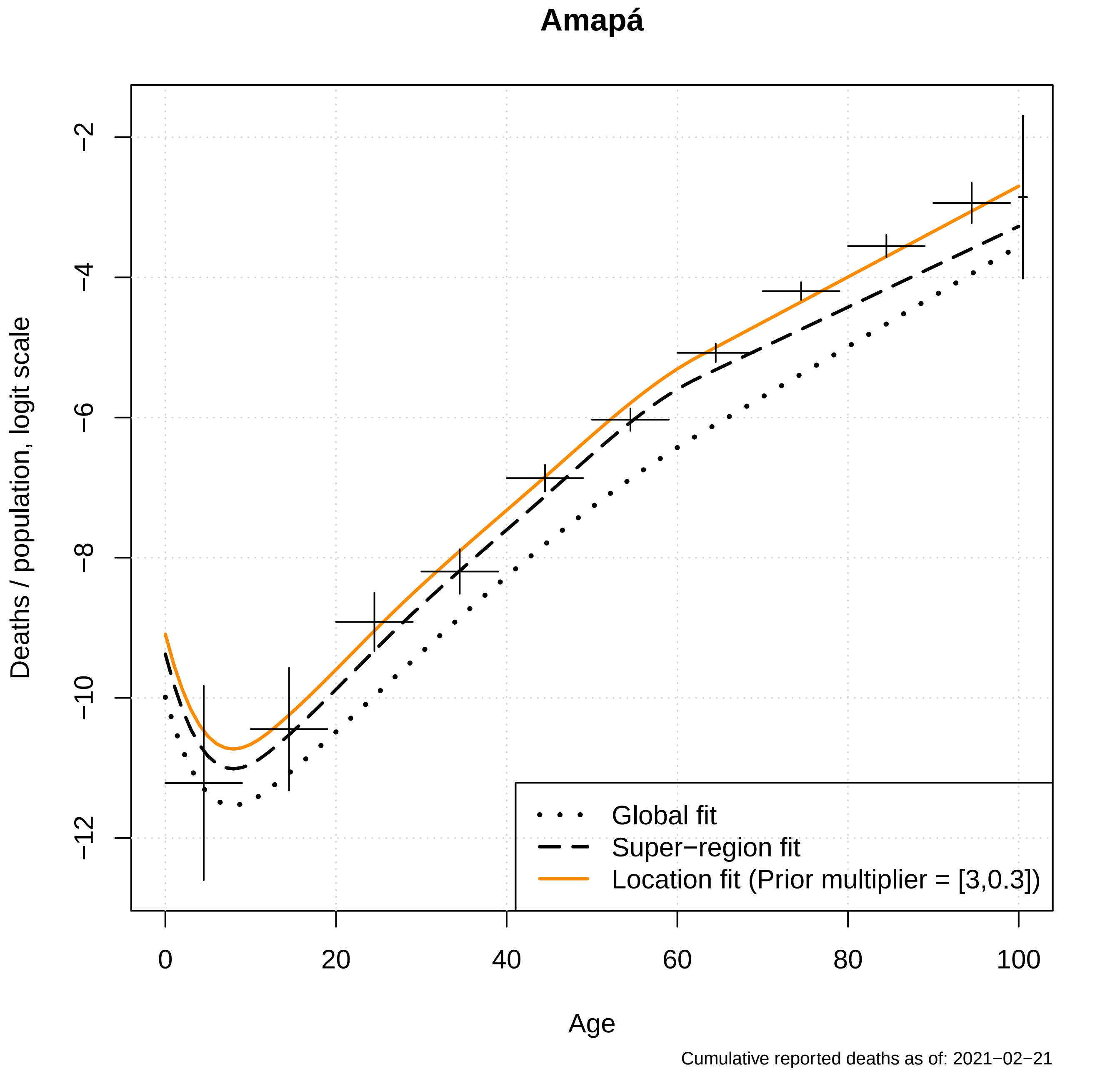

Amapá
Deaths / population, logit scale
−2
−4
−6
−8
−10
−12
0
20
40
60
80
100
Age
Global fit
Super−region fit
Location fit (Prior multiplier = [3,0.3])
Cumulative reported deaths as of: 2021−02−21

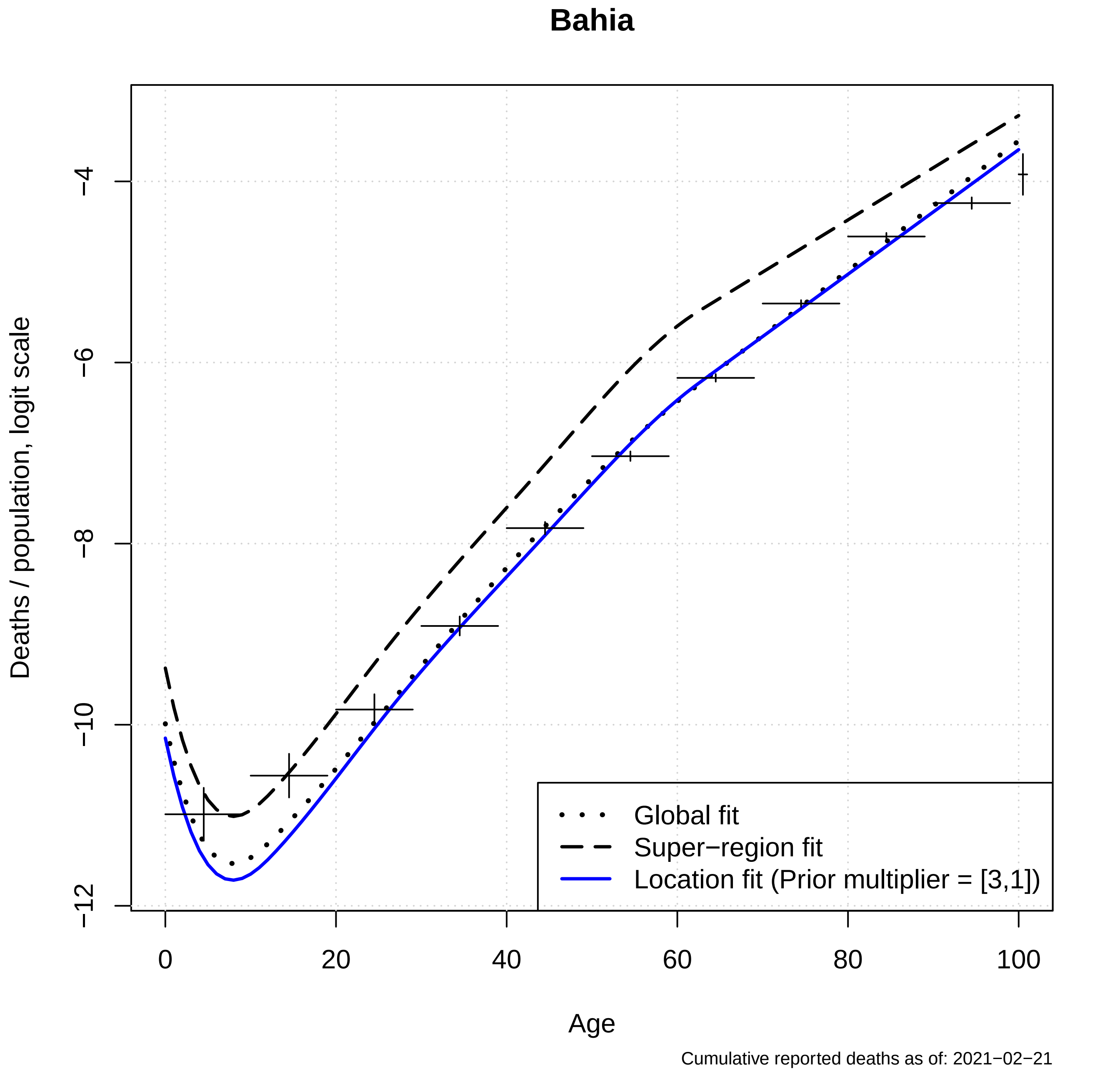
Bahia
Deaths / population, logit scale
−4
−6
−8
−10
−12
0
20
40
60
80
100
Age
Global fit
Super−region fit
Location fit (Prior multiplier = [3,1])
Cumulative reported deaths as of: 2021−02−21

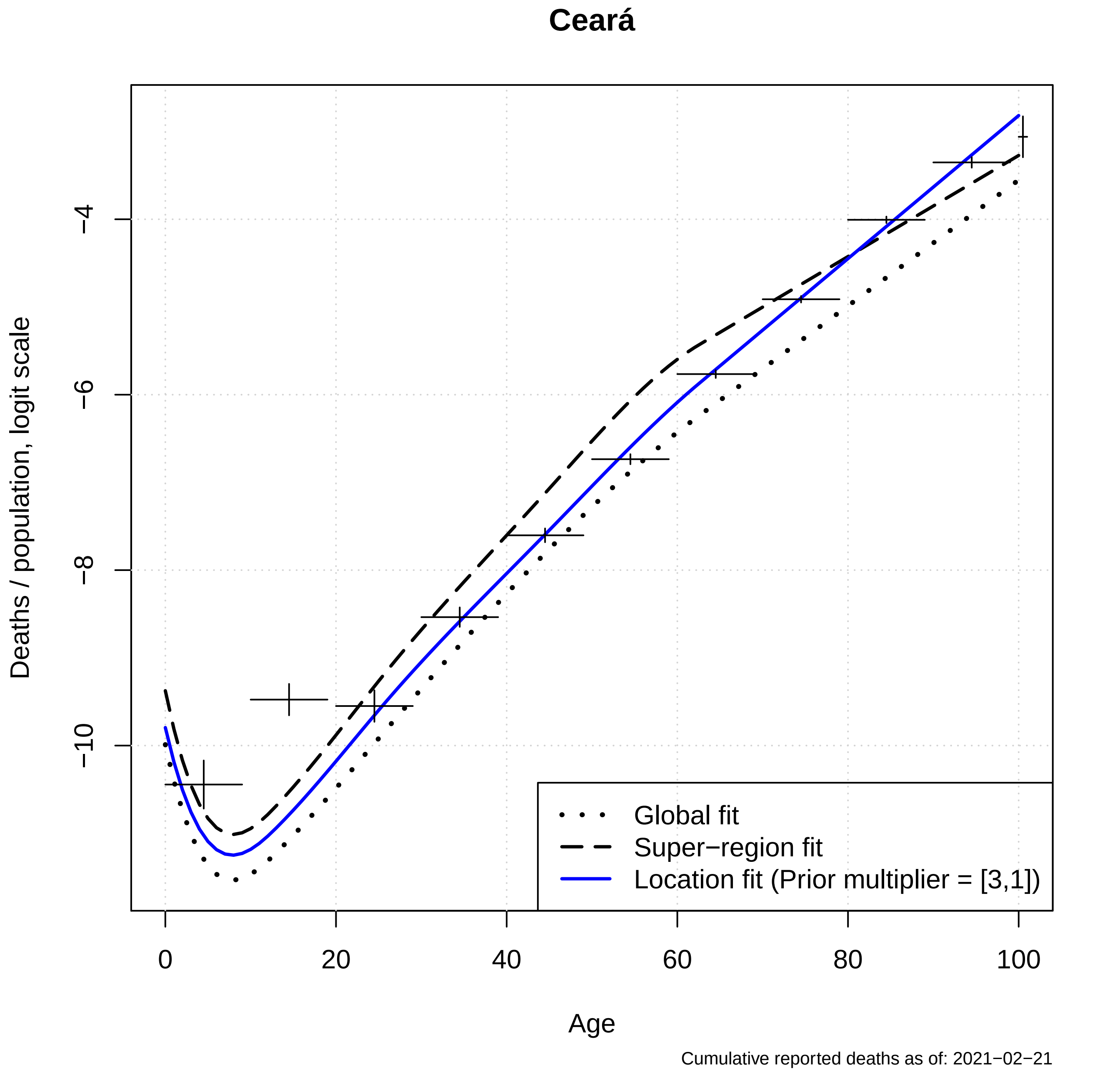

Ceará
Deaths / population, logit scale
−4
−6
−8
−10
0
20
40
60
80
100
Age
Global fit
Super−region fit
Location fit (Prior multiplier = [3,1])
Cumulative reported deaths as of: 2021−02−21

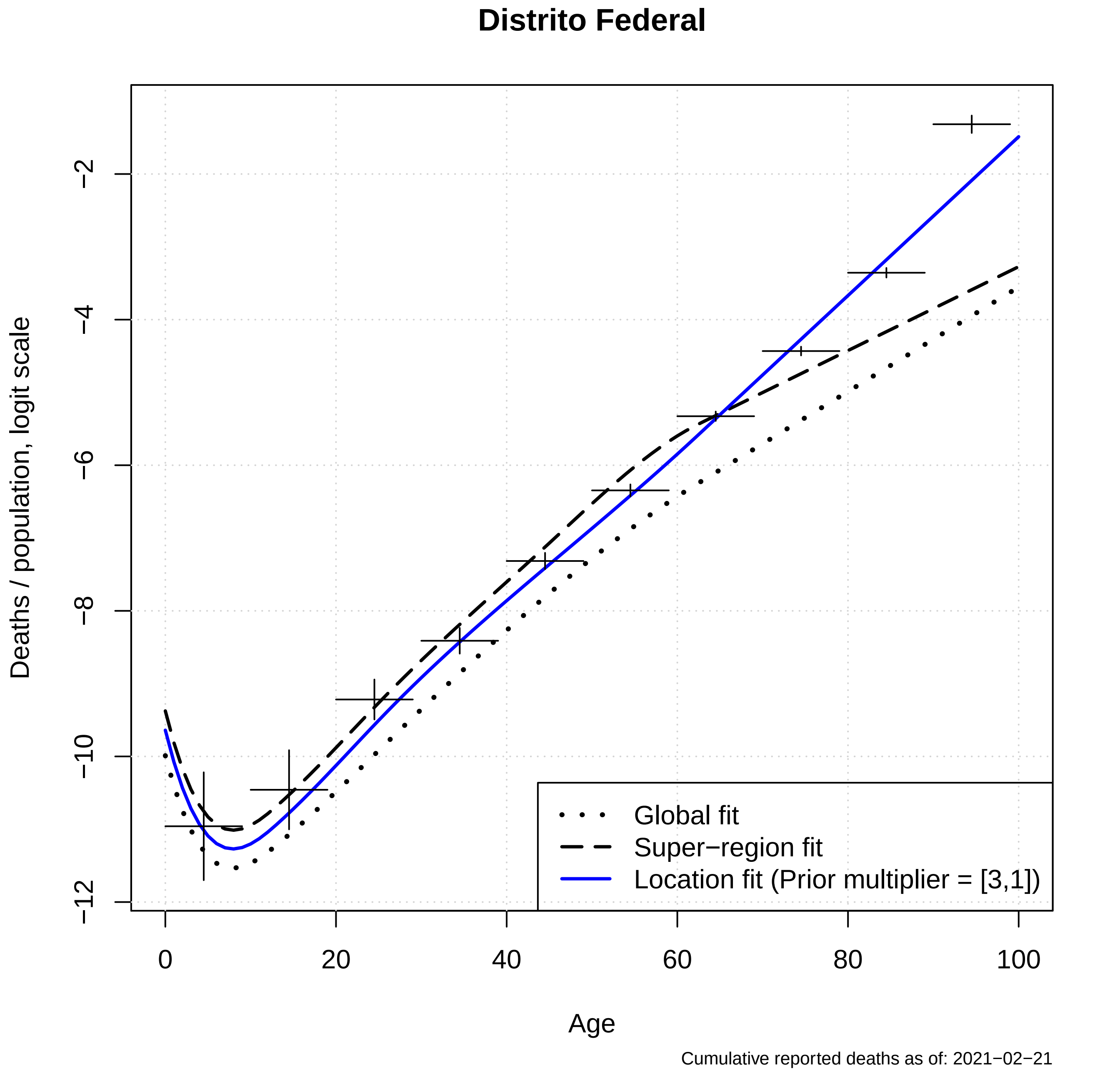
Distrito Federal
Deaths / population, logit scale
−2
−4
−6
−8
−10
−12
0
20
40
60
80
100
Age
Global fit
Super−region fit
Location fit (Prior multiplier = [3,1])
Cumulative reported deaths as of: 2021−02−21

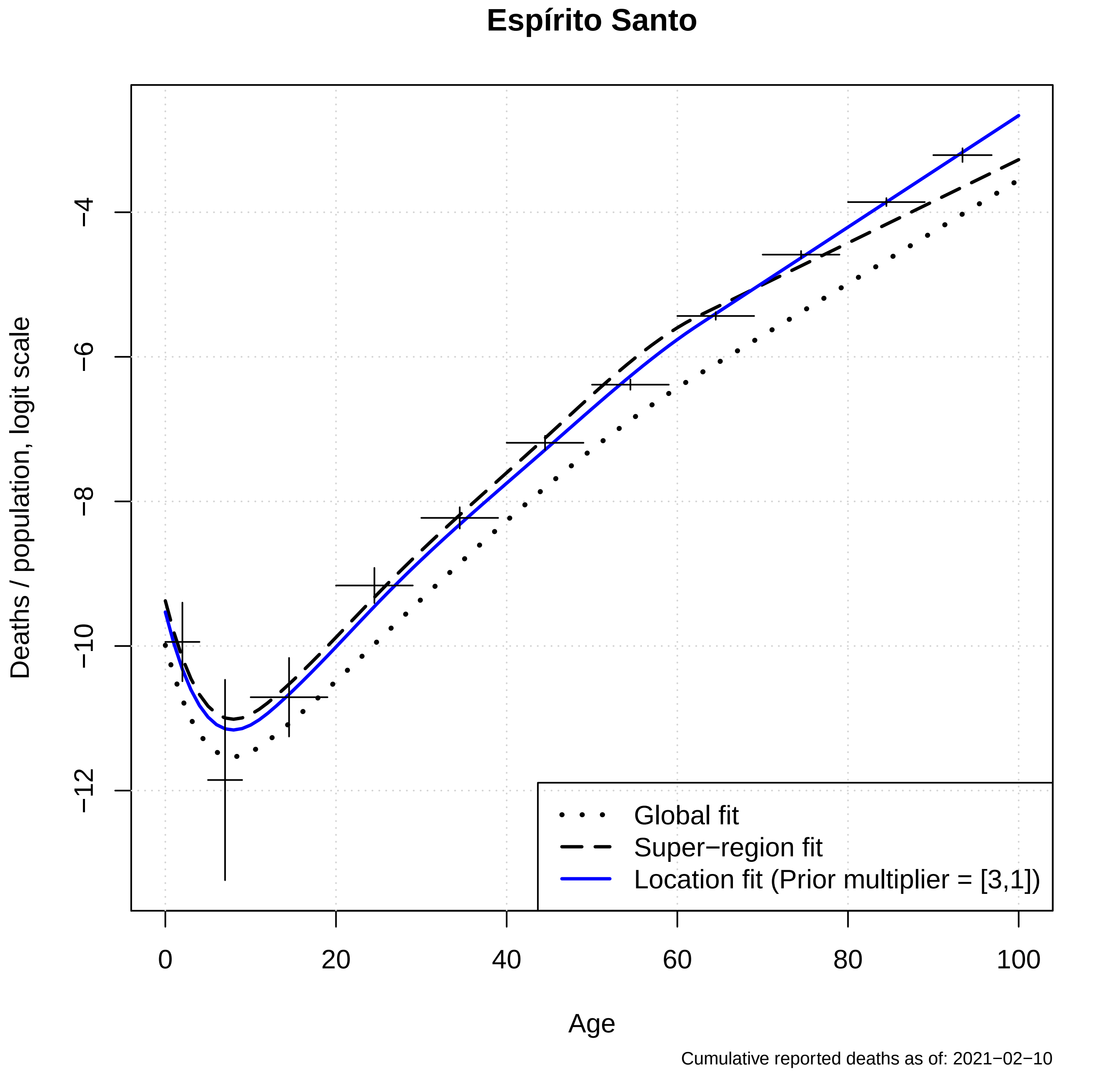

Espírito Santo
Deaths / population, logit scale
−4
−6
−8
−10
−12
0
20
40
60
80
100
Age
Global fit
Super−region fit
Location fit (Prior multiplier = [3,1])
Cumulative reported deaths as of: 2021−02−10

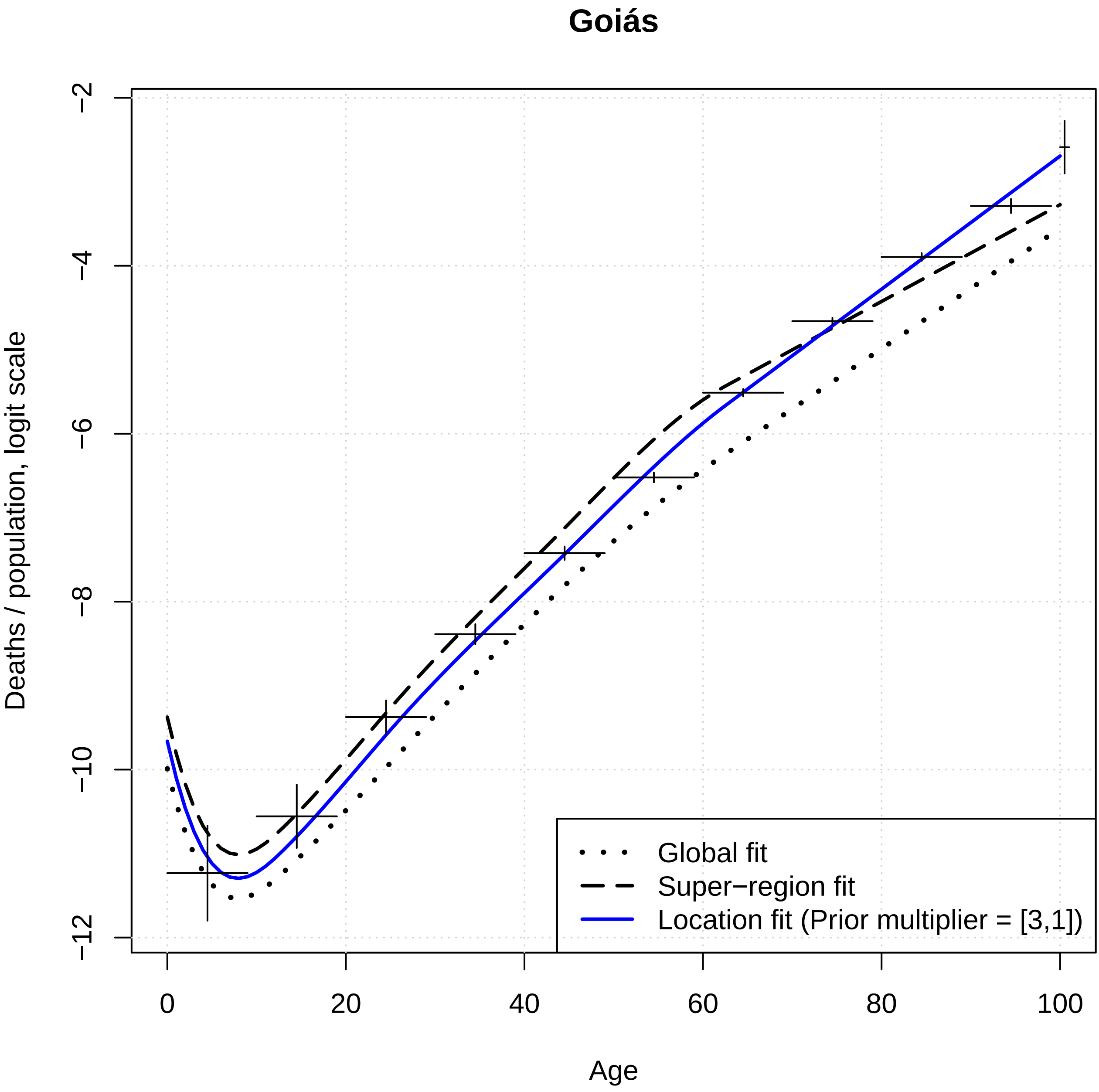

Cumulative reported deaths as of: 2021−02−21

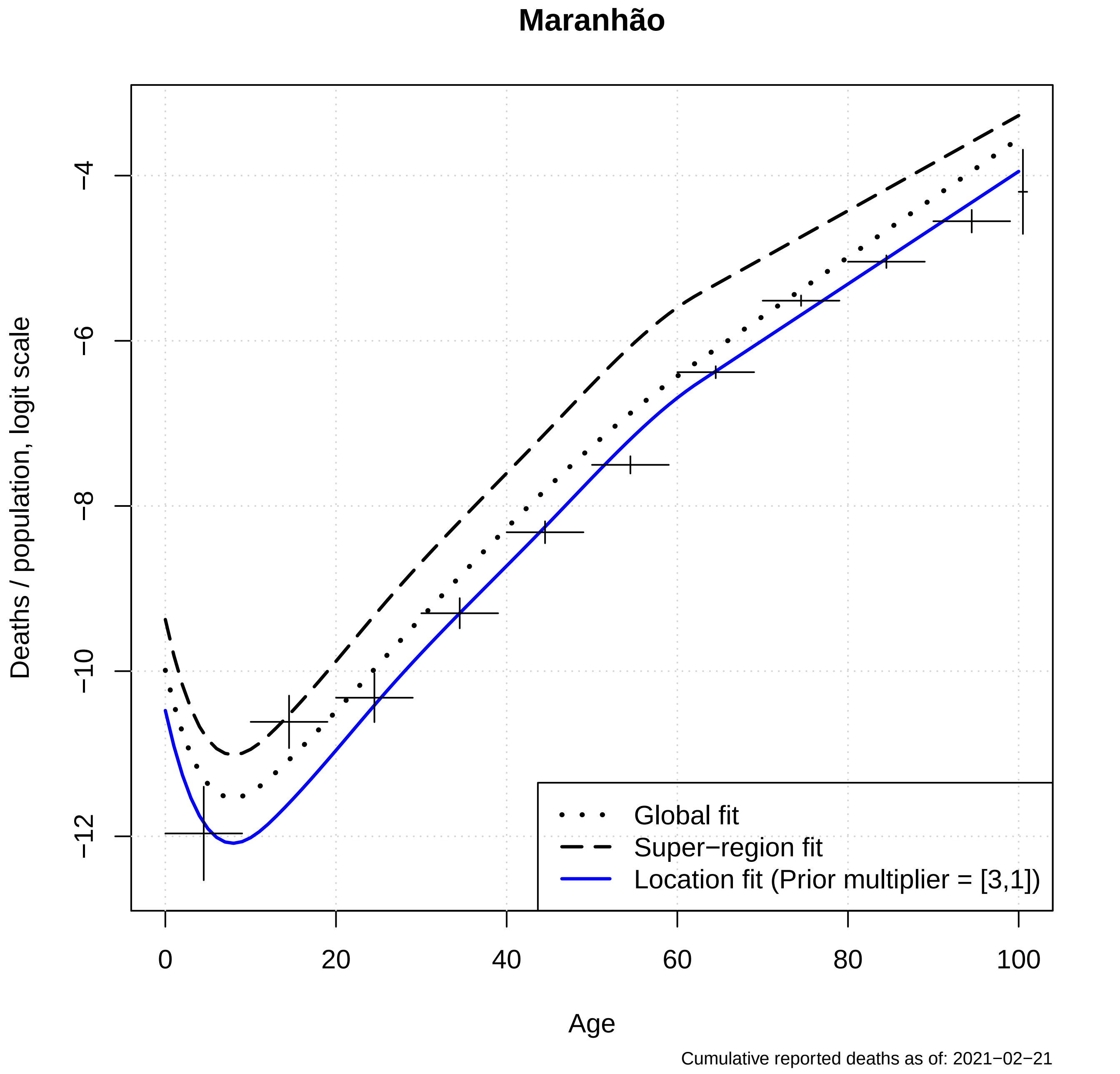

Maranhão
Deaths / population, logit scale
−4
−6
−8
−10
−12
0
20
40
60
80
100
Age
Global fit
Super−region fit
Location fit (Prior multiplier = [3,1])
Cumulative reported deaths as of: 2021−02−21

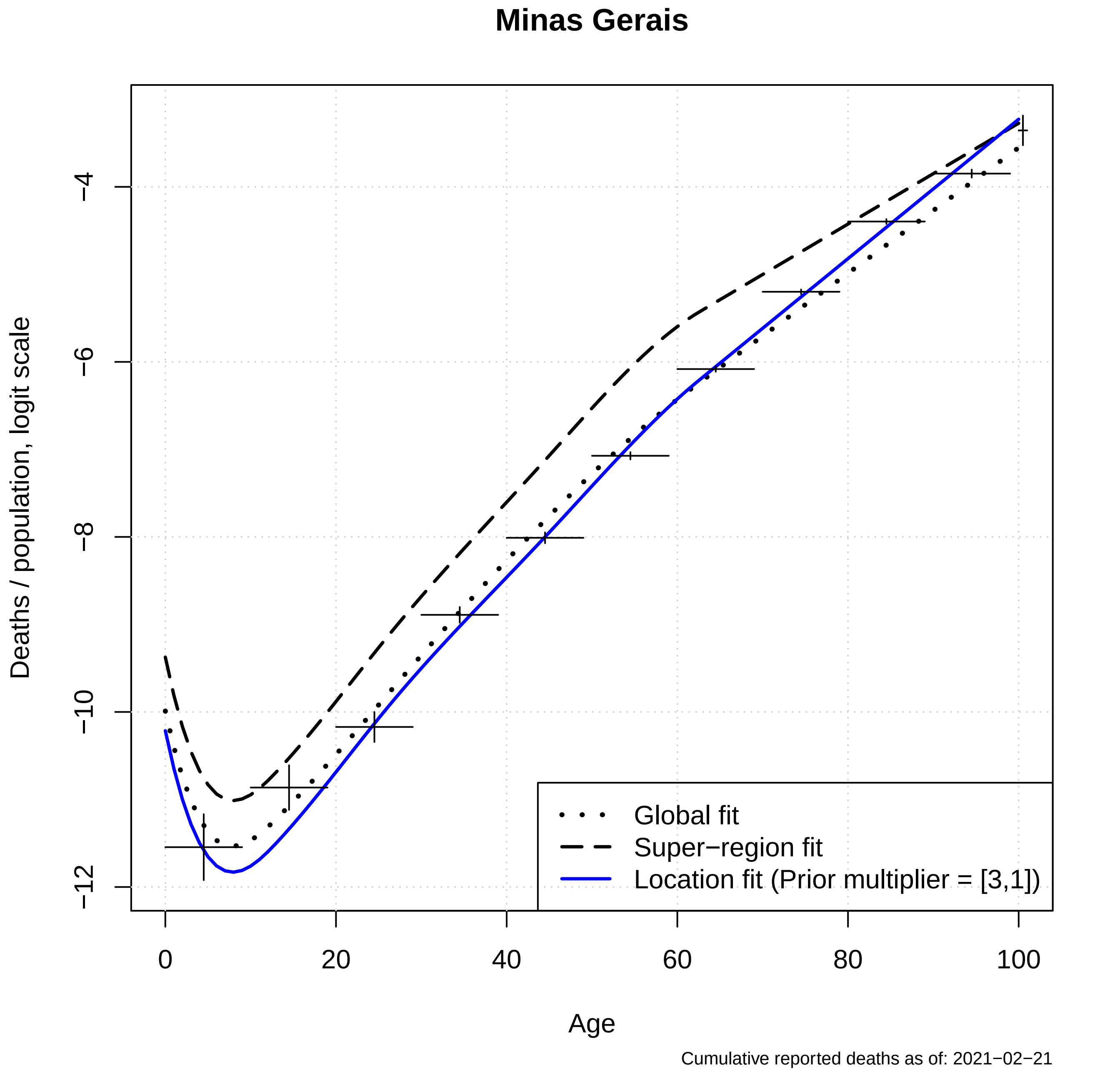

Minas Gerais
Deaths / population, logit scale
−4
−6
−8
−10
−12
0
20
40
60
80
100
Age
Global fit
Super−region fit
Location fit (Prior multiplier = [3,1])
Cumulative reported deaths as of: 2021−02−21

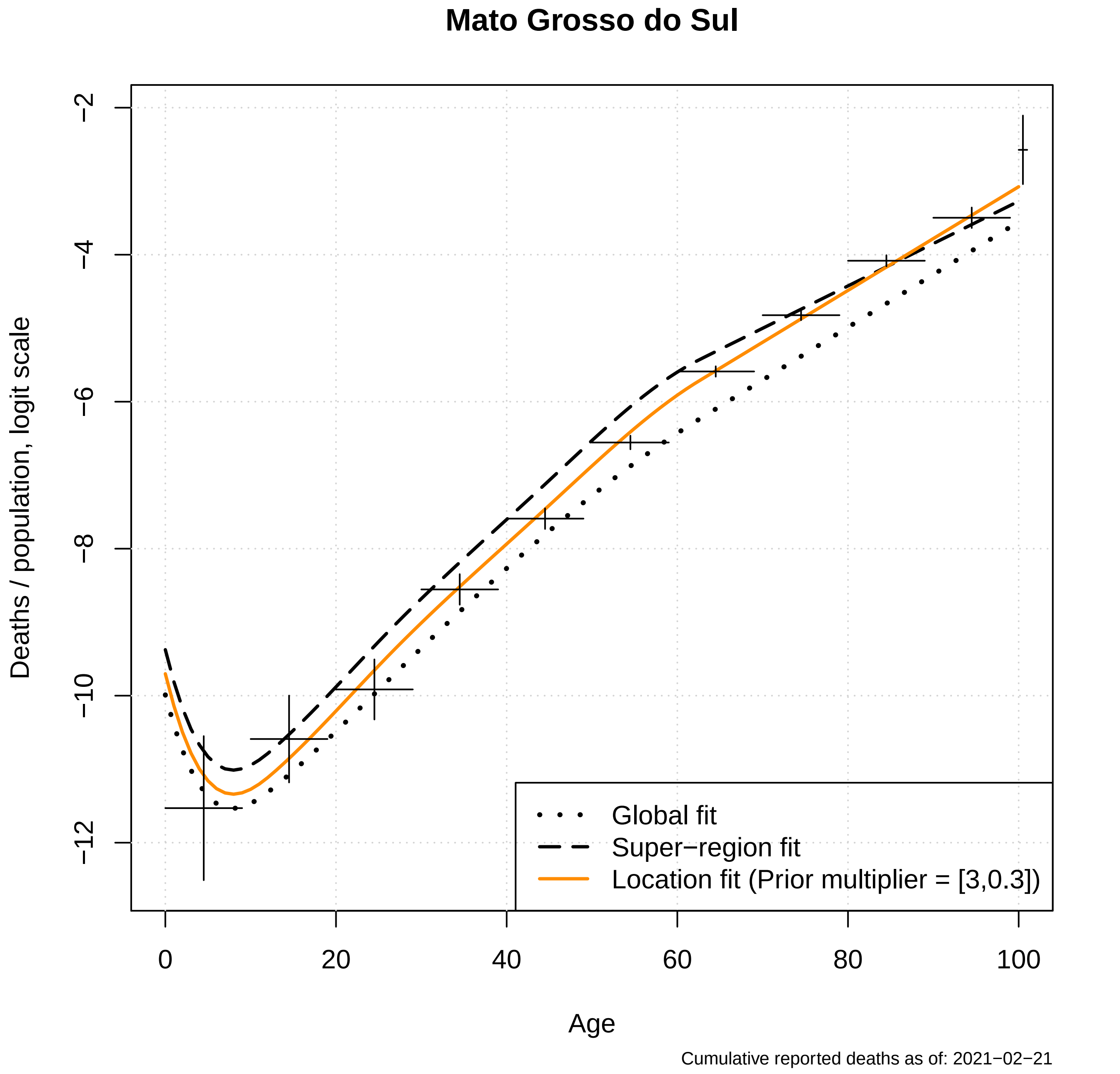

Mato Grosso do Sul
Deaths / population, logit scale
−2
−4
−6
−8
−10
−12
0
20
40
60
80
100
Age
Global fit
Super−region fit
Location fit (Prior multiplier = [3,0.3])
Cumulative reported deaths as of: 2021−02−21

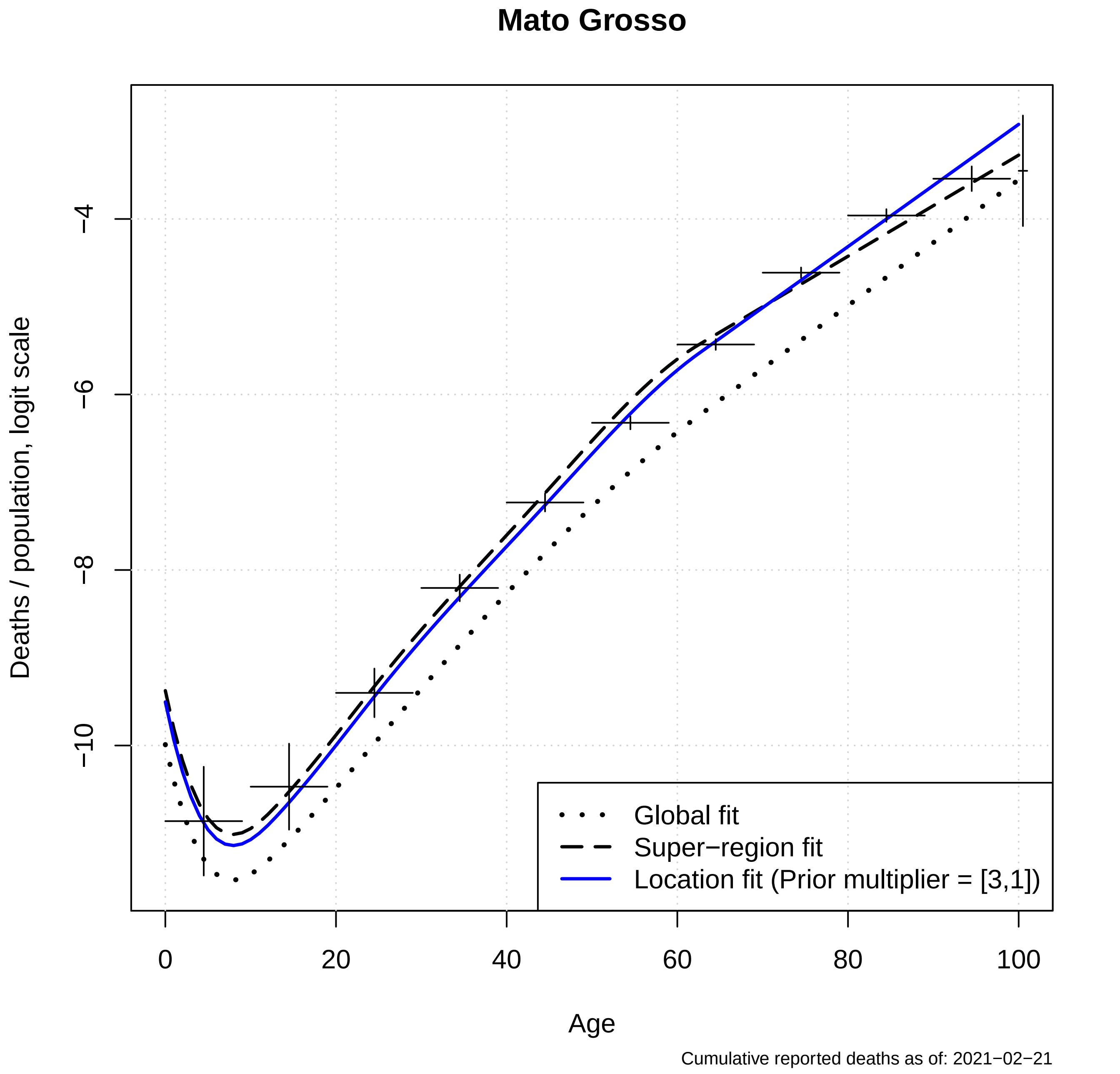
Mato Grosso
Deaths / population, logit scale
−4
−6
−8
−10
0
20
40
60
80
100
Age
Global fit
Super−region fit
Location fit (Prior multiplier = [3,1])
Cumulative reported deaths as of: 2021−02−21

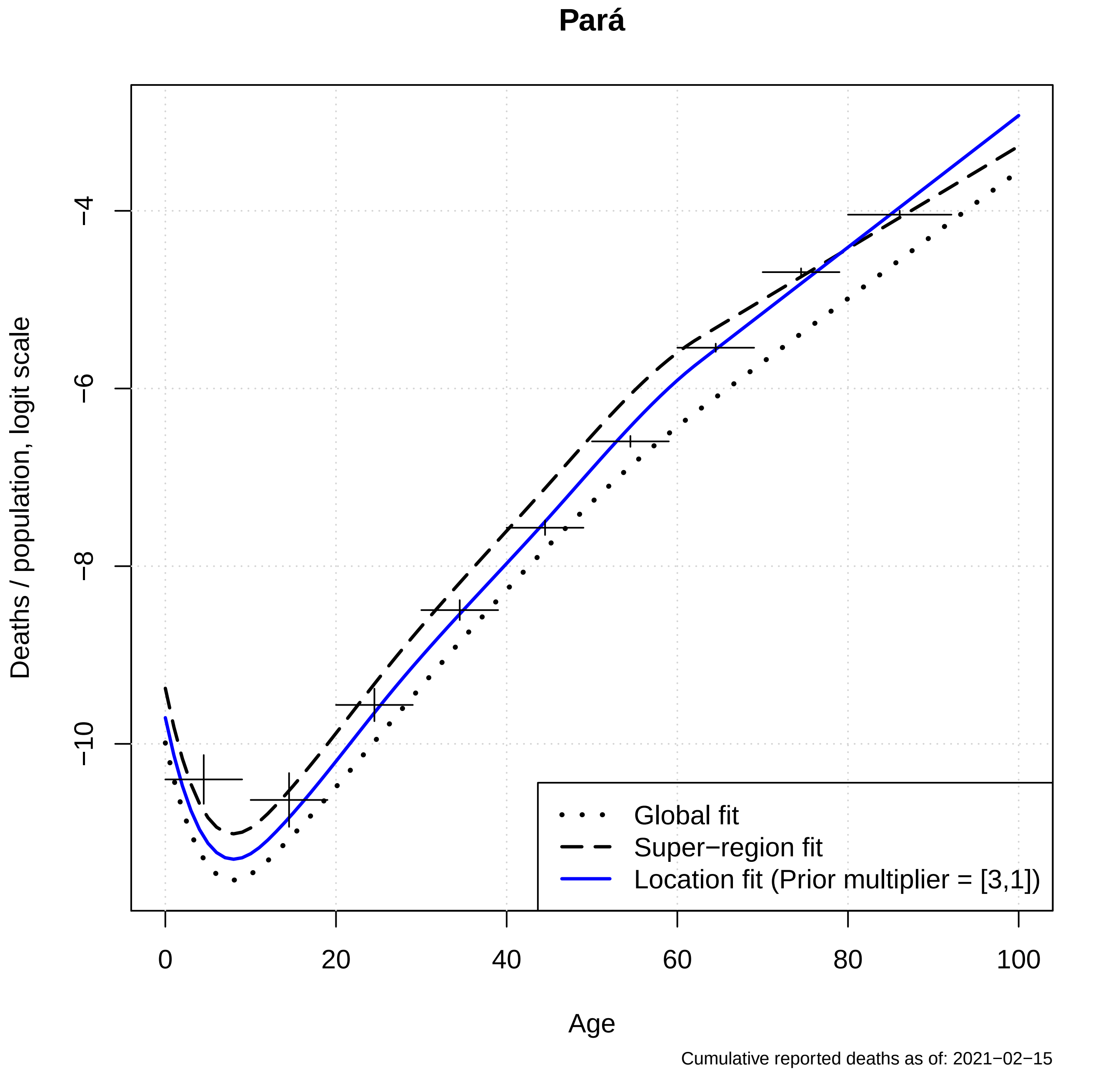

Pará
Deaths / population, logit scale
−4
−6
−8
−10
0
20
40
60
80
100
Age
Global fit
Super−region fit
Location fit (Prior multiplier = [3,1])
Cumulative reported deaths as of: 2021−02−15

Paraíba

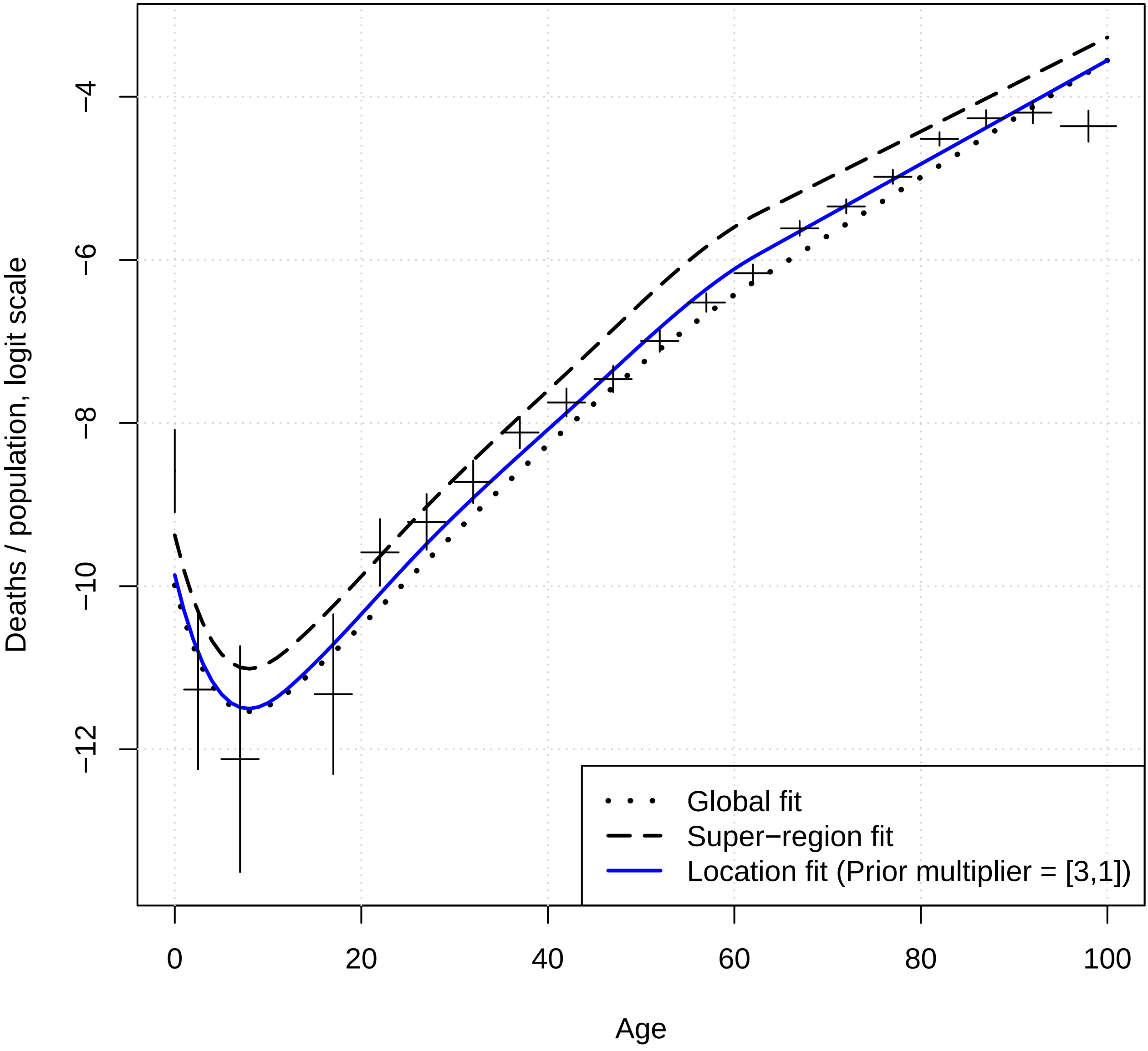

Cumulative reported deaths as of: 2021−02−10

Paraná

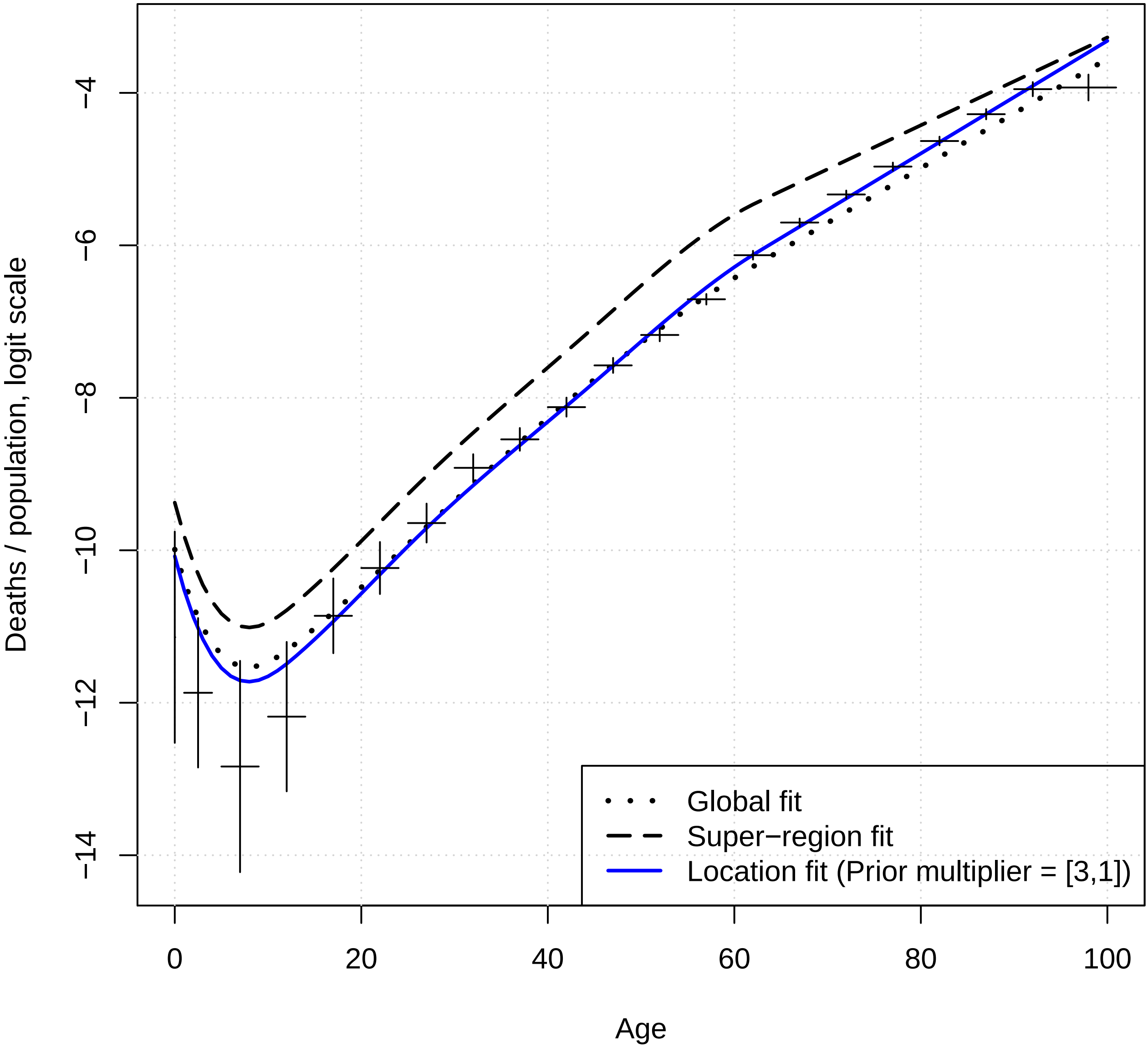

Cumulative reported deaths as of: 2021−02−09

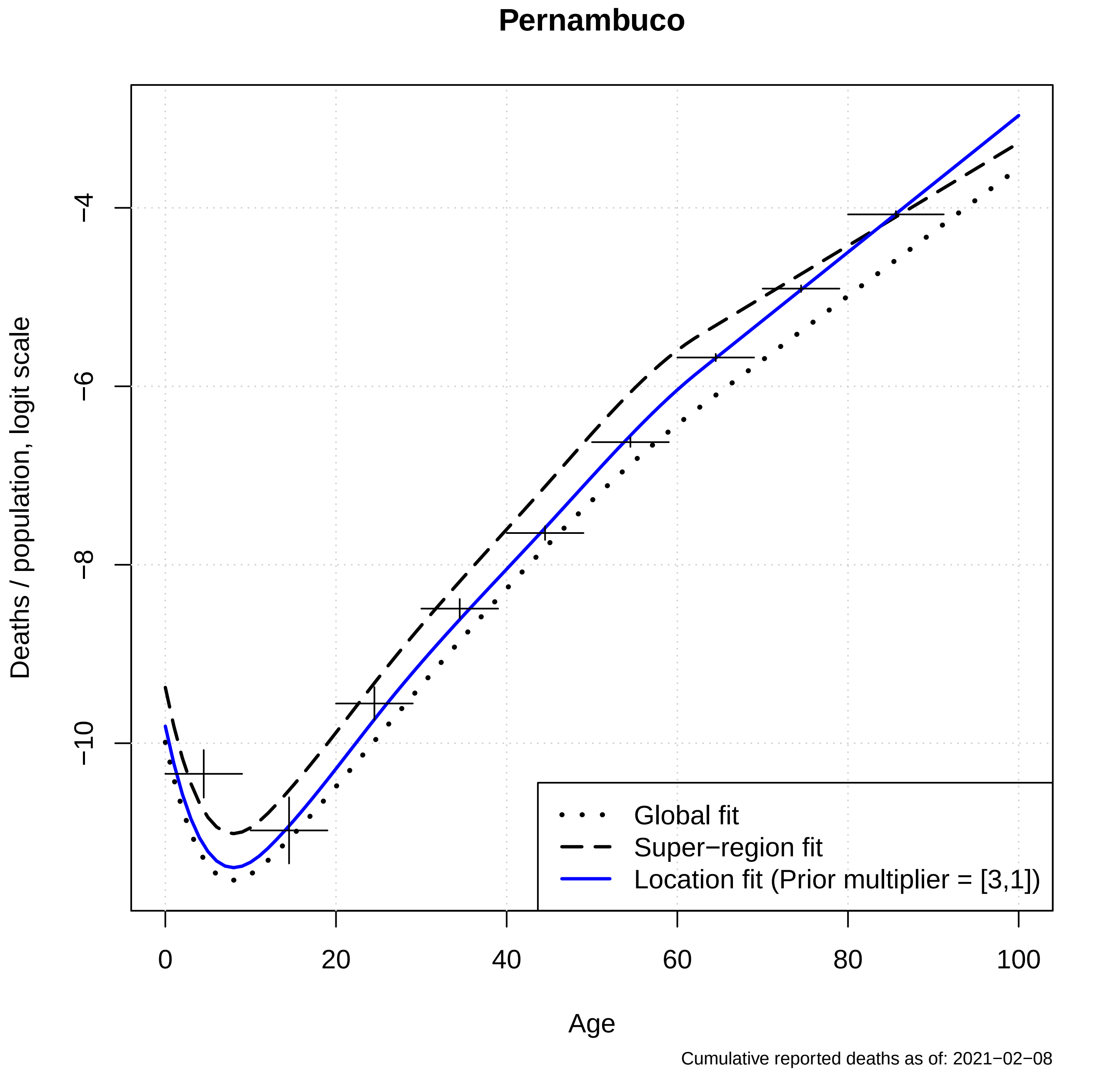

Pernambuco
Deaths / population, logit scale
−4
−6
−8
−10
0
20
40
60
80
100
Age
Global fit
Super−region fit
Location fit (Prior multiplier = [3,1])
Cumulative reported deaths as of: 2021−02−08

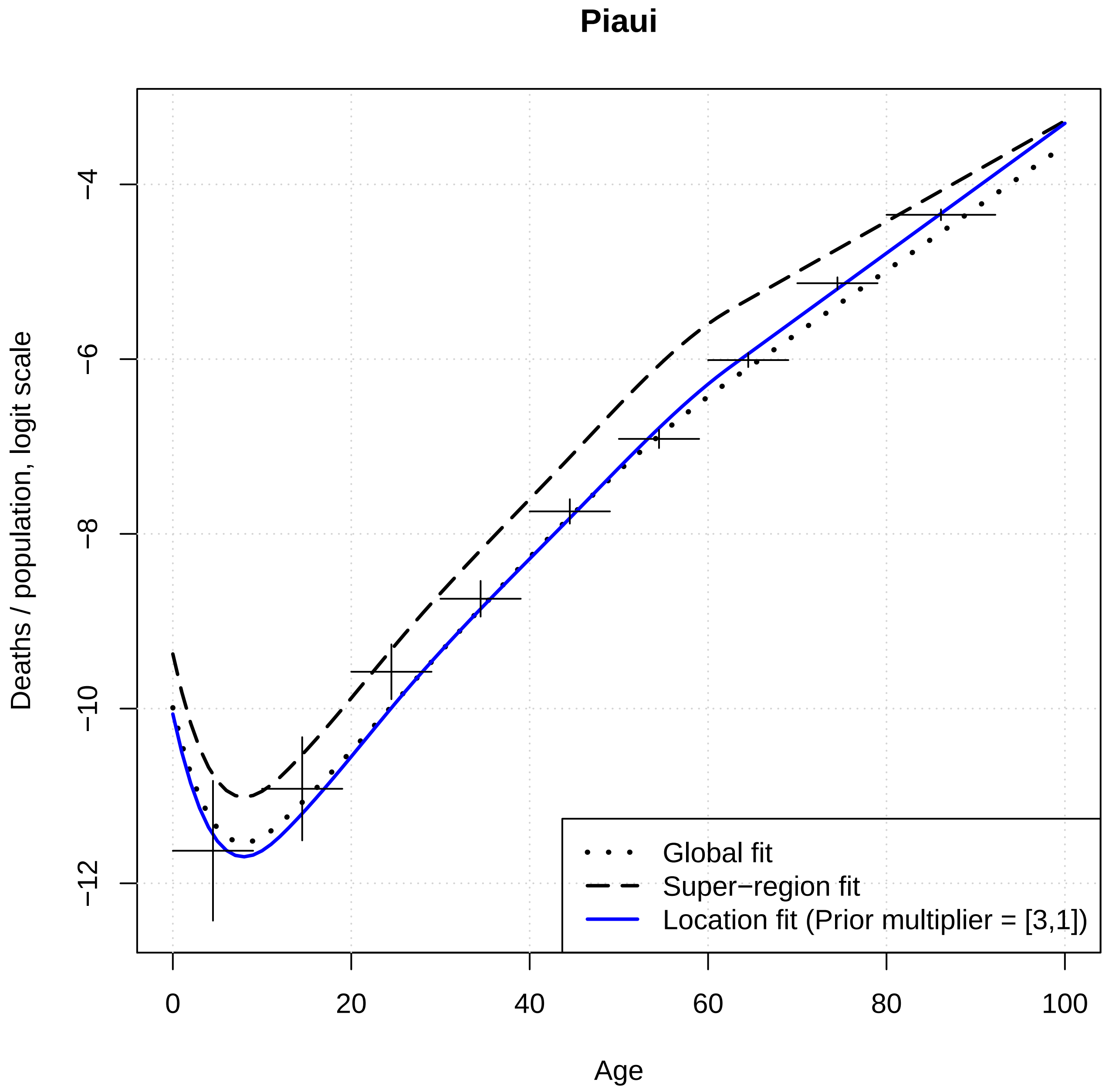

Cumulative reported deaths as of: 2021−02−10

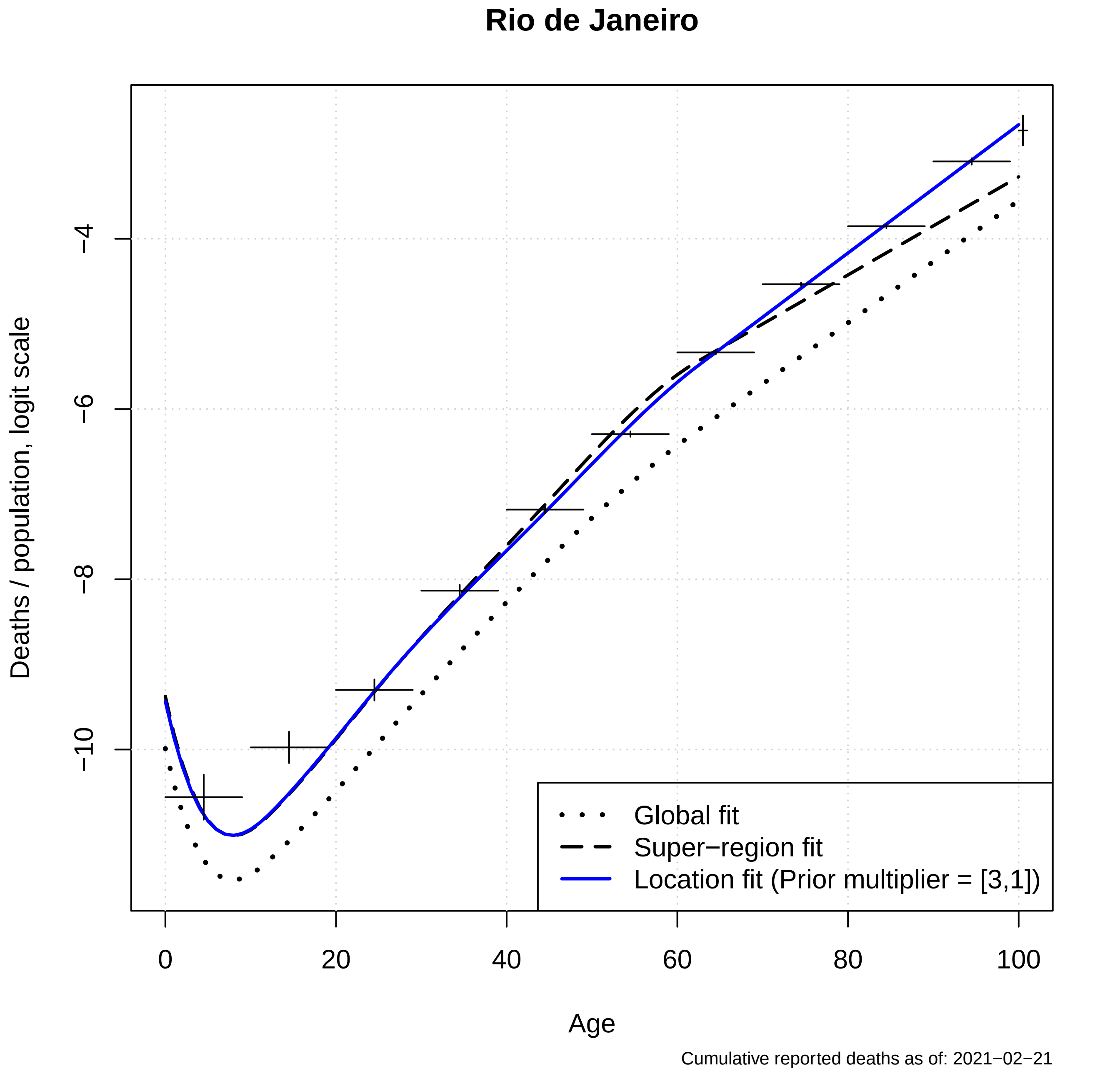

Rio de Janeiro
Deaths / population, logit scale
−4
−6
−8
−10
0
20
40
60
80
100
Age
Global fit
Super−region fit
Location fit (Prior multiplier = [3,1])
Cumulative reported deaths as of: 2021−02−21

Rio Grande do Norte

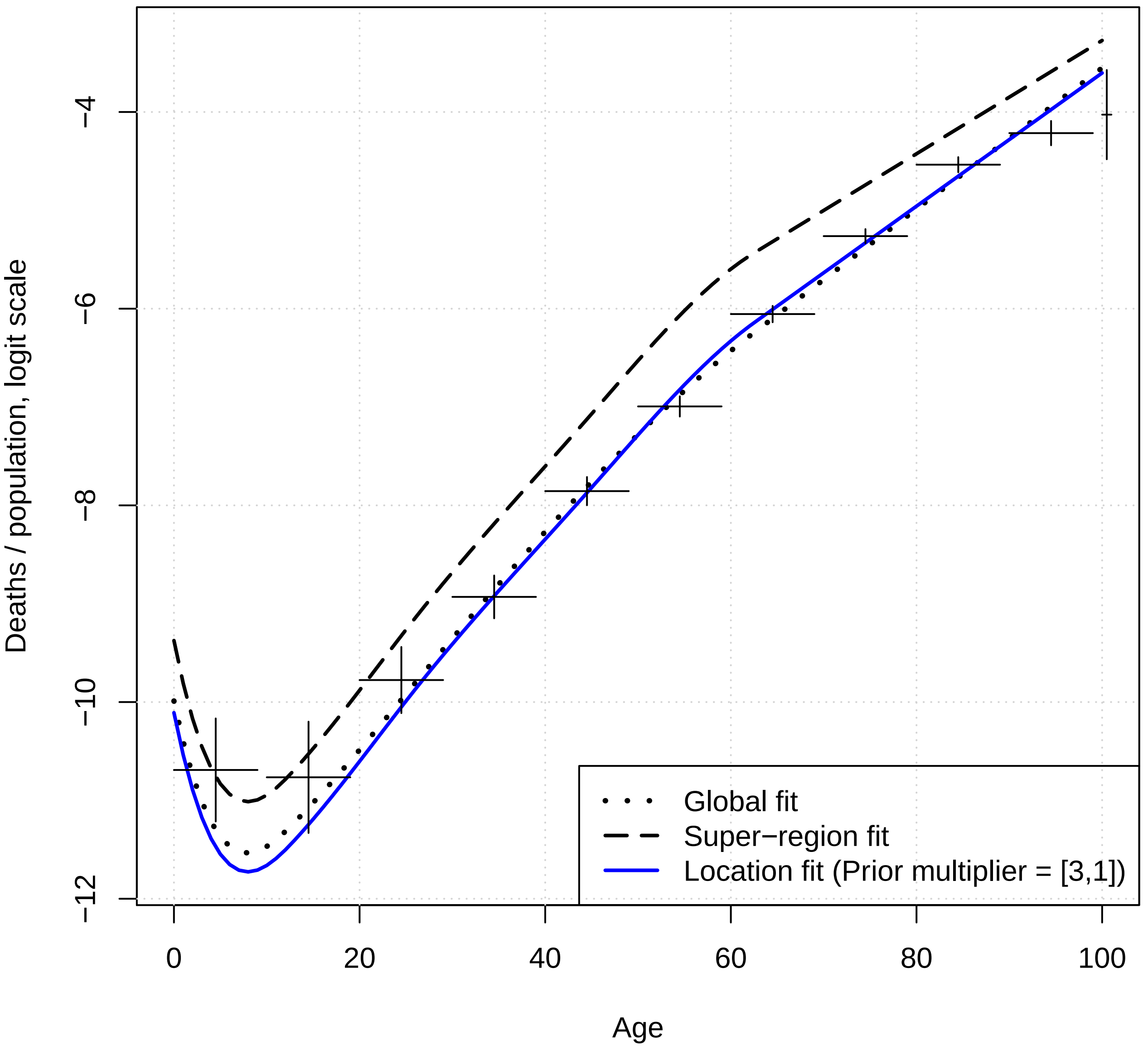

Cumulative reported deaths as of: 2021−02−21

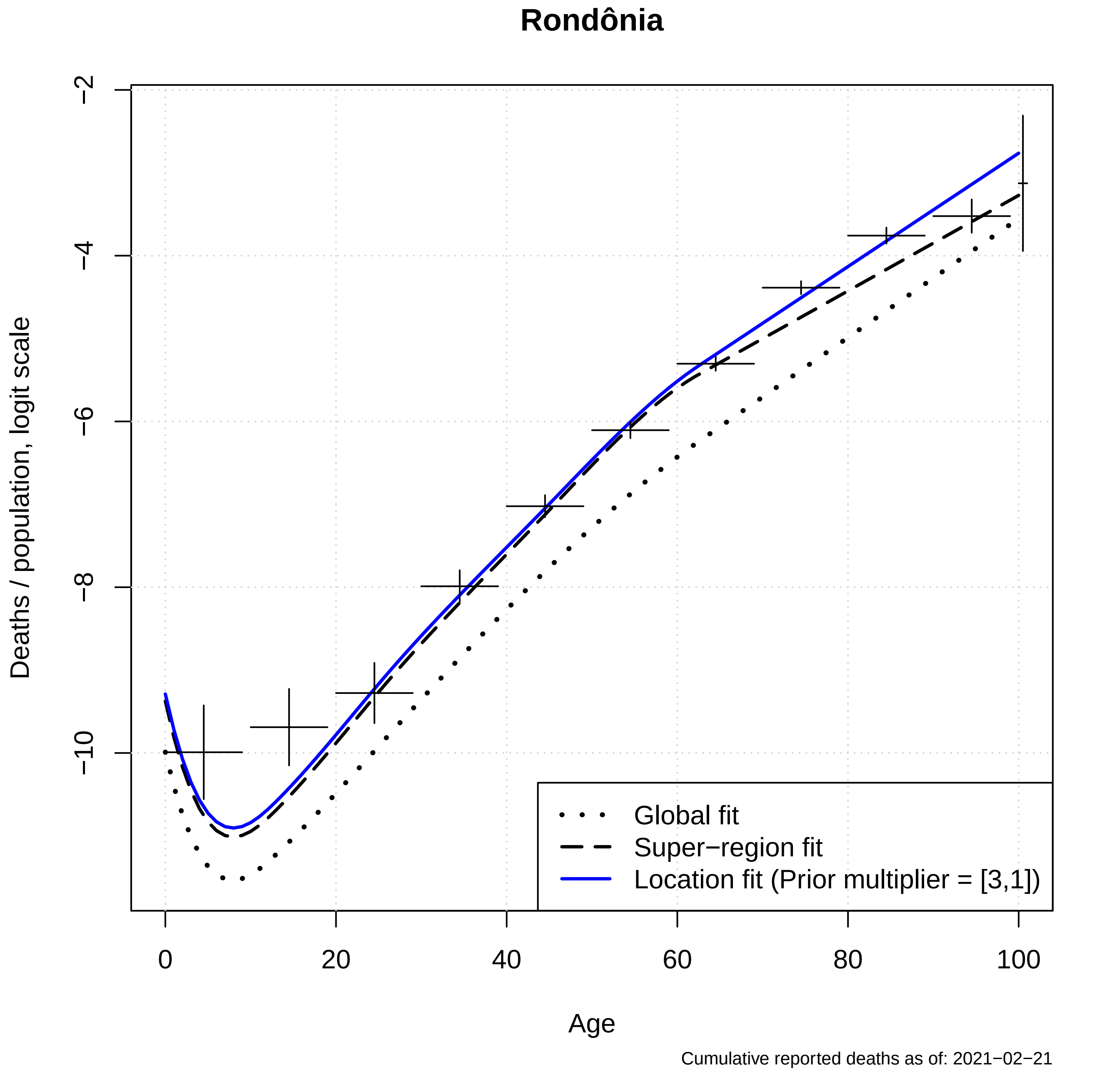

Rondônia
Deaths / population, logit scale
−2
−4
−6
−8
−10
0
20
40
60
80
100
Age
Global fit
Super−region fit
Location fit (Prior multiplier = [3,1])
Cumulative reported deaths as of: 2021−02−21

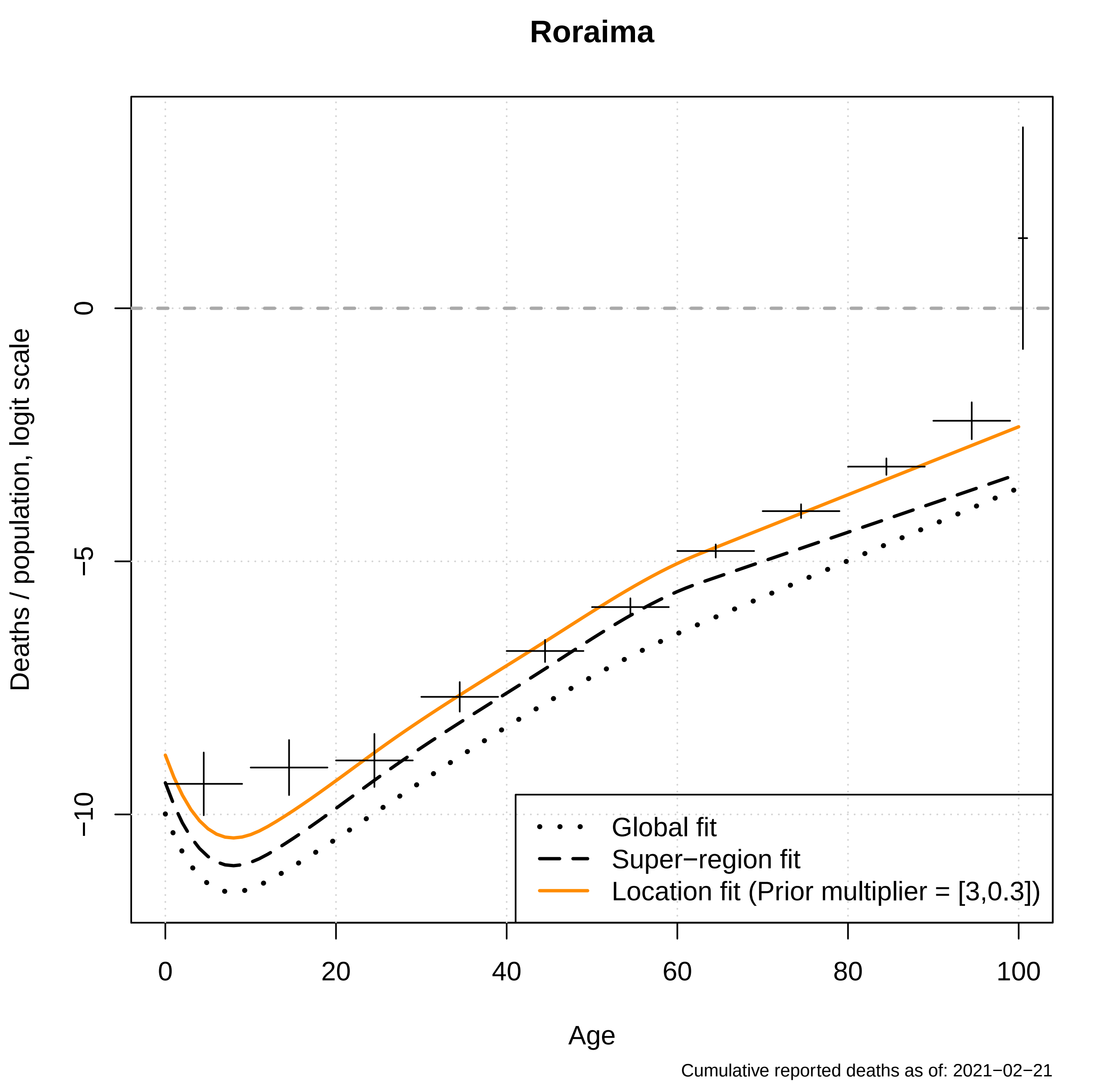

Roraima
Deaths / population, logit scale
0
−5
−10
0
20
40
60
80
100
Age
Global fit
Super−region fit
Location fit (Prior multiplier = [3,0.3])
Cumulative reported deaths as of: 2021−02−21

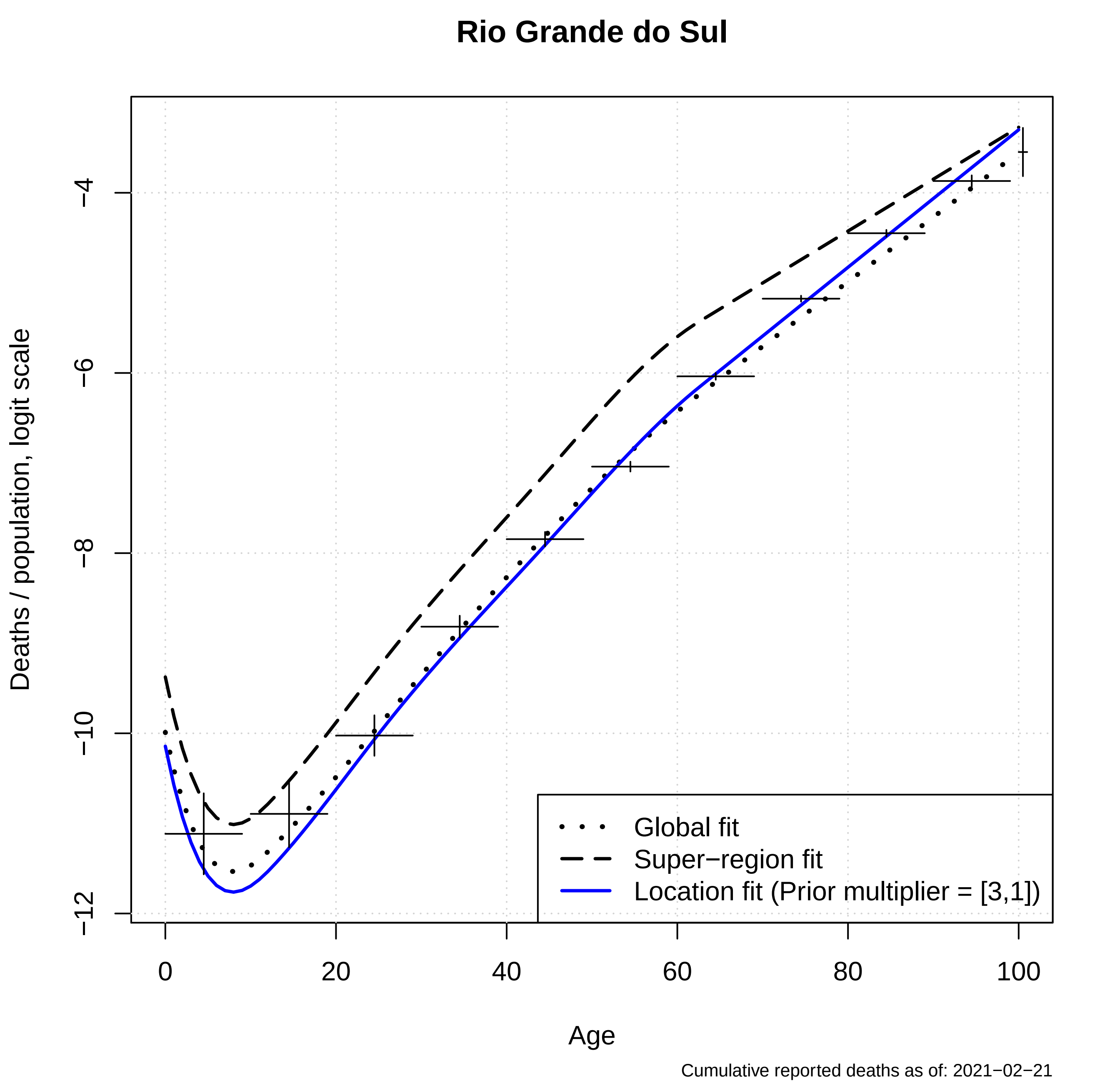

Rio Grande do Sul
Deaths / population, logit scale
−4
−6
−8
−10
−12
0
20
40
60
80
100
Age
Global fit
Super−region fit
Location fit (Prior multiplier = [3,1])
Cumulative reported deaths as of: 2021−02−21

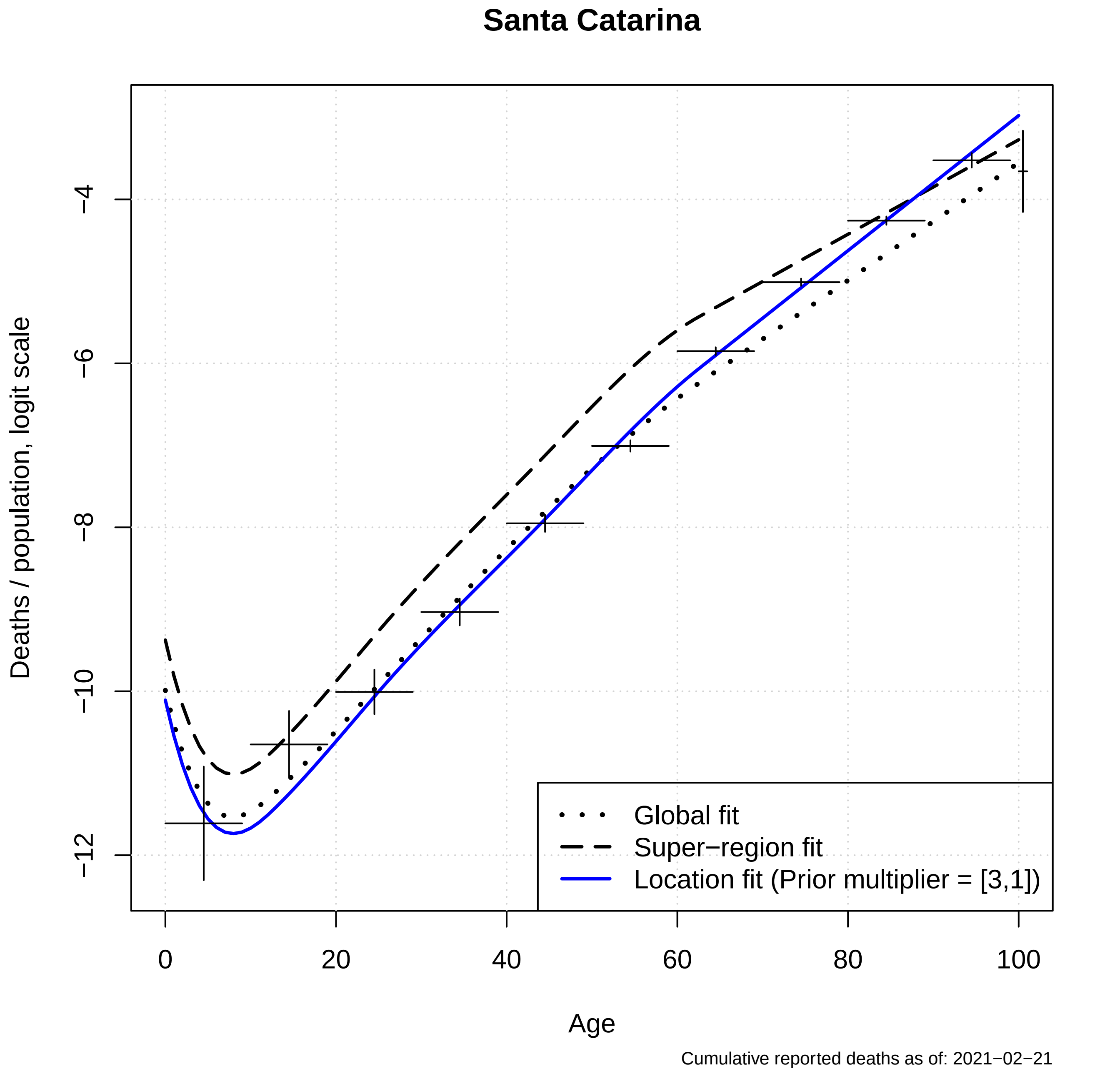

Santa Catarina
Deaths / population, logit scale
−4
−6
−8
−10
−12
0
20
40
60
80
100
Age
Global fit
Super−region fit
Location fit (Prior multiplier = [3,1])
Cumulative reported deaths as of: 2021−02−21

Sergipe

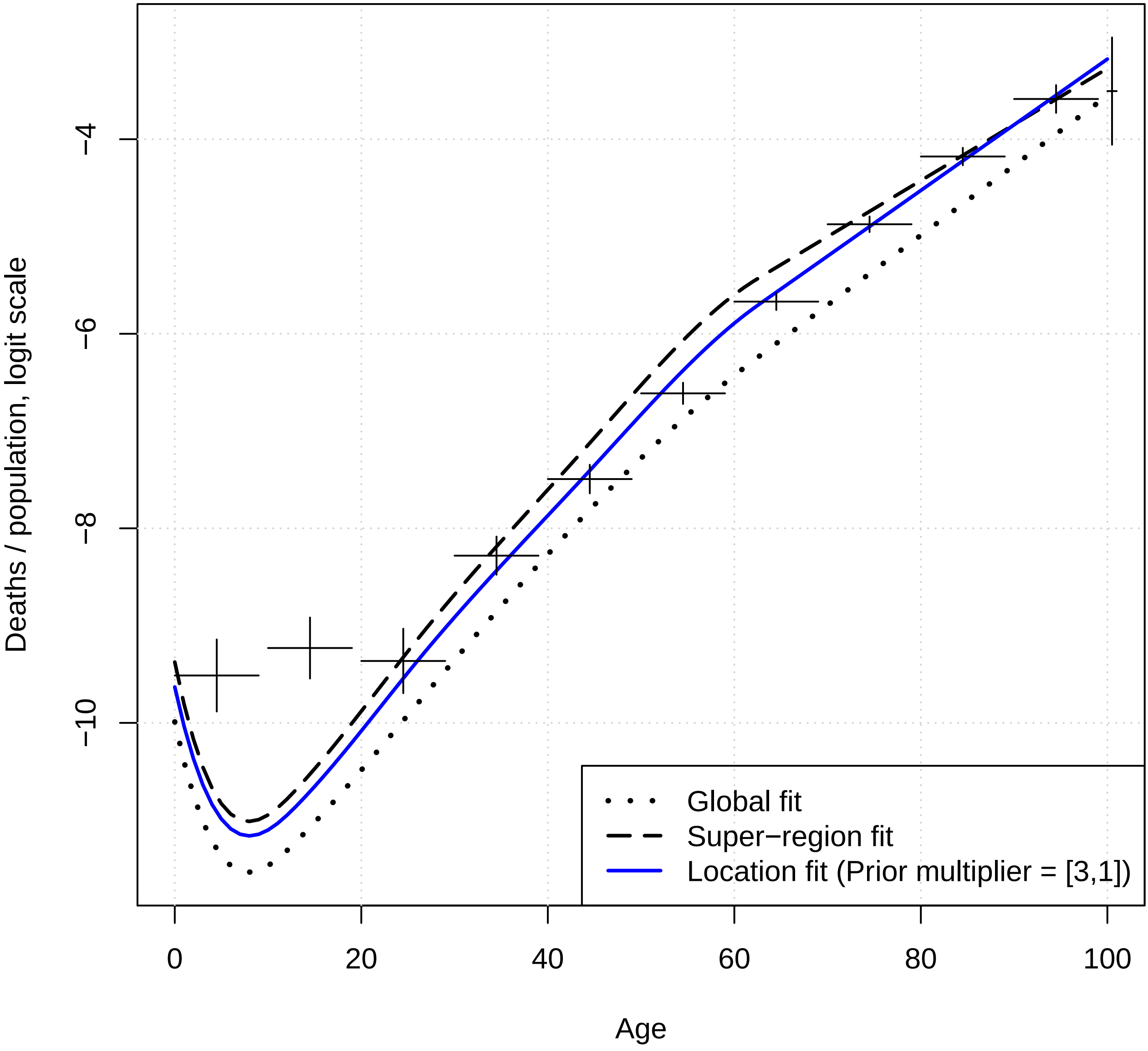

Cumulative reported deaths as of: 2021−02−21

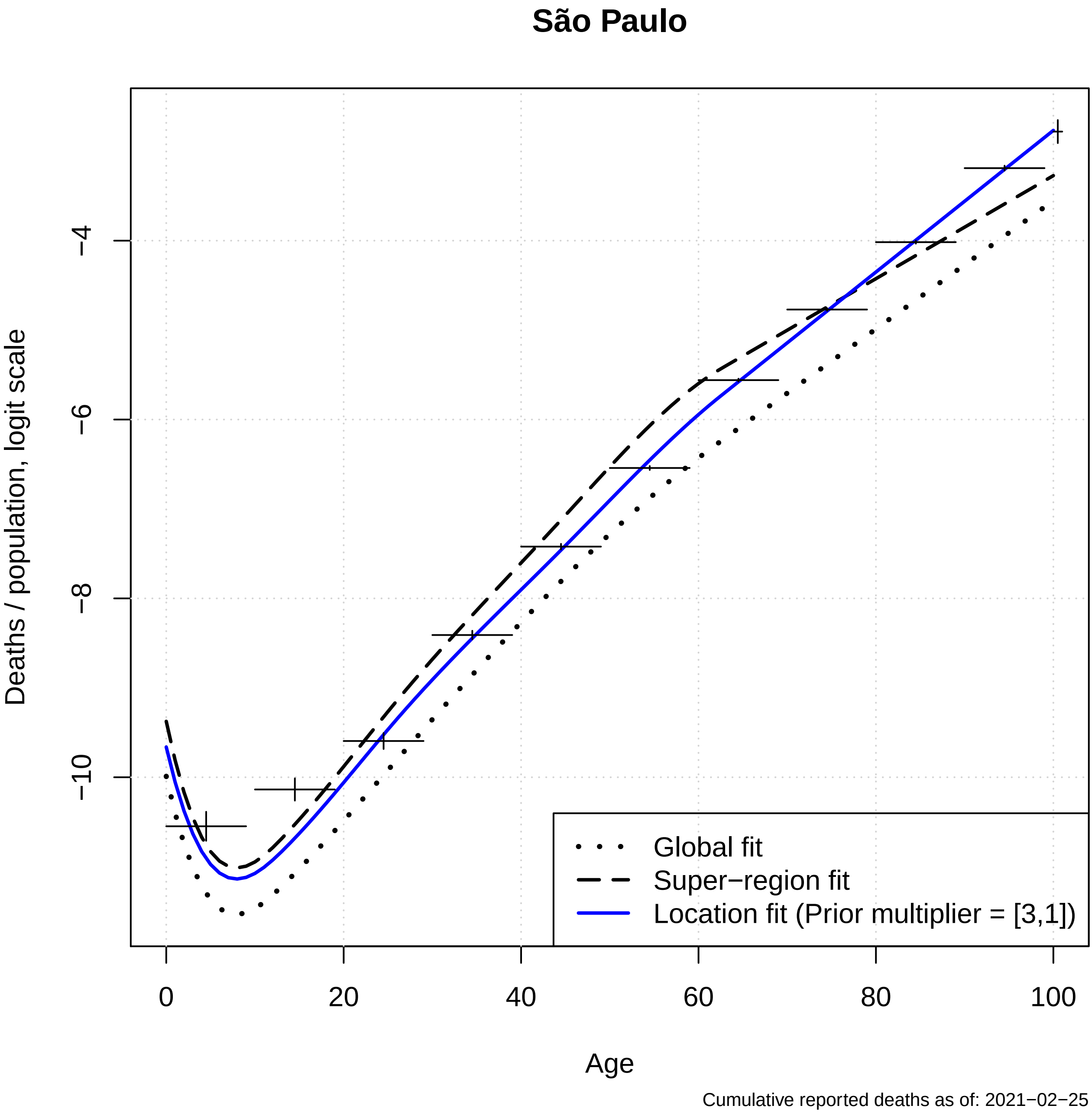
São Paulo
Deaths / population, logit scale
−4
−6
−8
−10
0
20
40
60
80
100
Age
Global fit
Super−region fit
Location fit (Prior multiplier = [3,1])
Cumulative reported deaths as of: 2021−02−25

Figure S8G. India subnationals

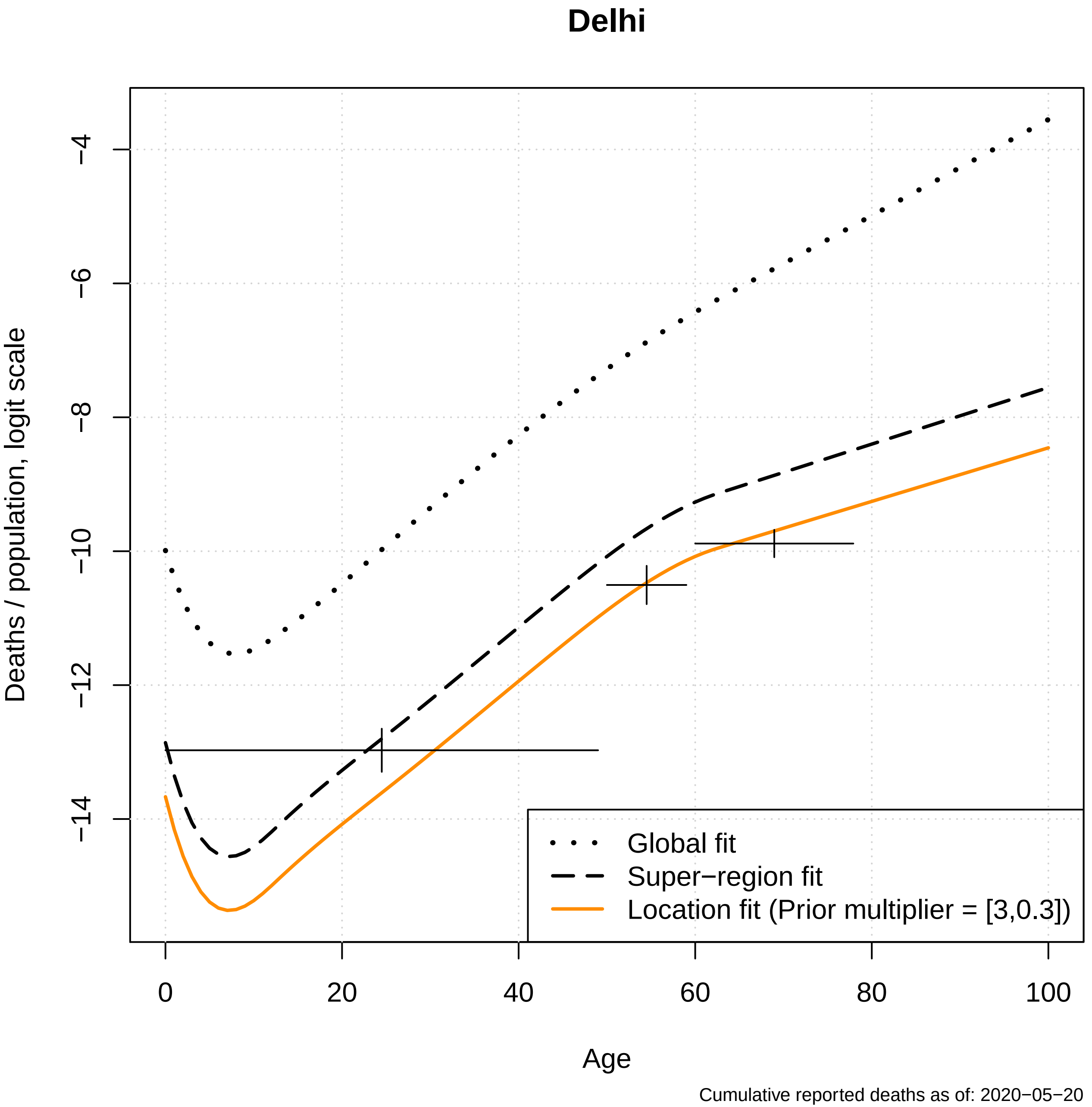

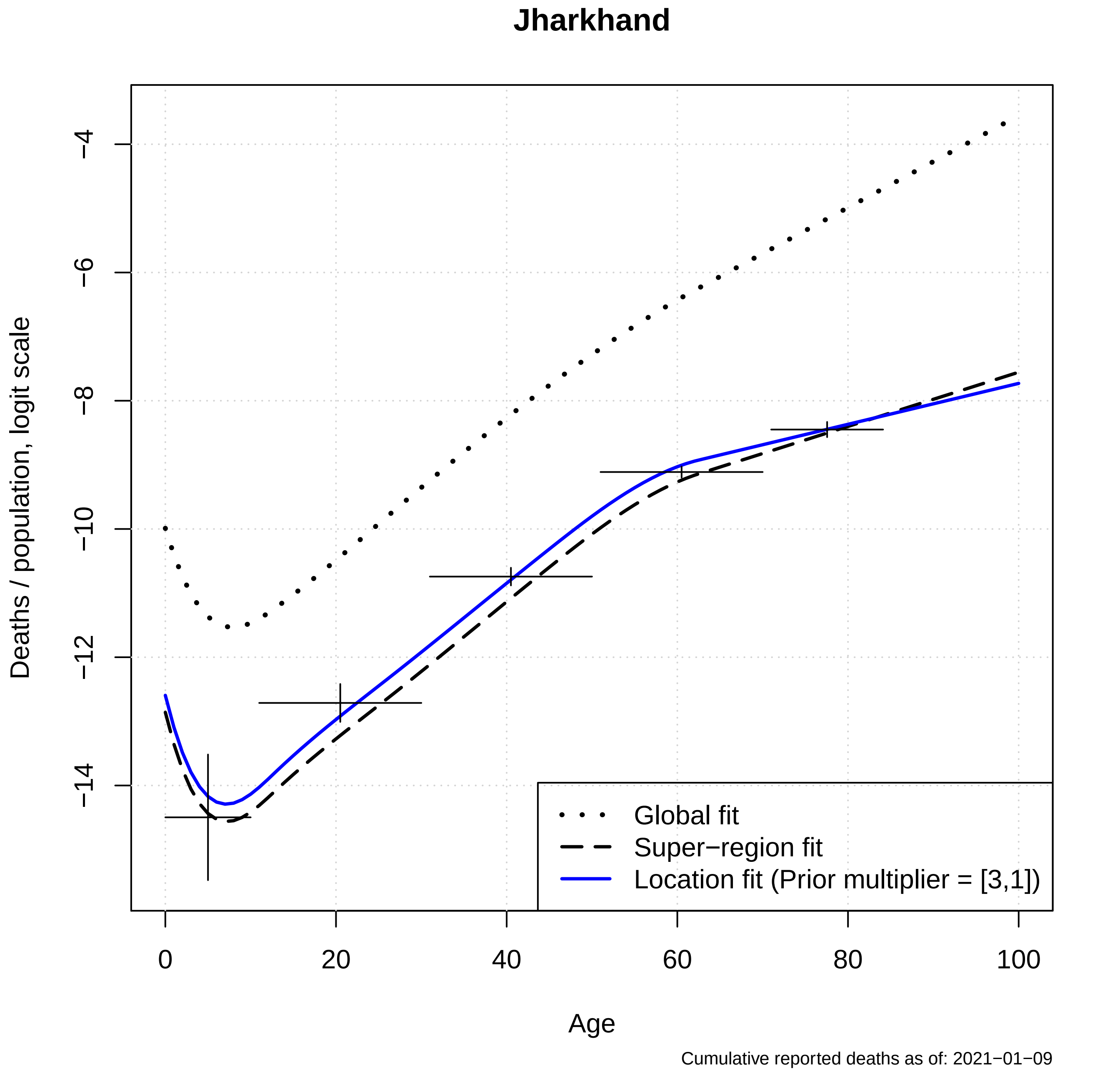

Jharkhand
Deaths / population, logit scale
−4
−6
−8
−10
−12
−14
0
20
40
60
80
100
Age
Global fit
Super−region fit
Location fit (Prior multiplier = [3,1])
Cumulative reported deaths as of: 2021−01−09

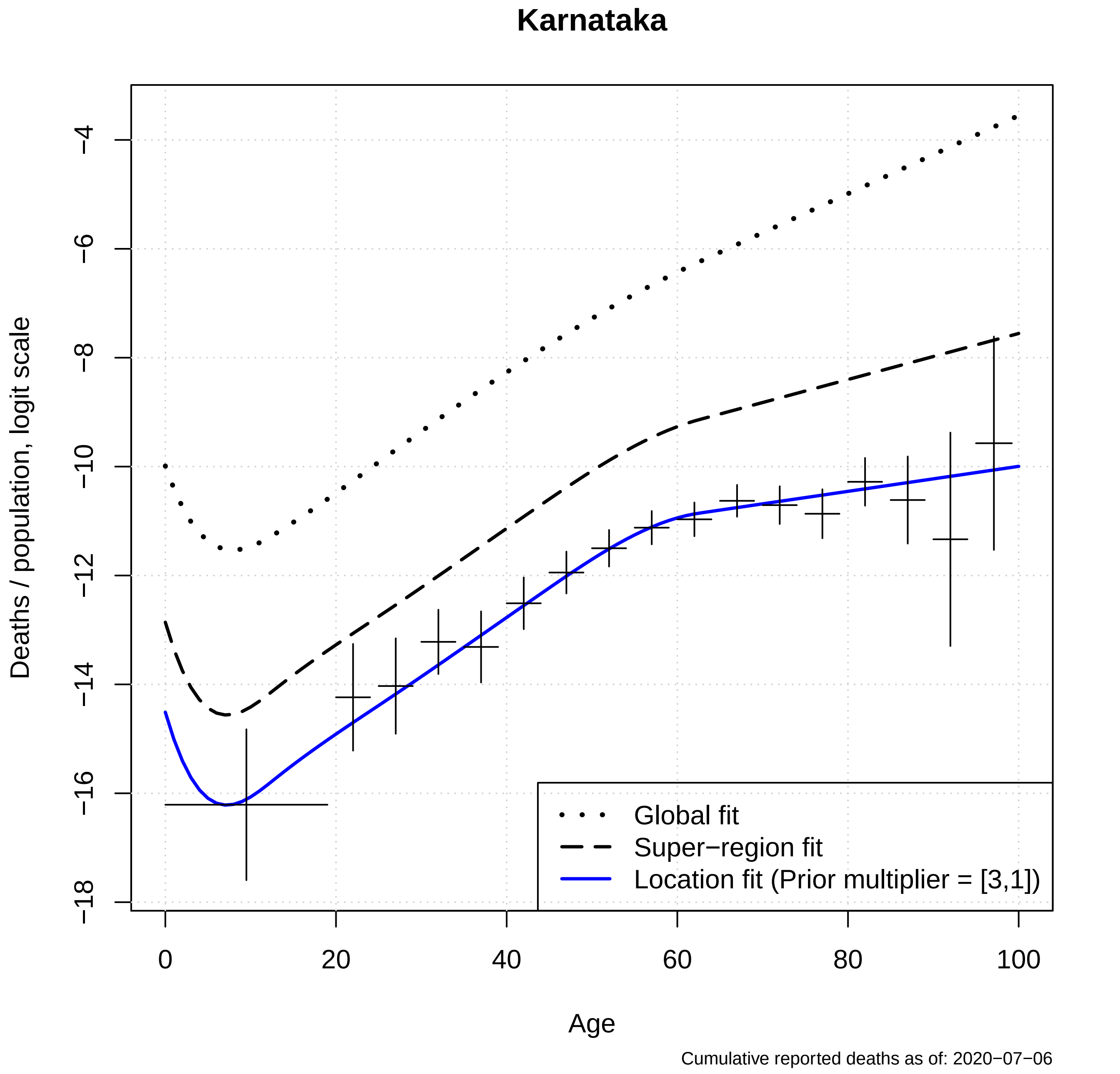
Karnataka
Deaths / population, logit scale
−4
−6
−8
−10
−12
−14
−16
−18
0
20
40
60
80
100
Age
Global fit
Super−region fit
Location fit (Prior multiplier = [3,1])
Cumulative reported deaths as of: 2020−07−06

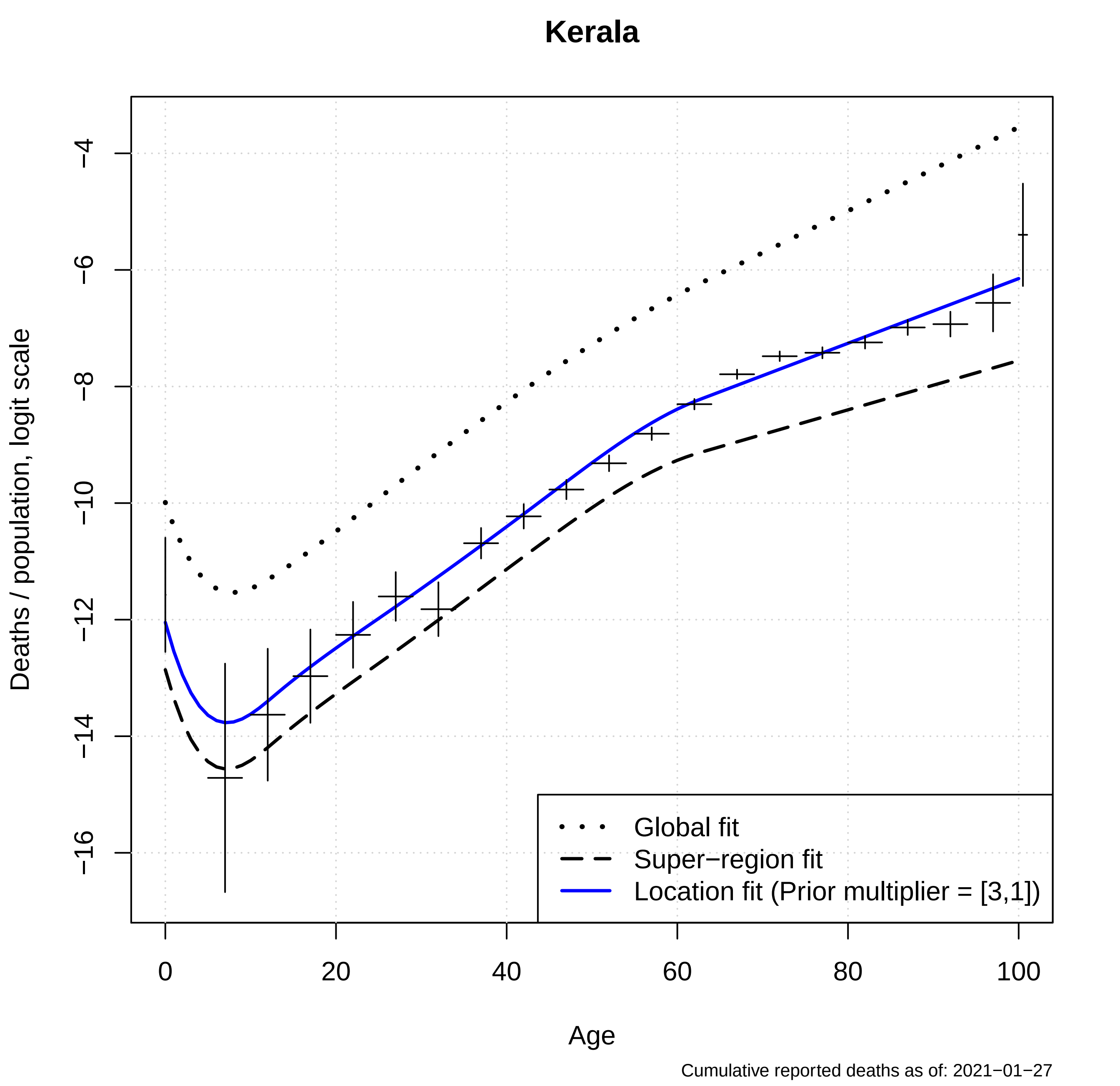

Kerala
Deaths / population, logit scale
−4
−6
−8
−10
−12
−14
−16
0
20
40
60
80
100
Age
Global fit
Super−region fit
Location fit (Prior multiplier = [3,1])
Cumulative reported deaths as of: 2021−01−27

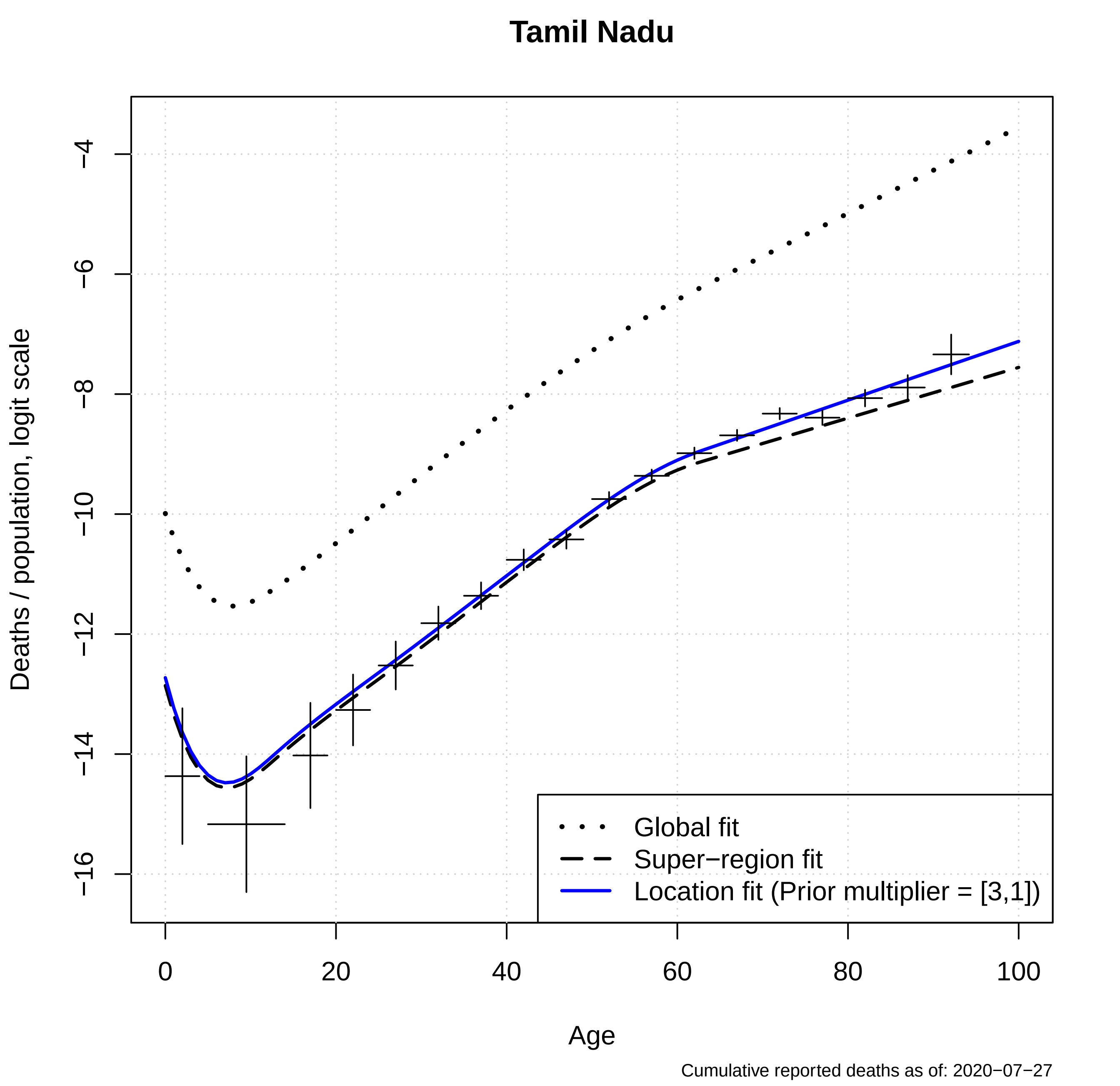

Tamil Nadu
Deaths / population, logit scale
−4
−6
−8
−10
−12
−14
−16
0
20
40
60
80
100
Age
Global fit
Super−region fit
Location fit (Prior multiplier = [3,1])
Cumulative reported deaths as of: 2020−07−27

Figure S8H. Canada subnationals

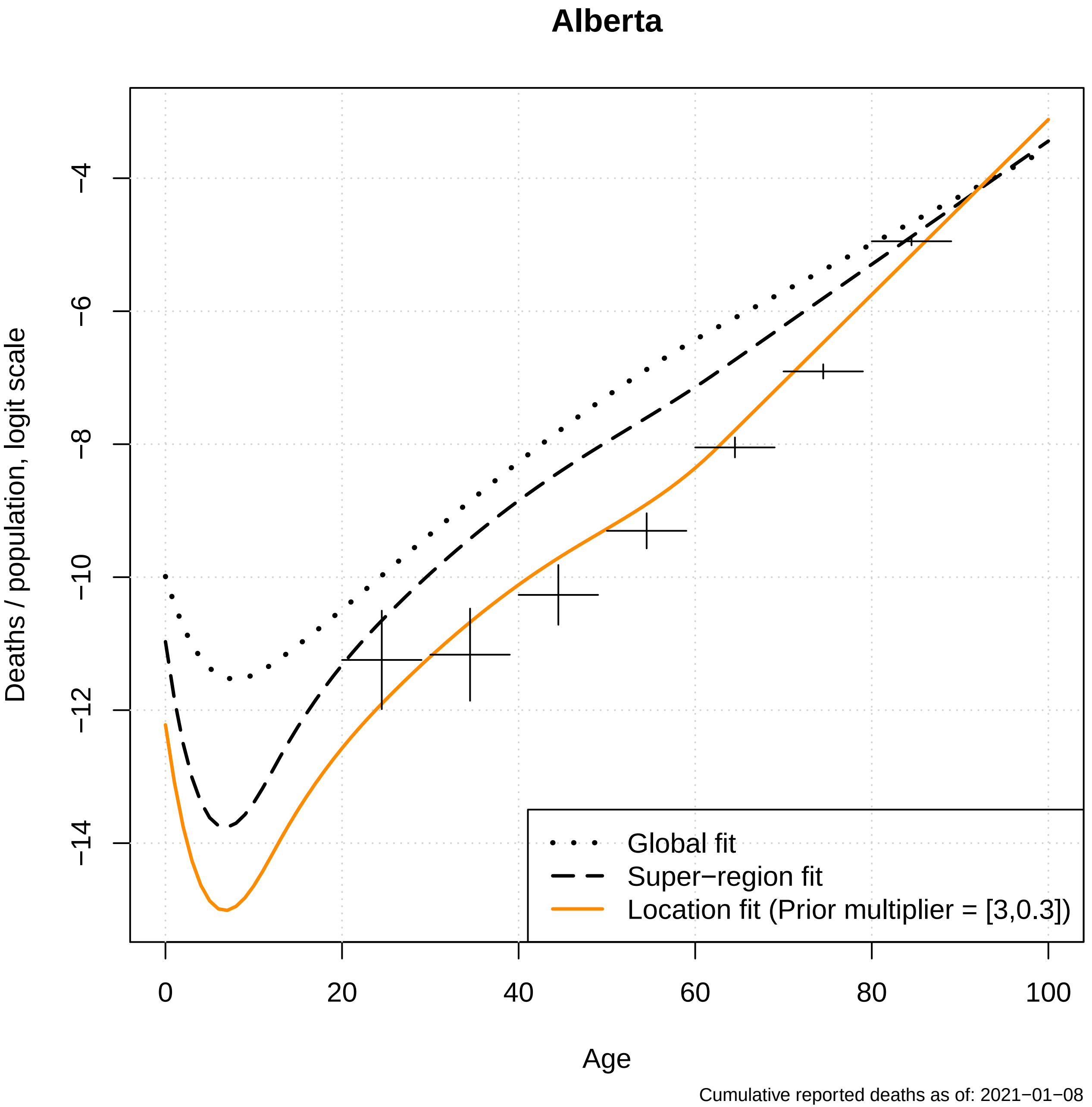

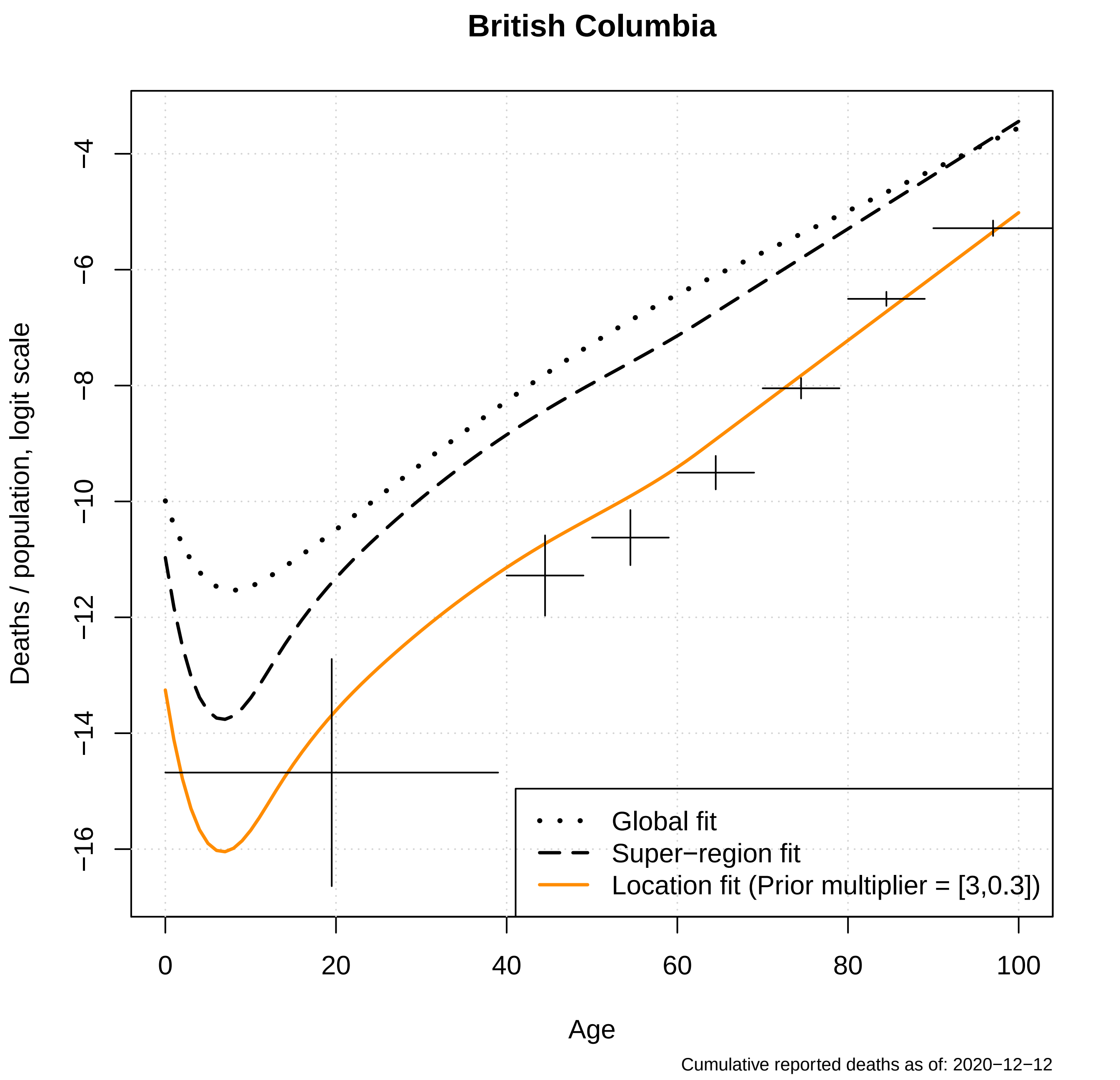

British Columbia
Deaths / population, logit scale
−4
−6
−8
−10
−12
−14
−16
0
20
40
60
80
100
Age
Global fit
Super−region fit
Location fit (Prior multiplier = [3,0.3])
Cumulative reported deaths as of: 2020−12−12

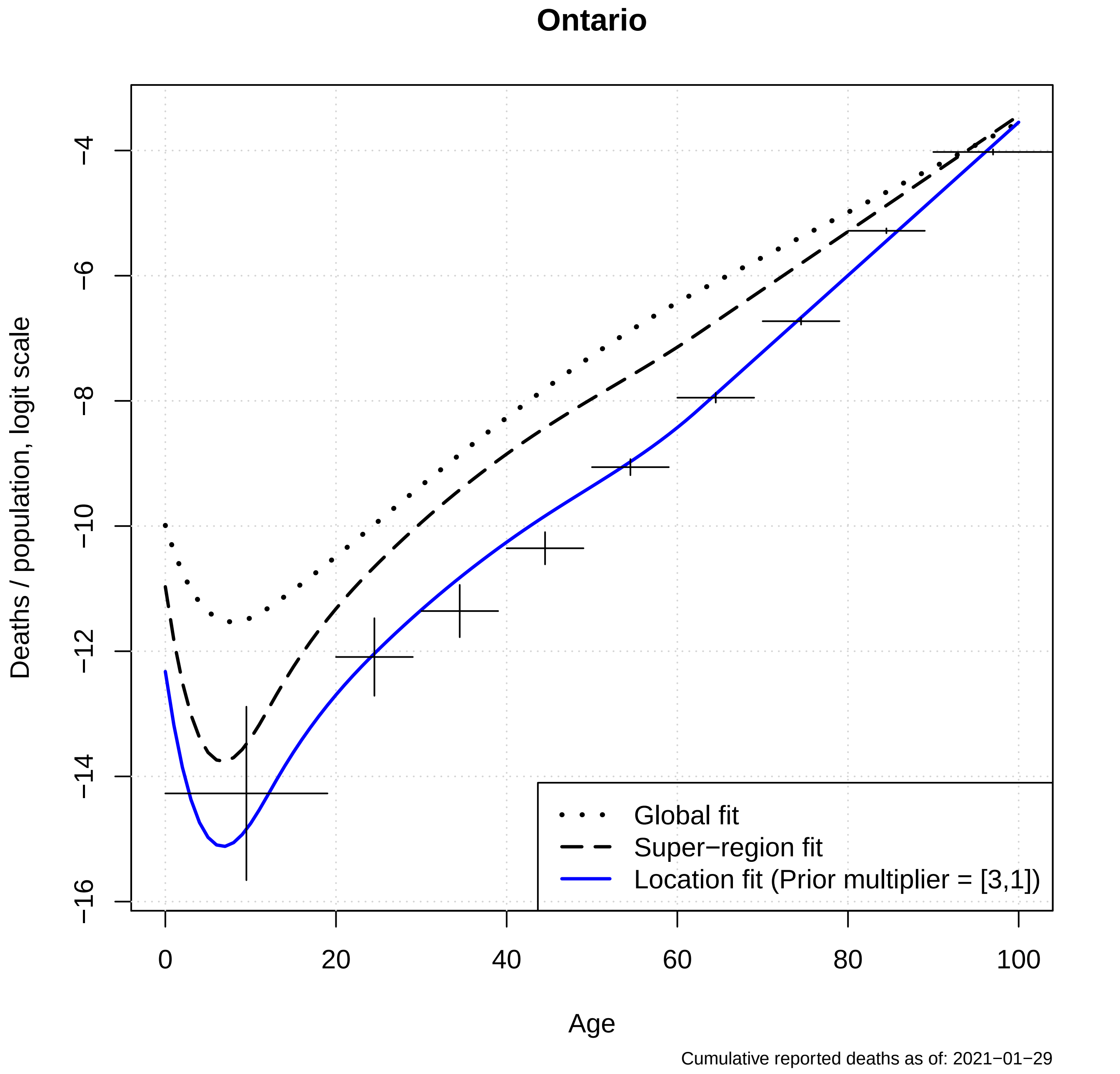
Ontario
Deaths / population, logit scale
−4
−6
−8
−10
−12
−14
−16
0
20
40
60
80
100
Age
Global fit
Super−region fit
Location fit (Prior multiplier = [3,1])
Cumulative reported deaths as of: 2021−01−29

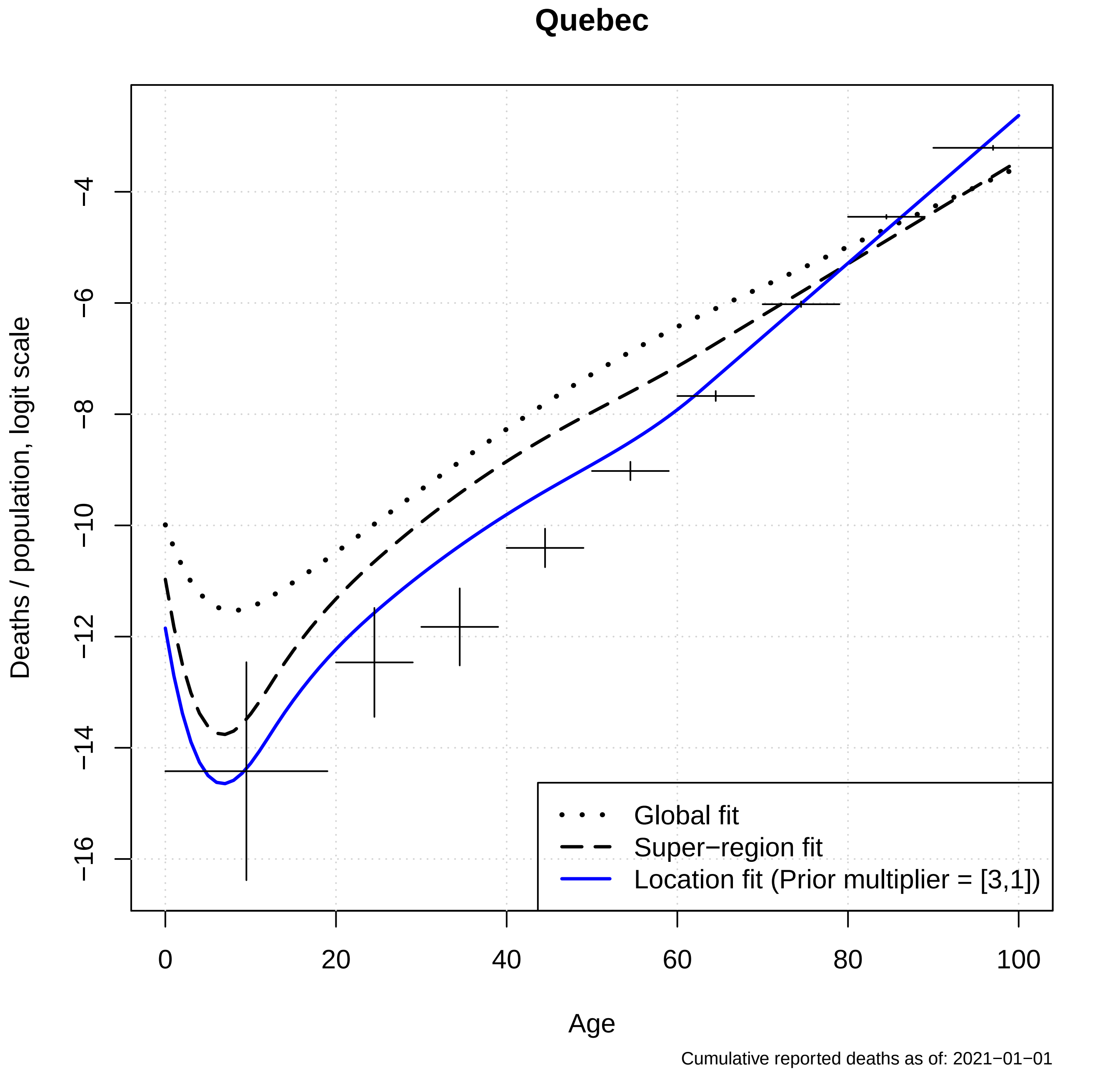
Quebec
Deaths / population, logit scale
−4
−6
−8
−10
−12
−14
−16
0
20
40
60
80
100
Age
Global fit
Super−region fit
Location fit (Prior multiplier = [3,1])
Cumulative reported deaths as of: 2021−01−01
